# Scrofimicrobium appendicitidis sp. nov., isolated from a patient with ruptured appendicitis

**DOI:** 10.1099/ijsem.0.006633

**Published:** 2025-01-21

**Authors:** Hiu-Yin Lao, Annette Y. P. Wong, Timothy Ting-Leung Ng, Ryan Yik-Lam Wong, Miranda Chong-Yee Yau, Jimmy Yiu-Wing Lam, Gilman Kit-Hang Siu

**Affiliations:** 1 Department of Health Technology and Informatics, The Hong Kong Polytechnic University, Hong Kong Special Administrative Region, Hong Kong, PR China; 2 Department of Clinical Pathology, Pamela Youde Nethersole Eastern Hospital, Hong Kong Special Administrative Region, Hong Kong, PR China

**Keywords:** peritoneal, ruptured appendicitis, *Scrofimicrobium appendicitidis *sp. nov.

## Abstract

A clinical isolate, R131, was isolated from the peritoneal swab of a patient who suffered from ruptured appendicitis with abscess and gangrene in Hong Kong in 2018. Cells are facultatively anaerobic, non-motile, Gram-positive coccobacilli. Colonies were small, grey, semi-translucent, low convex and alpha-haemolytic. The bacterium grew on blood agar but not on Brain Heart Infusion (BHI) and Mueller–Hinton agars. It was negative for catalase, oxidase, indole and aesculin hydrolysis. The initial identification attempts via matrix-assisted laser desorption ionization–time of flight mass spectrometry and 16S rRNA gene sequencing yielded inconclusive results. The 16S rRNA gene analysis showed that R131 shared >99% nucleotide identity with certain uncultured *Actinomycetales* bacteria. In this retrospective investigation, a complete genome of R131 was constructed, disclosing a DNA G+C content of 64%. Phylogenetic analysis showed that the bacterium was mostly related to *Scrofimicrobium canadense* WB03_NA08, which was first described in 2020. However, its 16S rRNA gene shared only 94.15% nucleotide identity with that of *S. canadense* WB03_NA08. Notably, the orthoANI between R131 and *S. canadense* WB03_NA08 was 67.81%. A pan-genome analysis encompassing R131 and 4 *Scrofimicrobium* genomes showed 986 core gene clusters shared with the *Scrofimicrobium* species, along with 601 cloud genes. The average nucleotide identity comparisons within the pan-genome analysis ranged from 59.78 to 62.51% between R131 and the other *Scrofimicrobium* species. Correspondingly, the dDDH values ranged from 19.20 to 22.30%, while the POCP values spanned from 57.48 to 60.94%. Therefore, a novel species, *Scrofimicrobium appendicitidis* sp. nov., is proposed. The type strain is R131^T^ (=JCM 36615^T^=LMG 33627^T^).

## Introduction

*Scrofimicrobium* is a relatively new genus first proposed by Wylensek *et al*. in 2020 [1]. According to the National Center for Biotechnology Information (NCBI) taxonomy database, the only one species under this genus is *Scrofimicrobium canadense* WB03_NA08, which was isolated from the pig intestine in Canada [1]. In the Genome Taxonomy Database (GTDB) (https://gtdb.ecogenomic.org/), three additional genomes are classified as *Scrofimicrobium*, including *Schaalia* sp. (GCF_014525425.1 and GCF_014069575.1) and *Actinomyces minihominis* (GCF_900187855.1). *Scrofimicrobium* belongs to the family *Actinomycetaceae*, which are facultative anaerobic Gram-positive rods. In this study, we described the isolation and characterization of a novel species in *Scrofimicrobium*, initially isolated from a patient with ruptured appendicitis in Hong Kong.

A 60-year-old Chinese man was admitted to the hospital in September 2018 because of right lower quadrant abdominal pain for 3 days and fever for 1 day. A contrast computed tomography scan of the abdomen and pelvis showed acute appendicitis with peri-appendiceal abscess formation. Laparoscopic appendicectomy with drainage of the appendiceal abscess was performed. The peritoneal swab was obtained, which later grew *Escherichia coli, Bacteroides thetaiotaomicron* and an unknown Gram-positive coccobacillus (bacterium R131). The patient was treated with intravenous amoxicillin-clavulanate and was later switched to intravenous ertapenem. He eventually recovered and was discharged after 15 days of hospitalization.

The bacterium, R131, appeared as non-motile Gram-positive coccobacilli under a microscope. It grew on horse blood agar as small, grey, semi-translucent, alpha-haemolytic colonies of 0.5–1.0 mm in diameter after 48 h incubation at 37 °C in ambient air. It also grew in an anaerobic environment. Matrix-assisted laser desorption ionization–time of flight mass spectrometry (MALDI-TOF MS) was used to identify the bacterium, but no reliable identification was obtained. Subsequent Nanopore 16S rRNA gene sequencing also failed to yield a conclusive identification until the discovery of *S. canadense* WB03_NA08. In this retrospective study, whole-genome sequencing (WGS) was performed to construct a complete genome of the bacterium. Phylogenetic analysis and pan-genome analysis showed that R131 was a novel species closely related to *S. canadense*.

## Methods

### Sample collection and extraction

The clinical isolate, R131, was obtained from the microbiology laboratory of Pamela Youde Nethersole Eastern Hospital. Upon receipt, DNA extraction was performed using QIAamp BiOstic Bacteremia DNA Kit.

### Phenotypic characteristics and biochemical properties

Gram stain was performed to examine the composition of the cell wall and the shape of bacterial cells. Transmission electron microscopy was performed to examine the bacterial cell using ThermoFisher Talos L120C Transmission Electron Microscope. MALDI-TOF MS-based identification was performed using Bruker IVD MALDI Biotyper. The biochemical properties of the R131 were determined using bioMérieux VITEK® 2 Systems coupled with VITEK® 2 Anaerobic and *Corynebacteria* identification card. Catalase and oxidase tests were also performed manually.

### Antimicrobial susceptibility test

The clinical isolate R131 was resuspended in saline with a concentration of 0.5 McFarland. The bacterial suspension was streaked evenly on blood Mueller–Hinton agars, and a maximum of six antibiotic discs were placed on the surface of an agar. A total of 14 Gram-positive spectrum antibiotics were tested, including ampicillin 10 µg (AMP 10), ceftriaxone 30 µg (CRO 30), tetracycline 30 µg (TE 30), vancomycin 30 µg (VA 30), gentamicin 10 µg (CN 10), meropenem 10 µg (MEM 10), cefoxitin 30 µg (FOX 30), chloramphenicol 30 µg (C 30), erythromycin 15 µg (E 15), co-trimoxazole 25 µg (SXT 25), ciprofloxacin 5 µg (CIP 5), ceftazidime 30 µg (CAZ 30), cefepime 30 µg (FEP 30) and clindamycin 2 µg (DA 2). The agar plates were incubated at 37 °C for 48 h aerobically with 5% CO_2_. The zone sizes were measured for the determination of antimicrobial susceptibility.

### Whole-genome sequencing

Both Nanopore sequencing and Illumina sequencing were used for WGS of R131. For Nanopore sequencing, library preparation was performed using the transpose-based rapid barcoding kit (SQK-RBK110.96) from Oxford Nanopore Technologies according to the manufacturer’s protocol. After pooling and adapter ligation, the library was loaded on the flow cell FLO-MIN106 R9.4.1 and sequenced using the GridION device for 48 h in high-accuracy base calling mode.

The high-accuracy Illumina sequencing was performed to further polish the nanopore reads. Library preparation was performed using NEBNext® Ultra™ II FS DNA Library Prep Kit coupled with NEBNext® Multiplex Oligos for Illumina® (96 Unique Dual Index Primer Pairs). The fragment size of the libraries was examined using Agilent 2100 Bioanalyzer instrument coupled with Agilent High Sensitivity DNA Kit. The libraries were quantified using Roche LightCycler® 480 System coupled with QIAseq Library Quant Assay Kit. After normalization, the pooled library was sequenced on Illumina Miseq system with MiSeq Reagent Kit V2.

### Genome assembly and taxonomic assignment

Both the Illumina sequences and the nanopore sequences were used for genome assembly. Before assembly, quality-control filtering using fastp [2] v0.22.0 for Illumina short reads and using Filtlong [3] v0.2.1 (with a minimum read length of 1 kbp and a kept-base percentage of 95%) for nanopore long reads were conducted. Then, a long-read-first approach using Trycycler v0.5.0 [4] was taken to assemble the genome. First, the long reads were divided into 12 subsets and assembled using Flye [5], minasm [6] + minipolish [7] and raven [8], making 12 different genome assemblies, with 1 assembly program assembling 4 sets of reads. Then, the 12 assemblies were merged into a single consensus assembly using Trycycler, and the single assembly was polished by Medaka v1.4.4 [9]. The polished long-read assembly was finally polished by short reads using Polypolish [10] followed by POLCA [11]. To avoid missing the small plasmids that may be underrepresented in the long-read sets, a short-read-first hybrid genome assembly was made using Unicycler v0.5.0 [12]. The quality and completeness of the genome assembly were evaluated using BUSCO v5.2.2 (bacteria_odb10) [13].

The taxonomy of R131 was assigned based on the 16S rRNA genes, the 23S rRNA genes and the marker genes across the whole genome. With full sequences of 16S rRNA genes and 23S rRNA genes predicted from the genome assembly, the taxonomy identity was assigned using silva ACT: Alignment, Classification and Tree Service (with sina 1.2.12 [14] and silva SSU and LSU databases 138.1 [15], default parameters). With the whole genome, the App ‘Classify Microbes with GTDB-Tk - v1.7.0’ [16] in KBase [17] was used for marker genes identification and taxonomic assignment. The degree of genomic similarity of the unknown bacterium with related species (species in the same genus, according to GTDB R08-RS214) was estimated using the Orthologous Average Nucleotide Identity Tool [18].

### Phylogenetic analysis of 16S rRNA gene

The full sequence of 16S rRNA predicted from the genome assembly of R131 was used for the analysis. The 16S rRNA gene sequence of *Scrofimicrobium* was downloaded from the NCBI server, and the aligned 16S rRNA gene sequences of a type strain from each of the other species in the family *Actinomycetaceae* and the outgroup *Escherichia coli* were downloaded from the silva RefNR SSU r138.1 database [15]. The 16S rRNA sequences of the unknown bacterium and *Scrofimicrobium* were aligned against the aligned sequences from the silva database using sina v1.7.2 [14]. RAxML v8.2.12 (with GTRGAMMA substitution model and a bootstrap of 800 replicates under the AutoMRE option) [19] was used to construct the phylogenetic tree. The resulting phylogenetic tree was visualized and annotated using the Interactive Tree of Life software (iTOL v6) [20].

### Phylogenomic analysis

The phylogenomic tree was inferred using the command ‘de_novo_wf’ of the Genome Taxonomy Database Toolkit (GTDB-Tk) v2.3.0 [21] based on the GTDB release 08-RS214.0 [22] (with ‘f__Actinomycetaceae’ as the taxa_filter and ‘s__Escherichia coli’ as the outgroup_taxon). The resulting phylogenomic tree was visualized and annotated using the iTOL v6 [20].

### Genome annotation and antimicrobial resistance prediction

Based on the classification that R131 was in the genus *Scrofimicrobium*, the genome was annotated using RASTtk [23] on the Rapid Annotation using Subsystem Technology (RAST) server [24] and the NCBI Prokaryotic Genome Annotation Pipeline (PGAP) build6771 [25]. The protein sequences encoded by the potential genes predicted from PGAP were further analysed by BlastKOALA [26] for functional annotation based on the Kyoto Encyclopedia of Genes and Genomes (KEGG) Orthology. The genome map was visualized using Proksee [27]. Antimicrobial resistance was predicted using ResFinder [28], ResFinderFG 2.0 [29] and AMRFinderPlus v3.11.26 [30,31].

### Pan-genome analysis

A total of four genomes were classified to the genus *Scrofimicrobium* based on GTDB, and their respective genomes were downloaded from NCBI. The genomes were annotated using PGAP [25]. Pan-genome analysis among the four genomes and R131 was conducted using get_homologues [32]. Clustering of orthologues genes was performed based on the OrthoMCL algorithm using the get_homologues.pl script, the average nucleotide identity (ANI) of the clustered sequences was also determined. Pan-genome matrix was calculated using the compare_clusters.pl script. The number of core genes, soft-core genes, shell genes and cloud genes was estimated using the parse_pangenome_matrix.pl script. A pangenome tree was illustrated using IQ-TREE 2 [33] and visualized using FigTree (v1.4.4). Core genes were annotated using BlastKOALA [26] with ‘Prokaryotes’ database.

### Digital DNA–DNA hybridization analysis

Genome similarity between R131 and the four *Scrofimicrobium* genomes was also evaluated using digital DNA–DNA hybridization (dDDH) values. The nucleotide sequences of the five genomes were submitted to Genome-to-Genome Distance Calculator (GGDC 3.0) (http://ggdc.dsmz.de/ggdc.php) [34] for analysis. While a dDDH value of 70% defines species boundaries, a value of 79% delimits subspecies [35].

### Percentage of conserved protein analysis

Percentage of conserved protein (POCP) analysis was performed to estimate the genome similarity between two microbial genomes based on the proportion of shared proteins. A threshold of 50% indicates that two strains belong to the same genus [36]. POCP values among R131, the four *Scrofimicrobium* genomes based on GTDB and the type species of *Actinomyces* (*Actinomyces bovis* NCTC 11535) and *Schaalia* (*Schaalia odontolytica* NCTC9935) were calculated using POCP-nf (https://github.com/hoelzer/pocp) [37]. Briefly, protein annotation was conducted using Prokka [38], followed by an ‘all-vs-all’ comparison of proteins using blastp [39].

## Results

### Phenotypic characteristics, biochemical properties and antimicrobial susceptibility profile

**Fig. 1.**
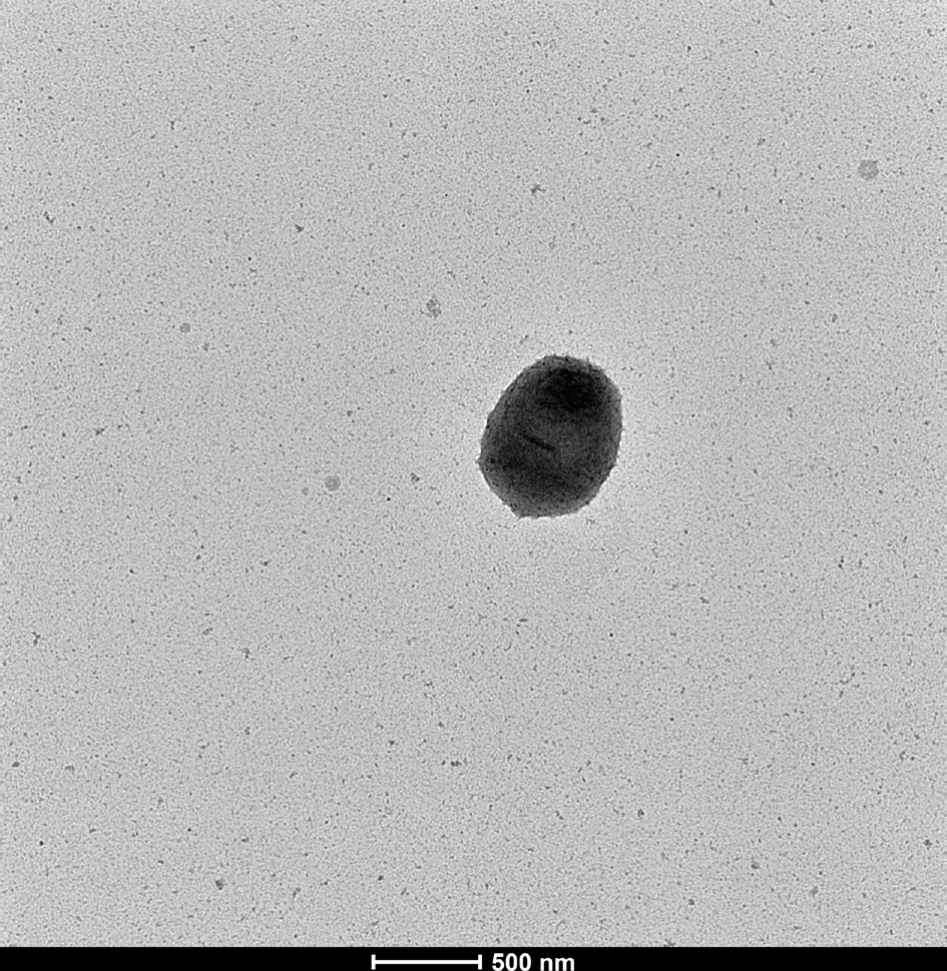
Transmission electron graph of R131.

R131 was non-motile Gram-positive coccobacilli. The bacterium was facultative anaerobe, which can be grown on blood agar anaerobically or aerobically (with or without 5% CO_2_) at 37 °C. Small (0.5–1.0 mm), grey, semi-translucent and alpha-haemolytic colonies were observed on the blood agar after 48-h incubation (Table S1, available in the online Supplementary Material). The cell size of the strain was about 0.5–0.6 µm wide and 0.7–1.0 µm long (Fig. 1).

The biochemical properties of the R131 are summarized in Table 1. The bacterium can utilize d-cellobiose, d-galactose, d-glucose, d-mannose, d-ribose, l-arabinose, d-xylose and maltotriose as sole carbon source. However, unlike its closely related species, *Scrofimicrobium canadense* WB03_NA08, it cannot utilize d-maltose. It also cannot utilize sucrose. The bacterium does not produce the enzymes catalase, oxidase, and phosphatase. Nevertheless, it produces urease, cholinesterase and phenylphosphonate hydrolase, distinguishing it from the urease-negative *S. canadense* WB03_NA08. It also possesses several glycosidases, including beta-glucuronidase, alpha-arabinosidase, alpha-l-fucosidase, beta-d-fucosidase and beta-galactosidase. Additionally, it harbours several arylamidases, including Ala-Phe-Pro-arylamidase, leucine arylamidase, l-proline arylamidase, phenylalanine arylamidase and tyrosine arylamidase. The bacterium is incapable of hydrolysing aesculin, arginine and tryptophan.

**Table 1.** The biochemical properties R131 and *S. canadense* WB03_NA08 In this table, + indicates positive, − indicates negative and +/− indicates weak reactions.

	Biochemical tests	R131	*S. canadense*WB03_NA08
Carbon source utilization	d-Cellobiose	**+**	**+**
	d-Galactose	**+**	
	d-Glucose	**+**	**+**
	d-Maltose	−	**+**
	d-Mannose	**+**	**+**
	d-Ribose	**+**	
	d-Xylose	**+**	**+**
	l-Arabinose	**+**	**+**
	Maltotriose	**+**	
	*N*-Acetyl-d-glucosamine	**+**	
	Pyruvate	**+**	
	Saccharose/sucrose	−	**+/-**
Glycosidase tests	5-Bromo-4-chloro-3-indoxyl-alpha-galactoside	−	
	5-Bromo-4-chloro-3-indoxyl-alpha-mannoside	−	
	5-Bromo-4-chloro-3-indoxyl-beta-glucoside	−	
	5-Bromo-4-chloro-3-indoxyl-beta-glucuronide	**+**	
	5-Bromo-4-chloro-3-indoxyl-beta-N-acetyl-glucosamide	−	
	Alpha-Arabinosidase	**+**	
	Alpha-l-Arabinofuranoside	−	
	Alpha-l-Fucosidase	**+**	
	Arbutin	−	
	Beta-d-Fucosidase	**+**	
	Beta-Galactopyranosidase indoxyl	**+**	
	Beta-Mannosidase	−	
Arylamidase tests	Ala-Phe-Pro-Arylamidase	**+**	
	Leucine arylamidase	**+**	
	l-Proline arylamidase	**+**	
	l-Pyrrolidonyl-arylamidase	−	
	Phenylalanine arylamidase	**+**	
	Tyrosine arylamidase	**+**	
Enzyme production tests	Urease	**+**	−
	Catalase	−	
	Oxidase	−	
	Ellman (cholinesterase)	**+**	
	Phosphatase	−	
	Phenylphosphonate	**+**	
Others	Arginine	−	
	Aesculin hydrolysis	−	**+/−**
	Indole	−	−

The susceptibility of R131 to 14 Gram-positive spectrum antibiotics was tested using the disc diffusion method. R131 was susceptible to all 14 antibiotics, including ampicillin (AMP 10), ceftriaxone (CRO 30), tetracycline (TE 30), vancomycin (VA 30), gentamicin (CN 10), meropenem (MEM 10), cefoxitin (FOX 30), chloramphenicol (C 30), erythromycin (E 15), co-trimoxazole (SXT 25), ciprofloxacin (CIP 5), ceftazidime (CAZ 30), cefepime (FEP 30) and clindamycin (DA 2) (Table 2).

**Table 2.** Antimicrobial susceptibility profile of R131 to 14 antibiotics

Drug name	Zone diameter (mm)	Inferred susceptibility
Ampicillin (AMP 10)	53	Susceptible
Ceftriaxone (CRO 30)	44	Susceptible
Tetracycline (TE 30)	47	Susceptible
Vancomycin (VA 30)	34	Susceptible
Gentamicin (CN 10)	28	Susceptible
Meropenem (MEM 10)	38	Susceptible
Cefoxitin (FOX 30)	43	Susceptible
Chloramphenicol (C 30)	38	Susceptible
Erythromycin (E 15)	32	Susceptible
Co-trimoxazole (SXT 25)	27	Susceptible
Ciprofloxacin (CIP 5)	20	Susceptible
Ceftazidime (CAZ 30)	18	Susceptible
Cefepime (FEP 30)	39	Susceptible
Clindamycin (DA 2)	15	Susceptible

### The quality of the assembly and the genome characteristics

**Fig. 2.**
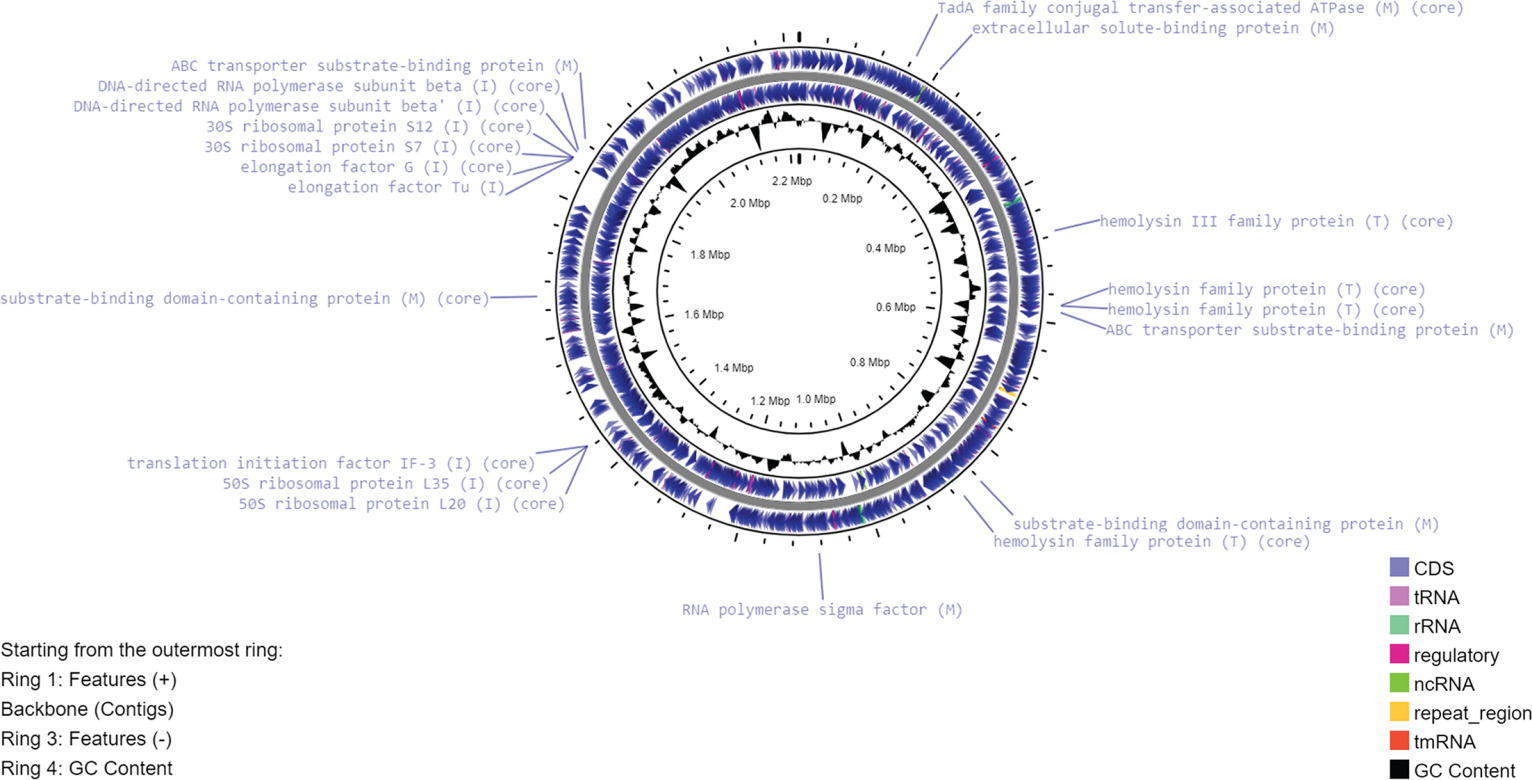
Genetic map of the chromosome of R131. Ring 1 and Ring 3 show the features annotated using PGAP. The innermost ring is a plot of G+C content across the genome. Selected CDS features are labelled with the name of the protein products of the genes. Each protein label is associated with a letter in a pair of brackets, with the letter ‘I’ representing the subcategory ‘Invasion and intracellular resistance’ by RAST, ‘T’ representing the Brite ‘Bacterial toxins’ and ‘M’ representing the Brite ‘Bacterial motility proteins’ or the ‘Cell motility’ KEGG pathways by BlastKOALA. The protein labels associated with the word ‘core’ are the core genes from the pan-genome analysis.

A complete circular chromosome of size around 2.22 Mbps was assembled (CP138335). The Unicycler hybrid assembly did not recover any small plasmid. The BUSCO analysis shows that the genome assembly contains 122 complete and single-copy BUSCOs (98.4%) and two fragmented BUSCOs (1.6%), indicating a high level of completeness of the assembly. The G+C content of the genome is 64%. Annotation using RASTtk identifies a total of 2054 features, including 2001 coding sequences and 53 RNAs. Twenty-eight per cent of the genes were assigned to subsystems by RASTtk. Nineteen genes were assigned to the subsystem ‘Virulence, Disease and Defense’, with ten genes involved in the resistance to antibiotics and toxic compounds and nine genes involved in the invasion and intracellular resistance. Annotation using PGAP identifies a total of 2027 genes/pseudogenes, including 1970 CDSs and 57 RNAs. Among the 1957 protein sequences encoded by the non-pseudo protein-coding genes analysed by BlastKOALA, 60.3% were annotated. The KEGG Mapper Reconstruction results from BlastKOALA include 198 pathways, 36 BRITE categories and 33 complete modules. The genetic map of the circular chromosome of R131, based on the annotation of PGAP, with the labelling of selected features from RAST and BlastKOALA annotations, is depicted in Fig. 2. Despite there are genes predicted to be involved in the resistance to antibiotics and toxic compounds by RAST and genes assigned to the KEGG pathways and Brite categories of antimicrobial resistance, there is no complete KEGG module for antimicrobial resistance. Moreover, the analyses using ResFinder, ResFinderFG 2.0 and AMRFinderPlus predicted no antimicrobial resistance in R131. It is concluded that R131 is susceptible to most of the common antibiotics based on bioinformatics prediction. This is consistent with the results of the antimicrobial susceptibility test.

### Phylogenetic and phylogenomic analyses

The bacterium, R131, remained ‘unclassified’ when being classified using 23S rRNA gene sequence against the silva LSU database and classified as belonging to the genus *Actinomyces* using 16S rRNA gene sequence (OR652275) against the silva SSU database. Using GTDB-Tk in KBase, R131 was assigned as a member of the genus *Actinomyces_I* in GTDB release 202 (this genus was later updated to ‘*Scrofimicrobium*’ in GTDB release 207). Despite these taxonomic assignments from silva ACT and GTDB-Tk, the specific species of R131 could not be determined.

**Fig. 3.**
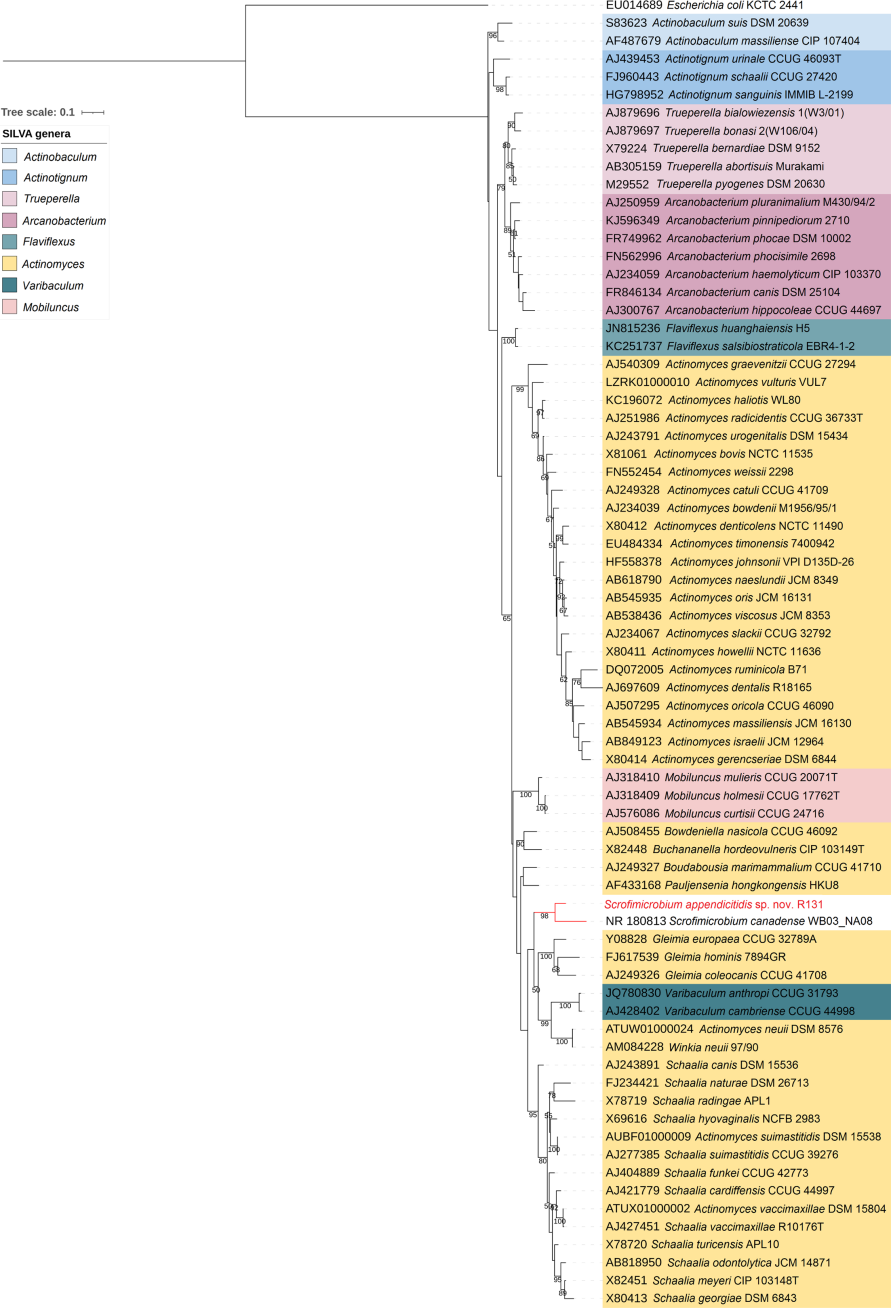
Phylogenetic tree of the family *Actinomycetaceae* based on 16S rRNA gene sequences. The numbers on branches denote the bootstrap values ≥50%. The colour ranges represent the genera in the family *Actinomycetaceae* according to the silva database classification in release 138.1. *Escherichia coli* was used as the outgroup.

Given that both tools recognized R131 as part of the *Actinomycetaceae* family, the phylogenetic and phylogenomic analyses focused on comparing R131 with other *Actinomycetaceae* members. The bacterium was grouped with *S. canadense* WB03_NA08 in the 16S rRNA gene tree (Fig. 3). Currently, the widely accepted thresholds for taxonomic classification based on 16S rRNA gene sequence similarity are set at 98.7% for species, 94.5% for genera and 86.5% for families [40]. Notably, the 16S rRNA gene sequences of R131 and *S. canadense* WB03_NA08 shared 94.15% nucleotide identity, bordering on the lower limit of the genus differentiation threshold.

**Fig. 4.**
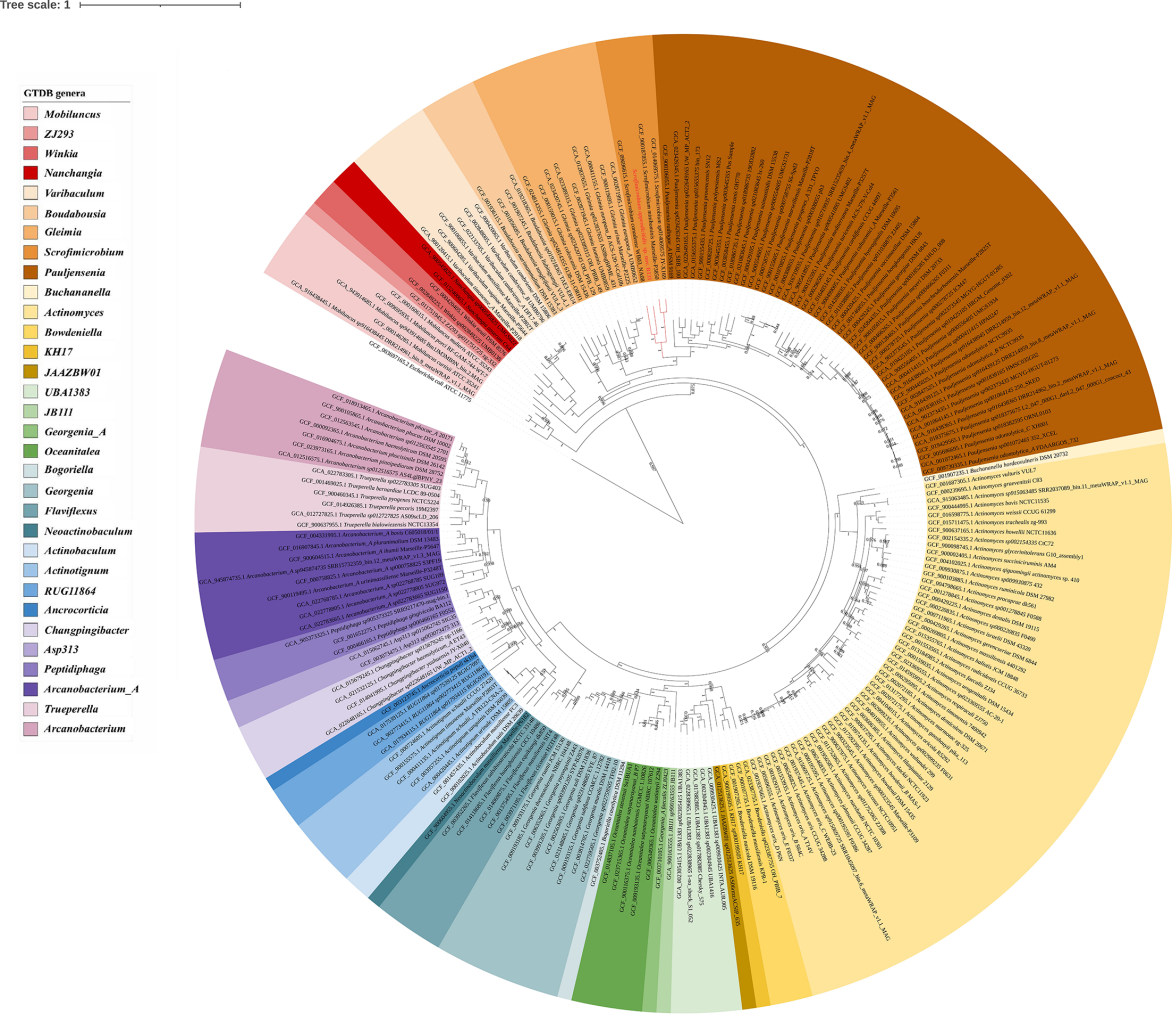
Phylogenomic tree of the family *Actinomycetaceae* based on the 120 phylogenetically informative markers protein of bacteria (bac120) in GTDB. The tree was inferred by the approximately maximum likelihood method using FastTree embedded GTDBTk. The numbers on branches denote the SH-like supports ≥0.5. The colour ranges represent the genera in the family *Actinomycetaceae* according to the GTDB R08-RS214.0. *Escherichia coli* was used as the outgroup. Accession numbers of the genomes used and the species names are given in the leaf labels. The leaf label of R131 and branches of the clade of its predicted genus are shown in red.

In the phylogenomic study, R131 was also clustered with the members in the genus of *Scrofimicrobium* (Fig. 4). The OrthoANI values between the genome of R131 and those of other *Scrofimicrobium* members range from 67.1 to 69.1%, falling below the 95% threshold for species delineation [40]. However, there is no standard ANI threshold for genus delimitation [36]. These findings collectively indicate that R131 represents a novel species, which closely related to the genus *Scrofimicrobium*.

### Pan-genome analysis

Four genomes, which were *Actinomyces* (*Scrofimicrobium*) *minihominis* Marseille-P3850 (GCF_900187855.1), *Schaalia* (*Scrofimicrobium*) sp. JY-X159 (GCF_014525425.1), *Schaalia* (*Scrofimicrobium*) sp. JY-X169 (GCF_014069575.1) and * S. canadense* WB03_NA08 (GCF_009696615.1), were analysed with R131 for pan-genome analysis. A total of 3994 gene clusters were identified, and the 5 genomes shared 986 core gene clusters (Table 3). A representative sequence in R131 from each of the core gene clusters was annotated using BlastKOALA, and the result was summarized in Table S2. The cloud genes, which are the genes present in two or less of the genomes, ranged from 361 to 1105 genes. The ANI values of clustered sequences (Table 4) and the phylogenetic tree (Fig. S1) showed that R131 was distant from the other four genomes, proving that it is a novel species in the genus *Scrofimicrobium*.

**Table 3.** Number of gene clusters in the five genomes

	Cloud	Shell	Soft-core	Core
*S. canadense* WB03_NA08	1105	114	1155	986
R131	601	109	1190	986
*S. minihominis* Marseille-P3850	361	204	1175	986
*Scrofimicrobium* sp. JY-X159	434	220	1209	986
*Scrofimicrobium* sp. JY-X169	467	250	1245	986

**Table 4.** ANI values of the clustered sequences in the five genomes

	*S. canadense* WB03_NA08	R131	*S. minihominis*Marseille-P3850	*Scrofimicrobium* sp. JY-X159	*Scrofimicrobium* sp. JY-X169
*S. canadense* WB03_NA08	100	62.51	59.7	59.34	58.68
R131	62.51	100	60.13	60.31	59.78
*S. minihominis*Marseille-P3850	59.7	60.13	100	74.91	74.51
*Scrofimicrobium* sp. JY-X159	59.34	60.31	74.91	100	96.82
*Scrofimicrobium* sp. JY-X169	58.68	59.78	74.51	96.82	100

### dDDH analysis

The dDDH values for R131 and four *Scrofimicrobium* genomes are summarized in Table 5. When compared with the four genomes, R131 exhibited a dDDH value below 70%, indicating that R131 represents a distinct species from *S. canadense* WB03_NA08, * S. minihominis* Marseille-P3850, *Scrofimicrobium* sp. JY-X159 and *Scrofimicrobium* sp. JY-X169. Additionally, the dDDH value of 84.1% between *Scrofimicrobium* sp. JY-X159 and *Scrofimicrobium* sp. JY-X169 indicated that they belong to the same species.

**Table 5.** dDDH values obtained through a comparison of the five genomes using GGDC 3.0, formula 2 (DDH calculated based on identities/HSP length)

	*S. canadense*WB03_NA08	*S. minihominis*Marseille-P3850	*Scrofimicrobium* sp. JY-X159	*Scrofimicrobium* sp. JY-X169
R131	22.20%	19.20%	19.70%	22.30%
*S. canadense* WB03_NA08		20.90%	20.20%	21.50%
*S. minihominis*Marseille-P3850			19.00%	19.20%
*Scrofimicrobium* sp. JY-X159				84.1%

### POCP analysis

Given that ANI and dDDH values are typically utilized for species delineation without providing clear indications for genus delimitation, a POCP analysis was conducted to ascertain the genus affiliation of the five genomes. Notably, discrepancies in genus assignments between NCBI and GTDB for *Actinomyces* (*Scrofimicrobium*) *minihominis* Marseille-P3850, *Schaalia* (*Scrofimicrobium*) sp. JY-X159 and *Schaalia* (*Scrofimicrobium*) sp. JY-X169 prompted the inclusion of the type species of *Actinomyces* and *Schaalia*, namely *A. bovis* NCTC 11535 (GCF_900444995.1) and *S. odontolytica* NCTC9935 (GCF_900445025.1), respectively, in the POCP analysis.

The outcomes of the POCP analysis, detailed in Table 6, revealed that R131 exhibited a POCP value exceeding 50% when compared against the four *Scrofimicrobium* genomes, signifying their shared genus classification. In contrast, the POCP value between *A. bovis* NCTC 11535 and *Actinomyces* (*Scrofimicrobium*) *minihominis* Marseille-P3850, as well as those between *S. odontolytica* NCTC9935 and the two *Schaalia* (*Scrofimicrobium*) species (JY-X159 and JY-X169), fell below the 50% threshold. Considering the results of phylogenomic study (Fig. 4) and the POCP analysis, it is proposed to reclassify *A. minihominis* Marseille-P3850 to *S. minihominis* Marseille-P3850 and to designate *Schaalia* species JY-X159 and JY-X169 as *Scrofimicrobium* species.

**Table 6.** POCP values of the studied genomes

	*S. canadense* WB03_NA08	R131	*S. minihominis*Marseille-P3850	*Scrofimicrobium* sp. JY-X159	*Scrofimicrobium* sp. JY-X169	*A. bovis* NCTC 11535	*S. odontolytica* NCTC 9935
*S. canadense* WB03_NA08	100	57.48	53.86	50.96	51.38	38.22	42.50
R131	57.48	100	60.94	58.82	59.12	44.13	49.16
*S. minihominis*Marseille-P3850	53.86	60.94	100	71.95	71.12	40.81	44.22
*Scrofimicrobium* sp. JY-X159	50.96	58.82	71.12	100	87.17	40.73	45.26
*Scrofimicrobium* sp. JY-X169	51.39	59.12	71.95	87.17	100	39.84	44.21
*A. bovis* NCTC 11535	38.22	44.13	40.81	40.73	39.84	100	47.44
*S. odontolytica* NCTC 9935	42.50	49.16	44.22	45.26	44.21	47.44	100

A POCP value below the threshold of 50% was highlighted in red, suggesting that the two strains belong to distinct genera.

### Description of *Scrofimicrobium appendicitidis* sp. nov.

*Scrofimicrobium appendicitidis* (ap.pen.di.ci'ti.dis. N.L. gen. n. *appendicitidis*, of appendicitis). The closest phylogenetic neighbour is *S. canadense* WB03_NA08, which shares 94.15% 16S rRNA gene sequence identity. OrthoANI, dDDH and POCP values against the genome of *S. canadense* WB03_NA08 are 67.81, 22.20 and 57.48%, respectively. Cells are facultatively anaerobic, non-motile, Gram-positive coccobacilli (~0.5–0.6 µm wide and 0.7–1.0 µm long). Cells grow on blood agar at 37 °C in ambient air after 48-h incubation, with or without 5% CO_2_. Cells also grow on anaerobic blood agar under anaerobic environments. Requires blood to grow, unable to grow on Brain Heart Infusion (BHI) agar and Mueller–Hinton agar. Colonies are small (0.5–1.0 mm), grey, semi-translucent, low convex and alpha-haemolytic. Utilizes d-cellobiose, d-galactose, d-glucose, d-mannose, d-ribose, l-arabinose, d-xylose, and maltotriose, but not d-maltose and sucrose. Produces urease, cholinesterase, phenylphosphonate hydrolase, beta-glucuronidase, alpha-arabinosidase, alpha-l-fucosidase, beta-d-fucosidase, beta-galactosidase, Ala-Phe-Pro-arylamidase, leucine arylamidase, l-proline arylamidase, phenylalanine arylamidase and tyrosine arylamidase. Does not produce catalase, oxidase and phosphatase. Cannot hydrolyse aesculin, arginine and tryptophan. Susceptible to ampicillin (AMP 10), ceftriaxone (CRO 30), tetracycline (TE 30), vancomycin (VA 30), gentamicin (CN 10), meropenem (MEM 10), cefoxitin (FOX 30), chloramphenicol (C 30), erythromycin (E 15), co-trimoxazole (SXT 25), ciprofloxacin (CIP 5), ceftazidime (CAZ 30), cefepime (FEP 30) and clindamycin (DA 2).

The type strain, R131^T^ (=JCM 36615^T^=LMG 33627^T^), was isolated from the peritoneal swab of a patient with ruptured appendicitis in Hong Kong, China. The G+C content of the genomic DNA of the type strain is 64%.

The 16S rRNA gene and genome were deposited in GenBank under accession numbers OR652275 and CP138335, respectively.

## Supplementary material

10.1099/ijsem.0.006633 Uncited Supplementary Material 1.

## Abbreviations

ANI: average nucleotide identity
BHI: brain heart infusion
dDDH: digital DNA–DNA hybridization
GGDC: genome-to-genome distance calculator
GTDB: genome taxonomy database
GTDB-Tk: genome taxonomy database toolkit
iTOL: interactive tree of life software
KEGG: kyoto encyclopedia of genes and genomes
MALDI-TOF MS: matrix-assisted laser desorption ionization–time of flight mass spectrometry
NCBI: National Center for Biotechnology Information
PGAP: prokaryotic genome annotation pipeline
POCP: percentage of conserved protein
RAST: Rapid Annotation using Subsystem Technology
WGS: whole-genome sequencing
