# Supplementary material for: Scrofimicrobium appendicitidis sp. nov., isolated from a patient with ruptured appendicitis
Source: Int J Syst Evol Microbiol. 2025 Jan 21;75(1):006633. doi: 10.1099/ijsem.0.006633 (PMC12281968; doi:10.1099/ijsem.0.006633)
Supplement: Uncited Supplementary Material 1. [file ijsem-75-06633-s001.pdf]

Supplementary Table 1. Colony, cell morphology, and MALDI-TOF-MS peptide mass fingerprint (PMF) of R131.

Colony morphology on anaerobic blood agar	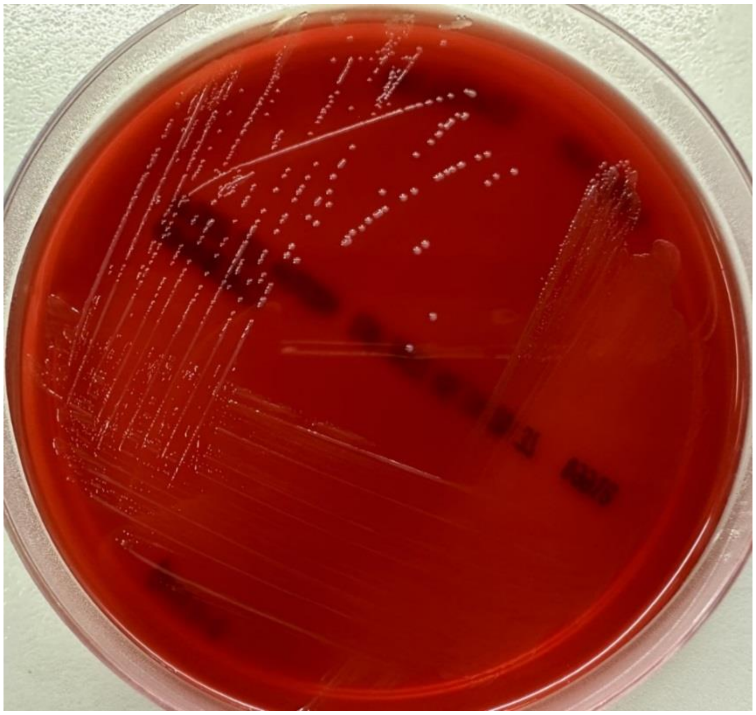
Alpha-haemolysis on blood agar	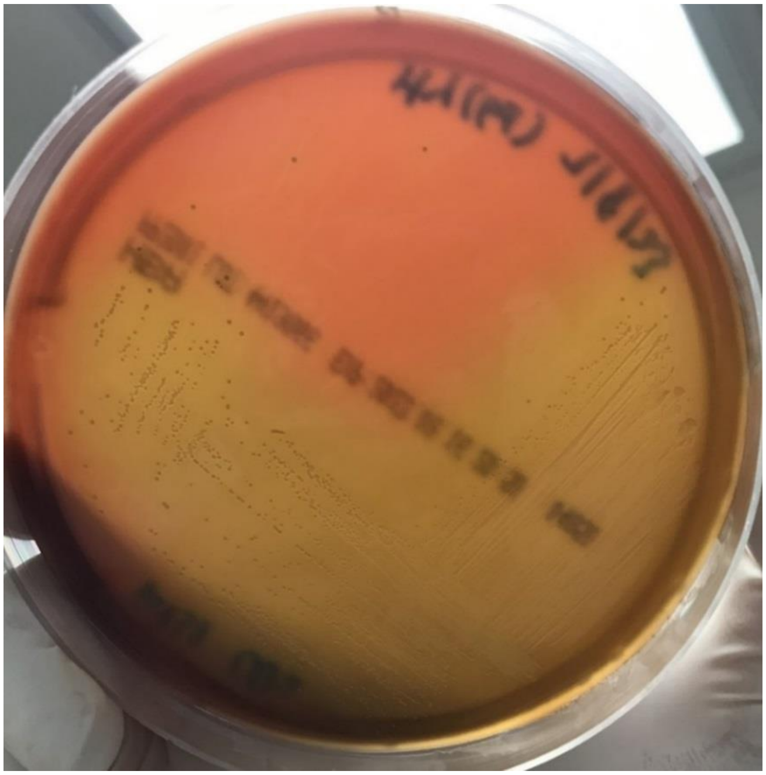

Gram stain	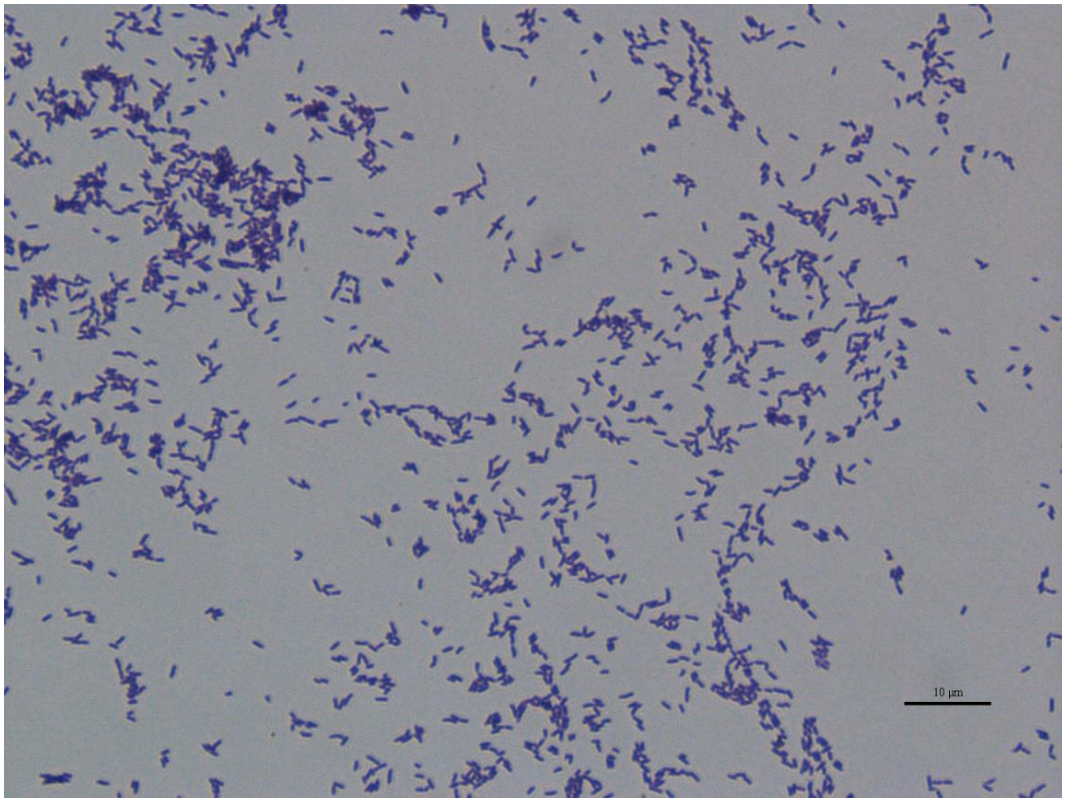
MALDI-TOF MS peptide mass fingerprint (PMF)	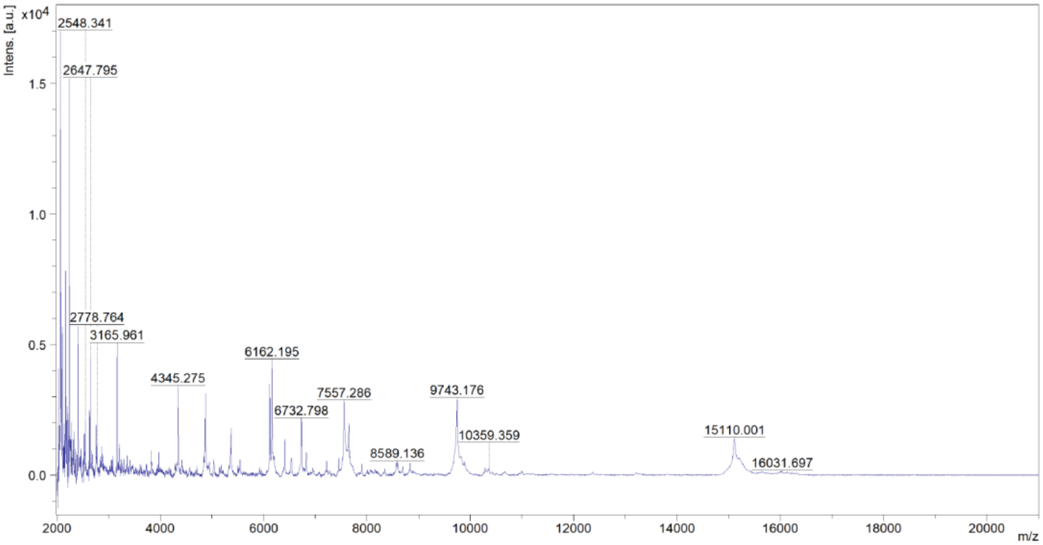

Supplementary Figure 1. The pan-genomic phylogenetic tree of the 5 genomes

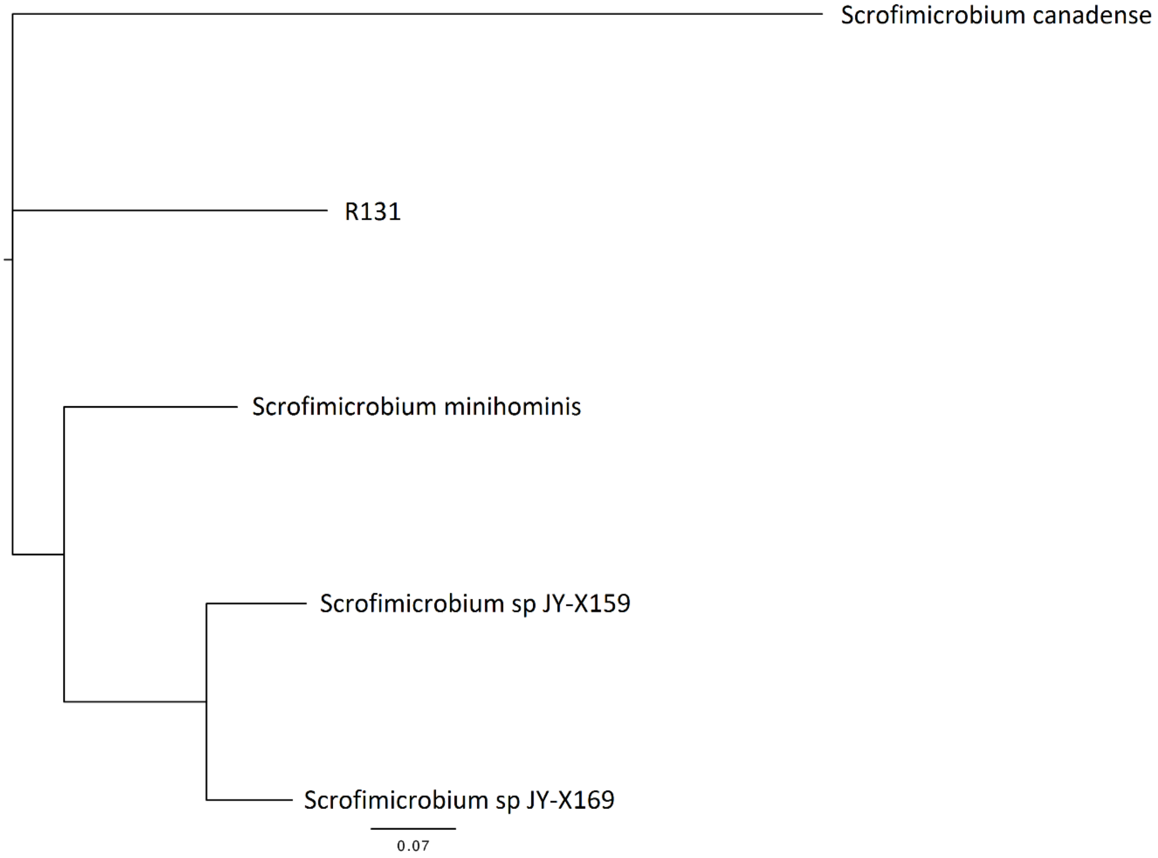

Supplementary Table 2 GhostKOALA annotated core gene clusters			
**Query**	**KO**	**Definition**	**Protein sequence**
gnl|extdb|pgaptmp_001579			MSENQLALKTVIHEIERGAARLGWDRPPAIYALVPTQELLAAPDLPADIAEQLRESWTGEAEHLSAILQDPLAEDNLEEILPELAWPDQVAGAAVAVERAIIPPEVEDQVPEDPHEAAHFVASHPDHTDVRLTVGVLRSGESWCAIRTRPFDSDDQVGSGENLIPGLVELLRLGFVEAEPTAD
gnl|extdb|pgaptmp_001580	K07177	Lon-like protease	MEKTSQPNRLKRALQWLVLIVVAVGLGLAVSLTPLPFVIYSPGPTFDVLGDQDGQPLIEIRGSDEPAEGELRMVTVSEEGGPGTTVTAPMLLRAWRTPGYSISRYSDVYPDDVTSEDMEAVSSAQMESSHSTAAVAALEYLGYRLPTVITVVGIAEDSGAAGKIEVGDQLVSIETPDGTVYPMKSPSAPFRLLRDVPAGTELQVTVERGGRDRTESVITTEDPADPEQGSKLGVVLSFDIDMPVEINFHLENVGGPSAGMMFALGIIDKINGGTLVGDNVIAGTGAMHYDGTVEPIGGIEQKMYGAFRDGAEWFLAPTSNCDEVPGNYPDGLEVRAVGTLSEAVDAVQKIAAGRGDEVPTCESVVG
gnl|extdb|pgaptmp_001581			MNDSRDDRPDEYSDEPERNPLEEILSAFLGPEAAAEAAKQFSLQGFDLSSMADAFGAGSNPVPLGQLQYLFQSTTGPINWRMVEDLAKQHAFQADREPIGFTETRLVNDALQIADLWLSQVTDFTVSDANFETWIKVDWVTQTLPAWKQICEPVAANASRALTEALQSEMGEAEDRGLPPEIQQLAGSLSTALPRMSGLAFGSQIGQALGAMSEHSFGPFDSGLPLARPGTTALVFYNIMEFTEGLTTDPREVLWYIAIRESAHSRLFASVPWLQADLLQAITRYSQEIRIDTEAIAESARSFDFQDPESLNRAMSEGVFSPEPTEAQQRALTRIETLLALIEGWVEVVTAEAGRDYLPDLDKMQELMRRRRVSGSNGEQLLAQLVGLHLRPRQARNAAKLWRLVQDASGPDGRDALWAHPDLIPTAQELADPEIFLATREAANDTTDEFDEALEKLLDGTLGWADGLEPPTDDK
gnl|extdb|pgaptmp_001582			MVERVREGVVLLPWADNLLQVGSSSEHSFVLSGLDEGTTTWLRRLLSRRDIPPLPSLTPDQLRLVGLLRSHGLVETRRSATVRDLHLRVVGLSRTTILFTRLAATSGVSFIDVRDAARVDEEVELLFGPDDLGSLRTEALKAELSSRRVAVGRSSRPHLVVSAERRVVDDQRAAQLLRADLIQLPIVADDRTIQIGPLLVPDLSPCHMCLEFHRRDCLPTWAKARKLLLRTPLSPPELPLAAAAAGLALHLVQSVLTPALRAGAGLPTWALGTAWRLTESGVEETRWDFHPDCGCRAQHMYRL
gnl|extdb|pgaptmp_001583	K03657	uvrD, pcrA; ATP-dependent DNA helicase UvrD/PcrA [EC:5.6.2.4]	MSEMKQPEELLAALDPQQQEVALQVGGPLCVRAGAGTGKTRAITYRIAYGVASGQLSPTSILAVTFTARAAAEMRSRLRDLGVHGVQARTFHSAALRQLSYFWERAFSGSVPQVVGHKAALVASAATRVGVRVDKTSVRDLAAEIEWAKVSMISLEDYPQQAAKLGRAAPADLSMEDFARIYDAYEQAKAERQVIDFEDVLTLTCGLLQERDDIRQKVRGQYRSFVVDEYQDVSTLQQHLLNLWRGPRRDICVVGDVSQTIYSFAGASPRYLIDFPTQFPEAKVVELNRDYRSTPQVVAVANQVMARARGINGLGPERGLEGAVRLVSQREGGPAVSFRSFPTDEDEARGIAQQIQDLSQQGRPLAQIAVLYRTNAQSEALEQALTDLGLGVQVHGGARFFEREEIRRALVLLRQGARMRELVGEGEAEAELSTQVEDILLGAGWTPEPPQREGASRERWENLEALAALARSKPELSLGQFVEDLLERAQAQAAPQVNGVVLSTLHAAKGLEWDVVFLAGVSEGLIPISLAELPETVEEERRLLYVGITRARDLLQVSYARSRSGGRSQKRKVSRFLAPLWPEEAAPARRRAEGDQSAKQKLSQDKERFLAESDPATLALFEELRRWRLGVAGELSRPAFAVMNDVTLRDIATAKPKTLRQLGAVRGIGHNRLADYGAQILQLIRQHGAEQVEDS
gnl|extdb|pgaptmp_001584	K03426	NUDT12_13, nudC; NAD+ diphosphatase [EC:3.6.1.22]	MSLQLMLDRGAIDSMGESRAEFRPADLTPGSWRSLVVTGRGEVAVQGDRLWTTRKLGSAEPYLLGRDPNGTIYLAQLWDGAGLPAGVQFRPLIEMAATLGDNESFLAAQAVALGRWHDTDRYCVRCGHRVQSAEAGWASRCQHCGHVEYPRTDPVVIVRVTDGQDRVLLAHNAAWDRPMLSVPAGYIEAGETPRRAIERELWEEVSVPVQNFSYLGAQPWPGPRSLMLAFHAETIGDEVEPVPDRVEIDYARFFARDEYVAALQTGQILAPRPSSIAAAMLSDWLGAPLPYPQ
gnl|extdb|pgaptmp_001585	K00989	rph; ribonuclease PH [EC:2.7.7.56]	MANEMSKSRADGRAVNQLRPVKITRGWGSAGEGSVLIEFGRTRVLCVASLTEGVPRWRSGSGEGWVTAEYAMLPRATSQRSGRESVRGKVGGRTHEISRLIGRSLRAVVDVAALGENTIILDCDVLQADGGTRTAAITGAWVALQDAINFGREQKLIRERKNHPALKDSISAISVGIIDGVPMLDLPYEEDVRAETDMNIVETGTGGLVEVQGTAEGAPFSRSELTELLDLAHHGNQQLRALQLAALELPLGETLASDWN
gnl|extdb|pgaptmp_001586	K01519	rdgB, ITPA; XTP/dITP diphosphohydrolase [EC:3.6.1.66]	MTTSPRLVVATGNQHKVEEIRSILTPYFSEAILADIAPLGQFTREEPVEDGVTFEANSLIKARAACRLTGLPALADDSGITVDVLGGAPGVFSARWCGHHGDDRANRELLLAQLADVPDQHRGCAFVACVTLVLPDGREFATEGRVKGHLARQSSGSGGFGYDPIFVPDGFSVTTAQLSPAEKNALSHRGQAVRLMVEHLQELLGD
gnl|extdb|pgaptmp_001587			MKLTIIGCSGSMSGPESAASSYLVQAEGPDDAGRTRTWSVVFDLGPGAFGALWKYLNPQDLDAVVFTHGHSDHMGDIISLYVHNRWNPAGQAPICRIYGPDNIAHRMCQLDGWATPEEISEIFDVVTVQPEVPFQVGPMTITPYRARHTVETFGYRVEAPGAKSLAFTGDTDTCESITRMALGVDLLLSEAAFTSADTVRGIHLDGERAGELATEAGVGQLVLTHIQPWTDPSVVLAETERTWAGPLEAARPGQVFEL

<table>
<tr><td>gnl|extdb|pgaptmp_001588</td><td>K01776</td><td>murI; glutamate racemase [EC:5.1.1.3]</td><td>MNNAPIGIFDSGLGGLTVARAVIDNLPDEDIIYLGDTAHTPYGPRPIAEVRELTIAGLDALVARGVKMLVVACNTGTAAALSDAHERYWIRQGIPVVEVISPAAGEAATQTRNGKVGVIGTTATVESGAYLRALQAVPGLEVFQQACPRFVEFVENGVTTGDELLAVAQRYLDPLIDYGVDTVILGCTHYPLLTGAISYVLGPQVNLVASSEATAKTVYSKLMELNLLHDPRPAGQVADYQFLATEDSPRFSALARRFLGPEVQHISTVNTV</td></tr>
<tr><td>gnl|extdb|pgaptmp_001589</td><td>K00763</td><td>pncB, NAPRT1; nicotinate phosphoribosyltransferase [EC:6.3.4.21]</td><td>MPASFSTAMLTDKYELTMVDAALHSGVSNRRSIFELFGRKLPATRRFGVVAGTGRILEALERFEFSEDQIQWLADQGTYSPLLLDFLQDFRFQGDIYGYAEGECYFPGSPLLTVEGTFSECTLLETLVLSVLNHDCAVASAASRMTIAAHGRPCIDMGARRTHERAAVSAARAAVIGGFISTSDLEAGIRYGIPTVGTSAHSFTLLYDREEDAFAAQIAQQGTGTTLLVDTYSISQGVENAVAAARAAGGELGSVRLDSGDLVAHAFKVRGQLDSLGATSTKIVVTSDLDEYSIAALGAAPVDIYGVGTKLVTGSGVPTAALVYKLVARENEAGQMIGVAKRSESKSTVAGRKVAGRRYDEDGYASEEVLVAGADWAQTQEQLRELGMRPLQQPLVLGGEINSDLWSAEAVLAAQELHRRARNELPYAAWRLSEGEAAVPTRYYETGELTK</td></tr>
<tr><td>gnl|extdb|pgaptmp_001590</td><td></td><td></td><td>MTEPQRGGSLDVLEQTQVKPDQGSGNGDRYAHYVRRDRANRSAVTGQAVVALCGKVWVPTRDAKNYPVCPRCKQLRDEMGKQGPSWPFKD</td></tr>
<tr><td>gnl|extdb|pgaptmp_001591</td><td>K02564</td><td>nagB, GNPDA; glucosamine-6-phosphate deaminase [EC:3.5.99.6]</td><td>MQVAVLGSEEEIGAVAAQRVARVLRRKPNAVIGLATGSSPLPLYQELIRLQQAGEISFAEAKAFCLDEYVGLPADHPEGYRNFIEREFTGQVDFAPGTVHAPNGQAADSVAAAKEYDEAIKAAGGVDIQILGIGSDGHIGFNEPGGSLTSRTHLGFLTEQTRVDNSRFFDGDINQVPTACITQGLGTIMDARELIMVVTGAGKADAVRELVEGPVSAHWPATIMQFHNEAIVLLDEAAASKLEGREHLQATWEGYLRTEWADR</td></tr>
<tr><td>gnl|extdb|pgaptmp_001592</td><td>K03530</td><td>hupB; DNA-binding protein HU-beta</td><td>MSMNRTEIVAAIADRAGLTKTQADAALGAFQEVLIDSLSKGEAVKVTGLMSVERVERAARTGRNPRTGEEIQIPAGYGVKLTAGSTLKKAVSNK</td></tr>
<tr><td>gnl|extdb|pgaptmp_001557</td><td>K01255</td><td>CARP, pepA; leucyl aminopeptidase [EC:3.4.11.1]</td><td>MRLTVDDSFDPIPSLSHLPTVGVTAAASGTEPEAHLVQAEAPLPAGAPTWEELAQVGFEGKAGQRVLLPGQPLRVLVGIDEFSSAALRDAGATAASAVPQSTNLTVHLPAAINQAIPSDRAQLVQALAEGVLLARYRYTPLKSADESPAFAHLTLVSDQPEVAEAARRGAVMARTTNLARDLANTPPRHLNADRLGAVAERLGHKFGLTVEVFGPEQLADLGLGGLLGVNAGSVEEPRMIKVSYRAGERQLALVGKGITYDSGGLSLKPSNPMHALMKSDMLGAGAILAAMTALADLEVDTSVTAWLMCTDNMPSGSSTKLGDVLTIRGGTTVEVKNTDAEGRLVLADGLVLAAESSPDAIVDVATLTGAALAALGPRSAALFANDDHLAHLVEAAAASTDETVWRLPLDARYRDQLKSHVADLSNIGGSYGGAILAALFLNEFVGEVPWAHVDIAGPMYSDQDDLWRVTGSTGFSARLLVELAQTFTTH</td></tr>
<tr><td>gnl|extdb|pgaptmp_000706</td><td>K03664</td><td>smpB; SsrA-binding protein</td><td>MPKDWKKPKPSESERRKAESDAKKVIARNRRARHDYEIEQTWEAGLSLMGTEVKALRMGRASLVDGWVEVKDGEAWLYGVNIPMYSQGSWTNHTPTRKRKLLLHKTEIQRMEHKVAAKGYTLVPLELYFLGGRAKVEVALGRGKKEWDKRQALREAQDKREAQRAMSRYVRRGNA</td></tr>
<tr><td>gnl|extdb|pgaptmp_000705</td><td></td><td></td><td>MRTVLRARLSRFLTPVIVAGLVGVGGLLATVPASGDERDDAVADQQEAEQKIDELQGEIEGLDADLAKLFTELEQVTGQLTAAQDDLAAAEEALSAAQLEYEAVTEQLAAAQATVERLAAELDSSAAEEEQLNAAVGTMARDLYRGHQSSPLSLVMSAEGTGDIAQRAASAVTMSRAQTRALEQVRSSMAVVRNQAEKQEATTKRVEELQAEAIQKQQEAEAAQAEVATRVENLATAQSDLKAKQQQWDARKAEAKKQLEQWEKSRQDAADRVAKIDEENRKKALEFAEAPGPSSGNSMFGFPLPAGSAVVTSTYGWRIHPIFGTSKLHDGVDFGAACGSPQYAIRAGVVVASYFDSGGGNLVTINHGMIDGSSWVSEHLHLQSSNVSVGQQVNRGDVIGWTGSTGNSTGCHLHLTLYRDGSTVDPLDYIG</td></tr>
<tr><td>gnl|extdb|pgaptmp_000704</td><td>K09811</td><td>ftsX; cell division transport system permease protein</td><td>MRLRFILSETGKGLARNKAMAVAVIIVTFVSLLFVGVASLAQLQVGKMRSQWYDKIEVSVYMCANKDQSPTCDLQQASDEQIEAVRQRLASPELAPYISKVYEETPEQAYEAFQEQYANNPMAQWTTPDMLGFSFRVKLVDPEQYQIIQEEFSGTPGVSEVRDQRDLIEPLFKVVENAKLISLGLAGVMVVAAILLITTTIQLSAMSREEETQIMRLVGASNLFIQAPFMIEGAIAALVGAGLAVGSLFAAVRLLIENWLAPSFPWTNFITTTDMWLITPILVGAALLLALIASAISLGKYTKV</td></tr>
<tr><td>gnl|extdb|pgaptmp_000703</td><td>K09812</td><td>ftsE; cell division transport system ATP-binding protein</td><td>MIRFENVTKVYAKGARPALDHVSLEIVREEFVFLVGKSGSGKSTFLQLVMREQRANSGHVWVLGQDVGQLSNWRVPKLRRQIGTVFQDFRLLQSKTVYENVALAMQVIGKPKHAIQSAVPDVLELVGLQGKERRLPSELSGGEEQRVAIARAMVNRPQLLLADEPTGNLDPETSLGIMRLLDRINRTGTTVVMATHDAKVVDQLRKRVIELEAGQVIRDQRGVYGGAR</td></tr>
</table>

gnl|extdb|pgaptm<br>p_000702	K02836	prfB; peptide chain<br>release factor 2	MSIEFAEEIRHLQSVMDNIVAVSRPERMRAEIQRLSDEAVAPNLWDDPATAQQVTSALSHLQSELERLEKTQARIDDLEAMVSMADESTDTDEAAELLAEA<br>GSDLAKLKDDLAQLEIRTLLDGEYDERFAVVTIRSGAGGVDAADFAEMLLRMYLRWAERHGYPTKMLNTSYAEEAGIKSATFEVDVPYAYGTLSVEAGTHRL<br>VRISPFDNQGRRQTGFAAVEVIPLIETTDHIEIPESELKIDVFRSSGPGGQSVNTTDSAVRMTHIPTGIVVSMQDEKSQIQNRAAALRVLQSRLLVLRHEQEKA<br>KQDELKGDVKASWGDQMRSYVLHPYQMVKDLRTEQESGNTSAVFDGEIDDFINAGIRWRKSSAQED
gnl|extdb|pgaptm<br>p_000700	K03657	uvrD, pcrA; ATP-<br>dependent DNA helicase<br>UvrD/PcrA [EC:5.6.2.4]	MSRNDELSQLVAQLGVHQPTPEQAEVITYPLRLDREGESVATPLLVVAGAGSGKTETMSLRAVYLAATQGIPDDAILGLTFTRKAAAELAARLSDRLAQLRLE<br>AGSDLGALEFESSPTATTYDSFALDVVREFGPQLGIPTDFSHLGAAASWQLMYSIIEEWPTRISEGRELSTITTAALELRDAIANQAMTLAEARRALERIDQRFN<br>AREHDEEAPKFTQPLAAGRDLNRERLALLPVIEAFEERKREERRLDFSDQVLLATRIVEHLPTARAELRDRHQVVFLDEFQDTSVAQLRFLSALFADHPVTAVG<br>DPNQAIYGWRGASAASLNDFHRYFTQNRRAPRGTLSLSTAWRNSTLILDVANRVAQDLARPPAWLESATMREELVFSPTLQPRPEAPTGEVTLRYRQTETE<br>SIQDVVEFLRDTYRQAEKNGLPRPTAAVLCRNRRLMAPVLEACRAAEIPAETGGDDGLLLHPAVLDVRAALEICHDLGRSSQIMRLLTNLDLGSKDLWALSRL<br>AKQLARRAKPKNSEQPDRREVPLLIDAVDYLSGPQAQRLELSEAEQISAIGRERIVRLGRQLRAMRRAADWPIVDQVENARRILQLDEQAFALGDLAGLTEV<br>LDAFTRAAQDYVDSAVQPTMGGFLSWLSAANQFERGLPLPTVQPNPEAVQVLTIHGAKGLEWDTVAIVGMQSGHFPGGQPGKLVDQPDGSVKATPPP<br>MPPTVSGWWQNLGELPYPARRDHEHLPNPDAWAGDEAFTKVFTEFKTCLGEYQLAEDRRLAYVALTRAKTRMFLTGAWYHGAKTRRYPSLFYQEAEEVP<br>GVDSRRDDPPTAVQVQEFMQVETEAVFPIQPGPLRKRVSLSAAKVEEQLALLSGGSGWRPADLLASLPDQELATSMQVLLREHEDRQALRRLSRSERARRE<br>VKLAAAGDRAFSATELARLDLVEDPWVELRRPLPARPMPAAAIGTAFHQWVESVLRRASARASEEEPGQDLLPLGGADQDLGADAVLDPEQRQVLGELQS<br>QFTGLEWLGRLRVEGVEVPFDLDLGGLLVRGRVDAVFTDPDDSSSWLVDWKTGRIPDLDRVEAIDRAEIIRYLVQLEVYTAAWQRRHGPEHPVQAALVFVR
gnl|extdb|pgaptm<br>p_000699			MTEPLELLRTGESFLVQGGGPGTGKTQFLHRAIDWIMTEDRDGWDWAPFLVLTPDRRRAAQFEREVPSELLGAITAPGGHRVVRGTNSYAYLVLSEWALERE<br>EPLPRPKLTGGAEEDLWFARWLGEHGADWPDLLPADALNSEAVRMELRNLVARLGESGLDGPALADLAREAGVPAWQLAAAAYSDYAGVEGCAFDQRT<br>PRIDSARIQLVAASLLEHWEERAPQEGVTACPPVPRWVLVDDLQDCTPATAKLLTAMQGAGAQIVATCSPATATGSFRGARYDLGPRLARERGWPVLTLTR<br>QWRLNPDLDQVVSAIDRWTAPRVQPEPLAVSGGAAQAVILTGPGRRGSWLAQQIRRHHYLDGIGWDRQAVLVRQAGDIDQLQRQLLRAGVRLAPGQR<br>ALQFSKIPVTAMLLQLLLPTEDPAQLAQELLFSALVQVDRLELYQLLRQDQRGDQSLADTLLSWLQEPDLVPAGAGLPRELRDRLERASAVWLAREEAAADG<br>AQGGLWRLWEAAGVAQQWQQEALRDGVEAERSDSRLDAVTGLFRRADLWEQQRAALGQGTQSSARVFAEELWAQTVSADSIATGGLREPGVEVLTVA<br>QAAGREWDVVYVVGLQEGAWPPRPRLSGLFHLARLQQLLAELPVSATAGQAQEFLGRLDPDLLRLQLDQRQYRLERRKDEARLLASACSRARDRLYLVAVE<br>SQEDAVSSFLNYLAKQGAVPDFRTEDGQVIPTEVPPPFDLDALVGRLRFATVDPQLSSADQTQAAALLAVLAEAGIDGADPRTWVGAGGISAAGEPIVDGKL<br>GLSPSQVETGENCLLRWFMQSAYGEDRAGIFTPTPLGAADKGNLIHAVAEAYPHGSRAELQAEFARQWDALELDENKWWVQRTREQMVNWVDVLASY<br>VAGVPGTVEVERQVRTELEHATVYGRVDRLEFLDDGAVRIVDIKTMKTAPSAQAVESNWQLVSYQLALREELAIAEAGLLTVAAPAKSGLLRAQLPLTDEEA
gnl|extdb|pgaptm<br>p_000698			MKIEIRMRHGGQLDFETAATAEQLRELLKQEDEILDLTDSKGNRALIPIHAISFIVIPHEREMRVGFARA
gnl|extdb|pgaptm<br>p_000697			MNATDNTAPLVPPVPSSAPLDEGTKNLVGLLAFSALAAMTRLAKDGDQAPSTEFHVEHARMAAQAFREFQHLERFAAENGFDLIEQASQFAGLFDEIDAR<br>TRPSNWWERSVKTYVTLGIFSDALSDINERHQLFVEEEWDGDFGQGLWVREHLAPLTEKDPQLAARLSLWARRVAGEAFGLLRATLFTYPELAVDPDTVDA<br>VVAATIRRHQERLEAVGLQP
gnl|extdb|pgaptm<br>p_000696			MTEELKTDQPSTPEARDFASFGIRSEVVEALAEQGITHPFPIQALTLPVALTRHDIIGQAKTGTGKTLGFGLPLLQHVHGPDDPEYAALSHPGLPQALVVLPTR<br>ELCKQVGEDLRAAAKHLSVRIVDVYGGVAFEPQIEALTRGADIIVGTPGRLIDLSRRKILNLSQVHSIVLDEADEMLDLGFLPDVERLLSSIHHDHHAMLFSAT<br>MPGPVIALARRFMTQPTHIRAQDPDDQGATVATVDQFIYRCHAMNKVEVIARILQARGRDRTIIFTRTKRSAASLTEELTERGFAVAALHGDLGQGARERAL<br>RAFRNHKVDVLVATDVAARGIDVDDVSHVINYQCPEDEKTYLHRIGRTGRAGASGNAITFVDWDDVPRWGLINKTLDLGLPEPVETYHTSPHLYEDLDIPTD<br>VTDRLPRSQRKLEGLQAERLEDIEGKGGRGRPAKSGRGSSNGRGRGRDQGSSRSAAPEAKSTRSRQRRRVRKSDSD
gnl|extdb|pgaptm<br>p_001310	K05595	marC; multiple antibiotic<br>resistance protein	MEISSFVNVTLLATTFTTLFVIMDPPGTIPVFLALTSRYSTRQKQRAAVLATATSFGVIVLFAVLGRYILQFLQISMESLQLSGGVLLFIVAIQLLMSNEYEGHGEP<br>ADAGADGGSHRVNAALVPLGTPLLAGPGAIVAVMISVEQGSNTIPGWTAVLAAVILMHLVIWLTMRFSLTLSRLLGEGGIMILTKVAGLLLAAIATQMMM<br>NGLFQFIAHSKEMGVI

gnl|extdb|pgaptmp_000695	K07053	E3.1.3.97; 3',5'-nucleoside bisphosphate phosphatase [EC:3.1.3.97]	MIRFDGHVHSDISDGTDSPARVAELAERAGLSGFALTDHDTTVGPAVQANLGQTGLLVVPGVEISTSYRGNSAHLLAYLPDPSHPQLQETLTRIRSARENRLRQIVENISADYPQITWERLVASQPLPAPTGEATPWGRPHVADLLVATGVVPNRTAAFETILSHRGPYFVKQWAPDPRDMVELVRAAGGVPVLAHPRTRGRQLPLPEEVLEGMVDAGLFGLERDHREHDEADRLALDQLAHRWRIALTGGSDFHGTGKPNRLGENLTSQAVLEQILAEGTPLSQLGFSS
gnl|extdb|pgaptmp_000694	K01262	pepP; Xaa-Pro aminopeptidase [EC:3.4.11.9]	MTNNTDETTKDLAERGSNRSHRPNSAAFKEFMGSNWGERPPLLTETAPVAQYLASRHHEAGAPFAGERLVIPAGSLKTRSNDTDYRFRAHSAFAHLTGLGGEMEPDSVLVLEPVEEDGQVTHQGVLYFQPRASRSSEEFYADSRYGEFWVGARPSLDEMELFTGLKCAPIDTLKDALAKDAGLVQLRVLPQVDATVEALVNEVRVQAGLPAGADAVAQDAALVEHLSELRLQKDQFEVAELQRAVDATALGFERIIRELPRAVGHRRGERVIEGAFATAAREEGNGLGYETIAAAGNHANTLHWMDNSGQVPADALVLVDAGVEIDSLYTADITRTLPVSGKFGPTQAKVYQAVLDAAEAALVRAGEPGCRFRDVHQAAMEVIAQRLEEWGILPVTAAESLAPEGQQHRRWMPHGTSHHLGLDVHDCAQARQEMYQDAVLQPGMAFTIEPGLYFRADDLAVPAEFRGIGVRIEDDVVVEADGSVRRLSEDIPRTIADVED
gnl|extdb|pgaptmp_000693			MSNNRAVSREMPTLPTGTEVASYSQYDQAVAAVAALAESQFPLQSVSIVGTDLHMVERVVGKVTPARVALVGAGQGLSWGLLLALMVMLFADKYSVIIPLLAIGAGIGVGMLTAVLSWSGQRGRRSFAAQSQLVATRYALLVSEQTDRAYQLLQGSQGNLNRVQRRRVRRPEASTGPTEYGSRPDEKPRFGVRLSEVSGESAPKPAEDSAPAGSPAPEAEHSEAETRPPTDD
gnl|extdb|pgaptmp_000692	K03593	mrp, NUBPL; ATP-binding protein involved in chromosome partitioning	MALTEDTILEVLAKVQDPEIHRPITDLKMVNSVAIDGGQVTVEILLTTPGCPLKSTITRDVEQAVQALPEVESVEVKMGVMDDDQKRELREYLTGHKERDIPFSQPGSLTRVIAVTSGKGGVGKSSVTANLAVGLAAQGLKVGVLDADIYGFSLPRMLGITNQPQSIDGMIIPPIGAGGVKVISIGMFVPEGQPVIWRGPMLHRALQQFLADVFWGDLDVLLLDMPPGTGDVAISIAQLLPGSEILIVTTPQVAAAEVAERSGSIASQTNQRVIGVVENMSYLTQPDGSRLDLFGSGGADMVSARLGKLLGYPVPVLGQVPLDIALREGGDAGQPVALSPESTPASEAFDELSGKLAQQARGLAGRPLGVSPL
gnl|extdb|pgaptmp_000691			MARDIAQVWDFCENYVSEPEPMVRAREVALELGADPVSPGTGALLRTLAATRDARAVIEIGTGAGVGSLWLLAGMNPDGVLTTIDPEGEFQRAARANFRQAGIASHRSRFINDRALDVLPRMAEDAYDLVVIDGPAEETPQYLDHAARMLRRQGILVVVNALWNGNVANPAKRDMNTVIMREMTRALLESEQFVCSLLPI
gnl|extdb|pgaptmp_000690			MAAMKPRTGDGPLEATKEGRGIVMRIPSEGGGRLVIELTPDEAEALAAALSAAI
gnl|extdb|pgaptmp_000689			MSKARRSYRSGSAIMRGEQIPTTTADTELLQSRRQDSWRVLRIQAEFVEGFGALMELGNAVSVFGSARSREGSPEYQLGEELGRLIAERGFAVITGGGPGVMEAANKGAHEAGGISVGLGIELPFEQGINKWVDLGVNFRYFFARKVMFVRYSRAFVALPGGFGTLDELFEALTLVQTGKIGSFPIVLVGRDFWGPLADWIRDRLREGGLISPGDEHLFTIVDTAEEAVATIFEFMEKNGQS
gnl|extdb|pgaptmp_000688	K03783	punA, PNP; purine-nucleoside phosphorylase [EC:2.4.2.1]	MENSPGIDNPFALAQQAADALRERSGVEKYDIALVLGSGWGESADLIGTTLSEVPAAEIPGFHAPAVAGHNASLRTIRVEETGKIALVLGSRTHYYEGRGTRAVAHGMRTAAAAGATQVVLTNGCGGLNPSWAPGQVVLIRDHINLTGATPLEGATFVDLTDLYSTRLRTLAHTVDQTLPEGVYVQFPGPQYETPAEVKMAGVIGGDLVGMSTTLEAIAAREAGMEILGLSLMTNLAAGIGTEPLSHKEVIEIGQAAAPRIAKLLAQIVNRM
gnl|extdb|pgaptmp_000687	K01840	manB; phosphomannomutase [EC:5.4.2.8]	MTNYNREEIERWITDDPDETTAAEIQALLDQADAGSEAAAEDLADRFAGSLTFGTAGLRGHLGGGPNRMNRAVVLRAAAGLMKFLQQKLPDGYTVVVGYDARYGSRQFALDTAAVAEANGGHALIFDSALPTPLTAFALRHLDADAAVMVTASHNPPEDNGYKVYLGGRVVQDSGQGAQIVPPYDQEIFTAIQSVPSVASIARAESGWEWLGSELVDRYLARIQELVQPGPRDLRIVVTSMHGVGGETMLAALDRLGFTDVTAVPEQQQPDPDFPTVAFPNPEEPGALDLAMALAAKLDADIILANDPDADRSSAAIPDPSAPGGWRQLSGNEVGGLLGEQTAAELAGQSEAVLANSLVSSRLLSRIAARYGLQHRRTLTGFKWISRVPGLVYGFEEALGYCCDPNYVRDKDGISASSRLAALAAQLKADGSSIQEMLDRQAREYGLHATAPLTIRVDDLSLIAQGMANLRAQGIQTIAGSPVVETVDLATGSDELPPTDGLLYLTEAGDQVVVRPSGTEPKLKCYIESIVPVEGDDVASARAVATERLEAIKVDMRAAMGI

<table>
<tr><td>gnl|extdb|pgaptmp_000684</td><td>K01619</td><td>deoC, DERA; deoxyribose-phosphate aldolase [EC:4.1.2.4]</td><td>MSERLTRDQVAHMIDHTLLKPEATVSDVEALVEEAARLGTFSVCVSPSLLPVEAPAGLAVACVAGFPSGAHDSSVKAAEAAAAAANGVDEVDMVINLGRAKMGDWQAVEDDIRAVREAAPEVLLKVIIESAVLTDDEIVAACRAAEAAGADYVKTSTGFHPAGGASAHAVRLMRQTVGDRLGVKASGGIRTAEAAAEMIEAGASRLGLSASAAILADWK</td></tr>
<tr><td>gnl|extdb|pgaptmp_000683</td><td>K00758</td><td>deoA, TYMP; thymidine phosphorylase [EC:2.4.2.4]</td><td>MMASELFDAVDVIRTKRDGWVLSDAQINWVIDAYTRGVVADEQMSALAMAIFLRGMNRQEISTWANAMINSGERMDFSALPRTTADKHSTGGVGDKITLPLAPLVAAFDVAVPQLSGRGLGHTGGTLDKMEAIPGWRADLSNEEIMEILAGPGAVICAAGKGLAPADKKLYALRDVTATVDTIPLIATSIMSKKIAEGTKALVLDVKVGSGAFMKTRAEAAELARTMVDLGVDAGVNISALLTNMDTPLGLTAGNGLEVEESVEVLAGGGPQDVVDLTVALAEEMLRLAGKPDQDVRAALRDGRAMDRWRQMVSAQGGDPDAPLPVAKHSEVITAPESGYLTRLDAWGVGVCAWRLGAGRAAQGEKVQAAAGVRMHAKPGDKVVAGQPLLTLYTDTPERFERSIEALDGSFEIGPSQPESVPVVLDKISN</td></tr>
<tr><td>gnl|extdb|pgaptmp_000682</td><td></td><td></td><td>MTVTEDQWAELHRLAVSAMERAYCPYSKFPVGAAALVDDGRMITGCNVENAAYGVALCAECGLISELVNTGGGKLVAFACVNSQSEPTAPCGRCRQLLFEHAAPELVLAMPAGRMTIDEVLPGGFGPADLDAKQEGK</td></tr>
<tr><td>gnl|extdb|pgaptmp_000681</td><td>K23536</td><td>nupC; general nucleoside transport system permease protein</td><td>MNQQQVTLPIQWRDPVVSAIMTVLTLILTLFSKGYSNISFNKATTWFEGGVWTIPAATTGWLMVAIAAVVTVLIWVRAIGRKKVSAWLLGLVGVAFVITFLVWMVADRPTANLPLVSLLSGGLMFATPLVFGALAGVVNERSGVINIAIEGQLLLGAFAGVLVGSMFGTSWVGLIGAPLAGMGLAVLLAWFTITFRTENIVVGVVLNMLALGLTSFLFSTLLKNSPHLNQPMRLPIIRIPVLADIPVIGPVLFNQTILVYLMYAAVIIMQFMLFNSRWGLRVRASGEKPKAADTVGIKVNSIRWHSVLLGGALAGLGGAVFTIGQDLAFSKDMAAGNGFIALAAMILGRWNPTGALAASLLFGFATSLGIVMQSVGAPIPAEYVLMTPYIVTILAVAGFVGAVRPPAEE</td></tr>
<tr><td>gnl|extdb|pgaptmp_000680</td><td>K23535</td><td>nupB; general nucleoside transport system permease protein</td><td>MSNAATLAPVDPPAPGQGHADETTNWVQKIIRTFFSPSAGTVALAILGALVIGALIVVFFDPQVQATMGYFFQRPGDFFSEAGRAFSSFFSALVRGAIFDWTQPTFAKAIRPATESIVRAIPLIIAGLAIALSFTAGLFNIGVQGQIILGAIFGGYVGLALNLPPVVHLLVAILAAVIGGALWGFIPGILKAQLGANEVIVTIMLNSIALLLLQALLNTEAFHGTGYAGKSMPVGPNAVYPLLMGSGFRLHFGFLVAILAAVFLWWLLDRSTFGFELRAAGANPEAANTAGINVKRTMMLTLVLSGALAGLAATAPVLGTEKGLSVGLAGTIGFDAITVALLGKSRPMGVFFAGLLFGALNAGGALMQSSAGIPVDIVQITQAIIVLMIAGSEAVRWMRQRRKQDQQTVEKV</td></tr>
<tr><td>gnl|extdb|pgaptmp_000679</td><td>K23537</td><td>nupA; general nucleoside transport system ATP-binding protein</td><td>MKLELEGITKVFGPLVANDQISLQVEPGEIHALLGENGAGKSTLMNVLYGLYQPDEGQILIDGKPVRFQGPGDAVAVGIGMVHQHFMLVPVFTVAESVALGYEPVGPLGVINHQKARQKVKELSDRFGFDIDPDAYIEDLPVGVQQRVEIIKALSRDAEVLILDEPTAVLTPQETDELIEIMRQLKASGTSIVFITHKLREVRAVADKITVIRRGKVVGEAKPTDSEQDLASLMVGRPVDLGVDKEPAQPAEAQLTLDNVTVIEDTGHTSLDGVSFEVRRGEILCVAGVQGNGQTELCEVILGSTAPAGGRILFGDKDVASTGIKQRLRSGLGYVPEDRSKDGMIATFSIAENMILDVYDQKPFATGMQMHPQVVGENAEKLREEFDVRVTSVHDPISTLSGGNQQKAILARELSRPIDILVAAQPTRGLDVGSIEFVHQRIVQARDNGTAVLVVSTELDEVTSLADRIAVMYRGRVVGIVPADTDRNVLGLMMAGVPYEEANK</td></tr>
<tr><td>gnl|extdb|pgaptmp_000678</td><td>K07335</td><td>bmpA, bmpB, tmpC; basic membrane protein A and related proteins</td><td>MKNTLKFGAIAAVAALSLAACGQAPAEQSSPSGDTTQSDAGAAPSSDVKACMVSDSGGFDDKSFNQSGHEGLMRAKDELGVAVDQAESQSDQDFVPNINNMVQGGCDIIIGVGFLMADAMDQAAAEHPDVKFALVDSTFASQAEGVDGLPNARALVFNTAEASYLAGYAAAAMTTDKVGTYLGANLPTTAIFADGFADGIAKYNEVHGKDVELVGWDKAKQEGMAVGNFEDVPKGKQFTEQLIEQGARVIMPVAGPVGAGTLAAAKDSADTWVVWVDADGVETQPEYKDIILTSVMKDIGNAVFDTIKAVQDGSFDANPYIGTLENGGVGIAPWHEFEDVVPQEIKDEIDQLKQDIIDGKIVVETVNAPK</td></tr>
<tr><td>gnl|extdb|pgaptmp_000677</td><td>K16011</td><td>algA, xanB, rfbA, wbpW, pslB; mannose-1-phosphate guanylyltransferase / mannose-6-phosphate isomerase [EC:2.7.7.13 5.3.1.8]</td><td>MSRQSQPKFLLDPLGRGQTLLQETFDRLREMALTITVVTGPQHYEEVRRQLPDLTEHGDNPFPGEILIEPSPRDSMAAIGLATYIIRERYGDGALVGSFAADHVIEDRAAFEAALSLAEQGARSGLLTTLGIVPTEPSTAFGYIEPDGEEELSAVRRFVEKPDEERAAEYVAAGYLWNAGIFVAEAGTIAAGLAAELPAMDTQLRAIARRHLKTLVETGENGQIRAEHWDRLTRIAIDYALAEPMAARGQVAVVRTSPNLGWSDIGDFAAVARLRGNDPLILSERHVEVLGLTGIGVVDTPDALLVLDLEQAQEVKEIVDRLRETGTNSLI</td></tr>
<tr><td>gnl|extdb|pgaptmp_000675</td><td>K02897</td><td>RP-L25, rplY; large subunit ribosomal protein L25</td><td>MAEQTNVLLAQLRTETGKGAARRSRRAGLVPAVMYGHGIDPVHLDLPGHEAFLIVKDNANAVVTVKYDGKQQLVLVKNVQVHPVRRNILHIDLLAVRADEKVQVEVPLVLTGESAPGTQHQQEEFFLLISAPATDIPEHIEVSLDGLEEGAIVRVEDLKLPADVIAETPADRDVVSILALAAEAEEEAATEEAAE</td></tr>
</table>

gnl|extdb|pgaptmp_000674	K00948	PRPS, prsA; ribose-phosphate pyrophosphokinase [EC:2.7.6.1]	MSGIVSSGEKQLILISGRAHPQLAAAVAEELGTEVSSTTAYDFANGEIYVRFNESVRGADVFVLQSHAAPINKWLMEQLIMIDAAKRASAKRITAVSPFYPYSRQDKKHQGREPISARLIADLYKTAGADRVMSVDLHAAQAQGFFDGPVDHLWAMPVLVDYVKERIDPEHVAVVSPDAGRIRVAEKWAAKLGNCPLAFVHKTRDTSRPNVAVANRVVGDVEGRQCVLVDDMIDTAGTIAEAVKVLYQAGAKDVIVAATHGVLSDPAPQRLEGCGASEVVVTDTLPISEAKRFPQLTVLSIAPLLARAIHEVFEDGSVTSMFDGVA
gnl|extdb|pgaptmp_000667	K15738	uup; ABC transport system ATP-binding/permease protein	MAHLLGAEKLHLEYPTTTIFTDVTLGISEGDRIGIVGRNGDGKTTLLRLLAGDLEPDRGQVIPRGDLRVGYLGQADQFPPEVTVREAVVGDLAEHEWAGESRIREIIAGLVADLPWESRVGELSGGQVRRVALASLLVQEWDLLILDEPTNHLDLQAIAWLARHLQQRWAPGKGGLLVVTHDRWFLDEVSNATWEVHDGIVEPFEGGYAAYVLQRVERDRQAAVKEAKRQNLLRKELAWLRRGAPARTSKPKFRLDAASALISDVPPLRDSVQLSQLATARLGKRVVVLLDVSFSYGDNPILSDITWRIAPGERSAILGANGAGKSTLLRLIEGDLSPTSGLVRHGKTVKLAVLDQRFRALEDIAEDRVREVLARTKTSFEVDGKELTPAQLLERLGFSTAHLSAYVGELSGGQKRRLQLLLILLSQPNVLILDEPTNDVDHEMLTAMEDLLDSWPGTLIVVSHDRYLVERVTDQQYAILDGTLRHLPGGVDEYLALSAAGGGSGLPESSVGSGPAVSGLAESGLAMSGLSGAERRALEKELAAIERKLARISGEIAKVHEQMAAHDQSDYSGLSDLTAKLRAKEDEVDDLESRWLELSNSLE
gnl|extdb|pgaptmp_001593	K00919	ispE; 4-diphosphocytidyl-2-C-methyl-D-erythritol kinase [EC:2.7.1.148]	MREAIGQAPAKVNLVLKVARPGPDHYHPLLTVFEAVNLWEYVQARTQRAPGVSVRTLAYRPSVDGVGAPALDPVATGALAQLPPEQHLAVRAAKVLQPLVAARWGATAAGLSLTVHKTIPAAGGMAGGSADAAATLVAVNDLWDLGLGLEQLEALGRRLGADVPACLRGNWSVGDDRGDRLLTVAPAPAQPIHWWALAFFRVGLSTPAVFRRFDELGLGAEELPSASDYLDRARALTGPAGELGPLLDNELQATALSLRPELAQAGQQALSLGATAWVVSGSGPTVAALCASHEEAVRVAEAWQAEAQWATSDLLGATVVGGAVGAAQTAAELPVWTETARSGSL
gnl|extdb|pgaptmp_001594	K02528	ksgA; 16S rRNA (adenine1518-N6/adenine1519-N6)-dimethyltransferase [EC:2.1.1.182]	METEGNLPPKYGPLLGPVAIREISAALGVSPTKKLGQNFLHDGGTVRRIVASAQIDPGDIVLEVGPGLGSLTLGLLGAQARVCAVEIDPTLAEQLPKTVAELQPDRADDLVVYQADALTVQSWAECAPGWAPPRRLVANLPYNVAVPILLHFLALLPTLESSLVMVQAEVADRLVAGPGSKTYGVPSVKVAWYGQAKRAGSVGRTVFWPAPNVDSALVDVQLFAEPRGDESLRQATFALVDAAFAQRRKMLRAALRSWVDSAEEVNALLERAQIDPTRRAETLSIDDFVQLGRASLTMSVSEAPLLAAARERGRQDA
gnl|extdb|pgaptmp_000136	K02025	ABC.MS.P; multiple sugar transport system permease protein	MAKKRRQRQPQAANASSKTRRLGLALAAPALIYIAIFLAFPLLYNLYLSVADASGANLVSGNLHLNGVENYRVILQDPAFWHSALLSLIFTVSCLVFQYIIGFALALFFRRPFMGNGPIRALLLVGWILPPVVTATAFRWMFDADYGVLNYLMQSMGLIDEPIRWLSQGATAMIAVIVANLWVGIPFNMLLLLSGLHLIDDTLYEAASVDGASPWRQFWSITFPLMRPVTVSVVLLGVINTYKVFDLIYVMTKGGPVDATTTLPIYTYLRTFSFFEFGQGSAASVLTLILPITLSYFYVKSLNEEER
gnl|extdb|pgaptmp_000137	K02026	ABC.MS.P1; multiple sugar transport system permease protein	MATALETPAELSPAANANRHGWQRWGKPAIAWLIAAIYLFPVYWMVNTSLKSSRDMFAAPPQLYPHDPQVKAYQTVFSPTYGIPQAFLNSLIIATSVLILTLLIAIPASYAVARLRGRVTTSMMIMLTVVQLLPAITISVPLFVMFRHLGLINSYVSIILADISATLPFAVILLRPYFKLFPYEVEEAARLDGLGTIGTITRIVLPTIRPGIVMIGSFAFLMAWGEFTFALTLTTEQTIQPLTVALNRIMGQYGTAWNDLMATAVIIAAPVLLLFIFMQRHIVAGLAGGATKG
gnl|extdb|pgaptmp_001615	K01951	guaA, GMPS; GMP synthase (glutamine-hydrolysing) [EC:6.3.5.2]	METSPQHPVLVIDFGAQTAQLIARRVREAHVYSEIVPHTWTAGQIAAKNPAAIILSGGPSSVWEKGSPNVNPDIFTLDVPILGICYGFQTMAQALGGAVENSGQREYGRTELSFTGEPSVLLEGTPNRQVVWMSHGDAVSQVPDGFVVTASTDQTPIAAFEDPARRYYGVQWHPEVLHSEYGQQLIENFLIRGAGLTPDWVPSSIVDRLVDQIRDQVGSAQVICGLSGGVDSSVAAALVHRAVGDQLTCIFVDHGLLRSGERQQVEQDYAANMGIKVVSVDESERFLTALAGVKDPEDKRKIIGREFIRSFEDAARKLTEEAAASGQKIEFLTQGTLYPDVVESGGGEGTANIKSHHNVGGLPDDLQFELVEPLRELFKDEVRAIGRELGLADKIVNRQPFPGPGLGIRVVGELTRERLDILRAADLIVREELTEAGLDQEIWQCPVVLLADVRSVGVQGDGRTYGHPIVLRPVVSEDAMTADWARIPTDLLAKISTRITNSVPEVNRVVLD
gnl|extdb|pgaptmp_001616			MKLPVATNRELWRGVAGLMGHHRWQLTLVVILQVAAASTTAALPWLTGDIIDRIQSGTQMRTLLWLIGVALVIVAFGAVLTFNAERRARVLGETVFAQLREELVETITHLPLSVVEEAGTGDLLGRSTHDIERIQFMVRQGISAIMAVVTTIVVTVVASVLTSPLLSLTLIIPIPLIYLTMRWYLPRTVPAYRASATAWANMSGVITESLDQAEIVDAARLRPLRDRRLDEAIRAVWRLERYTAWQRLVMWTGLVLAVFLPVGATIILGAYLYPVGLVTAGQITTVALYCYQVRGPVWEMTFWVDELQSAQAALGRIFGVQLVEPDRHPSGEVPETDKLDVDEVHYSYRSGSPVLHGVSLDLAPGEVLALVGPSGAGKSTLGRLVAGIHPPEAGSVRLGGVELVDLTEERLQREVAFVSQEHHVFGGTVADNLRLAKADASDEELKAALDTVGASPWVEQLPDGLETKVGTGGKELSPGQSQQLALARIVLQDPTVLVLDEATSLMDPGAARTLERGLGQVMSGRTVIAVAHRLHTAHAADRVAVMVDGRLAELGTHDELVRLGGEYAKLWSSWQNE

gnl|extdb|pgaptmp_001617			MEAYDNTRSYTGFLRDRAEQMNVTLPPLGKTPRRWSKSLRTPQGTPPELSAKPFTFIRRLLRLAWRPIALQTLLMTVSSVGGALVPYLMGTMINGLIEGGFD<br>AYTRGQALWFVLLIVLISAAEGIGQLSGIATWMGGSMIATWAVGRRVSRAGRAAKSDTPAGEVVTAMVNDSDHLGGAFVWLPELISNLAAAIVVVVIMFR<br>VSVPLGWVVALGVPAAIALVTLLARPLQDKLAIQREEQGKLTGISTDAVAGLRVLRGIGGEAVYNERYRAQSQKVRAAGVAAASNQAALVVLRNSTPQLLIA<br>AVIGYGAYLVSHGQLNAGNLVAFYGYALYMRMPIGTASAIVQHWTRGWVGAKKLAAAYATEPEVNDEQVDPTLPEPQWLEADLRDRSSGVSVPPGQLTA<br>LVSSSPERAAEVARRLARISDEDESAVSLDGVDLRAYPLETVRQGIYLSESSPQLFRESLQSAILGADAPEFPLRGVTELVYREHVENFAREEDTLFQPEPVDRTP<br>RLDQAIWAAAATDVEASMDWGLAGELTEKGRNISGGQRQRVALARALYAKTPILVLVEPTSAVDAHTESQIAARLRQVRSGRTTVVVTTSPLLLAASDRVV<br>VLDQDGKELGEGTHEQLWEGSGQAADQYRQIISREVSGA
gnl|extdb|pgaptmp_001619	K13788	pta; phosphate acetyltransferase [EC:2.3.1.8]	MSSRILVMGTGSSPLNTQLVHEISQLLAAHSQSTGGQTFSELDAFAGLKFTRVIADPNQALAEVAVSIRSNSSDTVLIKGVDGVPAPSFDVTGWNLDVAANT<br>GAQVLALIDGTGMNAELIEAEAAEFGRRAARHHATVGGLVVSGARGLDFQLGGIPAIEPPLSSEKLAEVTGADPSVVTPLMFQGDLLARASANRKTIVLPEPE<br>DDRVLRATAELLAAQVANIVLVGEAGAVQARADQLGLDISGARIVSPHQPDLVEKYAAEFARLRAKKGVTLEQARAKVQDVTYFATMMVQLGDADGMVS<br>GAIHTTADTIVPAFQIIKTAPGVSLVSSAFLMLLSDRVLVMGDCAVNPNPTPDQLAEIAVSTAQTARQFGLEPRVALLSYSTGSSGAGADVEAVTAATARVRE<br>LAPDLPVEGPIQYDAAVDPVVGQAKAPNSPVAGQANVLIFPNLNAGNIGYKAVQRSANAVAVGPILQGLRRPVNDLSRGALVEDIVNTVAITAVQAQAEG
gnl|extdb|pgaptmp_001620	K00925	ackA; acetate kinase [EC:2.7.2.1]	MPQTILVINSGSSSIKYQLVDPSIGVAMASGLVERIGEEMSTIHHRYSLKEVTINEPVPNHEAALQQVLDLFEEIGPQFEEAHIVGIGHRIVQGGRYFSGPTLID<br>DRVKRLIGELSSLAPLHNPPALKGIEVCQKLLPGIPNVAVFDTAFFQKLPNSSALYALDRDVAERYSIRRYGAHGTSHQYVSTRVTQVLGDKELKQIVLHLGNG<br>ASASAIVSGRAVDTSMGLTPLEGLVMGTRTGDIDPAVVFHLQRVANMSVSEVDNLFNKKSGLRGLAGDNDMRAVREMARSGNPRAREALEIYINRLVKYI<br>GGYAAEMGGVDVITFTAGIGENDIDLRREVCQRLEFFGVILDEEANNTRPREATTISRRDSSVRVMVVPTNEELAIATQAMTLV
gnl|extdb|pgaptmp_001621	K01950	E6.3.5.1, NADSYN1, QNS1, nadE; NAD+ synthase (glutamine-hydrolysing) [EC:6.3.5.1]	MSLYQHGFVRVAAASIPVHLARPQATAREVVAAAQQAHLAGAAVVAFPALTLTGVSAGDLFHQRVLLDEVEQALEAVRAASSELRPLLAVGAPLLVDGRVA<br>DCLVWIQRGQILGVSPNQTPPADSVHLFSTEANLDPALPPALGAAPVGALEFEFTDVPGLRVGAVLGQAQLETTPAGPATVWLYPGWESVRVDSYRRRVD<br>RLRAASELSGSALVSVQPGFGESTNEGAWDGSAVIVEHGQVLAEGTRWQRSSQLTYADIDVEALGQLRARRSPATHSEAWSQEPDLVVETTLGPLPDAELA<br>RDIPRFPFRVDEPEEVIQIQVAALVRRMTSIGTPKLILGVSGGLDSTLALLVCCEAMDQLGRPRADILAYTMPGFGTSSSTRQNAEVLSRTLGVSFSELDIRPTA<br>QAMLAELGHPVASGTEQYDVTFENVQAGLRTDYLFRLANLHGGLVVGTGDLSELALGWCTYGVGDQMSHYGVNAGIPKTVIQQVLAARATGELAEVLEAI<br>LATEISPELIPGQTTESLIGPYELQDFTLSYLLRFGFGPDKISYLAQRAWGEKYSAEQIEHWLGVFFRRFFANQFKREAGPDGPAVMPAGSLSPRAGWRMPSD
gnl|extdb|pgaptmp_001623	K01679	E4.2.1.2B, fumC, FH; fumarate hydratase, class II [EC:4.2.1.2]	MTEYRIEHDTMGEVRVPKDALYAAQTQRAVENFPISGQTLNPHHIAALGQIKRAAALANAELGVIGPEVSQAIVSAADEVIAGDHDDQFPIDIFQTGSGTSS<br>NMNTNEVVAHLASERLGSPVHPNDHVNASQSSNDVFPSSIHIAAYQAVEEVLLPGLQVLHDSLAAKSVEFAEVVKSGRTHLMDATPITLGQEFSGYAQQVA<br>YGMDRVRAALPRLAELPLGGTAVGTGINTPAGFSARVIELIAQHMGLPFVEAHNHFEAQAAQDSLVELSGQLRTVAVSLVKIANDLRWMGSGPRTGIGEIA<br>LPDLQPGSSIMPGKVNPVLPEATIQVAAQVIGNDAAIMFAGSQGNFDLLVMLPVMGANLLQSINILGRVSQTLATRCVDGIEANREQCLRYAQSSPSIVTPL<br>NRLIGYENAAKIAKHSVKENLTVREAAVDLGFVARGEITEADLDRALDVTTMTGQN
gnl|extdb|pgaptmp_001624	K00088	IMPDH, guaB; IMP dehydrogenase [EC:1.1.1.205]	MMVEVEIGRGKRGRRAYSLDDIALVPARRTRDPEDVSVSWQIDAYHVDIPLLAAPMDSVMSPRTAIEFGQLGGIGVLDLDGLWTRYADPEPLLAEVALLPE<br>REAAVRLQQIYQAPIQDELIYQRLQEIRAAGVVVSGRLSPQRVLQHWRTVVEAGVDLLVIRGTVVSAEHVSSLREALNLKRFIYELDVPVIVGGVTTYTAALHL<br>MRTGAAGILVGFGGGAARTTRQSLGIHAPMATAIADIAAARRDYMDETGGRYVHVIADGSVGDSGNMIKAIACGADAVMLGAALARAEEAPGRGWHW<br>GSEATHPQLPRGHRAHVGTVGTMKEILYGPSDRADGSLNFVGALKRTMASTGYSEVKDLQRVEVVVSPYHGSEDPHRKEQI
gnl|extdb|pgaptmp_001626	K02342	dnaQ; DNA polymerase III subunit epsilon [EC:2.7.7.7]	MNTWVTAPWLGFDTETTGISPTRDRIVSAASVVRIGGFLSTDPDQVQTWLANPGVPIPDRAAAIHGITTEQAVRDGRPAVEVLEEINATLAAHLSQGGPIIV<br>FNAAYDLPLLAADSARHGVPTLTDRLASFPGPILDPLVLDRELVRKRRGKRTLADLCAAYRIPMPDDLHQAHVDAQLTVTLMAAILEANPGLGRLSGPELVDY<br>QREAHARWAREFQAWLSQQGRRQHIDQNWI

gnl|extdb|pgaptmp_001627	K00088	IMPDH, guaB; IMP dehydrogenase [EC:1.1.1.205]	MGIYREDKFVGTGLTYDDILLLPELTDVIPSEVNTTARLSRNLTLRIPLVSAAMDTVTEARMAIAMARQGGIGIIHRNLPIAEQVDHVRRVKRSESGMVQDPVTVSKEATIDQLDELCGRYKVSGLPVLDNDGRLIGIITNRDLRFLPSSKWSTTTVGEVMTPMPLITAPVGISREDAKDLLAKHRVEKLPLIDDQGKLAGLITVKDFVKTEQYPNATKDKDGRLLVGAALGYWGDTWERAEALADAGVDVLVVDTANGGAQLAREMITRLKNHPDFQNIDIIGGNVATQEGAQALIDAGADAVKVGVGPGSICTTRVVAGVGVPQVTAIDMAARACSAADVPLIADGGLQYSGDIAKALAVGADSVMVGSLLAGCEESPGELVFSGGKQFKAYRGMGSLGAMSSRGRKSYSKDRYFQAEVTTDDQLVPEGIEGQVPYSGTLANNVRQLIGGLHQSMFYTGARTIPELQQRARLVRITSAGLRESHPHDVAITSQAPNYRSAERAS
gnl|extdb|pgaptmp_001629	K18955	whiB1_2_3_4; WhiB family transcriptional regulator, redox-sensing transcriptional regulator	MKDLSRLPGPLMEKWDWQYDGVCRDLDTEMFFHPEGERGAARRRRAAAAKAICATCPVLEQCREHALSAREPYGIWGGMTEEERRIYLGRRAAS
gnl|extdb|pgaptmp_001631	K04078	groES, HSPE1; chaperonin GroES	MSISITPLEDRIVIRQAEAEQTTASGLVLVDSAKEKPQQGEVLAVGPGRIDDNGNRITVDVAVGDTVIYSRYGGTEVTVDGEEYLILSARDVLAKLG
gnl|extdb|pgaptmp_001633	K06048	gshA, ybdK; glutamate---cysteine ligase / carboxylate-amine ligase [EC:6.3.2.2 6.3.-.-]	MEEATNHLDFAPSQRSTVGVEWELQLVDMDSNDLRQAANTVIKQAWQVPELKPLVHREMLLNTVEIASGAHTKVSDCMRDLRFAVTSLRPKTNELRADFAAAGSHPFAQPAYQRVTDSRRYAELVQRTQYWGRQMLLYGVHVHVGVESREKVLPIINTLLTYGGRLQSLAASSPYWAGQDTGYASNRAMVFQQLPTSGIPRQFTHWEELERYAADMKKAGVISTFDEVRWDIRPSPHLGTVEIRIFDACSNIREVEACASLAHALVDYCSSLVDAGEELPRLPDWFIEENKWRSARYGLDAQLITNASGQTEHVRDSLANLVEQLRPTARELGCEPGLELALEILEKGGSYTRQRAVAAAVPESPLDAVVSLLRAEMQEDRPLTAEEFLDTHWADAARGGTR
gnl|extdb|pgaptmp_001634	K25706	tsaD; tRNA N6-adenosine threonylcarbamoyltransferase [EC:2.3.1.234]	MAKKVVLGIESTCDETGVAVVRGDELLGQCTATSMDQFERYGGIIPEIASRAHLESFLPTLDQALQDAGVTLDEVDALAVAAGPGLVGSLTVGICAAKTLSATLGLPLYGVNHVIGHLAVDALVDGPFPERFIGLVASGGHSNLLLIEDIALSVRELGGTLDDAAGEAFDKVGRLLGLPYPGGPHVDRLARQGEPDAITFPRGLAHAKDRYPYDFSFSGLKTAVGRYLAANPLPEEDREDDREQAVANICRAFSESVNDSLTAKAVQAAKDFDCPTIVVGGGFAANSRLRELLVERADQAGVEVRFPPMKLCTDNGAQIAALGSALAEAGAPPSPMDFGPRTAMDLSQTYVA
gnl|extdb|pgaptmp_001635	K00240	sdhB, frdB; succinate dehydrogenase iron-sulfur subunit [EC:1.3.5.1]	MNLKLRVWRQNGPDAPGAMHEYEVRGVSEDSSFLEMLDILNEELFARGEDPIAFDSDCREGICGQCGVVINGIAHGPERTTTCQLHMRTFSDGDTITIEPWRAEAFPIIKDLVVNRSAFDRIIQAGGYITVNTGAAPEAHSQLVPKPTADLAFNAAACIGCGACVAACPNASAMLFTSAKVTHLGLLPQGHPENLSRVVNMLNQMEEEGFGGCTNIGECSAVCPKEIPLDVIATLNRQLGKAVIKGV
gnl|extdb|pgaptmp_001067	K00239	sdhA, frdA; succinate dehydrogenase flavoprotein subunit [EC:1.3.5.1]	MNWFTHLGARTKPAGPPAHLAQVRVGTALSAQIPPGDPAAAWATRTDSYRLVSPLNRRHFRVVVVGTGLAGAGAAAALGELGYQVDAVTYHDSPRRAHSVAAQGGINAARARRVDGDSLHRFVTDTVKGGDFRGREAEAYRLGQESTRVIDHLNALGVPFAREYGGSLATRSFGGVQVSRTYYSRGQTGQQLQLAATQALWRQVARGTVTLHSRTEMLDLVLAEGRAAGIIARDLVTGELTFLPAHAVVLATGGYGSIYHHSTLAVNCNATALWRAHRRGALFANPSFIQFHPTALPVSSKWQSKTILMSESLRNDGRMWVPAAPGDQRPPDQIPEAERDYFLERKYPAFGNLTPRDIASRAAAEQIGTGHGVGPLQNSVYLDFSDALDRLGQATLAERYGNLFTMYHHATGEDPTRQPMRIAPGAHFTMGGLWTDFDLMSSIPGLFVGGEAGSGYHGANRLGANSLLSACVDGWFVLPLAVPNYLAELGPTPLLTEADRPVASARQLVSEQLAALRSIGGTVGPSEYHRRLGEILTHTCGVERSEESLATGLRQVRELRAEFWSNLSVPGPEAGLNQTLERANRVADFLELAELMLVDAAERQESCGAHFRTEYQRGGEAQRDDRCWAGVSAWASAPLERGAPTMAPTLTQTLAPADPRPFVRHFEPLQFTDVKLSTRSYR
gnl|extdb|pgaptmp_001637	K00241	sdhC, frdC; succinate dehydrogenase cytochrome b subunit	MATKTVATKKRRAWTTGVFIKQLMAISGLFFVLFLLFHAYGNLKMFIGPEAYDHYAEWLKHDAFYPIFPHGGFIWVFRVVMLALILIHIFAAFYVWYNAKVARRHGYVVKKTAVDAYAARTMRFSGVALILLIVFHLLNFTTLTVNTWGGVEPSPYARMVGTFQNPFMFVVYLVFILVVAAHVGHGFWSAFQTLGWVRKETRRFMVGLSGVIAAIIFLMFMLPPLAIATGMIS

gnl|extdb|pgaptmp_001638	K03789	rimI; [ribosomal protein S18]-alanine N-acetyltransferase [EC:2.3.1.266]	MVAYRQLGLGDLDQVVRLEQEVFPQEAWSPELLAEELSGPHRYYLGAFEEGGQLLGYGGIAGVWDADLMTLGVVPAARGQGLGRALTEQLIEEARQHGCERIFLEVRASNRAAHELYRSCGFIELGRVRAYYRHPTEDALRMGLELVGR
gnl|extdb|pgaptmp_001639	K14742	tsaB; tRNA threonylcarbamoyladenosine biosynthesis protein TsaB	MLELCLDTSAGASVAVVEDGAVLARAREDNPRRHAEELGVLLETVCRAAGIDGPIRRAPWDRVCVGTGPAPFTGLRAGLITAQVFARTAGVELYGVPSLAIVARGALDLLPDGHEVLAVTDARRQEVYWARYRAAGPNGLEELSAPAVGRPESLGAQLRQGDAVLVGPGAHLVRAVLPASVGPVDEADAAVASRLVAAGLARGEDLPAQPLYLRRPDIHGAQA
gnl|extdb|pgaptmp_001640	K06925	tsaE; tRNA threonylcarbamoyladenosine biosynthesis protein TsaE	MTTWISEGAEQTRELGRRLGRMLRAGDLVLLHGDLGAGKTTFTQGLAEALGVRGPVVSPTFIVARIHESAGDGPDLIHVDAYRIQDELDLETLDLDTSLADSVTVVEWGAGKAEVLSEDRLEIDFLAEAATQDWTVAADERRVLTLKPVGADWAERLAKEN
gnl|extdb|pgaptmp_001641	K01775	alr; alanine racemase [EC:5.1.1.1]	MTNSGSDHFPGQIDVDLAAIRHNLRTLQQRARGSQLMAVVKADAYGHGRGPVALAAYEAGVRWFGVSQVNEALKLVAELTGAGVRDARVFAWLAAPNQDWEAALSAGLHLSASSTFTLESIAAAAQTLALTAPIHLKVDVGMGRGGARGDDFAHLALVAARLEAEGLITVEGIWSHLPEADDITEAGHETMAGQVARFERALAQAREAGLEPELRHLAATSGTLWYPETHYDLVRVGIGMYGLSPNPQVASSADLNLRPALSLGAPVILVKRLPAGAGVSYGATWRAERPHWVGLVPLGYADGIPRHGSNSAPVTVQTVDGPFLSQILGRVCMDQVVISLGEGDEPAARVGDWAVLIGSGPGEPSADEWARACGTINYEIVTRLAPTISRHYHQEDQP
gnl|extdb|pgaptmp_001642			MRVDLLIAILPSLLFGPLAIIIMYLGGDNRQQTLGEISGGFLVALVSVPFFAAGLTWQAALIGFVAGILLAIGIHYQIQSFYHVGVSRAMPISTAGQIVVLSVMGIIMFAEWRRPGALPVGLAGVALVTLGVVLANWSDRREVRATDLHWGRGLLALAISTFGLTSYILLLRYYSVDPLQVFLPLIGGSLAGALVLTSPRFTPELGPVDTRWSINTVRQMIPGVLWGLGVIVMQVSIARVGVATGFTLSQLGVIISAAGGVIILKERRTRRELWLMTLGIALLIGGAVLVGVAKALDAG
gnl|extdb|pgaptmp_001643			MNRVLSAGEVADLLPEPTEADHKYTRGTVTLVTGSSTYPGAGVLSVGGALGAGAGFVRFAGSERAADLVLSRFPEVVLAPGGADALVIGCGWDESLRDPARQALAEHAGPVVLDAGALLDRGLRAEVGTPAVLTPHPGEARRLWEQVGGGGPLPTDLTEAARALSGATGSTVVLKSARTLVATPDRVWEYRARTAWPGVAGAGDVLAGVIGSVLARHWQAQTTANLGDVVAAAVWLHGEAGAHAAGLTSAGAPTHPIRASDIIRGLAPAWQSLPRG
gnl|extdb|pgaptmp_001644	K00867	coaA; type I pantothenate kinase [EC:2.7.1.33]	MEFVDEIPGGRAPVAPFEEFTRAQWASLATRTPLPLTEEDVERIASLGDPIDLAEVDAIYRPLSAVLQLYVDGIRRIGMQQRTLLRGPARPPTPFVIGVGGSVAVGKSTVSRLLRLLLSRWPRTPQVELVTTDGFLFPNAELQARGLMQRKGFPESYDRAALLKFVAEIKSGAEEVTAPVYSHVTYDIVPGEQIVVRRPDILIVEGLNVFQPARVGPDTPGVSLADYFDFRIFVDAEVHNIEEWYVNRFLQLRGSAFTDPHSYFHTYADLSDAEAISTARGIWRSTNLPNLVDNIAPTKSRATLVLHKDASH
gnl|extdb|pgaptmp_001645	K03431	glmM; phosphoglucosamine mutase [EC:5.4.2.10]	MARLFGTDGVRGLANRTITAQLAVHLGEAAGRRLAGERKPDGSKPRAVIGRDTRVSGEFLDCAIAAGLAAAGVDVVRVGVVATPTVAFLSAHEPDIDLGVMISASHNPMPDNGIKFFAHGGFKLDDSIEDEIEALLGQDWERPVGADVGEIWEDPELAEKTYVEHLVRACGANLAGLRIAVDCANGGASRLGPEALRAAGADVVVINASPDGRNINDACGSTHPEQLQAITVSAAADFGVAYDGDADRCLAVDAAGNLVDGDQIMGALALDLKRQGKLHDDTLVVTVMSNLGLLLAMKELGVKTVQTAVGDRYVLEAMLEGGYSLGGEQSGHVIASAHATTGDGILTSLLLARVIKESGRSLAELVADIPRLPQTLINVPGVDRQRVDDPGLQARVREVEADLGDTGRVLLRPSGTEPVVRVMVEAATQDQADEAANRLVEAVREHLAL
gnl|extdb|pgaptmp_001649	K02996	RP-S9, MRPS9, rpsI; small subunit ribosomal protein S9	MAETTEVIELEEENAPSSYTSETPSAPQGAGQSITAPGAGLGRRKEAVARVRLVPGTGQWKINGRTLEDYFPNKLHQQLVRSPFVLLDVDGRFDVIARIGGGGISGQAGALRMGISRALNEIDRDANRATLKKAGFLTRDSRAVERKKAGLKKARKASQYSKR
gnl|extdb|pgaptmp_001650	K02871	RP-L13, MRPL13, rplM; large subunit ribosomal protein L13	MRTYSPKAGDADKKWYVIDATDQVLGRLSAQIAAILRGKNKATFAPHMDMGDYVIVINADKVVLTGNKLDQKKAYRHSGRPGGLKSVTYRELMAKNPERVIEKAVTGMLPHNSLGRAQAKKLHVYAGAEHPHASQQPTPYEIKQIAQ

gnl|extdb|pgaptmp_001651	K06173	truA, PUS1; tRNA pseudouridine38-40 synthase [EC:5.4.99.12]	MAGLSVADLQRLRLDLAYDGTFFHGWAAQPGLRTVQGDLEEALATVLRRPVQLTVAGRTDAGVHAAAQVAHLDVNEAEAGQLTVRRLQGLLDRLEDRYWRQGSFPERPGGTGDIVLRSITPVSQEFDARFSATARHYRYRIADRSSLRDPASRWSTWWSPSELDQELMNTGAQQLCGEWDFLSFCRPREGATTIRTLRHLTVTRDEADGTLAVEVSADAFCHSMVRTLVGALVEVGRGHRTLDWLAELVRHPSRQWGVPVVPARGLTLVGVDYPPREQWAERAREARRRRDSEESGTV
gnl|extdb|pgaptmp_001652	K00886	ppgK; polyphosphate glucokinase [EC:2.7.1.63]	MTSQPVLTLCIDCGGGGIKGSVVDQYGNLAAPAQRIQTPYPLPPNLLVEIVQRHADNLPAADRITVGMPGMIRHGRVIATPHYITKDGPRSKVIPGLVEEWKNFDMQLAVHQQLGLPTLVLNDAEVAGAGVITGTGLEMIITLGTGLGNAVFDSGHLAPHVEVSQGFVRWGLTYDSYIGEHERLRLGDAHWSRRVRRIVDGLRPMYLWDRLYLGGGNSKRITQSHLEKIGDDVVIVPNDAGIFGGVRVWEWQD
gnl|extdb|pgaptmp_001654	K03040	rpoA; DNA-directed RNA polymerase subunit alpha [EC:2.7.7.6]	MLIAQRPVLTEEVVNENRSRFTLEPLEPGFGYTLGNSLRRTLLSSIPGAAVTSIYIEGVEHEFRTIEGVKEDVSEIILNVKQLVLSSENDEPVLMYLRKQGPGEVTAADIVPPAGVEVHNPDLHIATLNEHGKLEIELTVERGRGYVTAAQNKDLDGKKSLIPVDSIYSPVLKVTYKVEATRVEQRTDFDKLIIDVETKPAIAPRDAIASAGKTLVELFGLARDLNIEAEGIEIGPTVTDETLAADLALPIESLNLQSRSYNALRRRGILTVGELVAHSEADLLDIRNFGTKSIEEIKESLASLGMVLKDSQMPGGADA
gnl|extdb|pgaptmp_001655	K02948	RP-S11, MRPS11, rpsK; small subunit ribosomal protein S11	MAQNSRANASRKPRRKERRNVTHGQAHIRSTFNNTIVSITDPTGAVIASSSSGQVGFKGSRKSTPFAAQLAAEAAARRAQEQGMKKVDVFVKGPGSGRETAIRSLQAAGLEVGSIQDVTPQAHNGCRPPKRRRG
gnl|extdb|pgaptmp_001656	K02952	RP-S13, rpsM; small subunit ribosomal protein S13	MARIAGVDLPREKRMEIALTYIYGIGRTRSQETLAATGVSPDLRVKDATEEDLVKLRNYIDQNFQVEGDLRREVQADIRRKIEIGCYQGLRHRRGLPVRGQRTKTNARTRKGPKRTVAGKKKA
gnl|extdb|pgaptmp_001657	K02919	RP-L36, MRPL36, rpmJ; large subunit ribosomal protein L36	MKVKPSVKKICDKCKVIRRHGNVMVICENPRHKQRQG
gnl|extdb|pgaptmp_001658	K02518	infA; translation initiation factor IF-1	MAKKDGVIEVEGRVIEALPNAMFRVELENGHIVLGYISGKMRQHYIRILPDDRVVVELSPYDLSRGRIVYRYK
gnl|extdb|pgaptmp_001659	K01265	map; methionyl aminopeptidase [EC:3.4.11.18]	MRRQKQSRLKSRTQIGYQREAGLVVAEVHRVLREAARPGVSLLELDRLAYEATVAAGAKPNFLHYQGFPATVCMSVNDVIVHGIPDERVLAEGDLVSFDCGAYVERDGKQWHADAAFTHRVGTVGKRLVQLDEITENAMWAGVAAVASAKRIGDIGAAIEDYVDEAGAGLDWTPGLIEGYTGHGIGNRLHEEPTVYNYRVRGRTEQVEPGLVICIEPMVVAGTIETKVASDHWAVLTRDGAPAAHWEHTVAVLDEGIAVLTTADGGVAGLAPFGVTPVVL
gnl|extdb|pgaptmp_001660	K00939	adk, AK; adenylate kinase [EC:2.7.4.3]	MTGIVMLGAPGVGKGTQAAKIVERLGIPAISTGNIFRTNIKAGTELGLLAKKYIDQGTFVPDSVTTPMVAARFTADDVKRGFLLDGYPRNLAQAHSLRDILAQEGLALDLVIELDAPEEVLVNHMMNRAKKEGRSDDKPEVFARRLAEYRERTEPIATYYADQDLLAVVDGVGTIDEVTARIFELPVLKGH
gnl|extdb|pgaptmp_001661	K03076	secY; preprotein translocase subunit SecY	MFRAFAQAFRTPDLRQKILITLLIMALYRLGTFVPAPGVSSQNINACLTQAEGTGLLDMFNLFSGGALLQLSVFALGILPYITASIIMQVMRVAVPRLDDLYQEGQSGQTKITQYTRYLTVFLAILQSTTMLTLAINGQMFPGCTSPLIPNGSPATYAFIILAMTTGTVVIMWFAEIITERGVGNGMSLLIFTSIVSTMPTQLADIWAGSGGWTKVLVIVVLILLVTLVVVYVESAQRRIPVQYARRMVGRRTYGGTTTYIPLKINMSGVIPVIFSSSIMMLPNMAAQFASPESGWAQWIQVNMNRTSPWYLLTEAVLILGFAYFYTSITFNPDEVADNLKRYGGFVPGFRAGRPTAEHLRYVVNRLTFAGAIYLAIIASLPSIIMIPLDLAPTQMPFGGTTLLIMVSVGLQTVK
gnl|extdb|pgaptmp_001662	K02876	RP-L15, MRPL15, rplO; large subunit ribosomal protein L15	MADNRRHDLNPGEGEVLKLHHLRPAPGAKTAKTRVGRGEGSKGKTAGRGTKGTKARYQVQPGFEGGQMPLHMRLPKLRGFKNPFRVEYQVVNLDKLQEVFPEGGKVTVDDLVAKRVVRKNQPVKVLGNGEVTLAFQLEVDKWSASAEEKITQAGGTISAR
gnl|extdb|pgaptmp_001663	K02907	RP-L30, MRPL30, rpmD; large subunit ribosomal protein L30	MAQLKVTLKKSTIGAKPNIKDTVRNLGLRKIGQSVIREDRPEIVGAIRTVRHLVEVEEVEK

gnl|extdb|pgaptmp_001664	K02988	RP-S5, MRPS5, rpsE; small subunit ribosomal protein S5	MAAPQRGRNSQESSDRAGRDGDRRGGRGGRQGDRRDNRRSEERNQYVERVVTINRVSKVVKGGRRFSFTALVVVGDGEGTVGVGYGKAKEVPAAIAKGVEEAKKNFFHVPMIRRSIPHLVQGEAAAGVVLLRPASPGTGVIAGGPVRAVLECAGIHDVLTKSLGSDNAINIVHATVAALKQLEGPEAVAARRGLPLERVAPTSMLAARAEGEAQKREEAEKVAAEAAAAGVNA
gnl|extdb|pgaptmp_001665	K02881	RP-L18, MRPL18, rplR; large subunit ribosomal protein L18	MSYAVKGKGKAIGRIRRHKRLRNKVSGTPERPRLSVTRSNRHMFVQVVDDTNAKTIVWASTMEPELRASSDEKTAKAHRVGQLIAERAQAAGITAVVFDRGGNKYHGRVAAVADGAREGGLTL
gnl|extdb|pgaptmp_001666	K02933	RP-L6, MRPL6, rplF; large subunit ribosomal protein L6	MSRIGKQPIVIPANVEVKIDGQQVDVKGPKGSMTVVIPSPISVAVEGNEILVTRPNDEKVSRERHGLSRTLIYNNIVGVTEGYSKGLEIVGTGYRAVAKGKSIEFALGYSHPILVDPPAGIEFKLDSPTKLTVIGIDKQLVGETAANIRKLRKPEPYKGKGIRYAGEVVRRKVGKAGK
gnl|extdb|pgaptmp_001667	K02994	RP-S8, rpsH; small subunit ribosomal protein S8	MTMTDPIADMLTRLRNANAAHHESVSMPYSKLKAAIADILLEEGYIEAVSVEDARVGKTLTLKLKYSGRRDRAIAGLKRVSKPGLRVYAKSTSLPKVRGGLGVAILSTSSGLLTDRQASDKGVGGEVLAYVW
gnl|extdb|pgaptmp_001668	K02954	RP-S14, MRPS14, rpsN; small subunit ribosomal protein S14	MAKTSMKVKAAREPKFAVRAYTRCTRCGRPQSVYRKFGLCRICLRELALAGDLPGVTKSSW
gnl|extdb|pgaptmp_001669	K02931	RP-L5, MRPL5, rplE; large subunit ribosomal protein L5	MTPRLKEKYQNEVRPALEEEFNYDNVMQVGKPIKVVVNMGVGEAARDAKVLEGALRDLAAITGQKPRVNRAKKSIAQFKLREGQAIGASVTLRGDRMWEFLDRLIATALPRIRDFRGLSPKQFDGNGNYTFGLTEQSMFHEINVDQIDRVRGMDVTVVTSATTDDEGRALLRKLGFPFKEN
gnl|extdb|pgaptmp_001670	K02895	RP-L24, MRPL24, rplX; large subunit ribosomal protein L24	MAAKIKKGDLVEVVRGRTSNEKQLAERNARREAEGLAPLTPGDKGKQGRVIKVFPAEQKVLVEGVNLKTRHVRQGQQGQAGGITTIEAPISIAKVALVDPETKKPVRVGFREEVVERDGRKRTVRVRVTRGGAKRGITAGKEL
gnl|extdb|pgaptmp_001671	K02874	RP-L14, MRPL14, rplN; large subunit ribosomal protein L14	MIQQESRLKIADNTGAKEILCIRVLGGSSRRFAGIGDTIVATVKDAIPGGNVKKGEVVKAVVVRTRKQTRRPDGSYIKFDENAAVILKNDGEPRGTRIFGPVGRELRDKKFMRIVSLAPEVI
gnl|extdb|pgaptmp_001672	K02961	RP-S17, MRPS17, rpsQ; small subunit ribosomal protein S17	MAEETTGTQRNQRKVRRGYVTSDAMDKTAVVTLEDRAKHPLYGKVVRKSKKVKAHDETNECRVGDLVLIMETRPLSKTKHWRVVEILEKAK
gnl|extdb|pgaptmp_001673	K02904	RP-L29, rpmC; large subunit ribosomal protein L29	MADKGLTTVELDAMDDAELRKELDKAKAELFNLRFSQAVGAQEDSGRMKTVRRNIARIYTVARERELGFRTAPSTEE
gnl|extdb|pgaptmp_001674	K02878	RP-L16, MRPL16, rplP; large subunit ribosomal protein L16	MLIPRRVKYRKQHRPGRGGMAKGGTELAFGDYGIQALGPAYLTNRQIEAARIAMTRHIKRGGKVWITVFPDRPLTKKPAETRMGSGKGAPEWWVAPVKPGRIIFELGGVDEALAREAMRRAQHKLPMKTRFVVREGGEN
gnl|extdb|pgaptmp_001675	K02982	RP-S3, rpsC; small subunit ribosomal protein S3	MGQKVNPTGFRLGITTEHRSRWFADSTTKGQRYRDYVAEDVAIRKMLADGLERAGISKVEIERTRDRVRIDLHTARPGIVIGRRGAEADRLRQDLEKLTGKQVQLNILEVKNPEIDAQLVAQGVAEQLAARVSFRRAMRKAMQSAQRAGAKGIRVQVSGRLGGAEMSRSEFYREGRVPLQTLRANIDYGFFEAHTTFGRIGVKVWIYKGDVTEREFARQQAEAHNRGGSRRGERRGGGRRQGRGNAPEQTAAPEAAQPVSNETASQSAAPAAEASSTGTEA

gnl|extdb|pgaptmp_001676	K02890	RP-L22, MRPL22, rplV; large subunit ribosomal protein L22	MEAKAQARFVRVTPQKARRVVNEIRGKSVLDAADILQFAPQRVAKDVRKVLLSAVANAQVKAESTGEKFNEAELWIRETYVDEGPTMKRFIAKAQGRAGRILKRTSHITVIVGTKDEKEGNR
gnl|extdb|pgaptmp_001677	K02965	RP-S19, rpsS; small subunit ribosomal protein S19	MPRSLKKGPFVDAHLQKKIDEQNEKGTKTVIKTWSRRSMITPDFLGHTFAVHDGRKHVPVFITEAMVGHKLGEFAPTRTFRSHDKDDRKGRRR
gnl|extdb|pgaptmp_001678	K02886	RP-L2, MRPL2, rplB; large subunit ribosomal protein L2	MGIRKYKPTTPGRRNSSVSDFAEITRTTPEKSLVRPLTKSGGRNSYGRITSRHRGGGHKRAYRVIDFKRNDKDGVPARVAEIEYDPNRTANIALLHYFDGEKRYILAPEKLRQGDVVENGPKADIKPGNCLPMRNIPLGTVIHAVELNVGQGAKLARSAGSSIQLVAKEGRFAQLRLPSGEIRNVDIDCRATIGSVGNAEQGNIRWGKAGRMRWKGHRPKVRGVAMNPVDHPHGGGEGRTSGGRHPVSPWGKPEGRTRKPNKPSDKYIVRRRKTGKKR
gnl|extdb|pgaptmp_001679	K02892	RP-L23, MRPL23, rplW; large subunit ribosomal protein L23	MTVELSKNPRDIVLRPVVTEKSSGLMDEGKYTFEVDPRANKTEIKIAIEKIFGVKVSKIATQNRQGKVYRTRDGIGKRKSVKRAIVTLREGSIDVFGAGN
gnl|extdb|pgaptmp_001681	K02906	RP-L3, MRPL3, rplC; large subunit ribosomal protein L3	MTTDTKQAAAPVKALLGRKLGMTQAWDENGRLVPLTVVQVDKNVVTQIRTAETDGYSAVQVGFEDIDPRRVTKPLAGHFAKAGVSPKRHVAEFRTADAADYELGQELDAAVFEAGQKVDVSGNTKGKGFAGVMKRHGFAGGPASHGAHKIHRKPGSIGACATPGRIFKGQRMAGRMGNVRSTVMNLTVQGVDSEKGLLLIKGAVPGPKNAVVMVRSAVKGA
gnl|extdb|pgaptmp_001682	K02946	RP-S10, MRPS10, rpsJ; small subunit ribosomal protein S10	MAGQKIRIRLKSYDHEVIDSSARKIVDTVQRAGATVVGPVPLPTEKNVFVVIRSPHKYKDSREQFEMRTHKRLIDIIDPTPKAVDSLMRLDLPADVNIEIKL
gnl|extdb|pgaptmp_000651	K00566	mnmA, trmU; tRNA-uridine 2-sulfurtransferase [EC:2.8.1.13]	MRVLAALSGGVDSAVAAALAVEAGHDVTGVHMALSAEPAACRVGSRGCCSVEDSADAARAAEIIGIPFYVWDLAEEFEQTVIEDFVREYRAGHTPNPCVRCNEFVKFRELAERAQALGFDAVCTGHYARRVDGPGGPELHRGYDELKDQSYVLAIMGREELSRVVLPLGEAPNKAWVRAEAERRGLGVSNKPDSYDICFIPDGDTSGFLRSHLGEEPGQIVDVEGNVLGEHTGYYQFTVGQRKGLRIERPADDGKPRYVLETRPETNQVVVGASTLLSVDTIFGMDPVWLVDDLTVDEAATASFGSTLGGRALADVDPAVITVQTRAHGTPAVVTEVSMQPDGQVRVHLAEPVRAIAAGQSLVLYRGTQVVGEATITRAVREAAQPISA
gnl|extdb|pgaptmp_000650	K04487	iscS, NFS1; cysteine desulfurase [EC:2.8.1.7]	MLTYLDHAATTPISDAAENAWREAVAQLRATPGNPSALHAGGRAAKRLLEDARERVARSLGADKNEVLFTSGATESDALGVMGAARGARRLDPARRRIVVSAVEHDAVAQQEAGAQRCDYDWEVLPVRPDGVSTFAVAEPETVAVASMSLVCAETGVIQPVRELVDEISGRAWVHTDAAQAIGQIPVSYADLGVDLLTVGGHKIGAPVGVGALVASRAVTIDTDRAGGGQERKIRSGTVDVAGACALAAALTDAVEFVSARAHHYEKLREHLLAGLPPEVHATTTAPSSPAIVHLSLPTAHPEILLLKMDEAGVMVSAGSACHAGVTRPSEVLLRMGRNEREALGVLRVSFGPENTKAEVHRFLRALPEALAAAQNLDRRDQ
gnl|extdb|pgaptmp_000649	K03522	fixB, etfA; electron transfer flavoprotein alpha subunit	MEKPIIVAVEREGEHGGLTKAMEQVIGAAHQLTTGPVWAVSAAAEPDLADLGAAGVSEVFIRAGGADPRLPAATAELVAAVIDERGPVGAVILPGTYWGKETCGHLSALIDGAAAVDVATVGEEDGQLVASKSVLGGTWTVTRFKLERGVPLVALTGSATFPLGATPTICQLTALAVAARPEVSAVQVVSARRQEGDASARLAEADIVVVGGRGAEDNFELVHEVAQRLGGAVGATRVACDEGLADRALQVGQTGITIAPRLYLGLGVSGAIHHTCGMQGSEVIVAVCDDPDAPIFELADFGIV
gnl|extdb|pgaptmp_000648	K03521	fixA, etfB; electron transfer flavoprotein beta subunit	MKVVVCVKHVPDGQGERRLEDGRIVRGEDDTLNELDEYAIEAAVSLVEEQGGEVIALTMGPADADEAILRALQMGADRGLHITDEQLAGLDAPGTAGVLAAAVRSLGDVDLVVAGMASLDGMTSLVPPALATYLGLPYLGLASQLELGNGQLEARRVVDGWEETLRAPLPAVVSVTDQVNEPRYPSFKALKAARAKPLDEVGWDDLAHFAPAGVRLASHSEVLEARPQERTGPHEIVEDTGDGGIRLAQFLRTHLN

gnl|extdb|pgaptm p_000647	K01214	ISA, treX; isoamylase [EC:3.2.1.68]	MLHSLSQAVLRPSPYLAPRDNPDPHRLGVYPNARGGLDIAVVAAHATGVDFCIFEGTGRGERERRWRLRGPVEGIWHGSIDGLFEGTAYGFRVFGPWDPD<br>NGLYHNPAKLLLDPYGRGLAGEPDLSPALHAHQVDHELYPAAYPLAPSPLNSARHAPRSVVVGNHFEVVPGPRISWDETVIYETHVKGFTRNFPDLPEDLQG<br>TYAGLAHPKVVQYLKDLGITTVELLPVHAKMDEPFLTDRGLTNYWGYSTLSFFAPEPSYATASARQRGPQAVIDEFRGMVSILHQAGIEVLLDVVYNHTCEG<br>GDAGPTVCWRGLDSLTYYRYTNDHPRHSIDETGTGNTLNFSNPRVVQMTLDSLRYWVREMGIDGFRFDLATTLGRLDNGYTPYHPLFVAMAADPVLREAK<br>LIAEPWDIGLGGWQTGNFPIPFSEWNDRYRDSLRTFWLVDFKNILAGRGGHGSSDLATRLSGSSDLFGRPPGPQRGPRASINFVSAHDGFTLADLTAYEHK<br>HNMANLENNQDGSSNNLSWNHGVEGISYATEMDVDMADSSGVVEDIVFARERSIRNLLATVMISAGTPMIVAGDEFARTQFGNNNAYCQDSPISWINW<br>DLTKSQQHLLQTSRYLLALRRRHPVLRPNTFLTGQAPDGDTMPDVSWFRADGSVPDGADWENNRTFQMRRSGYRHGDVDLLAVFNATLDTAEVTLPDS<br>HGSPWVLVVDSSWPRPKTGGITSVETALDQGEQFTPGQQVQMEPQSLQLYFSAPATGS
gnl|extdb|pgaptm p_000643	K00688	PYG, glgP; glycogen phosphorylase [EC:2.4.1.1]	MRTVLPNELAVLDTLARNLRWCWNEPTVKYFASLDPDAWERAGHDPVALLGEISPERFQQLAADSAVVEEAQQLAADLNLYLTEDKWYQSEFSNSDKPA<br>AIAYFSAEFGITTVMPQYSGGLGILAGDHLKSASDLGVPIVGVGLLYGAGYFRQSLSRDGWQRETYPLLDPHNLPLTLLREADGSAARISLPLPEGRVLNAQIW<br>VAQVGRVPLLLLDSDVPDNDDASRSVTDRLYGGSAEHRLEQELLLGVGGVKALRVYSRLTGTPRPEVYHCNEGHAGFMSVERIRELMEGEAQVDLGTAIEA<br>VRSGTLFTTHTPVPAGIDRFDKNTVRTYLSAMPLPGVDVEDILALGNESYEGGNPHVFNMAVLGLRMARKANGVAKLHGKVSRGMFASLWPGFDVPEVPI<br>TSVTNGVHGPTWRSPSFQSMALDYMSVLEDAEGNGWLKSEDQGGVPDEVLWNKRRELRSALVDSARERARAAWLERGASPAEIGWTGSILDPDVLTIGF<br>ARRVPTYKRLTLMLSDPERLTRLLTDPERPIQLVIAGKSHPDDEQGVGLIQKLVQFADNPKVRDRIVFLPNYDMGMAQVMMPGCDVWLNNPLRPLEASGT<br>SGMKCALNGALNLSILDGWWDEMYDGSNGWAIPTADGIEDPQRRDQLEAQALYDLIENTVAPRFYDRDERGVPRRWLEMMRHTLATLGPKVQATRMV<br>RDYVTNLYTPVAAASRTLDAAPWEQARDLAQWKAKVRGAWSGVHVDFVDAQVPDVAELGDRVSITAEVSLDGLDPEDVAVQVVSGRVGSDDRLEDFSIA<br>NLQADGVGADGRRRFMGEETLGVSGPVGYAVRVVPSHPLMDGDTELGLAVNAEPSSVRSHTHTTMGR
gnl|extdb|pgaptm p_000642	K16147	glgE; starch synthase (maltosyl-transferring) [EC:2.4.99.16]	MKQRLINRIPAIRVFPVVENGTFAAKSTEGEAFPIRATVFREGHDAFAAEAVLIDPDGLEYSRTLMYDIAPGLNIYEAWVAPDRPGLWSFRIDTWSDPWATW<br>KHNARAKIGAGVDVELMCEEGARLLERAASGANVAGRRPPSRPAYHKKKPGKLALVELSRAIEALRDRELPAGERLRVATSSTIEQVFSLSPLRDLLEATVRYP<br>LQVDRRTALYGSWYEIFPRSYGAYVDEDGNWVSGTLAGAAQELDRIKDMGFSVVYLTPVHPIGTTFRKGRNNTLTALAGDPGSPYGIGSPDGGHDAIHPDL<br>GTFEDFDNFVAKAERLGLEVALDIALQASPDHPWVREHPEWFTTRADGTVAYAENPPKKYQDIYPLNFDNDPAGIYQAIWDLIELWISHGVKIFRVDNPHTK<br>PVNFWQRFLAQMRAEHPDVIFLAEAFTRPAMMETLGMVGFHQSYTYFAWRTFKAELEEYFVEVAEKTAHLMRPAFWPTTHDILTQQMTTGGAAIFAIRA<br>VLAATGAPTWGIYSGYELVENVQRPGFEEQIDNEKYEYRPRDWARAEPLGISRLLTLLNRARAAHPALQQLHQISIHPTSHEDIIAFSKHVPARFSPTGEDDTV<br>LVVVSLNPHAEVEGTVYLDPARLRPALSAHEAAPGFGPGGLHLVDELDGIEYRWGYENYVKLSPWSRTAHVFGVRS
gnl|extdb|pgaptm p_000640	K00700	GBE1, glgB; 1,4-alpha-glucan branching enzyme [EC:2.4.1.18]	MIPVADSILTEVAHGRYHSPHSVLGAHLGDGSVTIRTVRHLADSVTVLTTDGATPAKHEKDGVWVAVLDRTDLPDYRLEIAYGDSVITVDDPYRFLPTLGEM<br>DTYLIGEGRHEQLWHVLGAHRKYYDGPMGPVEGVAFAVWAPNARSVRVVGDFNFWDGTGSAMRTLGESGVWELFIPDVAVGARYKYEICGPDGNWFQ<br>KADPMARATEVPPATASVVTESQYNWSDNSWMQTRGSRSPHSEPMSIYECHVGSWRQGLSYRELADELVPYLLNLGYTHVEFMPVAEHPFGGSWGYQV<br>TSYYAPTSRYGVPDDFRYLVDRLHQAGIGVIMDWVPAHFPKDAWALARFDGTPLYEDPDPLRGEHPDWGTLIFNFGRNEVRNFLVANALYWLEDFHIDGL<br>RVDAVASMLYLDYSRQDGQWRPNQYGGRENLEAIQFLQEANATAYRRNPGIVMIAEESTAWPGVTASTTGGGLGFGFKWNMGWMNDTLRYMREDPV<br>NRRWHHGELTFSLVYAFSENFFLPLSHDEVVHGKGSLLSRMPGDRWQKLANLRALYAYQWSHPGKQLLFMGQEFAQDNEWNEAYSLDWWLLDDPANR<br>GVSDLIRELNRVYRSAPALWDDDYTGFEWINAGDADHNVISYIRQGLDEHGEMDYLVCVINFAGNPHEGYRVGLPFGGLWEEVLNTDDALFSGSNVINVG<br>PLTSEEISWDGREHSVALRVPPLGATWLRPVR
gnl|extdb|pgaptm p_000639	K05838	ybbN; putative thioredoxin	MSDTYGAVDLSAISDAAIVDVTQANFEEEMNLSQSVPVVLLFYPAQDLGSQQVLTVLEASARKHAGAFQLGKVNVDAVPELAAALQIKSLPTSIALVARRPV<br>PLFEGPVTPEQFEALITELLQVAPQLGVTGRIENTNPAHEAPRAAEMQDDWEGAVALWKKVLANNPSDREAKQALARAEFQVRLAHEDESELARADRLFA<br>QGDEAAAYNLLLGLVAGEQREPARARLVELLNLGSDPAVVKQARSRLATMLMV

gnl|extdb|pgaptmp_000635	K01443	nagA, AMDHD2; N-acetylglucosamine-6-phosphate deacetylase [EC:3.5.1.25]	MSTKLWRATLVLTDQLLEDGAVVFEGENILWVGPAADLPAEYSDVEDQTPAGTTYVTPGFIDVHCHGGGGCSFPDATEVAQVETAAAEHLKHGTTTMVASLVTAAQDVLVERARTLAQAAQNGTIRGIHFEGPFLSEARCGAQDPRFLTDPTPAAMAELMEAAGGYALSMTIAPERTMSPVGVEALGVLTQAGAIPSWGHTDGGIEVTDEAIAEGVAQLSRRRASVTHLFNGMRPLHHRDPGPISSYLRAAADGEVVVEMICDGVHLDPELVGNIMETVGRDNCVFVTDAMAAAGMADGEYVLGPQKVRVEHGVARLAHSDSLAGGTSHILDCVRVAVTRGRIDLVDAVFLGTAQGARLFGWNDRGELEVGRRSDLCALTEDLALVAVARGGELI
gnl|extdb|pgaptmp_000634	K07503	nucS; endonuclease [EC:3.1.-.-]	MRLIVADCAVDYSGRLDAHLPKAKRLLMLKADGSVLVHSDGGSYKPLNWMSPPCRLEVVEPTTDEADEGVVQVWQVVPLKTDGQLRVRLYQIHAELDEDLGVDPGLTKDGVEAHLQELLALQLTSVLGEGWKLGRREHPTPIGPVDLLVYDPDGRPVAIEVKRRGGIDGVEQLTRYLTLLSREEHLREIRGIFAAQEIAKQARTLAGDRGIECVILDYAALRGMDDPEERLF
gnl|extdb|pgaptmp_000624	K02114	ATPF1E, atpC; F-type H+-transporting ATPase subunit epsilon	MSKRLSIEVVNRTSTLWEGEADYVSIPALDGRLGVLPGRQPVLAVLNTGNVEVRGADPGGEVNISVSGGFASVDDDFVTVVVDEGTVI
gnl|extdb|pgaptmp_000623	K02112	ATPF1B, atpD; F-type H+/Na+-transporting ATPase subunit beta [EC:7.1.2.2 7.2.2.1]	MAENDKKLAEGRVARVVGPVVDVEFPPDAIPALYNALLVELDLTGQGEGEGKTTMTLEVAQHLGDNLVRTVALKPTDGLVRGQAVTDTGSPITVPVGDITKGHVFNVTGECLNSDTPLEIKERWPIHRDPPHFDQLEGQTKMFETGIKVIDLLTPYVQGGKIGLFGGAGVGKTVLIQEMIQRVAQDHGGVSVFAGVGERTREGNDLIHEMEEAGVFDKTALVFGQMDEPPGTRLRVALTALTMAEYFRDEQKQDVLLFIDNIFRFTQAGSEVSTLLGRMPSAVGYQPNLADEMGLLQERITSAGGHSITSLQAIYVPADDYTDPAPATTFAHLDATTNLAREIASRGLYPAVDPLASTSRILDPKYVGEDHYNVATRVKQILQKNKELQDIIAILGVDELSEEDKITVARARRIEQFLSQNTYMAEKFTGVPGSTVPLSETIEAFRRVADGEYDHIPEQAFFNIGGIEDLERKWHELQKQ
gnl|extdb|pgaptmp_000622	K02115	ATPF1G, atpG; F-type H+-transporting ATPase subunit gamma	MAGQQRIYKQKIRSTETLAKVFRAMEMIAASRIGTARRRATEADPYTAALTRAVAAVTVHGRVDHPITQPRKDTNRVAVLAVASDRGMAGAYSATILRETERLLQELREDGKEPVLYASGRRAVQYFNFRHVPIKQSWTGESDNPDNEMMDEVAQTLLDAFLDPTDGVAEIYLVFTRFKSMVTQVPEIRKMLPLTVVDTRQIPGVDDSDREFKEDDAYPEYEFIPSAREVLNQLLPLYIRDRIRSAMLMAAASELASRQQAMHTANENANELITDYTRLANAARQADITQEITEIVSGADALTQQ
gnl|extdb|pgaptmp_000621	K02111	ATPF1A, atpA; F-type H+/Na+-transporting ATPase subunit alpha [EC:7.1.2.2 7.2.2.1]	MADLGIRPEDIRAALDSYVDSYEPASTAAEEVGYVTEAADGIARVEGLPGAMANELLRFADGTLGLALNLDPREIGVVVLGGFDSIEEGQEVRRTGEVLSVSVGDGYLGRVVDPLGQPMDGLGEITDIDGRRALELQAPGVMDRKSVHEPLQTGLKAIDSMIPIGRGQRQLIIGDRQTGKTTIAIDTILNQKANWESGDPQKQVRCIYVAIGQKGSTIASVRQTLADAGALDYTTIVASPASDPAGFKYLAPYTGSAIGQHWMYAGKHVLIVFDDLSKQAEAYRAVSLLLRRPPGREAYPGDVFYLHSRLLERCAKLSDELGGGSLTGLPIIETKANDVSAYIPTNVISITDGQIFLQSDLFNANQRPAVDVGISVSRVGGDAQIKAMKKVAGTLKLTLAQYRSMASFAMFASDLDATTRKQLVRGERLMELLKQAQSSPMPVEQQVVVIWAGSNGFLDDLELSQVLPFQDNLLDYVGAHSNVLATIAETGLLTDETEAELRRVVTECHDVFVDAAGEADAGAVEAERTEETIAVGKRRSDQD
gnl|extdb|pgaptmp_000620	K02113	ATPF1D, atpH; F-type H+-transporting ATPase subunit delta	MSAEQQTNQTAAEQTLLDALTSEVLSDQAGRQLFDLADLLDQEKSLLRALTDPGRSSADRQALARNLLQDRAVTPVVNALVQLAGQHWSRPDTFRKTVEDLGVRAILHGAKFAGELSRVEDELFALNQLASLERELRIRLSDVGGTNDEERQAITDRLLAGKVADPTLALVKRAVHISGRGRLLQTLRHFAERAAQAHNAQLVTVATATELTDAQLARLRDLIKTQVGGDISLAVSVEPDLIGGFRINYGDEAADSSIRSELGAARRALTR
gnl|extdb|pgaptmp_000619	K02109	ATPF0B, atpF; F-type H+-transporting ATPase subunit b	MNTVVFAAEVPVEPDGLSIVLPPLYEIFWSAIIFLILWLVLGWALPKIYRVLDQRQEQIETGLQAADTAKEEAALAQRERREIVRAANEEAREIRERADQDAKRIVREARVEATAEAQRITENATRQIEADRKSAELTLRRDVGELATELAEKIIGEQLKDRELSTRVIDRFMDELEADLKTTTAGVTK
gnl|extdb|pgaptmp_000618	K02110	ATPF0C, atpE; F-type H+-transporting ATPase subunit c	MTGSIATIGYGLATLGPGIGIGILVGKTQEATARQPEVAGRLFTNMIIGAALVEALGLIGFVLALIS
gnl|extdb|pgaptmp_000617	K02108	ATPF0A, atpB; F-type H+-transporting ATPase subunit a	MEKPKRRYPLWWWVGLVAIVVIIALTAIPGFWETGRAPGNSIHSPGMGDFFPETLIWDETAFEFNRLTLARLLVAVVLSAILVTVALRAKLVPGRGQATVEFLAEFVRKNIGIELLGHERGRKYAVALGTIFFGVLGMNLTGVIPGINIAASSVMSVPLVFAVFAYVTFIGAGIKARGGFRFFKEQLFPPGVPWPVYILITPIELFSTFVVRPATLAVRLLSNMIAGHMLLAITYFGTQSMVVAAGGMKPLAILTFSGSVVLTLFELFVAALQAYVFTILTAVYIKMSVEAH

gnl|extdb|pgaptmp_000614	K07566	tsaC, rimN, SUA5, YRDC; L-threonylcarbamoyladenylate synthase [EC:2.7.7.87]	MNATESTRAGQLIVLPTDTVYGIGANPFDQEAVTRLLAAKGRRETMPPPVLFARADEALAVADWGRVGERAARAAARLAQRYWPGALTLIVPTSAEFGWDLSLRGHTVAVRVPDQDQTRELLAETGPLAVTSANLTGEPPALTIEQARAYFGEKVSVYLDGGPARIGEASTIIDCSQWPPQLVRAGALDWEEIARDLGSLT
gnl|extdb|pgaptmp_000613	K02493	hemK, prmC, HEMK; release factor glutamine methyltransferase [EC:2.1.1.297]	MSSRPGPAPLTGLLRWGTNQLRRAGKPEQEARWLLEWALGVDTLLRAQPEVGARAAERYRSAIAQRRAGFPLQHITGTMQFRSLTLQAGPGVFAVRPETELLVEVADVHRGDLVVDLCAGSGAIGLAVAAEHSGVEVIGVELSPVAAAYARRNADQVSLAPGSSYRLVQGDATVALPELDGQVNLVLTNPPYVPGTPALTGEVLFDPEMALYGGGEDGLVLPRGIARRARRLLAPGGQLLMEHDERQGPSLCRIAEELGFTDPQTLPDLTGRPRFLSARVPA
gnl|extdb|pgaptmp_000612	K02835	prfA, MTRF1, MRF1; peptide chain release factor 1	MSSSDELQAVEPLLTEYHELEQLLSDPNLHNDQARARQVGRRYAELGQVVRAAEELRRVEEDLAAARELADEDASFAEEATELAARREEAHQRLLRILAPRDPDDACDVILEIKAGEGGDESALFAADLARMYLRYAEQKGWSVTEMSANHTDMGGYKDITLAIRAKGNPAPEDGVWAHLKYEGGVHRVQRVPVTESQGRIHTSAAGVMVMPELEVDDEVEIDMNDLRIDVYRSSGPGGQSVNTTDSAVRITHVPTGIVVSMQNEKSQIQNRAAALRVLKTRLAAEAKAQREAEASAQRLSQVRTVDRSERIRTYNYPENRIADHRTGFKAYNLDQVLEGSLEPVIQSAIEADEAARLAAAGAGKK
gnl|extdb|pgaptmp_000611	K02909	RP-L31, rpmE; large subunit ribosomal protein L31	MKQGIHPDYVETTVTCTCGNTFVTRSTIPSGEMRVDVCSNCHPFYTGKQKILDTGGRVARFEKRYGKRTK
gnl|extdb|pgaptmp_000610	K03628	rho; transcription termination factor Rho	MDQKNGVPSEADLNKLKLPELRALAGQLGLRGTSRMRKPELVAAIVGKPAPAADLLDSLPAPAPQTASAEASSGSSRPRRRVSTDSAVEPKSAAHALLDAPAKKEEISSRPARRPEKKSDTVDVRAAVAEDLQAVRARKQGDDSAPAEDVSRKLDSIDLPEGGEPNRSRGRRDRGRRRGRDRQDRPERTEQQSNGNNNSAPAEDLVPIAGILDVQANHAFVRTSGYLPGENDVYVTLGNVRRWGLRAGDAVMGAVRPAREGERRQQKYNALVRLDSVNGMSVEQATSRREFGKLTPEYPREQLRLETTPKALTPRVIDLVSPIGKGQRGLIVAPPKAGKTMIIQQIAKAIEVNNPDVHLMVVLVDERPEEVTDMRSIVKGEVIASTFDRPAHDHTMVAELAIERAKRLVEMGADVVVLLDSITRLSRAYNLAAPASGRILSGGVDAAALYPPKKFFGAARNLREGGSLTIIASALVETGSKMDEVIFEEFKGTGNMELRLSRQLADRRIFPAIDINSSGTRREELLLKPEELQIMWKLRRVLGSLDQQSALELVLGKLKETQTNAEFLMLVQKTTPAE
gnl|extdb|pgaptmp_000609	K00872	thrB; homoserine kinase [EC:2.7.1.39]	MRLRQDRVELSVAATATGLSGPPAHLALALGLHDRYVINAVAGHTRVLSYGLTGSFDPNTGEPAEEPTGETHPTIRGIRAGLDHFDASQVGVELHLQAGIPRGRGLGATSAGILAGLVAAHGLLDVPVEPQLVTELAVSLGAERVRVQAALAGGVVVALDPERPALSLRPRPELAPVAFVPDFARVGELPSPARLSLEAAGREAARLAWLALLLGNGDLPVELLLAATEDPVLLPAWAGAVPASHALIGWLRENGVVATLTGTGPTVLSLTAVSETLREAARRSGWHVMETEAASTALRCDFAPKL
gnl|extdb|pgaptmp_000594			MRSDEVDFEYERRFLVRRLPADLLGDQRPNVIVQTYFLAAGGYGLRIRLQATEARRHLPQDASGKEPIELFAQDFDLCMLTVKGPGNSGIRYEAERELDLNVGIEMSLLGGITLSKRRYGVWLDEDGWVIDQFSGENAPLIIAECERTSPVTNLIVPGFCLAEVTNDARFSNDSLVNHPYSEWAEQYQRELAQP
gnl|extdb|pgaptmp_000591	K01887	RARS, argS; arginyl-tRNA synthetase [EC:6.1.1.19]	MTPEELSDIIATVIATAAQEGTLRIPVEDLPTTVKVERPRSREHGDWATNVAMQLAKKAGLAPRQLAEILADELAEQPAIDQVEVAGPGFINLRLSAGSAGELARAIVTAGEEYGTNQAEAGKTINLEYVSANPTGPIHLGGARWAAVGDSLARLLRASGASVVREYYFNDHGSQIDRFSKSLLARARGQEAPEDGYGGQYIADIAARVQAEALAAGEPDPATLPDDQALEAFRSRGVDLMFSEIKRELHEFRSDFDVYFHEDSLHESGAVEAAIARLRERGVIFEAEGATWLRSTDFGDDKDRVLIKSNGEAAYFSGDVAYYLDKRSRGADVSVLLLGADHHGYIGRMMAMCAAYGDVPGENLQILIGQMVNLVKDGEPVRMSKRAGTVVTLTDLVEAVGVDAARYSLVRASMDSNLDIDLDLLTKRSNDNPVYYVQYAYARTRSVVRNADEHGVRDQLDYDASALDHPADKELLGVLAQFPTVVGMAAAEREPHRVARYLENLASTYHAWYGQCRVTPRAGEEVDAGHVARRQLNEAVSVVLRNGLGLLGVAAPERM
gnl|extdb|pgaptmp_001896	K05823	dapL; N-acetyldiaminopimelate deacetylase [EC:3.5.1.47]	MSIRDYRRDLHRIPELDFDLPQTLAYLRKALAKLPGTVIEPAPSSLCIYFDAGAPDTIAFRADMDALPVTEDPDREYRSEHEGKMHACGHDAHMAMLLGLCDFVGEHLDQLPHNVLAIFQPAEETIGGAEGICRSGIFQQYGVRAVFGLHMWPGLPAGTIASRPGPLMARSSEITVDVIGHSVHIAKADQGRDALMAAAHLLTRVEAQVQQVPGHRLLSFGYGYGGTVRNAVAGTAHLEGTMRSFSDQTFEQLRTILLSEARSVEAETGCQVLVDVTAGYPPVINDEALLARVEQLAPIHRIEDPQMTGEDFSFYQREVPGVFFFVGTGNEAALHSSSFDLDEAALDSGLDLLRTLLFLEGI

gnl|extdb|pgaptmp_000457	K06942	ychF; ribosome-binding ATPase	MSLTIGIAGLPNVGKSTLFNALTRASVLAANYPFATIEPNVGVVPLPDPRLNQLAEIFGSERIVPATVSFVDIAGIVRGASEGEGLGNQFLANIREADAICQVTRVFADPDVVHVDGKVDPAADIETISTELILADIQTLENRLPRLEKEVRAKKADPEVLATATKALELLEKGELLSGPAGSKLDPKILREFQLMTTKPFIFVFNMDSAAMEDQQLQEQLRQMVAPADAIFLDAAFEAELVELDEEDAKEMLAEAGQAESGLDQLARVGFHTLGLQTYLTAGPKEARAWTIHQGDTAPQAAGVIHTDFQKGFIKAEVVSFEDLVEFGSVAEARAKGRVRMEGKDYVMHDGDVVEFRFNV
gnl|extdb|pgaptmp_000456	K03527	ispH, lytB; 4-hydroxy-3-methylbut-2-en-1-yl diphosphate reductase [EC:1.17.7.4]	MSETLVPNLNTRKVLLAAPRGYCAGVDRAVEAVERALELYGAPVYVRKEIVHNKFVVETLTSRGAIFVEETDQVPPGSHLVFSAHGVSPQVRESAAARDLLTIDATCPLVTKVHREAVRFAREEYDIILVGHVGHEEVEGTQGEAPNHIQVVGGPDEVDQVVVRDPERVVWISQTTLSVDETMETVRRLRERFPKLQDPPSDDICYATQNRQEAVKAIAPQVEVMLVVGSANSSNSVRLVEVAKDHGAERAYRLDAASELQPEWFDGVTRVGLTSGASVPEILVRDVLDWLGEHGFGEVEEVRTQVETTTFALPRNLKNALVSEGLLPAQVPGGRVPSKNHTR
gnl|extdb|pgaptmp_000455	K03601	xseA; exodeoxyribonuclease VII large subunit [EC:3.1.11.6]	MNSYQPAPDPQRQLAPLAGQTTPDNPWPLRLLAQKMREYVDRMPALWVEAQIMEYKPRPGTRMAFFVIRDTDADVSINVTTFPGVVEAAGPGFEPGARVILQVKPNFWETRGNLSLRAGKILIEGEGDLLAQIEQLRRQLAAEGLFRPEHKVPLPFIPQRVGLICGRNAKAREDVVVNALARWPATQFEIREVAVQGERCVDEVSAAIQELDAHPEVDVIVVTRGGGSVEDLLPFSDERLVRAAFVCRTPLVSAIGHEEDAPLLDLVADYRASTPTDAARRIVPDVVELQTQLQQGMVRLRSAVDRRLARERELLSQLTSRPVMVHPGAPLEQQRQLIAAEQLRLGAALTRRVSREEGLLTGLQSSLRALSPQATLARGYTILRAPGGQIVRSANDLQRGDLLEGVLAEGTFIVKVMGVNPEGTIAPTAPTDADSDPTRL
gnl|extdb|pgaptmp_000453	K00847	E2.7.1.4, scrK; fructokinase [EC:2.7.1.4]	MPEANPTKDRAVIIGESLVDLIKYEGTDQVPVGHPGGSPYNVALTLGRLGQSVNLVTALADDDHGLQVLRHLHESGVRLGAGSYALDRTSTALAQIDSSGSASYIFDFSWVLPAEVPIPPTAAFVHAGSISTATEPGAGIVLETLRRARNQALITYDPNVRPTLTPDRDQVRARVEEFAACADLIKVSDEDLAWLYPESSLDEVARAWFELSPAQLVVVTCGGDGPTAWLRSGGKVSSPPQPVNVVDTVGAGDSFMGGLIDALWRRGLTFRAGADRLDHLDEAAVKDLLDEAGAVAAITVSRAGA
gnl|extdb|pgaptmp_000451	K11068	hlyIII; hemolysin III	MPVASLKPVEWIPGQPVSQKPRLRGWIHLVAAPLSTAASIVLLILAPTTATKWASAVYLASSLILFGVSAMYHLFYWTPPLQAFWRRLDHSNIFLLIAGTYTPITVALLEGTQRVVLLSIVWVGAALGILINLTWPSAPRWLSTLIYVVLGWTAIWFLPSFWTIGGPAIVILIIVGGVLYTIGAVVYALKRPDPWPHWFGFHEIFHLFTVLAWACQCVAAYLAIL
gnl|extdb|pgaptmp_000450	K03624	greA; transcription elongation factor GreA	MSEAKWLTQAQYDAIKNELQDRVERRRPEIARLIEAARQEGDLKENGGYQAAREEQSMNETRIIQLEEILKNSEVGETPADDGVVEPGMVVTAKIMGKEEKFLLGSRNAGGDLGITVYSPEAPLGKALLGASRGDTVSYEAPNGKEIVVEILEVIPYQA
gnl|extdb|pgaptmp_000448			MSTQGNAHVPGPPPVSRALRVAAAWSWRLLVVIGLVAVILWILNPVRTAVISMLIALLLAVLLNPLVNWLQRRLKMGKTGAAVVGFLVGVLLILGIFSLVANQLISNSRSLVNQTLEGINEFLLWLNDTALGAENTGVSDLLDDFQTQLMSFLRDHSSTIASEALSIASSAAGLVASAIVVVFALFFLLKDGRSMWIWFVRTLPESAREQTHEAGIRGWVTLSSYVRTQVQVAAIDAIGIGLGAFFLGIPLAIPITVLVFFFSFIPIVGAFISGGIAVFIGLVNNGLTTAVIMLVVVLAVQQIEGNLLQPLLMSHAVSLHPLAVVLVVTIGSLVAGIPGALFAVPLLAFINSVVLYLHDHDPMPSLATDIDRPGGAPGTLAQQIENSYRSGTSTVKVLRRKSESESAESESAGSESAG
gnl|extdb|pgaptmp_000447	K07304	msrA; peptide-methionine (S)-S-oxide reductase [EC:1.8.4.11]	MPELHPVLHTPVDYLPQPGEAVIYLAAGCFWGVERIFWQTPGVVATTVGYTGGHTSNPSYVQVCTGTTGHAETVRVVYDRSQITDGKILSIFWENHDPTQGNRQGNDVGTQYRSAVWTTDREQDEAAHQIREAYQQRLTAAGFGAITTTIEPFGPEQVFWPAEDYHQAYLWKNPNGYCNHGFNGVGCPVGLGA
gnl|extdb|pgaptmp_000446	K01802	E5.2.1.8; peptidylprolyl isomerase [EC:5.2.1.8]	MENVIVTGEYGDKPTLEFTSAVASPDLQVEVLEEGTGLVVQAGDTLVCHYLGQVWNGNHFDNSYDRGAPLTFQVGVGQVIQGWDDGLVGQRVGSRVLLSIPSYLGYGPYGQPAAGIKGGDTLVFVTDIVDAN
gnl|extdb|pgaptmp_000445			MSNPVFNRLEGEWAAKPGGPSTTQTAPAQPIYDPAAFAQAQQAYYEPAATAVETGRMTWDDVIVKTALNLGTLLVGAAASWYATVVNPQTGMVLMMVGLVAGLILALVNIFSRTVRPPLILAYSLAQGVALGALSSVTEQILPGVVLQAVVATGVVFAVTLLLFSSGKVRNSPKLTKFTLIALLGIIGSRLLIWVLGLLGVPGMQFGGEGINLLGIPLPILISLFAVVVGAICLIQDFDQARVGVEQGVPAKYAWACAFGIMVTVVWLYVEILNILSYVNSR
gnl|extdb|pgaptmp_000178	K07319	yhdJ; adenine-specific DNA-methyltransferase [EC:2.1.1.72]	MGDNLPVLQALPSESFQLIYIDPPFNTGRKQSRSTLQTKRSVDGGRVGFGGHTYESVRLNLQSYDDQFSNYWAFLEPRLIEAWRLLTPSGTLYLHLDYREVHYAKVLLDQIFGRESFLNEIIWAYDYGARTKRRWPTKHDNILVYVKDPNLYYFDSAEVDREPYMAPGLVTPEKAKRGKLPTDVWWHTIVSPTGKEKTGYPTQKPLGIIRRVITASSRPDDWVLDFFAGSGTTGDAAQQLGRKFLLVDDNPQSYDICTKRLPTAHFVDLSGGESPDKAGGLTF

gnl|extdb|pgaptmp_000441	K01524	ppx-gppA; exopolyphosphatase / guanosine-5'-triphosphate,3'-diphosphate pyrophosphatase [EC:3.6.1.11 3.6.1.40]	MTRVAAFDCGTNSLRLLIADLNPGGPDPLVDVSREMRIVRLGEHVDETGRLSSAAIERTLAAVDEYRELIDRFGAERLRFVATSAMRDAANGNELVEAVRARLGVTPEVIPGNEEAALSFAGATATLGGYPNTPILLVDIGGGSTEFVLGDDWVQASLSVNMGSVRVTERFDTQPENLAGIAAATQWVDQQLDLVEEEIDFGLIRSVVGVAGTVTTLAGQALDVPEYSPNVTHGAFLSWEQWGAAARFMIEEPVETKAALPFMPTGREDVIGAGALIWQQILRRVRRRTEDLGLDLGGAYCSEHDILDGIALSLAD
gnl|extdb|pgaptmp_000440	K09009	uncharacterized protein	MDFSLLGPTAGPATDEDLTTLEHQLGRVPRGVVGVAARCVCGRPLVVITKPRLDDGTPFPTTFYLTSPELTKACSRMEADLEMDEFNDRLAEDHSFQATYRRAHEDYLQRRAVLGEVAEISGISAGGMPMRVKCLHAVVGHSLAAGPGVNPIGDEALERISARGWWSAETCTCH
gnl|extdb|pgaptmp_000436	K01689	ENO, eno; enolase [EC:4.2.1.11]	MAFIEEIRAREILDSRGNPTVEVDVVLDNGVAARAAVPSGASTGAFEAVERRDNSDRYQGKGVEGAVEAVVDIIAPEVEGMDASDQRAIDRVMIELDGTKNKGKLGANAILGVSLAVAKAAAISAELPLYQYLGGPNAHVLPVPMMNILNGGSHADTNVDIQEFMIAPIGAPSFREALRWGAEVYHTLKGVIKERGLSTGLGDEGGFAPSLESNAAALDLIIEAIERAGFKPGTDVALALDVASTEFFKDGAYQFEGEARSTDYMIDYYEKLIADYPLVSIEDPLSEDEWDAWKKLTDAIGSRVQLVGDDLFVTNPERLTRGIKDGVANALLVKVNQIGSLTETLDAVEEAHRNGYRSMTSHRSGETGDTTIADLAVATNSGQIKTGAPARSERVEKYNRLLRIEEALGED
gnl|extdb|pgaptmp_000433	K01056	PTH1, PTRH1, pth, spoVC; peptidyl-tRNA hydrolase, PTH1 family [EC:3.1.1.29]	MSESIKLIVGLGNPGAEYANTRHNVGVMTVELLAERLGVSLRSHSSRTRTASGRLGVLPGGAPGPQVHLAVSTSYMNVSGGPIGRLADFLRIAPTEILVVHDDLDLPAHQLRLKRGGGEGGHNGLKSLSAHLGTKDYARLRVGIGRPPGRQDPASFVLTPIPTSERPEWDVTIQLAADVAEETVLRGLVAAQQDLHARS
gnl|extdb|pgaptmp_000430			MYLPPKTDDQEWLGVLIAIPEPWVTELTAARRELGDPAADCVPAHLTLIPPTPVNVDEREDVFRHLQHVASHFGPFRLSLSGTGTFLPTSPVVFLDVTEGADACMSLADELRSGPLDHQARFPYHPHVTLAHGLPEESLHRARAGWADFEASWMVPGFRLDSVDPNGRYNTRALFDFAL
gnl|extdb|pgaptmp_000429	K07058	membrane protein	MPGSEPTTRYGPNFVPHSADFRRVWRETTGIDRLKNLLELSLHLRPLRAFARFGTANGSLLAAGISYRALFSLTAAITLGGMVFSALLGSNEALRTAVVEAVNSWLPGLLKSGTHPDGLVDPATLGSGPGTTVAGVVSLGVLLFTATSVVASLSSAIRAMFALSTVREPFPVTVGQRFLGLVALLVGISSTAGIVFLTSWLGTRATRLGVEKTWFDSISTGLTWVAAVVINMVLVAVVIRWVAGVRPPRPDLLWGSGLAGLSTALLQLAGTSVVQNVRGPILTAATTLITLILWVNLQARVVLLASAWIANPPRLQTRVSRESRREGLRPNYVTLSSPESLEQ
gnl|extdb|pgaptmp_000426	K01491	folD; methylenetetrahydrofolate dehydrogenase (NADP+) / methenyltetrahydrofolate cyclohydrolase [EC:1.5.1.5 3.5.4.9]	MKYPWPAPGGALPLDGVKLAADVKEELKSRVEALAKEGVRPGLGTILVGDDPASQAYVNGKHRDCAEVGIHSIQVHLPETATQAQVERAIDELNADPTCTGFIVQLPLPRRLNQDAILDRINPDKDADGLHPYNLGRLVTAINRSPLCPQPCTPRGIIKLLQRGGVSLAGATVCVVGRGLTVGRPLGLMLTRKDINATTILCHTGTKDLKSIISEADVVIAGAGVPGLITADMVRPGAAVVDVGISRVDGRIAGDVAPGVERVAGQLTPNPGGVGPMTRAMLLANVVEAAERQVSQ
gnl|extdb|pgaptmp_000424	K05346	deoR; deoxyribonucleoside regulator	MIDRDRERAMLRAVELYYFEGFTQAAISERLGCTRWTVGRLLKEATDAGVVEINIHHERARRHDLEQRLECGFGLVRARVVPTGSTPRETMSRLAHAGADFLTDIRPRPRVVGVAYGRTMGAMARALPERWIQGVTVVQIAAAPANLDDAFVGSSIRMISRRGNGVSRTLPSVPVFATAEQKRRVEAQEEVATCLGLARKADVVFYSPGPAGAESLLIDSEGFSPAEAEQLRGGGAVAVIGNRLITAEGEPADAEVDQRTLGVGLDDLRQARLSVAVGGGADKHEAFRAIVRGRLANVLVIDSDTAEMLLEAGLADQNAEWSD
gnl|extdb|pgaptmp_000423	K00024	mdh; malate dehydrogenase [EC:1.1.1.37]	MTEPRIVTVTGAAGNIGYALLFRIASGQLFGPDVPVKLHLLEIPQAVKAAEGTAMELDDCAFPLLSGIEIFDDVNQAFNGTNVALLVGARPRSAGMERADLLAANAGIFGPQGKAINDGAADDVRVLVVGNPANTNAVIAQQSAPDVPATRFTSMMRLDHNRAISQLASKTGASVADIKNMVVWGNHSADQYPDVSYATVAGKPAAELVEEAWLADYFRPTVAKRGAAIIEARGASSAASAANAAIDHVHTWIHGTPEGEFVTAGVYSDGSHYGVPAGLSFGFPVIAKDGEYQIVDGLDLSAGTRAGIDHNIKALQEEFDAVKELGFIK

gnl|extdb|pgaptmp_000422	K01810	GPI, pgi; glucose-6-phosphate isomerase [EC:5.3.1.9]	MTNSAPIPAPTDPTRTPAWAHLDQLAEDFYPDLRHWFEADPQRARTWSFTAADLWVDLSKNLIDQEIVAALLELARETGVEEHRDRMFRGDRINVTENRAVLHTALRRPAGDVVEVDGANVVPDVHEVLNRMYDFADRVRSGQVTGISGKKLTSIVNIGIGGSDLGPVMVYEALRPYVDPELSCEFISNIDPNDAGEVVSRLDPETTLVIVTSKTFTTLETLTNARVVREWLLRSLRERGLVTDDANAAEAVERHFVAVSTALDRVAEFGIDPANAFGFWDWVGGRYSVDSAVGLSLAIALGPENFRDLLAGFHAMDQHFQNAPAAENVPLLMGLINVWYTGFLGADTHAVLPYSQYLHRFPAYLQQLTMESNGKSVRWDGSPVSYDTGEVFWGEPGTNGQHAFYQLIHQGTRLVPADFIAFANPTHDFRDGDNDMHELFLSNFFAQTKALAFGKTIDEVRAEGTPETIAPARVFGGNRPTTSIMGDRLNPRALGELIALYEHITFVQGAVWGIDSFDQWGVELGKVLAQQILPAVAGNKEVLAEQDPSTRSLIEYYREHRRH
gnl|extdb|pgaptmp_000421	K00850	pfkA, PFK; 6-phosphofructokinase 1 [EC:2.7.1.11]	MGDDLIVGIDPRTGEPARIGVLTSGGDAPGMNAAVRAVVRTALAAGAQPYAIMEGWIGALRGGEAIREMNWQSVSSILATGGTVIGSARCDEFRTYEGRNQAARNLLERGIDRLIVIGGDGSLSGADEFRREWPQHVAELAERGEISAEVAANHPRLTLVGLVGSIDNDLVGTDMTIGADTALERIITAIDQISSTAASHQRTFILEVMGRQCGYLPLMAAVAGGADYVFTPENPPAPGWEAEMAERLTLGRAAGRRESIILVAEGARDTNGDPITTEDVAAALREHRGEEPRISILGHIQRGGTPSAYDRWMSTLLGYAAVQEVLNPAAHGQACILGVRQNRVARIDLELAVANTRAVAKLITAGDYESARQARGRTFYAMGDIFRVLSDPPQEDPAPEREIRVAVLHAGGLAPGMNTAARAAVRMGIHRGWTMLGVDGSWAGLADDRVRELTWADVEGWAFDGGAELGTRRAVPPVESYYAIGRALERNRIDALLVIGGFKAYLAVREMVEERKRFPAFNIPLVLVPASIDNNLPGAELAVGADTALNNNVWALDRIKQSAAASRRCFVAETMGRKCGYLAMMSGLATGAEFIYLNEEPLTLDRLSHDGERLRRSFEAGRRLLLVVMNEETSQYYDRKFVARAFEAVGQGLFDVRHSALGHIQQGGAPTPFDRLLATRLVNQALIQLEEELAHNSIEVKYVGMTSSRIEAHPIEQMDRDVDLPNRRPRQQWWSMLQPVVEVVSLENSGSPVRQIPIADAQLQPITPA
gnl|extdb|pgaptmp_000420	K00031	IDH1, IDH2, icd; isocitrate dehydrogenase [EC:1.1.1.42]	MEKIKVVGPVVDLDGDEMTRIIWQFIKDRLIFPYLDLDLKYYDLSIQNRDATDDQVTVDAAEAVKKYGVGVKCATITPDEARVEEFGLKKMWRSPNGTIRNILGGVIFREPIIIDNIPRLVPTWTKPIIVGRHAFGDQYRATDFKVPGAGTITLTYTPADGSEPEVHEVVEIPENGGVVMGMYNFNESIRDFARASFAYGLQRGYPVYMSTKNTILKAYDGQFKDIFQEVFDAEFKDQFEAAGLTYEHRLIDDMVASSLKWEGGYVWACKNYDGDVQSDTVAQGFGSLGLMTSVLMTPDGKVMEAEAAHGTVTRHYRQHQAGNPTSTNPVASIFAWTRGLAHRAKLDETPAVAEFAQTLEQVVIETVESGKMTKDLALLIGPDQPWLTTEEFLTAIDENLQQKLA
gnl|extdb|pgaptmp_001788	K00075	murB; UDP-N-acetylmuramate dehydrogenase [EC:1.3.1.98]	MTNLAELTTFRIGGPAGSLVDVNGPQELAEAVRTADREGQPVLVLGGGSNLLVADEGFPGVVIRDLSQDIAVVDSSGCGGNSVRVSAGTNWDDFSAYCVQQDWMGVEALAGIPGTVGAAPVQNIGAYGQEVAETLASVRVLDRLTGRIEQLALFDLHLGYRDSVLKRSLVSERAGGGRLWGPTGRWVVLEAEFQLRNASLSSPVRYRELADHLGINLGERAPSARVREAVLDLRRSKGMVLDPSDHDTWSAGSFFTNPIVDEAVELPADAPRFPVEQRSLVNSIAGKAPVVPGLVKLSAAWLIAHAGFSKGYPAGAPASLSTKHVLALTNRGGAKATDVMALATEIQRRVADKFGVQLVPEPVLVSMKKA
gnl|extdb|pgaptmp_001789	K02913	RP-L33, MRPL33, rpmG; large subunit ribosomal protein L33	MRPKITLACTQCKERNYITKKNRRNTPDRLEMKKYCPRCNASTLHRETR
gnl|extdb|pgaptmp_000631	K10234	aglG, ggtD; alpha-glucoside transport system permease protein	MSVGASTLAKRSLTSRFGSLVAALIAILWTIPTFGLLVTSFRPEGLIKTTGWWTFFGDPQVTLENYHNVLFGSTSQGDLSNYFINSLVITIPATIAPLVIATMAAYAFSFMKWRGRDTVFVIVFAMQIVPLQMSLIPLLRIFSSSFGKDFPFMSIWVAHTAFALPLAIFLLHNFMAEIPRELVEAAEVDGAGHVATFLRVILPLMVPAIASFAIFQFLWVWNDLLVGLTFSGGQSLTAPLTARLQSMAGSRGQDWHLLTAGAFISMIVPLAVFFSLQRYFVRGLTAGSVKG
gnl|extdb|pgaptmp_001793	K09767	yajQ; cyclic-di-GMP-binding protein	MADSSFDVVSKLDRQEVDNAVNQAAKEVSQRYDFRGVDASIEFKGDTITLEANTAERVNAVLDVLQSKLVRRGVSLKVLDLEGREPQVSGKIYRLPIPLREGISSENAKKITKLIRDEGPKGVKAQIQGDEVRVSSKSRDALQDVIALLKNAKLDVALQFVNYR
gnl|extdb|pgaptmp_001794	K03799	htpX; heat shock protein HtpX [EC:3.4.24.-]	MRRVENSLKTLVLFLVMWGILLILGGIIAGATRSATWIWVFAGLGLASTLYTYWNSAQLALRQMRAYPVTREQAPGLYAIVEDLSARMEMPTPSIWIAPSQTPNAFATGRNPKNAAVCCTEGILQLLDERQLRGVLGHELMHVYNRDILTASVASAIGGLIASLAQFAFIFGGRERGGNPIAGLLMMILAPIAATMVQLGISRTREYSADADGATLTQDPLGLASALETIERGVSAAPMAPNPAHDTVSSMMIANPFRGQGVAKLFSTHPPMEDRIARLRAQAQR

gnl|extdb|pgaptmp_001795	K00528	fpr; ferredoxin/flavodoxin---NADP+ reductase [EC:1.18.1.2 1.19.1.1]	MNLKVAVVGAGPAGIYASDILSKSGLDVSIDLFERLPAPYGLVRYGVAPDHPRIKQIIVALYKILQRGDIRLLGNVEVGLDVSFAELEENYDAVIIATGADRDAPLDIPGIGLPQSYGAADFVSWYDGNPDYPRTWPLNAKEVAVLGVGNVALDISRILAKHPEDLLPTEIPPNVYEGLVANPVTDVHIFGRRGPAQVKFTPLELRELGKVPDVDLIISEEDFDFDEGSEEALRSSNQQRQVVKTLTQYASVDPEKHQASRRIHLHLFEAPVEILADEDGNVRALRTERTELVGDGNVRGTGVFHEWPVQAVYRAVGYYSSPIDGAPFDPVRGVVPNAGGRVLEEPGSHEVIPALYATGWIKRGPVGLIGSTKSDAQETIANLVADAEAGVLTSTGSPEQLLAMLEARGVPVVTWDGWEMLDAYERELGSEFGELPRTGGIRERVKVVSRLAMTTIARGEGRPETLIGEPGELGKPSAPERFDDYTGYKQ
gnl|extdb|pgaptmp_001799	K00341	nuoL; NADH-quinone oxidoreductase subunit L [EC:7.1.1.2]	MTLASTVGEATGPAAYAYLLFLVPGIVAALLLVLGRRADRWGHWLAVAASWFSFVLAAVIFWQMLQLPGAERRLSLDLFSWIPAGDFQVNFGTLVDPLSMTFVLLVTLVGSLILVYSVGYMEHDPDRRRFFAYLSLFIASMLILVLGNSYATLFLGWEGVGLSSYLLIGFWNQVPDNATAAKKAFIMNRVGDMGLLIAMMAMVANFSSVNFEVVNAGIPTVSEGQATVIGLFLLLAATGKSAQYPLQAWLGDAMAGPTPVSALIHAATMVTAGVYLIVRSGVVFLAAPVAMTAVAVVGVITLFVGALIGTAKDDIKKVLAASTMSQIGYMMLGAGLGPVGWAFAIFHLLTHGFFKSLMFLGAGSVMHAMNDGVNMRRFGALSKVMKVTWLTFTAGWLAIMGIVPFSGYWSKDRIIEAAFAAGNFGHAETPWIGWVYGSIAMIAAGITAFYMSRLYFMTFHGKARWDDETPHESKPIMTVPLVILALFSIFLGGALAGGRFTTWLAPSVGETVHAEPVVPVWFIQVGTTVLVIVAAVIAWWMFAKREVPVAVPEGNTFVRAARQDFYQDRFNEAVFMAPSVALMKGVTAADQYGIDGTVGAVGRFANWLGRLVAWTQSGYVRAYAGYILGGVVIALAVVLGFRL
gnl|extdb|pgaptmp_001800	K00340	nuoK; NADH-quinone oxidoreductase subunit K [EC:7.1.1.2]	MSLALYIALALVLFAIGAVTVVVRKSAIISLMGVELMLNSANLTFISFSRILGTVEGQVMAFFVMVVAAAEVVVGLAIIVAIFKSRATTDLDDIVSLRN
gnl|extdb|pgaptmp_001801	K00339	nuoJ; NADH-quinone oxidoreductase subunit J [EC:7.1.1.2]	MNSVIAWPEMSGLGEALLFWSCAVIMIVAALGVLMFRKAAYSALCMVAVMLGMAVLFFALQAPFNGAVQVIVYTGAIMMLFLFVIMMIGLGASDGFREQRRGYIIFAVVLGVALAVLTVGALLHSTVVGSGSIGLDPYSSAPITSLAVSLFQNHWFTIQLAAMLLITSAVGAVLLTHSDRLGVKFNQINTADARMTEFRVKGVHPGQQAAPGVYAHSNAVGTPAIAGDTLAPVPESVPRVLRLRGLEQPIGAVSPEAAEALQLVKAGRRDQTMWSSAPKVPQSQAWGMGGKAAPTGLHQ
gnl|extdb|pgaptmp_001802	K00338	nuoI; NADH-quinone oxidoreductase subunit I [EC:7.1.1.2]	MSENLRYEQDPKGPIGEFFAPVAGYGVTFSSMFRPTVTEQYPFEGPFVQPRFHGRHQLNYYPDGLEKCIGCELCAWACPADAIFVEAASNTPDEQYSPGERFGRVYQINYLRCIFCGYCIEACPTRALTMGNDFELAEYTRLDDIYDKKMLLAEVPEGAVAAPHPMVKGLSDGDYYRGEVKGSTREQIEWVREHRPDEPTLATAKAVDEGAEQ
gnl|extdb|pgaptmp_001803	K00337	nuoH; NADH-quinone oxidoreductase subunit H [EC:7.1.1.2]	MNLALMSAEPIPADFSQETWWLTLIKALFIVVYLILSVIMALWVERRGLGRMQTRPGPNVAGPFGLFQALADAGKLLFKEDIWTSRADKFLYFLAPAIMAFTAFSVMAVIPMGPNVNMFGHSTPLQVADMPVSALYILAITSLGLYGIVLGGWSARSTLPLYGAVRSSAQVISYELSMGLALVSVFLMSGSMSTSEIVSAQKPFWWVIPLFPAFVIYVISMVGECNRLPFDLPEAEGELVAGHMTEYSSMKFGWYYLSEYINMLNVSAVATTMFLGGWHAPFPFNLIPALSGGWWGMLWFILKVWLFMFFMIWTRATLLRFRYDQFMKLGWKILIPIALVWLVMVSVLQGVRLWSGASLPTIMGVIIGVLVVVVFLAFWLTDRKPKVEEEPGEFDAFAGGYPVPPLPGQALPPSPRAGRVPVTATKEDSDE
gnl|extdb|pgaptmp_001805	K00335	nuoF; NADH-quinone oxidoreductase subunit F [EC:7.1.1.2]	MSVFTEPGTLTPALTARWGQPESWTLASARKAGAYEGLARAQEMEPGEITELIKAAGVRGRGGAGFPTGLKWSFLPPDDGKPRYLVVNADESEPGTCKDMPLLMADPHLLVEGVAIASRAINCHHAFIYLRGEVVHVYRRLLEAIHEAEDAGLLGDLKITAHAGAGAYICGEETALLDSLEGRRGHPRLKPPFPAVAGLYQRPSVVNNVETIADVALIFRHPEGWYTTMGTEKSKGCGIFSVSGHVNNPGQFEAPFGITMRELLEYAGGVRDGHQLKFWTPGGSSTPIFTEEELDVPLDYEGVAAAGSMLGTRALQVFDETTSVVRTILKWSEFYQHESCGKCTPCREGTYWIKQIMLRLEAGKGVPGDIDLLDEIAGNIFGRSFCALGDAAATPIMSGIKRFREEFEAGLHTPAWELFPYEKSTIFAKGGAR
gnl|extdb|pgaptmp_001806	K00334	nuoE; NADH-quinone oxidoreductase subunit E [EC:7.1.1.2]	MSFSAEVEQRLRADAAEIIARYPSDHARSALLPILHLVQSVEGYVSADGVALASELLDISRPEVSAVATFYTQFKRHPNGEYNVGVCTNALCGILGGDEIYEALSEHLQIEHDETTADGKITLERLECNAACDYAPVMIVNWEFFDNQTPESAVALVDKLQAGEPVAPTRGPDQLRTFKEVSRTLAGFRDDLANQGPSAGSPSQRGLEVAREHGWEAPVLKGDSK

gnl|extdb|pgaptmp_001807	K00333	nuoD; NADH-quinone oxidoreductase subunit D [EC:7.1.1.2]	MNMKVQAAQGAEEVNLDDFTDVIAQGGDWEDILEEIRKVSTERVVLNMGPVHPSTHGVLRVVLELDGETVRETRVDIGYLHTGIEKNMEFRTFTQGVTFCTRMDYVAPFFQEVAYCLAVEKLLGVEAPERATLIRVLMMELNRIASHMVAIGTGGNELGATTMMTIGFRGREEILRIFERITGLRMNHAYVRPGGVAQDLPEGTVDYIRSLLPKVRRDIGEMQDLTLQNPIFKQRFIGVGYMPLSAMMALSMTGPSLRAAGLPTDLRKDQPYCGYENFEFDVPTYDSSDAYNRTLVRFDECYQSLRIVEQVLRRLEETDGAPVMVADKSIAWPAQLSVGADGQGNSLAHIANIMGQSMESLIHHFKLVTEGFRVPAGQVFQEIEHAKGIMGVHLVSTGGTRPFRVHFRDPSFNNLQSLSMATVGGQLADVVVTLGSFDPVAGGVDR
gnl|extdb|pgaptmp_001808	K00332	nuoC; NADH-quinone oxidoreductase subunit C [EC:7.1.1.2]	MSNDLQPTGDKEVQAPVRGFAEPEVIRVQQGSFGVQDAGDTTGFGGLVETFTLPGESARPYGGWFDSVVDILEELLAVDGVPVGEAIEKVVAEHDQLIIFVTREHLRRIARHLRDDQDLRFEMCLGVSAVNYPGDVGRELHGLYTFRSFTHNRSLSLEVAVPDADPHIPSIVDIYPGNDWPEREAWDLMGIVFDGHPALTRIEMPHDWVGHPQRKDYPLGGIPVQYRGATVPPADTRRSYN
gnl|extdb|pgaptmp_001809	K00331	nuoB; NADH-quinone oxidoreductase subunit B [EC:7.1.1.2]	MGIEEQLPSGIALTSVENVLGLARKHSQFPVTMGLACCAIEMMAAGTPRFDMARFGLEVFRASPRHSDLMIISGRVSQKMAPIVRNVYDSMPEPKWVISMGACASSGGMFNNYAIVQGADHIVPVDVYLPGCPPRPEALIHAILTLREQIGKEPLGANREKIARAAEKAALEATPTHQMKGLLA
gnl|extdb|pgaptmp_001810	K00330	nuoA; NADH-quinone oxidoreductase subunit A [EC:7.1.1.2]	MTNPYVPLLIMGAVALLVSLGGLGASAILGPKQRSKVKAQNYECGIDPGPQRIEDGRFPVKYYLVAMTFIVFDIEVVFLYPWAVTFADVGVFGLISMVLFIGLITVPFIYELRREGLDWT
gnl|extdb|pgaptmp_001811	K21401	menJ; menaquinone-9 beta-reductase [EC:1.3.99.38]	MADDQHADVIIVGAGPGGASAAYHLAKAGVDVLVLEKSTMPRDKICGDGLTPAAVSELTLMGVDTSSWIHNRGLHVIGGGNDLYFEWPDQKTLPGYGLARPRMDLDHELIQHAVAAGARLAEGHLVYEAVTNSLGRVTGVRARVGRGAGAREVTATGKLIVDAGGVAARLATSLGIEKKQNRPLGVAARTYFRSPRADDQWMESHLELWNGEPGKSELLPGYGWIFPVGNGLVNVGLGSVSSRSQATKLPYKEVFQQWTHHLPEDWGFTPENQVGPLRSAALPMSFNRKPHYSNGLALVGDAGGMVSPFNGEGIAPALKAGRLLAQAALQGLSRPTELSFDRALSAYPQQLADEYGGYYSLGRVFVALIENPKIMRFCTYYGLPRRRLMKLVNKLLSDGFERSGGDVDDRLITGLTKMVSKV
gnl|extdb|pgaptmp_001813	K02361	entC; isochorismate synthase [EC:5.4.4.2]	MEPELFVRIERTTTTDLLSLVPSELLCWIGPDRKLVGWGEALKIEFVGNHAIRDAARRWDDLVDHCVLTDLANSELARSAPIALASFGFAAHTPGYLIVPKVLLVQEGDETLIITASTEGPAESAPELTRHEFSTPQGLWTDPGRMTQSQWLTAVRRLTNLLQAGAASKVVLTRDIVVSAATEIDERFLVQRLTELYPTTWVYAVAGLVGATPEMLAAMNGTSVVSRVLAGTSAPGQGQELLDSLKNRSEHHFAVESVARALAPRAEKLNVPTEPKLLDLPNVTHLATDVTAEIKDANVLDIVDALHPTAAVCGTPTKLAFDILEDLEGTQRGRYSGPVGWIDASGSGEFGIALRCGQIEGNSIRVFAGGGIMPDSIPEVELAETRAKMRPVLEALGLED
gnl|extdb|pgaptmp_001815	K08372	pepD; putative serine protease PepD [EC:3.4.21.-]	MSDSYGPDRHPAEQPGDPAQRQWAPPARPVPDPTRPVYPGPTVPTLAPSVQPGTPPPSGPIPPPTSGPVFGAPVPRPSAPSKGPGWVGLIAAMAVTALVSVGATVAITDATGAVNQSTTTESTTPPVTSTSSGNPDWEAVAAAVRPAVVTIQSEGGGEAGTGSGVIIDQDGNIITNHHVVSNVLEGGTLTVSLNDGRLYSATVVGVDQTTDLAVIRLDNPPDNLTTARLSTSDDLQVGQPVMAVGSPLGLSDTVTTGVISALDRPVVVAGEQKNSSPENPFGFPLPGEETQQTEAVVTNAIQIDASINPGNSGGPLFDETGSVIGINSSIASMASGSSEAGSIGLGFAIPVDLVKLIADQIIQTGQAQHSMLGVQIGTGSAEVNGETRMGAVVAEVQPGTAAAEAGLQVGDVILQIGDHPVVSGPSLTGFVRRFSVGDTVQLQVARDGQLIEVGVTLQAKS
gnl|extdb|pgaptmp_001816	K02551	menD; 2-succinyl-5-enolpyruvyl-6-hydroxy-3-cyclohexene-1-carboxylate synthase [EC:2.2.1.9]	MTENPSILTAQTLIATLLAARVPVAAYCPGSRNAPFAYALAQAEAEGQIRVGTFSDERSAGFWALGMAKALAAQAGQPVPVPVFTTSGTAVTELHSAVEEARLQGLALVVITADRPHELRGVGASQTTIQEGIFPGVVAASIPAGRSGERVATAAPLTRLLRAACDTPGPVHLNVAFADPLVPASDLVLPTVPVPTYLATPPTLPSWDEVVDPSLWTVVVAGDSADPAVIAAATARQVPILAEPSSGATASPSWVPHTPLLLRYLYQQRDIQQVVVTGRPTLSRPVVALLGRHDVRKIVVSPRADYPDQSYTAQVVVAGLAASDGGAGDPGWLATWRAGADCVGQVVAEVSGAELNLLAISRLLWASQPEVALWLSASNAVRGFDLAAASPARAQVYANRGLAGIDGTVASALGLASGLGRPVRAVIGDLAFAADLSTLVQQPGLPADLQVVVLNDGGGSIFASLEHGGAPADLYERYFAVAPRMDVVALAEAAGWRAERVSRFDELEPALAAPVSGRSVIEVVMPRPDALLRELAARTEAALSRLHVSQLSPRVNPEN
gnl|extdb|pgaptmp_001817	K02549	menC; o-succinylbenzoate synthase [EC:4.2.1.113]	MSTSLMLTDVPLTEWSHPTRWEDLGIDRTIIFHLPLTTTFRGVNSREGLLVHGPHGWGECAPFLDYQPDEAAQWLRSALAQATRPGPAPVRTEVPVNVTIPVSSPAAAAARAAEAGCRTAKIKVADPRVDLDADAARVAAVAEVLADQFGADARVRIDVNGSWSRDEACAAIEFLQRAAGPVGGLEYVEQPCWEVEDLAAVRERVEPPIAADESIRRADDPFSVVGAVDLAVIKVAPLGGVRAALDLAGQLPLPVVVSSAIDTSYGLAAGVQLAAALPDLPHACGLDTARLLATDVVAEPLRSAGGNLDVARAARVLTAEPVAGAADRSVVERWLARTEAMLTEVNARD

gnl|extdb|pgaptmp_001820	K00611	OTC, argF, argI; ornithine carbamoyltransferase [EC:2.1.3.3]	MTDLSRYFAPGSHLLRETDFTAQQWADLIELAGILKEQKRTGTEVPYLRGRNIALVFEKTSTRTRCAFEVAAADQGASTTYLDPSGSQLGHKESVADTARVLGRFFDGIEYRGDRQSSVETLAELSGVPVWNGLTDEWHPTQMLADSLTMKEHAGGRNLSEVAYAYVGDARFNTGRSLLVNGALLGADVRIVAPEALWPPADVIAQAEEIAAQTGARITVTEDLAAVAGVDFVHTDIWVSMGEPAEVWAERIQLLRPYRVDEALMATAGPNAKFMHCLPAFHDLNTTIGRQVYQQFGLEGVEVTNEVFEGPRSIVFDQAENRLHTIKAVMVRSLGRI
gnl|extdb|pgaptmp_001821	K01661	menB; naphthoate synthase [EC:4.1.3.36]	MSSLPFVSDTFDPARWQEVPGFTFTDITYHRGRSRGPQDGAPTGAPLPWVRIAFDRPEVRNAFRPQTVDELLVALEDARTNSEVAAVIVTGNGPSAKDGGYAFSSGGDQRVRGKEGYQYGPAGDAQSRARHGRLHILEVQRLIRATPKPVIAAVNGWAAGGGHSLAVVCDLAVASAEHAQFMQTDANVGSFDAGYGSALLARQVGDRRAREIFFLARPYTAQQAESWGAVNQAVPHAQLEETALEYCRIIATKSPQAIRMLKYAFNLVDDGLAGQQMFAGEATRLAYMTEEAREGRDAFLEKRDPDWSQFPYYY
gnl|extdb|pgaptmp_001119			MRHKSAKFWIIGVAAIVVLGAAVLGYSLYFANRAAPNVTIAGQSITGMTKAEATSWLQQRVDATQVKVTLDGKETDVPLADLGYFVDVPKTVEQALSDRTSFVGQLKALFSKSNVEPVVTTNDEAQSGFQAELLTTAGEPATNAKVELQQNKFTVIPGQAGVEVDTKPAVAAAATAAKTLTTQTVALTTVPVEPTITTEAAQQVADDANRIVNLPIALTTTLNIYSPTPEQKAAWVKIPAPYQGQSEADTVPAPKVEVDQAAVTKWVTDTTTASNDEPQPGVRNVNVRGDVVAVVSDGESGWTANNVEALTKGVLQAVNSRTEYSQEITYDEVKNTEWTDQLIAPGSESLAYQAAPGEKWIDINLSNFTTTAYEGATPVRGPVYMVPGAPQMETVTGRYKIWLKVPSQTMRGTNLDGSTYETPDVPWAMYFHGDYALHGAYWRSSFGYGGPAGSHGCVNMPVDEAKWFYDWAPVGTTVVSHY
gnl|extdb|pgaptmp_001823	K01911	menE; o-succinylbenzoate---CoA ligase [EC:6.2.1.26]	MPASTTPEFPAEIIRVRLEQSSVDLLVEAIKQRLFGTVRRPLIVLGTEDKQVEKALQNLPPATLTSDVILATSGSTRGRPHLVGLSWEALRASAGRTIDFLGPARWLLPLPPHHIAGFQVLVRSVLLGVSPLVVGRTEDIPAAVAAAREPLITSLVPTQLRRLQGEDLSGLSRILVGGARLDPALARQCAHLPLVTTYGMTETCGGCVYNSRPLPGIGVRIESGLVHLSGPVLMDGYLGEPSPLVTLDGTRYLVTSDLGRMDEGRLTIEGRADHVIISGGENLSPGTIEEAIGSWRPDLNAVVIGVDDPDWGQVAVAALEGAGSPALVGPELRAAVSAQLGAHHAPRAVVFVGDLPLLSSGKVDRRRLVVTVTEMISKQDCWSVD
gnl|extdb|pgaptmp_001824	K02548	menA; 1,4-dihydroxy-2-naphthoate polyprenyltransferase [EC:2.5.1.74]	MSSHLPELPARPTWRDWLEGARLRTLPAAAAPVLVGAGAAAQLGAFSWGKSVLALLVALLLQVGVNFANDYSDGIRGTDQVRVGPVRLTASGLVPQRQVLALALGCFALAGLAGLALVAWAGTWWFLLVGALAIAVAWFYTGGKNPYGYLGVGLSELFVFVFFGLVATVGTNWVQAYAAPAWLWLAASGMGLASVSLLLVNNLRDIPTDREVGKTTVAVRMGDRASRLVFLALLVAASLLGAGGLALGASWVWALWLAVVLLVVSIPAALPVLAGATGPGLIVALRNTGLYTLIYGVLVGALLAL
gnl|extdb|pgaptmp_001825			MPRVMLFAFAIGVTLYALLDWGMNSKSQTPGGLSRWLWLAVIIIFPIIGPAAWVILRLVGQAERKRGPTAAPPPQRGAPDDDSEYLREWSDRIARRQRKSPPPEKPKDEDEED
gnl|extdb|pgaptmp_001826			MAFTTVHLLRHGEVDNPDGVLYGRLPQFSLTPLGREMASEVAQYLSLEERDITRVIASPLLRAQETALPTALAYHLPIEADPRLVEAGSTFQGENVNGNRWALAHPRNWSRYVRPLEPSWGEPYRLIRERMCSAISSAIDEAWGHEALLVSHQLPIVMVQRFIQGKPLAHNPLWRECSLASLTSLLFDDHTLVGWSYTEPAGHLLHEAADVTPGASVARVNEG
gnl|extdb|pgaptmp_000882	K01187	malZ; alpha-glucosidase [EC:3.2.1.20]	MQTDDVTSTQPTSDPNWWRQAVVYQIYPRSFADANGDGIGDLAGIISRVPYLRQLGIDAIWLSPFYPSALADGGYDVDDYRDVDPRIGTLAEFDHLVTALHEAGLRLIVDIVPNHSSNRHVLFQEALASPKGSPARDRYIFRDGLGPDQSEPPTDWVAAFGGSAWEPVGDGQFYLHMFAPEQPDWNWAHPDVHAEFLETLRFWSDRGVDGFRIDVANFLTKDLSEPLPTQAELDAHPRHLGNGRIDDRDEVHEIYAEWRELFNQYDPPRVAVAEAWTPANRRVRYARPDSLGQAFNFDLLRANFDADEFRQIIAENLALAAEAGSSTTWVLSNHDVVRHATRYALPDAPDTQDRDRRWLLQGDVDLVEDRPRGLRRSRAATMTLLALPGSTYLYQGEELGLPEVRDIAPDQRQDPTFFRSPGVDVGRDGCRVPIPWTREGSSFGFGSNGAHLPQPPEFGQYSVEAAEAKPDSILHLYRSALALRRQLESGEELSWLEAPAHVLMFARPGGWVNVTNFGVDEVEVDGGEILLASQPVTLSEAGKLRLPSETTVWLRRR
gnl|extdb|pgaptmp_000974	K00928	lysC; aspartate kinase [EC:2.7.2.4]	MADLLVAKFGGSSLASSEQFAKVKQIVEANPQRRYVVPSAPGKRDSTDHKVTDLLYMCHQLAEHNIDCTEVFATIAERIEEIHDRLDLEVDISSELQRIFQQISAGATADFVASRGEYLSGLLLADYLGVPFVDAAEVIHFDDDATLNAERTATALAQMAQTHERAVIPGFYGSGPDGQIFTFSRGGSDVTGAVVAAGVDADLYENWTDVSGFLKADPRLVSNPSPIGRVTYKELRELAYMGAPVLHEEAIFPIRAKGIPIHIRNTNEPDAAGTLIVDDRAADGFHGVTGIAGRPDFTVISIEKTLMDDERGFFRKLVSVFETNGVSISHMPSGIDSVSVIVPAEEVRFKLNKVLEEIRIYLHPDTITVQDEIALLAIVGHGMIRTKGVAAKVFTALAQAGVNIRMITQGASELS

gnl|extdb|pgaptm p_000544	K01126	E3.1.4.46, glpQ, ugpQ; glycerophosphoryl diester phosphodiesterase [EC:3.1.4.46]	MIPVAANPYPHIFAHRGGADEAPENTLSAFRQAQSLGVRYLETDAQLTADGVVVLSHDEVVDRCYDGTGPISSFTWDELSKLRNSAGEAMPRLDEALAEFP DLYFNIDAKTDEVAEPLLETLTEAGALGRSLIASFSEKRLNRIRELGGEELSTSLGVAAVVRLMLAAETVSAAETWRVDGPGRSVRAVQVPEKTRGIRVVNPRFI ATAHTAGLAVHVWTVNEPAAMVRLLDWGVDGIVTDRPTMLREILRVRGQWSE
gnl|extdb|pgaptm p_000542	K03699	tlyC; magnesium and cobalt exporter, CNNM family	MSPWWGLGLTVVFLAINAFFVGAEFAVTSSRRSQIEPLVAEGRRGSAQALYALEHVSLMLAICQLGITVMSTSLGVIAEPALAHLLAVPLEALGASSVTVHAIS FVLALLIVLYLHVVFGEMVPKNISIANPQKLLLWLAPPLVTIGHVIRPLVVAMDKTANWFLRRFHIEPSAEISATFTVEEVANIVQISQEEGKLTDELGLLTGTLE FSAEQARGVMVPLESLTTVQDTCTPAELERAVAQTGYSRFPVERGGQIIGYVHVKDVLYADEETREQVIPEWRIRPLIRLEADEEIEEVLRQMQKSGTHMAA VVPDLDTESEDLLGLIFLEDVLELLVGEVRDSLQRTIGPPA
gnl|extdb|pgaptm p_000541	K03699	tlyC; magnesium and cobalt exporter, CNNM family	MLVDVLLILLGIVLTFGTAVFVAAEFSFVALDQASVAQKAETGGARINLVLKGLRTLSTQLSGAQVGITLTTILLGYTTQVALADLLTEWMGGWGWAVAGLV AGLVAALLVNGFSMLFGELVPKNMALADPLKTASLVAPLQLGFTWLFKPVITMLNGSANFILHRFGIEPQEEISSARSAAELEALVRHSAELGTLDEGTADLLT KSITMEGLTAQDVMTDRGRMTTLTEESTAADVVEAARASGHSRFPIIGSTSDDVVGLVNLRRAIAVPYERRGEVPVMSSSLSIPPPLVPETMRLAPLLVQLRD EGMQMAVVVDEYGGTSGIVTLEDVVEEIVGEVSDEHDRRRLGIRTAGPGRYRVPGTLRPDELADQTGIDLPEDGHYETLAGLVISHLDRIPEVGDRVEVNGV
gnl|extdb|pgaptm p_000540	K01616	kgd; multifunctional 2-oxoglutarate metabolism enzyme [EC:2.2.1.5 4.1.1.71 1.2.4.2 2.3.1.61]	MTTNIESEHSDWGPNEWYVAQMYEAFIASPDSVSPAWREFFTRKPEEGVNAVAHQGTEALLSESTVSAEEAPAGEETNVVSVAKSDLPPAPPTAVSEATSP YTRTHGQSSPAARRGQAAPEQAVARLKGSARAVVKNMEASLAVPTATSARQIPAKLLIENRAVINAHLERTVGGKVSFTHLIGYALVEALVEMPSMNVRYLE QDGKPSIENFAHVGLGLAIDVPRADGSRSLLVPVVKEADTLTFLQFRDACNDLIVRARDGQLSAADFQGASVSLTNPGTIGTTHSVPRLMPGQGVIIGVGAT DYPAEWAGLPLERLAQIGVGKIMYVTSTYDHRVIQGAGSGEFLRLVHRKLTGEDGFYDRVFSSLKVPYPPYRWERDVVYDAERDKGKPARIVELIHAYRSRG HLAADTDPLAYRVRHHPDLELSRYGLNVWDLDRVFPTGGFGGTNQMMLRDLLRQLRDTYTRSVGIEYMHIQDPAQRLWVQKEVEQPYQKPSREEQHQIL ETLIHAEAFEEFLQTKFVGQKRFSLEGGESLIPLLDAVLNSAARSGLNEVAIGMAHRGRLNVLSNIAGKSFAQIFSEFEGQKHKDQYGSGDVKYHLGTEGVYSA DDGVATNVYLAANPSHLEAANGVLEGVVRGKQDELGDPSFPVLPLLIHGDAAFIGQGVVSEIFNMSQVDGFRTGGTLHVIVNNQIGFTTGPTAGRSTRYPT DLAKGMQLPIFHVNADDPETVVRMARLAFKYREEFHKDVLIDLVCYRRRGHNEGDDPSMTQPVMYKLIDSIPSTREVYTRNLVGRGDITLEEATEYHRQYEN QLSSILEETREHGWEPEANERHGLEVPTAQLPGHGMMIGWSSAAPAEQLERVGEVQVAFPAGFEPHKKIRQLGEKRLAMARGEVPVDWGFAELMALGTL LMDGTPVRLTGQDARRATFVQRQATLHDQNDGREFTPLNFLVEDQAPFYIYDSTLSEYAPLAFEYGYSIERPDTLVLWEAQFGDFANGAQTVIDEFISSAEQ KWGQQSSLVMLLPHGYEGQGPDHSSARIERYLQLAAEDNLRICQPSTPANHFHLLRRQAYLRPRKPLIEFTPKQLLRLSAATSSLSEFTSGEFQPVLGDREFDG PNVRKVLLCSGRIYYDLAKERAKQGRTDIAIVRLEQLYPLPLVELTAALTNLGHADLVWVQDEPQNQGPWPFLALNLFPELKRKVSVVSRAAASAPATGLGW
gnl|extdb|pgaptm p_000538	K03088	rpoE; RNA polymerase sigma-70 factor, ECF subfamily	MEPLDTPPLLEAGPGSERDKILTQEAVFAEEALPLLDQMYGAALGLTRNPTDAEDLVQETFLRAYDRFDQYTPGTNIKAWLYRILTNLYISRYRKVQREPAQD ELEESGTTDGRSAEAEAIAALTEEQARAAVDNLPETFRLPVYLADVEGFSYREIAEMLEIPPGTVMSRIHRGRKMLRSALMEVAAEYGIGI
gnl|extdb|pgaptm p_000536	K00800	aroA; 3-phosphoshikimate 1-carboxyvinyltransferase [EC:2.5.1.19]	MARWMAPARRGPLRASVTLPGSKSQTNRALLLGATARQPLEITGALLSRDTLLAARALQQLGVRFTGDLTVHAPERFTLSGQIDCGLAGTVMRFVPALAAF GEGTVRFDGDEAARTRPLKDLLDALVALGARVEYHGEPGSLPFSLTGRTGEFPTEVTLDSSATSQYLSALLLAAPAAPQSFTVSLTGTVPSAAHVEMTTRMLA DQGIPVRYDHGYLASATRPAGVPITVEPDLSNAGPFLAATLICGGQVTLRHWPAESTQAGADWASLLPLFGAEVIEHGSDLVVRAGSAPWEGVDLDLGRIG ELTPTIAALCTLATTPSRLRGIAHLRGHETNRLAALVTEIRRCGGEAEETETGLIIRPGQLHPADFRAYADHRMATFGALLGLALPGSSVDDIACTEKTLPQFPAR
gnl|extdb|pgaptm p_000535	K06949	rsgA, engC; ribosome biogenesis GTPase / thiamine phosphate phosphatase [EC:3.6.1.- 3.1.3.100]	MGRQDSGTDDPRVKVRAGKGSRPRSKRRPDYSKAARGQVVAIDRGRYTVYVDGVRVQCVKARELGRGAVVMGDLVHLTGDLSGTGGTLARIVEILPRHG VLRRSLEEVPGARGEKIVVANADQLVVVTAAADPTPRAGMVERCLVAADEAQIPVILCMTKTDLADPAPFLRQFAGFDLQVVQTQRGGDLSELAEALAGRF SVLVGHSGVGKSTLINELIPGADRQVGEVNELTGKGRHTSTSAVALELPAGGWVVDTPGVRSFGLSHAEEENVLRVFPGTLAATEYCLPNCSHLAGEPSCAL DTWARGEAPFDEGDLAWRQDLVQRARGLLEAVATPSP

gnl|extdb|pgaptmp_000534	K05602	hisN; histidinol-phosphatase [EC:3.1.3.15]	MDDPRKYLDDLRLAHYLADLADDLTMKRFGAVDLQVEAKPDLTLVSDADRAVEDMIRNHLATSRSRDAVVGEERGATGSSRRQWIIDPIDGTHNFVRGVPVWATLIALVEDGDVVVGLVSAPALHRRWWAATGLGAHTGTSLQRCRQIHVSKVRHLDEAFLSYSSLDGWLEADRGREFLRLQSSCWRTRAFGDFWSYMLVAEGAVDLACEPELALYDMAALVPIVREAGGRFTSLEGEEGPWGANGLATNGHLHEAVLAALTVTE
gnl|extdb|pgaptmp_000533	K01951	guaA, GMPS; GMP synthase (glutamine-hydrolysing) [EC:6.3.5.2]	MPGRFVMISSREQEVLAHNERQALLGFTGLTEHELRWVRLEHEPFPALKLDEWDGIILCGSRFDSSAPEESKSDWQLEVEAGLRELYGRVLEADFPFLGLCYGMGTLNTYLGGVVDSTYAEEISAPELTLTDEGQRDPLLTGVAPRFRAYVGHHEAITELAPGLVTLVSGKQAPIQMIRAGKNVYATQFHPELDQAAIELRIDIFSDAGYYPLEERARIEAGVRGVNTRSAHRVLRNFAQLHQESRHLR
gnl|extdb|pgaptmp_000530	K03070	secA; preprotein translocase subunit SecA [EC:7.4.2.8]	MSLLDKILRAGEGRTLKQLDRIADQVDALAEEFSAMSDEELQGKTAEFKDRLEAGETLDNLLVEAFATVREASWRVLHMRPFHVQVMGGAALHRGNIAEMKTGEGKTLVATMPSYLRALSGKGVHVVTVNDYLAKYQSDMMGRVYRFLGLTCGCILNGQTPDERREMYNCDITYGTNNEFGFDYLRDNMAQRQEDMVQRGHNFVIVDEVDSILIDEARTPLIISGPAAGDLNHWYTEFARMVRTMERDVDYEVDEKKKTVGVLEAGIDKVEDQLGVENLYEAANTPLIGFLNNAIKAKELFHRDKDYIVQNGEVLIVDEHTGRVLPGRRYNEGMHQAIEAKEGVEIKAENQTLATITLQNYFRLYPEGSRAGMTGTAETEAAEFASTYKIGVVPIPTNKPMIRVDQPDLVYPTLEGKLRAIVDDIQERHRNGQPVLVGTASVESSELVSSLLRQRRIPHQVLNAKQHEREAQVVAMAGRKGAVTVATNMAGRGTDIMLGGNSEFIAQANLEAAGLDPVANPEEYRAAWPEALEAAERAVAAEHDEVVELGGLYVLGTERHESRRIDNQLRGRSGRQGDPGESRFYLSMEDDLMRLFNSSMAQRIMASGAYPEDMPLESKVVSRSIQSAQSQVEARNQEIRKNVLKYDDVMTGQRELIYGERRRVLEGENMREQIQFFMDELITELVAQFAAGEAPAEWDLKELWTNLRGYYPVSITIDEVEAEHGGISGLTRDALTKEILGDIHTAYEELEEQINDNLIARIQLGEEPMRELERRVVIATVDRLWREHLYEVDYLKEGIGLRAMGQRDPLVEYKDEAARMFQSMMGRIREESVQQVFGFGRQFEALEQQAQAQAEAAASLQQATATGPAEPGSGDQTEAEPAAAPAKPAAQTEGANLAKVASVMG
gnl|extdb|pgaptmp_000529			MDIIINGRNTDISPTFRELAEGKLEKVTLFYPRAQRVDVVVTRQKNPRLADTAERIELTVYGKGPVIRAEAEAADRYAAIDLAVGKLYERLRRLRDRVKDHRRHRNEPKLEVELDAAHGDSAETGEDLSNAENFHLRSAGDLQVGEAREEQLGDSPVVVRQKVHEAAPMSVDEALYQMELVGHPFFLFVDEDTKQPCVVYHRSGWTYGVIRLNTRVDL
gnl|extdb|pgaptmp_000528			MSLLYPQSCAGCGRWDWRLCPDCQMLAGAEPNLGSLDNDWGVPTVPVWSLGRYGGPLRSIILSAKHERGQNLDGFLYQAGATLAVAAAEVLEPARQIWVVPAPSSWRRRWDRREIVPSVARGVGDSLAASLPGSRVRLAPALKLRVGRRSQTGRGSRARRSGREGAFSLVAEPPPGCAVVLVDDVLTTGATMREMWHEVGEPVQVGLVLARA
gnl|extdb|pgaptmp_000527			MKTKWRALVLVGALFLGGCATMPTSGDPQAFEVAAPNTEPVEQLGFGPVRNSTPERIISDFLRASAAGSTDDYLTAKKYLKDDLASSWNPRAGVLIYPTEQTPGLRLEMTGTGTATVYLETEVTATLDEEGVLSPGQATTVELSFDLVRSVDDQWRISRMDDGVILSKANFQAVYQAQQLYFLAPDQESLVPDPRWFPRRRLASHLVTGLLAGPSEQLAPAVYSALSGSLTLPTQGVEVDGQTVRVNMEGEVPGDRRTQENISRQLSATLFQMTNVTEVVTQINSVLLPATPDPFSASLELDAAVALSDGAIAVSAGEGWSPVVPAELVGPDAKSPARAPIADGAIAWIQDGISVWDGTEVHHVELATPTAPSVDRWNWTWTSSGEASGVVAINSRGESVELALPQGVSTQIRKVVVSPDGVHLLVLVDGADGLQAMNAVVVRDPLGAPQSIDQIDLVSLAGAGLVDGDWAGSTQMVFLAGHGEEREVRIVALGGFAQVLGAPSDTIRVSAGGQAGRILLETATGQYYSRSGGVWRSLEARVSAISYAG
gnl|extdb|pgaptmp_000526	K07654	mtrB; two-component system, OmpR family, sensor histidine kinase MtrB [EC:2.7.13.3]	MSALLQRGRRRLHRSLSIRVALTLTLMLLALMMVVGLIAVGQMRNELFHVRKDAILEDASVRFTQAQNTLDQSTAATTDQVQDLVSQLVAYTRDSAAGAGAVSVMLLRAPDSSNTFIVNELSNPQMRQTITPALRELVEGGQSAWQSIAIPEAVPGIVVGSLVDVPQAGPYELFIVYSLESEEQSVALVIRIFAVATLPLLLIIAVASFWLVYALLRPVRSTAEAAAKLADGDLESRVEVSGQDEMARLGTAFNNMAQSLQQQIDEYDTLSQLQQQFVSDVSHELRTPLTTISIAGEMIYEARGELSGAPQRSAELLFSEVGRMQEMLADLLEISRYDAQSTQLDIEMTDLYALTQKTMASVQELADHLGVYVTLSARPNSPLAEVDSKRIERVIRNLLVNAYEHAEGKPVQVEVEAAPGEITVRVVDHGVGMSEETIARVFDRFFRADPARARTTGGTGLGLAIAKEDIAAHRGRIVARGALGQGATFEFTVPRKFGFPLSSPGGAQ
gnl|extdb|pgaptmp_000525	K07670	mtrA; two-component system, OmpR family, response regulator MtrA	MTSRILVVDDDSALAEMISLVLAAEGYEVVECFDGAKAVEEFQRVDPELVLLDVMLPGKNGIEICEEIRRQSNVPIVMLTARSDTSDVVAGLGAGADDYVPKPFKPKELVARVKARLRSVEQPEDEQMTLGELNIDVSGHQVRRGNERINLTPLEFDLLVTLARAPWKVFTREELLESVWGYRHVADTRLVNVHVQRLRSKVERDPENPSLIVTVRGVGYRAGTGM

gnl|extdb|pgaptmp_000524			MRYCSKPGCPGHAVGTLTYDYQNSTAVLGPLATTAEPHSYDLCERHVTQLTVPKGWDVVRLEINYDEAAPSDDDLMALVEAVREAAQQPAPEVSGGFTSL<br>VKQRAHFEVVDGISEPPPPPESAPEPGPFQT
gnl|extdb|pgaptmp_000523			MMEVVSPAGSFRTPRRRKDRHGRRQNGVLVPAILPASRSRREKFDREILAILTRMKARFPEIGEIEFGVEDVPPSTPAPWEDFDVCLARSFPRDRTRGLANRI<br>VVYRRPVMQRCGPEGCYPLLRLLLAYRISELLTVDPEELLAIG
gnl|extdb|pgaptmp_000521			MHALVVTTGGPDLPEVLSALPVQTTPPDLLTVVTVGEVTLPELPPEVQVVSLKSADNFGQALRQVITSASQPDWYWLLHDDARPEPRALAELMRAASQGA<br>AIAAVGPKQVGWDAPQVLLELGIEATATGRRVPLGTEKEIDQGQYDDRVDVLAAGSAGLLVRADAWHQLHGFDPHLGPYGDGLEFGRRLRRAGYRVVVA<br>PKARVAHRRASLERGEFRRRREAQLYNWLLGTVALWAALLIPLLALWSVARALARLIMRQPRLAGAELGAYLDLLWGLPALIRGRRRLARVSVVPRRQLCPL<br>EASAAGLENRRRLARRVAHHRPKPIDQLDQISRRLLAEHRRGTRLAFLTALAIPTLLSLYYWYPLLGGFTGGSWAELPGSLWDQAWSPWVAAGSGAPGPVN<br>PVLPLLSIFWLNPPALTQALLVAALPLAAAGGWLVARLFTHSPAWRLGAALTWALQPLFLEAIGRGDLSLILVWLGLAPVLVGLWRATRPAVGLRVEGVVDT<br>MITSQYDPLTWGAVAALGLLAVGAGSPALIVPAAVLGYLLAATGTRRALSFWAVTLAWVPAALVGAPSLVTSLEAALTPTGGFARVGAQSVLTAWEWAGA<br>GVLLLAALASLLYTCWPGRGRPWLVRSAWLGAVASVWVLVGTDGEPGSVRATLFLSLLVAALGAAGPSTLPRRTRWVGAGASLATAAGLASFVGLLVVGPL<br>APGTPAGLSPHPGAPLVAREAAASERSARTLVLTQTSEQVEAQLWRDGTRGQEDLSAGLLAEAANPAREHLADAVGQLTARPSTEAAQALADHAVEIILLTP<br>TSPVGYGSLHDHLDATEGLERIGTTDLGTMWRVRPGGRLPALATLGPEGRPVPAVSVSLSEPTTLTLAETADPSWRAWLGSVELEPTGSDWRQAFAVPAGE
gnl|extdb|pgaptmp_000515	K12990	rfbF, rhlC; rhamnosyltransferase [EC:2.4.1.-]	MDSKLVAVAVTYNPDLNAFRSLLAALRPQVDELVVVDNGSVEAAAVGREVAEAGANWLPLPENLGIAAAQNRGIDWARTAGATHVLLSDQDSLPEPDMV<br>ERLWECLTCCDESLGAVGPVPLDGRGDEAEALVYSFTTWGPKRRTVPGPGQVLDVPFVLASGCLIPLAVLDRVGPMNESLFIDHVDLAWCLRAIEDGYRIKV<br>CGDAILHHSLGDEVAHIPGRKRPVHLHSAPRNYYMMRNTLFLLRASFLPRKWKLGYLLWMTKYTGYYLLASPGRLPIFLRALRDGLTGRGGPLS
gnl|extdb|pgaptmp_000507			MRVAVIGMGKIGLPLAVQFADAGQDVVGVDVNPQTVDLINQAVEPFPGEAFLQEKLSELVPAGRLRATTDYAEAIPGADAIVIVVPLFVNDATWAPDFDW<br>MDAATRSLAEHLTPGTLVSYETTLPVGTTRGRWKPMIEEISGLVEGKDFHLVFSPERVLTGRVFADLRKYPKLVGGLSAEGTERGIEFYQQVLSFDERPDLPRE<br>NGVWDMGSAEAAEMAKLAETTYRDVNIGLANQFAVYADQEGIDIEKVIDACNSQPYSHIHRPGIAVGGHCIPVYPRLYLSTDPDASVVRTARQFNAGMPK<br>YVVSRVEEVLGSLANQDVAVLGASYRGGVKETAFSGVFETVKQLEERGANVKVQDPMYSDAEIEAFGWAPYHVGEPVDAVIIQADHAQYRELTPLDFPAVK<br>LLFDGRRITDPAKWVGTPRLVIGHAS
gnl|extdb|pgaptmp_000506	K01791	wecB; UDP-N-acetylglucosamine 2-epimerase (non-hydrolysing) [EC:5.1.3.14]	MRILSVVGARPQFVKLAPIDREFRRRGIDHVIVHTGQHYDPLLSDVFFQDLGISAPAVHLGVGSGSHGKQTGAMMAALDDVFLEYQPDWVLVYGDTNSTL<br>AAAICAAKLHLPVAHLEAGLRSFNRQMPEEHNRVLTDHCADLLLAPTQVAAEHLAREGVDPARVVVTGDVMTDVLYQVMEQVRDNPSPFPDLGPGEYSV<br>ATIHRAENTDDARRLEAIIDSLGAVDHPVVLLAHPRLLAKCEHFGVPLKRGHLQVHDPVPYPELVSAVLNSRGVITDSGGLQKEAFLMRVPCTTVRPETEWVE<br>TVELGWNVLVQPGGDLTLAASRPRPAPTDAAPYGTGQAAQIVADALQTMGSRE
gnl|extdb|pgaptmp_000500	K24846	pglK; ATP-binding cassette, subfamily B, bacterial PglK [EC:7.5.2.-]	MRNAWRQTQELMPYLPPTARRYIRVYIILSCLLTLLDVAALMLLALSLSAMMQGVPVELPVIGSVPPDKYIWLLLVVSLLVILKSILSLLQQWAATRRFAEFELS<br>LGVKLFDAYIGAPWVERLSRTTSQLVRMADVGVAAVVSGLLLPLIQLPATLASSVLILGTLLFVQPMTAVISIVYLGGMAFLMSVVLTKRAVEAGRVNRDYSFR<br>VASLMTDMVGALKEITLRNKFDEVAGAVKANRTHAARARANIQFLASVPKFIMDTALIGGFLLVGVISYLVEGSLDEAISAIVLFAVAGMRLVPALTTLQGTA<br>NTINANRAQVDAVLFDMQEAEEYRAAVEHVGKVPLAHEPRELVLSGVTFQYPTGERPAVADVSLAIKMGTSVGFVGSSGSGKSTLVDIILGLLTPQAGQVLV<br>DGQELTEVLADWRSRVGYVPQEVSLFDGTISQNVALSWSGDIDQDRVIDCLKRAQLWEAVQARPGGLNAKVGERGMAFSGGQRQRLGIARALYSNPYILI<br>LDEATSALDTKTEAEVAQAIANLRGDVTLISIAHRLSTVKDADELFFMEGGQVLAHGTFHEVVNQVPMFREQAQLAGLVNEE
gnl|extdb|pgaptmp_000493			MANIRVGVLGIGSMGRHHVRNARALEGFDLVAVADPGGDKFGVAGDLDVLGSVDELIAAGIEAAIVAVPTVFHEDAALKLAEAGIHTLVEKPLAGDLAAGQ<br>RMTEAFEAAGLVGAVGYVERCNPALLEMRKRIADGQLGEVYQVSTRRQSPFPARISDVGVVKDLATHDVDLAAWIAGAPYELVFAQVSHRSGRDHEDMLT<br>ASGRFTNGILVNHLVNWLTPYKDRATIVVGEHGALVADTAMGDLTFFENGEFALEWDQIAAFRGVSEGQVIRYALKKREPLAVEQGNFRDAINGLSSNIVT<br>MREGLDDMRVVEAMLESAATGESVKL

gnl|extdb|pgaptmp_000492			MTTEELAFIPPAKPIIGEEEVEAVSAVLRSGMVAQGPQVAAFEQEFSEQVVDGVHSVAVNSGTSALHLGLLAAGVGPGDEVIVPSFTFAATANSVAITGATPVFADIEPTYFCLDPKAAEAAITERTKAIMPVHLYGHPANMDAFEDLAEKHGLALFEDAAQAHGASLDGQKVGTFGTFAGFSFYPTKNMTSGEGGMVTSASAEVARRVQVLRNQGMAKQYANEMVGLNNRMTDINGAIGRTQLKHLDEWTQKRQANAAFLDANLEGVVVPAVAEGAVHVYHQYTIRVDAADRARIVEQLRDQYQVGSGVYYPIPNHRLVSLAHFAPGLDLPETEKAAAEVISLPVHPSLSQADLERIVTAVNAVVRAGA
gnl|extdb|pgaptmp_000491	K13018	wbpD, wlbB; UDP-2-acetamido-3-amino-2,3-dideoxy-glucuronate N-acetyltransferase [EC:2.3.1.201]	MPRIVDSADVSPQASIGEGTSVWHLAQVREDAQIGENCIIGRGAYIGTGVHLGRNCKVQNYALVYEPAYLADGVFIGPAVVLTNDHNPRAVNPDGSVKSASDWEPVGVTIGEGAAIGARAVCVAPVKIGAWATVAAGAVVTKDVPDYALMVGVPARRIGWVGKAGLKLEPEANRPGYYRCPEAGTMYREISETELEEVETDDN
gnl|extdb|pgaptmp_000490	K22907	ppgS; polyprenyl-phospho-N-acetylgalactosaminyl synthase	MCADFAPITSTWLVIPLYNEGPVIGDVVREARKTFPNIVCVDDGSSDSSAEIAADAGAYVVQHPINLGQGAALQTGIEYVLHYTEAKYLVTFDADGQHSTDDAAAMVARAEADDLSVIYGSRFLEGQVDIGWAKRLVLRTAATVTRWRTGLKLTDAHNGLRLLRRDAAASVSLQQDRMAHASEIIGQLAKTDFKWVEMPVHIRYTDYSKSKGQSLLNSVNILVELVLG
gnl|extdb|pgaptmp_000489	K09153	small membrane protein	MSYWLIKTILIIGLVVVTYFMMRPIKSANHLALRRLGVMLIVVAAGFAVVFPDVINRLAWLIGVTSGVNLLVYVLFLVVFTQMATGYRRDSANDRKLTLLARALALESAPKPPTGLSDPHGSTHRSNDAPSGAPDESDRAH
gnl|extdb|pgaptmp_000488			MSKAKSRANTDRLPIAHASPNPYRFPWVRSVLAAVLAVALFGGVAGALMINNLDHQIKDSVISTANRGSDNSQSATVVNEELPPDAFEGRPVNILVSGIDSRYNENGEIGAGTIDLDPTIRSDTTMILHLSADRQHATILSIPRDMMVDIPSCQTADGSYTYAHYGMFNSAFADGAGLDDIAGGIACTQATVEDFTGITIDGFVVIDFTGFSRLVDTLGGVDICLDEAMYDELAGLDVPAGCQTLHGQQALAFARARKDLADGSDLRRIDRQQWLIGQMVSQILDSNMFTNLPSLYKFVQEGLSTSKFSPSLNSWRTDAALLNSIRNTPRENIRFVTTPYLPDPEDENRVLPDYYQASAIFQALIDDQPLPAGTLFRNLQNQTFIVGDAGEAIMTDDLGNPYELDENGAPI
gnl|extdb|pgaptmp_000486	K01784	galE, GALE; UDP-glucose 4-epimerase [EC:5.1.3.2]	MTILVTGGAGYIGAHVVRLLQERGEKVVVVDDLSFGNPKRIGDAKLVKTDISTDEAWRLLTDTMVDDDVEAVIHFAARKQVGESVQRPSWYFRQNIGGMANLTRAMNDAGVEKMIFSSSAAVYGMPPVDVVPEDIDLKPINPYGETKLIGEQLLADCEVAWDLQWVALRYFNVAGTGWVDLQDPAVLNLVPMVLDRLAKGQAPKIFGDDYPTSDGTCIRDYIHVMDLADAHIKALDQLNSGKLEHHQFNVGTGKGTSVREIIEGLRDISGMDFPIEVAPRRAGDPPKLIGDASRIQEDFGWSAQYGVKEILESAWEAWQQGPRKIEWSN
gnl|extdb|pgaptmp_000484			MRARTTRLIVTVVTIVALIMGIPGSVVAGMLVWNSAQSALDTRVQTLGRSIDRRLDDDLWVTQPLVAALTNPVSGEQDAFTEIRVGGEYKVISGTKRSGPLLEAKVRSSNGATVIMQISAYPVATSVARAAMFFAGGALLSILLGWYLAYRMSRRLSAPLIYLAAQAEQIGSGQVRARLKPSGIEEIDLVQEELVRTGERMAGRLAAERQRSADASHQLRTPITALSMRLEEIEMITTEDEVREEARACLDQVERLTSVVTELLDERRRNQSSTEALHILEVFNTQREEWEDQFAAAGRELVFLDEAERPILAEAGKISQVLATLIENSLRYGAGKTVVRARKAASSRGVLIEVSDEGEGIEDDMVDEIFDMGVSGHGSSGIGLALAKDLSQAMGGRLELTQTHPPVFTLSLAAIPSNFDPDLVMPQGPLLSVGRRSRRL
gnl|extdb|pgaptmp_000483			MTTVLLVEDDPAISEPLARAFGREGYEVRAHGTARGALNEVGSADLIVLDLGLPDMDGLDVAREIRSRGLDTPILILTARTDEVDMVIGLDAGADDYVTKPFRLAELLARVRALLRRSGSEAADSEIRVQDVVVDLAGHRVFVGGEEVSLSAKEFDLLRVLVSSAGTMVSREDLMREVWGSDPQGSTKALDTHVSWLRRKLGDNTADPRYISTVRGMGFRFEK
gnl|extdb|pgaptmp_001874	K06980	ygfZ; tRNA-modifying protein YgfZ	MSDYLAGAVFAEGQVAPIHFGDPVTEQFEYEAGRALHPRGPVGLVTVTGADRLTWLTTLSSQDLAPLGEDGSSEMLLLDPNGRIQFAMGVVSQGDTVWLLTDPEPAPELAAFLESMKFMSRVEIRDRSADYQVFETAGGASPAGAEATWVDPWPGPSLGGARYFQGSHPGEAFQHRLMIVPVDRAAQFVADSGLGLAGSLAAEATRVAAWRPGWTSEVDDRTMPAELDWLRTAVHVSKGCYCGQESVARILNLGKPPRRLTFLQLDGSLSSIPAPGDPVELNGRPVGVITSVARHAEMGPIALALLKRSVDPAAPLTIGPIAAAQELIVPVEGKSDHSPSERPGAGLRRLDHGGRDIRTTGPGTVR
gnl|extdb|pgaptmp_001875			MFEIPENLPLSLAPLAWVLGRWQGWGTLAGDDDEPDAIILQDIQAEVVGQQMRVVTSLYHGKVEGEIDWQMNAAAGLDLIEPGELFREETSYWRLATPLAVLPPEGEEPRELQVTSADTQGLATLWVGVSMGPRIRLTSDVIARDASAPQMTTVHRMFGLVGGELFWTSETSLDQGEPQVELSGRLRRASQEAEEA

gnl|extdb|pgaptmp_001876			MSFLPQNVLDDIHARAPQYDADNTFPTEDFDQLRECGYLGAYVPTKFGGAGLTLEQIAAEQTRLAQAAPATALAINMHQIIVGVGRHMVRSGLSAGEQILRDAAAGELFAFGISEPGNDLVLFGSITEARPDGEGGYSFHGVKIFTSLAPAWTRLMTFGKDATDPEAPKSVFAVLHRDRGGFTMKNDWDVLGMRATQSNSTILEGAAASADEILTVTDPGPSANPVVMGIFANFEILLAATYQGVGQRAVQVAVEHVKKRHSVKNQTTYANDPDIRWRIASAALTMEAVGPQIRELARAFEDQVDLGNWWMPRLSAVKNAASEATLRAVEESVRACGGASYYSKHELSRLYRDALAGLFQPSDQESLHGAWATMLLGPIEH
gnl|extdb|pgaptmp_001878	K01939	purA, ADSS; adenylosuccinate synthase [EC:6.3.4.4]	MPGIIVVGTQWGDEGKGKATDQLGSNVEVVVKFNGGNNAGHTVVVGGEKYALHLLPAGILTPHATPVIGNGVVVDLEALFSEIDLMESRGVDTSRLLVSANAHVIAPYNRLLDQVTEKSLGNKQIGTTGRGIGPTYADKMNRIGVRIQDLLDEEVLRQRVRGALYEKNQLLEVVYRQSPVDPDQVADELLAYADRLRPMVADTSLFVNRALAEGKTVLFEAGQAAMLDIDHGTYPYVTSSNATAGGALTGAGVGPTQISRVVGVTKAYTTRVGEGPFPTELSGAEGDHLRELGGEYGVTTGRPRRVGWFDPLVVRYANRVNGLTDVVLTKLDVLDSYDEIAVCVAYEVDGQTLTEMPMDQGSFARAQPVYQLLPGWKSDTTSVRKFEDLPAAAQDYVLFLEQQIGCRISSIGVGQGREATIIRHELG
gnl|extdb|pgaptmp_001879	K13821	putA; RHH-type transcriptional regulator, proline utilization regulon repressor / proline dehydrogenase / delta 1-pyrroline-5-carboxylate dehydrogenase dehydrogenase [EC:1.5.5.2 1.2.1.88]	MSKRYPTDSYYDLHLAADRALQTANRWTQLSSHYPSGKAAELLASVLEDPNGLNPTVLPVDGVIRPLDMRVAAHNLRQLTRYSPRFLPPWLRIPAQVGGVAGFLAPSVVVPAARRVFSELVGDLVVDVTDSKLGPAIEKLRADGSRLNINLLGEAVLGDAEADRRLAANKALLARDDIDYVSMKVSAVTGPRSHWDVESIVDQAVERLLPLYQLAASSPTPKFINLDMEEYKDLDLTIQVFERILEREELAHLEAGIVIQAYLPDALPAYQGLVAWAQRRIEAGGAPIKVRLVKGANLAMERVEAEVHGWPLALWESKQATDANYLRILNWALTPERMRAVRLGVAGHNLFTLALAWELADLRGVRDRIDVEMLAGMAEAQAQAVRDEVGSLLLYVPVVDPREFDVAIAYLVRRLEENSASDNFMSHVFEMGEDQKVFDLERDRFRRAVTQLVAEGEKRCQANRTQNRQTETVRQLEAEMRGPGGQWKFENVPDTDPALAANREWARQIVGRVPESTLGEDTVASHTVANRTELNQRMKKALTAQRKWAKKSAAERSDLLHRVGIELSRRRADLLEVAAAELGKGIDQTDPEISEAIDFAHYYAQQLLELDRLPGAKFVPAALTVVTPPWNFPLSIPLGGTIAALAAGGAVMLKPASAAKRCGALLAECLWAAGLDRNLAQLVVPGNREVGRQLVTDERVERVILTGSSETAEMFLSWRRDLTLLAETSGKNSIIVTPSADLDLAAKDVVASAFGHAGQKCSAASLVILVGSAGRSKRFHDQLLDAARSLHVAWPTDLAEEMGPLSELPGEKLTRGLTKLEPGQSWALKPEPLDDSGRLWSPGIRGGVQPGSEFQQVEYFGPVLGVIRVATLAEAIEVQNSTAFGLTAGIQSLSADEVNYWLDRVQAGNVYINRGITGAIVQRQPFGGWKLSSVGPGAKAGGPNYLFGLGRFEPVDEPKGTVGGEGYPAVPERELAVEKRQLLEAVEVADRLLTPDQAERVQRAAYNLERACTTHFDRLNDPTGLKYERNVLRYLPARAVLRAESAASDADILLAAMAAVAVGEFRPSPEDPDFLVRHCAGQTEAEPLGYQGAQLVLSTTRALPDPIQDWAHRYGFACLVESPEEFAQRLDGAEVHHDGRVRLLGSERATLMGQLSAPIDYAIWDGPVTTAGRVEILPFVHEQAVSLTTHRYGTRSSLADQVLNS
gnl|extdb|pgaptmp_001880	K03218	rlmB; 23S rRNA (guanosine2251-2'-O)-methyltransferase [EC:2.1.1.185]	MSKGNQGRRGAQRKAEKGPVIGSGGKHKRALSGKGPTPKAEDRTYHAAHKRKLEKEREKEVAQARAQAQNRTRVRLQPGTELVVGRNAVIEVAEAGIQIHRIFVSSDPGDGRMRDVTTALANSGAPFIEVTRRDLDIASGGATHQGIGVEVAEYEYWDLDELLVRALEKPSGNPGLLVALDHVTDPHNLGAVLRSGAAFGVDGVVIPQRRSAGVSVTTWKVSAGAAAKVPTARVSNLVQALKRCQDAGFFVVGLEGAAGPTIRGLELADVPLVLVTGAEGKGLSRLVAESCDLLVSIPMLGMESLNAAVASGIALYEIAEKRREMAEK
gnl|extdb|pgaptmp_001882	K01770	ispF; 2-C-methyl-D-erythritol 2,4-cyclodiphosphate synthase [EC:4.6.1.12]	MSATFRVGQGVDVHAFSGRKGTLHLACLDWPEELELEGHSDGDVAAHAACDALLAAANLGDLGSVFGTDDPRWAGAAGATLLGEVLSRLTEAGWAVENVSIQVVGNRPRLAARKDEAAAALSAALGGADVSLGATTTDHLGFLGRTEGLAALATALVSR
gnl|extdb|pgaptmp_001883	K06902	UMF1; MFS transporter, UMF1 family	MSSVAESGASPKIPAHKVWSWAAWDWGSAAFNAVATSFVFSVYLTTDGLFASSATANQYLSIGMTVAGLVVALLAPIAGRRADRAGRGLLSVGAFTFLVVACLAAMYFVAPNSGPSPQAMLILGVALLGLGNIFFEFASVNYNAMLNYVSTPADRGQVSGLGWGSGYLGGIVLLLILYFGFINPEVGLFGVTGENGLDVRVAMLFAALWMALSALPVLLNPPPGYAKAKGHGQAGSLAENYRALFRLVVSLAKDSPDTLKFLIASAVFRDGLAGVFTFGGVIAGAVFGFSASQVIIFAIAANVVAGVSTVLVGRLDDLWGPKRVIVFSLVAMVFAATGVFIFHDLGPMVFWILGLLLTIFVGPAQSASRSMLARMIPAGREGEVFGLYATTGRAVSFLAPLLYGVAISLGNRITGGDGTPFGILGVVVVLLVGLLLILPVREGAKISVAHLES
gnl|extdb|pgaptmp_001884	K00991	ispD; 2-C-methyl-D-erythritol 4-phosphate cytidylyltransferase [EC:2.7.7.60]	MASPSAVVVLTAAGTGTRLGADVPKALVQLGEHSLLAWSLRGLAQTEAVAAVAITAPADALDQFWQEIGRYTFPFPITVVPGGNARQESVACGLDALSQLTAQEGLTPSWTTPTLVHDAARALTPPEMIGRLIRLVGDGTPAVIPGLPVTDTIKEVSGEDQELSPVRATPPRARLRSIQTPQAFFWGVLLAAHERARALADDEATAATDDAALVEAEGGQVWVTPGHPHALKITTKKDLETAERMILGLDF

gnl|extdb|pgaptmp_001885	K07736	carD; CarD family transcriptional regulator, regulator of rRNA transcription	MTFKVGETVVYPHHGAATIEEIITRELNGEPTTYLVLRIHQPEQQDKQKELIIQVPADKVDEVGVRDVVDDKGVEEVLDVLRAKDVEEPSNWSRRYKANQEK<br>ITSGDIVRVSEVVRDLSRRDADKGLSTGEKRMLTTARQILISELALARDIDEEKAGERLDEVLSEAGPDEAAE
gnl|extdb|pgaptmp_001886	K01834	PGAM, gpmA; 2,3-bisphosphoglycerate-dependent phosphoglycerate mutase [EC:5.4.2.11]	MTYTLVLLRHGESEWNAKNLFTGWVDVPLSAKGEEEAKHGGVLLKEQGILPDLLFTSLLRRAITTANLALNEADRHWIPVERNWRLNERHYGALQGLDKKA<br>TRDKYGDEQFMLWRRSYDTPPPAIEPGSEFAQDGDPRYAGEPIPHTECLKDVLERLLPYWNGTIVPALQTGKTVMVAAHGNSLRAIVKHLDEISDDDIAGV<br>NIPTGIPLLYELDEQTLKPVTKGGRYLDPDAQAKIDAVANQGK
gnl|extdb|pgaptmp_001892			MPKIAELDVYPLCLGTNTFGWTSTRAEAFAVLDEYTAAGGNFLDTADAYVRWAGQGGESEEIIGQWLRESGRRAEVVLATKVGKLPPHDGLSAEAVSAAV<br>DASLQRLGVEQIDLLYAHYYDPEHPTDPAIFDRLIRDGKIRTYGLSNHSPAQVREVLEAADRAGVARPVALQPHYNLLLRGEYEGDLQDLTVAEDLAVMPYYS<br>LAAGLLTGKYDLSAPLAGARAQMAGSYLNERTPAIIDAVSGVAQEHNVEPAAVAIAWLLSQRGITAPIASARTPEQLFPLFEGVSIVLSEEELAQLEQASA
gnl|extdb|pgaptmp_001893			MTSYVAALQAVTETDTSSRRRAEYSSDASNYRVVPSAVVFPRSTEEVLAIHRIAREYEVPITSRGAGTSCAGNAVGGGIIIDYSRHLNRIISIDPEARTARVQPGV<br>VMTDLQAAARPYGLWFGPDPSTKNRATFGGMIGNNACGPHALAYGRTADNVVSLDVVDGQGHRFEAGTDLDVVPGLAELVGRNLAPIRRELGRFTRQV<br>SGYSLEHLLPEKGSSLARALVGTEGTCVSVLEATVQLHPLPPAPLLVVLGYPNMIEAARAVPTLLPLRPQAIEGMDSRLVEVVRAAKGSVPQLPGGQGWLFCE<br>VAGETAAEAEQRARELIERAGAEDSLVVTDPGQAAALWSIRADGVGLAGRTPAGVPTWPGWEDAAVPPAALADYLTEFEQLMERHGLTGLPYGHFGDGC<br>IHIRVDWPLSDPDDVPAFGTFLEEAATLVAKFGGSTSGEHGDGRARSSLLRHMYSDEVLALFAQFKGLFDPQDLLNPGVVVNPEPPTANLRRPQAHPLATN<br>GFAFREDGGDFTKAVHRCVGVGKCRSTSGGFMCPSYRATRDEKDVTRARARMLQEVANGTMVAGWDAPELAESLDYCLSCKACGRDCPAGVDIARYKSE<br>ATYQRYRGKLRPMNHYALGWLPRWARLVSAFPPVAALANSALAITPLRRAVFAVAGIDQRRQMTGFATTRFSRWFKRRRQPTVGRDVLLWADSFSENLD<br>PSGAQAMVELLQGAGYQVRIPSQQACCGLTWISTGQLDGAKKRLSQLLDVLSPAAQQGVPIVGIEPSCTAVLRDDLLDLLPDDPRAQAVAASVRTLAELLTD<br>PVLGPGPDWEPPQSLAGVRVIAQPHCHQHAVMGFEADAALLQRLGAEVTQLEGCCGLAGNFGMERGHYEVSVAVAQNALLPALQDPDAAGAVFLADG
gnl|extdb|pgaptmp_000395	K08483	ptsI; phosphoenolpyruvate-protein phosphotransferase (PTS system enzyme I) [EC:2.7.3.9]	MSSEAINQEQVVVHGTPVVAGIAYAPAAWTQRPPLPPASAPDLPEDEREAEIEAFDRAAAAVAERLLQRAEAAEEHAAEVLTMTAGLAKDKGWRREIVKAI<br>KTGVPAIQATMAATEKFVLLFEKAGGLMAERTTDLRDVRDRVIARLQGNPEPGIPVRDHPIVLLGDDLSPADTAGLDRNDYVAIVTQGGGPTSHTSIIARQLG<br>IPCIVAARELELIPEGAEVLVDASVGTVTMGVPAAEAERLVAEDSARLELVRAWRGPGQTQDGVPVQLLANVQDGPTARTAADGQAEGIGLFRTELLFLDTA<br>TEPSVAEQAGSYGEVFSAFPEGKVVVRTLDAGSDKPVPFATLAEEANPALGVRGIRVTGHNPELLTHQLDAIAEASKKNPGTDVWVMAPMISTVPEAKWFA<br>GLCRERGLKAGIMIEVPAAVILIDRFIEVVDFVSIGTNDLTQYTMAADRMSPHLAEYTDPWQPAVLELIQWTADSGRQHDVPAGVCGEAAADPLLACVLIG<br>MGITSLSMASSAVPAVGAQLSQVTFAQCQAAAEAVREAEDSGQARALARQALGLA
gnl|extdb|pgaptmp_001910			MRKRTAITVGTVLALALSACTPVQQVQSDSASLAGEALVEQAPAGFEQYYGQEIDFQPCDADQVTLPRMSPPKALNRYRCATVTAPMNWDDPDSAPIELA<br>MAIYGTNDNQPNLFFNLGGPGGDAVQSLSSFVELMVPAPVVDNYQIVAVDPRGVGASSPVSCWDDEGRDQFLADSDDPRDLPLDELVATAHQETADLYA<br>QCLERTGELLNYVDTDSAARDFDMIRAALGIEQLDYLGYSYGTQLGATYADLFPARVGRFVLDSAVDPALGINEVASLQAGGMEESLAHWIEVCQQDDDCP<br>VTGGVAGGQEQLAEFLDSLKADPLPTDSPDRPLTAALGRTGIIGSLYSPESYPLLKVALQLAFQGDGSMLLLLADFYNGREDDGSYNNSQDAFLAVNGLDYAP<br>EGTPEEWQAEAERLAEDYPVLGSSFGYASAGMDAWTATPRVRSGPVRAEGSAPILVIGTTHDPATPYVMAEGLVEQLANAVLLTYDGWGHGAYQQGGSE
gnl|extdb|pgaptmp_001911	K02341	holB; DNA polymerase III subunit delta' [EC:2.7.7.7]	MSVWDRLIGQAGAVEQLRAAAAAGRRALAGDERAKVAHSWLFTGPPGSGRSVAARCLAAALQCTGAEPGCGECSGCRSVLAGSHPDVNELTTEAMTYK<br>VEEVRGWLEVAYSRPSLGRWRVLIVEDADRMTPQTSNVILKSLEEPPAQTLWLLCAPSPDDLLITVRSRCRQLRLTTPPVDSLTKLLMQEAGVSQDQAHLAA<br>QISQSHVGYARALARDPQLRQAQVEALMTALRPQSVGEAVVAAQQLLDLAKKNSVNRLEERNAEELATFKQNLGLQPGERVPRPVQAQIRQLEEDQKRRA<br>KRSLADELDRILVDLLGFFRDVTVVQLGSPVPPINPDLMDQVSWWAERVDARGVVDRTEAINLARERLQTNVATTLMLEALLISLVRPDLAN

gnl|extdb|pgaptmp_001912	K00943	tmk, DTYMK; dTMP kinase [EC:2.7.4.9]	MDQLVSRAAQRLRELPRGMFIALEGGDGSGKTTQINLLRQRLEAHGANVLTTFEPGATPLGHQLRELVMHGPEDVDPRTEALLYAADRAYHVATMIRPALAAGTTVITDRYLDSSVAYQGIGRGLGERAIRDLSLWATDGLLPDAVIVLNIDPEVGLSRRGEEKDRLERAGDRFHEQVARHYQEAARLEPGRYRLVDANGSVEETFTGVVTALLEVLGGDR
gnl|extdb|pgaptmp_001913	K03168	topA; DNA topoisomerase I [EC:5.6.2.1]	MPGTKLVIVESPAKAQTIKGYLGDGYVVEASVGHVRDLPTPSALPPSMKKGPYSKFAVNVDDGFKAYYQVNPDKKKKVAELRKALKEADELYLATDEDREGEAIAWHLLEVLKPKVPVKRMVFHEITKEAIQRALENTREIDRSLVDAQETRRILDRLYGYEVSPVLWRKVAPSLSAGRVQSVATRLVVDREIERMRHVSASYWSVDTEVQTAEGSFGAKVISVGGRPVATGSDFAENGQLKEKAQQADAVALDEQRAEALAQALAGAEAAILSVTQKPYRRRPAAPFTTSTLQQEASRKLKWNASTTMRVAQSLYESGHITYMRTDSTALSGQALKAAREQVTKQFGPELLPDEPRFYGKVAKGAQEAHEAIRPSGEQFRHPQSLGKDVSAQQLALYDLIWKRTLASQMIDAQGYTATIKVSADAAGEEVISSASGTVITQPGFRLLYQESRDQGRYDKEKGGERELPAVAEGESVSVASATADGHQTQPPGRYTEATLVKIMEDLGIGRPSTYAATIQTISDRGYVTHRGQYLVPTWLAFSVTRLLVENLADLVDYDFTAEMEQGLDRIAAGEEQGPNWLKGFYFGNQSAKSEDFAARGLRDTVENLGDIDARAVNSIEVAEGITLRIGRYGPYLETADGKRASVPPEIAPDEMNEQVAHELLEEAAADGRELGEDPDSGHLIIAKTGRYGPYVTEVLPEDDKGKPRTASLFKSMDLKTVTLEEALKLLSLPREVGVDPADGEMITAQNGRYGPYLKKGSDSRTLASEDQLFDLTLEQAIEIYSQPKQRGRGTAKPPLREFGTDPISGKKVVVKDGRFGPYITDGTTNVTVSRAETVEGLTEERAFTLLADKRAKGPAPKRKAPAKKKTTRTAKK
gnl|extdb|pgaptmp_001914			MNPDLVARLRADLAGADWTVDTVDQLLGPVAGAALAREQRVPALVRLRSVHTPPAQLTKAFILGERVDLTEALPTLGLAGAQELGLIDQDGSPLMDLRPHAAELPGGNYHWWIASDRCEMQTGRPLQPNHVLGIGPATLSLLRMTVREPVEVALDLGTGCGIQALYLATHAHRVVATEISARACAYTRFNAALNQVQLDVRQGSLFEPVAGEKFDLITSNPPFVITPPSLRTEGLLEYRDGGMDRDDLVAQVISTGPNLLRPGGVMQMLANWEVPHRGSWEDPIRTWLTRSGVGLDAWVVLRDRLDAAQYSELWLRDAGGNLQGRQRWEADYEQWIADFAQAGVDEVAMGFLALRRRSRDGVELAAELVEEGTFPDGKTVLAALDHVQLAPNWEDLAPVRAEDVREERHYQPGEADPQVIRLTQGAGMGRAIRVGSAMAALAGAADGELTAGQIVSAVAMLTDRERDQVAAEVALELPALLRAGMMAWAGSEQ
gnl|extdb|pgaptmp_000569			MGEIRIKRVYQPAAGEDGYRVLVDRIWPRGESKEKAHLDLWLKEVAPSTELRKWFGHDPEKMAEFTRRYTAELENNPEAVAQLRELVAQHPVVTLVYSARDETDNQAVVLKQFLERQS
gnl|extdb|pgaptmp_001578	K09118	uncharacterized protein	MPKSNPLKNPLVITLLILALIGLVIYAMAHTWTEVLWFQQVGADRVIWTRWVAIAVIALVSFLVLFASVALTVQWAYRKRPLMGARSTMRVYQQALEPMRNLVFWGVPALIALTMAGTYASSWQQILMWLHRQPFGEVDPQFGIDISFYVFTVPVLQLLLSLLMNAAVLSLVGVLIVNYVYGGLTFSPKLRATRPARKQIGLIAAVISLLFGLRYWLGIYSMLYQNGGPNAIVDGALYTDISATLPAHLILAVVSVLVAVMFIVAAFRGTWRLPVTGVAVTIVSALVIGMAYPALIQQFRVTPNARQMEQPYIQRNIDATLKAYGLDEVEYQTYAAKTDASPGQLREDSESTSQIRLLDPAVVSPTFRQLQQSRPYYTFDEQLSVDRYTIDGERRDTVIAVRDLNLNGLQEEQQTWVNLHTVYTHGYGVAAAYGNAVRPDGTPSFWEQSIPSVGEIGDYEERVYFSPKAPVYSIVGAPEGTPPQELDYPDDNAPSGQVQTTFTGDGGPSVGNFFNKLLFAVKFGSTDIFFSSQTNSESQILFDRDPIQRVKKVAPFLTLEQKAYPAVVDMDDDPSTTQRLVWIIDGYTTANSYPYAEHQSLTEATTDSRTLAGPAAAQFETINYMRNSVKAVVDAYDGSVKLFSWDDQDPLLGAWQGVYPGLIQDKSEISGDLMSHLRYPEDLFKVQRSLLATYHVTEAAEFYTGGDRWRLSEDPTSASTDAAGQTQQKLQAPYYLTMQMPTQESAEFSLTSVYVPAGGGEARRAAMAGFLAVDSETGNEPGKVRDGYGKLRLIALPSSTTVPGPGQVQNAFNSDQTIASELNLLNQQGSKVISGNLLTLPVGGGLLYVQPVYVQSTGSTSYPVLRYVLTAFGDQIGFARTLQESLDQTFGGDSEATVAGSEGEPEAPDESTDQTLQQQLSKALASARDALKAGQDAMSNNDWTAYGKAQNDLQKALEKAVQIQSQLDAEAAALLGEPADPADGGAPPGDVGPETELGSDAEQG
gnl|extdb|pgaptmp_001614	K03424	tatD; TatD DNase family protein [EC:3.1.21.-]	MAKRTRQWPPLGEALPVPVIDNHTHLPVHEGEIPKVDGVKLSLSEQLERAEQAGVRGLISSGCELPALTPTVEIARAHPQVRAALAIHPNEAALHAGNLEPSPDGYQHEQQEHHVPLIVALERVAALLGEPEVVAVGETGLDYFRTAEPGREAQKESFRAHLEMARARDLPVQIHDREAHRDTLELLGDPAWRQVTAVFHCFSGDAQMARVLAERGWYASFAGPITYPANGELREAFGAMPRELVLVETDAPYLAPAPYRGCPNASYVMAHTVRYLADLWEVSPAEAAKQLNENTRRVYGD
gnl|extdb|pgaptmp_000646	K01867	WARS, trpS; tryptophanyl-tRNA synthetase [EC:6.1.1.2]	MTTNNSNEATLKRALERSAEIERAIDEDPSQFRVLTGDRPTGRLHLGHYFGTLQGRVALQQRGVETWILVADYQVITDRDAVGPIRERVIGLLADYLAAGLDPDQTTIFAHSQIPELNQLMLPFLSLVTESELHRNPTVKSELEASGGRAMSGLLLTYPVHQAADILFCRANLVPVGQDQLPHLEQARVIASRFDRRYGRANPEQPVFRRPEALLSKAPYILGTDGNKMSKSRGNTIELAMTADETAKILKKAKTDADRHITYDPEGRPEVSNLLLMASMATGQEPEELAAQIGDGGGGALKALVTDAINELLAPIRARRAELMADEDYLLGVLRRGNERASEVAVQTLTEVRQAMGMVY
gnl|extdb|pgaptmp_000636			MRHSEVGPGGYDYQVQRVRGSAKTYRCPGCNQVIAAQVDHVVAWREESLLGWDSGVETRRHWHESCWARGLAIDGKP

<table>
<tr><td>gnl|extdb|pgaptm
p_000452</td><td>K12503</td><td>E2.5.1.68; short-chain Z-isoprenyl diphosphate synthase [EC:2.5.1.68]</td><td>MAVNLLYGVYERRLQRALRYAPIPRHIAVILDGNRRWARAVGSAPSVGHKAGADRIGEFLEWSEELGVEVVTLWMLSTDNLMRDEDELQQLLEIIIGSVQSL
ARRQRWRLSLVGDLDLLPADAAERLRQAVSTTEFLDTMQVNIAVSYGGRRELVDAVRELLVEAEADGKTLGQVAGELSDEDITAHLYTRGQPDPDLVIRTSG
EQRLSGFMMWQAAHSEFYFCEAYWPDFRRVDFLRALRAYSQRERRRGR</td></tr>
<tr><td>gnl|extdb|pgaptm
p_000434</td><td>K03723</td><td>mfd; transcription-repair coupling factor (superfamily II helicase) [EC:5.6.2.4]</td><td>MSQLRGLLPLLARDEAVEAVTPYLWEPGHGTVVCPVGVRPALVAHALSNRSGTAPVVVVTATGREAEQMARAISCWGPEATVFPSWETLPHERLSPQLDT
MARRVAVLRRLAHPEPNHPQAGPIQVLVVPIRALLQPIIGGLGDLRPVQARPGDFIDRDTLVHELVQLGYEPSDQVRGRGQVSVHGGIVDVFPPAGDHPVR
LEFFGDEIEEIRWFSLDDQRSLGAAETGLWAPPARELLLTDAVKEKARAAQIPGAADLLELATEGIYSPGIESLAPLLVERMDSLVDLLPVGTLLFMSEPERIQA
RAADLVTTSQEFLHASWGAAAAGGAIPLGAQSASLLSLDEVWGARKSASWWKLTALPGPELADQLAEQLAQREPAGADQTPADPPAAAVVASPRLAMIG
AREVRPYRGDFARATSDLGQLERAGWNLVVTTPGSGSARRLVQVLAEGDVAARLDPELTELPSPGLVHVTAAEAGAGFVLSAQELAILTEQDLTGGRSGGST
RDMRKMPARRKKGLDPLALRPGDFVVHEHHGVGRFLEMVSRTTGSGEQQVTRDYLVLEYASSRRGGPRDTLYVPTTSLDLVSKYSGSDEPKLSRMGGAD
WAKTKQKAQKAVQEIAAELVRLYAARQTAGGFAFSPDTPWQRELEDAFPFQETPDQLLTMDEVKADMEKPVPMDRLLTGDVGYGKTEIAVRAAFKAIQD
SKQVAVLVPTTLLVQQHYDTFRERYAGFPVNIAQLSRFSSPAEADRVRAGLLDGSVDLVIGTHSLLTGSVRFKDLGLVIIDEEQRFGVEHKETLKALRTDVDVLA
MSATPIPRTLEMAVAGIREMSVLQTPPEERQPVLTFVGAYSSAQVAAAIRRELLRDGQVFFIHNRVESISSVAAGLAELVPEARIRVAHGQLGEHQLEQVIVD
FWNHEFDVLVCTTIVETGLDISNANTLIVDRADTFGLSQLHQLRGRVGRGRERAYAYFFYPPGKTLTETALERLRTIAANADLGSGLAVAQKDLEIRGAGNLLG
GAQSGHIEGVGFDLYVRMVSDAVAQYRGEKPVETTEVRLDLAVDAHLPEEYIPGESARLEVYAKIAAVNDPGAMRELRDELADRYGPLPAPVERLFLLARLR
SMLRQLGVDEAVTQGNYLRLGPVELMDSTALRLKRLYPGSVIKPATRQLLVPLGGRRHHGEEPLVDEALVTWVEDLLRKVLVPNRRVALARAAKQGG</td></tr>
<tr><td>gnl|extdb|pgaptm
p_000428</td><td>K01142</td><td>E3.1.11.2, xthA; exodeoxyribonuclease III [EC:3.1.11.2]</td><td>MSSALITTLNLNGIRAAHRRGLMEWLALANPDVLLLQEVRAPEEVTEALLGPEWNVHTHPCAIKGRAGVAVALRRDGQFQAEGEPRVGLSPDEPDYDTGR
WVELDLVGPTDLTVVSAYFHSGSVGTPKQEQKMAHLPRIEVRLAQLLDQNALVAGDFNVVRSELDIKNWKGNHNKSAGVLDEEIAFLNRWVDSGWSDVV
RDLAGPNQGPYSWWSWRGKAFDNDAGWRIDYQYASRGLAQSAQRYEIFRAPSWDARFSDHAPVTVEYQFS</td></tr>
<tr><td>gnl|extdb|pgaptm
p_000425</td><td>K00600</td><td>glyA, SHMT; glycine hydroxymethyltransferas e [EC:2.1.2.1]</td><td>MSEPLLNLPLEQLDPEIFAAIQGELTRQRDHLEMIASENFVPMAVLEAQGSVLTNKYAEGYPGRRYYGGCEWADVAESLAIERAKSLFGADFANVQPHSGA
QANAAALQALAEPGDVILGLDLAHGGHLTHGMRLNFSGRYYRPIPYQVDPNTYRVDMDQVRSLAREHRPSVIIAGWSAYPRTLDFAAFREIADEVGAALM
VDMAHFAGLVAAGLHPNPVPFADVVTTTIHKTIGGPRSGLILSSRAEKWGKKLNSAVFPGQQGGPLMHVIAAKAVALKVAASPEFKERQERTIEGAKILAER
LSQPDMSELGIDVLTGGTDVHLVLVDLQNSALSGRDAEDLLAQINVTVNRNAVPFDPRPPMTTSGLRVGTPALATRGFQADDFREVADIIAVALREKAAGR
EVDVAGLLARVKALTDAYPLYPELDQ</td></tr>
<tr><td>gnl|extdb|pgaptm
p_001796</td><td></td><td></td><td>MEHVENLLVQSTSSRRGFVSELIGHLSSAGGKRLRPVLTLVSAQLGQDEPRDEVIKAAVVVEMTHLASLYHDDVMDSAPTRRGVDSAQHLWGNNRAILAG
DVLFARASLLTAELGPETVSYHARIFERMCEGQLNESFGPTAQDDPVEFYLQVLADKTGALVAAAAYLGAMHGGASEEHARIVEEFGERIGVAFQIADDVLD
LTSPRELSGKTPGTDLREGVDTLPVLLLRRREDADSQRILALLDTDLSSDRALAEVVDAICAHPVLEETRQMARDWAKAAEDALAPLPDSEAKTALLAFAAQM</td></tr>
<tr><td>gnl|extdb|pgaptm
p_001797</td><td>K00343</td><td>nuoN; NADH-quinone oxidoreductase subunit N [EC:7.1.1.2]</td><td>MNLQNFTAPAIDWMILLPVVVVLGGAVVGVLIEAFAPRRSRRRVNVVWTVLVLIAGLVTAALQWKGAVAGSSQTGQYISDPWSVGLQVILLLVALLAMLV
MADRTELGDGDFAAQPADRPGSGEEALSIAKKYQRSEIFPLLLFSVGGMMVFPATESLLTMFVALEVMSLPLYILAATSRRRRGQSHEAAMKYFILGAFASGF
FLMGAALLFGYSGGSLDFSQIAAGIPAFNNMEWLLVVGVFMVMVGLLFKVAAVPFHAWTPDVYTGAPTSITGFMAAGVKIAAFGAMVRFYQIVVGPLQ
WDFRMLFAVIAALTILVGTVGGLVQKDIKRLLAYSSIAHAGFLLIGVISLVKGSAGSIAFYLLAYAVATIGAFGVVTVVRVKDADGNIGGQATNLRLWKGLGKR
SPMTALAMLVFLLSFAGIPLTSGFVGKFVVFANGISGGLGWLVGIALVASAATAVFYFRVISYMFLQEPEEGTTVVSSEGLSAAAIAIAAVLTVLLGVVPGPILD</td></tr>
<tr><td>gnl|extdb|pgaptm
p_001798</td><td>K00342</td><td>nuoM; NADH-quinone oxidoreductase subunit M [EC:7.1.1.2]</td><td>MIEGSFPLLSILVAVPALGALLLWLVPSLRARGREFAIAVSLVELVLAIVVAFQFDWTAASAYQLFESASWIAPLGVSWSLAVNALGLVMILLSAVLVPLVLLAT
PASSDRRFEGGYAALILLLYAFIIVIFAAFDVVVFYLAFEAMLLPLYFMIGRYGTGENRRAAAMKFLIYSLVGGLAMLGGLVTIYSSAGHSGFNGVLFRYDTLAA
VLPQTAFGIQMAIFITFFIAFAIKAPMVPVHTWLPDTAAAARPGTSVLLVGILDKIGTFGMIVLCLTFVPDAAYASRWTILVLAVVSILWGGFAANGQKDLLRL
VSFTSVSHFGFMVLGIFIGSQVALTGAMFYMVAHGLSIAALFLISGFLIERGGSQEIARYRGMQRVTPVLAGTWLFAGMASIALPGLSGFVPEYLVLMGTYSV
NIPLAIFAVFGIVLAAMYILLPYQRMFTGPKRADLEDAPDLNGVQKLAITPLLVGMLALGIWSAPLVNSLSQVAEPTVVTVDEGN</td></tr>
</table>

gnl|extdb|pgaptmp_001804	K00336	nuoG; NADH-quinone oxidoreductase subunit G [EC:7.1.1.2]	MSDELVSLTIDDVEVQVEPGTLIIRAAEQTGVRIPRFCDHPLLKPVAACRQCLVEVGTPGPDGSIRMMPKPQPSCAIPVSPGMVVKTQHTSEVAYKAQEGIMEFLLINHPLDCPICDKGGECPLQNQAMTEGRDTSRFIDAKRVYAKPMRLTSQIMLDRDRCILCQRCVRFAKEIPGDAFLDLQGRGGGTAPTDDHTFMGEQVGTFDAEVLDYYVPGVEGPGRTDISGPYGQEGIISSVYAGPQRESERDLSGRTFASYFSGNIIQICPVGALTATSYRFRARPFDLVSTPSVTEHDASGSAIRVDIRRGEVVRRLAGNDPEVNEEWITDKDRFAFHWTDLEDRLRTPLVRENGELVPTSWSDALDRVARNLTGKVGLLPGGRLSFEDAWAWSKFARRVLGTNNIDARWREQSDEEAQFLGALVAGQGLGVTYQDVERAPEVLLVALEPEDECGSLFLRLRKGVKVKTVAPFLTRGSEKLSAQLIPAAPGAEVAALEQVELAEGAIILVGERAARTPGLLSAAASLAAKTGARLQWVPRRSGDRAAVEAGLLPNLLPFGRPVEDAEARESLPWGEQIPATPGLSAEQMLAGELDALILGGIDLRDWADPAAARAAIERTGFVVSLEVRRSELTELADVVLPVAPAVEKNGTFINWEGRLRPFGQSYASRSLTDRLVLNMLAKEMGVDLGVETLPDLYAEVNPLMVWRGARTAVPHEAPSAPASDQLTLTFHKVQIDLGRLLDGADHLANSARVPVALVSPATMERLGLAEDQQVTVTGERGSITLPIESREITEGTLWLPECSLGAGVHSEVGTAG
gnl|extdb|pgaptmp_001812	K03183	ubiE; demethylmenaquinone methyltransferase / 2-methoxy-6-polyprenyl-1,4-benzoquinol methylase [EC:2.1.1.163 2.1.1.201]	MTNDPLRADLAKEPSQVASMFNHVASRYDLMNSLGSLGQVHLWRRATVSAISPEPGERILDLAAGTGTSAHAIARTGASVVACDLSPGMIAVGQERYPELEFVEGNATDLPFADDEFDAVTISFGLRNVADVPAALTEMLRVTKPGGRLVIAEFSSPTNRFFDGLYRFYLGGVMPPVARAFSSDEVAYDYLIESILAWPGQQHLGEMIQQSGWEDVQFKNLSGGIVALHRAHKAY
gnl|extdb|pgaptmp_001881	K01883	CARS, cysS; cysteinyl-tRNA synthetase [EC:6.1.1.16]	MKLRLYDTKSRTVQVLEPVHPGRVGIYLCGPTVQGSPHIGHLRAAVAFDVLIRWLGRLGLSVTYIRNITDIDDKILTKAREAGEPWWQLAARYEREFEAAYRQLGLISPTFEPRATGHITDQVQLIQRLLDRGHAYADAAGNVFFDVHSQPDYGSLTNQQLGDLLTTEDPAQEDEANRGAKRDPRDFALWKAAKPEEPETASWPAPWGRGRPGWHLECSAMSHRYLGETFDIHGGGLDLRFPHHENEQAQSHAAGWEFARLWLHNAWVTQAGDKMSKSLGNTLALDALLEQYPASVIRLALGTVHYRSMIEWGDETLARAEGTWDRLAGFVADATARVGDPGPVDLPADRLPTGFVEAMNDDLNVAGALAVIYEHLKLGRRALSAGQDEAVRTELQLVRSMLDVLGIDPGSSHWARPDGTDPAGEAQYRALDSLVSTLVEQRNRARSEKDWAAADRIRDQLTEAGIVVEDGADGSVWKVRV
gnl|extdb|pgaptmp_001034	K00945	cmk; CMP/dCMP kinase [EC:2.7.4.25]	MSAVLVAIDGPSGSGKSTVARRVAEQLGIGYLDTGAMYRALAWLALSEGVDLHDEVAVRALADRLDLELSPGRVRVGEVDVTAAIRTPEVTAAVPPVARNLAVRAWMAQEQRRQMLAAREAGTGMVAEGRDITTVVCPEADVRVLLTASEEARLRRRTLELHGELTEETLEATRAQIVDRDRSDATVSEFFDPAPGVVLIDSSNASIDEVVEQILSLV
gnl|extdb|pgaptmp_001033	K04517	tyrA2; prephenate dehydrogenase [EC:1.3.1.12]	MSSLLATTGPVLVWGTGLIGTSIALALRGGGVTVYLRDISPTSLALAADMGAGVPVDEVEEPTPALVVVAAPPDVAAGCVIEALREYPRAVVTDVASVKKNVEDAVEAAALDSDLSRYVGSHPMAGRERSGAGYAVQDLFYGQPWVIVPTARSAPESVLAVRNLAVDLGAVPLEMTPAAHDEAVAYVSHVPQLISSLLAGRLLAAPGEALALAGQGLRDTTRIASSDPRLWTAIIAGNAGPIARILDDLGDDLQLLVERLRRFDEGNPGAVGIISEVMTAGNRGQSRIPGKHGGAQRRWAEIEVMVPDQPGALAQLFGDFGTAEINIEDLTLEHASGRRFGIATIMVDPANLLQAVDELEARGWRILSSGGVE
gnl|extdb|pgaptmp_001032	K06178	rluB; 23S rRNA pseudouridine2605 synthase [EC:5.4.99.22]	MSKDPHQSDGIRLQKVLSQAGVASRRAAEEMILQGRITVDGQVVRKLGTRVDPQKQTIHADGELVITDPAKHIVLALNKPVGMVSTMSDPEGRPCLSDVLADYPERLYHVGRLDIDTSGLLLLTNDGELANRLSHPSYEVEKTYVARLHGEVRPGVRKQLLRGIELEDGPIKVDSFKIKEQYGDITTVEITVHEGRNRLVRRLMEEVGHPVRELVRVRFGPIRLERLQPGTVRRIKGNDLTALYKAVDL
gnl|extdb|pgaptmp_001031	K06024	scpB; segregation and condensation protein B	MNSILSALEAILMVVEEPVTTERLAEAVGITPAETELALEELQRDFDGQAPGSRMRGFELQRVAGGWRIYSRPRWASVVGRFVVGSDAAQLSQAALETLAIVAYRQPITRLQVSQVRGVNVDSVMRTLQARNLIEEEGTSETGAYRYRTTPYFLECMGFETLDELVPLAPFLPDAAAVAALTIEVEEKNE
gnl|extdb|pgaptmp_001030	K05896	scpA; segregation and condensation protein A	MARPPSTPAELDAFQVELEVFQGPFDLLLQLIARRQLDITEIALAQVTDEFIAHMRRVPDLSTTTEFLVVAATLLEMKAAQLLPQTERDPGVDEDLSARDLLFSRLLQYRAFKGVAETIRERLSQQRLAVPRSVPLEPQFAQLLPELVWQTTPEQLAALALGVLAEKPRPDQAQHVNRPGASLEEELAIVEKRLRRGREATFADLVQDAANVAVVVTRFLAVLELYRRGDVQFTQPQELGPLTIRWEAPS
gnl|extdb|pgaptmp_001028	K04763	xerD; integrase/recombinase XerD	MADELGDPLAWFLDEMRVVRGASPHTVSNYGRDLGKYRTFMETRHLRRWEDVRPSTVEEFVAQLAHGDEEHPPLAASSVARTLSAVRSFHRWLMTQNLAQSNPAAQVKPPKTGESLPKALSVAQVARLIEGSQAGPELVALRNHALVELLYGTGARVSEATNLALDDLDLEGEYPSVRLFGKGSKERMVPLGAYAHAALGAYLSRSRPALAQRGRGTAHLFLNLRGRPLSRQSAWEIIQQAASAAGLEEEISPHTLRHSFATHLLEGGASIREVQELLGHASVTTTQIYTRLTPQGLLEVFQAAH

gnl|extdb|pgaptmp_001027	K00615	E2.2.1.1, tktA, tktB; transketolase [EC:2.2.1.1]	MALQWDERDDKAVVTAKVLAADAVEQAGSGHPGTPISLAAAAYLLYQRHLRFDPRDPHWLGRDRFVLSAGHASLLQYIQLYLAGAGLELGDIKRFRTMDSKTPGHPEYGHTDAIEITTGPLGSGLAAAVGMAMAARREHGLYDPNTPLGESPFDHNVYVIAGDGCLQEGISSEASSLAGTQELGNLILIYDDNHISIEDDTSISFTEDPVARYEAYGWHTQRVDWIGQDGQYTDDVEALHQALLAAEAETGRPSIIALRTIIGAPTPKMQNTGAIHGAKLGEQALEGLKEALGVNPKTMFDVDPEVVAYTRQNALDRAEAALADWRPKFAAWAEANPEQRELLHRVRSGEMPAGLAEALPEFPAGQAVATRAASGKVLNAIASVMPELWGGSADLAGSNNTIMSGYPSFFPAERSSKAFTGNAFGRNLHFGVREHGMAGIMNGIAADRLTRVYGATFFVFADYMRGAVRLAALMDLPVVYVWTHDSVGVGEDGPTHQPVEHLTAYRAIPNLAIVRPADAAETAQAWKATLEQQHPVGMILTRQALPNPARGEGEELSSAEGVARGGYILADTDGTPDVILMGSGSEVQLALAAREVLAKDGIAARVVSMPCMEWFQQQPQAYRDEVLPPAVTARVSVEAGLALPWTPYVGSTGKSVSIETFGAVGSANELFQKFGITTEAVVAAAREVLA
gnl|extdb|pgaptmp_001025	K01803	TPI, tpiA; triosephosphate isomerase (TIM) [EC:5.3.1.1]	MARTPLMAGNWKMNLDHLQAVQLVNELAMALEDRNFDYSSEVVVIPPFTDIRSVQTTIDGDNLKLAYGAQDVSLHDSGAYTGEVSATMLEKLGCKYVVVGHSERREYHGETDEIVGQKAQVVAAHNMTPIVCCGEVLEIRQQGTHVEHVLSQVRGALAGFTGEQVAKLVIAYEPVWAIGTGEVATPADAEEVCGAIRQLLVDLYGAEVAESTRILYGGSVNSGNVKELMAQPNVDGGLVGGASLKAQEFAKIADFKHL
gnl|extdb|pgaptmp_001023	K00134	GAPDH, gapA; glyceraldehyde 3-phosphate dehydrogenase (phosphorylating) [EC:1.2.1.12]	MTIRVGINGFGRIGRNYLRAIYEQGADVEVVAINDLTDAKTLAHLLKYDSVAGRLDAEVTYEENAIVVDGKTITAFAERDPANLPWGDLGVDIVIESTGFFTDATKAKAHLDAGAKKVIISAPAKNEDATFVMGVNEKDYNPATDNIISNASCTTNCLAPMAKVLDEAFGIERGLMTTVHAYTGDQRLLDAPHSDLRRARAAALNIVPTSTGAAKAVSLVLPQLKGKLDGYALRVPVPTGSVTDLSFVASKEVSVEAVNAAIKAAAEGDLKGILEYTEDPIVSTDIVGDPASSIFDSQLTKVIGNEVKVVSWYDNEWGYSNRLVDLTVYVGERL
gnl|extdb|pgaptmp_001022	K09762	whiA; cell division protein WhiA	MKDELVRIRVDQPTQVAAEAAAILRFAGGLHLVGGRILIEAEVDSLLVAHRLRTFLHLLYGAESTVVVVSGGALRKGKRYVVRVVKEADKIARLTGLVDAAGRPVRGLPAQIVGAGVDEAIAAWRGAFLARGSLMEPGRSASLEITCPGPEAALALVGCARRIGATAKTRQVRGADRVVLRDADAIGTLISALGAKETFATWQARREKREARGSANRLANFDDANLRRSARAAVAAGARVERAFEILGDSVPDHLREAGQLRVQFKQASLEELGQKVDPPLTKDAVAGRIRRLLAMADKLAAEKGI
gnl|extdb|pgaptmp_001020	K06958	rapZ; RNase adapter protein RapZ	MTREPDPLTVPSGIPALDALSVRSEPVENEIIIVTGRSGAGRTHAANALEDLDWYVVDNIPPALLPHLAGMMTPVGDGVHRLAAVVDVRGREFFHALEKALDELRAAGYPYRIIYLDASDRELVRRYESNRRPHPLQGEGTLLDGLTAERQLLQPLRDRADEIIDTTKLSVHDLSRHVRDLVASEQVRPVQITVESFGFKHGIPLDADFVADMRFLENPYWVEELRHLTGKDQPVADYVLNQPGVREFGDTYARLIEGTLDGYRRELKPYVTVAIGCTGGRHRSVAMTEYLARRLRRRGHQVQVM
gnl|extdb|pgaptmp_001019	K03703	uvrC; excinuclease ABC subunit C	MAARQPWWRPPTSEIPTEPGVYRFRDGEGRVIYVGKAKNLRARIVNYFQDPSVLHPRTAQMVAHARSVQWTIVASEIEALTLEFQWINELEPRYNVIFRDDKSYPYLAVSMGEQFPRVAISRDARRPGTRYFGPYTHVWAIRETIDNLLNTFPVRTCSPGVLRRAQAQGRPCLLGYIDRCSAPCVGRISEADHRHLAEELCSFMEGKTAPFVRQLEAEMREAAAQQDYETAAKRRDQLRALEKVQERNTIVLPVDTDADIYALIGDDLDVAVHVFYVRGGRVRGTRGWVIERVDERTEPELMRDLLEQTYLDQPARRQKVAPVSVDDVEHTPLEAIPARILVSHQPAETQFLEEWLGSLRGAKVKIGVPRRGQKADLMNTVAQNARHSLEVYKTRRAGDLTQRAVALEELQTALGLAEAPLRIECFDISHTGGTNRVASMVVFEDGAPRKDAYRTYNIQGQGETSDDTAAMSEVLTRRFRRGVEPDEDLESGAIDAQTGKPRRFAYRPDLLVVDGGLPQVNAARAALDEVGVEIPVVGLAKRLEEIWLPAEPFPVILPRSSAGLYLLQHLRDESHRFAIKQHRAKRSKAQTRSALDAVPGLGPARQKALLKTFGSIRKIRAASAEELASVPGVGPKLAATIQASLAGGGKPSAAGQEGPEGGKLGP
gnl|extdb|pgaptmp_000786	K01897	ACSL, fadD; long-chain acyl-CoA synthetase [EC:6.2.1.3]	MSNENNPSADDVSEDSPVTEALEWTGPVLERVDEHLSIPALLERRVSANPRSPIIELKSEMGATWRPIYAGDFWREVQAVAAGLIGMGLEFGDRFAIMSHTRYEWTLLDFAGWTAGLAPVPIYETSSASQIAYILQDAEVKLVVAESISNANLVRAAAEQAGVSVQVLSLDSQAIATITEAGRTIPVTEVTARANRLTTDSLATLVYTSGTTGTPKGVVINHGNFTELTVNSYLWMPEVASHPKSRLLLFLPLAHILARFLQVYQVCSQGVLGHSPDIKNLLPDLASFRPSYLLVVPRVLEKIYNSADASTGSGVKNRMFRWAANTAVEYSKALDTEEGPSRALATQHKLASALVLSKIINLVGGNAEFVISGGAPLSEHLSHFYRGAGLPVLEGYGLTETTGPISVNTPRLNKIGTVGPPLPPMSVRISDDGEVLVKGSSVFQGYLHNQELTDQVLKDGWFATGDLGSLDRDGYLRITGRAKEVIVTAGGKNVIPSALEDELRGHPLISQIVVVGDKRPFISALITLDTEMLPLWLKNKGLPALSAAEAIENIEVRASLDRAIARTNEKVSRAESIRKFEILPTDLTEANGMLTPSLKVKRSVVLREFADVIDRIYGGPVEY

<table>
<tr><td>gnl|extdb|pgaptmp_001015</td><td>K03701</td><td>uvrA; excinuclease ABC subunit A</td><td>MTNELIIRGARQHNLKNVSLELPRDQMIVFTGLSGSGKSSLAFDTIFAEGQRRYVESLSSYARQFLGRMDKPDVDFIEGLSPAVSIDQKSTSRNPRSTVGTVTEVYDYLRLLYARAGVSYCPVCGERITAQTPQQIVDQILNWPEGTRFQVLAPVVRGRKGEYSELFAELLARGYSRAKVDGEMHRLDNPPVLEKKLKHDIDVIVDRLVIREGLKGRLTDSVENALALAEGLVVTDQVDLAEGDPDKYRRFSETRACPNDHQLGLEEIEPRTFSFNAPYGACPECAGLGIRTEVDPGLLVPDEELSLSEGAVAVWGPNQRYHQQVLASLGEQLGFDVDAPWKDLDPEVRQAIIFGKDFEVHVKYKNRWGRQRAYTTGFEGAANYVERKIEETDSNYVRDKLDGYMREVPCPKCHGARLRPEVLAVRVGGLNIAELTALSIDEALEFLTQLELTGREAQIAKPILTEILARLNFLVNVGLTYLTLARSAGSLSGGEAQRIRLATQIGSGLVGVLYVLDEPSIGLHQRDNRRLINTLEHLRDLGNTLIVVEHDEETIESANWIVDIGPGAGERGGDIVYSGPVAGIMTHEDSLTGQYLSGAKQIRIPAERRPVDPDRVLTVKGAKENNLQDIDVTIPLGLLVAITGVSGSGKSTLINQILYRSLANRLNRARLIPGRHRSIQGLEHLDKVVHVDQSPIGRTPRSNPATYTGVWDHIRKLFADTNEAKVRGYGPGRFSFNVKGGRCEACKGDGTIKIEMNFLPDVYVPCEVCRGARYNRETLEILYRGLNVADILDMPISQAADFFTAIPRIARHLNTLVEVGLGYVRLGQAATTLSGGEAQRVKLASELQRRSNGRSIYVLDEPTTGLHTEDIRKLLLVLQSLVDKGNTVVVIEHNLDVIKCADWVIDMGPEGGSGGGQVVATGQPEQVAE</td></tr>
<tr><td>gnl|extdb|pgaptmp_001196</td><td>K09780</td><td>uncharacterized protein</td><td>MALYVIEYRYDEDLSSLVNDFRPAHREYLRRLQDQGNLVASGFLRDAVFNGALLILNAETAQQATALLNEDPFVTNGLIHSFQVREWHPTLGYLADDFDKTFPAS</td></tr>
<tr><td>gnl|extdb|pgaptmp_001197</td><td>K01055</td><td>pcaD; 3-oxoadipate enol-lactonase [EC:3.1.1.24]</td><td>MTAFELQQDGPAGAPTLVLAHSLGSDVRIWDEVVADLADSWHIVRWNLPGHGASEIPSGPARMETVVEVLLSQLDEAGIGRFHVAGISLGAIASLAVAEMAPERVLSLAMLDSGAALLPSEPWFDRAELVRAEGMSSLVDATMDRWFTPEFRAGKGESRYRRTRETFLACPVEGYAYCCELIGNTDLRADLSQIQVPTLILTGDEDAGMSPGKAEELAHQIPGAAGSFQVIKNSRHLTCVEHPRQVAAALTQVMCQVI</td></tr>
<tr><td>gnl|extdb|pgaptmp_001198</td><td>K12952</td><td>ctpE; cation-transporting P-type ATPase E [EC:7.2.2.-]</td><td>MSFPPYPDGLPDEAVAERVQAGQTNRVPRTTSRPVSQILRSNIFTPFNAILTTAMVVVLAMGDWRDSVFALVMVINAAIGVCSELRSKRVLDSVAVLAAPTSQVLRAGKVQTVNNDDLVVDDLVELRLGDQIPADGTVLSTRGLEVDESILTGESLPVKKQVDDQLLSGTAVVAGSGRMWVRQVGADSWAQKITAEARKYSVVTSEIQRAIDRVLKWIMVVLPVVVVLLVWSQTRADAGNWRGAVILAIAGVVGMIPQGLVLLTSMNFGLAAATLSRRGVLVQELPAVEILARVDELCLDKTGTLTTGGIRGQELTVLPGAASEADLAGALGRLTSDETNASATAVQELLRSQAWSATDLPTELTEVPFNSTRKWSALSGANGSWVFGAPEILLANARGDAAEAARQLVAESSSRGQRTLCLSRAGGPIAENQPLPEDLSPQLIAVLSEDIRPDAAETLEYFRSQGVRVRVISGDAPGTVGAIADRLDLGGDGRTLQVVDARTLPEIDTPEFDRATAEVDVFGRVTPEQKRALVRSLQRGGHTVAMTGDGVNDALALKEADLGIAMGNGAPATKAVSRLVLMKSEFSVLPGVVAEGRRIIANMERVSSLFLSKTTYAVMLAVIVSVCGWVYPFLPRQLTFIGALTIGTPAFFLALAPSHRRYHPGFLRRTLWLAVPSGLLMGTGALIVYLLLGEHTEIGQTGATLTLILGALWLLGITARPFNLWRLVLICVMAAGALLGVLIPWTREFFALQWPDPQQWALIGGVGLAISALMEVCYRYTTRFRHEGS</td></tr>
<tr><td>gnl|extdb|pgaptmp_001199</td><td>K05794</td><td>terC; tellurite resistance protein TerC</td><td>MEVSVWSWLILGAIVLVLITFDIVGHVRTPHAPTMKEAAWWSVGYIGLAILFGVLTWWEFGSVYAGEYFAGYLTEKALSVDNLFVFVLIMQSFRVPREYQQKALLSGIIIALVLRLVFILVGAAIIERFSAIFYLFGAWLIWTAIMQARQGVEGESDDGEYRENRFTIFVRKMFPVTDGFVGQRFLARQHGKTYLTPLLLCVLALGTADLMFAFDSIPAIFGLTQEPYIVFAANAMALLGLRQLYFLIDGLLSRLVYLHYGLAIILGFIGVKLILHAMHENNLPFLNGGEGFHVPDIGIGMSLSVIGGVIVLTVVASLIKSRMDRARPPQVEQ</td></tr>
<tr><td>gnl|extdb|pgaptmp_001200</td><td>K03702</td><td>uvrB; excinuclease ABC subunit B</td><td>MILRSDEHPFEVISEYTPSGDQPTAIAELARRLNEGEQDIVLLGATGTGKSATTAWLIEQIQRPALVMEPNKTLAAQLASEFRSLLPNNAVEYFVSYYDYYQPEAYVPQTDTFIEKDSSINDEVERLRHSATNSLLTRRDTVVVSSVSCIYGLGTPEEYVARMIELEVGMRIDRDELLRTFVDMQYSRNDIAFTRGTFRVQGDTIEIIPVYEELAIRIELFGDEVDSLAVLHPLTGQLIRETKRVHVFPASHYVAGRERMQRAIASIEEELEERVRWFEQRGKLLEAQRLRMRTTYDLEMLREMGMCSGIENYSRHIDGREAGTPPHTLLDFFPDDFVLIIDESHVTVPQIGAMFEGDMSRKRTLVEHGFRLPSAMDNRPLRWEEFLERIGQTVYLSATPGPYEMSKAPDVVEQIIRPTGLVDPKVVVKPTEGQIDDLLDEIRVRVDRDERVLVTTLTKKMAEELTVYLAERGVRVEYLHSDVDTLRRVELLRELRLGKFDVLVGINLLREGLDLPEVSLVSILDADKEGFLRSTRSLIQTIGRAARNVSGEVHMYADQITDSMREAIDETERRRARQIAYNEERGIDPQPLRKKIADVTDMLAREDIDTQELLAGGYRAGNELSEPAAGAPSIRTQLSAQAEGDLAGLIEQLSAQMHAAADDLQFEVAARLRDEINDLKKELRAMRAAN</td></tr>
<tr><td>gnl|extdb|pgaptmp_001201</td><td>K00859</td><td>coaE; dephospho-CoA kinase [EC:2.7.1.24]</td><td>MSLLRLRPPSHPEPGQPYLVGLTGGIGTGKSTVAEQLGALGAVVVSADELAREVVAPGSPGLEQLAREFGTGVVRPDGQLDRAALARLVFDDPAARLRLEQITHPLIAAAKIEQFAGLAPGQVGVYDVPLLVETGMEGEFDAVVVVEAPLDLRLARLHRRGLPNREARARIEAQATDEERRALAHFVLVNDGDRDQLDRSVLEL</td></tr>
</table>

gnl|extdb|pgaptmp_001203	K02945	RP-S1, rpsA; small subunit ribosomal protein S1	MTTSSVQPTAGTPQVAINDIGSEQDLLKAIDETIKYFNDGDIVEGTVVKVDRDEVLLDIGYKTEGVILSRELSIKHDVDPEDVVAVGDKIEALVLQKEDKEGRLLLSKKRAQYERAWGEIEKIKNEDGVVSGTVIEVVKGGLILDIGLRGFLPASLVEMRRVRELGPYIGRELDAKIIELDKNRNNVVLSRRSWLEQTQSEVRTNFLNTLQKGQVRKGVVSSIVNFGAFVDLGGVDGLVHVSELSWKHIDHPSEVVEVGQPVEVEVLDVDMDRERVSLSLKATQEDPWQTFARIHAIGQVVPGNVTKLVPFGAFVRVEDGIEGLVHISELAQRHVEMPEQVVKVGDDVFVKVIDIDLDRRRISLSLKQANEGVDPTSEEFDPSLYGMAAEYDENGNYKYPEGFDPETQEWMEGYDSQREAWEAQYAEAQARWEAHRAQVQAALEADSEQAPSDEQAPTSYSSADASEGTLASDEALAALREKLTSNN
gnl|extdb|pgaptmp_001204	K02335	polA; DNA polymerase I [EC:2.7.7.7]	MVAYVSKTLLVIDGHSMAFRAFFALPVESFTSKGGQATNAVYGFLSMLVRLIDTEHPTHIAVAFDVGRETFRNREYPEYKGGRDETPAEFKGQVEIITELLHRMGIRTLTKDDFEADDILATLTREGTEAGFRVLLASGDRDTFQLVNDQVTVIYPGRSASDLKYMDPAAIEERYGVVPERYPEIAALVGESADNLPGVPGVGPKTAAQWLNKFDGLDNLLAEADQVGGKRGEALREHVEAVRRNRRLNRLVSDLDLGVEVGDLVPTEADYQGLQQLFDALDFTGLRSRVYQALQVEPNGLSEPADSGEDDGVGLERTLVVEPGRNVADWLRSRDHFALAYSGVGRPAAGDLTGFALLAEGEALVVDPAQLDPAQDRAFADFFASDPDLVIHEAKAATHAFAARGWQLPGPSFETELAAYLCRPDRRGYSLAELAREYLGEELEEADEGALFSAADLDEDAEPGWVRRLAQQALLTARLEAPLRAQLDADQMTALLTTMEIPMQRVLARMEQAGVAMDLDVLDELAQELAAGAARAQEDAFAAIGHEANLSSPKQLQVILFEELGMPKTRKTKTGWTTDAGALTDLFAKTGHPFLEALLRHRDHTKLGQMVEGLRAEVKPDGRIHTTFQQTVTATGRLASAEPNLQNIPTRTATGRRVRGAFICGPDYEALMSVDYSQIEMRIMAHLSEDQELIAAFNSGEDLHRTMASMVFGVPVSEVTSELRSRIKATSYGLAYGLSPFGLSRQLGVGVDEARDLHSRYFERFGGVGRYLHEVVEVARQTGYTETMFGRRRYFPELRSDSHRVREMAERAALNAPIQGSAADIIKIATNDVYQRLRDGKYRSRLLLQIHDELLLEIAPGERKEVEELVRESMGQAAEMSVPLEVAVGVGRSWKDAAH
gnl|extdb|pgaptmp_001205			MSRTPPSAGQNCPQTASSLSDKLQLEVLECTPERTVVAMPVAGNTQPMGALHGGASAALCETAASLAACAHGEPNGLVGVGTNLSITHLRPVFSGRVVAEAVATLLGRTVTAHSVQVHDDAGRLVAIAQVSNQLIAKPV
gnl|extdb|pgaptmp_001232	K22010	pdtaR; two-component system, response regulator PdtaR	MTKESVDTKRVLVAEDETLIRLDIVETLRGAGYEVVGEAGDGEEAVKLALELEPDLIVMDVKMPKMDGITASEEILKEISCAVVMLTAFSQTELVERASEAGAMAYVVKPFGPADLIPAVEIALSRYSQIESLEDEIADLAERLETRKRVDRAKGLLMEKMEMSEPEAFRWIQKTSMDRRLSMREVADAVIDQVAE
gnl|extdb|pgaptmp_000883	K22468	ppk2; polyphosphate kinase [EC:2.7.4.34]	MDKDLYEAELRRLQAELVEMQAWVKATGARIVVLFEGRDAAGKGGAIKRITEYLNPRIARVVALPMPTERQQTQWYFQRYVEHLPAAGEIRLFDRSWYNRGGVEKVMGYCTPEQHQLFLRQCPIFERMLVDDGIIVLKYWFSVSDKEQMKRFRSRLTDPMRRWKLSPTDLESITRWEEYSRAKDEMFVHTDIREAPWFVVESDDKRKARINVIHHLLSKIPYTHIDRPTLEIPKRPKSTGYRRTDRSLQHEIPDYAASLSQESVQFTYRDVEDDELPDNGEVDSSEQNHPNQANPAQANETDE
gnl|extdb|pgaptmp_001253	K00873	PK, pyk; pyruvate kinase [EC:2.7.1.40]	MRRAKIVCTIGPATDSPEQIQALVDAGMDVARINRSHGTAEQHEQVIKRVRRASETSGRPVAVLVDLQGPKIRLETFAEGPQRLEVGDIFTITTRDVPGTKELVGTTFKGLPGDCRPGDRLLIDDGNVSVRVIEVTDTDVVTRVEVPGMVSDHKGLNLPGVAVSVPALSEKDREDLRWGLRVGADFIALSFVRNAADYDDVRQIMEDVGIVRPVIAKIEKPQAVDALTSVIDAFDGIMVARGDLGVEMPLEAVPLVQKRAIELARRASKPVIVATQVMDSMIHNPRPTRAEASDCANAILDGADAVMLSGETSVGDYPIEAVRTMASIIESTEENGGERIAPLGAFAVDRSGVICEAAANIAESLDSRYLVTFTQTGSTAKQMARLRSPIPHLAFTPIVEVRNQLALSWGVQPILAPEAEHTDQMVALVDEVVQDARIAHMGDGLVLVAGMPPGVPGSSNLVRVHNVGDLKTQ
gnl|extdb|pgaptmp_001254	K13292	lgt, umpA; phosphatidylglycerol---prolipoprotein diacylglyceryl transferase [EC:2.5.1.145]	MIAMSIPSPAQGVWWLGPIPIRAYGILMVTAMALATWVAYKRYRARGGVGEVVLDAALWAIPFGIVGGRLYHVFTTPESYWGPGKDPWAILRIWEGGMAIWGAVALGAVGAYIGLRRAGQRVGPFADALAPGLLLAQVLGRWGNYFNQELFGGPTTLPWGLEIDAAHLPPGYPEGTLFHPTFLYEGIWNITMALLLIWLDRQFRFKSGQVMSLYLVMYGVGRFWVEAIRIDEARTYLGLRLNGWTALFVVVLGIILFFVTRRVGAPAEVLPPERADYFARVGKQDVEEADDSARVAVELAEEDEPLPDSREETAI
gnl|extdb|pgaptmp_001255	K01695	trpA; tryptophan synthase alpha chain [EC:4.2.1.20]	MTSSTPVIKAQVARGNAALIGYLPVGFPSVPQSIEAAQVLLDNGVDAIELGFPYSDPAMDGPVIQQATTIALQRGVHLEDLLGAVEQLSAGGAPILSMTYWNPVHWYGVERFARDFAAAGGSGLITPDLPPEESAQWRAASEQYDLERVFLAALSSTEDRLKLIAQESRGWVYAASAMGVTGQRSWIDERLSALVGRVRAAGADTVCVGLGVSNGQQARDIGAYADGVIVGSAFIKPLLTQEYDRALASLAALAQELRRGVDGARKGNN

gnl|extdb|pgaptmp_001256	K01696	trpB; tryptophan synthase beta chain [EC:4.2.1.20]	MSEQVPESTQHYFGQFGGRFAAEPLMAAIDELDREWHRLWHDDSFRAEFDLLLRDYAGRPSLLTEVPRFAAGTGARIFLKREDLNHTGSHKINNVLGQALLTRELGKKRVIAETGAGQHGVATATAAALLGLECTIYMGKEDTERQALNVARMQMLGAEVIAVTQGSQTLKDAINEAFRDWVTNVETTNYVFGTAAGPHPFPDMVRDLQRVIGDEARAQILAQTGSLPDAVFACVGGGSNAIGIFTAFLSDPEVALFGAEAGGDGVTTGRTAASITVGEVGILHGAKTYVLQERDGQTRASHSISAGLDYPGVGPQHAHLAESGRATYLPVTDAAAMEAFRRLSQTEGIIPAIESAHALAAALEWLESGQAPAHPTVIVNLSGRGDKDMDTAAAYFGLGQAKEE
gnl|extdb|pgaptmp_001257	K01609	trpC; indole-3-glycerol phosphate synthase [EC:4.1.1.48]	MSVLDEIIAGVREDVARREAEVSLDELKLRVQQAPDAKDVISALRDVPGAVSIISEVKRASPSKGQLSDIPDPAHLARLYEEGGAAMVSVLTEERRFHGSLADLDAVRAAVDIPVLRKDFIVTPYQIHEARAHGADAVLLIVAALEQPVLVSFVERVKSLGMTPLVEAHSRLEALRALEAGADLIGVNSRNLKTLDVDRRVVEEVIDVIPAEVVAVAESGVRNSRDVLDYALVGADAVLVGEALVRSGNPLEQIKDMVSAGQHPALKTDRKQRVKDAREDM
gnl|extdb|pgaptmp_001258	K01496	hisI; phosphoribosyl-AMP cyclohydrolase [EC:3.5.4.19]	MGLERAIRERLKWNEAGLVCAVVQDYDGGQVLMVAWMDQEALARTLAEGRVTYWSRSRQEYWRKGDTSGHRQYLRGISVDCDGDALLLQVEQIGAACHTGTRSCFEAGGPLPFTSIEAGGTVR
gnl|extdb|pgaptmp_001265	K02500	hisF; imidazole glycerol-phosphate synthase subunit HisF [EC:4.3.2.10]	MVAIRVIPCLDVAGGRVVKGVNFENLRDAGDPVELASVYSAQGADEVTFLDVSASLEERGTMRETVRRTAAEVFVPLTVGGGIRTVADVEALLACGADKVSVNTSALARPELITEVAKAFGSQVLVLSVDARRVKGEFQPYRYEVTTHGGKRSAGVDALAWTHEAQERGVGEVLLNSMDADGVRTGFDLEMLSQVREVTSVPLIASGGAGEVHHFVEAAQAGADAVLAASVFHFGVVRVSDVKEALAAAGFEVRPA
gnl|extdb|pgaptmp_001267	K13571	pafA; proteasome accessory factor A [EC:6.3.1.19]	MDRRIYGVETEYGITCAGGDLDAEAAARELFAPLIQRGRTTNMFLPNGGRLYLDVGAHPEYATAECDNLWDLLAQDRAGSQLLSDLATEANGRLEAGRIHLFRNNFDFDHNSFGCHENYLLRRRRDFREVADALVAYFVTRQVVAGAGDLKRNPEGQLQYVFSARADQMFDSVSAATTRARPIINTRDEPLADATEYRRMHVIVGDSNMAEPTTALKVGATELLLDAVDRGIHLADLALADPIEAIQAINFDLSGQLLLPMQDGRQLRAVDIQQEILDRILGLEPELSEMQRYLVGLWQRAIEAVRSGNWAPIETEIDFAIKKRLVDQYRERSGAELADPRVARLLLSYHDITEAGLRGKLEAGQVMLRLTSPEQVTVATTTPPPTTRAHLRGRVIAAAERNRRDLACDWIHLRLEDGGLNLALQDPFATESEAVDQLIAAIEQSSPQLPA
gnl|extdb|pgaptmp_001268	K13570	pup; prokaryotic ubiquitin-like protein Pup	MSSTQIYGGSGPDDEEPMDLPGGQIQVNALDSILDEIDTVLEQNAEAFVQGFVQKGGQ
gnl|extdb|pgaptmp_001269	K20814	dop; Pup amidohydrolase [EC:3.5.1.119]	MKRVIGTETEYGVYRPGDPAANAVALSTEAVQAYAPYARGGLSHHGPVEWDYRGEDPLNDIRGRRLERASADPSQLTDDPYHLAPPGGSETVAAPTPEELRQFRPTSIVLSNGARLYVDHAHPEYSAPEVASARDAVRWDRAGEVVAQRIMSEADLVLVKNNVDGKGATYGSHENYQVPRSVDLDDLIRFIIPFMVTRPIFCGSGRVGLGPRSDQPGFQISQRADYVENDIGLETTFNRPIFNTRDEPHADGHQWRRIHVINGDANLFDVSILLRTGTTALVLWAIEQGTDLTWEGLDLLGPIGAVKTVSRDLTLQTKLPMAEGSEMTAIEIQRRYLEKVLDAFAETGTQPTVDQQEVLDRWDQTLTKLATDPMSLAGQVEWIGKYQLLERQRQRLGVGWDDARLVAMDLQWSDLRPEKSLVQALAARGLVERLFSDEEIAWAADNPPSGTRAQARGQAVSTRPDLKQASWTSLVFDEGQAESYRRVPLADPTK
gnl|extdb|pgaptmp_001270	K13527	mpa; proteasome-associated ATPase	MSEMTPDTVSQLRLSVAGLEEKNHRLSKALVVARDQIEGLQEQLERIHRAPATWATFIEAFPEKEELEVYHAGRLMRVTSAPSLRLDDLSPGQLVRISDQMVAVAPGEFPRTGSLGSVLELHGTDRVLVSIDTGQEHVLRLAGTLRHGGVKPGDTLAVDLRSGFAYERLVRSHIEQLFTPEKPDVSYADIGGLDAQIEMVRDAIELPFQYPEKYRAFGLRPPRGILLYGPPGTGKTLIAKAVAASLGGPEAEQETYFISIKGPELLNKYVGETERLIRAIFGRARALASQDVPVVIFFDEIEALFRTRGSGVSSDVETMIVPQLLAEMDGVESLDNVVVIGASNRPDMIDPAVLRPGRLDVRVRIDRPTRRQARDIFSKYLTPDLPLDPELVSRAGSPEAAAKAMIEAALDRLYTQDETTLLFDMHLADGKVRQVYLSDLVSGAMIAGTVERAKKRAIKDSLEGGAQGLTTAHVLAGVDEEVQESADLAATTTPDEWARTVGLRAEEVVRIDTRRNQ
gnl|extdb|pgaptmp_001271	K07442	TRM61, GCD14; tRNA (adenine57-N1/adenine58-N1)-methyltransferase catalytic subunit [EC:2.1.1.219 2.1.1.220]	MSKDMGQARRRGLLQPGDRVQITDPKGKMHTIILVPGGRFQSARGALNHDDVLGGPDAQVVTTEEGRTFQVIRPLLSDYVLSMPRGAAIVYPKDAAQIVQMGDIFPGARVLEAGVGSGALSLSLLQAVGEGGHLLSVEQRPDFAQIAAANVDLWFGQRHPAWELQVGDVGEVAGGLEPASLDRVVLDLLDPWSYLEQVRRALRPGGVLICYVATVTQLSRVADDLRATEAFTEPQSWESTVRPWHVEGLAVRPEHRMVAHTGFLLTARLLGQDQHVHQLARRPAKAAVDQPGKWARVTNWRLEDLGMRSQSPKKTRRIQRDLEGRLNQWLGR

gnl|extdb|pgaptmp_001272	K07465	putative RecB family exonuclease	MRAPALSASSSREYLQCPLKFRYSVVDRIPQPPTAATIRGILVHSVLEHLYGLAAADRTWEAALDLLPTRWEHLEQKEPEYSEVIESVPELLAEAQKLLQTYFTLENPHNLEPEARESFVEARLPSGLMLRGIVDRIDRAPDGRRRVVDYKTGKSPSPRFLDESLFQMRFYALLLREVGRAPSRMQLLYLKDGQTLTLDPNPEDVDRFEGELLDLWHRIAGDLGSGQFSPRKTPLCGWCPYQAQCPLFGGQIPPAAEEDLARVSQIGPSN
gnl|extdb|pgaptmp_001274	K06153	bacA; undecaprenyl-diphosphatase [EC:3.6.1.27]	MDWLDALIFGLVQGLTEFLPISSSAHLRYVGELIGSSDPGAAFTAITQIGTETAVLIYFWKDIVRIISRWWGSLRRQVPTSDPDVRMGWMIIVGSIPIGFLGLLLEDWIDTSFRNLWITVLMLAAFGLLLGWADRVGSSKKELTNLSWRDAILFGFAQALALIPGVSRSGGTITAGRLMGYSRVAAARYSFLLALPAVFISGFYKVFKVATGGDVIYVGPTIFATVVAFVVGYLVIVWFLKLISHVSFTPFVIYRLAFAALIALSLLVGWVPAGGL
gnl|extdb|pgaptmp_001275			MEQRLLGEHGLILSSLGLGTRTWGLDTDPHEAAEMLTVYRDAGGSVLEVEDDPRFPEPAQTVGELTRPGELQLILRSTGRLPARGSLLDSLDRTLTHLGADHVDLWIPMGPRREAPLAELVEAAEVAWRSGRARYVGLGGLSWWDGGAATTMGPFFSAWAGRLSILEPALSQARAVRDAGLGLIAGAPLAGGMLTGKYRHSTPPDARATSPRFKAELAPYEEAGPRAVIEATCRAAEGLERSPAQVALAWARDEAGVTSAIIGPRGVRQLEHLLQIDGWRLPRALRDVLTEVALRPN
gnl|extdb|pgaptmp_001291	K22745	AIFM2; apoptosis-inducing factor 2	MAKVVVIGGGYGGITVAKGLDSVADVVLIEQRDQFVHHAAALRAAVDDVWGNAIFMPYSYLMQRGQVVHGTVSRVEGTTVHVFGQEPIEADYVVLATGSNYAFPAKHTDGSNKVAKARIEQLHESLQHADSALLLGAGTVGLELAGELAHAYPHMHIEIVEKEGEILPNPGYTAEFRHEIEEQIAKLDVKVHLNTRLAVRPPTAVGELGHFEVTTTSGDSIEADIWFQCYGSTTATGYLAGTEYQDLLTRDGTIQVEPTLQVVGHPHVYAVGDITNIDDSKRADVARQQARVAIANIANQIDGGTPDVMYESSKEWVVVPLGPNGGASQLLDSHGRTRIVGAEQTAEIKGADLMVSVIRSQLNLP
gnl|extdb|pgaptmp_001276	K01284	dcp; peptidyl-dipeptidase Dcp [EC:3.4.15.5]	MTNLLPDFSTLTLDQIEADILAGMEEQRAQWEEVATNPEAPTVANTLVALDESGARLDRAGAIFWTLASSIGGDELDRLQEKLVPLLTEHSNTYLLDERLYRRFLELSDPDPQTQWEIAEQIRAFEQHGIACPDKEQLRRLNVRIAELETEVDQRISKQLEETGLSVTDEAELAGLDEATRAGYRRDHDWFIRCRNFSTQLDQSVLEEPRVRRALLEVSVTRGRTGATDTRALIVELTQLRSERAHLLGFPDHATVVMQGETIPGPDAAQDLLVEVGRAARARVDEDAQKLRQLCPDLEAADWPFYENQLRAQELGFDAESLRPYLELNRILTDGVFYAANRLYGITLTERPDLRGWHEDVRVWEVKEEDGTALGLFLGDYFTRPGKSGGAWMSELQEAHPASGQLPIITNDANFTKPAPGQPLLLTWDDVETLFHEFGHALHGLFSDTKYRDSAGTNVPRDFVELPSQLNEMWAFHPEVLSRYARHYQTGEPLPADLLAAVVGSKSFGQGFATLEYVEAALIDQAWHREGTLPTEADQVPTFEVEALERLDLWHDLVPPRYRSTYFAHTFAGGYDAGYYAYMWAETLAAEVEEWFRAQPNGGLTREAGEKLRRELLSRGNSRPPLDSFRAVTGKDASAQSVLRRRGLV
gnl|extdb|pgaptmp_001111	K00145	argC; N-acetyl-gamma-glutamyl-phosphate reductase [EC:1.2.1.38]	MPLTAAVAGASGYAGGEVIRLLCSHPQISPVTVTAHSQVGQRLGSVQPHLRQVAEAELVETTAENLAGHDLVFLALPHGKSGELSDRLAQLSPETVVIDCGADHRLHHRDDWDAFYGGEYHPAWTYGVPELPVAGGKQRAALQQTKRIAAPGCNASTVALSLAPGIQAGLIDPSDLVSVLAVGPSGAGRALKPNLLAAEILGSASPYAVGGVHRHIPEIKQALRETLPADRQPEIQLCFTPVLVPMARGILATSTARVQPGVGEAELRAAWEAAYSDEEFVQVLPAGQHPATAATVGANTLLMSLTFDPASHRLVVVAALDNLVKGTAGAAIQSANLALGLPENSGLTMNGVAP
gnl|extdb|pgaptmp_001112	K00620	argJ; glutamate N-acetyltransferase / amino-acid N-acetyltransferase [EC:2.3.1.35 2.3.1.1]	MSVTVPEGFAAQGVAAGLKSTGLADLAVVVNLGVNPVGAAVFTSNRAKANPILWSEQAIRSGQVRAIVLNSGGANCFTGEFGYETSVLTAKLAAGELGVDPNQIQVCSTGLIGEGGPAFREAILHALPGALATAHEDGGEAAARAIMTTDTRPKMAAYRGEGWSIGGMAKGAGMLAPGLATMLVVLTTDVGLDPIGARRALRSATAQTFDHLDSDGCMSTNDQVTLLSSGMSAAQPSEEEFTAALTEVCRDLALQLLDDAEGASHNITIRVVGAVSESDALEVGRSVARNNLFKAAIFGNDPNWGRVLAAIGTTRAAFDPYEVDVWMNGVRVCHHGGPDQPRDQVDLSGRNTEVLIDLQTGAAEGTIWTNDLTHDYVHENSAYSS
gnl|extdb|pgaptmp_001113	K00930	argB; acetylglutamate kinase [EC:2.7.2.8]	MTKVSYRFRAPEEARRFANTLVQSLPWLKEHHQKIVVIKFGGNAMVSEELQRAFAEDIAYLRYAGVQPVVVHGGGPQINQMLERLGVRSEFAGGYRVTNRETMAVVRMVLQGQVNPELVSLINSHGPLAIGLSGDDAAIFNARKMVLKEDGRELDLGLVGEVVDVDPEPVLGQLAAGRVPVISSIAPNVELPGEVLNVNADLAAAALATALGATKLVLLTDVEGLYENWPNKDSLIASITADELRARLDSLEAGMIPKMRACLDAVDGGVGTATVIDGRVPHSVLVELFTDSGIGTEVRSDIDKC
gnl|extdb|pgaptmp_001114	K00821	argD; acetylornithine/N-succinyldiaminopimelate aminotransferase [EC:2.6.1.11 2.6.1.17]	MTSVERGWAERSADVLIQNLSVPLAQMERGQGCYLWDNAGKQYLDFLGGIAVNSLGHCHPAVVQALTEQASRLDHISNYFVSRPQLDLAERLLDLAGATGGGVFLANSGTEANEAALKLARLHGNSQGKSRIIAFEGAFHGRTMGALALTAKAKYRDPFAPLTPGIEHLPVDEAALREAMDDQVAAIFVEPIQGEAGVVELPAGFLEAARELADRHGSLLILDEVQTGVGRTGRWFGFQHTSVVPDAITVAKGLGSGFPIGALVAFPSCSGLFYPGSHGTTFGGNPLAARVALQVLQVLEDAAVLENVQVQSERLRTGLAELGSDLIASVRGRGLLLGIQLTRPVAAEVTAAAFARGLIINAPAPDVIRLAPPLVVGRREVGEFLELFAQSLDAS

<table>
<tr><td>gnl|extdb|pgaptmp_000282</td><td></td><td></td><td>MTASTKPVDASATKRAGGAARIIGIILIVAGAIMLVSGGVAWGAVSSQLKVQQMVVPDDAPSNAGKAVAGPFTAWSMQEIINIHAEHSTEGETYATLGDK
VNEAKAEFGDDSEEAAKLQALRNTAQSAAFLRASLFTSILAFGVSALVMGLGVTTAITGAGFVVSGKKD</td></tr>
<tr><td>gnl|extdb|pgaptmp_000112</td><td></td><td></td><td>MTQPATPASVPTAPLSRLWGIDVARFCAIVGMMAAHLLGWGEYTPWLEAITSGFPSTLFAVLGGFGLVFASRRYASAPSSWPGIAAGIARGVVVLVLGLAL
EWLPQHPIAIILVYYGTAMILVAPLIYLRARWLLLLATALALLGPQLLGALPHDGYLELGNPVAAAQSVLFAGMYPALTWVAYLLIGVLLCRWFLAEREAGRSE
RAGAWLFIGGLGAGAVGWLAGWLYQEFWWEPPWDSGAGAPVSAGWNALLQLSTHTGSTVDLVRTAGLSVALIGLCLLATARSTAVPLGWRPIVGMGA
APLTAYTLHLLMTSFGLFFAGGMSVLLEPERVGWFSIAFVVQLLVLLVGGAYLFSRQRRGPLELLVSRVAQTAGELAWGRWEPRR</td></tr>
<tr><td>gnl|extdb|pgaptmp_000906</td><td></td><td></td><td>MKTKLTPAGLAGVLLAGALALSGCSAAAMNGAGPVLAPMATRDLGVSGSEAMFSADAAAASPSELGSKITVNGDAQVITGDPAGAAAKFTELVKGMDGTI
DNTWESDYDSGKSSSVSARVPADRYEELVAQLPDLGKVESQNTSSMDLTQQYIDVNARKQALEDSLRRVKALADEATSTAELLQAEDMIAQQQGELDSLN
QQLEWLDQQVDMSTLTVTFSSQAPGSNPGVSFGWIGDVLLKSLYTLAWAVVFLVPWAVIALVVWLVIRGLRRRGRKHRYPAPERAERQAEAEQPQPTEA</td></tr>
<tr><td>gnl|extdb|pgaptmp_001296</td><td></td><td></td><td>MPLNSIGDANNRRSQAWRVFFDASTRLQGILETKLKKAAGITLADYNILLTLYEAPGRSLRMGELADRTVFSPSRLTYLVSRLEKSGWVRKHPADGDGRGYV
AALTKTGVELTEASTAIHQETVRRLLLDDLTDSEIDRIVEAFERVDEQSRP</td></tr>
<tr><td>gnl|extdb|pgaptmp_001297</td><td></td><td></td><td>MEQTPAQPSPPRPGAYRVPKTPYRIGLEQETEASLRRPSLEVGPEPAPTAPVRVAPWLVKAGQSAWYTIGIMVIVVGIVFATIKITPVFMGVFAALVLKALLN
PLVDRLARFMKRWVAVIVSLLVFVALFVGMLTFVITSVAGQWSQLGSKLNHGVDMIIDFLQSLPFNINVTSDDIYQWIHDGVDMAQNYLSTNWQHLASEA
LSNAGSIVIGITVIFLAIFVAIFFLNSGSEMWRWFLNMLPEQSRAKTNTAAQAGWGAFSGYARGTVIIAVSDGALAWLFLEIVGIPLAPALGVLVLIGAFIPMV
GAPAAMLVAMIVALAVDGVWKAVIVGIGIALIGQFEGHVLQPLVMGRQVSLNPVVVIIGVISGTIVAGLVGAMIAVPVMGVAWAVFSSLYHRDPPIVGPLP
GKLPDSVLKEQKIQLPKLPVLDHEQFTRGRKTDPGN</td></tr>
<tr><td>gnl|extdb|pgaptmp_001298</td><td>K14162</td><td>dnaE2; error-prone DNA polymerase [EC:2.7.7.7]</td><td>MGAPYGELHAHSAYTFLEGTDLPETLIVQAHELGLGALGILDVDGMYSVVQTAQAAKTVGVPTVFGAELTVAATPDESGWAAPLGVASSAQVRLPVLAVN
QQGYHELCAAISAHNLNHPGQRKVPWELGELARFQRGNWRVLTGTAHGPLRQALGSGGTSAARAVLSRLQDLFGPDRVVVESCLHPGDPYNLGATLAEL
GREHRVEVVATGAVRCSSPRQQFLADVMTASRLNLELKAARPHLPPFGSFLRGEAEMKRIHRAYPRAVEAAGELAQEWAFDLTLMEPELPLCDIPPGHTND
TWLRELTYRGARQRYGSAEQDPAAWQTIEHELEIITELGFSGYFLIVKDIVDFCRQQDILAQGRGSAANSAVCFSLGITAVDAVRHHLLFERFLSPERTGPPDID
IDIESARREEVIQYVYSRYGRERAAQVANTISYRPRSAIRAAGKALGYSEEVLKGWSKELSRGWSGTGGQSSIPPLVRGIGASLQKLPRHLGIHPGGIVLTRTPV
SEICPIGWAAKENRTVLQWDKDDCADAGLVKFDLLGLGMLTALRKSFNWLKALGVVGTDRKALGLYNLPPEDQRVYDLLCAGESVGVFQVESRAQMNTLP
RLQPREFYDLVIEVALIRPGPIQGKAVNPFLRRRGGYEEATCHPLLAGILERTLGVPLFQEQLMQIAIVGAGFTAAEADGLRRALSSKRHVEKMEEIRPRLLAG
MEAHGLPSDLQEELYDSLRGFAEFGFPESHAFSFAYLVYASAWLKVFFPEHFYASILASQPMGFYSPASLIADARRHGVTCLGPSVTYSVEEASVQTADGQEL
TPDQIEVEFPADRLMRTHPNWVIRLGLDSINGLSGQIISRIVQARQAGPFHSLEDFARRTNARVSELELLAQAGAFSDLGVSRRRALWAAAQLANPHEEQPF
LPGTQLGQAAPMLPGMTEGEELETDYQVLKLSAGKHPMQLVRSELEGERVLPLAELADAEGERVVKIGGLITHRQRPGTAHGVTFLSLEDETGLANVVCTG
GFWKCYGQIIMTSRALVVTGRLEKKDGAISVRAQKVERLSVRVDVLSRDFR</td></tr>
<tr><td>gnl|extdb|pgaptmp_001299</td><td>K14161</td><td>imuB; protein ImuB</td><td>MFPTASDSRATGQRKIVLWVPNWSVAALASAVPAGAPAATVVGGRVHECTRAASRLGICPGMRQLTAQSICSELLLIPHDPVRDAAEFEGLLQVFDQLVAG
VCAIRPGLAWAPLPAEARWRKEEGELIQSLLEQCVSETGIEVYAGIGEGAAAAVGAARRGLILPAGQSSGFLRDLALIELVDYLPEEIQADYDEALNFLQLLGIR
RVSQLLELGRTQLVSRLGTVGEKLWSLAEGGDLFVRAQATAAPQIKARYEFTPGVGAVDHALLGVRRVAQEFVDLMAGKGCVAHTLIVRLELSTGLESTRR
WSLFDLTQSAQLSQRIIWQIRSWQDRMQQSGELGEEEVLLTAVSLEGTDVVTQALPQFLWGDKPQSDSVNRAVAQLQLLLGEESVRQPLLQGGMDPRER
VELVPWGAQVEPVPREGEWAGSVQESPLLLFDQPPLAKVLGQQSDGTWGQLWINRRGILTGIPARIAVLEERPELPSGGYGVGRIEGMWAVRGKWWAL
RQEADAARCYLRLQVENSVDFLLIQRGQEWRVEGLYLQQAQPAPNFPLAER</td></tr>
<tr><td>gnl|extdb|pgaptmp_001300</td><td></td><td></td><td>MEAADRLQAARQALQVGEGKTGLRSYQTRTLAPERPAGGVYRVEPNLPRLVLAIRSALTEDSWLALVGVANLGWEALAQAGIDLDRTVLVTCEPAQAAQV
LSTLLEGFSLVVVGDVTVSLSQQQVLAARARKLERILFTTSHWPVASTPWQRRSGANSWGRTGKVG</td></tr>
</table>

gnl|extdb|pgaptmp_001301	K03216	trmL, cspR; tRNA (cytidine/uridine-2'-O-)-methyltransferase [EC:2.1.1.207]	MLHMVFFEPKIPGNSGAAIRLAACTGAMLHLVEPLAFDMDDAKLRRAGLDYHDLAHVRVHPNLEEALEQIPGRIWAFTGHATRWHTEVEYRDGDGLLFGPEPTGLPEEVMAHPRVEDRIRIPMREGIRSLNLANSASIGLYEAWRQLGFPDGV
gnl|extdb|pgaptmp_001302			MSANDSTAARSVPGENTSGRGIRDDRRPSWGLGRIVMVLFWLFGLLTAVPALVALIRATDAPIGPRLIAVLAGLVYLVIAVGITHNGRKMRHIAWAATTVALVGPLITGLFELGTDHAAAVTSAWSRFGADYWYVPLALPLIGFVWLWRSDPRRIVELAEGIERPKRFPWHNG
gnl|extdb|pgaptmp_001303	K00382	DLD, lpd, pdhD; dihydrolipoyl dehydrogenase [EC:1.8.1.4]	MNDPDFDVVILGAGSGGYAAALRAGQLGLKVALIEGDKVGGTCLHRGCIPTKAYLHAAETADTVREAGTFGVEAALQGINMTQVGQYRDGVVTKLYKGVEGLLASRQVEIISGWGRVVSANTVEVNGRQITGKNLILATGSYSRTLPVLPFGGRVIASEQALAMDWVPNSALILGGGVIGVEFASIWRSFGAEVTIIEGLPHLVPNEDESISKILERSFRKRGIKFQTNTRFASYSQDESGVTVTTEDGKTYSGDVLLVAVGRGPVTEGLGFEEIGVKLDRGFVLTDERLRTGVDGVYAVGDIVPGLQLAHRGFLQGIFVAEEIAGLNPDPIDENSIPRVTFCEPEIASVGLTQRQAEEKFGADAVKTVEYNLAGNGKSNILGTTGIIKLVQQADGPIVGFHAIGARISEQIGEGELIVGWEAYPEDLARLIHAHPTQNEAIGEAAMALAGKPLHAHD
gnl|extdb|pgaptmp_001029	K03496	parA, soj; chromosome partitioning protein	MIYSRRVDEDLQPSLVSLEEDDQFPLPEALAAHGPARVIAMCNQKGGVGKTTTAINLGAALALYGRRVLIVDFDPQGAASVGLGINALELDTTIYTLLTNPKADVHQAVRPTRVANLDVLPANIDLSAAEVQLVNEVARESALARVLRPLMDEYDVIIIDCQPSLGLLTVNALTAAHGVIVPVETEFFALRGVALLVETIETVRDRINPRLKIDGIVPTMVDTRTLHSREVLERLEETFGDLVYDTRIHRTVKFPDASVATEPITTHAPSHSGAKAYKRLAREVIFRGEAP
gnl|extdb|pgaptmp_001026	K03075	secG; preprotein translocase subunit SecG	MIVVRIILVVLIVITGLLLIGAVLMHKGKGGGLSDMFGGGITSSAGSSGVAEKNLNRITTGLLVVWSVSIVAYGVLIRFWS
gnl|extdb|pgaptmp_001024	K00927	PGK, pgk; phosphoglycerate kinase [EC:2.7.2.3]	MLRTIDSLGELAGKKVLVRSDFNVPLKEGQITDDGRIRAALPTLRRLLDGGAAVIVMAHLGRPGGQVDPKFSLRPVADRLGELLSQTVELAEDVTGPSAQALAQALQPGQVLLLENVRYDGRETSKVDEERVALAREYAALADLYVSDGFGVVHRKQASVYDIAQLLPSAAGELVFKEIDSLSKVTDNPARPYVVVLGGSKVSDKLGVISNLLHKADALLIGGGMAFTFLAAEGYGVGKSLLESDQLETVKNYLAEAKELGVDLVLPVDVVVAPEFAADATPTVVAADQIPADQMGLDIGPKSQELFAKYVLDAKTVAWNGPMGVFEFAAFAGGTKAIAQAMEDSAAFTVVGGGDSAAAVRLLGFDEAKFSHISTGGGASLELLEGKTLPGIAVLED
gnl|extdb|pgaptmp_001021			MSLLDNQGFPIRGNSGQRVVAFGGGHGLSATLKALRHLTHQLTAVVTVADDGGSSGRLREEMEILPPGDLRMALASLCHESEWGLTWRDLMQHRFETDGPLNGHALGNLLITGLWQMFDDPVEGLDWVARLLQAHGRVLPMSLDPLQIEATIEVEGRLEVVRGQTEVATVGHPISEVRLIPDHPRVPKEVIRATQEAEWVVLGPGSWYSSVLPHLLVSDLHRALVTTEAHRALIMNLARQEGETELLSTADHIRVLYQMAPDFKLDVVIADPTAVDDVDDLVEAAERVGARLLLRQVRSGSGAPIHDPLRLAAALRDAFEGYLGEVGKTENWLA
gnl|extdb|pgaptmp_001273			MALLFDLDGTLVETEPLWQRVQQQIVESLGGEWSLALNQRLIGNGLMTGSQILREATGTSVSVETIAQLVLDRMAEALAAGEALARPGADAAFVLARRLGLATAVVTSSYRVLAEPALRALPDAHPDALVTGEMVTRAKPDPEGYLLAARTLGVPIEQCVVVEDSAIGVEAALASGAHAVAVPNHTPIPTDPRLTVLGSLEDLTEDFLAQLLGPIWLTRAKSSSAAGGI
gnl|extdb|pgaptmp_000215			MTTLPPPADQAERLIELGVPALLGQNPDAVREAATALGSPAPGLLVTEAARTHLDELVPLICREGQRGFIVEDFVDTADFVPVSGLDPSPITLPDGWYWLEDPRRGDEFENASPAEAWEQIVAANRVPLTAAEGVFWLLQQPDTLERNHCFMTVGSRKPRPRGGYDSRTPALWISNGTGRDGSDRKNAPKLGWCWWNNRHTWLGIAHAAARHPAQP
gnl|extdb|pgaptmp_001488			MSLNEVTAVRSGVRSALAIGGLISIVIGIVILVWPGKTAMVVAAILGLYTVVAGLSYIGVGLSSLDKTGWGRLGHILLGALYIIAGVVFFTNLQSTTAILVILITIMIGAVWIVEGIMALTTLSHTKSKGWSIFYAIISIIAGLILLFSPMASAVMLWFIIGISAIVMGVVQLVRSFTV
gnl|extdb|pgaptmp_001489	K02911	RP-L32, MRPL32, rpmF; large subunit ribosomal protein L32	MAVPKRKMSRSNTRTRRSAWKAKLTTLENINVSGREVRIPRRLAKAYQRGLVEIED

gnl|extdb|pgaptmp_001490			MRTLRSGSSTTVEVEIKRSRFIATVARTNSAQEARQLIDLARSTFPDARHHCSAYVVEADRVNPLQHFSDDGEPAGTAGKPMLDVLTHAELGNVTAVVTRYFGGTLLGTGGLVRAYSGAVQEAVAAASIVEVHTLARFRTELNPAVGGRIEAQLRDAGWQVLDSVWGQTLRLDVAAPPEALARLNSELSAWLQSPSAFQQVGTIDCEVDSDAAEQ
gnl|extdb|pgaptmp_001491	K01738	cysK; cysteine synthase [EC:2.5.1.47]	MTNIYPAIDATIGNTPLVRLSSLAPEGVTILAKVEAFNPGGSVKDRIGKYIIAAAEKSGALPPGGTIVEATSGNTGIALALIGAARGYRVVIAMPASMSKERRSLLRALGAELVLTEPSEGMRGAVAAAEKIVAENEGAILASQFTNPANREAHYETTGPEIWRDTDGQVDYLISGIGTGGTISGAGAYLKEQGEVTLVAVEPAESPLLTSGQAGPHLIQGLGANFIPEVLDREIVDEVIDVRGEAALETARRAAREEGLLVGISSGAALAAALEVAARPEAQGKTIVAVLPDTGERYLSTALFAEPEE
gnl|extdb|pgaptmp_001492	K00640	cysE; serine O-acetyltransferase [EC:2.3.1.30]	MSLFSFLTVLREDLRTAIAQDPAANSPWEVAASYPGLHAIWGYRLGHALWNRGLRAPARILQNLIRFATGVDIHPGAKLGRRLFIDHATGVVIGQTAQVGEDVLIFHGVTLGGVSMSPGKRHPTVGNGVVIGAGAKVLGPITIGDYSRIGANAVVISDVPEDSVAVGVPAKVRCPLPKTYRASLMQEPELYI
gnl|extdb|pgaptmp_001493			MARITLQADLQRLEAPRRPGELELATLTMYDVPAMASLKLVAYDEPLTFESLLESSDEMRMAFEGAFGTPRDDSFIGAWLGGQLVGAVMAVLDPPWDDAPRGPFVTELMVDPEYRRQGVATALVGELAARAAEWGYDSLTLRLDLRQSPGAYGLYQDLGFSVITEED
gnl|extdb|pgaptmp_001494	K05522	nei; endonuclease VIII [EC:3.2.2.- 4.2.99.18]	MPEGHAIRRLALAFDASFVGEQCELSSPQGRFAAGAAQLDRWWMSAAQTKGKHLFLGFGPEPGETEEWIHVHLGLYGGWRFAGKSEVGTALSPQRLDPDPGILPATPSEHWPPEPRGAVRLRILTAESLADLIGPNRCQLVTAEEMAGIEARLGPDPLADDALSEPVRQDFVARVRKSSRPVGELVMDQSVSAGVGNIYRAEALFRTGINPYRAGTRVSAARLGALWDDFVVLMRRGVIEGAINTVEPDEAQDWDPEAERWYVYHRTGRPCLRCGTSIRQAELKGRALFWCPQCQR
gnl|extdb|pgaptmp_001495	K01869	LARS, leuS; leucyl-tRNA synthetase [EC:6.1.1.4]	MVDDSASEPQFRYNAELAGQIELKWQQLWQERGSFQADNPVGNLAGPKAQAEPWFVMDMFPYPSGSGLHVGHPLGYIATDTMARYQRMRGKNVLYTLGFDAFGLPAEQYAIQTGQHPRITTEAAIANMSAQLKRIGLSHDPRRSFATIDDNYVRWTQWIFLQIFNSWFDPEAPNGEGSLGRARPISELIEQFESGQRELPDEVAAGRSWDQLSPAEQSGVLDRYRLAYISESPVNWAPGLGTVLANEEVTSEGRSERGNFPVFTRNLRQWNMRITAYSDRLASALDQIDWPDKVRSMQRHWIGKSTGATVKFQVEGRGELEVFTTRPDTLFGATFMVVAPEHPILIDELPGAWPEGTRPAWTGDHPSPAEAVRTYQAWTASRTERERQEDAGEKTGVFTGLFGTNPVNGKPVPIFIADYVLMGYGTGAIMAVPAHDERDYAFARRFDLDIIPTIEPAEGFNLDEAAWTGDGSLINSANDEISLNGLNKAEAVSAITAWLAEKAYGEATTTYRLRDWLFSRQRYWGEPFPIVYGEDGRVHALPEDQLPVTLPHLDDFSPRTFDPDDANSNPETPLGRAEDWVEVELDLGEGLQKYRRDTNTMPNWAGSCYYELRYVDPTDPDHLIDPANDEYWMGPRPTKPAGGVDLYVGGVEHAVLHLLYARFWQKVLFDLGYVSAAEPFHRLFNQGYIQAYAYTDARGVYVPAEEVEGDERSGFTYRGEPVTREYGKMGKSLKNSVTPDEMCASYGADTFRLYEMSMGPLDLSRPWDTRAVVGAQRFLQRLWRNAVDEETGELTVVDQPAGEETRRLVAKTVAEVTEEYNHLRINTAIARMIVLNNHLTQLESVPREALETLVLLVAPVAPHLAEELWSRLGHDQSLSDHPFPVVEDESLLVEDLVTCIVQVNGKLKAKLEVPTDIGEDELREQALGTEQIQKVLAGREPLRVIVRAPKLVNVVPPRD
gnl|extdb|pgaptmp_001496			MSTVFEMIIDGQIPSKFIWADDQCVAISTIEPIEAGHTLVIPRQAIDKWTDLPPALLDHLFRVAQIIGRAQEQAFDVPRSAVIIAGFEVPHMHIHVVPAHTEAAVQLPRARAASDEELTVAAQSLREVLRQSGYEQHVPLELGSPRVS
gnl|extdb|pgaptmp_001498			MTPSKTAPQLSRPVAIALLLFAVIGLGASFLLLKSELELLADPAANLGCDINPLIACSDSLMTPQAHLLGVPNSMIGMMAFSALVALAVTLAAGVRLPRWIWGGLGVGTLVAMVYVLYFLLQSLLTFRALCPYCMIVWAATIGIFTIVWAALFAGGMFGGRGVALGRSVLRFWPLVVLAMYLLVVLAIVLSLPDKIGYLL
gnl|extdb|pgaptmp_000557	K02529	galR; LacI family transcriptional regulator, galactose operon repressor	MADRQRSVSTKDVAALAGVSIGTVSNVLNAPHKVAEATRLRVNGAIRELGWERNENARQLRAGRSNTIGMLVPNLYNPHFAELFHGAEEFLYERGFIVNVSNANELPEREELILDQFRRQRVGGVMMAPVGAAMASAANLMERQIPVVLLDEANNSEFSGVGSDNFTGGLLAGHHLIDQGHHRIAFVGASERLVQVRDRLRGCQVAVQAHPGASLRVISTAQMDPEAGRRAAEEVLKLDLPFRPTAVFCASDLVAMGFLQGMTAAGLRVPEQMAIIGYDDIDFAASAAVPLSSIRQPTHQMGREAASLLLGAMNAISQTGEMPRISKLFNPDLVVRESTLRS
gnl|extdb|pgaptmp_001980			MKKTSKMTAILSGAVLVGAAGLAVSVPALAAPGDGGTGSLTVHKFEQPGSGDFGPADGTQITPPQGALPVGDVGFTVCSVTGIDLAVPSDWDRLKDLVGTVDNNGDLVVTEGGSPVAVSCQAEVRTDVDGEAAFASLPADRAYFVYESTPADNSLYAMIPTLVTVPFPGNGTGAAWNYDVHIYPKNALVGSGATKNGEIIGSDVTFDITVPINNLGLDDNGNQVLYTQFVITDQLSSALTYGSSSVQLLDSNGDEVTMTAGTDYTLAESAGLVTLTMVDPAGLALLDANIGGKLVLTITADANGTGDTSNTATITINGKGNDVSVVDPEEFYSGAYIMKEASNRGATANVPLAGAGFELYTKAGLTSCPAYDQLAADAEMKQVDLGSNYVSGTDGKTPELVLAKGSYCVYETVVPAGYKGAIGGALLDVQAEGANLTVVNTQVGSDEGDLPSLPVTGAQGRVLLAIAGVGFLTIAMGLYLVRRNRQRETESQQ

gnl|extdb|pgaptmp_000580	K23188	fepC, fagC, cchE, desF; iron-siderophore transport system ATP-binding protein [EC:7.2.2.17 7.2.2.-]	MSASRLGARDLATGYGAREVISHLDFAVPDGSFTIIIGPNACGKSTLLRALAHLLPARGGQVLLDGRDVARLPAKELARQVGILPQSPLAPEGITVRRLVSFGRHPYRGLLGGWSGQDQAAVDEALARTHLTELSDRLVDELSGGQRQRVWIALALAQETDLLLLDEPTTYLDLAHQIDILDLCAQLNQAGRTLVAVLHDLNQAVRYATHLVAMRDGKIVAEGRPEEIVNQDLIRDVFGLDTTIIPDPETGRPLVIPCAPPAQQLRR
gnl|extdb|pgaptmp_001472	K13288	orn, REX2, REXO2; oligoribonuclease [EC:3.1.-.-]	MSETVLKSPLVWIDCEMTGLDPTHDELVEVAVLITNAHLKVVGPGLDLVIRPSEGALNQMDDFVREMHTKSGLIDEFAQGLELSEAETQVLDYIRRYVPEPRTAQLAGNSVGQDRIFLSAYMPRVVEHLHYRIIDVSTIKELAKRWYPRVYACAPKKDGDHRALADIKESIQELEYYRRALFPAQLDPRSGAYVRIAESVADLPRVDEEPAAH
gnl|extdb|pgaptmp_001471	K03111	ssb; single-strand DNA-binding protein	MENNRITLTGWLGTDVTLYAEGDDRVPMAQFRMVTPKGRFDDRGSWVEAEGTWYTVKAFGDLALNVNNSLRKGHPVVLVGKYVARGWMAKDGSLATELQIHAFTVGHDLRRGVSSYAKLIRTEPERPSEAEEAGEQGAAVEPESSAEDTGTEEAEGYESGTVPF
gnl|extdb|pgaptmp_001465			MAEYIYSMVRARKAHGDKVILDDVTMAFLPGAKIGMVGPNGAGKSSILKIMAGLDEASNGEARLTPGYSVGILEQEPKLDEDKTVLENVQQGVGEILGKLHRYNEISEEMANPDADFDALLDEMGKLQTEIDAAGAWDIDSQLDQAMDALRCPPPETPVKVLSGGERRRVALCKLLLEAPDLLLLDEPTNHLDAESVLWLEQHLTQYPGAIIAITHDRYFLDHVAEWIAEVDRGRLYAYEGNYTTYLEEKQKRLQVQGKKDAKLAKRLKDELDWVRTNPKGRQAKSKARLERYEEMAAEAERTRKLDFEEIQIPPGPRLGSVVIEAKNLQKGFGDRSLINGLSFSLPPNGIVGVIGPNGVGKTTLFKTIVGLEPLDGGELTIGETVKISYVDQNRAGIDPEKTLWEVVSDGLDYIQVGNVEVPSRAYVSAFGFKGADQQKPAGVLSGGERNRLNLALTLKQGGNLLLLDEPTNDLDVETLGSLEKALEQFPGCAVVVTHDRWFLDRVATHILAWEGTEEEPDKWYWFEGNFESYEKNKIERMGAEAARPHRVTHRKLTRG
gnl|extdb|pgaptmp_001464	K10805	tesB; acyl-CoA thioesterase II [EC:3.1.2.-]	MTDPIQIPIIDAEPVASVLRTLTLQEAGPDFFAAESLPQVRRVYGGQVIAQALLAAAATVPDRTRQPHSLHAYFLRGGDPDRPFSLAVERLRDGRSFSSRHVSCRQDGAEILSLNCSFQGPEVGLDSAEVPPIVPGPEQLTSALEIFRSMNHPVAKFLGKTAAFDVRHVQRSLYTGADPARSNSQQLWMKPRNPLPEGMDQLIHRALLAYVVDQVMLEPALRATGLSWLTPGMSLASLDHAMWFHRDVDINEWLLFVGHAVSVGGGRAKTDIRIFNPAGQLVASAAQEGMIRVPTEETQGSGRWGFGTEQSDPPASTTRS
gnl|extdb|pgaptmp_001462	K00705	malQ; 4-alpha-glucanotransferase [EC:2.4.1.25]	MTLDAEGEERLRHLADLNGVSTGFWDWSGNYQQVAAHTLIAVLSELGVPVSDDSDRAQVEAAITWTEDQPWRQTLPACTVVREGSSQEIFVHVPHGRGVQVQVELENGDRVELAQLDWYFDPRSVDGELQGRATFQVPSGLPLGYHQLTATVHLDDELEVVTRPLYVVPNRLEPALLSGRNRYWGVNVQAYSVRSHRSWGLGDAADLADLTAICAQQQADFLLINPLHAAEVVAPVEDSPYLPISRRWLNLTYIRPEAIPEYVQLGPRQQAKIEQARLQAASLPAPRGGGLNRDGSWEAKKEALEQIHPLPRSLHREAQLGAFIEAGGPSLYRYALWCALVERVGSTDLGEEFSSPDSPAVHALEEELAPRIHFYQWCQWVAAEQCQEPNRVAHSLGMRVGIMADLAVGVHRFGADYWANPEVFASTMSVGAPPDMYSQQGQDWSQPPLNPRALERVGYQPLREIFAATMRLSGALRIDHILGLFRLWWIPCGERADHGAYVYFDHEAMVGILLLEAHRRGVMVIGEDLGTVEPWVRQYLNERGVLGTSVLWFEKDEHGWPLGADRYRENVLATVNTHDLPPTAGYMEGTHTRLRDRLGLLVEPVEQVMAQDLEEQDRMRQRLIEWGLLAEDAGPAEMLEAMHRYIARTPARLVAASLVDAVGEKQPQNLPGTHNEYPNWRIPLSDAEGTPIWLE
gnl|extdb|pgaptmp_001460	K02803	nagEb; N-acetylglucosamine PTS system EIIB component [EC:2.7.1.193]	MSKPEQIIEGLGGPTNIKILEPCITRLRVEVNDPSQVNEAKLKEAGAYGVVQVEGTVQVVVGPEADHLAREANSLL

gnl|extdb|pgaptmp_001459	K01256	pepN; aminopeptidase N [EC:3.4.11.2]	MPGLNLTHEEAQQRAQVVKPHSYEVLLDLTTGDKTFASQTKIAFSATEGASTFADLVAAQVHQIRLNGQEVPTEHFADNRIWLDNLAAENELVVQADCLY<br>MHTGEGLHSFVDPADGQRYCYSQFEVPDARRVFTTFEQPDLKATFQFSVITPDGWTVLSNSPTPAPTTVEQGLRFDFEPTPLISTYITAIIAGPYVGETGSLTSV<br>DGREIPLGVYCRQSLKEYLDADVIMDTTRQGFKFFEEAYGIPYPFAKYDQIFVPEYNAGAMENAGCITFRDQYIFRSKPTEWELESRANTILHELAHMWFGDL<br>VTMKWWNDLWLNESFAEFMSHLALAEGTEWTDAWIGFMSRKEWGLNQDQLPSTHPIKADMPDLQAVEVNFDGITYAKGASVLRQLVTYVGRDNFFRG<br>LHEYLTKHSWDNATLDDLLSELEVTSGRDLRAWAQVWLEEAGVTLLRPVIEVDDQGNYARVSITQEAFTPGASLRPHRLAVAGYDLNEDGNVTQSFRVEVD<br>VDGAETVIDELAGRPRPALLLINDGDLAYAKIRLDEDSLQFAVNQIDKVSDPVTRRTLLSAAWDMTRDGDMSATDFITLSLRAAPVETNIASLTSNLARIHTAV<br>THYTAPANRGARLESTGDRLLLLVKANPAGTDQQKLLVKSLAQNAVSDQHFEFLTGLFEGNHPLHGLDVDIDLKWTLLTALVRAGRAGEAEIAALSEEDPTLT<br>GAQNAARARATVNSDEVRAQTWEEALTNSEIPNDTLWAMLAGFWAHAATNPAAYASYVEKYFDSVVRVWDSHTFHIGSRLIEATFPSPLSGYLPEVNVVE<br>RAEQWLANHQEQPASLRRLVLEGQADAQRMLTAQRCDAEGPRQ
gnl|extdb|pgaptmp_001458	K07386	pepO; putative endopeptidase [EC:3.4.24.-]	MTNNLKAHVLEEAAMDPTVRPQDDYYRYINGTWLENHEIPADRASDGSFFALRDLSEVNCHRIVKDAVAGKIDDPDAERIAILFQQFMDEEEANRLGAEPL<br>VPYLTQIFSAADHEQLAGVLGELFAMGLGGHFDLGVSPDINDSSRYILYVNQAGLGLPDESYYREEQYASVREAYRAYLQQIFTLAEVSENPAAAADAVFEFE<br>TKLAAHHWDVVRDREVEKQNNLRSWEELKAENPGFAWDRWARDLQLNLAEVPAFNVNQLDFLEAASQLWEQTDLHVLKLWLSRKIIAFASPYLSDLFVLE<br>NFNFYSKTLAGIQEMRPRWKRGVALVEGLVGEALGRLYVERHFPAEYKERMDELVAHLIEAYHSSISTLEWMSEATKAKALEKLAAFTPKIGYPSRWRDYRG<br>LELSSEQTLLANIVAATTFDTAWELSKLSRGVDREEWFMTPQTVNAYYNPLMNEIVFPAAILQPPFFSPEADDAVNYAAIGAVIGHEIGHGFDDQGSQFDAR<br>GEMQNWWTEEDREEFKKRTQALIAQYDAYHPADLGEEHHVNGALTIGENIGDLGGLTIAWKAWEKALAEQGLTPQTAPDIDGVSAIDRFFVSWARIWRG<br>KQRDEYAIQLLAVDPHSPAEFRCNGVLANFDEFADHFQVKQGDQLWIDRADRVSIW
gnl|extdb|pgaptmp_001455	K01870	IARS, ileS; isoleucyl-tRNA synthetase [EC:6.1.1.5]	MSQKHLPRHRRDQQVTAKPSLPDLEVETLKYWQDQDVFQKSIDQRPAGKKGSNEFVFYDGPPFANGLPHYGHLLTGYVKDAVGRYQTMLGHRVERRFG<br>WDTHGLPAELEAERELGIEDKSEIEGPGGMGIEAFNAACRRSVLKYTGEWENYVNRQARWVDFADDYKTLDPEYMESVLWAFRTLYDKGLIYEGYKVLPYC<br>WNDETPLSNHELRMDDDVYQERTDQTVTVGVALVDEAGRPTGERALLWTTTPWTLPSNLAVAVGPEIDYVEVVPSTGDLAGERVILAEARLADYAAELGE<br>DPAVARRLRGTDLVGRRYQPIFPYFAGKDPNAFQILGADFVTTEDGSGLVHLAPAFGEDDMAVCLAAGIKTVVPVDEAGKFTAEVPDYEGVQVFDSNRLVI<br>ADLREGTGALSEVADDRRAVLVKQASYAHSYPHCWRCRQPLIYKAVSSWFVEVTRFRDRMVELNQEINWVPNHIKDGQFGHWLAGARDWSISRNRFWG<br>APIPVWKSDDPRYPRVDVYGSYEQLAADFGVSADSIDLHRPYVDELVRPNPDDPTGKSMMRRVPEVLDCWFESGAMPFAQVHYPFENREWFEDHYPGD<br>FIVEYIGQTRGWFYLLHVLATALFDRPAFYNAVSHGIVLGDDGRKMSKSLRNYPDVNGVFDTYGSDAMRWFLLSSTVVRGGNLIVTEGGIRDAVRQILLPLW<br>NTYYFLALYANVCHGGEGIDLHRLGADDPVAELPEMERYLLARTHEVVLQMRASLDAYDIPSAAQTFRDHLDVVTNWYVRTQRQRFWDEEQDSFDTLYTV<br>LVTLTEAVAPLLPLLAEEIWRGLTGGESVHLVDFPTPPAQWADPELVETMDSVREVVSAAHSLRKANRIRVRQPLAAMAVVAANADHLARFAPLIGSEVNV<br>KEVRVEDPTHSGFEVRQELALNPRAFDPQLRPLTSKLFAAQKQGQWQAEADGSVTFPGVEFQGEPVRLSGDQVTLSTRVVAGGDEVATVLDSGCFVVLDT<br>AISEELAAEGYARDVIRAVQDARKNADLHIADRINLHLGVPAEFLAAARTHAELIAAETLATTVDLQEAPAAELVIDLSVVKD
gnl|extdb|pgaptmp_001454	K11754	folC; dihydrofolate synthase / folylpolyglutamate synthase [EC:6.3.2.12 6.3.2.17]	MSDNDDFPGIDPELVPYLIAAAEDDEAEDSAEPEAEESERLTALRNLVETSLLAGPDPDLIAELMGEIRAEDDDLFAEEESDGTALNPADAAAERAQVRALAS<br>ELFARAPEHDFAPTLDRIEQLMDLLGAPQNAYPSIHVAGTNGKTSTSRMIDALLGTFDLRVGRFTSPHLRDVRERITVEGEPLSPAQFLAAWEDIAPYVAMV<br>DEASTKAGGPRLSFFEVLTAMAFAAWADYPVDAAVVECGMGGTWDATNVIDSGIGVITAISLDHQQWLGDTIEQIAAEKAGIIKDKMTIVCQRQEPEALRII<br>EQRCRETDSVLRLEGRDWEVVARQSEGNGQILSLRTPTAVYADLYLPLHGEHQARNAGAALVAVEALMGTEPLRSDLVDTGLQAVRSPGRLEVLRGSPTVV<br>VDSAHNPGGAQALRGALEEVFDFGFAVGVYSAMRDKQVELVLAELEPVLDELVITQLGGPRAMPIEELEQIASDVFGPDRVHVRDELAEAIDRAAELVDLAK<br>NPSEDKGIVIFGSVVLAGDATNLLAPDRRLG

gnl|extdb|pgaptmp_001453	K05878	dhaK; phosphoenolpyruvate---glycerone phosphotransferase subunit DhaK [EC:2.7.1.121]	MKKLINDPHDVVKETLEGFQAAFPNLVKVHYEPDWVERAVAKGDGKVALVSGGGSGHEPLHAGYVGVGMLDAAVPGAVFTSPTPDPIVEATRAVDRGA GVVHIVKNYTGDVLNFETAAELADMDDIKVETVLVDDDCAVEDSLYTAGRRGVAGTVLVEKIAGAAAERGDDLATVAGIGRRVNENMRSMGLALGACTVP HAGKPSFELGEDEVELGIGIHGEPGYRRGKMEPADALAEELYTRVRDDLGLQARDKVIALVNGMGGTPESELFIVFRKVAELLDRDEIVLARPMVGNYVTSLE MPGCSVTLLRVDDEMLDLFDAPAHTPAWVVAEKA
gnl|extdb|pgaptmp_001452	K05879	dhaL; phosphoenolpyruvate---glycerone phosphotransferase subunit DhaL [EC:2.7.1.121]	MTKSVDWAREWIRRSAKAVSENREYLIELDRQIGDGDHGENLDRGFSAVLRGGGLEEAETGADVLKYVAKTLMSTVGGAAGPLYGTAFLRAAQKFPAEELT SADVAELLAAALGGVQARGKAVAGEKTMVDVLIPASEVAAAAAEAGATVPETWQQVAERAAVAAEETIPLKATKGRASYLGERSIGHQDPGATSTSLVLQA GADAAAEVFGGSGGE
gnl|extdb|pgaptmp_001451	K05881	dhaM; phosphoenolpyruvate---glycerone phosphotransferase subunit DhaM [EC:2.7.1.121]	MSEGARISLVIVSHSRKLAEGVRELAQQMAPSVQIEAAGGREDGGIGTSFDLVSSACERALSASGERGVVVLTDLGSANMVAESVIDFSAHPERIILVDAPLVE AAVVGAVASQQGEDLSGVILAIKQGAAQALEEAPEVGSADPLEGDAVTQTATVGDKAGLHARPAAEFVKMASVFDAQIRIDGADAKSLMEVMALGRRQG EQVVISATGPQATEAVANLSSALTAGFDK
gnl|extdb|pgaptmp_001450	K00940	ndk, NME; nucleoside-diphosphate kinase [EC:2.7.4.6]	MPTPDLLDPEKEHTLILIKPDGFRRGLTGEVLRRIEAKGYVLKGLKIKRADEELLREHYFEHLERPFFADLVEYMSSGPVVAVVAEGDRVIEGMRNLMGATNPT LAAPGTIRGDLGRDWGTGNIENIVHGSDSPTSAEREIALWFPELQYHD
gnl|extdb|pgaptmp_001447	K03529	smc; chromosome segregation protein	MHLKTLTIRGFKSFATATTLRLEPGITCIVGPNGSGKSNVVDALTWVMGEQGAKSLRGANMADVIFAGTAARPALGRAQVELTIDNSDGRLPIDYAEVTIAR TLFRGGGSEYTINGASARLLDVQELLSDTGMGKQMHVIVGQGQLDAVLRATPEERREFIDEAAGVLKHRRRKERALRKLGAMDADLLRVLDLTEEIRRQLRP LARQAKAARAAAGIQDRIQRAQLHLLVDDLLQTGRRLEVDQQQVRRLRADEAAREEAVHRLDRDLREVEEAANELGRQLEQATELAHQFDSLQERLHSILT LTQERLAQLGTPAAAVSEAAVALAEEKAAEAERARGEQAEVVERARQAVAAAESERREVESQQAELAREIRQAEQQAAQRQAALQRVREREIQTRSRVAA LQEALDAARLRFDQARERLVQAQTQLKAATPGAPDQEQLPRVRAELTEADLGVEAARAQLEQAQDQQRGAQAELARWQARREALGQTVESSARAQAHE AYPLLLPAGSEQPARMDQRLRVSRGWEKALSALLSQWEQAAVIAPSPQPAQVVEAVGAASAVAAVFADSAPAQPESAPPRDADQEWSALEVVRADQLLA PALAELLADAWVCATVAQATELLAANPQAQRVATADGTVLTRHSVRGPDQSTPSLLELRSHWEDAGERAEAARRELDRATAAVASARTALTDATAAQAEL KTRVRAAEEEQARLARAHSQLEAQVEAAAEAHTRSEAEVSAAGERLGVATEELERAQADAARAAEPEVPDPLPDLRSQADALTRAQQQAAAGELEAKLAF GTAQEQLRSAQRQADAFRSRWEQLQVDRAQGLEQEERRQRRAQRLERLRQRSHEARERARIHARQAADGREQLSQRVATLRSQREDLAERLERERWEQ VAVSEERQQAEVAIAGTKLQHDRAREELLAQVELDPEHPDPDSRLAELTETYGPHQPVVDAEGNSEPYVRERVSAELQAAQAELARLGVVNPLAVKEYEALE ARHQFLVDQVADLRKSKADLLAIIKEVDERVRLAFQEAFADTQARFAQVFATLFPGGKGELSLTDPDDPLTTGVEIYARPAGKRVTRLSLLSGGERSLAALAYL VAIFRARPSPFYVMDEVEAALDDLNLSRVLQVMEELRADSQLIMVTHQKRTMEIADALYGVTMREGMTKVVSHKL
gnl|extdb|pgaptmp_001445	K03110	ftsY; fused signal recognition particle receptor	MTSTPDLTTILLAVVAVIAVIVVAYLMVRAVRRRGQSGPAVPPTPTVSEAPETPEAPERGPEEARGPPETQLETPVEAPEVPELERPEPAATRMQRLRARLA RSGGIGNALLAVLSRGELKEADWEEIEDTLLVADVGLPAVEEIMAGLRTEMKVRGSVDPTEARQVLREQLLALVDPTMDRSLHLDRAGEDPAAILVVGVNG TGKTTTVGKMARLLRAEDKTVLLGAADTFRAAAAEQLTTWGDRVGVQVVRSDREGADPAAVAFEAVGRAKAEDLDVVLVDTAGRLQNKATLMDELGKV KRVMEKQAPVSEVLLVLDATTGQNGLRQAQVFAEAVGITGIVLTKLDGSAKGGIVISVQRELGVPVKLVGLGEGPDDLAPFDPDAFVDAILGQD

gnl|extdb|pgaptmp_001444	K03106	SRP54, ffh; signal recognition particle subunit SRP54 [EC:3.6.5.4]	MFNNLSDRLSASFKRLRGKGVLSEADVDQTVAEIRRALLDADVALPVVRDFTARVREQAYGAARSEALNPGQQVVRIVNDELIEMLGGQTRELHFAQRGPTVFMLAGLQGAGKTTLAGKLGKWLRESGKSVLLVASDLQRPNAVTQLQIVGERAGVQVWAPEPGNGVGDPVQVARSGVNHAIENGIDVVIVDTAGRLGVDEEMMDQARRIRDAVEPHEVMFVLDAMVGQDAVQTATAFRDGVGFTGVVISKLDGDARGGAALSVRGVTGAPILFASTGEGLDDFERFHADRMAGRILDMGDILTLIEEAERKFDQAETEKMAQRAMSGELTLEDFLTQLQQMRKLGSMKKVLGMLPGMGQMRDQLENFDERDVSRVEAIVRSMTPAERADVKLLNGSRRQRIAAGSGTTVTEVNQLVKRFEGAKQMMKQMGSGGAPGMPNLQGLAGMGGPGKKTRARQQAQQAAARKKKIAKKARSGNPAKRRQQELEAMLPQDQRPETAAQPGAAFGLTQPAPAARPTMDDLPEDIRRLMGGR
gnl|extdb|pgaptmp_001443			MKPVTGWVYWRGQEAKYPSWQFGQLEADPKGGLKFTAEADRPGEHPRQWIIPGLVDAHNHLQIGGDGPVSEEVALDRAIQELSSGVLALRDLGNPHESPAHHLARAPRLVTAGRHLARPKRYLPGLALEVEDQADLPAAVAQQVQAGNPWVKLVGDWIDRSRGSEADLDPLWDRAVLIDAVQTAHELGARVAVHVFGRSALDDLFEAGVDSIEHGTGMTWEHCQEAAARGITVTPTLGQVELFPQFAGAATRYPNYAQTMINLYQNWRTWWANLVEARVQLLPGSDAGGYQPHGQLFRELYRWEGAGLDATTIIDYATWQARDFLGFDSLSTGAPADFLVLRDDPMVELTVLGEPLRIVFDGQDV
gnl|extdb|pgaptmp_000861	K08167	smvA, qacA, lfrA; MFS transporter, DHA2 family, multidrug resistance protein	MSRELSAARQWASLAVLVAGVLLLAIDATVLYLAVPALTADLAPTATQVLWIGDIYSLAVAGFLVTMGNLADRVGRKRLLLIGAAAFGFASLLAGFAPNPGVLIAARLILGIAGATIMPSTLSLIRVIFPDEGQRTKAVAIWSAASSGGIAIGPLVGGALLEHFWWGSVFLINLPVVVLLLIFGAWLLPESRNPQPARFDLLSSLLSLLAIVPFVFVIKQLAGGDFGPVTIGAFVAGVVGLVLFVTRQRRSKDPMIDVGLFANPAFTGAVLVNLISIFALAGVLFFFSQYLQLARGLSPLGAGIAQLPVALGAMSAFVFVGFLFRRLGRGRAIAAALLLGAAGLVGVSVAEHTQSLGWVLVALVPLGLGNGIAEALSIDAVVTAVRPEKAGSAASISETAYELGGALGIAILGSVVTVLYRAHLVLPAHLSPAAAEQTRDSLASAAQVLDPGSTAFAAAREAFVNAMETTTLIAAAIMVVAALVADLVIPAGKERNVVHA
gnl|extdb|pgaptmp_000814	K01092	E3.1.3.25, IMPA, suhB; myo-inositol-1(or 4)-monophosphatase [EC:3.1.3.25]	MASKVELAARLADLASAGALSVAPYLRRVARTISSYETKRDLHDPVTVHDRAVEADLRRFFGRAIPGSRLLGEEMGEEVLPLEPASTDPSQGGEATPADPELARLGARVRWLVDPIDGTANFASGSTYFGTSVAAELDGKVVAGAITIPYTGELFAADLERAWHVDREGRITELHSRGPRTENGALLVSYYPNRRALLVEPDLALRHLTDLTTNYMVVRRTGASALDLAMVAAGWEGALLGMSFGPWDVAAGIHLVKVAGGHVLNLSTGADLPDGLRPALLATVGTMEAPVAERVLREYVATLTTS
gnl|extdb|pgaptmp_000816	K06960	uncharacterized protein	MLADALEHLVLGIVDHPEDVRVSSRRQRRGQLLEVRVNPEDLGRVIGRNGRTARALRKVISALSTGGFVRVDVVDGH
gnl|extdb|pgaptmp_000817	K02860	rimM; 16S rRNA processing protein RimM	MTQEKLVAGIVGAPRGLRGEVSVILRTDLPEERFEPGTTLTTNRPEWPALTVESLGYHRDRAYLTFAEITTREEAEALKGAELLVDPDLEEEDAWYAHELIGLRVLDSEERELGVVTGLQVGLAQDLLEVKANGQTVLVPLVIELVPEVDPAAGIVRVTPPDGLFPGSTN
gnl|extdb|pgaptmp_000818	K00554	trmD; tRNA (guanine37-N1)-methyltransferase [EC:2.1.1.228]	MRFDLVTIFPEYFQVLDLSLLGRARQQGLVQVEVYQLRDWAEGKHRSVDDAPAGGGAGMVMRPDVWGRALDQLLAEPVAAPEAAPVAESATGPRRVLAIPSPAGEPLTQARLEDLTGADQVIVACGRYEGPDARIAEHYRSIGVEVLEFSLGDYVLNGGEVAALALVEGVGRLLDGVVGNPDSLVEESHAGAGLLEYPVYTQPRQWRGLEIPPVLASGNHAQIDRWRREQSLRRTAERRPELLSPEGLSTPDLEVVARAGYLLVPRRARVHLRPATGEDVPALVALARRTFPDACPPEVSAEDIAHFIDEEFTPARFTAHLANPDASVWVGEVEGELVAYTLCFRQAPADLRAAPPGAAYVSKCYADPSVRGSGLTGALLDEAVADLRARWGVDSVVLATHIGNRRASKFYRRHGFKKRGRRHFLVGQTDNIDDVFVLDLTRA
gnl|extdb|pgaptmp_000819	K02884	RP-L19, MRPL19, rplS; large subunit ribosomal protein L19	MQKLDSVDAASLRDDIPPFRPGDTVKVHVKVVEGNRSRIQIFQGVVLARSGQGVSETFIVRKVSFGVGVERTFPVHSPSIDQIEVVTRGDVRRAKLYYLRNLHGKAAKIKEKITR
gnl|extdb|pgaptmp_000820	K03100	lepB; signal peptidase I [EC:3.4.21.89]	MSFLSRRTAQHKAAESSEGSWWSWVREVLIVLILALVISTLLRHFVVQVYSIPSPSMVPTLQVGDRIVVDRIPGSGKDIHRGDVVVFEDSQGWMASADQGRTSFLRPIGEFLGLVPANGEQIIVKRVIGVGGDQVACCTAEGQLTVNGTPISEEYLPDGEVPSRDEFEVTVPEGHYWVMGDNRSHSADSLYHYQRGEQPFIADQDVIGRVWSVIWPVNHWSSVSHRDAFADVG
gnl|extdb|pgaptmp_000821	K03470	rnhB; ribonuclease HII [EC:3.1.26.4]	MLADLTREQILAEQYGLVAGVDEVGRGCLAGPVCVGLVVAPLDRPIPTGLTDSKLLSPTRRQKLVPQVEEWALAWALGWAGPDEVDHLGIVGALRAAGHRALAQIDLEVGIVLLDGNHDWFSDLWTPPVVTQVKADRDCASVAGASVLAKVARDRYMESLADPGYDWAHNKGYGSAKHRAALANLGVSDHHRKTWKLL

gnl|extdb|pgaptmp_000822			MGSDDIENYENSRELDLFREYKDVVGLFNYVVETERRFYLCNQVDMQARPAGGDVFFELTLTDAWVWDIYRPSRFVKSVRVVTFKDVNVEELSKPELGLP
gnl|extdb|pgaptmp_000823	K07460	yraN; putative endonuclease	MTKGTNQLGRRGEAAAERYLTARGLKVLQRNWRDGPRGEIDLILEDGATVVFVEVKTRRGGEVAEVLSPAKRFRLTCLAAAWLRQSPGFHPYRIDLVGVQPGPQGAQIHWWKGIDR
gnl|extdb|pgaptmp_000824	K07391	comM; magnesium chelatase family protein	MSTAYAISLTGLRGVPVRVEAQLGTGLVQTTIVGLADTALRESKERLRSALQSCQVPSLNRRLTINLSPASLPKTGSGFDLSIAAAVLSVRGLVDPELLPGTVFAAELGLDGTVRPIPGILAHAWSAQDQGFRRIVVAEESRAEADQVQGIEVIGCRHLSQLVQAFQPGGAGWDQFCPPPAVAADPVPGALADEDVDLNEVRGQPAARWAALVAAVGGHHLLLHGEAGSGKTLLAERIGTLLPPLDSHDALVLGAMRSATSSGGTLDRRAPNQIAGPNTTVPALLGGGPRLVRPGLISLAHGGVLVLNEAPEFPRRVLDALRGPLDNGEVTIRRTGGVVTFPAQFQLVLTANPCGCGPRCHCTPSQRHRYRQRLSGPLLDRIDIQWEMNQPTLTELERDQPISSAEAQQQVAAARARGRARWSTWPLNAHVPGRFLRTEGSIPDSFLRVLELTVSRGNLSLRGADRILRLAWSIADLAGHQRPTAEDLSSAMTLRLDHPWGRS
gnl|extdb|pgaptmp_000825	K04096	smf; DNA processing protein	MSSLPIDPADPVLAGAVWTHLAEPGDPAVRILQRVLGSEAALQWVFADEAGPVPEETIPWATCHERWHPRARALNLGQSLTELHRLGGRIIVPSSPDWPAQLGALEDREPQALWVLGAGNLGAHSVALVGARASSDYGNRVGATLARDLVSAGYPVISGGAYGIDAAAHWASVAAARPDGFAGVAVLCGGVGNRYPRSNEEMFQQLMKVGMLISEVPPHWRPARWRFLERNRIIAALSQVTVVVEAGVRSGALATANRALEQGRPVGAVPGPITSPTSSGCHQLLRDGAELIGSLDDVLNLLPGQGVGGESAGTTASPADLSPVARVVWDAFPRSGLAGVQQLGLAGGLGVSEVETALLEMQLRGLVGREGATWVRLEVQ
gnl|extdb|pgaptmp_000826	K03733	xerC; integrase/recombinase XerC	MLRWERYLRERQGFSAATVRSYRADVDNLLEFLGIRQLEELNDALTVRTLRAWLSHRVAEGKSRATIARNAAAVRSFTAWAHREGWLTVDVGQGLVTAGVENHLPQVLSQASVTQLLDYAAQHAETPIAVRDWAMTELLYSSGLRIAELCSLDVTSVQDPMIRVRGKGGKERVVPYGRPAARALDRWLERREELAAPGEVALFVGQRGGRLNQRVARAALHQLTQAAGVEDISPHALRHSSATHLLEEGADLRHVQEFLGHSSLQTTQRYTHVDAARLTKVFRQAHPRA
gnl|extdb|pgaptmp_000828	K02967	RP-S2, MRPS2, rpsB; small subunit ribosomal protein S2	MAVVTMRQLLESGVHFGHQTRRWNPKMKPYILTERNGIYIIDLQQTVADIDRAYDFVKTTVARGGSILFVGTKKQAQESVEEQARRVGMPFVNHRWLGGMLTNFNTVAKRIQRLKELEAIDFDDVASSGHTKKELLMMRREKDKLARTLGGIRDMNKLPAAVWVVDTNKEHLAVTEATKLSIPVIGILDTNCDPEEVTYGIPGNDDAIRAVDLLTRVVADACADGLLARSAPKKDSAEVADVEPLTEWERELLEKNSEEAAEAAPEAAPEAAAEEAKEEAAEA
gnl|extdb|pgaptmp_000829	K02357	tsf, TSFM; elongation factor Ts	MANFTAADVKALREETGAGMMDVKNALTEADGDKDKALEIIRLKGLKSLSKREGREALAGLIVAAVNGNVGTMVEVNSETDFVAKNQKFIDFAGQVLEAAVAANAGDLEALLAAPAGDGTVQDLVNQMGAVVGEKVEVRRVARVEGENVDLYLHQTNPDLPAQVGVFVVTDANGAEVAHDIAMHVAAFRPSWLDRDSIPSDILDKERDTLTKLTLQDGKPEAIVPKIVEGRLNAFYKDNCLVDQDFARDPSQTVGKVLKGKNAKVTEFVRFQVGA
gnl|extdb|pgaptmp_000830	K09903	pyrH; uridylate kinase [EC:2.7.4.22]	MSARRVLLKLSGEVFGGGKVGLDPNVVSQVAEQIADANRAGIQIAIVVGGGNFFRGAELSQHGLDRSRADYMGMLGTVLNALALQDFIQQAGTECRVQTAITMQQVAEPYIPLRAIRHLELGRVVIFGAGSGMPFFSTDTVAVQRALESHCDELLVGKNGVSGVYTADPRLDPEAKLLPKLTYAEALAQNLKVMDAAAFALCQENGLNTRIFGMAEPGNVARALKGETIGSLVTRD
gnl|extdb|pgaptmp_000831	K02838	frr, MRRF, RRF; ribosome recycling factor	MIDDILLDAEEKMEKAVEATAGDFANIRTGRANPGMFTELLVDYYGAPTPLQQLASVNIPEARTVLISPFDRSATNDIIAAIVAADLGVNPNDDGKVIRVTLPILTEERRREYVKQAKGRAEDGRVQIRGMRRKAKDLLDRLKKDGEAGEDEVDRGEKSLEALTKQYTDKIDRLLEAKEADLMEV
gnl|extdb|pgaptmp_000832	K00981	E2.7.7.41, CDS1, CDS2, cdsA; phosphatidate cytidylyltransferase [EC:2.7.7.41]	MLSLWQAPPTPNYQPLKPSGRAGRNMPAAVTSAVILLAALAIALLFARPVFVGFVALLVVVASWEVAGAFARKGLVVMLPPIYLGGIAMVLTGAFVSSFWVMASLSLTFCAIVIWRLASTKLESSAVMDILASVFVAVYIPFTASFVALISERSVSVWPVAFFVIIVVCNDLGGWMAGIMFGRHPMAPKLSPKKSWEGFAGSVILTTLAGVGSTFVLEIPWWWGILFGIFAAGLGTLGDLLESLIKREVGLKDMSAIVPGHGGLMDRLDSILFAAPAFYFLFALALGWMG
gnl|extdb|pgaptmp_000833	K06941	rlmN; 23S rRNA (adenine2503-C2)-methyltransferase [EC:2.1.1.192]	MNQLAREVRPTDQPPEGATDPQARPVLSFTAKRRGKPPVHLADLTGAERRDWFKEQGLPGFRADQLSRHYFGHNLTAKMTDVPVSLPKETFLPELITLAHEQQADGGETIKHLWSLYDHARVESVLMRYPGRTTLCVSSQAGCGMACPFCATGQMGLTRNLSAGEIIEQVRQARIACETGQLAGGPTHLTNVVFMGMGEPLANWKALKAALHRLIDPAPEGFNLSARNVTVSTVGLVPGIGKLADEGLPVTLAVSLHAPDDDLRDELIPINSRWKVGELLDAARTYFVRTGRRVSIEYALIKDMNDQQWRAQLLADELNRRGHGWAHVNPIPLNPTPGTIWTASTKAAQDQFVATLRAAGIRTSIRDTRGADIDGACGQLATSWAEKDKELK

gnl|extdb|pgaptmp_000834			MAEIFPKAGLLRRGYDRETVDQFFAQARAAYEGGIPAEQFSASQVRQASFELRRGGYDTRSVDSALNRLEAAFTQRDKVDYISVNGEANWYAKVAESATTLYPRLLRPDGARFSHPPRGEKGYRTDEVDALMHRITLFFDQNQPLTVGDVRLALFHSAKGEKAYREDQVDAYLGRVVEIILAAS
gnl|extdb|pgaptmp_000835	K00099	dxr; 1-deoxy-D-xylulose-5-phosphate reductoisomerase [EC:1.1.1.267]	MKLVILGSTGSIGTQALNVVRSHPDQFEVVALAAGGSQPELLAQQVREFSPERVALGTGELADLSAAFPQVEFSLGPEAVEELAGSYPEATVLNGITGGVGLGSTLAALAAGSTLALANKESLVVGGSLVRQAMRRPGQIIPVDSEHSAIAQALLAGVHHRGLVSPVVDGRSELSQIVLTASGGPFRGRSRAELTGVTPEQALAHPTWSMGPMVTINSSTLMNKGLELIEAALLFDVAPAQIVPVIHPQSIVHSMVTWEDGSTIAQAAPPNMEVPIALGLDWPHHHPGVGVPLTWDQAQSWTFEPIDPQVFPALDLAAQALAASATHPAVLNAANEVAVEAFLAGRLSWLSIVDAVRDAVEAHPGQSNPTRAEIEEIQAWAVARARAYVAAH
gnl|extdb|pgaptmp_000836			MFLLGILALIVGLIISVALHELGHLMPAKKFGALVPEYWVGFGPTLYQRRVGGTTYGIKAILLGGYVRILGMFPPGKTAGHRRTLVEEARLQSAEELAAARRQGLTGTPFYQLGTGQKLIIMLGGPVMNLLLSFILTGIVVTGIGWQQPTTTVSDVVSTTTELGLTGDSAPTPASSAGLQAGDQIVAWDGHPVEDWSQLQTLMETAPSQVTVDRAGQRLELDITPLATDQGPKIGIFPELERVHGTVGQAASAATGLLTGTVQALVTLPANLWELTTELATDAPRDPAGAVSVVGVARLAGEVSTVSSGLERVSMLLSLLASLNMALFVFNLIPLPPLDGGHVAGALWGGVRNLWARLRRQPRPAPVDTAKMVPLSYGVFLVLIVMSLILMAADIIKPLELF
gnl|extdb|pgaptmp_000839	K01881	PARS, proS; prolyl-tRNA synthetase [EC:6.1.1.15]	MLTSMSDLFVRTLREDPAEAELASHKLLLRAGYVRRVAPGIYTWLPLGLRVLRNVERIVREEMNRVGAQEVLFPALLPAEPYRATGRWDEYGPTLFRLQDRRQGDYLLAPTHEEMFTLAVAGMYNSYKDLPLTLYQIQTKYRDEARPRGGLLRGREFVMKDAYSFDLTDEGLDESYRKQRGAYQAIFERLGLDYVICSAMAGAMGGSKSEEFLHPSPVGEDTYVVSPGGYVANVEAVTTLSPADQSVEGLPEPRELPTPGAGTIEALVELANEQYPRLDRPWTAADTLKNVVFALTYPDDRRELLVVGLPGDREVDEKRLEVAVAPAEFEVATEADFAAHPELVQGYIGPTVLSPNREDGVRYLVDPRVVPGTSWITGANRVDHHVFGLVMGRDFAAEGTIEAAEVRVGDPAPDGSGPLELHRGIEIGHIFQLGKKYAEALDLKVLDQNGKAQVVTMGSYGIGVSRVMAALAESSCDDKGLAWSPVVAPFSVQVVVAGKGNELDAAALRLANSLDDAGLEVLLDDRTGISAGVKFADAELLGNPIILVVGRGLKNGLVELRDRRSGERREIPVETAVAETLALWDRLRGEG
gnl|extdb|pgaptmp_000840	K01462	PDF, def; peptide deformylase [EC:3.5.1.88]	MAIREIRVIGDPVLRTPCEWITDIDDRVKSLVEDLLETVDMDGRAGLAANQIGVGLRAFSWNIDGDIGYVLNPKLVATDTEHIQDNEEGCLSVPGLWYRTERSWYARVEGINLDGKPVVVEGEELMARCLQHETDHLEGMIYIDRLDRATRKQALRDIRQANF
gnl|extdb|pgaptmp_000841			MTRGFTRGVLFVHSAPPALGPHINWAVGNALSREVKLQWTDQAAAPGMLRAELSWVGPTGIGARLASALRGWEHLRYEVTEEATASTDGGRWAHTPELGIFHSQMDSVGNVVVPEDRIRAALEEATDFRSLQDALDLALGQAWDDELEPFRYAGAGAPVRWLHRVG
gnl|extdb|pgaptmp_000842			MGSIADLLGGELPDEWEQSDRVARQEGAREVALEAILTETNYPPEQMSPDLRLREELELDGLPLWAVVAQIERELKVTIPDREVKSWVTLADLLDAVERAG
gnl|extdb|pgaptmp_001733	K00140	mmsA, iolA, ALDH6A1; malonate-semialdehyde dehydrogenase (acetylating) / methylmalonate-semialdehyde dehydrogenase [EC:1.2.1.18 1.2.1.27]	MTSTELPVLNQWIDGQVSPGAPADSVAVENPGTGAAIATLNFTCAADLDRTVEAARRAQTQWARVSLARRTEVMFKMRELILANQDRIAQAIVAEHGKNYSDALGEIQRGRETLDFACGIASALKGEHTVDASTGIDIHTVRQPVGVVAGITPFNFPMMVPMWMHPIALATGNAFILKPPSTAPSASLIVAELYREAGLPDGLFNVVFGEKELVTGILEHPGIDAISFVGSTPVAKIIRDKGIAAGKRVQALGGANNHAIIMPDADLDFAAQHISAAAFGAAGERCMALPVVVAVGGVGPKLAEKVAAHARQIKVGYGMDEGVEMGAVITRAAKERIRGLIDDAQERGSQVVLDGRDFQVAGHEDGFFLAPTVLSGVPLDAPAYREEIFGPVLAVVEADSYEEAINLVNGSPFGNGAVIFTNDGGVARRFTLDVQAGMVGVNVPIPTPVAYYSFGGWKDSMMGDYDIHGPEGVRFYTRLKAITSRWPSEAGTYAATMSFQREE

gnl|extdb|pgaptmp_000852	K00163	aceE; pyruvate dehydrogenase E1 component [EC:1.2.4.1]	MTPSKDQRPVVNGILSQVPDNDPQETQDWLESLDALIDEKGGPRARYVLLHMLDEARRRDVQIPQEMTTPYINTIPVEQEPFFPGDEAIERKYRRWIRWNAAVMVTRAQRKGVKVGGHISSYASVATLYEVGLNHFFRGKDHPGGGDHIFFQGHASPGPYARAFLEGRLSEEEMDGFRQQVSVEGGLPSYPHPRHMPNFWEYPTVSLGLGPAEAIYQAWFDRYLHLRGIKDTSQQHTWAFLGDGEMDEPESRGMVQLAAQQGLDNLTFVVNCNLQRLDGPVRGNGKIMQELESFFKGSGWNVIKVVWGRGWDQLLAADKEHALVNVMNETLDGDYQTFKANDGAYVREHFFGRDPRTKAMVENWTDDQIWALTRGGHDYRKVFAAYKAAMEHTGQPTVILAHTIKGYALGSNFAGRNSTHQMKKLTLEDSKQLRDRLQIPITDEQLEADPECPPYYLPPEDDPALLYMKERRERLGGYLPERRANTPFLPELPGRPFDALLKGSGGQQVASTMAFVRVLKDLMRDKNAGKYFVPIIPDEARTFGLDAIFPTAKIFNTHGQNYTAVDADMMLSYKESIEGQILHTGITEAGSAAAMQVVGTAYATHNLPMVPIYIFYSMFGFQRTGDQFWAAADQLSRGFVVGATAGRTTLTGEGLQHLDGHSHILAATNHGFVTYDPAYAYEISHIFRDGLHRMYGEGDSRDPNVLYYLTVYNEPIHQPAQPENLDLEGLIKGIYRLDDHAGFGGPKVQLLASGVGVPWIREARRMLAEDWGVDAATWSVTSWNELRLDGMEADRHNFLHPEEEPRVPFVAKQLAGAEGPFVATSDWDNLVQDQIRPWVPGEYLTLGADGFGVSDTRRAARRFFHIDAESVVVRALQGLAGQGKIDPAVVKQAIDR
gnl|extdb|pgaptmp_000853			MAVSLGFAPGQIVQEFYVDDDVDDALRDSIIADTGHELVDLDYGDVVDGVIIWWRAEDAEEEDLDDVLVDALSNLDDGGGVIWVLSPKAGTAGSVPVADIEDSARSCGLQCTSATKVADGWAGIRLVARGRGK
gnl|extdb|pgaptmp_001473	K01754	E4.3.1.19, ilvA, tdcB; threonine dehydratase [EC:4.3.1.19]	MNAADVTRAEANLRPVTRLTPIEHSVRLSEVAGVPILLKREDLQVCRSFKVRGAYNRISQLDPSEREVGVVCASAGNHAQGVAFACQSLQIHGTIYLPVSTPRQKRDRIRALGGQWVELDFVDGAFDHAQQVALAHAEQSGRTYIHPYDDPAVMAGQGTVAIELFRQLEGGVETVLVPVGGGGLIAGMAAWLKEARPSIRIVGVEPAGAASARAALDHGSPKTLAGIDSFVDGTAVGRTGDRTFEVVRALVDDVVVVDEGAVCTEMLSLYHQDGIIAEPAGALATAAVCAAAAGRIGDLGLSGPTVAIISGGNNDLSRYAEVMERSMHYEGLRHYFLVTFPQQPGALRHFLDLVLGPEDDIVHFEYTKKNNRDLGPALVGIDLARPEDLGSLLARMEASPLHIEQI
gnl|extdb|pgaptmp_000837	K03526	gcpE, ispG; (E)-4-hydroxy-3-methylbut-2-enyl-diphosphate synthase [EC:1.17.7.1 1.17.7.3]	MPKVKDTPFPRRKTRLIHVGDVPVGGGSPISVQSMTTTKTHDIGATLQQIAELTAAGCDIVRVAVPTDKDAEALPIIARQSTIPVIADIHFQPRYVFAAIEAGCGAVRVNPGNIRKFDDQVAEITKAAKDHGTSLRIGVNAGSLDRRMYDKYGGATAEALAESAIWEASLFEEHDFHDFKISVKHHDPVTMIRAYEILADAGDWPLHLGVTEAGPAFQGTIKSATAFGALLREGIGDTIRVSLSAPPVEEVKVGLQILQSMGLRERTLEIVSCPSCGRAQVDVYTLAEEVTEGLKDLTVPLRVAVMGCVVNGPGEAREADLGVASGNGKGQIFIRGQVVETVPEDQIVETLIKHANLLAEEMGLGEGEVEVIA
gnl|extdb|pgaptmp_000089			MSFFDRFENAVEKGVNSVFSRVFKSGLKPVDVSSALQRAADDGLLDVAEGQGITANEYLVKVSPSDFTTLGEDGLGALSAELAEDLTNYVSKQGYALLGPISVSFEGSDQEFTGNLEVVAQRRRGPAAPAAGAVASPDHPIIDIDGEKWLLTEPVTVIGRGSEADIQVNDSGVSRKHVEFRITESGVILTDLGSTNGTFVEGHRVDAATLVDGNQIVIGRTPIYFWTHPEDVSAQ
gnl|extdb|pgaptmp_000090			MNSELAFTIFRLGFLVLLWLLVLGMVAVLRKDIYGTVVTARGAGRSQAQAGRRGRLKTEKVGGLAQPHNLLITGGPLTGTKIPLSGATISIGRAPSSTLVLEDPYTSSRHASIEENNGDWIISDLGSTNGTFVDDERLVGPRRLLVGETVRIGQTTFQLVK
gnl|extdb|pgaptmp_000091	K20074	prpC, phpP; PPM family protein phosphatase [EC:3.1.3.16]	MDVQFEFTARSDVGLLRENNQDSGYAGQHLLVLADGMGGPAGGDIASSVAVAALAPLDEDAVPFEQMLTLLRQGLQSAHDELIERSRVEPQLRGLGTTCIALLRSGNKLAMAHIGDSRAYLLRGESLTQVTTDHSFVQYLVDSGQISPEEAENHPKRSVILRVLGDSPGVVSADETMREAIVGDRWLLCSDGLSGVVSAETIAEVLRRVPDLDACADTLIELALLGGAPDNVTVVLADVVPSSVEVSDTPIVVGAAAVERSNPSRRIPGAAGRAAALISETEEPTPIAGGTEEGPHTPKKRHWWTAAAALIVVALIAGGLWWGWQWTRTQYFALGDNGRVVVFQGIPQKIGSLELATPVEVTSIRLEDLPTIDQQRLEDPVTRSSREDIDQYLAELRLRQKAAEPTTGTGQSQSGDDSKPTPSPTPTPSPSSSPKAAAPNEGGGA
gnl|extdb|pgaptmp_000092			MATVQVSPARPRRAVEMVLMLLALCVGVGGYVLTSLNRTGEFPPNLGLHVGLLVTLAVMAEIGVYFLAPYADPVILPAATALTGLGLAMIYRLDLSYAALGEATVGTRQLLFAGVALAVAALILVVVRDHRVLRRYTYTFGLISILLLLMPLIPGLGVEHYGARIWVRIFGFQFQPAEFVKVTLAIFFAGYLVTNRDKLSLGGPKILGLRLPRLRDLGPILVVWAIGIGILVFQRDLGTSLLFFGLFVTMLYVATNRVSWLVLGALLFIPAAIIIVKAMPHIQNRFDVWLNAFDPAIYSQTGGSYQVVQGIFGLANGGLLGTGWGRGYPQLVPFAQSDFILASLGEELGIVGMFAILLLYLILIQRGLRAALGTRDGFGKLLATGLSFSFALQVFVVLGGITRVIPLTGLTAPFLAQGGSSLIASWMIVALLLRISNAARRPAPTPTPWKYDSVPTTTGELPAVEAERPRSRWLARASRRAGGPTTAPISGPATPARPVSPDDQPTIGDIR

gnl|extdb|pgaptmp_000093	K05364	pbpA; penicillin-binding protein A	MNAQVRRIFAVALLMFLVLALSLTLIQVVNAPSLKADGRNSRQILQAAERERGAIIVEGNPIAYSERLDDGTERFRRVYPAGGTYAAVTGFFSAVNLYATGMEAAANEVLEGETSELFMQRLRNLFAGRPRQGGGVELTLSAALQQAAADALGDRAGAVVVEDVKTGKILALYSSPTFDPNPLASLDTAVALEADQNLQDDPSRPMNNRAIASDRYAPGSVFKILTATAMLESGLTPDTVVDSPPTMTLPGTETQLSNIEGSYCGSGQVSLREAFARSCNTTFALAVANLPAGKLQQVTKDYGFGAELEIPLEVTPSYFPDELNAAQLATSAIGQFEVAVTPLQMAMVTQAVANGGQMMQPYLVNRTLDADNRERSVTEPEVLATPISAEVAAQLTDMMKAVVSEPYGTGASMALDGVSVAAKTGTAEVGDGSYTNAWSVAFAPADNPQLAVSVIVEGDENNPTPHGGDVAGPIVRKLLEVGLQ
gnl|extdb|pgaptmp_000094	K12132	prkC, stkP; eukaryotic-like serine/threonine-protein kinase [EC:2.7.11.1]	MSAAENRTGRRLGGRYMLLTLIARGGMGEVWKARDTLTGALVAAKVLRPELTGEEVSLSRLRLEAKNAMRAKHPNIAAVLDSGEEDSQGWIIMELVEGQPLNEFMGDGLKLSPAELIPVLLQTAYALDAAARADVVHRDIKPANILVKADGRVKLTDFGVSLAQGQANLTAAGMVMGTAQYLPPEQALGKVATPAGDLYALGVIAFEALAGARPYTGDSQVDIAFAHVNEDIPPLPADVPVPLADLVTRLLSKDPDDRPNTGAALARELTRVAEEIGVGTAPVPLRVQQLPADTPLPTAPQGQPLPAAAQVPEPAGPSDRWLDFDLDPDGSPAAEAEPTVAPKVELSGPVAQAEAALASPAPSASSPSASSPSSNPPVPPVHHVRKQWLPVSPDAVPAASQRPRRPVAPSRAAAEGARASRSPAPTRSERPAADRTETGWGLWVIVGLTVLTVILIVVAMFRDHEVTGSEDTPAAAVTQQMQEVHTWLTPLPGV
gnl|extdb|pgaptmp_000095	K12132	prkC, stkP; eukaryotic-like serine/threonine-protein kinase [EC:2.7.11.1]	MANSPSRRLGGRYEVRSLIGRGGMAQVHLGFDTRLSRVVAIKMLRIDLARDTIFQTRFRREAQAAASLNHPNIVAVYDTGEEDVIGADGKPVSVPYIVMEYVEGHTVKDLLSDGTPVPLNEAVEITMGVLAALEYSHASGLVHRDIKPGNIMLTNDGSVKVMDFGIARAMTDSQATMTQTNAVVGTAQYLSPEQARGERVDERSDIYSAGVVLFELLTGRPPFTGDSAVAVAYQHVSEIPPLPSSIAGDVPESLDRVVLKAMAKNPADRYQTTTLMRVDLERAGKGLSVSAPQTSSWTAVTTPMSAAPAATAVYSGPLRSNGTPAAGNLALQNTTTNGEAIQDKPKRGRGVLIGFLVVLALALVGGVAYFLLREPPVDEVEQVAVPALVNKSASVAQTELEALGLKMTVGERVKDPEVEKDHVVSSNPPEGQLVDVGTTVTVTVSDGPGSATVPDMTGWTQAQAKAELERLGFKVGTVTTKDQANAEKDTLVSTDPPMGSTQPVGATINLVMASGNVEVPQGMEGADADSVADQLHSLGLNTSQKTAYSDLYPEGTVVSLTPSGLVPVGSTITITVSLGPEPIPDPTPTPTPTPTPTETPKNPDDG
gnl|extdb|pgaptmp_000099	K07284	srtA; sortase A [EC:3.4.22.70]	MTHQAPRQTRTRSQKMTAGQFIMVSIGELLIILGLLVGGYVVWQLWWTSVQVSGGVNESISTFQAEHPVQDTTTIAPERTDPPPAVAQPGYGETFGVLHVPKWNWMQIPITQGIGTDVLDLGHAGHYPDTQLPGEIGNFAVAGHRRTYGNNFRRVDILEPGDPLVVETADAYLVYEMTGNEIVDPSQWQVIAPVPNNPGEIPTQRLMTMTTCHPEFGNSERFIVYSELKYWTSKADGIPQVLADEPTR
gnl|extdb|pgaptmp_000101			MADKRNKKSGGSRGNDQDVSARWTDGIPLSPSWWAPAFITVLIVGLLYLVVFYLSSGRFPIPQIGNWNILVGVGIMLVGFGMTLRWR
gnl|extdb|pgaptmp_000103			MSDLPRFGQYAAVPRQEQCPRHPGETAVDYCKRCNRPTCAQCTIPTEVGSICVDCARPTNRRRAASLGLRSGPVVTYAIMAVTFAVYLIGQIWPTIDTYLAFNPVLAAVQPWRFLTVSLVHGGFLHILFNMMMLYFLGAGGEKILGHWRFLGLYTMSTLGGSVAVLAWALIQPQSLTVWTVGASGAIYGLFGAVLVQQLRNRINPTSILVLLAINLIYSFTAGGVSWQGHLGGLIAGALMAWIYAVLAQPRIGVTQRKQNIWEALATLGMVVVLGAISYGLYQPLIG
gnl|extdb|pgaptmp_000104	K03767	PPIA; peptidyl-prolyl cis-trans isomerase A (cyclophilin A) [EC:5.2.1.8]	MKATIHTNHGDINVELFPSEAPNTVKNFAGLATGERQWTDPATGQPTSKPLYNGVVFHRVIPGFMIQGGDPLGTGMGGPGYTFNDEISPNLQFNKPYLLAMANAGIRMGKGTNGSQFFITTDATSWLNGKHTIFGEVADDESKAVVDSISATPTNAQDRPLQDVVISSIDIVD
gnl|extdb|pgaptmp_001283	K03885	ndh; NADH:quinone reductase (non-electrogenic) [EC:1.6.5.9]	MSTQRPHVVIIGGGFGGLAAVRGLRRAPVDITLVDDHAANVFQPLLYQVATAALNPGDITWFLRSVRAKQPNVHFRRAAMTGLNPEAKVVSLSDGTELTYDYLVLALGVSANFFGIPGAEKHAIPLYQRAQALRIRDRLFGEMEWAATHPDDLRIVVVGGGATGVETAGALAEMRNLDLPVVYPELDPNRVHITLVEMAPHVLAPFQPKLRRYAANQLTKRGVDLRLNTAVKEVRADGVLLDHETETEFLPANLVIWASGVSAHPQVADWGLTQGRGGRIQTDQHLRALGHRDIFAIGDAAWTEDSPLPQQAQPALQGGKYVARMIRAAVAGQTEPAAFHYVDKGTMATIGRASAIAQITGLPSLRGLPAWLIWVGIHIAQLLGNRNRFATMVNLGQKYL
gnl|extdb|pgaptmp_001050			MTVDEDPLHSWSDENYPRPLLFRSAWAPLNGPWEFAADPDNCGLEQAWFAADCEAFAEVIQVPFPPGSPSSGVWGDQPEQVPEVVWYRKTLTAEQLRQLTESDRVRLNFEAVDYRADVWVNGQHLITHEGGYTPFSAEWPRLTRQAVEVVVRCEDSRSPSQPRGKQAWRDQVDSIWYHRSTGIWRDVWIEAVPTHTVENFQWETDLVRGALRGTVGFNRFVPSGSTLELRLRRGPEVVASLTQAVSGLFAQVQFDLPMLRNRMDWTDWVWSPEHPHLLDLDLTLTTTSGQDRVLSYVGLRTVEAGAQYLHLNRLPVYLRGVLDQGYWPDTYFTAPSPTALKRDAELALELGFNLARIHERSADRRYLTWADRLGLMVWAEFPSTYAFDEDAVRAVTAEWTELVLRDRAHPCIVTWVPFNESWGVPEIASDPRQSAFVSAVVSLTRALDQTRPVSANDGWEQLETDLVTTHDYGAFGSELRANYHSQEAVARSVGGVGPQGRLTLLNQDWAGDRPVLVSEFGGVSLSEKAGWGYSVATNSAELADRLDQLFSALWDSPVLAGFCYTQLTDTAQEANGLCWPNRQPKVPLDQLHAI

gnl|extdb|pgaptm p_000874			MDAYFAAQIVAAERPLLAAGEPLMARAARGLATQIEAELGKKGGPILLVVGAGNNGGDALYAAAELSNWGRLVQVLPVAGRIHQGGLQAALDTGAELLAEPGAPVAELVAAVGEYAPRASVVVDGVLGTGSAGRAALRNPAREVVAVLSQLRSAGQLGLVVAVDLPSGLDCDTGEVPDPAVLPADLTVTFGAYKVGQLVGEGPRLCGRLHLIDIGLGPQLSQLSPTQVG
gnl|extdb|pgaptm p_000388			MPESVAEFVAQVVPAKRQRDAHTLLELYGRISGEQPELYGTIIGFGHYHYQYASGREGDAPAGAFAPRKAATSIYLPDGIGAHRALLDQLGPHREGVGCLYLTDLEQVDLAILEQIITRSYATLSADTYRLRARDGEE
gnl|extdb|pgaptm p_000066			MSENAVVAEVEAEYDEPRKVELTLAKIDPWSALKISFILAVAVGIATVVITAALWLLLDGMDVFGSVEDFLTRLGAESFLELMEYVRLPRVMSYATILGIMNVVLFTAVCTLGSLLYNLIASLVGGLKVSLMDE
gnl|extdb|pgaptm p_000070	K03629	recF; DNA replication and repair protein RecF	MYVSDLALNDYRSYRELVLQLPPGAVVFLGRNGRGKTNLVEAVAYLSTFSSHRVSADRALVRRPLAQSEPEAPVDPDAGRDPGAARSSKSASSASAAAAAGSPEPPTAAVIRARVHHGSQDRLLELEIVQGKANRARLNRGPVPARELLGSLRTVMFAPEDLQLLRGEPGARRRFLDEISLQLKPAYAGLRRDLEQVLRQRAAVLKQLGPHADLRLADDYLAGWDEALARLSAQVSAHRLSVIASLRPGLKRHYRNVSEDDKPVELEYRSHLEKLEARLGLDTGTDLAIPLVDNGYFPDLSAPIEQLAGRYLAALRARRAEELRRAVNLVGAHRDDFEAELATMPVKGYASQGETWSVVLSLRLAQMDLLTADEDTPVLILDDVFAELDERRRLALVQSIAPVQQVLITAAVPTDIPAQLEPARFEVTLDEQLVTQVSPLGASFESVTASAGAAGGDGD
gnl|extdb|pgaptm p_000071	K02338	dnaN; DNA polymerase III subunit beta [EC:2.7.7.7]	MRFKVGRDTLSEAVQWSARAVPQRPAVAVLAGVRLHAADGVLELSSFDYELSARSQVEAEVETPGEVLVSGRLLAEICRSLPNRDVIMELADNHLQIRCGNSEFSLSTMNLEDYPTLPQMPPVQGKVDSAELARALQQVSVAASREEALPLLTGLKLVISGPQITLLATDRYRLAMRTLEWEPKDPSLQAELLVKSKVLADVAKSMAAAGEVELALSELSDNSRNSMIGFSAGGRQATSVLMDGDYPPVMKLFPDETPLTYTVNRQELLEAVRRVSLVAERKTSVRLTFADGVLTLEAGHGESASAQEAVGLAASAEDLQTAFNPQFLQEGLAITDTEYVQFGFTHPTKAAVMCGLAGPNEPIDERFKYLLMPIRFGI
gnl|extdb|pgaptm p_000072	K02313	dnaA; chromosomal replication initiator protein	MSEDLLHQAWTKAMEQVIVDYNVKLGHAPVLRMAKAKGDLDGTILVAVPNDFTRDFIENRIYQPIIDALAAELGRQVQLAFTIDPSLGEEALAATSSSGPNQPAGASPNPQTPAPAEAEQWIMPAPPSHNTVPLPSVAPQMAIDSVSSTRLNPRYVFDTFVIGPSNRFAQAAASAVAESPANSFNPLFIYGGSGLGKTHLLHAIGHYALALYPHLRVRYVNSEEFTNDFINAIRIGKNEEFKRRYREIDILLIDDIQFLQNKEGTMEEFFHTFNALHNANKQVVITSDLPPKMLSDFEDRLRSRFEWGLITDVQPPDLETRIAILRKKAAAENIEVAPEVLEYIATRISSNIRELEGALLRVTAYANLTKEQVDLNLATTVLKDLIMDADDGQITPALIMGQTASYFDVTMEQLSSAERTQKTVNARQIAMYLCRELTDLSLPKIGEAFGGRDHTTVMHANRKITAQMAERREVYNHVTELTSRIKQKARER
gnl|extdb|pgaptm p_000073	K02914	RP-L34, MRPL34, rpmH; large subunit ribosomal protein L34	MTKRTYQPNNRRRHKVHGFRLRMRTRSGRAVLANRRRKGRSRLAV
gnl|extdb|pgaptm p_000074	K03536	rnpA; ribonuclease P protein component [EC:3.1.26.5]	MLPRQNRLVESAAFKRALRVGARGGNRFLAATIAVPPVGTAICHPQKGEAETSVRVGLIVAKREVPTAVARNRLKRQLRHLMRPRLGNFDPGTDIVLRVFGRCQGLSSTELALHLDKALAQAQRKLLGPQAMVKGRP
gnl|extdb|pgaptm p_000075	K08998	uncharacterized protein	MINPVSAALQWLVRGYQKFISPALGPRCRYYPSCSNYMLEALRVHGAIKGLLLGTWRVLRCNPWSLGGVDHVPEKGKWKAPEWIPPDDWVGHDLPDQKDR
gnl|extdb|pgaptm p_000076	K03217	yidC, spoIIIJ, OXA1, ccfA; YidC/Oxa1 family membrane protein insertase	MDTWLHWIIVAFAWIWVKIHDVVVALGLGFGGGAGWIISIVILTLLVRAAIIPLYLKQIKSQRGMQAIQPEIQKLQAKYKGKTDQASRQRQSEELMALYKKNGTSPYSSCLPLLVQMPVLFALYRVIFAVQQLNAGTYVYDNLGPLTKEVAAEIANSKFLGIGLFESLNSTPGALKIVFVVLIGLMVLFQFLTMRMSMTKNMPPAQDPNNPMVRSQKTMMYMMPAMFIFMGFIFQMALLIYMITTTIFSWVQQVWVIKALPTPGSPAYDELIAKREKKYQQWGQPYFEGYDRELEQLTQSAGTDSEAASDLAVKTLAEAKKRAKGEKVDVDFPEEWSTEEQLSVLRGLAFDDWKALPDETWLRELKKTKASAADAKTRKQPKKLSREQRMRRAQLEAADADAQAKYEERQARKEQQKAAKAGTTLTPEEVERRRQERRAAERKQRKQGKKKK
gnl|extdb|pgaptm p_000077	K06346	jag; spoIIIJ-associated protein	MSEDSRSDEIKRLEEEGELAADYLEELLDIADFDGDIEIDVENGRAAVAIVSEGDDSDLLRLVGDDASVLDALQELTRLAVQAQTGERSRLMLDVANYRTNRRSELRVITEEAITQVKVKGEPVELEPMNPFERKVCHDVIAAAGLISESEGVEPNRRVVIHPNPAQAEETE

gnl|extdb|pgaptm p_000078	K03501	gidB, rsmG; 16S rRNA (guanine527-N7)-methyltransferase [EC:2.1.1.170]	MGEDPRVEQPNELVKGLFGTAYYPVEEFARKLAEEGELRGLIGPRELDRLWSRHIVNCQALVPFLPKRGSVIDVGSGAGLPGIVIAATRPDLDVYLVETMERR<br>CQWLAEVVEDLGLENVQIVNARAEELGRSLRADAVTARAVASLDKLIRLTSKLIAPKGKLLALKGRRAQEEVDKAAKELKRYHLSAQVHEVVSVMDGESTFV<br>VECVRQAT
gnl|extdb|pgaptm p_000079	K03496	parA, soj; chromosome partitioning protein	MIQTEKTSAPLANELARNAAQLRALDRAEFERPSRPVVITVSNQKGGVGKTTTTVNLAAGLALGGLRVVVIDIDPQGNASTALGIEHGVGTPSTYDVMVEGL<br>PLAETLQECPDSPGLFVCPATIDLAGAEVELVDSVRREYLLSSAVADYLAQQPDVDIILIDCPPSLGLLTLNAFVAAEQVLIPIQAEYYALEGLSLLWSTIGRIQAH<br>LNPKLSIRHILLTMVDGRTKLSAEVCQEVRSHFPEQVFEVEIPRTVRISEAPSYGQSVVAYDPRGTGAVAYRMAALELNKRLLQERGN
gnl|extdb|pgaptm p_000080	K03497	parB, spo0J; ParB family transcriptional regulator, chromosome partitioning protein	MAGKASGSSRRGGLGRGLGALIPTGQEEEVTKRERPLDVLFPDLQGKNRSGDEVVARGGSARDLLNPPKRATKGSNVSRETIEPKEKKVNVSRETSASTKAA<br>SATPAPGKSAPAKSVSAKSAPAKSTKSKNVSRETSGDTELVDVPGASFGHIPLEAIVPNRKQPRQIFEQSDLDELAESIERVGLLQPIVVRPLALDQLDQATLAE<br>LRENHPEARYELIMGERRLRASELAGLTEIPAIVRNTEESSMLRDALLENLHRANLNPLEEAAAYAQLMADFACTQDELATKIARSRPQIANTLRLLKLPASIQQ<br>WVAAGVISSGHARALLGLATEAEMEAIGERIVQEGLSVRATEDLVRRKRELGEERSPQRSKQRTQSALALSVAERVSNLLDTQVTVSEGKKKGRIIIDFADAD
gnl|extdb|pgaptm p_000081	K00384	trxB, TRR; thioredoxin reductase (NADPH) [EC:1.8.1.9]	MSVANVVVVGSGPAGYTAAIYAARAGQKPIVVAGAIDAGGALMTTTEVENFPGFPDGIQGPDLMENFRAQAEKFGTEIIFEDANKLELEGPVKRVHLDDRV<br>LEAKTVILATGSQYRHLGVPGEDAYSGRGVSYCATCDGFFFRDKRLAVVGGGDSAMEEAIFLTRFASEVVVIHRRDELRASAIMQQRALEHPKISFVWNSEVT<br>EMFGTASLEGVRLRDTVTGETSELAVDGVFVAIGHDPRTDLVKGQVELNQAGYIKVEEPTTVTNQPGVFACGDVVDHIYRQAITAAGTGCRAALDAQLWLE
gnl|extdb|pgaptm p_000082			MAEPVPPEQASEEPLAPRQQSLLRSSVLMASGTMVSRVLGFLRNAMLIAAIGVSLGASDAFGAANMLPNSVYNLLAAGVFDAILIPQIVRALKRKDGNVYV<br>NRLITAAGTILFGITVLTMIGAPLLLSITSSGFPPDVRNLAIAFAVWCLPQIFFYGLYNLLGEVLNARGIFGPYMWAPVVNNVVAITGLGIFLYLWGPSGDVFPA<br>SEFTSQQMVVLAGSATLGVVFQALVLIIPLRHSGVKLRPDFRFRQTNFGSASKVAGWTFATLMVSQLGVLSTTNLASHAVRAAEEADIVVAGLSAYNTAFMI<br>YMVPQSLIALTLSTAIFTRLANNAADGDYQAVARNYTMGVRLIVMLSMLSVAVMLVAAVPLMQLVMPKFDANAASMYASVVVALIMGVPSTGIVMISQR<br>VFFAFENAKPVFLMGIVPTVLQLIVGWSFYFLTGPEWWMVGASIGETVCRILQGFIAIFWTAHLVRTLNAGRLIAYYVRYLVAFAISAVGGWGLLHLIGPASLS<br>SSSTGRFADAFWKVLLVGVVVTGIYLLVLTAIDKSGTRMLGSYLGDRLPKKFQPAFLRSKPEATPNSKSAPSPEAAEAKAPGDEAEEPLTSQLPLLGRMPLGTA<br>GAAGAAGLAGVAGPGGDDNEGSPLVAPSWDEIVDGDFASRSVTRSLGGFQELSTGQIPMIMSQSQRPQSNDTPEGISLNDEETKSPGSDGTEPDSGAPES<br>LPPGEAITTPVGEGESVSDQWRESLDALLMGDNELEHPTAPAASDAPKVRFGQVGATPVAQASPAGEVPAEPSRGSMIPKLPGVGGSGVKRPSGHGPRFN<br>PTGPAMAIAALLVIGGAVFAVNQLRQPIELPTSGDQQMSGEQSGQQSGEVPAADGAAPADPATAPPVINSIWVSSWQDDGGDHPELVGALTDGDPETL<br>WYTRYYDLNQFGEDSMISLLVNLQSEAVVSEITLDVQGSGGEVAIRLPEGDDPRVGQVLATSAIDGTTTIKLAQPTKMSRIGINFISLPTDNEGLNRAQISGLSI
gnl|extdb|pgaptm p_000083			MTRNSQWRAHATALIWCFLLLLGGAGWSWLAPSAHADETTPGTNRGDEFSIAGLSPELYEPGDDLRLTLDVGALRPGTHWFAEAFLQPTQFNSAEQMSY<br>FLQGEGFPGWRIGADQITVRDEATLDWTVPADEMPIPDAQTTGARGLTVRFSNGDQLWEARSLLIYAPLEELSRTRVSLLAHDSLSPTQVDSVEQFVDQFT<br>QLGGFSLAVSPQVAYGTEGRNAELIDQHRQTLVQSGADLVVLPEANADISALAAADLTPLMNLALESRTEGPDDPDSDPQDQPQSADPPIAPVPTDRLLTDI<br>AMASPTGFTLEALQKLTGNTVIAPATWGVPELWHGLITPTGRLQIGMENGVIATEGTPNSIRVVDRWVPVEELLNEPVESPGQELLIRQQLRTISMMVGLQ<br>DPSDQRSLFAELQPGFDWAKPELTNRVLALLNNQWVEPTGLSELLESTPSEIGRQVVPRNGDEVEAYLTQLAPLAREYQRAEAVAMAGRDGNMVLAPYRE<br>TVLAPTAASLTESERTALVTQAVKDLTSLSQSIKVLPLSTVNVVHHDADFPVSITNQGPEEVTLEVGLVPSSPHLQAGQLAHATIPAGGQVEVKVPVRAVGSG<br>DVSVQVVARNLSGQIVDDSQSVTVRVRPTWEDIGTIVVVGAALLLFTFGIIRSVRRGRRRVRRAAPPPPSSAAPVIDSN
gnl|extdb|pgaptm p_000085	K00970	pcnB; poly(A) polymerase [EC:2.7.7.19]	MGVALGDNNSWELVGSGNVPQLLANARQTLAALPRPLLTLAELFTQSGYELSLVGGPVRDAFLGVEPHDFDLTTNARPDQTEELLDRWADTTWDMGRDF<br>GTIGARKGELVVEITTYRTEEYRSDSRKPTVAYGDELEGDLSRRDFTVNAVAMRLPQMALVDPFGGLEDLANGFLRTPVSALQSFEDDPLRIMRAARFAAQL<br>GIDVDLSVMEAMEKLADRLQIVSPERIRAELERLIVSPFPRRGIELMVYTGVADQVLPELSALVATADEHGRHKDVYQHTLTVLDQAIALETGPDGPVPGPDF<br>VLRFAALMHDVGKPDTRKFEAGGVSFHHHELVGAKLTKKRMKALRFDKRTTEAVTQLVAQHLRFHGYGEQSWSDSAVRRYVTDAGEQLQRLHRLTRADC<br>TTRNRRKADFLSAAYDDLERRIDQLAQQEELSSIRPDLDGEQIMSLLSLKPGPQVGRAYKYLLGLRMEEGPLGEEEATKRLLSWWESEGSR

gnl|extdb|pgaptmp_000037			MSGSDGPKWQNLGPGDQRPEPGSVPPPPAPGEVPFPKRRSASQRAGATGGPEPDPTGTRFPPLPKRSELNRSANPVDATVASTPAEPSRVAPSDFDGLVAGTTSEPTGTPDQPEETMTTTKPKKKSWWKRVLLGLLILFFLALVGGSALFFYLYGRTEIPQPDAFSQAQTTKVYYSDGTTEIGTFSEVNRTIIDTSTLPAYVGNAIVASEDRTFYTNSGIDVKGIARALLTNIRTGSRQGGSTLSQQYIENYYIGETTTSYRGKVKEAILALKINRTQSKQEILGNYMNTIYFGRGAYGIEAAAQAYFGIPASQLTLSQSAMLAGIIPSPSAWDPAIDPDQAKARWARVLELMVEDGWATQAEVDAATFPETIPPADPSQGATGVNAYLLQQVRNELIGRDDITPEMLDAGGYTIITTLDPTMQAAAVEAVKVMPEDTPDTVRVALSAVDNATGEILAEYPGSDYQRVQTNAVTQDISMAGSTFKPFALLSSIEQGGSVYDVYDGNSPLEIDDVEVENNDNVSFGQVDMITATQYSINTAYAQLNAKYGPQNTLDTLVKAGIPADTIGLEPNLLNVLGSAAPTNIDLNRAFATLANGGERITPHIVRTVNNSAGVPIFQADTARERVFDPKNVSAIMPALESVVDDGGTGETVAVLGRAMGGKTGTSTDQKSAHFIGFIPQITTAVSMYGVAEDGSSIALPNIGGVGQFHGSDWPADVWLAFMKSATANLPSIDFAWKVAVPTPKPSVTPTPTPTTTPSPTPTPTVTEEPEPEPEPEPEPEPEPTPEPTPTPTPTPTPTEPPATDPDQGG
gnl|extdb|pgaptmp_000036	K02939	RP-L9, MRPL9, rplI; large subunit ribosomal protein L9	MATRKVVLNRDVEKLGSAGEVVEVRDGYARNYLIPRGYAVKWTKGAQRHIDQIVESRRRHEIASVEDAIAIREQIGGLDQVSVTRKAGSNGRLFGAVSPKHVAEALSAALGRVIDHRKVELPQVIKSTGTYPVVIKLHPDVVAESSVTVVAE
gnl|extdb|pgaptmp_000035	K02963	RP-S18, MRPS18, rpsR; small subunit ribosomal protein S18	MAKPVLRKPIKKKANPLKSAKIENIDYKDTALLRKFISDRGKIRARRVTGVSVQEQRRIAKAVKNAREMALLPYSSSGR
gnl|extdb|pgaptmp_000034	K03111	ssb; single-strand DNA-binding protein	MANDTVITVVGNLTADPNLRFTGNGAAVVDFTVASTPRTFDRQSGQWRDGETLFLRCTAWREYAENIADSLSKGMRVIVQGRLTQSSYERDGQKRTSYELQVDEVGPALRYAKAQVTRTPRQDGGGFNQGGGQQGGYAQPAQRPAQAPQQADYSQGGFGAPAGGQAGDPWGTPPGGGSSFDDEPPF
gnl|extdb|pgaptmp_000033	K02990	RP-S6, MRPS6, rpsF; small subunit ribosomal protein S6	MRKYELMVILDPSIDERTVAPTMDKLLQQVTAAGGSVENVDVWGKRRLAYPILKNSEGIYVVINMTTTSEVALEINRQLTLNEEILRTKLLRADEK
gnl|extdb|pgaptmp_000032	K26937	dinF, mepA, vmrA; MATE family, multidrug efflux pump	MSQNSEPRVEGSPVQGRPVDFSARAILSLAIPALGSLIIEPLLIMIDSIMVGHLGVTSLAGLSLSSTVLNTLVGVFVFLAYSTTAVTARLFGAGRRRDGLQAGVQALWLAFGLGLVLVLLLELTAPWLVQLLGADPTVAPEATVYLRAGAPGMIGMLVILAANGVLRGLLDTRTPLYVLAAGAAFNVALNALLIYGLNLGLLGAGLGLSLAQTAMGLAMTWAVVRQARREQVSLAPSRAGVLSSAGAGLPLLIRTISLRIALLLTVTVAAQAGTVALAGHQVVSSVWSMAAFALDALAIAAQGLVGVALGRGRGSELSQLIRRLSWWGVGAAVVIGVVTAGLAPWLPWLFGPDPQMHEVATAALRVAGLAMPIGGLVFILDGVLIGASQGPYLARTGLITLAVYLPTLLALHLWISDRSPMDSAGQVEALTWLWIAFAGWFMLARAVTNSWRAYRPSALLGK
gnl|extdb|pgaptmp_000031	K02314	dnaB; replicative DNA helicase [EC:5.6.2.3]	MDPLSDGFDRVPPHDVEAEQAVLGSMMLSKDAIMEVAEILTSADYYQPAHETIHDTIVELSSQGAPTDVITVAGELKRAGHLQRIGGAPYLHTLVSLVPTAANAAYYARHVRENAILRRLVSAGTRIVQLGYAQDGGDVDDIVDAAQAEVFAIAEKRQREDYIRFADMSEAILEELEDIASRDGKLSGVPTGFEDLDRLTQGLHGGQMIIIAARPAMGKSTLALDFCRSASLHKGITSAIFSLEMSRSEIAMRLLSAETGVFLSKMRSGQMTPEDWTRVSKRLSDVASAPLYIDDSPNLTMMEIRSKCRRLKQQQNLGLVVVDYLQLMTSGKKVESRQQEVSEFSRQLKLLAKELDIPVVAVAQLNRGPEARTDKKPMIADLRESGSLEQDADVIMLLHRPDYYNEDERVGEADIIVAKHRNGETRTIPVAFQGQLARFANMARSPGPGADPF
gnl|extdb|pgaptmp_000030	K00189	vorG, porG; 2-oxoisovalerate/pyruvate ferredoxin oxidoreductase gamma subunit [EC:1.2.7.7 1.2.7.1]	MVEIRIHGRGGQGVVTAADMFATAAFSEGHYAQAFPSFGSERTGAPVVSYCRISDQPIRTREPVLEPDILIVQDPTLLAVVDVFAGLKPGGFAIVNTTKSVAELHLPDLPPDRTLVTIPASDLAKRHVGKPLPNAALMGAVAAATGVVKLSSVEEAIRHRFRGHIANANVAAAQDAYEYLRAAGSGVKEEAHASAN

gnl|extdb|pgaptmp_000029	K00169	porA; pyruvate ferredoxin oxidoreductase alpha subunit [EC:1.2.7.1]	MRQQIEGSQAVARTVAACRPEVVAAYPISPQTHIVEAISALVKSGELEGCEYINMESEFGAMSACIGASAAGARTYTATASQGLLFMVEAVYNASGLGLPIVMTVANRAIGAPINIWNDHTDTMSQRDSGWLQLYAEDNQEAADLHVQAFRIAEELSLPVMVCMDGFILTHAVEVVDVPEAEQVARFLPPYEPRQVLDPEHPVSIGAMVGPEAYTEVRYLAHHKQMQALDLIPQVQSDFQEIFGRDSGGLIRPYRLEDAETVVIGLGSVMGTIKDVIDERRAQGEMIGALGIVSFRPFPVAAIHEAIRHVKRMVVVEKAFSVGIGGIVSSMVRSAVRGEGITCYEVVAGLGGRPITKASLHQLFEQAGADQLEFLTFLDLDRDLVDRELERERLVRRSGPMAENIVR
gnl|extdb|pgaptmp_000028	K00170	porB; pyruvate ferredoxin oxidoreductase beta subunit [EC:1.2.7.1]	MEVELGIPAYRPTEIPARENVKFYQVGSFAVGNRLAELADRSVQSDPDRFNSLTSGHRACQGCGEALGARYALDTAMDASGGDLVAVNATGCLEVFSTPYPETAWTVPWLHSLFGNAPAVATGVAAALKARGKKTRVVAQGGDGGTVDIGMGTLSGMFERNDDVLYICYDNEAYMNTGVQRSGATPPTARTATTQPVGEHPGNTFGQGKDMARIAMAHEIPYVATATVADLRDLEYKVRKAMTMHGARYIHVLVPCPLGWGSLSNETLKLARAATQSGFFPVFEAEAGEVTSVQKIRRPVSVEAYLRPQKRFAHLYRPRRDEETIARLQAMCDRNIRRYGLIAEEQLDEDVLERVLAAPYDPEGRYESMDGYEALRGATARTKRAHADTRPVDKRAGGAQGHGHVEAGTGTGESPTIVKEKSDEQN
gnl|extdb|pgaptmp_000027			MSKTEPRRDTEPFAITLEVGSSLLNETGSWRTERPVYVNLLPPCNIACPAGENIQQWLYKAEDGDYESAWRQIMVDNPFPAVMGRVCYHPCQTACNRGQVDEAVGINAIERFLGDKAISAGWNVEVTAPPSGKRVLVVGAGPSGLSAAYHLRLLGHRVTVRDAGPMAGGMMRFGIPKYRLPRDILDAEIKRIEDLGVNFEFNSKVENIAEVTKDYDAVFIAVGAHIGRRAQIPAGESAKIMDAVGLLADTEVGEQPLLGRRVVVYGGGNTAIDAARTAKRLGAEEAIIVYRRTRDRMPAHDSEVTEAEEEGIMMRWLSTIKHVEGGKIQVEKMELDENGFPQPTGEIEELGADSLVMALGQESDLSLVENAPGIEIDDGVVKVNSQMMTGIPGVFAGGDMVPSERTVTVAIGHGKKASRYIDAYLRGGAYQPAPKHPEASLSRMNTWYYSDAPHQVRDKLEGARRASTFDEVVQGLDEESALFEARRCMSCGNCFGCDNCFGVCPDNAITKIKPGEYEFKYYYCKGCGICAEECPSGSISMVPEEV
gnl|extdb|pgaptmp_000016	K10823/K15583		MTDKTFFPGDEPLQEYEDWKDQPDPLTEQSDRLAEAEIKTETPSIPTEVHPEDANPLLRITDLDVAFQSSTGMVPAVRGANLTVYPGQTVAIVGESGSGKSTTAAAVIGLLPGTGKVTGGTIEFDGRDITNLSKHEWVELRGSGIGMVPQDPMTNLNPVWRVGTQVKEALRANNVVPKSEVGQRVAEVLEEAGLPDAERRAKQFPHEFSGGMRQRVLIAIGMAANPKLLIADEPTSALDVTVQRRILDHLASMAAEKGTAVLFITHDLGLAAERAEQLVVMHRGRVVESGPALDILQHPQHPYTKRLVAAAPSLASKRIETAHAQGIEVTEEELIGSGAGATSTEAIVSVRNLVKEFDVRGAKGEARILRAVDDVTFDVRRGTTLALVGESGSGKSTVANMVLSLLTPTSGQVFFNGEDLSTYSKQQLFAMRRKLQVVFQNPYGSLDPTYSIFRIVEEPLRIHGVGTRKERIDRVTDLLDRVSLPRSVMRRYPNELSGGQRQRVAIARALALDPEVVVLDEAVSALDVLVQDQILKLLNELQSDLGLTYLFITHDLAVVRLLADDVVVMENGKIVERASADDLFDNPREKYTQELIEAVPGRQIQLAL
gnl|extdb|pgaptmp_000015	K15582	oppC; oligopeptide transport system permease protein	MSEHLIPSKNIKRTRHGQDRYVSDVDETGLGAVDAVKDESAPSSMWGEAWKNLRRRPLFWIAAFIITLVLITALFPSLFSSQNPRFCELTNSLAPAGPGHPFGFDKQGCDIYSRVIYGARASVTVGLFTTLCVVIIGTVLGTLAGYFGGWVDTLNSRFTDIIFAVPLLLAAIVIMQLFAAKRNVWMVVMVLAIFGWPQIARITRGAVMAAKNEEYVTSARALGASTWRIITSHILPNAAAPIIVYATVAFGTFIVAEATLSFLGLGLPADVVSWGGDISRAQVSLRVQPMVLFYPALALALTVLSFIMMGD
gnl|extdb|pgaptmp_000014	K15581	oppB; oligopeptide transport system permease protein	MARYLGRRLLQTIPVFFGATFLIFAMVYLMPGDPVAALGGDKGLDPVVQERIRAEYNLDKPFWMQYLLYLKGVFTFDFGKTFSGRPVSEVMAHAFPITVKLALMAIAWEAIFGIILGVIAGLRKGKPVDSTILVLSMLLISVPTFVMGFLLQFFVGVRWKLLPITASSTTDFKSLLMPSIVLAAVSLAYVIRLTRQSISETTSADFVRTARAKGLSGGSVMNRHILRNSLIPVVTFIGADLGALMGGAIVTEGIFNVKGIGGMLWEGIKKGEPSTVVSITTALVLVYIVANLLVDLLYAVLDPRIRYE
gnl|extdb|pgaptmp_000013	K15580	oppA, mppA; oligopeptide transport system substrate-binding protein	MKLKRAWLAVPAVLALSLGACSSGGGDKDQSGTDEKDNAVSSEGTSGGVVTANGTEPQNPLIPADTNEVGGGRIVDLLFAGLLYYDKDGQVHNDMAESIETEDSQTYTVTLKEGQTFSDGSPVTASSFVDAWKKAAAEDALNINFFEPIEGADDYGAGDLTGLKVIDDTQFTITLKRQEADFPLRLGYSAFYPLPQSTLDDKEAGGENPIGNGPYMLAGDGAWVHNEKIDLVPNPSYQGDRKAKNAGVTFVFYTEATAAYNDLLGDSLDVLDQIPDDAITVFQDDLGDRAINQPAAVVQTFTIPERLANFDGEEGNLRRQAISHAIDRAEITKTIFNDTRTPAQDFTSPVVTGYAEGLPGSEVLKFDPEKAKELWAQAEEIAPFEGTFEIAYNGDGPHEAWVTAVTNQLTNNLGIDAQPKAFPDFKSFRDAITNREITTAFRTGWQADYPSAFNFLSPLYFTGAGSNDGDYSNPAFDAALTEAAAATSVDESNKKLDEAQTILFEDLPAIPLWYQNGFGGYSTHVQNVEFGWNSVPLYYEITKD
gnl|extdb|pgaptmp_000011	K06213	mgtE; magnesium transporter	MKIPFTRRKITVETRSPRSVPTSLQEVVQIGTPRAMALWLASTYNDERDTQLGQLNREQSLSLTDLLTPESAEELLESVSPHTAFAFLKFLPVQMAAGLLESLDSDEAARIVDLMNEDERQRILRAMENSHSALVRGLLAWPEDSAASRMRPDFLHVGPEATIQDAVDAARGDPDDLAEGVFVTTDGKAGQIVHGWLSPSALVLGRRAHPVTTQMTPVSRLEQWAIKPLDDQEKVSFRARERDADVIPVMDGKYLLGVISSDTVADILQEEATEDAERQGGSAPLDLPYLQASPILLWSKRVVWLLVLFAGAMYTGNVMQAFQDELEAVVALSFFVPLLIGTGGNVATQITTTLIRAMGMGEVHLRDLGRVIWKESRTGLLTAITMATAGAIRAWTLGVAGEVVLTVVLALAAIVLWSALIASILPLLLRRLGVDPAVVSGPMISTIVDGTGLLIYFTVAQTVISGL

gnl|extdb|pgaptmp_000008			MFGRYREILSLPGALKFSIAGVFARFPMALVGISIILMVKQLYGNYALAGAVSAAGVISFAVGAPLLSRLVDAHGQARIMVPSLIISGISLSILTVAAMNQADPWILLVTSAIGGATSGSMGALVRARWAFVTDRPGQIQAAYSLEAAFDEVVFVIGPVLATLMATSIHPTAGLWLAVLLVVFGGLWFLSQRKTEPPVIKPDAHVGGRSVMLNPAMIVLALTYVGAGALFGANDLAVVAFTEEHGQPGLAGVLLAVFSFGSLIGALVYGARTWRWPLWKLFGVGVFVLGLGVSTFVFANSLVMLAIIMVLTGLVVAPTMTNVSTVVQRIMPSSRLTEGLAWMSTAMNIGVSIGAGVAGPVVDVQGAHGGFVVVIGSAWLMVLAMALGLRTLRRETEELPRAIDTGIM
gnl|extdb|pgaptmp_000116	K01835	pgm; phosphoglucomutase [EC:5.4.2.2]	MHERAGQLATAEDLIDVDALLSAYYEVQPDPNNPNQRIVFGTSGHRGTSLNGTFNEAHIVSTTAAIVEYRTRQGYDGPLFMGADPHALSEPAWRSALSVLSAAGVQTYVDSRRAWTPTPAVSLAILMANGAPGELRQSGPGLADGIVITPSHNPPADGGFKYNPPTGGPADSDATSWIAARANELMAGDWRSIPQAEIGAPSQADNVHFYDFLANYVAELETVIDFQAIRDAGVRIGADPLGGASVDYWGAIGERYGLDLTVINQRTDPQWPFMTLDWDGKIRMDCSSPYAMASLLERMQPDVAGNYPFDIATGNDADSDRHGIVTSDGLMNPNHFLAVAIEYLFEHRPGWSAEAKIGKTLVSSSLIDRVAAAIGREVIEVPVGFKYFVSGLLDGSLGFGGEESAGASFLRKDGTVWTTDKDGILLCLLAAEITAVTGKSPSQLHREQVERFGESWYARIDQAASKEEKAKLANLSPDNVTASELAGEAITAKLVRAPGDNQPIGGLKVTTENAWFAARPSGTEDVYKIYAESFISEEHLGQVQSAAKQVVDEALGQ
gnl|extdb|pgaptmp_000117			MGKASRRKKIDPSKRKNIRPAIAFVDRPFQGLERERELVAMREIIPCATMTAKTTAEHGGTEFDFVTLLPDGAGAMVRGDGRILVGVQTRFSTGDLSHDLGAALTAALKMQLEGEEGVAQFDVRDPAPRLQELLAEGELAPMTVLKDFGYWFDPNEEIDAEMAAALEQNREDMVPTEEVPGVEGMYWCEMNNNFIRYVLAMDEDLLFTALARLQAAGQAQLGEGSKFVGAFRACGIAIPVFQVNPELSAADFAAPAAQLRDNLELALKQTEPLTDDERRARAGMVSRQVTIR
gnl|extdb|pgaptmp_000118			MERESETSAGPQRVAVIIACQDVRKYISSTVRACRAIPSVDLIVVVDDGSTDDTSQVARAAGAVVVRHSVSRGRASALETGVKVAAMRDRTDWPARNLLFLSPDLGDSAVEANALVEAVNSGLADCAVGVVPVEDGTRRGATWVMAGNGIRQSTGWDVSGPLSLQRCLTREVINEVMPFSNGWGADVGMTIDLLIAGFSIVEIPCAFHHLDEHRVEPNSHKRAQYWDIWWAIKARRLRRRRVPLVLRIPSWDQEVGRPYAIRGRTLSASRPDEGGDQPEEESTGA
gnl|extdb|pgaptmp_000120			MSDFDRTTELYVTGLTCGHCVASVTEELEEINGVKNVDVILVKGGESKVTVLTDTAIDDEAYRDAIQEAGYDLVRISRDL
gnl|extdb|pgaptmp_000122			MARYYPLRRIPEAARPGHFARRMLPLIPAILRSAAWTLAASGAPPGSPMREVLQAGAVQRGDQALEKAQDTFFAEPGLDTLLVRELSKLGSTISRWNRVRLWTYIALGFDRDGRAARNLRRIPRMGYDSLMLTGLAFGKSLGYFTGGKVHAQLWWDSLGEVGQLPPEHPTPQVRRFQPPRSLGDLAADIDDLYWADAYGQAVKITQVGRGSNRRWLVSVPGTDHPEPESKPNVADLETNLREELNLPSAMRRGVIAAVNQAMAAAGIEPSARVHERVLVCGHSQGGMVAVGLAATAPAELGFDVEGVITMGSPTRRLRLRPSVDMLALEHVQDVVPALDGTPRKVADQRVVVQRSLVKPRLGSLYYAHASSTYTDTLRQLERRVQITRWGREAQVVEALQRYLPRPGEETRVTHHYIWQELVPTHANTAWTEYLELDRPDWEPVVYGDEVVSPELPPTPQELMEKVEAALENSALLAQLSRPEEAPRET
gnl|extdb|pgaptmp_000124	K01756	purB, ADSL; adenylosuccinate lyase [EC:4.3.2.2]	MNSAAPTPSFTPLTHEALSPLDGRYREQTAPLATYLSEAALNRARLHVEVEWLIFSVAAGIYPGLAPLSAEDQAYLRSLPANFSEETRARLADFERETRHDVKAVEYLLREHLTGAPEGSPLPELGELVHVFCTSEDINNLAYALCLQGAIAQVWRPAAASLQATLAELAHRYADIAMLSRTHGQTATPTTLGKEMAVFAHRFARQLDRLDQCEYLGKFNGATGTFSAHVAALPQVDWLEVSRGFVTHLGLTWNPLTTQIESHDWQAELFGVIVHFNRIAHNLATDCWTYISLGYFRQDLAAQGSTGSSTMPHKINPIRFENGEANLEVSNALLESLSATLVTTRLQRDLTDSTTQRNIGSALGYSLLALDNISRGLAGVAADPEVIAADLGSRWEVLSEAVQQVLRVQDAIGSARVDSPYEQLKALTRGQGITEESLRQFIENLPVPDSVKETLTALRPETYVGLAPQLPELLD
gnl|extdb|pgaptmp_000128	K08972	putative membrane protein	MQLVLRLLGNMAGIWITSLIVSSVRFTQQDDLATNLLALAAVALVLTFVNSLIRPVVKVIGFPLYVITFGLFALITNALIFSLTGWLAGLFHLPLQVDSFGGALLGGTITAIISALVVAVLGSFSKDRY
gnl|extdb|pgaptmp_000130	K01808	rpiB; ribose 5-phosphate isomerase B [EC:5.3.1.6]	MTKVHIAGDHAAFELREALIKHLGERGYEVVDHGAYVYDALDAYPPMCIECAEAVVADGGASLGVVLGGSGNGEQMAANLVKGCRAALVWNESTAQLARQHNDANVVSIGARQHSLEEAIRLVDVFLETPFSGDERHLERIRMMADYEAAHHA
gnl|extdb|pgaptmp_000129			MNKRTLIKNGWLPLQHGAWSMTFTPLLLGIILGGPSWLQLLLVFAWTAAFLFFNVFGLLIKARRKERYWKATITYGVLAGVGALALVLIRPHLLVWALPLAVFFSWAIIEILRRNERSLGARVSAILASSLMVPVAFSLGSHPLDWRHAWVATAVVALYFVGTVPYVKTLIRERGKRSWLVGSLTYHVVMLAIFIGGAAAHLLTWFVPVVGLILLARAWAYPLVSLRRGRPLHQMFVGLTEFGYSALVLISLIVGYPAV

gnl|extdb|pgaptmp_000145			MVENSEQPRAKRAARSMTIIQFSEGIIAFLVLAALIGVLAAGFLLPVVGAAGAAVKAGPASFDEIPSDLELVTPAEESRMLDADGNEITRFYSQRRIIVASDQIAQVMKDAIVSVEDRRFYEHHGVDPDGLARAAINNLGGNATQGASTITQQYVKNMLVERGIQAGDQDLIDEAQEQSTERKLREVRYAMGLEARMTKDEILTGYLNIAPFGPTVYGVEAAARQYFSQSASDLSLAQAALLAGITNSPVEYNPLVNPEKAQERRDVVLRSMLDEDKITRDEYNEAKAMNVVDYLKPDNRTEGCIGATGSMGYFCTYALEELLSDPAYGETRAERLHLLETGGLVIRTTIKPKLQADAWNTVTGTVPVMDESGVNTAIVSVVPQTGQIVAMAQNTNFGPPTEAEPRNSEVNFNVYQNRGGGTGFQPGSTMKVFTLAQWFKEGKGAYDSVGSNNRDYPAGSLKCPSNPDFYTGDFRFDDLAGKDGPHSVLDTMKLSINQGIASMASQVDYCQIFQRAAEAGVVDVDGNALSPNNPSQMIGGDTGVSPLAMATAYATIANNGIRCQATALTEVSDRDGNPIKTYTPNCTQAWDTKVANQVATVLKQVANSYDVYLSRQFGAKTGTTDDNANTWMVGFVPQLATAAWVGTAQNSSRPIQNMWINGQYYDSIYGGTFVGPIWTTYMEEAITGTEVINIPDVWIGNKPLPLPVPKTEEEKKESDQTPTNQGNQGNNQNNPGQGTGGEQDDD
gnl|extdb|pgaptmp_000146	K18955	whiB1_2_3_4; WhiB family transcriptional regulator, redox-sensing transcriptional regulator	MTQATSLIDRRWVDRAVCAGGEPDALFVQGAAQRQVRQRCLACPVRIECLADALQTEANFGVWGGLTERERRAILRHYHDVEDWYSWLLHSDDGLAEELRQNRIPRVFARMRDCA
gnl|extdb|pgaptmp_000147			MNKWEYATVPLLVHATKAILDQWGEDGWELVQVVPGPAGSENVVAYMKRPKAQ
gnl|extdb|pgaptmp_000148	K10914	crp; CRP/FNR family transcriptional regulator, cyclic AMP receptor protein	MSIDDQFISRVPLFAGLNEEQYQALAARTGTVSLRRGEVLFEEGERGDRLFIVTEGKVKLGHTSEDGRESLLAILGPGEMIGELTLFDPGPRSTTATAVSPVSMLVLEHRDLMEILDVNPELAKHMLRALAQRLRRTNESLSDLVFSDVPGRVAKALLDLSDRFGTVTDNGVHVPHDLTQEELAQLVGASRETVNKSLADFVSRGWIRLEGRAVTLLDVDRLSRRAR
gnl|extdb|pgaptmp_000153			MSVDEVHEACAQLRFAEGLRRRWEEARAESAIREACGLALLAGARVSPTQLRAVVVTGRQEGRPGPDLAVALGAWKATWTIVSGLPPLNVRSGLGPAKSSGLSLRQSLAGMQRDYGGYLAEVGLADTAQLTIPRDPTQWGQLLAGLEGDTQPALTLAGRAWAALTVLEPFEVGSELMGILTAKLVLAQRGVEPTAVSVLTALAVEQRARYQLALAELRAGESAEWDQFVADAVVRGCEVGQQVAREVQAGRLAP
gnl|extdb|pgaptmp_000155	K02283	cpaF, tadA; pilus assembly protein CpaF [EC:7.4.2.8]	MGGSKQVPAERGLRWMRREVAVGTTPVSAVVQRAETASGAADRALSWRSLRAGQLGMSGELFDLLSEPDVTDVLINGTEAWVDRGSGCQRVELERLSEFDTRRLAVQMAAAAGQRLDDASPLVDAFLGDNIRLHAVLPPLSREGPLISLRVLRRGGYSLTQLVELDTCTPELGRVLAGLVKNRVSVLISGATGAGKTTLLSALLQEVDEAERIICIEEVSELFPTHPHVVHLQERQANVEGVGQVTLVELVRAALRMRPDRLVLGECRGPEVREVLSALNTGHSGGFTTLHANSLAAVPSRMVALGQLAGLSKSVLTTQVATAFQAVVHLGRGSDGQRRVLEVGLLREENGLVTAPAWQWSAGEYRAGPAEAQLSALIGEDSLGD
gnl|extdb|pgaptmp_000156			MAIDLAFLGMAGGILLLTAWSLVGQLHRRRQFREELQRRTARIGVEGEPPAERREFWLARRRRQPRDLAAGEVAELALAVAARLRAGDPAGTAWEKAWPTERLGPFPGLDELGAPRSLKEAAESKLQRSSEQLARDSMRSLRSACRFSHLVGAPLAGVLELIAESIAQAGQALAAQRQAFLGPRLSAQILALLPILLVVGSQLLGLGSVVWLVSSPLGWGCLLLGSGFLGAGHVSTRALVRRAEREGAGQIGSTLVCDLACTGLSGGSSIPHLLTSLSEALQVPELDRIARELVLDTPWVQAWTPLPPETELLARGLRPAWEEGISPILLLTHLGASRRRDMVARAQEAARRLEVQLVVPLGLLLLPAFIVLGIVPLVFTLLGQQGPL

gnl|extdb|pgaptmp_000067	K02469	gyrA; DNA gyrase subunit A [EC:5.6.2.2]	MSDEKNSEELNSSDQNREAAEVAEAVAAAAGEGTVGVIGRIDPVDLESEMQRSYLDYAMSVIVGRALPDVRDGLKPVHRRILYTMYDGGYRPDAGFYKCMRVVGDVMAHYHPHGDASVYDALARLVQWWSMRYPLVAGQGNFGSPGNLGPAAPRYTECKMAPLAMEMTREIDEATVDFQENYDGRAQEPTVLPARFPNLLVNGSEGIAVGMATRIPPHNLREVSAGVQWYLEHPEATREELLEALLERIKGPDFPTGATILGRKGIEQAYRTGRGAIVQRAVVDVEELHGRTCLVIKELPYQVNPDNLAAKIAELTNTGQLTGIADIRDETSGRNGQRLIIVLKRDAVAQVVLNNLYKRTQLQDSFPANMLALVDGVPRTLSLDGFVHYWVKHQLEVIVRRTKFRLAKARERLHILDGYLRALDMLDEVIALIRRSPTVDEARTGLMELLQIDEIQANAILALQLRRLAALERQKILDEHAEIKARVEDLQDILSSPTRQRQIISDELAVIVDKFGDERKTAIVPFGGDLSDEELIAEERVVVTITREGYAKRTREDNYRSQKRGGKGVRGAQLRGDDAVEHFFVTSTHDWLLFFTNQGRVYRAKAYQLPEGGRDAKGQHVANLLAFQPGEAIAQVMRIESYEDAEYLLLATKSGLVKKTRLAEYDSPRSAGLIAINLREDGDGQPDEVVSAEIVNATDDVILVSKQGMSIRFTADDEQLRPMGRSTSGVMGMRFRPEDELLTMEVVEPDADLLVVTEGGYAKRTPLAEYRVQGRGGLGIKVADLAPERGALVGALVIEPEEDVMVITESGKLVRVSAGDVRSTGRNTKGVIFARPDESDRVIAITRNGGDEEDQEDAESGEAETTESEGTENE
gnl|extdb|pgaptmp_000068	K02470	gyrB; DNA gyrase subunit B [EC:5.6.2.2]	MVDGQDQIESNSAPAPGNDHYEAADITVLEGLEAVRKRPGMYIGSTGERGLHHLVYEVVDNSVDEALAGYCDHIKVTILADGGVRVEDNGRGIPVDLHPTEGKPTVEVVMTILHAGGKFGGGGYAVSGGLHGVGISVVNALSARVDTVVKRQGYAWRMSFANGGHPISQLARGEETEETGTIQTFWPDPEIFETVNFSFEVLRQRFQQMAFLNKGLRITLTDERPGQSVAGDEIAGDAEEVTATEEEFRTVSYCYQYGLRDYVEYLDTTKKAQVINNDIIDLETENAEGTLSLEIAMRWTQAYAPTIHTYANTINTTEGGTHEEGFRTALTTVINKYARDKNLQKEKDENLTGDDVREGLTAVLSVKLTEPQFEGQTKTKLGNTEARTFVQTQVYQLLGDWLDAHPQEARAIVAKGQAAQQARVAARKAREATRRKTALDSVSMPSKLRDCSSRNAEECEIFIVEGDSAGGSAVTGRNPDTQAILPLRGKILNVEKARIDRAMNSETIQALIGALGTGIGEEFDREKLRYHKVIVMADADVDGQHIATLLMTFFFRYMKPLVEEGHIYLATPPLYRLKWTNAPHQYVYSDPERDEALEEGAARGWKLPKNGAIQRYKGLGEMNDQELWETTMDPDRRLLKQVEVGEAAAADEIFTILMGDDVESRRTFIQRNAQDVRFIDA
gnl|extdb|pgaptmp_000084			MTTHDRLPVPGIPHPPRQRSATRSWNRFRLPISEETSAGGLCVRVDRGIPFVAVIARRGRNGKLEWCLPKGHLENGETAVEAARREIEEEAGVRGEPIQRLCTIDYWFSSPRSRVHKTVHHYLFEYVGGEITVDNDPDQEAEEAAWVPLRAALTKLAYPNERRVVQVALELLYQGSQR
gnl|extdb|pgaptmp_000119	K17686	copA, ctpA, ATP7; P-type Cu+ transporter [EC:7.2.2.8]	MTLPNEFDSTPTVHPDSHHGTEVGEDAHRRARSLRTRFLLTLPLGVVIMALSMVPAWQFPGWQWVVGVLALPVVTWGAWPFHSAALRAARHGSTTMDTLVSLGVIASSLWSYWALLLGGAGEIGMHMSMSLIPRASVHGAHPELYFEGAAMIVVFLLAGRWAEAATRYRAGDALRALLTLGAKEATVLDQQGQERVIPAADLQVGDQFLVRPGDKVATDGVIIRGSSALDTSLLTGESVPVDVGPGDEVTGATLNTWGSLVVEATRVGSETTLAQIGRLVTEAQAGKAPVQRLADRVSAVFVPIVIGISIVTLVGWLLAGGSVQAAFTAAVAVLVVACPCALGLATPTALLVGSGRASQLGIIIRNAEILEQTRTVNAATLDKTGTITTGNLAVESVWPEDSGVLALAGSVESHSEHPLARAIAEAARAKTPELAPSTSFTNHAGGGVSALLDGDLVLVGSSRWLARHGVSLPVDLTARHEQAEASGATAVLVARIPNWHESTADMSEQFTPAAAADGDPLIRVNVKVGGMTCASCVNRVERKLRKLPGVDAQVNLATETAAITLRADHSDEELVNAIVGAGYEAEVTGREQLGNTLVAPARTEGKQRTLPDSLPDTQVLGLLVLRDTIKPSAAEAIAELRAEGIEPILLSGDNQRAAEYIAHQVGITRVLAEVLPEEKQREVARLQEEGRVVAMVGDGVNDAAALAQAANQGVGIAMGSGTDAAIAAADITLVNSDLRSVPRAIRISRQTLSTIKGNLFWAFFYNVLAIPLAVAGLLNPMIAAAAMAFSSVFVVLNSLRLRRAG
gnl|extdb|pgaptmp_000144	K07098	uncharacterized protein	MIRRVPDLGATFQGSLVETGRQPSIVPPRVVGAMRRLALTGAIATGLSLGTLAYAHWESRQPVLRRYQIPVAPREGFTRLRILHVSDLHMFPGQEFITRFLRQVSEREEIDLVISTGDNLGDADSLDQLLAAYEPLLAYPGAFVLGSNDYYSPQTKSWISYLKPNRSATATKRHRRTVPDLPWMELVEHLGAAGWKDLSNRSDSILVQDQALTVALMGVDDPHIRRDRMPTPPASWLEPGAVRLGLTHAPYLRVVNEMVREQADLVLAGHTHGGQVRVPGLGAVITNSDLPRQYASGMHVWGDQPAGSGPSWLHVSAGLGTSPFAPIRFACPPEASLIELVPAD
gnl|extdb|pgaptmp_000756			MGLRHVVMWELNADDRAETAKELAAELRGLAGRVPGVESLSATVNTEAIEGNWDLVLDVTFTDRAALDAYQVHPDHQAVAAKIRAAASRRAAIDGLV
gnl|extdb|pgaptmp_001993			MTSQQSEAGHAAIAQGRTALGIELGSTRIKAVLIDEAGVPLATGGYGWNNTLVDGLWTYPWDSVWEGLRGAYADLVADVRQRYDLELTTVGSLAISAMMHGYVPLDEAGEPVAGFRTWRNTNTAAASTELSELFQVNIPHRWSIAHLYQAVLDGEDHLSRLAHITTLSGLVHLRLSGQFVLGVGDASGMFPIDSEAGDYDRTRLAQFAEVAGARGWNWDVDQLLPRVLVAGQEAGRLTEEGAALLDPTGKLQPGIVMAPPEGDAGTGMVATNALAPRTGNVSAGTSIFAMVVLERTMAGYFPEIDPVTTPEGLAVAMVHSNNGTSELDQWVGVFAEFARSAGQELELGQVYQILYQQALTGAPDAGGLLAYNLLSGEPIVNLEEGRPLYVRSRGESLTLANFIRAQLLSVFGTLRLGMEILADQGVEVELLRAHGGLFKTAGVAQRLMAGALQAEVEVGSGAGEGGAWGAALLALYRAQGEGKLLRDFVAERIFADAEAVRVAPDPDDVAGFEQFMDRYRRGLPVVAAAVDCVD

gnl|extdb|pgaptmp_000725	K03077	araD, ulaF, sgaE, sgbE; L-ribulose-5-phosphate 4-epimerase [EC:5.1.3.4]	MGQQQQWSPEMEAAIRAAQQRVCALHAELTRWNLVVWTAGNVSERVRGSAPDGSDDLMVIKASGVTYDQLTPDHMVVCDLDGNLLRGEHSPSSDTAAHAYVYRHLPEVGGVTHTHSSYATAWAACGEPIPCVLTMMGDEFGGEIPIGPFALIGDDSIGRGIVETLSNSRSRAVLMQNHGPFTVGRDAQASVKTAAMVEEVAKTVFLARQLGTPLPIPADKIDALYQRYQHVYGQH
gnl|extdb|pgaptmp_000726	K01804	araA; L-arabinose isomerase [EC:5.3.1.4]	MRSAMAGREVWFLTGSQELYGPDTLAQVAHQSQQVVEQLNLSGDLPVPVVWRPTLKDPEEIRRVILEANGRDEVVGVILWMHTFSPAKMWITGLGTLQKPMLHLHTQESEQLPWDTIDMDFMNLNQSAHGDREFAYLVSRLGINREIVVGHVSRPDVRARVATWIRACLGWAEAHQLKVVRFGDNMRNVAVTEGDKTEAQRVFGVSVNTWGVTDLVNQLEQVSEAEAAELAQTYEELYDVQPELRVGGERHESLVYAARQEIALRTFLTEQGATAFTDTFEDLGGLRQLPGLAVQRLMAEGYGFGAEGDWKTAVLVRLMKVMGEGLPGGASLMEDYTYNLVPGQELILGAHMLEVCPSLTTTRPRVEIHPLGIGDREDPVRMVFSADPVPGLVVALADLGSRFRLTANLVDLVDPLEPMPNLPVAHAVWKPQPDLVTSAECWMHSGGAHHTALTTAVSVDVLRIFADLARTELMVIDADTTTSRFRQELRWNKPAF
gnl|extdb|pgaptmp_000724			MTEQDSASASDTGRRRRSSGTRPPSMADVAAVAGVSHQTVSRVLNQADAVRPETRDRVLAAIDQLGYRRNETARTLATRTSRLIGVITADFVLYGPATTLLSIQLAAAERGYMVSVATLTEFSAQRLRQAIDQFLSQGVAGIIISAPVVRIAEELERITVPVPSIAVASGWINPDSTIARIGVGQRVGVRQALEHLAATGCDEVAHFAGPPHWFDSEERAIQWNESMAELNLRFGGYDRGDWTAESGYVMAQRMLAGHLPEAIVIANDQMALGALRAFGEAGVRVGKDVRIVGYDDEAGTAFFYPSLTTVRQDFFRLGREAVQMLLAVIRGKAVGNILIPSELIVRDSA
gnl|extdb|pgaptmp_000727	K10546	chvE; putative multiple sugar transport system substrate-binding protein	MNVPFKNKLMAAAGLLTVGALALSGCDGGGAGSTNTEQTESGDAAASGDAGEAECGVGMTVGVAMPTQTSERWIADGEAVKAGLEEAGYQVDLQFANDDIPTQTQQIDQMITNGVDVLVIASIDGTALSSQLDAAGAAGIPVISYDRLIRDNENVDFYVSFDNYLVGVAQGTALLHGLGLVEEDGTKAADAPKGPLNIEIFAGSPDDNNAGFFFNGAMDTLKPYLDDGTLVIKSGQTKFDQAATLRWSQEAAQKRMEDLLSSTYPDPSELAGVLSPFDGISRGIITALQGVGLGPTIADGLPIVTGQDAEIASIKLIADGVQQSTIFKDTRLLADRAVQVVQDLACGREPEANNTTDYDNGVKVVPSYLLDVVTVYQDNIESTVIESGYWTEDEVKSGVAN
gnl|extdb|pgaptmp_000728	K10548	gguA; putative multiple sugar transport system ATP-binding protein [EC:7.5.2.-]	MTENILEMRDICKSFSGVQVLKNVNFDVRRGEVHALCGENGAGKSTLMKILSGVYPHGEYTGQIIYRGTEAKFGSVRDSENAGIGIIHQELALVPYMTIAENLFLGSKKPTKLGLIDWNQVNSEATKMLQRVGLHEPVVTPVSQLGVGKQQMVEIGKVLAKDVELLVLDEPTAALNDADSEVLLDLVRQLRDSGVTIIIISHKLNEIEAIADRTTILRDGETAGTLDMSDPAVTKNTIIELMVGRTLSQFYPPRQSNPGEVLFEVRDWTVHHPTQEDRVIIKDASFNVRRGEVVGLAGLMGAGRTELAMSIFGRTYGSKISGQLFKDGQEIKLGSVAAAIKHKVAYATEDRKVYGLNLIDDIRRNIGIAGIHKHSKRGVVNDNEELAVAIRYQEAMNIRANNVLQLAGSLSGGNQQKVVLSKWLYTDADVLILDEPTRGIDVGAKYEIYTLINDMVAQGKAVIVISSELEELLGISDRIYTLAYGRITGEVPIEEATQTELMRLMTIEPVRETRS
gnl|extdb|pgaptmp_000729	K10547	gguB; putative multiple sugar transport system permease protein	MSVRDFLKNTMGTSIRQSGIFVAFVAIVIAFSIINPTFLSANNLTNIVLQYSYILVLAIGMILVIVLAQIDLSVGSVVALTGAVSAVLVIRHGFPWWVGVVAALATGIAVGVFQGFWVAYVGIPGFIVTLGGMMLFRGLTYNVLDNVSLSPFPKGYYNIANGFLNGLFGGYGVDVFTLVIFAIGVIGFVYFQWRARSEKVKYGQIVESLWIWGIKMLILAAVVMAFGYKLATARGLPIVLIILAVLIFVYGFISQRTVFGRDVYAIGGNKSAAALSGINVKTVQFWVFVNMGFLSAVAGILYSARMNGAQPSAGNMFELDAIAACFVGGASTTGGIGRISGALIGGLVMAVLSNGMQLMGASTSTQQIIKGLVLLLAVAFDVYNKRRAAVSA
gnl|extdb|pgaptmp_001977	K02004	ABC.CD.P; putative ABC transport system permease protein	MKQLVQANLRTHGRRYLATGLAVFIAVVFVVAVLMLSGALGTSIGNSFADQYRGSSAVVEDREGTGTDVVEEVSGTPGVELVVPALNTYAQFGGSGQQFGSIAGVLPEPLFRPHLSQGELPTDPNQIALDAKLASALDLTVGDRLTIHPYTEWHSLDVEVSGIFGEGNSPASVNSDALMTPAGIHQLSGDSYASTYLVVGSDGFTQEQVAAAVRAALSSDELTVQTEQEAVEAALAEANLASSGMSLALMVFPAIAIVVAIIVVSTTFQVLVRQRQRELGLLRCVGATGRQVRRLILVESLLVGVVASALGVAVGVIASALAISGFGLLPSLTQAFGSASWLTLFLVFVLGVLVTVLAGLRPALQVGRVAPLTAFTAAAVEPVERTRGWWVRLSLGAFVSIAAGTVMAITANWHSTAGLGLAIVSGMATLVGLTLFLSAVFPRLVAWVGAPMRSTLGQLATGNTQRSPGRTAATGVSIFIGVSLIVMMLVGASSLSLTMNRELDSRLATDLVITSSNGELTEADVQAVTDLPGVAKTAPEWGLADASVDGEPVTLYGVDASDRVARTQGEPLGDGRVLVPETSGLESGQTVNVCVAQNCADLEALVDTSWLAQDGRAGVSQATLDRLGAGVTPQMQAILAQLDSTSDYRTVSNAIGKLDQSWSLGGAASTRAQFDEMITMILTVVVALLGVSVVVSLVGVSNTLALSVAERTRENGLLRALGMTRRQVSRMLTWEAVLISFTAALVGVGAGILFGWIGTVAVMMEVAAAQLDIPWLQIVLVLALAIVAAALSSWWPGRKAGRTSPVEALAV
gnl|extdb|pgaptmp_001976	K02003	ABC.CD.A; putative ABC transport system ATP-binding protein	MLEPPIVSLQQVEKTYGTGEAAVTALNQVSVDFYPGAFTAIMGPSGSGKSTLLHLLAGLDVATAGSIRLEGVELTGLGDDQLTRLRRDRIGFVFQSFNLVPTLDARANILLPPSLAGQKVAAEWFDQVVAALGLTDRLSHRPHQLSGGQQQRVAVARALVSRPAVIVADEPTGNLDSHSSAEVLRLLRAAVDQLGQTVIMVTHDAPSAAQSDRVLVVRDGQIVADLDRPGVEEIEAVSR

gnl|extdb|pgaptmp_000444			MTSQPDHHEAHLPSEPSGSIAEPGSGSPRRPWKTRRRHRVPRAVVELLGADGTLHPALLPGIGVAETDRRFGVDWWVFGVAGAFIALFVTFGLSDSAALGELASRGVNWVSTHTGWLFSLLTVAVFAFMIVVGYSSKGRIRLGTDEERPEFSTFSWVAMLFSAGMGIGLLFFGPYEPLTFFLTPPPAFDVDPGTVDAMRAAMAQTLLHWGPLAWSYYALVGGAIAYSTYRRGRSAVISAIFDPIFSDRLKGPLGRVIDIFSILVTLFGTAVSLGIGALQIGRGLELVTGIGPVGNTLLISLMVLLTIAFIFSAVSGVKRGIRALSNINMFLALGLGLFVFLAGPTLFLLDFIPASLISFFTNLGPMLGVFSSADQTSAEFMSSWTTYYWAWWVSWTPFVGMFIAKISRGRTLREFVTVVIIIPSLVCLLWFGTLGGTSMWFESQGLEMSGAASSEDLLFQLLAKLPLPVVLSVIAMASLMIFFITSADSASIVMASIAEKGRPTPSRPTTVVWGVALSATAITLFLAGGADALSALQALVTISALPFAVILIGLMVAWWRDLSTDPLVLRREYATVAIDEGIRRGIRQHGDNFAFSAGPVAPADGAGSWLDSDDPALTQWYEDALVAADEATDDWSENPAQSGKPESQE
gnl|extdb|pgaptmp_000982	K01955	carB, CPA2; carbamoyl-phosphate synthase large subunit [EC:6.3.5.5]	MPKREDIQSVLVIGSGPIVIGQAAEFDYSGTQACRILREEGLRVILVNSNPATIMTDPEFADATYIEPITPDMVERIIAKERPDALLPTLGGQTALNTAVALSESGVLDRYGVELIGVQVEAIQAGEDRQLFKEAVEACGAEVCRSFIAHSMDDCLSAAEALGYPLVVRPSFTMGGLGSGMAFDEDQLRRIAGAGLQDSPTHEVLLEESILGWKEIELEMIRDLADNIVTVCSIENVDPVGVHTGDSITVAPAMTLTEAEYQNICQIGADLIRQVGVRSSGCNIQFAIHPENGRVIVIEMNPRVSRSSALASKATGYPIAKIAARLAIGYLLTEIPNEFAPSLTAADGPQIDYVAVKVPRFAFEKFTAGDTTLTTTMKSVGEAMALGRNFMEALNKAFRSIDKPGVGFSWSEPVPTAAQVADLLQAVQTPTEERLNQIQQAIRGGATLEQLNEATDIDPWFLDQFFLLEEVAQELAGSAHLDASLLRRAKEIGFSDQQIAQIRGLSEETVREVRHAYDLRPVYKTVDTCAGLSESNSSYYYSAYDTQTEVAPRTRPAVMILGSGPNRIGQGIEFDYSCVHAALTLKGQYETIMVNCNPETVSTDYDTADRLYFEPITFEDVLEIYQAESQVGPVVGVIVQLGGQTPLKLARELEAAGLPIWGTSPSAIDLAEDRGQFGQVLLDGGLAAPAFGSASGLRQALQVADTVGYPVLVRPSYVLGGRGMEIVYSPDQLTDYVRRTVPGAAQSIADRGQARFPSPVLIDHFLDEAVEIDVDALFDGTDLFVGGIMEHIEEAGIHSGDSSCILPPVTISSQMITQIREATRRIAEGVGVRGLLNVQFAISSDVLYVLEANPRASRTVPFVAKATGVPLAQAAALIMSGRSVAELKQTGVLPQADPTEGSWTGPLAVKEAVLPFKRFRRPDGRVVDNILGPEMLSTGEVMGLDANLPMAFAKSQAAAYGGLPTGGTVFVSVADRHKRSIVFPVERLHDLGFEILATRGTAEVLRRYAIPVREVARLSESGPDSELDNIVKLIVDGQVDMVVNTPTGQEDSRSDGYAIRNATTSADRPIVTTIQQFGLAVLAIEAVQKGNFTVESLQEYEQRRALPSRES
gnl|extdb|pgaptmp_000884	K09760	rmuC; DNA recombination protein RmuC	MSLESILLVGVIVLVAVLIVLVLRRGGDGADGGRLTQLVEDTARLEQQIVTSGQMQGQRTVELQQVLDARLRDQVGQNRDEARQRLQADAAARLELQEILTGRLDRMDRSLGELRDSLTKGLDQLRTTNAAELERIRLEVAEKLQASLAQSLKENSEQIEKLTETSSKRQDELRDALRLELDKVRTQNDEQLEKMRLTVDEKLQGTLQKRLGESFQLVSDRLEKVQRGLGEMQTLASDVGGLKRVLSNVKTRGTWGEVQLSRQLEDVLSPSQYEENVAIDPQSRERVEFAVRLPGKSEDSPVYLPIDSKFPQESYERLLSAQEAGDVVEVERATKELEQAIRLQAKTISTKYIHPPLSTDFAIMYLPTEGLFAETVRIPGLASSLQVNERVMITGPTTLMSLLGSLQMGFKTLAIEKRSSEVWQVLGAAKAEFAKYGQVWERLGKQLQTAQRTVEDAGRRTRAVERKLRDVELLEVEGEAEDLIDEVLLIEE
gnl|extdb|pgaptmp_000909			MITEALSALTGRRVAVLTGAGVSTGSGLPDYRGQDAPPRTPMTFQQFISEERYRRHYWARTYLGYQHMNERRPNVVHEALARWEYQPGSALVGIITQNIDTLHEKAGSGGRRPVIDLHGRFDRVLCRQCGHRFTREFVQGMLAQANPGFHELVGDVEIAPDADAVLEETDDFHLVVCPRCGGTLRPDVVFFGEVVPPARVAAATALVDEAEALLVLGSSLAVHSGLRYVTRAEGKPIVIINRGPTRGDRRASVKLDGDLAELVPPLLERLAPL
gnl|extdb|pgaptmp_000747			MHAEEIGDEEFEALVQDALDRVPDEFRDQMENVAVVVDDEPPAGQHLLGLYEGVPQTEEGDYPWQLPDVITIYQGPLVRMCADRDELAHEVYVTVVHELGHYFGLDDERLHELDWG
gnl|extdb|pgaptmp_000746			MSKPRVTLATAAQKPNLYPGETGLLDALASRGVEPRIAVWNDPSVNWDEAGLVVVRSVVDYATDREGFLKWADSLPRVLNHADILRWNSDKHYLMELEKRGLPTIKTTWLSAEKGYTKHQVHSRFPAAGDFVVKPAVSSGVRDVGRYSAINIPQRQAAMEQVMRLLRTGRDVMIQRYQEEVELLGERSLVFLNGLLSHTVDKTALLSRDQVTGQNVQTVDVSARAATDEELRWGEDIRVVLHAYVRERMGRDEQFLFNRVDIVPDGKGSFFVMEVALVDADLYLGSTNRALDNFADAIT
gnl|extdb|pgaptmp_000745	K01921	ddl; D-alanine-D-alanine ligase [EC:6.3.2.4]	MSFSDRPVVLVLFGGQSSEHEISCATAAGVLEAIDQTRWEVIPVGITRDGHWVPQPNDPARYRLGETGGYEVLSQGEQVSFLSGTTSDGHPRLVYFQVDEEGRPLAESLHPGPVVDVVFPLLHGPFGEDGTLQGLLELSGVRYVGCGVSASAVAMDKRLTKTVLEDAGIPVGRWEGVSARQWQADREGVLARLDRLGLPVFVKPCRAGSSLGITRVTDAGELAAAIEAAHEHDPQVIVEAAHAGREIECGILQLPGGELVASPLGEITVTDAQFYDYESKYFGQGGVQLSCPAQVNHAAAIQEAARVSFEALGAEGLARVDFFYQEETGSYIVNEVNTLPGFTPYSMYPTMLAQAGYPYPKLVATLLEEAMSRPVGLR

gnl|extdb|pgaptmp_000744	K00057	gpsA; glycerol-3-phosphate dehydrogenase (NAD(P)+) [EC:1.1.1.94]	MSKQRAVVLGTGAWGTTFAQVLAHAGMNVTMWGRSEDTVSFINDGENSRYLPGIELDPAISATTELDEALRPDGENPALVALAVPTSAIVSVTQEAELTEADTLVLSLAKGLEPHTYRTVQQMIAEEGQIAPERIAVLSGPNLSREIADQQPAAAVVASDSVETAKQIAIWCHNPYFRPYVSTDVIGVELAGASKNVIALAIGASEGMGLGSNTRATLITRGLAEMTRLGVALGANPQTYAGLAGVGDLIATCSSKLSRNYSLGFRLGQGMSLREALDLSPGVVEGVATSAPVLELAQSCGVDMPITAGVVAVLSGKATVQQMGESLLARPQKMDGWEVELLD
gnl|extdb|pgaptmp_001927			MKKLKLTVGLAAASLALAACSQTPTPAQSETTPAPTPVESTQSDAPQSEAEGASPAPSASPAESESSTTPIQGDRWVNEIVPVEPDYDRAAGSNLLIHDVRVGEHPGFYRVVFEFVGDGTPGWHGSWSDTVPVEQGRGEPLSVTGSTFLDLAFTGVQMPMDDADYEVYYQGPDTLSVGPIEIDVDGTFEGQLHVGLGLDQQRQLQIATLSNPTRVVLDIKN
gnl|extdb|pgaptmp_000743			MSQLAQIRKLGPLYQLAYALLTPILGPLTRPHWSGQAPQGGAILVSNHLSNLDPLVLAYAFGVRGHEVRYLAKAELFKVPVLGSILTRWGMVPVQRGSGHAADALDQAAIAVQSGQLISIFPEGTITQDPAFWPMKMKTGAARLALATGQPLVPVLLWGTQDVMDRKSPWLRLRRTDVYIHVLDPIDISDLPADPANHEVVTEVTGRIQSALRTAVSQLRGQDAPERIWDPKSDEHSEASVKKFSSWRRELARKNGRQDILPGRR
gnl|extdb|pgaptmp_000020	K07029	dagK; diacylglycerol kinase (ATP) [EC:2.7.1.107]	MTLLVSRISARGRAERQGREALRILRAAGWDVTVQLTDLSDIPADLAEAAGGPLVGAVGGDGYLSAVARGVVNGGGGILVPFPAGSGNDLCRTLGLGVDPVARARSLAEADEAELAQRVQRLDGMWVRTADRDERQLALGVVSLGIDATANLVANRSWIRHGPLSYTWGGLHAFFSHRRGLVSGTVDGREHDFSGWIASVSNTGRIGGGIQLVPSSDPSDGMLEVFNVGNISRWRALPLLARVLSRRAPDSPLVKLFRGVGVTIEADPSLPVMADGDLLGHTPVRVDGAPRLVRVLV
gnl|extdb|pgaptmp_000742	K00790	murA; UDP-N-acetylglucosamine 1-carboxyvinyltransferase [EC:2.5.1.7]	MNKILRVEGGAPLEGAIEVRGAKNFVPKAMVASLLGETQSQLRNVPQIRDVDVVSDLLSAHGVKVQYDAERGVLDLDPTNVELAARSDIDALSGSSRIPILFCGPLLHRLGEAFIPELGGCNIGGRPIDFHLETLRRFGAQIDKRPEGVYITAPRGLRGTVVELPYPSVGATEQTLLTAVQAEGITTLKGAAVEPEITDLINVLQKMGAIISVDTDRTVRIEGVDRLRGFEHTALPDRIEAASWGAAALATHGDVFVRGAHQPDMTTFLNTFRKVGGEFEIRENGIRFYHPGGSLKPIVLETNVHPGFMTDWQQPLVVALTQADGLSLVHETVYENRFGFTSALRKMGANIQLYKECVGGVPCRFGQRNFYHSAVISGPTPLHGADIWVPDLRGGFSHLIAALAAQGVSNVSGIEVISRGYEHFMQKLRQLHAHVEYV
gnl|extdb|pgaptmp_000741	K01704	leuD, IPMI-S; 3-isopropylmalate/(R)-2-methylmalate dehydratase small subunit [EC:4.2.1.33 4.2.1.35]	MEKFTEHTGVGVPLRRSNVDTDQIIPAVYLKRITRTGFEDALFAAWRKDPNFVLNDPHYQNGSVLVAGPDFGTGSSREHAVWALKDYGFKVVLAPKFADIFRGNSGKQGLVAGVVSNEDCETLWKILESNPGTEVTVSLEDRTVRAGGATFPFQIDEYTRWRLMEGLDDIGLTLREADAIDAFEAKRPAWKPKTLPVKTAS
gnl|extdb|pgaptmp_000740	K01703	leuC, IPMI-L; 3-isopropylmalate/(R)-2-methylmalate dehydratase large subunit [EC:4.2.1.33 4.2.1.35]	MGKTMAEKVWEEHVVKHGEDGVPDLLYIDLHFVHEVTSPQAFEGLRLAGRPVRCPDQTIATEDHNTPTKDIDLPIADLTSRLQIETLRQNCAEFGVRIHSLGDIDQGIVHVVGPQLGLTQPGATIVCGDSHTSTHGAFGALAFGIGTSEVEHVLATQTLPLQPFKTMAITVNGKLKPGTTSKDIILAVIAKIGTGGGQGYVLEYRGEAIRDLSMEARMTICNMSIEAGARAGMIAPDDTTFDYIKGRPHAPEGAEWDEAVAHWRSLVTDDDAVFDAEVILEAEDIEPFVTWGTNPGQGVKLSESVPDPEDMKDPIDRATAHRALAYMDLEPGTPMRDIAVDVVFLGSCTNGRIEDLRMAADVLRGRKIADGVQMLVVPGSARVREQAMAEGLDQVFLDFGAEWRNAGCSMCLGMNPDQLTPGQRAASTSNRNFEGRQGKGGRTHLVSPLVAAATAVRGTLSSPADLDPVPAN
gnl|extdb|pgaptmp_000739			MDGVTDSSGVGVLDKAALVLGALEAGPATLAQLVAATHLARPTAHRLAVALEHHRLVTRDMQGRFVLGPRLQELASAAGEDKLISSSMPILIALRDHTKESAQLFRRQGENRVCVASAERQVGLRDSIPVGAALTMKAGSAAQVLLAWEEPERLHRGLQGASFTATMLSQVRRRGWAQSVSEREAGVASVSAPVRGGDGRVIAALSISGPLERMGRQPGRVHGPAVVAAANRLSEFLSTAEKMGL
gnl|extdb|pgaptmp_000723			MRIVHVTDCFLPMMGGIETQVSQLAAQQAQRGDQVTVLTCTPGEVELPYRVLRSVWANPVHAPVDPRAPRRFHQVLERLNPDVVHLHLGELTPVVQALLWRLRNAHIPTLVTVHSVWNRRVTLPIYRWLGKSFRDAHIGWTGVSHLVAGLVAEAVGAENVQVLPNGVDGSRWRVEPEPHEGLVAVTAARFAPRKRIPALLEILREAAGEVEPGSLRAVLAGEGPGFESAQSFVERHGLEDVISLPGRLSAPELRRLYAGADVFVSPSINEAASIAAAEAQAAGLAILTRSQSGLGERIGPDEGVRAQTDAELTQTLVEWTRQPAQLQAIREHNRGTVCPLDWQVVMPQVDSAYKWAQERAGRNLPQAAE

gnl|extdb|pgaptmp_000718	K26364	mimosinase [EC:4.3.3.8]	MSIFEPSVAHLRETGSLKWTGVSAPGDRPAQGAWVAEMDFGTAPVVAERLKKAIGDGMLGYLPYWLEDATAEALTRFQEARYGWAIEPSWVRTANSVLG<br>ALEATISHLTRPGSSVIVPTPAYMPFLTIPGRHNREVIEVPSLHTAGQSCPDAWSLDLAGIKAGLEAGAGLVILCNPWNPTGRSLRTEELVALHDLVSQYDAM<br>VFSDEIHAPLTFADTPTFVSYASLGPHFAAHTVTAVAASKAWNIAGLSAAQVILPDRELREQWDQKASAISRSAVPLGLLGAVTAYESGSDWLEEVLAYIGRN<br>LDLLDEVVAATAVDYTRPEATYLTWLGMDAYDLDQCPQSLLLRDYSIAANAGETLGAPYCQWVRLNAAMVRPEWERTMDLLAQALKAWPLKG
gnl|extdb|pgaptmp_000717			MRIVRFSDGDNPRYGMMQEDSTRIFVLRGDPLFGKIEATGEVLDLDEVRLVSPVIPRSKVVAVGKNYLDHVREMGGTEAAAEPILFMKPNTSVIGPDDPIVL<br>PRWSERVEHEAELAVVIKTLAKDVPVDQVDDIILGYTAANDVTARDKQKQDGQWTRAKGFDTSCPLGPWITVDPDLDVDNLLITSSVDGQLRQSASTADM<br>IVPVRELVSFISGVFTLLPGDVILTGTPAGVGPLEPGDRVDVTISGIGTLSNPVVRR
gnl|extdb|pgaptmp_000716			MKAPLNELLDQLEDSGEVDSPDALFDAFSQWADSTGRPLYPHQEEALLELLAGNHVVAQTPTGSGKSMMALAGHFLSLARGGRSYYTAPLKALVSEKFFDL<br>VEAFGAANVGMITGDISLNSKAPIICCTAEILANQSLREGAALDTDLVIMDEFHFYSDPQRGWAWQVPLLELTKPQFVLLSATLGDMSELAEDLAERTERTVG<br>MVTEATRPVPLEFEYCLDELPVVVERLLAEDRAPIYIVHFAQREAVNTAKGLQKLSILSKEQKAELKEALRFESFGRGFGQTLRELVLHGIGVHHAGMLPRYRR<br>LVERLTQRGLLAVVCGTDTLGVGINVPIRTVLFTSLIKFDGRKTRHLSAREFHQIAGRAGRAGYDQVGYVRVLASEAEVEQAKHRARLSAAQEEANTKKLKKL<br>AKKTSPKSSTPGKISWTKGTFERLVSAEPEVLRSQFAVTHAMFLNVLAGPGDPEARLLALAQDNHDPDQDANEHLRSLGDIYRSLRQAGVITRLPHAEAQRQ<br>GVPRLQVVADLPDEFALNQPLAPFALAALDLLDPDSPDFTLDIISVIESVMEDPKPLLFAQERRAKDAAFHSMKAEGIEYDERAALLDQVTWPRPLAELIEPTF<br>RVFSQTNPWVGEAEPSPKSVLREMIETAATFTGLIAKYDLHNAEGVILRYLTDLYRALRQIPPLSCQTPELAEVIDWLEKLVRSVDSSLLDEWERLNRGETSPSA<br>GAGSGAGAGQPQDSGMEPAFGADPDGTVTYRTNPHLLQRDVRTAVFRLVEQLADDRVEQVASYGSDTGWDADRVDRALAQYWAEYDWMGIDHRAR<br>SAELFRVRRNPAVDEVERAVEEGGAQLEGSTDEDRWWLIDQVVLDPNEDGDWRVTLALDLWKTREEDRPHLLLLGFGPH
gnl|extdb|pgaptmp_001537	K01687	ilvD; dihydroxy-acid dehydratase [EC:4.2.1.9]	MPRQLRSATSTQGRNMAGARALWRATGMGDDDFGKPIIAVVNSFTEFVPGHVHLRNMGKLVADEIHRVGGVAKEFNTIAVDDGIAMGHSGMLYSLPSR<br>EVIADSVEYMVNAHCADAMVCISNCDKITPGMLLAALRLNIPVVFVSGGPMEAGKNIPADAIIGDSQTGHGNLITVMNATADDSISDEKLLEIEKLACPTCGS<br>CSGMFTANSMNCLTEALGLSLPGNGSTLATHVARKALFEEAGRLIVDLCQRYYNEDDDSVLPRAIATKDAFRNAMALDMAMGGSSNTVLHILAAAHEGEV<br>DFDLEDIGEIGARIPVLSKVAPNHNDYHMEDVHRAGGIPALLGELRRAGLLNRDVHTVHSPDLDSWLDRWDIRAENPSQEALDLFSAAPGNVRTTEAFSTN<br>NRWAELDTDAANGCIRDLEHAYTKEGGLTVLRGNIAEDGAIIKSAGVDPSLFRFQGRARVMESQEEAIEKILDKTVEAGDVVVIRYEGPAGGPGMQEMLYP<br>TSFIKGRGLGAKCALITDGRFSGGTSGISVGHISPEAAAGGAIGLIEDGDEILIDVNRGLLELLVDDAELERRRAEQEAREHPWTPRTRERHVSPALQLYAATAL
gnl|extdb|pgaptmp_000606			MSHQARSSAKKSRPTWLKVLAVIVGLLLLVVLAAAIAVFTFGKRVADTYDSGVTVVEEAFPDEADRPQEQDTKAQTILLLGSDTRSAIDPDDVNAAQDSRSD<br>VIMVLRIPADREQAFLVSFMRDSWVDIPGYGEAKLNAAMAYGGVPLTVQVIEGLIGSRIDHVAMVDFAGFKGLTNALGGVQVQNANEFSTGDFHFPAGTI<br>ELDGTEALAYVRARYPFADGDYQRVRNQQAYLRGLVSSLVSRGTLTSPGKIQDAVAAISPYLTVDPGMDSGYLLKLLPSMRNIRTADLEFFTAPTAGTGTSAD<br>GQSIVVLDEERMAKLKQAFETDTLAEYVETQDLSAN
gnl|extdb|pgaptmp_001538	K01809	manA, MPI; mannose-6-phosphate isomerase [EC:5.3.1.8]	MLRITGFPQHYAWGSHVRLPEFLGTSPSPEPLAELWFGAHELGSATTATGERLADLIATHPREYLGPSTRFMFGDRLPYLMKLIAPAAPLSLQVHPNKRQAE<br>AGFSAEEEDGIGRSDPQRIYRDDNHKPELLYALTEFTLLAGFSVRRQVRELLDGLEVPLAYRLGRRLRLAAGRAMKPVVSWLLDPDSAPDRAEIDQFAQACSE<br>RLEAGRSPLPLLDSTVRYLAEAYPGDPGVIVAFLMNPVRLEPGEAIYLPPRTLHSYLDGFGLEVMANSDNVIRAGLTPKHIDRTQLVEVGQFDAHPPVRLAPEY<br>PSEGIRRYYAPVEDFELSVVSLDAQTMPLLGTGPRLVICLQGSPTVMTRAGELELRRGECVFVTASEGPMLSSGTGVLAQCAVP
gnl|extdb|pgaptmp_001684	K00368	nirK; nitrite reductase (NO-forming) [EC:1.7.2.1]	MVEKQKVTVAQGASAVLGIVIVAVLALAAALISPSLVSGTGSEPAAGSGSAPASSASSATGQTTEVTVTVDGMRFVPGTIEVPAGNNLVVNFENTGDQRHD<br>LVFANGVATEALAPGASARLDVGVITSNLDGWCSLPGHRQMGMVLEVVATGSESETTDQMGMGHEGMDHSATASAMPTMADLMDEATKHDPYPAR<br>VEPLPPADGPQTREYTFEVVESEEDLGAGIVRPLWTFNGTGPGPILHGRVGDEFVITLVNNGTMGHSIDFHAGEIAPDEVMRTIEPGESLEYRFTAGRSGIW<br>MYHCGTMPMTLHIANGMFGAVIIEPDGLEPVDQSYVLIQSEYYQDEAGNTAADKLNTMIPDVAMFNGRAFQYDVHPLTAKVGDRVRFWVLDVGPNSPL<br>AFHIVGTQFDTVWSEGHYSVHHGQSTDGLTKGVTGAQVLPLQAAQGGFVELVAPEPGHYAIVNHIMTLAEKGAHGILQVE

gnl|extdb|pgaptmp_001685			MSIPLSGGATGPTGPGSQPSNRPRRTRVDRITTMWMILAALILAYSFVFGGQLNQQWWTTVHLITLGVITNAILQWSWYFSRSLLRLPPSDKHSGAHQTARQVLFNLALVELVAAMWLASPVGAVIGATAIGLIVAWHVVALVLAGRSALAARFAVIIRYYTASGAFLVVGTIYASLLTIVLLSPDPPAELVRIQGGLTVAHALVNGLGWVGLTIAGTLVTLGPTALRTRMADGAVTRAVQILPVLIAALLVATVAATFGLLALAGLAILVYAAALVWGIGLPLLQVAVRKPLAEYASWNFAAGILWFAVGLVWLAAELAWAPGADAFRESSRLVVGILGVGGVLQILIGALSYLLPVVVGGGPTPVRVGNATLQVAGGLRLAVRNAGLLLAAFAGLSGVESTLLTGIWAGLIIVSFLGDIAAMGAAGVRQAKAKREPTHEGGIRG
gnl|extdb|pgaptmp_001686			MKKDSASNEVEQPRPTPVHPPAATPRVPRGRLLLLAGGGLGLLAGLNAALLRLGLAAPVQSDSLASLHGILMLYGFLGTAITLERAVALQSDREAFTVWAYLSPAASALAVLAALLGLAQPDLFGSHLGPGLLWTLSMVTLVAIYLVIWRRQQTVFLLIQILGAVAGAVGVALWARGLEIPLVLPWWAAFIVLTIVGERLELARIAFAGGTTEMRVLLESLLYFLALVATLFTPAWGYPLMGLALGLLMLDVGYHDIARRTVHTAGLTKFMAASMLAGYGWALLAAGIWVVRGPVLSGYGYDTVVHALTIGFGLSMVMAHAPVIVPAVVRRPVPYHPIMWAIWALLQGGLLVRVLGGARATWGQPSAEVAWQFGGAVDVLTVLVFVLSTLTLIIWHGRKDRTR
gnl|extdb|pgaptmp_001539	K00826	E2.6.1.42, ilvE; branched-chain amino acid aminotransferase [EC:2.6.1.42]	MTESTVRQLSELEEAAAVPVPNADEVASRFPVSQNPTPASDEERAQALRDLDFGTTFSDHLAIEAWNPEEGWHGRSVEAYAPLALDPSAAVLHYGQEIFEGLKAYRWADGSVWTFRPTFNAARMNQSAVRMAMPSLPVEDFLGSIVQLVRVDANWVPSIPDSALYLRPMMIASEPFLGVRPAREYLYLVIASPVGPYFKGGLKPVSIWVSTDYHRAAPGGTGAAKTGGNYAASLLPQQLAAQQGFDQVCYLDAATNSVLEELGGMNVFVRRADGTVLTPALSGSILEGGTRSAIMQLLADRGVPVTETALSLAELVAGIEAGEITEMFACGTAAVVTPIGRIAGDGFDFTLPGTELTAAIHDELGGIQRGLVPDRHGWMYRLV
gnl|extdb|pgaptmp_001541	K00053	ilvC; ketol-acid reductoisomerase [EC:1.1.1.86]	MAEIFYENDADLSIIQSKKVAIIGYGSQGHAHALNLRDSGVDVVVGLRPGSASWAKAEAAGLAVADVPTAVGQGDVVMILVPDQHQRGLYADQIEPNLKPGAALFFAHGFNIRFGYINPGPEHDVCMVAPKGPGHKVRETYLEGKGIPAIVAVEQDASGQAWELALSYAKAIGATRAGVIKTTFTEETETDLFGEQAVLCGGVSQLIQYGFETLTEAGYQPEIAYFEVLHELKLIVDLINEGGITKQRWSISDTAEYGDYVSGPRVIDPHVKENMKGVLNDIQDGTFARRFIADQDNGGQEFKALRAQGEGHPIEKTGQELRKHFGWAQQDSDYVDGSAAR
gnl|extdb|pgaptmp_001542	K01653	E2.2.1.6S, ilvH, ilvN; acetolactate synthase I/III small subunit [EC:2.2.1.6]	MENLHTLSVLVDNRPGVLARVSGLFARRNFNIKSLTVGETENQRISRMTIIVDADQVPLEQVTKQLNKLINVIKVVEMGPRESIERRLLLLKVNADQSCRTAVLQIVDLFRAHVVDVQTETVVVESIGSRDKLEALLAALEPYGVRELAQSGAVVIGRGPKSITDQIKEKY
gnl|extdb|pgaptmp_001543	K01652	E2.2.1.6L, ilvB, ilvG, ilvI; acetolactate synthase I/II/III large subunit [EC:2.2.1.6]	MKDGDRAVTQQETITGAEAIVRSLECLGVTEVFGMPGGAILPTYDPLMDSSIRHILVRHEQGAGHAAEGYAVSSGRVGVAIVTSGPAALNTMTPLADANIDSVPIVVITGQVAAPLIGSDAFQEADVVGASMPVTKHSFLVTEADDIPARIAEAFEIASTGRPGPVLVDIAKSAQTAQCNFQWPPQTDLASYRLPTKPSMKQIKAAAKALCEAQAPVLYVGGGMTRSGASDQLTKLVELSNAAVVTTLTARGAFPDSDPHNLGMPGMHGTVAAVGALQRADLILALGSRFDDRVTGQLDSFAPRARIVHVDIDAAEISKNRTADIPIVGDLNRVLELLVEQLPHAQEKYGAPDLSGWWRYLNRLVSQYPLGWTEPSDGELAPQYVLQRLSELTGPEAIWATGVGQHQMWAAQFIDFEHPRHFVSSSGLGTMGYGVPAAMGAQVANPDSVVWCIDGDGSFQMTNQELATCVINNIPIKVALINNKVLGMVRQWQNLFYDQRYSHTDLAEGGGEQVPNFCKLAEAYGMPSRRVTKAEEVDEAIKWALEINDRPVLIDFSVSKDAMVWPMVAAGVSNDDIRYARGMAPQWDGEE
gnl|extdb|pgaptmp_001544	K07085	putative transport protein	MVAIFEFLESQQVLLVFLLIGIGMAFGHIKIKGVSLGAAAVLFAAIGLAAWGAHLGVDLRVEAWIGHLGLAVFAFAIGINSGASFFHNLKTALGPILAMIVLFAVAAGAAWGLGHYVFGLDSAMIAGTYAGATTNTPALGAAGEASGNPEAATVGYAIAYLFGVIGMMGASLAALSYAKHDKDAPSPLANRTIRVERNDGPFLGDIYDKFGGKVTFSRLRRGETGPITRPSMSDTLGVDDLVTVVGPEELVAKVAKELGHSSSHSLMKDRSYLDFRRITVSDPKIAGKTVGSLEMSKKYSATISRVRRGDIDMVGEPDLVLQQGDRVRVVAPTSKMEAITKFFGDSARGLSDINPVAMGLGIALGVAIGVIPIPLGGGSTFSIGAAAGALIVGLIFGKIGRVGNVVLAVPFTACQVLIEFGLLLFLAQAGSNAGGQILDAFAGGTWAKLLILGIIITLIMAVGLYAVMRWGFKMGGTKLAGLVGGAQTQPAVLAFANGRTNSDPRVALGYALVYP
gnl|extdb|pgaptmp_001548	K00027	ME2, sfcA, maeA; malate dehydrogenase (oxaloacetate-decarboxylating) [EC:1.1.1.38]	MAYSPSYTASYRLLVDQSKIRVSDIVDRVSRTGAFVKGLDVAESEGSQISIDLTADLRDSDHRRSLTHALKSLDGLTVSSVADQTFLDHVGGKLEVVPKAPLRNRDDLSRIYTPGVARVCTAIHDNPSKAHFLTMKANTVAVVTDGTAVLGLGDIGPEAALPVMEGKAALFKHFGGVDAWPVALDTKDTEEIIAIVKAIAPAYGGINLEDISAPRCFEIERRLREELDIPVFHDDQHGTAIVVLAALINALKVVGKRIEDVRIVSGVGAAGNAIIKLLLAQGAKDIVGYGRSGALAGDEVEGMDPDRRWLAEHTNPRLVHGTLQEGLAGADVFVGVSRGGILSGEDISTMAEGAIVFALANPTPEVEPVEAAKHAAVVATGRSDFPNQINNVLAFPGLFRGLLDARVQEITTEVLRVSAEAIAEVITPEELSANYIIPGVFDDRVARNVAKAVKRSVANLDAIEVVPQEDLGEPLTR

gnl|extdb|pgaptmp_001550	K02433	gatA, QRSL1; aspartyl-tRNA(Asn)/glutamyl-tRNA(Gln) amidotransferase subunit A [EC:6.3.5.6 6.3.5.7]	MNPLLKESALELAAKLRAGTITSVELTQACLDRIGELNGYLNAFITVDEAGALAQAAEVDRRRAAGEELHPLAGVPIAVKDNIVTHGLETTCSSRILAGWIPPYDATVVTKLRAAGLPILGKTNLDEFAMGSSTEHSAFGVTRNPWDPERIPGGSGGGSAAAVAAYLAPLALGSDTGGSIRQPGAFTGTVGVKPTYGGVSRYGLVAMASSLDQIGPVARNVADAAALQELIGGHDHLDSTSLPEPVPAVAAAAEQARANRDASGLRIGIVKELSGEGYDAPVLAAFERTVDQLRAAGAEIIEVSCPSFDYAMAAYYLIMPAEASSNLARFDGMRYGLRVEPTDGPVTAESVMAATREAGFGDEVKRRIILGTHVLSAGYYDSYYGAAQKVRTLIQRDFDRVFAECDVLVSPSTPTPAFHIGERIDNPLAMYLNDAATVPANMAGVPALSVPNGRTEEGLPVGFQVVAPAKEDALMYRVAQLVEDLSEDISAHCPAADWSANVGEGE
gnl|extdb|pgaptmp_001551	K02435	gatC, GATC; aspartyl-tRNA(Asn)/glutamyl-tRNA(Gln) amidotransferase subunit C [EC:6.3.5.6 6.3.5.7]	MSKFSADEVARVAQLARIALTEEEIERLAGELDVITSAIDKVSEVATDDLPATSHPLPLTNVMRADVRGPVLNRDEVLAAAPASEAGQFMVPQMLEED
gnl|extdb|pgaptmp_001552	K06131	clsA_B; cardiolipin synthase A/B [EC:2.7.8.-]	MLDRLRKARVPGGVTGFPALHPSDTAVSEDVVRTYTEGQSLYHDMLTAIRGAKELIFFETYVWRADLSGQEFKTALIEAAERGVEVYVIYDGFGSFLQSPRFKMFPRIPTLHVHKLPEIRFGLLTLNARRTGRTHRKLLVVDDQVGFIGGFNIGDDFGTEWRDTHLRVIGPSVTELSDGFVTFWNFLRRRHEPELEDRYASPWMAEITTAFNLPSRLLFPIRGLYLEAFDRAQDHIYLTTPYFIPDREFTAGLLAAVHRGVQVRILIPEYSNHILADWVSRPYLGPLLRGGVEIWLYQHAMIHSKTMTVDGIWATVGTANIDRLSMMGNAEVNMQIVSPEFAQRMEEAFNNDLTTARQLTLAEWEQRSWLTKFTEVLLGPFNPVV
gnl|extdb|pgaptmp_001553	K01972	E6.5.1.2, ligA, ligB; DNA ligase (NAD+) [EC:6.5.1.2]	MSETVVPPEAAARWRDLADLIEEARHRYYDLDQPTLADAEYDAAFRELTELEAQYPQLASQDSPTATVGGAAQSTFDPVVHREQMYSLEDVFSLDEVAAWQRKMADNWPGEDLAFTAEVKIDGLAVSLTYQNGRLIQAATRGDGKVGEDVTANVRTISSVPARLQGSGWPELVEVRGEVFFLLKDFESVNDQRLAQDERPFVNPRNAAAGSLRQKDPAVTASRPLSMFAHGIGYVEPGPGFVAPTTQEGWYQQLRNWGLPTSPYTQVVHTVDQTRALIERVGSERAQIEHEIDGIVLKLNDLDQQRELGFTSRTPRWAVAYKYPPQEVFTRLLDIQVQVGRTGRVTPFAIFEKVLVAGSHLQHATLHNEEMVRRKGILIGDKVIVRKAGDVIPEVVGPVLEDRDGTERPFVMPTRCPSCGTELAPAKEGDIDLRCPNAGGCPAQITERLAHLGSRGALDVEGLGDEAALALTQPEAGRDEVIAALATGATLTLEDGTVLSLAQPELIPHGERFDRAAQLLPPPTGPVLTSEKDVFDLRVEDLADVLVWRPVNVKGVPTGDWRQVRYFWSQPWRKQGKKFVPVQSVPRKNTVTMLEQLEAAKTKELWRLLVALSIRHVGPTAAQALAQHLPSLDRIEAATREELAQVEGVGQVIAESLEGWFAVDWHREIIDAWRASGVRFADQQVEELPQVLAGLTVVVSGAMPGFDREEAKEAIVARGGKAASSVSKRTAVVVAGPGAGSKVAKAEALGVPVLTEEHFADLLARGQVAIEEARANQS
gnl|extdb|pgaptmp_001554			MTEDLSPARALTTAHHLACVSGPDRGLVLPLVDGATLGRQTVPTWSDPSVSRLHARVRLGGNTRRAVGWLVLSSGREVPLRPGRKVQLGAGQWQLRARPTNLSWPSPTLRRAGGVRPWLRWLPLLLIGWWIWRYLPLSPTVLALAVGGALCLGLVGRWAWQLRRRHRLDGAFLALVWAANRSARSRDGPVEPVAVWTGSWGRGKLGLAPGEAVGVIGTDAGVAARWLAVQALVQRGSLRLDRAAGDQAKCDQTSGYQANGDQAGTLVLSDERGQIRLSWAPTVGELPEAPARLVQVCAPVGPRWLEQLGRAETAPSSLPSTVSATDLGLDLSVEATASRWRDFTWDWAVPVGLSPDPEPTVFTLDLPGQGPHALLAGATGSGKTVALQTWLWSLAIHVPPSSLRLILIDYKGGAGLQPLLTLPHVEVAVSDLEVDRVAWVIRRLRLLLQERKAQLRAAGYADLRQWEAAHQEGLAPLAPARFLVVIDEFQALAEDQAELLGAFTRLATQGRSLGLHLLVATQRPGHAISADLRAAIDLRIGLRFAEVADSVAILGDGRGAELPRNPGVAYCGPKLLQFAQLGPLPLPEVPPGPRTNWPVPLPTVLVSGPDTPRMELGVREGNPELVRVSWVGGALAIAAPRSAQAEVQTLAHRYGAQLAQESGSALTIVSTGAEGTVDPSNLADLSSVLLALDRGVPAVLVVDDLAAVSQEFDLHGLGLEFAHLWRSLVQRAGPPIHRIVAVDLDGTPAGRALTDRLFRLPTREALLEPALLRTLPQLDPDGTGVALSRGEAVSPTGGRLVALGQGFRPETPGPFLLQTFTRWDRTQPPAAPPLHSPWPTRSAQFSWREGPQLVATTPRPGPLATWQLPPGAGTWLTQLGFTVTELSQWTGVLTLGSTPFLALEPPLELLRSLANRSPGAALWLRAHYPYPPGCGVTNVDGTLSLVTILAPNTP

gnl|extdb|pgaptmp_001555	K18955	whiB1_2_3_4; WhiB family transcriptional regulator, redox-sensing transcriptional regulator	MDWRSKAACLSVDPELFFPIGNTGPAIAQAAEAKRVCATCEVQEVCLQWAISNNQDAGVWGGMSEDERRALKRRAARARRNS
gnl|extdb|pgaptmp_001556	K00936	pdtaS; two-component system, sensor histidine kinase PdtaS [EC:2.7.13.3]	MRTLGELVERSSSLQLGQADLDWLHLLMADWQVLADLAASDLVLWLPDSDGGFVAVALCRSGTSSTVHLDDIVGLHAPATRAEMLGEAFTSGRIVQSNDIRWAGSYSLSASYIPIAHQERIIAVLSREGNFSSFGNSSNHAKWTTQAADALCEMITLGEYPYESSPSMASRGGPRVNDGAILLSADGAVLEATPNANSCMRRLGVGPHLEGKVLAEHLTSVIRNHHNVDETLAVVAMGRASWVVEVEGHGATIAMRALPLTRSGRRVGAVLLTRDITETARREQELMTKDATIREIHHRVKNNLQTVSALLRMQSRRSDSPEVKQALYEAGRRVEAIATIHEALSHNVDEIVPFDDVAESILRMAAGVASTSHHVDVQVTGKFGLVPADAAAALATVLTELVTNSVEHGFPDRGGEITISAERCGDDLCVVVADNGVGMTEGSALGGLGTQIVNMMVTGELKGDISWDPGPDGGTVVTLRLNVERG
gnl|extdb|pgaptmp_001558			MQFSEKYEYPAGFDQIWEMYADPEFHAQRLSAAQLEEATVNAHLDGDDLTVELRGQVSPEAIPAQVSRFVKGRLSLSLTETWHRTGSEATGRMTVDISGAPVKIGAELALTSPAAALTERAMTGDLKVSIPLLGPRLEKEAMRFIPMLVNAELSAAEAWLSSH
gnl|extdb|pgaptmp_001559			MSDSNTPTGLFIERVGTRQYAGTNDRGATISIGHGEGQWSPGELLKLALLGCNAMSADSRLARSLGEDFQMGAVVDAVYNKDEDRYESMSVELIPEFGDADEETVAKAKKYGLLGIDRYCTVGHTLDHVVPHTATITKED
gnl|extdb|pgaptmp_001560	K00560	thyA, TYMS; thymidylate synthase [EC:2.1.1.45]	MSPDRQYEDLLADVLAHGTRKEDRTGTGTLSVFGRQLRYDLSAGFPVITTKRVHLKSVIGELLWFLRGDSNVCWLQEHGIRIWNEWADENGDLGPVYGVQWRSWLGADGETIDQLSEVLDTLRTNPDSRRMIVSAWNVGQLSQMALQPCHAFFQLYVADGKLSLQLYQRSADLFLGVPFNITSYALLTHLLAAQANLEVGDFIWTGGDCHIYLNHLDQVREQLSRDPYPFPQLQLAQAPSLFDYDFDDIEVVNYQHHPAIKGAVAV
gnl|extdb|pgaptmp_001561	K00287	DHFR, folA; dihydrofolate reductase [EC:1.5.1.3]	MRFASIWAQDHRGVIGSGTAMLWRVPADSRFFRQMTIGSPVIMGRASWEVLGEPLPQRVNIIVTSQPDYRAEGGVVVHSLDEAFERGRAEAARLGSDVVWVAGGARIYRETMDRVDELVVSQLDLTVPGELADLAHVPPVDERTWQVDPTRSDPDWREQSGDARWKVITYVRRPSEPND
gnl|extdb|pgaptmp_000686	K15987	hppA; K(+)-stimulated pyrophosphate-energized sodium pump [EC:7.2.3.1]	MHFAASDDTHRPSRSRLAKGALLGTFVLLFLGACSGSGAPPGSGEVHGGEASLELPDLNAALTWAGWGGRTVLWVALAVCLIGFAFGIVNYMRLQRLPVHRSMLEISELIYTTCKAYLLKQGRFLLILWVFITAVIVVYYKVLVNFPWGRVLTIVLFSLLGMAGSYSVAWFGIRVNTFANSRTAFASLRGKPLPLHQIPLRAGMSIGMVLISLELFMMLLILLILPPEIGGACFIGFAIGESLGASALRIAGGIFTKIADIGSDLMKIVFKIDEDDPRNPGVIADCVGDNAGDSVGPSADGFETYGVTGVALVTFIILAVPDPAMQALLLVWIFVIRAAMLIASGIAYWANAAWVSHRWRHASQMNFETPLSSLVWVTSVICIGFTFGMTGLILGGAHSGLGWKLATIISCGTLAGALVPELVKSFTSTKSRHVRETVKSSREGGASLNILSGVVAGNFSAYWLGLAIVGLMSVAFIISETGLGDFMQAPAVFAFGLVAFGFLGMGPVTIAVDSYGPVTDNAQSVYELSQIEQVPDVNAEISRDFGFTPEWDRAKLMLEENDGAGNTFKATAKPVLIGTAVVGATTMIFSIIIGLTSGLSENLDKLSIMHAPFLLGFILGGAVIFWFSGASIQAVTTGAYRAVEFIKAKIKLDHTSKKASTADSRRVVEICTEYAQQGMLNVFLAVFFLTLTFAFIEPFLLIGYLMSLAIFGLYQAIYMANAGGAWDNAKKVVETDLHAKGTALHDATIVGDTVGDPFKDTSSVALNPVIKFTTLFGLLAVELAVSLTGQGQQVLVHVLAAAFFLVANFFVYRSFYGMRIRTTSEEENP
gnl|extdb|pgaptmp_000685			MKIAVKRAATEIDEAGNVAGHEAGDTLTRRFLRIFPGAQIIGPERRAGNGFEVVPLSQIDPRDTVIVNFDVYDSPTIWNDLYLSTGHQPKVMNFLWWPLSQLETPAQQATTALSCGLFPTFANSRRTAGEITELVRRLTVQPIYEQMTLGWVNLGFRLDHVQQRRDTEVPIVEYPAIYLNSLKRPELFMEILERVHQRTPVQVEMRLEESNLVSEKAMRFSRLPWVWVGPLTSSRTSYWEALSRTTALLATASEESYGLAYVEALGAGVVGVFPDLDWARAILPPEYPFFYQSKVEAEEMLYRAVTDPDGCRAEMDRAVGGSFVEWISQHHSDATFDREVQDFVAQTFAD
gnl|extdb|pgaptmp_000315	K02483	two-component system, OmpR family, response regulator	MANRDALPAPEARLLVVDDEPNIRDLLASSLRFAGFEVREAADGAGAYHEAQEFHPDLIVLDVMLPDMDGFTVTRRIRDAGMRIPVLFLTARDDMRDKVQGLTVGGDDYVTKPFGLEEVVARIRAILRRTKTGPDDDSILRVADVEMNEDAHEVRRAGIPVDLSPTEFKLLRYLLLNAGRVVSKMQILDHVWEYDWDGDAAIVESYISYLRRKLAVSEDLPELIQTRRGVGYLIRESE

gnl|extdb|pgaptmp_000708	K02484	two-component system, OmpR family, sensor kinase [EC:2.7.13.3]	MRLTLRSWPLRRKLLVGIVAVVLSALLIGSIATLLSLRASLYQRLDRDVLIGLELAAGPPGSSPGGRDPGPTDSTGPRQRIDTLEVTFSADGTPLRSAYVTPAGETISLTEPQSDLIWSAIQVGSGPWTVEVGEPLGSFRLAAQPTQAGVVVSGLSTRDVSATIASLERILLGVMGATLLVVIVGTAWLVTRTLRPLHRVADTAERVSQRPLAAGAVTLPERAALSADPRTEVGRVGAALDTLLGHVEQALGARQESEDRLRAFIADASHELRTPLASIRGYAQLAQSEDTEKTPTQERSLSRIESEAGRMAALVEDLLLLARLDSGQTQPHGPVDLALLAIEATSDAHAAHPDHHWSVEVSESVVVSGVDHQLRQVFVNLLGNAGAHTPPGTRVTVTVATKPDRALITVSDNGPGIEPDLLPKVFDRFTRADLARNRAAGSTGLGLSIAAAIVQAHGGTIEVTSSHAGTTFQVDLPLHPAD
gnl|extdb|pgaptmp_000545	K03523	bioY; biotin transport system substrate-specific component	MNSVVRARVQVNSLVRSAAAVVGGVAALTLLSQVTIPLPFTPVPLSLGTLGAMLLGVFLGSKRGVAAAALYAVLGMVGAPVFAGWSSGVMIVSFGYVLGYVAAAGLMGLWMERARHGGYFSALGATLGSSVIIYLFGLGWMMGMFAMPLEQALALGVVPFLVGDALKSLVIAGTARALKGRRIPW
gnl|extdb|pgaptmp_001466	K03524	birA; BirA family transcriptional regulator, biotin operon repressor / biotin---[acetyl-CoA-carboxylase] ligase [EC:6.3.4.15]	MTGSLADPNGFAPHPHPGVILASATSTNQLLAQQWRNFDPFTFLAAREQSAGHGRLGRPWLAPASSALLVSILAPVPAGPADPALTALAAALATADLVAEELPSASITLKWPNDVLLAGQKVAGILTEYLTESEGQHQVVVGVGLNLTVPEAILSQFGATSLAAHGWSHATGTAVPDSVLADLAERLAQLLARRVSRLDQVPAEFSERCPMLGQPATALLPGGGEVSGLARAITATGELVIGQTPVRAGEVTLAKKKEQI
gnl|extdb|pgaptmp_001467	K11263	bccA, pccA, accA3; acetyl-CoA/propionyl-CoA carboxylase, biotin carboxylase, biotin carboxyl carrier protein [EC:6.4.1.2 6.4.1.3 6.3.4.14]	MSTVNTVLVANRGEIALRIVRGAHDHGLRSVAVYAESDRGSHYVREADEAYSLEGTTAGETYLDGAKILAIAERAGADAIHPGYGFLAENPDFAAAVAEAGLTWIGPAAESILALGDKVQARGVAQRVGVGPVPGTDHPLTGRAEVEEFIAQHGFPVILKRADGGGGRGISVISGPTDLEHFFTGRDEDSLSAYFVERYIPRARHIETQCGRDQHGNFTVYSTRDCSVQRRHQKLIEEAPAPFLSEDALAQLHDWSRRLFEGVDYVGLGTCEFLMDEGGQIYFLEVNPRLQVEHTVTEEVTGLDLVGQQLKIAAGEELDRPEGVRGHSFELRITSEDPRLGLTPSLGTLTEIKWPTGPGIRIDTGVEEGAEVTPEFDSMIAKLIVTGQDREHALRRARRALAELAIDGVGTPTSLYQLILDHPAFTGDSLDIWTRWLEEDILPDFLAQFADAELGDEGAAPQPTPLARFVIEIDGRRHELGVPQNLLTASNTTAAPPRPPQPLRSQRESRGQAQAGAADPNLVPSPLQAIVVRVGVEPGQQVAEGDLLLVLESMKMEKYVHAHRDGEVEEILVVAGDNVSPNQPLVKLCPQSEETL
gnl|extdb|pgaptmp_001468			MTLTLPPRTPSAADPDSSREDYVNHLDELAQQAEARAAARQHPAGKLTARERLSQLLDPMSFQEIGQFAGGNVGKDFLGAAVITGHGTINGRRVAVYAQDFSVRGGTLGRAEGDKILHLLDVALEMKIPIIALLDSGGARIQDGVVALTQYGRIFRKTCQASGVVPQLSLILGPCAGGAVYSPALTDFIIMTRDNSHMFVTGPDVVKAVTGEVVSFEDLGGAELHNFQSGVAHYLADSEADALEYARALLSYLPSNCEERPPSYAYEPVAQELVNSAALDSLVPASSRQPYDMVEVIRHLVDHGEFVEVQEMFARNIVVGFACLEGTPVGIVANQPMFDAGTLDVDASEKASRFVRFCDAFGIPIITLVDVPGYRPGTEQEQAGIIRRGAKMIFAYANAQVPMVTVVLRKAYGGAYIVMGSKSLGADMNFAWPGAEVAVMGADGAVSIMHRRELKAAEEAGEDVEALRAQLVQRYTEESVNPNLCVSEGEFDAIITPSQTRQALIDS

gnl|extdb|pgaptm p_001469	K11533	fas; fatty acid synthase, bacteria type [EC:2.3.1.-]	AGFDARTQRPVSAQGHSQGVLAAALYEAWQAGDEQLIAQVFALARLLGAATTQAVRTNGSAPADGATPMLSVRGISRRDLEVLLADAPASVTLSLINGPQ<br>RFILSGRPADLQRLVAAADEWAARDAQAVKSKLRGGVPFSPKFDYLDVAAPFHSPLLESALEQVLTWARACNLPEVLTETLAGHVLVHPVDWPAQLAEARG<br>DGAHWFLDAGPGDTLARITEANLEGTGLGVVALGTPEGRDRFLTHGFQHPTPVNWRQFAPRLVQLPGGKTVVDTKFSRLTGRSPILLAGMTPTTVDPEIVA<br>AAANAGHWAELAGGGQVTEEIFTKNLKSLREQLHPGQTAQFNAMFLDPYLWNLQFGAQSIVRKARAAGAPLEGVVISAGIPELEEAVKLVRELNADGFPY<br>VAFKPGTVNQIRQALAIAREIPDLPLIIQVEDGHAGGHHSWENLDDLLLTTYAEIRECENVVLCVGGGIGTPERAADYLSGEWALVHDQNLMPVDGVLVGT<br>AAMTVKEARTTDEVKQLLLETDGIPGVPADDQTGGWIAVQQSRGNMTSGLSHLRADIHEIDNAAAACARLIAEVSGHADLIEARRDEIIAALNATSKPFFGD<br>VDQMTYAQWARRFAELSYPWVDITWTDRFLDLLRRIEGRLSEAEHGQIPTLFPDVESVEDPWVALDRLEAAYPQAKEVQVGPTDAVWFVALCQRPKPMP<br>FVPVLDDDILRWWGQDNLWQSQDPRYTADQVRIIPGPVSVAGIDRVNEPVGELLGRFEQACVDRATRAGETPTERVCRLEDAVDPAEQIIAARHIMWNG<br>NLIDNPAHLLPREVLTITRQDGPRKVYDVQVSFDTYWDQTPGGEANHAVRQLTIPLDLSAESASGAVPVVDRDTLPPNMYALLAATAGVGGSTITGDPITAL<br>PVMGPSTDSVFGEATWTFTIDENLSALHRSVTAEALPADYQVATWVPDALLGLCWPAIYAALGSALVNDYPVIEGLLSAVHLDHTARLDVDVEELLAHGPTE<br>LTVVARTVELLESSSGRVVHVVETLKHGDREIGEFSERFAIRGRIGSDAVPPTVALAAGQAGPMLETPRSPLRRARVIAPADMTPFANVSGDFNPIHTSHHAA<br>VVAGLEAPLVHGMWLSAVAQNLVSAEGPDGEGWPITGWTYNMYGLVNLLDEVDITVERIGRLPGGGLVLEVTCKIDGQVVSRATATTAAPRTAYVYPGQ<br>GIQRPGMGLDEIDASPAAREIWERADRTTRDKLGFSIVAVVRDNPTEMKAMTASGEQVWRHPDGILNLTQFTQVALATVAFAQTARLRESGALVEGAYFA<br>GHSLGEYNALSAYGEVFPLETVLEIVFHRGSTMHHLVERDEHGRSNYRMGALRPNQFGVDNDSVVAYVDSVAEESGEFLQIVNFNLADQQYAVAGTIAGLA<br>ALEADANRRADAFGGKRAFMYVPGIDVPFHSRVLRSGVPEFREKLMVLLPKEINTERLVGKYIPNLVAQPFELTREFAEAILAEVPSEPIRALLETPGAWEDAL<br>SRPDELSHLLLVELLCWQFASPVRWIETQALLFSPRADGGLGVQRCVEIGLASAPTLAGLAAKTLQLPSFAGINSSALNVQRDEAVVTCTDVRALFVAEDEEE<br>ADSAPAPAAATPAAAPAASGPANPAPAASAPAPAVSAPAAGSGERPADLKYAASDAIRTLMAYTNKLRADQIESADTTDTLTNGASARRNQLLMDLSAELG<br>LASIDGAADATVTELYASVDRLAHNYRPFGPVLTEAIRDRLRRVFGGAGVKASRVNERVTGAWQLGPGWADAVTAQILLGTREGDSVRGGSLATLPVAASS<br>TAEADELIDQAVQEVARERGITVALPQAGGGGGEVVDSAALNELREQLLGGNGLLAQSAHQLLEALGLEGPALLPELEDDADEKLAQRVLEAVSAELGTGW<br>VDQVRPVFDANRAVLIDDRWASAREDLARLGTSGQLADTASFVGAGSAVAEQARWWAGQVSDPALSERFWQIATQAESTEELPYAGEVALVTGMTPASI<br>TGQVVAGLLAGGATVIATASRVDAQRLQFAKQLYREHAAPGAQLWLVPANLSSYRDVDALIDWIGSEQTKVVGPTTTVLKPAQVPSLFFPFAAPRVLGSLA<br>DSGESFEAQSRLLLWSVERSIAALARLAGNASGATRVHVVLPGSPNRGVFGGDGAYGETKAAFDAITNRWKVEKIWGDHVTLAHPRIGWVRGTGLMGGN<br>DPLVEAAEAAGIHTYSTEEIADRLLELCGPEARARAAEAPLNVDLTGGLGDDIDLVALKASALATAPEETVADEGQDVLIPALPTPASPALPGFEPELWADGDT<br>KPEDLVVVVGIGEVSTWGSGRTRHQAELGIDPTGEVDLTPAGVLELAWMMGLLKWQDSPKAGWYNTDGQLVPENEIFDLYRDEVVARSGVRSFIDDAAIE<br>QLGTLEEASVYLNQDLTFTVPDRETAESYLVEDPRLTKIAPAADGEWEVTRLAGAQARLPRRATLTRTVGGQLPTGFDPARWGIPASMIESMDRMTAWNL<br>VTAVDAFLSSGFTPAELLQAVHPAEVASTQGTGFGGMTSMRKLFVDRFLGEDYPSDVLQETLPNVVAAHVMQSYVGGYGAMIHPVGACATAAVSIEEGV<br>DKIATGKASFVVAGAIDDMAVESIVGFGSMNATANSEKMAAKGINERFYSRANDRRRDGFIEAQGGGTVLLARGDVAEAMGLPVLGVVGYAQSFADGIHT<br>SIPAPGIGALAAGRGGLESRLARGLARLGVQPNDIQVVSKHDTSTNANDPNESDLHTRLARALERTPGNPLFVISQKTLTGHAKGGAAVFQVAGLCQLFADG
gnl|extdb|pgaptm p_001470	K00997	acpS; holo-[acyl-carrier protein] synthase [EC:2.7.8.7]	MTVIGIGTDIVDIPTFVEQLGLPGSRLAQAFTARERRAATRAEENGSGTGQHLAAVWALKEAFIKAWSGALFGSQPPLGRDAVKWTEIEIRHDGWGRPQV<br>ELHGEVAGAVSASVGSPTVLASASHDGDVACALVVLDK
gnl|extdb|pgaptm p_000976	K18887	efrA, efrE; ATP-binding cassette, subfamily B, multidrug efflux pump	MLLKLLIRYLKPYKWSLLGILLLQIASVWATLYLPNLNAQIIDQGVAQGDIPYIWQRGAIMLGISFVQIVASVGATYLAARAAMALGRDLRDAVYDRVVGFSEK<br>DVRGFGAGSLITRNTNDVQQVQMMAMMSATMLVMAPLMAIGGVFMALRQDVGLSWIIAVSVPILLALAGLIIMRLVPLFRAYQEKLDTVNLVMREQLT<br>GVRVIRAFVREQIEEARFRVANTDIMVVGRKVGSLFVTLFPMVNLVLNVTMIAVLWFGGHRMDAGEIEVGTIMAFMQYVAQILFGVLMATFMAVMIPRA<br>EVSAERINEVLVSPESVLEAESPVTTVSEPGRIEFDNVSFAFPDAEEPVIKNVTFTIEPGETVAFIGSTGSGKSTLLNLIPRLFDATEGTVRVGGTDVRKLALNTLW<br>NQLGLVPQKAFLFAGTVASNLEFGLEGATEEQMWTALEVAQGKDFVSDMNGGLQARIAQGGTNVSGGQRQRLAIARALIRRPEILLFDDSFSALDLATDAR<br>LREQLRLNFPDTTQVVVAQRISSIMDADKIVVLDQGRVVGLGRHEELAQTCPTYQEIIESQLSAEEIR

gnl|extdb|pgaptmp_000274	K07085	putative transport protein	METILAQQPLLTLFIVVALGAAIGAIRLGPVRLGAAGALFVGLALSAASPSLANDWTIVQQLGLLLFVYTVGIAAGATIRSGLKENLSLLLLAGLASLVGAVVIAA<br>GAGLLHLPAPLSAGLFTGALTAAPALDAATRLTGSADPAVGYSFAYPLGALVGVILVSIVAGRPWRGRRDTPSLAGRGLHAVTVRVAESVNPREIAAWADQR<br>IRLSYVEREGTTRVLIPGEDLRAGDLVVMVGEPGSVEETAREIGDIAADHLADHRDEVEFERMVLSNPDLAGREVGELNLPARFGAVVTRVRRGDLDLLARD<br>DLALQLGDQVAVVVARDHLQAVRDHFGDSQRQVSEVDALALGVGLVLGLLLGMVSLPLPGGESFALGAAAGPLVMGMVLGSFRRTGPLVWTLPDSANLT<br>IRQLGLLFFLAALGLGAGPQVAQLLRSPSAWPALIISVAAVLVACLVVLVGGRLAGMSAPRTAGGIAGFLGQPAVLQAANSRVRDERVESAYSTLFAASIVVKI
gnl|extdb|pgaptmp_000907			MSFRRFFSGLFFRFTKWNLVVEQGIPQNGAILVGAPHTSNWDFFLMLAIAGQTGMRYKWLGKNSLFQGPLAPILRALGGISVDRSASTGMVGQLVEQMR<br>NSSGEVLAITPKGTRSKREYWHSGFYRIAEESGLPLILCFVDSKTNTTGLGPIMAVTGDVHADMEKIRAFYAGKEGIRPELTSVPRLRSEEA
gnl|extdb|pgaptmp_001456			MTNAHSSKRALVTGASTGIGAATVRRLRATGWQVIGVARREERLARLAEETGCEYFAADLTEADSVQRLRDWALARGPVDTVVNVAGGALGVDSIAEADI<br>DRWRTMYERNVITALLVSKAFLPEMRQRGGDLVFLTSTAAHDTYPGGGGYVAAKHGERIIANTLRQELVGEPVRIIEIGPGMVKTEEFSLHRLGDQAAADRV<br>YQGVAEPLTAEDIAETIAWTVELPAHVNIDSMIVRPVAQATNTVVARNSQ
gnl|extdb|pgaptmp_000719	K01885	EARS, gltX; glutamyl-tRNA synthetase [EC:6.1.1.17]	MDTNNSAIRVRFCPSPTGTPHVGMVRTCLFNWAYARHTGGTFVFRIEDTDAGRDSQESYDQIIESLRWLGLDWDEGIEVGGPHGPYRQSERTEIYREVIDQ<br>LIQGGYAYESFSTPEEVEARHVAAGRSPKLGYDGFDRDLTDEQRAAYRAEGREPVIRLRMPDEDITFTDLVRGDVTFKAGSVPDYVIVRANGDPLYTLVNPVD<br>DALMKITHVLRGEDLLSSTPRQVVLYRALIELGVADEMPKFGHLPYVMGEGNKKLSKRDPESNLLLHRQNGMIPEGLLNYLALLGWSIAPDRDIFSMDEMV<br>AAFDIADVNPNPARFDQKKCLAINGDHIRLLEADDFRNRLVPFLAEADLVSATDFEALTEREQQVLTAAAPLIQTRIQVLSEAVGMLGFLFSADESVHVEDDA<br>RKTLKDGADRVLETAIEVIEGLAEDQFGVEQLESALREAIVEQMGVKPRLAFGPLRVAVSGRRVSPPLFESMEILGRTTTLGRLRSLRDSLGA
gnl|extdb|pgaptmp_001536	K03800	lplA, lplJ, lipL1; lipoate---protein ligase [EC:6.3.1.20]	MHGEYKMPGGKLVVADVEVNEGVISSLTLSGDFFIEPDEALFRMQAAVLGAPADLSGAELTTRMEVALEPTDVLMGVSPGAIGVAIRRALGAALSWDDIDF<br>EVIHGPSVAPVINVALDETLVEDVAAGRRRPFMRIWEWNAPQIVIGSFQSYDNEINQEGIDRHGITVSRRISGGGAMFMEPGNCITYSLVVPTALVDGLSFE<br>AAYPFLDEWVIEALKKVGVNATYVPLNDIASEKGKIGGAAQKRFANGYMVHHVTMAYDIDAVKMNECLRIGKEKIRDKGLRSAVKRVDPMRSQTGMARE<br>DIIEVLKDHFAQKYLADPGEITAADLEVAERRAEDKFSSPEWVYRIP
gnl|extdb|pgaptmp_001540	K00052	leuB, IMDH; 3-isopropylmalate dehydrogenase [EC:1.1.1.85]	MSVLQVAVVPGDGIGIEVTAQAVRVLEQVLDQAGAGLNLDYFDLGANRYLETGDILTEADQEALARHQAILLGAVGDPRVAPGILERGLLLKLRFGFDQYVN<br>LRPSFYYPGVDSPLQHPDGTDLVVVREGTEGLYVGNGGSVRTGTPQEIATESSINTAYAVERVVRYAFELAASRPAQHLTLVHKTNVLVHAGGLWQRQVEA<br>VAQHFPQVQVDYAHIDAATIYLVKDPQRFDVIVTDNLFGDILTDEAAAITGGIGLAASANLNPEGRFPSMFEPVHGSAPDIAGQGIADPIAAIGSVELLLANN<br>GFPAEAERVRQAIMADMTWRAENPGQRRSTAEVGDAVLAALN
gnl|extdb|pgaptmp_001549	K02434	gatB, PET112; aspartyl-tRNA(Asn)/glutamyl-tRNA(Gln) amidotransferase subunit B [EC:6.3.5.6 6.3.5.7]	MTEVMAYEDAVRRFDPVMGIEVHVELGTETKMFDCAPNAFGGDPNTFITPVSIGLPGALPVTNAKAVEYAIRIGLALNCEIAEYCRFARKNYFYPDLPKAYQI<br>SQSDEPIAANGWVDVELSDGEIFRVQIERAHMEEDAGKNTHIGGADGRIHGAEYSLVDFNRSSVPLVEIVSRPIEGAGERAAEVAAAYVQTLRDIFRAIDVSE<br>ARMERGNVRADINVSLRPTADSPLGTRTETKNVNSFRSIERAVRFEVGRQAAILADGGSILQETRHFHEEDGTTSPGREKSDADDYRYFPEPDLVPLQPARE<br>WVEEIRASLPELPLAKRQRLNAEWGFSALEMRDVISAGALDLIEATTLAGASPAAARKWWMGELSRLANDQEQALADLSVTPAQVAELQGLIDEGKLNDKL<br>ARQVLQGVLAGEGGPTEVAEARGLAIVSDDGALNAAIEQVLAENPDVVEKIRGGKLQAIGALVGGVMKLTRGQADAGRVRELIAQTLGV
gnl|extdb|pgaptmp_000977	K18888	efrB, efrF; ATP-binding cassette, subfamily B, multidrug efflux pump	MIGLMAPYKWAMLLVSLLGAGGVVLSVVAPKVLARATDLLFSGFISKRIPAGANLDQAIEMMRAKGDTDMANMVESMNITPGQGVDFKALAAVLTLVLV<br>LYLVSALLMWLQGYILNIVMVKAMWRLREQIEDKIHKLPLSYFDRNRRGDLISRVTNDIDNITQTLQQSLSSAITSVFMVIGVVAMMLSISWKLTLIVLVSLPL<br>MAIIFGVIGPKSQKAFATQWMKVGALNNRVEESFSGHALVRVYGQTERFESQFAQENEELYRASFKAQFLSGAMMPSTRFVGNIVYVGVAVIGGLMVASG<br>KMSLGNVQAFIQYAQQFNQPVGQLGGMAVAVQSGVASAERVFEILDAPNEEPDSPDAPAPVEGDGKIIFENVSFSYTPERPLIKNLSFEVQPEQTVAIVGPT<br>GAGKTTLVNLLMRFYELDGGRITVNGQDISKLTRKDVRARTGMVLQDPWLFAGTVLDNIRFGNEDATEEQVREAAKATYVDWLIKSLPHGYETVLDEDAA<br>NLSAGERQLLTITRAFVSQPSILILDEATSAVDTRTERLLQKAMGALQKGRTSFVIAHRLSTIRDADLILVMEDGDIVEQGNHEELIKRHGAYYRLYNAQFEGSF

gnl|extdb|pgaptmp_000714	K03564	BCP, PRXQ, DOT5; thioredoxin-dependent peroxiredoxin [EC:1.11.1.24]	MSKLTVGEPAPDFTLDTLSGPKSLQELREGADRGVIVYFYPKAGTPGCTTEACDFRDNLNSLKGAGYTVIGISPDPISALEKFTDKQNLNFLLASDPDHEVMTEWGVWGEKKNYGRTFLGVNRSTFVVDPDGTLTLAQYGVKATGHVARLRRELGID
gnl|extdb|pgaptmp_000968			MAASASPFRALGHYRLLPGLVGWPALIVAFLARTPFAMIPLGVMTAFTAATGSVAIGGLATGIASISTAIAAPLLGRVAERVGQKKLLLSVVPVNALGLLGLFILSFRSEVHWSLWALCILTGATAVPVGSFTRSRWVQKTTTPYQLSAAFSYESMADELVFVLGPALVGVAASTAMPSAPLALAFIVMLAAGLPFALTAPGKTALPTEAKEVSTEPAPPIFRVIWTVILPIAVLVCVGAFFGSSQAAITERADQMGMSGQAGLVYAVMGIGSAISALMVVVIPERFAFWKRIFISALGMAVGMVLIALAPNLGLTALFMGLTGFFIGPTLVTAFSLTERMAPPSGMTVAMTTMSSSVTVGVAFGSSVGGALAARAGAETAFLFGAGASALIVVLALLLATPKQLQRVSRN
gnl|extdb|pgaptmp_000967			MAKNSSSGADTSSAPKKNRWYKNLADSYRIVARTYKWVPYAMIILPILLLGGSVALGMVLKPVWMWLITGVMLALLADMLILSTLLRPAMYSQVDGTVGSVYVVISQIKRGWVINDQPVQVTREQDLVWRIVGRAGVVLISEGPSNHVRPLLVNERKKINRITANAPVVFIECGHDEGQIPLKKLPRKLRSLKKQLTKAEVPAVAMRLDAIEAKSSPIPRGVDPNNVRMNRRALRGK
gnl|extdb|pgaptmp_000298	K02030	ABC.PA.S; polar amino acid transport system substrate-binding protein	MRTRTGIAAIAAVAALALAGCSDPGTTTDTQSEPQSDSGQSQAAAGMEITPFDISTISVQEDIAALVPQAIKDRGILRNGASTDYAPGEYRADDGQTPVGYDVDLVRALARVMGLEDGQTSHAEFPTIIPALGSKFDVGVSSFSVSPERIEQVNMISYVEVGSAYAVAAGNPKDFDPNNICGTTIGVQTGTYQHDYLLAQSDQCVADGKKAIEVMPLTVQTDVSTKVIGGQYDATLADSTVIGYTVKKSDGKLEQIGDVIESAPQGITVKKDDEALTEAIQKALQYLMDNGYLTQILAPYGADGAALTTAELNPAS
gnl|extdb|pgaptmp_000299	K02029	ABC.PA.P; polar amino acid transport system permease protein	MPSTIDGVELLHARPVPRPGRWVSAFLVLVIVALVIKGLITNPNFQWSLMGQWVFSKTIMTGVLYTLLLTVVAMVIGTVLAITMAVMRQSPNPVLRYVAMFYIWFFRGTPIYTQLIFWSLLPSLYPQISIAIPFGPELFSAQTVDLLRPLMMAFLGLGLNEGAYLAEIIRAGLLSVDKGQWEAATALGMNRGKIFRRIILPQAMRVIVPPIGNETISMLKTTSLVSAIPVTTELTFVASTKGQALFAPIPLLLAAAAWYLVITSLLMVVQHRIERYYGRGFDSRTKADKASSRQAAINSAPTTRDDPFLDVTP
gnl|extdb|pgaptmp_000300	K02028	ABC.PA.A; polar amino acid transport system ATP-binding protein [EC:7.4.2.1]	MTQTPMVQVKDVHKLFGDLHVLKGVDLEVAPGEVCVVLGPSGSGKSTLLRCINELEKISAGRIYVDGELLGYREVTKGGKTTLHELPEKAISRQRSRIGMVFQRFNLFPHMTALENVMEAPVQVAKRPREEARARALELLDRVGLSDRPDHYPAQLSGGQQQRVAIARALAMDPELMLFDEPTSALDPELVGEVLAVMKELAASGMTMVVVTHEVGFAREVADEVVFMDEGVIVEQGAPAQVIDHPQHERTREFFSKVL
gnl|extdb|pgaptmp_000961	K02013	ABC.FEV.A; iron complex transport system ATP-binding protein [EC:7.2.2.-]	MSSTAALANPLVRVEAARVDRAGQSILQDINWQILPGEHWVIVGPNGAGKTTLARLLTGRDYLSAGTIEVLGEDLDQHSAQDLASRVGFASVEVGQRLGVQDSVLEVVLSSAWGQSTSFGEEYEAPDRERAADLLAALGVGALADRSFASLSEGERRRVLLARALMADPEILILDEPTAGLDLGGREILLTALTEIMGAPTAPTTIMITHELEEIGPKFTHALLLRAGQVVGAGPIEEMLTGPNLSTAFDLSLQVSQDQGRWRAIAAGQ
gnl|extdb|pgaptmp_000960	K16148	glgM; alpha-maltose-1-phosphate synthase [EC:2.4.1.342]	MRVDILTREYPPFIYGGAGVHVNELAKVLRGRVDVRVQAFDGPRTPGEDGGDEGVVGHADLPELTGGNAAISTLGVNLEMVNACAGTDLVHAHTWYTTFAGYLAQQLYGVPLVISAHSLEPLRPWKAEQLGGGYQVSSFAERIGYENADAIVAVSHGMRDDILRCYPSVDPDRVSVIHNGIDLARWRRPEGAEAQARAEQVVRDHGIDPNRPTVVFVGRITRQKGLPYFLRAVELLPKDAQVVLCAGAPDTPEIKQEVEDLIAELQQKRTGVIHIGQMLPHSELVAILAAADVFVTPSVYEPLGIVNLEAMALGLPVVGTATGGIPDVIVDGVTGYLVPIDQAQDGTGRPLDPTQFEGDLAERLTRVLENPERARQMGQAGRLRVEEHFSWDQIGRQTVELYRRL
gnl|extdb|pgaptmp_000959	K00975	glgC; glucose-1-phosphate adenylyltransferase [EC:2.7.7.27]	MAKNNVLAIVLAGGEGKRLMPLTVDRAKPAVPFGGHFRLIDFALSNMVNSGYLRIVVLTQYKSHSLDTHISKTWSLSSMLGNYVTTMPAQQRRGRHWYLGSADAVYQSLNTVYDESPDYVVITGADNIYRMDFSQMVDHHIASGLPATVAGIRQPIELSNQFGVIDAKDGVVHQFLEKPADAEGLPDDPTKVLASMGNYVFDTDALIAALKADADDPDSKHDMGGNIMPYFVERGECGVYDFLENHVPGATDRDRDYWRDVGTLDAYYEANMDLISVHPIFNLYNFEWPTLTHMNAALPPAKFVYAGEDGRLGQAIDSLVSAGVIVSGGQVFHSVVSSQVYIHSWARVVDSVLLDGVEVGRSAVVKRAILDKNVIVKEGASVGVDHERDRARGLTVTESGIT

gnl|extdb|pgaptmp_000958	K01079	serB, PSPH; phosphoserine phosphatase [EC:3.1.3.3]	MTGSSHQLTVYEGSTPVGPSAPELKGKHWWGTIDRPSQPGAIGHSQIGPQWLQDRPFLLISDVDSTLLAEEVIDELAALAGVGAKVAELTERAMAGNMDFSDSLVARAQQLRGLPATAFDEVRARLHVRPGAQTLVDWVHSLGGQVGLVSGGFTPVVAQLSSELRIDHHLAIDLEVRDGALTGAVEGPIVTARTKLEFLTRLREQTGLPVVALGDGANDLLMLGAADVGIGIGAKPIVRQSVANYLEAGRLDPVIGLLGHSEI
gnl|extdb|pgaptmp_000957	K08296	sixA; phosphohistidine phosphatase [EC:3.1.3.-]	MNTAILIRHAHAQDLAAGGDRFRPLSNRGRRQAENLGRDLAPRLAQPALALVSPAVRAQETFELLSEAAGVSIPSRTVEAIYGYEVDGILEVIRLCGEGSLVLVVGHEPLISDAAWQLAGPDGGAPVSVPTATALVFESELPWDQWQIGAARFRETYQG
gnl|extdb|pgaptmp_000956			MAITKGPEALICSARGCEELATGAIIWSNPKLHFGRHKTWLTCPEHREFLTNYLGYRRFPVEYLPLDEFLAREEAAEADQS
gnl|extdb|pgaptmp_000955			MISVQDFSLRIGARELLGPTSFHVGKGAKVGLVGRNGAGKTTMMRLLAGENDRGVAEHGGTITRSDTVGYLPQDTTEGDPNQSARARIISVRGIDTLLERIKKAEQEMSQQTGARQQKAMERYVRLDHEFHQSGGWAANSEAAQIAAALGVDERLLEQPLGTLSGGQRRRVELARVLFSGAETLLLDEPTNHLDHDSIIWLRDWLRTYSGGFLVISHDVNLLRDTVNQVFYLDARRARLDHYHLGWDDYLRQREEDERRRRKERANALRKAETLQAQGEKMRAKATKAVAAQQMLRRAEQLREAAGEEMGQEKVARIKFPDPAPCGKVPLTGHELSKSYGSLEVFSGVDLAIDRGSRVVILGLNGAGKTTLLRILAGVQDPDTGQVEPGHGLKLGYYAQEHDTLDLVATVEENMRRAAPGLDDTKLRSVLGQFLFGAEDISKPAGVLSGGEKTRLALAILVVSSANVLLLDEPTNNLDPASREEILAALRTYQGAVVLVTHDPGAVEALGPDRVLLLPDGDEDLWDPSYLDLVTLT
gnl|extdb|pgaptmp_000954	K09125	yhhQ; queuosine precursor transporter	MTELSPRATRVYDVLAISFVGFLLIANVAATKLFMVQLGSWHLIFDGGALLFPLTYVLGDVLAEVYGFARARRVIVIGFALSALAALTFWVVGALPPAPDYEHQEAFTAVLGFVPRIVSASLLAFLAGQLLNAYVLVWIKRQWGPGRLWVRLLGSSVVGEAADTLIFCTIAFAGILTGAEFWNYVLVGYLYKLAVEVIILPLSYPTIRW
gnl|extdb|pgaptmp_000953	K00773	tgt; queuine tRNA-ribosyltransferase [EC:2.4.2.29]	MVSNWIGAPAQMWAPTEAGPDRGFTLEAELPGTLGRTGVIHTAHGPIRTPAFIPVGTKANVKGLVPEMVRDLGAQAVLANAYHLYLQPGSDLVDEAGGLGRFMNWPGPTYTDSGGFQVLSMGAGYKKVLSDQFAGKIAGDTAAQERLAVKESRASVDDDGVTFRSHLDGTKHRFTAEVSLGIQHQLGADIMFAFDELTSLLHPYDYQVESLARTEAWARRCLQAHRRLTEERVSKPYQQLWGVVQGAQYQDLRERAATTLARMDEDGWQFDGFGIGGALEKERLGTIVGWVSAQLPADKPRHLLGISEPLDFFQAIEAGADTFDCVNPSRVARNAAIYTPDGRFNITNARFARDFSPLVTGCGCYTCTHYSQAYVRHLFKAKEILAATLATIHNEWFTVRLVDAIRQSIADGTYWELKEDVLERFYGN
gnl|extdb|pgaptmp_000951	K04488	iscU, nifU; nitrogen fixation protein NifU and related proteins	MSSLEQLYQQVILDHSRRRSGFGPIAGLEYTSHQVNPTCGDEVTLGVKVGPDGQLDEVHWEGDGCSISQASLSMMTEMVEGADLDRLRQLYHAMDTMMHSRGQGVDEELLDELEDAAALEGTSKFANRIKCSLLGWSALRDALAQAGYDIAITEEDSTDE
gnl|extdb|pgaptmp_000950	K11717	sufS; cysteine desulfurase / selenocysteine lyase [EC:2.8.1.7 4.4.1.16]	MSTAEVASSLAQPFTAAELEAIRADFPILSRHGRGGRKIAYFDAAATSHKPNRVIDAEAEFYRNHNAAVNRGTHLLGDEATESFESARATVANFVGGRPDEIVWTKNSTEGLNLVAYSFSWLGPQDRIVITRAEHHSNLVPWQQLAARTGAELRWLDLTSDGCLDLDTLDVITPNTKVVAFTHASNVTGAVSPVAEVVAAARQVGALTVLDTCQSSAHQPVNVSQLGVDFAVFSSHKMLGPTGIGALWGRRDLLADLPPFLTGGSVVADVTMETTEFLPAPNRFEAGSQPVAQAAAWAEALRYLQELGMDRVAAQEHALLQPLFDGISEIPGVRILGPATTEGRLGVVAFAVEGVHPHDVGQVLDADDVAVRVGHHCAIPLHRFFGVRSSSRASLSVTNTVSEI
gnl|extdb|pgaptmp_000949	K09013	sufC; Fe-S cluster assembly ATP-binding protein	MSTLNIKDLRVSVETPEGTKQILKGVDLTVGSGEIHAVMGPNGSGKSTLAYALAGHPAYTIDGGEAWLDDQNILEMSVDERAKAGLFLAMQYPVEVAGVSVANFLRTAKTAIEGKAPAVRQWVKDVDRAMTDLKMPGEFANRDVNVGFSGGEKKRLEILQMELLEPSFAILDETDSGLDVDALRIVSEGVNRVHRKNGNGVLLITHYTRILRYIKPDFVHVFVDGKVATQGGAELADQLEEQGYDKYLVA
gnl|extdb|pgaptmp_000948	K09015	sufD; Fe-S cluster assembly protein SufD	MTNTLTTPRPHSHGAAPSPAGHSSRADRPTSFSVAEIPVPHGREEDWRFTPLRRIRPLFELENYTGTNTVSVEGPAPVQVETVDRDDPRLGQVLAPGDRTAVVSWNEFPQSTVVTIPSEAELTEPVFVKITASDAPSAQHLLIKAEKFSTGVVILQHSGPGWLNQTVEVRVEDGARLQLVSIQEWDRTAVHASDHRVSVGRDASLDHLVVTLGGDLVRMCVDTEYTAPGGEMRLNGIYFVDAGQHMEHRPFIDHSQPKCYSRVTYKGALQGKDAHSVWVGDCLIGELADGTDTYELNRNLLLTEGAKADSVPNLEIENGEIEGAGHASATGRFDDDQLFYLMSRGIPEIEARRLVVRGFFAELVNEIPVPEIRDHLMDAIEAELATTETPQS

gnl|extdb|pgaptmp_000947	K09014	sufB; Fe-S cluster assembly protein SufB	MTQSLPATPVDERPQGVNELGTGPMSDDEIIDSIGAYEYGWKDSDDYSKGVPKGINEDIVRYISATKNEPEWMLERRLKAFDFFERKPMPTWGPDLSGIDFDNFKYFVRASDRQVKDWEDLPDEIRNTYDRLGIPEAEKNRLVAGVAAQYESEVVYQQIQEDLERQGVIFLDTDSGLREYPEIFEEYFGKAVPAGDNKFAALNTATWSGGSFVYVPPGVQVEIPLQAYFRINTEAMGQFERTLIVADEGSYVHYVEGCTAPIYDTNSLHSAVVEIFVRKDARVRYTTIQNWSNNVLNLVTQRAMVDEGGTMEWIDGNIGSAITMKYPACYLRGEHARGETLSIGFAGEGQHQDTGAKMVHMAPHTSSSIVAKSVSRGGGRTSYRGLVEVHARATKSKSNVLCDALLVDKISRTDTYPYVDVRTEDVEMGHEATVSKVNEDQLFYLMSRGLDETEAMATIVRGFVEPIAKHLPMEYALELNRLIELQMEGSVG
gnl|extdb|pgaptmp_000946			MAELKDLGTRQVVLDLVVEKGPVTSGTIAKMLSLTTAAVRRHITTLEENGDIVEHEVPVLKPRGRGRPARYYVATDIGRDRLSDGHSDLAIKAIGYLATIAGPEAVDSFAAARSRDIERRYQPILNEVGGDARKRAQALADALTDDGYAASVRPVGNGDFAVQLCQGHCPIEQVAREYPQLCEAETAAFSKLLGVHVQRLATLAQGEHVCTTVVPVGVAPLHPGARRVLKHSNSHN
gnl|extdb|pgaptmp_000944			MVNLRYHIVSLVAVFLALALGVVLGAGPLQRQINAASEGTNLAEKSSQLESQLATVQAEADQYATFVTDTAEQVLPGSLADRKVALILLPGANAEVAESVQATLREAGATVTGAAQLTDNWVSPGQREYRDTLANPVSSHLAASNQSGAADSILAQALVEALTGTGAEVDLLREILTDVDTPLVVANSMPEAPADQLVLITPSTPYPKAGQEEDSQSGSQPAASEQALTALAGALADRTEGAVAYGAAVTDDDVIALLRGQGTSLATVDQIGTPMGNLNVALVLANQSRGAFGQGIGATTAVAP
gnl|extdb|pgaptmp_000943			MSRKVNTSALAGSIRLDESIPRLAGRLEPGEIAIIEFPDLDRSSALALLSRRPVAVLNAASSTTGRRPSLGAQLLVDGGITLVDDLGSDLMTLTEGDQVRIQGGDVYRGEELIASGQRRDSAELHQAQASGRERLGPAVESFARTAGLTWESESEQYLHGEGVPPVPALSGRTVVVVTPGLTSIRQLRRLKAFCNDFSAYFIAVGEGANSLKSVGRKPDLILGDISNLPEPMLTRGTPLVLLERPDGQVTGGDRANVLTLKFMRMVTSAAPADAAVLLADANGADQIVLVGDDEGIEGFLEKTGSEVTAGFFIQLRSEAKLVSAPAVQRLYRPGVRTWQLVLMLIAALLVMVAAVLFTPWGQSLGWGLYDWVQGWWPWAGDPNVTAAYFGS
gnl|extdb|pgaptmp_000942	K03631	recN; DNA repair protein RecN (Recombination protein N)	MIEELRIKGLGVIDEAHLELAPGFTVITGETGAGKTMVLTSLRLLLGEKGDGALVRTGHPQIEIDAIIQPTATVAKHLAELGFESEELILSRTVPANGRSRAAAQGRPVPLRTLEELVSPLLTIHGQADQWRVRRSQVQRALLDTYAGEQHQQLLARYREQWAAVTTLKRTLDELHRDHDQQQIEINYLREVITTLTQLAPQVGEEEELPALIERYSHVADLAQTVGDAVQTLQGEDNLVGVLDLLGQCAAELRSAAGLDSALTSYSDRLAQVEGEVADIAADLWQYVDQLSEDPDELASLQQRRADLEALMKGRATTVAELLDWQVEAEARLAELTGSGADPEQVAQQLAAAQSQLRELGDQLHRSRHQAGLRLAKIVNRELHELAMPQAQFRVQVQAEEPQAHGSDDVQMELRARPDAPFRPLGDGASGGELSRVMLALEVALGEQAEPGTYIFDEVDQGIGGHTATEVGRRLAQLGQTQQVVAVTHLPQVAACADRHYVLRRHGQQTSVEEAVGEDRVEEIVRMLGGKADSDPVRRHAAELLADKPWQDRERKKEG
gnl|extdb|pgaptmp_000941	K00858	ppnK, NADK; NAD+ kinase [EC:2.7.1.23]	MAKRILLECHPNREDVGPAADTVRAVAAALGMQVTVSPDPDQPPELVLALGGDGTFLAGARTARHYDIPLLGLNFGHMGFLADTSDDSLEVVVERIRRDAFEVENRMTLEVEIISPLGALARQWALNEAAILHSDLAHPADLAFAVDGQVVSTYGADGIILATPTGSTAYSFSAGGPVVWPDTEAIVMAPLGAHGLFTRPLVVSPNSVLEVGILPSNRRAPQVWIDGLLALDAPAGSVVMTTRGARPVRLARLENHPFSERLVNKFNLPVSGWRSPRPHGPNR
gnl|extdb|pgaptmp_000940	K06442	tlyA; 23S rRNA (cytidine1920-2'-O)/16S rRNA (cytidine1409-2'-O)-methyltransferase [EC:2.1.1.226 2.1.1.227]	MTLRRLDAELVRRKLAQSRQQAKEMVEAGQVKVNGQLAAKAATQVSTAAPIVLVDPPADQYVSRGAHKLLGALAALGERAPQIAGRRCLDAGASTGGFTDVLLRRGAASVVAVDVGYGQLAWSVRQDPRVENLERTNIRYLEPDQVAPAPDLVVGDLSFISLELVVPALIRCAPKADFLLMVKPQFEVGRGALGKNGVVWDPEQRAQAVAKVAHFCAEQGLTVWAIEPSPLPGPAGNVEYFLAMSSGPGPVALPDLDEAARDAVNRGPHPNSEDSHG
gnl|extdb|pgaptmp_000939			MAEDRNSNRGQRGPGGSGRPGGKDFGSRGGKYDRGGKFDRSGKPERGGKFDRGGKKNFGGKSKFLAKNKPHHREYRSADPNEPVIPAGVTADELDADALKALKTLSGANQEIVARHLVTAGQLLDIDPELAYQHAQAAVRRAGRVDVVREAAALTAYVSGRYDEALREVRAVRRMRGDVSLRAIEADCERGLGRPERAIEIVEETDTSQLSLEDQVELILVAAGARADLGQMDYSLMIVENALRSLPEDTPSELLRWLGELRAERLQDLGRLEEAAQVLDELPPEEDPMEIVDLDLLLEADVDKVRTDLRGGGEPLAKMFDGALLDLDGVCYAGAEVIAGGPGAIDLAEDHGMQIGFLTNNSSRSPQAVADKLSALGYVAEANQVMTSAMDLLMDLKEKFEPGTKILVTGSEELARMTAEAGFEVVGEAAAEPAAVVQGLSQDLGWAQLTEAALAIQAGAIHFATNLDPKLPTERGFAVGNGSLVAAVRNATGVRPIAAGKPRPDIFIRAAQMLRMERPLVIGDQLSTDIAGAVSAKMASLHVLTGVSDARDIVLAPRGQRPSFVALDLNGLNEVHPRPRHHRDGTWTCGVSQVVAIDRRGRIRIGEAWLTGSEELVTLNLDTYRALIAAAWEIEDERTQVACPPLKVVPNDDETGVVEPAPEPEAETAPVEEHGPVEEAGPETAPVADAEPEANAPVSDRSAESD

gnl|extdb|pgaptmp_000952			MSNPMANTEPVEQRFQMPEAVPSQGITVIQNGTVTAEEILEAMKDVIDPELGINIVDLGLVYGVSIAADNAIVLDMTLTSAACPLTDVIERQVQMVLGPYSTDVSINWVWLPPWGPDCITEDGRAQLRAIGFNV
gnl|extdb|pgaptmp_001953			MRRFLAGLLLTVAALMVPVTLVSQWIQNYLLDTDNFTALYQPVAGKSAFQQYLAGQVSEAAGEAIEQSAVAQFTQSAAGAVDDVVGLFGLDLGLSDTSSDWVSRLGDRVAETVHGEALPALQSPQFAQVWTEGINQIHSQLITGLTGDGPDSQTLVLQAGPFVSIILEYLQQQGFSFVSFLAPLAADSEVVLVEVNYPPAARTFVNLIVDYGPYLPWVTAGIAALGLLLSRARLVSLGRFAFATAFLSALVWLVAPLLGAFALADVGGGQEVAKLLWDVAVTPLRSEALLVAGSALLLSIFAGALH
gnl|extdb|pgaptmp_001965	K18479	yihS; sulfoquinovose isomerase [EC:5.3.1.31]	MGWFDSIEHSRWLSEEMRALLRHGHAAVTRTGFGYFTSDGQVDQTRPVDLAITARMTYVYSLGVLMGIPGSRRYCDHGVRCMKEYFRDPEHGGWYKAITHTPDQDGHGVPWDEEGSRKWQYAQAFLILAASTASIANRPGAYELLHLALAEQKEHWLDPDTSLVRDCATSDWGEFKDYRGMNSLMHTVEAYLAASEAVQDVEWLRAAERMLRFVYQVGVENQWRIPEHYTSDWRPMLDYNRDQPDTPYYPYGFVIGHGMELARLAVQTQAGLQAQGLDDYDYLTVMAEELFERARTDGWRRDGQPGFVYTVDFDGTPVLSDHLQWVVSEGICATAFIRRAKLDAGAVAGEVEAYEHSYRVWIDYLNDYMQLRPGVFARALSANNEQTSDTVSSRPDVYHTLQALLAGRLPVWPPFASAINHGLLDQPASTNQAEKRTRKRFLRG
gnl|extdb|pgaptmp_001966			MTSDWLITPPPSGQEGIPFAEAVARAEADLPAVLPDDPSQLLFALDIDGTMVGTHGVTDRMKQALAEAEAAGANIVIATGRGVYSTRHVVEELGLHRSWVVCSNGSLTVRWDGDGHQVADINEFDPRPTGEQFLETFPGILLGVDVGAGGMLVSQLFPAGELMRQELADSLEAVLGRRATRLVARAPWLERDDFAQQIDEMNLVDVEYAVGWTSWVDIGPAGVTKATGLQRLVDQLELPSTGTFAIGDGENDLAMLRWAHHGVAMGSADEAVRSAADAVTTAVEHDGAAAVIRAVL
gnl|extdb|pgaptmp_001967	K01875	SARS, serS; seryl-tRNA synthetase [EC:6.1.1.11]	MIDLRMLRENPDAVRASQVARGEDPALVDQVIAADELRRSLLQEFESLRSEQKAVSRSVGKASPEDRPAVLARAKALADRVKAAEQEANEAEARARDLTLALSNVIEPGVPSGGEDDYVVLRQEGPAPRDFAAEGFTPKDHLELAEGLQAIDVKRGAKVSGSRFYYLTGIGARLELALLTMAVDLGTAAGFTMLTTPTLVKPEIMQGTGFLGAHSDEIYYLPADDLYLVGTSEVALAGYHQGEIIDLSEGPERYLGWSTCYRREAGAAGRDTRGIIRVHQFNKVEMFSYCRPEDAAEEHRRLLGWEEEMLARVELPYRVIDTAAGDLGSSAARKFDCEAWLPTQERWMEVTSTSNCTTFQARRLQIRERREGGTETVATLNGTLATTRWLVALLENHQRPDGSVHV
gnl|extdb|pgaptmp_001968			MNGPMLVLLYALVVSGGVSAALLRTLHRRPAHLPRPGGKTVAPGRVWVIANPTKPDDYARFQQTINTLCQRMTGHPAQWLETTREDPGTGQAAKALATKPAVVIAAGGDGTVRAVAAGLAHSGVPMGIIPVGTGNLVARNLGLPLDLPSALEVAVSGRTAPIDLAWLRLERVSEPGELPAEGGLLRALRPEVDVAENEFAYLVIAGIGFDGETMANTSARLKRAVGWSAYVFTALKSLRIERMKATVTLYHPQGVAGARPKWAKAVPPQVYQAIEDSHTLGQDAANQDRYVTGLRARTVLMANCGTLPFTVLAPYAEIDDGLLDVIAIDTKGGLFGWINLAAKVLAQSVGLRPFNLRRDLGQISFQQCHAVQVDTNRPYPVQVDGDPVGTARTVITRLDEG
gnl|extdb|pgaptmp_001969	K04518	pheA2; prephenate dehydratase [EC:4.2.1.51]	MTQEPTNFPGPPFAGPEVTISFLGPFGTFTEQALWQVAPAGAKLQPKTSVGQALEAVRTGEADRAVVPIENSIEGGVNATIDALSQGGLVIVCEMIVPVSFSFAVRPGTRKEDVKFIGTHPHAWAQCRGWVEENFPGAVHVPTTSTASAAELLGGTADVSIQAALCNAVSVERYGLEAMYQDVADNRGAVTRFVMVSLPGAIPPKTGADKTTVQVKLPDDEAGALLTMLEQFSARGVNLSRIESRPGGDGLGSYAFSIDIAGHVQEERVKAALVGLYRTCPEVKFLGSYPRVDGVRAQVRPGTSDDDFGRAWEWMNSVLFEGKAD
gnl|extdb|pgaptmp_001970			MTSWLLAVALAAAMALVAVGTSYFWLRAARVPAFIAGVFAPAVTVALVVGLGYLYNWLGIFWSGARVIPVLALIGVTGFGVFVWRWRTGWGRRGASHPVQPWGWAFWAAVALGWVLAVVPMVVSGPATNPVQQWDPSFHMNGVWSINHLGDGRMGSGLDESFREGNATAYPLGWHIFTALFTTPSTVVLGANASTLALILLWVVGAGAYTRMLFPQAAWVAPLLAGGMLSMPGDALMAYSQWPNATTVALLPGIASMMILLGRHLLEWWDGTGQVSRSKLVGGVIVLALSIIGAAVVHPIIAFNLLVLLTPAVLAGVFHLTGKYWPARRWGRLGSLWAAVVAGVAVVLVVMQSPSVRSMGEYPRSGVSWQVAFANLLTPTPPYPNSLSLFVWSGILAALLVVGLIRSQPRWPAFSFALFALITFVAYAPNSAFRQWLVAPWFLDPRRTMEPESLAIVPLAALGVVAVAHWLSQAGLRYANALLVVVLLVASGGEGLGARIGAAQSVYDPDRLGKPGMATAGELEMLRSLPDLLPPDARVLGDPQNGSVYAQVIGQREVFFPALTLSSNPSDNETILVQRFNQIQTDPSVCQAVREEGITHFYADNDGHYYSRLRSDRTPGLYNVDTSTGFELVAEGDTARVYRITACD
gnl|extdb|pgaptmp_001971	K03975	dedA; membrane-associated protein	MGPWVLLGVALIVFIESGVLFPILPGDSLIFAAGLLHTQLNLNLWLLIGVILLAAFLGAQIGYWLGRRYGRGLFKPDARILKTEHLEQAENYFARYGGRSLILGRFVPIVRTFVPIAAGTARFPFGRFVVFNTLGAAIWGVGVTMIGALLGDRPWVHDNLEIIILLIVLVSVIPMVVEVLVQRRKAKQSD

gnl|extdb|pgaptmp_001972			MDELLVRKRLPEPVVVADRLPVRQRRPEDLYEAGANLLGIALVLLLGVYAHSTTLGVTEDVRVAFGSVIRQLLLVPVSVIQGLFVILAPTAVIFALARRGRIRSILAVLVTGAVTAILGWLISMLIPHLPVPLVDSLLVNTPSGAVTSVDVVIMVLVAALTVAGDGSRVKSIRYSWYGIWFLLVVGVIRGTTTVAAVLVTVLLGRMIGCLARWILGFSDRRATPTDLVEALLTVGITPKTVIRADLDQELDSWRITESDRQPDFASGLVNPELEREHLAGLCRLKQAEPHRSSFADRHYQVVTADGRHLDLHVLDPSRAITGTIGDLWDNFRLRGLSRWISPTLKANAERAVLTTHVAVEAGAHTPPPVGIAEAGDSVVVVWEQLPPVSTLLALRHHEVPLGDEVLTQAWEQLLSAHSRGVSHRNLDVDSLVLDADQQLWILHWDQGEVATGELNRHIDRAQMLAHLALVAGPERALASARRYFSVSELLAISLVLQGAVLPPGVRGQLRRTKVLEELRSNLAELTEATPQVTTPLRLQRFSLRTVLMAILALAVLVAVVGGLNFNAIMNAAQDANAWWMLAAFLIGSTTWLAAAMPLVAFAPKKISLWQATLAQMGGSLANIVAPAGVGPAAFNLRFLNKQGVAMPLAVATVTLVQISQFLTSVVLLLGIVVITGTSLDIPIPTMTIVWVLAAILALVATLLAIPMIRNWIWAKVKPTWEQVYPQLLWIMGHPKELGFAMAGNLLGNLGFIGAFAASLAAFGYYLSPMTVTITFLVSTTLGSIIPSPGGIGPVEAALTGGLQVAGIPGAVAISAAVIYRLVT
gnl|extdb|pgaptmp_001974			MDLFSAGGLFVAVIMYAISVSPSLLPRRGFWHGLVSGTLMGAGYALGWIGQNLLTYAAHRLGFRVELSETAQIWVPTIFWVAVGLWFLRSVIGSYLSSRRAARLVEMRPESWREYLLGLLITLVFFAALVGVARAGAWVYTQLIHWLGQWMYEPLAGLLAATVLVVLALFVSNKVVLGGFLMFFAREAERRNNRTAAGVHEPVVAERSGSPTSLCSWESVGAQGRTFLGRGPHRHRIGQVMGETALEPIRVYAGLVPGSVDFAEEAALVVQEMHRTGALDREVVLISVATGSGWVDEWIVQSMEYLTRGNCATISMQYSYLFSAAIALTQKELCLRSAEVLFAAVRAEIDQLPPERRPKLIISGESLGAEAAQAPFLNFADMRGRVDGALLVGAPYRARISRELTQGRHRGSPEVAPVYDSGKHARFVNEPGQLDRDLFGREFGPWEHPRIIFAQHASDPVVWFNRTVAFREPDWLRERVGLDVSPNMRFTPLVTLVQLIGDLPIAGTVPGGHGHTYTEELLPSWARILDLDVSPELLARIGRAIRSDVESSGRR
gnl|extdb|pgaptmp_001975			MSAHNFTDETDLPQPLPPAFQEVAPGYRLDDPHAAPTLRWGILGAGGIAHTFAATVSAHSSGQIAAVGSRNLDRARAFAHEFNLPHAYGSYEELVASPHVDAIYVATPHIRHRDDALLALRAGKPVLVEKAFTMTAAEAREVFDEAATRNLFVMEAMWSRHLPHYRFIRALIESGAGGQLVAASADHSQWLRHVPRMVRPELGGGALLDLGVYPLHFLHHALGRPTELVAAGLPTGTGVDASEVILARYPAALGVASATMDGINSTAGTLTFANLAVELPEQFYRPTVVHLRTFPDRAEGGTEQLVTTWDARVPGGFQYQAAEVARCVAAGLTESPVVTWADTLDVMEMMDEVGRQLAAAGTFRE
gnl|extdb|pgaptmp_001528	K07071	uncharacterized protein	MNVSATGHLVVSGGSGLIGRALIEAARARQIPVVQLVRGLARTDEESQWDPLGGRIDSQVLVGARAVVNLNGASIGRLPWTRSYRRKLISSRLAPTRTLASALRELGAGAPAFISASAVGYYGNQPGRVLTEESPVGNTFLAGICRQWEAEAMAAAPASRVVTLRTAPVVSRQGVLKPMVRLTKLGLGGRLGPGSQYLPWISLVDEVEAILHLVDSDWSGPVNLTGPAPATAGELGRALAKQLHRPYWLPTPAWALRLALGADAADSLLLADARVVPAALLASGFRFQHRTVDEAVAAALAAKS
gnl|extdb|pgaptmp_001997			MLAASDLHDHVAQLSPPARRILIDGPSGAGKTTLAQQLAPLGYLILHLDDWYPGWDGLAAGSALTEDLLTSDRPAFPRWDWAKGRVSEWVPVDPTRPWVVEGCGSLTPGTAALADLRLWCDVPAEVAHQRGLARDGAAYEPWWERWHRQEQQHWERHRPWTLADIRVDCRDADS
gnl|extdb|pgaptmp_001996	K01552	ecfA; energy-coupling factor transport system ATP-binding protein [EC:7.-.-.-]	MNTEVSLSKALTLAAHGWSWRYAGREAWAVEDLTLQIPPGQRVLLLGASGSGKSTVLAGMAGLLSGEDGEERGAFTLGGVPTSSGPVRGEVALVQQDPDSQVVMEQVGDEVAFGLENLGVSPEQIWPRVEQALAQVGLDVPLDHPTHQLSGGEKQRLALASALAMEPGVLLLDEPTSNLDPAGVREVRASVASALTGSDTTLIIIEHRVATWVDLVDRIVVLSPTGILADGEPGEVLTAYRDQLLAAGIWVPGVELPVQRLVGERTGTCLQATDLTVGYEAGEPISVHAQLDLGAGISTCLVGANGAGKTTLALTLAGLLAPLAGSVTSAGHVQMVFQEPSYQFLRHTVREELELSLQFSDLSSEERAARVDQFLALMRLRDLADAHPQSLSGGEKRRLSVATGLIGHPPILLLDEPTFGQDRNTWLALVHLLQDAVRAGTSVIVITHDEELVEVLGQEVVTVHPQRELRVEPPPPSAGILDRVNPLFRLVGLALMTVPMFFSVDLLSTAVALALVAGLLPLVGWGPRQLLRRIWPLLVAAPLAGISMLLYARPGGTVYWSWGPAAITQNSVELSLAIAIRVLALGIPAIVLLSSAQPTRMADALTQVAKLPPRPVLAALAGIRLMSLMLSDWQALGRARRSRGVAPQSKWKEFFTGSFSLLTFALRRASSLSVTMEARGFGAPTRRSNARTSPVGWADLWMVVVAIIIPTLA
gnl|extdb|pgaptmp_002009	K00324	pntA; proton-translocating NAD(P)+ transhydrogenase subunit alpha [EC:7.1.1.1]	MRIGVPREPDSQPLVAATPNTVKKLKKLGYDVCVEAGAGTRASYPDYQYEEVGAELVDRNTVWGSDLVLSLDAPSDEDLALMHEGEVLITRLAPARSPELLEKLRAAGITAMAMDAVPRISRAQAMDVLSSQANVAGYRAVIEAAADFGRLFTGQVTAAGKMPPAKVYVIGAGVAGLAAIGTAASMGAEVTASDVRPEVAEQVESMGARFVGLHRDMEESSDGYARELTAEETAAVNRLYAEEAAKADIVITTANVPGRRSPILLDDAAIAAMKPGSVVIDMAAANGGNVTVTRPGEKVMTDNGVIVNGPLDLPGALPTQSSQLYAQNIVNLLKLLTPEKNGEIDLNLDDEIIRGITVTAQHDLLWPPPPVKVSAAKPSPAAVTPVEPTPEEAEAMAIRKRTIWGAWGGFGAALLVALYLVTPMAAIPSYMVLALSIIIGFYVITNVSHSLHTPLMSVTNAISGIILLGAILQVGSDNLAVTIISFIAIMLASINIFGGFTVTARMLTMFKG
gnl|extdb|pgaptmp_001994			MSASERLAELNLTLPPVAAPVAAYIPALVDDGKVRTSGQLPFQDGVLVSTGACGSPNVELSSAQVAARQAALNALAAAAEAAGGLELIESVIKVTGFVSSTPDFYGQPQVVDGASELFQVLFGTSHIRSAVGVAALPLDATVELEVEFKLASSELRPIQN

<table>
<tr><td>gnl|extdb|pgaptmp_001990</td><td>K03704</td><td>cspA; cold shock protein</td><td>MTTGVVKWFNSEKGYGFITPDDGSDDVFAHYSNIQSNGFRSLDEGEKVEFEITTGPKGLQASEIVRLNG</td></tr>
<tr><td>gnl|extdb|pgaptmp_001998</td><td>K07090</td><td>uncharacterized protein</td><td>MTVTNSEQLWARPNRRQTILLIVLGLGAGLLSGMFGVGGGIMIVPGLVAIVGFAPRLASGTSLTTIVPLALVGVASYAAQGDVSWEAAGLLALGGIGGAQIGTWLLSRISSRRLQIVFAGFILFSVVMMFINVPSRAAVLEINWATAVGLLVVGILTGTLSGLLGVGGGIIVVPALMLIFGASDLVAKGTSLLMMIPSALGGTIPNIRRRNLNLPAALIVGLSASTTTLLGSYLAHRISPQVANLLFAVFLLFVAVSMLRKALRTPR</td></tr>
<tr><td>gnl|extdb|pgaptmp_002000</td><td></td><td></td><td>MCGRFTMFDEDDELVALFDIDVMEGEHLPSYNQAPSQWVRAVVGNQPRVLTMQRWGLVPHWAKAGFKPLINARSETLTEKPSFRVAASRRRCLIPTNGYYEWMTQPDGQKQPYFLSAPVPAEADGAAIQPGPAGFPTPVLAMAGIYEFSRTERGDELVTCAVITRSAPDELGRIHDRMPVFVPPELHTPWLDPELQDRDQVRDLVAAIPVLPLATRPVQRAVGSVRTQDPGLIFG</td></tr>
<tr><td>gnl|extdb|pgaptmp_002001</td><td></td><td></td><td>MEENAVWAVFRVVDLTGVLLNGVLGGKLARQKHFDAVGFMILAVMSAMGGGIVRDVILQHGPPVAITDPFYIGTALVGASIAFLWRLDSRGWRIALILADGLVLGSWAATGAMKTLSMGFGLMPALLLGLTTAVGGGMIRDISAGNVPTVLGGNYLYATPALVSAGIMVAFFNLGYPMWGMIVATVVGSSFTALAHWRRWRLPEHTDWTLTLSSEQLRYLLDRKAAARKRTRTGLDESFEETGLPRTDEAQTRDEEG</td></tr>
<tr><td>gnl|extdb|pgaptmp_002003</td><td></td><td></td><td>MGTKAGIILGLGIGFVLGARAGREKYEQIKGVTRQLRTLPIVSRPLDAAGEKVADVVRSKGNEISDAVADVVKEKVFGMPTNRLVIDATVVEHPRENAPESPDVSAPRNS</td></tr>
<tr><td>gnl|extdb|pgaptmp_002004</td><td></td><td></td><td>MTNNGALSLPPDEEILGIFWEAARQKLASASWEDLFGPRLRSSLRPPAMQLADDAEEASDLARQIRDAGAMIVRSPAADFSEDSPAPEAGDLTIICDGGGVPLVLVRTKQVDRVGDEIVEELVSLYPTSGK</td></tr>
<tr><td>gnl|extdb|pgaptmp_002005</td><td></td><td></td><td>MSEISLVTPRGVNLAGNFVVPVDATDAAVLFSHSFFNNRESGKYFERLAAAYRKLGYATLKFDYSGHGESDDDIIVVDHQEEDLRAASGWLADQGFDRQLLHGHSFGTLAPLKARPAAVQSMILSGVITGPLSFDWEQIFSPSQLDELEQTGRTRIVDDSPSSRQYFEISKQTLQDLSLNRAADLVDDLSYPVLLLHDIDDEQAGLLEMTTDVFARLPDGSRVEAVRDANFGPGEKLPYLAGLCQEWARQHLPVRP</td></tr>
<tr><td>gnl|extdb|pgaptmp_002007</td><td></td><td></td><td>MTEQEILTLARELMDQHGLQQWNLTLSRAKTQAGCTYPSRRQITLSRLLLPKFSPDQVRDVILHEIAHALAGPRQGHGPAWQKLARQLGATPQARLNLPQPVLESSWVGTCPGCGLQVHRHGTPRRVRSCARCSPQFDLDYVFDWQFRGARKVPGGQYVRELTAIRRRRPS</td></tr>
<tr><td>gnl|extdb|pgaptmp_000466</td><td>K01595</td><td>ppc; phosphoenolpyruvate carboxylase [EC:4.1.1.31]</td><td>MPELRTNASTDELPEQIRADVRLLTTLLGRVLSESGSPSLFEDVEALRHATIAAYRDDSPEAFAQAAEIAESFTVQRADEVARAFTVYFHLVNLAEEMQRIRQVREGRPEVEGAVDTIPKAIAQLSQEIGEEAARRHLEHLRFHPVFTAHPTEARRRAVASSVRRLSALLAERAFTTLDEAGELRMERRLLEEIDTLWRTAPLRSIQPGPEDEVRSLMAIFDETLFTTIPRIYRYVDDSLQGENAGREQPIVQPFMRLGTWVGGDRDGNPFVTASITKKAMAIASDHVLRGLQATAERIARSITLEADETPASPELQDLWQRLAMIDEVGAADAEVRAPNESHKQVLMLIATRIAATRTRNADLAYAHPDELLADLQIVQRSLVLAGANRQAYGSLQVLIWQVETFGFHLAELEVRQHSQVHGEALAELEAGGELSDQTREVLDVFRAVAQIQHRYGPRAAGRYIVSFTRSAEDLAAVYRLAEYAGAEVTLDVIPLFETYNDLRSARAVMSEAVELPQFKARLEQTGGQLEVMLGYSDSSKDVGPVAANLALYDTQAELAEWARARGFELTLFHGRGGTLGRGGGPTNTAILAQPPHSVEGRFKLTEQGEVIFARYGDPTIAMRHIDQVAAASLMAIAPSIEQRNVAAATKYSAVADQLSKVSRARFDSLIQAEGFAPWFATVTPMEEIGRLQLGSRPARRGLSVESLKDLRAIPWVFAWSQARINLAGWFGLGSALEAVGDLELLRRAYEEWPLFRTVIDNVGMSLAKADPRIARRYLELGGRPDFTELILEEMDLTTSWLIRIVGGEKLVENRPALRRAVELRSPYVDALSLIQLRALRMLRSDEEAGPEWEHLLLLSVSGVSAGLQNTG</td></tr>
<tr><td>gnl|extdb|pgaptmp_002019</td><td></td><td></td><td>MSARTRANPRPALFVLLILVAVVLAWVAVRLLGSSPPEQPVAAPEPLEPEPVTPTLDFTGFDPGNIISDDVFYHSEAMNQEQVAAFIAEVNHGCRTGDAPCLADYREDSLTFPANDYCFEFTGQSNDSAAAIIDRAAKSCGVNPQVVLVMLQKEQGLLTASSYNLTPGRYDIAMGYGCPDTANCDPQFFGFSNQVYHAALQLRRYANEPGLYSFQPQMDNSISYHPDPACGEGTVWIENYATAGLYNYTPYQPDEAALAGTPGPCSSVGNLNFYAYFRAWFG</td></tr>
<tr><td>gnl|extdb|pgaptmp_002020</td><td>K25307</td><td>wzb, etp; low molecular weight protein-tyrosine phosphatase [EC:3.1.3.48]</td><td>MTYRVLMVCTGNICRSVMAETVLAQRVDGLEVQVDSAGISAEEEGNPIDYRAARILSERGYRVPDHRARQLEPADLADFDLILAMTDGHRRGVARLAERSGIEPRDVRMYRSFDPAAAGNLDVPDPWYGDLSDFAQTLSTIEAVTPGLIEFLAQRA</td></tr>
</table>

gnl|extdb|pgaptmp_002021	K09164	uncharacterized protein	MDAINHRAAWEQWREHRNETLRQPHGWLSLVNLEWVGDTAAPLSSFPGIWSAKDHQVTATFSPEDQVTRDGHPVVGQVHLEIPRGESDTSLLDARGRQAEVASRFGRIAVRTRDPEAPTRTNFSETRTFDFDPKWIVTVEWEPYPEPTEVTVPSAHQSKPMTLTAQGSATVFGQKVTITGSDRDHLGLIFHDETNGNQTEGWRSAPAKLDDGRLTIDFNRAVNFPAHFTPYGTCPTPPAGNTLPVAVTAGEKKNR
gnl|extdb|pgaptmp_002022	K09815	znuA; zinc transport system substrate-binding protein	MRKLYKSVLAVAVGSLGLGLLSACSDAGAQSESSDATGDQVSVATSIYPMTFLAEQVVGDSGRVIDLAPTSGEAHDLELSPRQVSDLMKADVALYLGDGFQPPVEQAVAQRDGASVDALQVVSEGELRSGDPHIWLSPELMSQVAGSLADQLAQVDPDHADQFQANAERLQQELGQLDQEYRTALAGCAAGSLLTSHEAFGYLADRYGFEQHGVMGINPETEPSPKRLQEVQQLVKAEGIDSLFIEAGDSSGQKLAEILELTPKELHTLESKPEGMDYFEAMRANLEALTNGLGCAAG
gnl|extdb|pgaptmp_000003			MNYTQHGHQVREHRLTVPLSHREPHGEQIELFAREVTRPGGEKLPPLLFLQGGPGGAGPRPGDFATGWIGEALADYRVVLLDQRGTGQSSQDAVLPASDRALADHLSLFLQDQIIADAEALRAHLEVDRWVTLGQSYGGFLTLAYLSAHPEAIERSFITGGLPGLGSIDEIYRLTYRATAARNRAYFRHYPQDEATLRAVAGHLEQVEERLPTGERLTPTRLRMLGMSLGTQTAYDLLHYLLEGPFVSRGGERRLTPSFLAGVGAQVSHAGRPLYAALQEAIYGPTSPAGTRWAAERLAGEFAGFNLEADPLDQSEPWYLTGEHMFRAFFEEDPSLVPYLGAVDLLAEKTDWAQIYDQSVLSQIDVPIAAAVYYDDMFVPRELSLETAALLGARTLVTNEFQHDGLRAG
gnl|extdb|pgaptmp_000114			MRRSGSHPKSSRPRFDDRPRNGTGSELVELLHRIDGSSYGAYKRALGSWNFGAYRVDFTRVQADPYAPPSSIRVRYRRDQVDLSETDLHSQDRQLATADFLARRAGDLIRQHAPTLSIAPCGPEILERSSARVGSEDFELRIAVRLPARGRTVLGRQAALIFTEQLPRVLRELFNFSNSAHREHWLAHVHALADHRALQQLLVERDLVGFIADGSQLARASGVSAQPLRSGVPFAAPDSRRQQVELPHAGVVSGLAIPAGITVLVGGGFHGKSTVLTALEQGVYPHVPGDGRELVAALPSAMKVRAADGRPITRVDVSAFINHLPTGSSTTRFSTQNASGSTSQAASIIEAIQAGSQLLLFDEDTSATNLMIRDARMRTLVQADQEPITPLVDRIRSLPSERGVSVIMVTGGSADYLDAADLVLQMDAYRCLDATERAGEVMAAFPRQRTDLPDFPTPLPRLVVRVRPGGERSRTKTQGVDRIELDRQGVDVTDVEQIVDPGQTEAIAWIIRGVTEQLADGHRTLEQLVDEVLDRVEDRGLDALVDFGARPFPPFLARPRKVDICAGINRHRSLKIG
gnl|extdb|pgaptmp_000111	K00525	E1.17.4.1A, nrdA, nrdE; ribonucleoside-diphosphate reductase alpha chain [EC:1.17.4.1]	MVDNLTDTGLETAMNPELDYHALNAQLNLYGPDGTIQFDADRKAARQYFLQHVNQNTVFFHNLREKLDYLVEEGYYEQEVLDQYSFEQIKALFKRAYGFKFRFPTFLGAFKYYTSYTLKTFDGKRYLERFEDRVCMVSLYLARGNYALAEKIVDEIMTGRFQPATPTFLNAGKKARGELVSCFLLRVEDNMESIARGINSSLQLSKRGGGVALSLTNLRESGAPIKRIENQSSGVIPVMKLLEDSFSYANQLGARQGAGAVYLNAHHPDIMAFLDTKRENADEKVRIKTLSLGVVIPDITFELAKNNEDMYLFSPYDVQRVYGKAFSEISVTEHYREMVDNPQIKKRKINARRFFQTLAEIQFESGYPYILFEDTVNRDNPIAGRVTMSNLCSEILQVSEPSEYNPDLSYAHVGKDISCNLGSLNIAKAMDSEDFSLTVETAIRALTAVSDLSNIESVPSIAKGNARSHAIGLGQMNLHGYLGRERIHYGSEEGLDFTNIYFMAVAYEAIKASNKIARERGETFDGFEKSKYASGEYFEKYVNQDWVPQTERVRELFANSSVKLPTREDWAELARDVAQYGMYNQNLQAVPPTGSISYINNSTSSIHPITSKIEIRKEGKIGRVYYPAPYLTNDNLEYFQDAYELGPEKIIDTYAAATQHVDQGLSLTLFYPDTVTTRELNRSYIYAWRKGIKTLYYVRIRQMALEGTEVENCVSCML
gnl|extdb|pgaptmp_000110	K03647	nrdI; protein involved in ribonucleotide reduction	MQPNVVYFSSATENTKRFVERLGVPAHRIPLRRGDEPLAMQDDYVLVVPTYGGGNHRGAVPKQVIKFLNDPENRSHCRGVISSGNTNFGTAYCLAGDIVSQKVGVPHLYKFELLGTPEDVQRVKEGLRKFWSTI
gnl|extdb|pgaptmp_000109	K06191	nrdH; glutaredoxin-like protein NrdH	MNVTVYSKPRCVQCDATYRALDKLGIKYSTVDVTQDADSLQYILGLGYQQAPVVVVGESHWSGFRPDRIKALAEELVGVAN
gnl|extdb|pgaptmp_000108			MTDATPPIMNVESFNLDHRAVAAPFVRVADRKELPGGDVLIKYDVRFTQPNVAHLEMDTVHSLEHLTAELMRNHTDRLIDFSPMGCQTGFYALTLDIGYDDFLTLLEQTLTDILAATEVPAANEVQCGWGAHHTLAGAQEAARQFLAARDQWSTIFRPDHREAQQ
gnl|extdb|pgaptmp_000107	K01243	mtnN, mtn, pfs; adenosylhomocysteine nucleosidase [EC:3.2.2.9]	MRVAAVIACAMTEELEPFLARAQHVSEVGGPASSRDHQRFYRAELAQRPVALVLSGIGLVNAAGAATVALAHFSTDRYLLAGTTGGLGTRVQVRDVVAGTRARYFDADATGFGYEPGQIPQMPAEYVGLEAPAFATHAGLVLSGNSFVQADIAEQTRARFPGALAVDMETAAAAQVCYQHGVGWLSLRAVSDLCGPEAGQEFHVGAEVAAELSAQAVERYLAD
gnl|extdb|pgaptmp_001978			MEPTPATPNPYAQADPDLYHRVRPGYPEAALEALLTRVGPVRSVADIGAGTGIFARQLLDLEPQVRVEAVEPAPEMPRGAPTQPRLTWHRGTSEATGLAPASVDLVVWAQSFHWVDRERTGPEASRILVPGGHGAIIQNQMEVGEPWVHRLSRIMRSGDVIRADWNPRLPGFKTRLVQVFPWVQTLTVDEVMALARTRSSYLGSGPARQRRMQANLNWYLRDHLAYPKEGQVRIPHLTFLWILDPA

gnl|extdb|pgaptmp_001995	K16925	ykoE; energy-coupling factor transport system permease protein	MNMTKQSGWRVVDLVTAAVLGVATGIIFIIWNQIGYLAFTSLDVFTAGLGGLVNGIWYLGGPLGGLIIRKPGAALFVEVVAATVSMALGSQWAIETLFSGIA QGLGAELIFALFLYRRFGAGVAALSGLGAATGAMILELFVGSTPNIAYAPIRLFTYWTTSSLSGIVLAGLLAWVLTRALAQTGVLDRFASGRELRRRV
gnl|extdb|pgaptmp_002008	K00325	pntB; proton-translocating NAD(P)+ transhydrogenase subunit beta [EC:7.1.1.1]	MDLLIHIVQLAYLAAAILFVFALSGLSSNKSAQRGNRAGITGMTIAVSATIVLALASEPPRGIIATILLIAVAMLAGAGIGIWKAQRVEMTGMPELIALLHSFVGL AAVLVGYNSFLHPDALAQSLGGFHLGEVFLGIFIGAVTFTGSVVAFLKLSGKISGAPLTLPHRNLLNLLLIVISAGLMVWFIAVGHSVDAWIPLGVMTALALLLG WHLVAAIGGGDMPVVVSMLNSYSGWAAAAAGFMLNNELLIITGALVGSSGAYLSYIMCRAMNRSFISVILGGFGTDTSSAPTGDEEVGEYREIDAAGTAEL LKNSRKVMITPGYGMAVAQAQYPVAELTKALRERGVDVTFGIHPVAGRLPGHMNVLLAEARVPYDVVKEMDEVNDDFGDIDTVLVIGANDTVNPAAEEP GSPIAGMPVLKVWEAGNVIVFKRSMATGYAGVQNPLFFKDNTQMLFGDAKASVEAILRAL
gnl|extdb|pgaptmp_002002	K22736	VIT; vacuolar iron transporter family protein	MSDTPTPSRRQIRRWRRYLAEERLEADTYRNLARRQRSPHREIMLELAAAEGRHEQYWLELLGDQAYPPPRVPWRRRLMSSLANRFGSVFVLALAQRSEQ RTGYDDDHDVPDQMAADEHIHGEVVRSLAAESRAHMAGSFRAAVFGANDGLISNLALILGVAATGMTPSMVLATGVAGLLAGALSMGAGEWISVSSQR ELLSASTPDDDAHRSVPALDVNANELALLFRARGESPETAQEHAERVFAALGEAGDSASGQLPLRSALGEGARQTSTEAVGTPFRAAVSSFIFFALGALLPLIP FMFGVGGWMPIVISSVLVGITLLGTGGVVGLLSGGTPWRTALRQLAVGYGAAAVTFALGTLFGATIG
gnl|extdb|pgaptmp_002006	K03578	hrpA; ATP-dependent helicase HrpA [EC:3.6.4.13]	[illegible] SGLIGHTQPRRLAARSVAERIADELGQTVGREPGQVIGYQVRFTDEVGPTTLVKLMTDGILLNEIQVDPDLRRYDTLIIDEAHERSLNIDFILGYLARLLPRRPDL KVIITSATIDSDRFADHFSQFTGDRVPVIEVSGRTYPVEIRYRPLSPDQSGTPTQTPTEEAEPSAAHTPGAGAPQLVLEDPDDDLATLGYGLGEAIDLEQGLCGA VDELLSEPAGDILVFLPGERDIREAQVALEEFLRERRPPVSVELVPLFARLSAAEQHRIFAPHSRRRIILSTNIAETSLTVPGIKYVVDAGLARISRFSNKTKVQRLPI ERVSQASANQRSGRAGRLEDGIAIRLYSAGDYAARPRYTEPEILRTSLAAVILQMAALGLGEVSQFPFLDPPSSRAVRDGVQLLVELGALTEAGKLTSIGRKLAR LPIDPRLGRMLLAGQENGCASEVLVIIAALSIQDVRERPLDAQAAADQAHARFNDSRSDFMTYLNLWRYLNVQARDLSGSAFRRLCHREYLHYLRFREWRD VVGQLRQMCREMGIQVEALGEPDRGEIRQYADPASAVVEFGRGVRAVDYDQLHRALLVGLLSNIGTWEQAKGTYLGARATRFTVWPGSGLAGGHPPWI MAAELVETSRLFARTVAQIKIEWVEHAAQHLLTRSYSEPYWSRAKGAALIKERVSLYGLTLAADRSVLLASLGDRQLGASVAPQLAPPQPGSIAAIAQGLFAQ AGSGGWSSQFFGPKPEAEGLTARELAREMFLREALVEGNWRVSYLKFQRENQELLDEAREVEQRSRQAGKVAGEEELFRFFDERVPAEITSARSFENWWK KTRPDQPRLLNYRWEDVIHDVSRGGEDGFPDHWQQGELSLPLSYDFQPGSDTDGVTVTIALAQLPQVQDEGFDWLVPGLLSQLCAGTIRALPKEKRRLLAP APDVGEQVAKRLTHRGETVLEAAVSPAEEAEDPRSLGASLDRLAQWAQQTGKAKGPLTVSPQSVNPVPEPTSEAPAPTPSPEVTRRFFPAFAQAVANLRGV ELTEADEQHVSQHLPAHLQMHFQVVDRNGKVLARGSSLLELQRRLAPQADRAIHQAVRQAVRQAGKERPGRAHAPTADAGSWQQEEVTEFPSTPIAPVV ETTNAQGLTVRGYPALVAAPDRDLVSLRLCATAEQAELSHRDGVARLLERQLRLPLTRVTTRWSGREALLLAASPYPSTEALVAAAQLAAARTLRAELAPQPI RRAEDFAALVSRARDRFEEEVYRLLQLTVKSLEAQRRVEEALDRHPEESLEPVRRQVRRELKRLLSADFLTETDRRTLPSLPRYLDALAIRLDRAARGPAALRTD QERWSNLLELRTRWEDARDRAAGRPLNLQVHDRLSRVKWLLEELAVSQFAEQLGTAERVSPQRISRQLDAIWEG
gnl|extdb|pgaptmp_000001	K00526	E1.17.4.1B, nrdB, nrdF; ribonucleoside-diphosphate reductase beta chain [EC:1.17.4.1]	MTQSNHEVFEAINWNKIEDEKDLEVWDRLTGNFWLPEKVPLSNDIQSWATLNAAEKLMTNRVFTGLTLLDTLQGTVGAVSLIQDAVTPHEEAVYTNISFM ESVHAKSYSSIFSTLLSTEEINDSFRWGEENPQLQKKAEVINALYEGDDPQKRKIGSVMLESFLFYSGFYAPMYWSAHAKLTNTADLIRLIIRDEAVHGYYIGYK YQVANRNASQERLDELKDFTYQLLYDLYDNEEQFTADLYDPLGLTEDVKKFLRYNANKALMNLGYEALFPADETNVNPAILAALSPNADENHDFFSGSGSSY VIGEIVDTEDEDWDF

gnl|extdb|pgaptmp_000871	K18675	chbP; N,N'-diacetylchitobiose phosphorylase [EC:2.4.1.280]	MQYGYFDNDHHEYVITRPDVPVSWTNYLGTKDFAAVLSHNGGGYAYYKSPEYGRITRFRQNGVPLDRPGHYLYVRDNEDGDFWSVSWQPVGKPFEGDGPDVASYRSAHGMGYTRYEADYRGIHAEQTVFIPLSDDVEVWDVRVRNDSDQTRHLNLAAYVEFSFHTISIDNQNLQMSLYSAGSSYEDGIIEYDFYYEPWTYHFFTSSSEPSSWDSLRDSFIGPYRTESNPLAVERGAGSCQSGTTQNHCGSLQHTVELAPGESARIVYLLGYGSREEAGRPMQAKYSDLANVDAEWEALNQYWAAKRSKLQIDTPNEGMNTLINTWTLLQAETCVQWSRFASFVEVGGRNGLGYRDTAQDVMSVIHSNPTKTHQRLVELLRAQVSSGYGLHLFDPKAFDPDAVPLPDVPSPTVVPTPSTEDLIHGLEDTCSDDHLWLVPSVVEFVKETGDFSFLDRVIPFADGPEATVYEHLKAALDFSSAQIGPNGVALGLRADWNDCLNLGGGESALVTFLHAWATRSFLELARALGRDEDVAKYEAELARIERVANEQLWDGAWWIRGYTRDGVKIGSEANEEGKIFLEHQAWPVIAGITSQERGEQSMNSVRELLGSRYGNHLNWPAFTKVDDTVGFVTRVYPGIKENASIFSHPNAWPIIAEALLGRGDEAVAFYDAISPYQQNDNIEVRGAEPYAYVQFIYGRDHERFGLAQNPWLTGSAGWMYTAVTKYILGIRPGLDGLTIDPAIPHDWPGFSVTRQWRGATYQIRVQNQGQVGHGVKSLRVDGVELDPSQPIPPAEPGTEVAVEVE
gnl|extdb|pgaptmp_001929	K00963	UGP2, galU, galF; UTP--glucose-1-phosphate uridylyltransferase [EC:2.7.7.9]	MSGLLAAQEKMRAAGVSEAAIEVFSHYYHQLEQGVTGMIPEDTIEPYLDPPLLADLTVSEEAARAALAQTAIIKLNGGLGTSMGLDRAKTLLEVREGKNFLDLIVGQVQSVRERYDVQLPLLFMTSFRTDDDTQSYLARYPELAVGDLPLTFVQNQEPKLRADDLTPVSWPADPTLEWCPPGHGDLYTALIGSGILDQLLAAGYRYASVSNGDNLGAYPSPTIAGWFASTGAPYAAELCRRTINDKKGGHLARRKRDGQLILRDTAQTLPEEMDFFTDEHRHPYFHTNNLWMDLRQLKDRLTGGSAVLGLPLIRNEKTVDPTDSDSTPVIQVETAMGAAIEVFPGSTAICVGRDRFLPVKTTNELLLLRSDVYRLSPAGRLETDLDHSPEVALSGKYYKTIAKFDQRIPSAPSLRAATKLQVDGDWQFEAGVTIAGAAEFGPEGGTVTAGVHGSAQ
gnl|extdb|pgaptmp_001084	K01989	putative tryptophan/tyrosine transport system substrate-binding protein	MKKSLSLLALGAAAVLGLSACGTGSSAQSEGTDKSESSQSAPASAESYQIGITQLMSHPSLDAARDGFVQAIEDAGLDVTFDEQNANGDQSVASSIAGTFNSGNYDLILAIATPNAQAVAQAITDKPVLFTAITDPVDAGLVDSMDQPGGNMTGTSDANPVKEQLQLIKDVVPDAKKVGIIYSPGEANSVVQVEWAKEAVKDLGLELVEAPAVSSQEVLQAAESLSNVDAIYVPTDNVVVTSLETVLKVGEERQIPVFGAEGDTVARGAIGTYGLSYFDLGYQTGQMAVRILVDGEDPATMPVETLSTPMLYLNKGAAERMGVELPESLLAEVTPENLTE
gnl|extdb|pgaptmp_001083	K05832	putative tryptophan/tyrosine transport system permease protein	MITAVELGLIYSIMALGVYLTFRILEFADLTVDGSFTTGASVAAVGILAGVNPILSTLAAFVAGVGAGLITGLLNTKGNVDGLLAGILTQIGLYSINLRIMGTSNLGLLRVDTVFTPMREAGLLGTWLGPAVMLPFAAVVLLALIWFLHTDLGLAMRATGDSPKMISSFGVSTDFQKILGLCLSNGMVAVSGALIAQYQGFADVGMGIGMIVSGLASVIIGQAILGRLTIWRAATAVVVGAVLYRVVIQLALLVGFNPNDMKLISAVLVILALLLPQWRGFGRIRRALGLRWRSPEEKTRARLARENAALEED
gnl|extdb|pgaptmp_001082	K05833	putative tryptophan/tyrosine transport system ATP-binding protein	MSVTPALSLSSISKTFFPGTVNERRALVEVDLELAPADFVTVIGSNGAGKSTLLNVVAGSLRADAGTVKIGERDVTRLNDYRRARYLGRVFQDPSAGTAPHMTIEENLSMAQQRGRTRSLRLGVTSRKRREFREALGILHQGLEDRLGDWVGLLSGGQRQSLSLLMATFTRPQILLLDEHTAALDPQRAELVTRLTGELVAAHNLTTLMVTHNMEHALRLGNRLIMMHEGRIILDIGGEEKAAMKVPDLLAKFESIKGLELSDRALLQ
gnl|extdb|pgaptmp_000236	K07552	bcr, tcaB; MFS transporter, DHA1 family, multidrug resistance protein	MSNPPSGAGVNPGDELSRRQRLTYVVVLGMLTALGPFTVDMYLPAFPAIQHQFGVDPAAVQLTLTGTMVGFAAGQLIVGPLSDKVGRKVPLIVAALVHIAASIGVAMAPDIIWLGVLRVIQGFGAASSGVVSMAMVRDLFGGRRLVKMLSRLALVNGLAPVIAPVLGSQLLAIMSWRGIFWVLAGYGILVSIALVVLIAETHPPARRLALQKPLRERYRAVLSDRIYVGSLVVTAMNFTALFAYLSSSPFLFQQVYGMNPQQYGILFAINSVGVIVGVQTSAHMMHLGRWSPQWIIATALAGQIVLGAVIFGLSLAGAGLWGTIIPLWFFIFCCALNFPAIQFLALANHGEEAGTAASLLGAVNFGVAGILSPIIGVLGVGSAEPMAGMMVVAALLGTAALWLIIRPR
gnl|extdb|pgaptmp_000975	K01714	dapA; 4-hydroxy-tetrahydrodipicolinate synthase [EC:4.3.3.7]	MDRLFTGSGVALVTPFLDGEVDWAALEQLVDLQLAGGTDALVPCGTTGEPSTLSSDEHDAVVKFVVERVAGRVPVIAGAGSNSTAAAATKANKMQDLGADGVLIVTPYYNKCTQNGLRQHFEAVADAVQVPVIMYNVPSRTGVNLAPHTAEQLSEHPNIWGLKEACGDLGQVQELFRRCRGKLPIYSGNDDQVYALLALGGDGVISVAANVAPKRMHELVQSYRDGQHEQALQLQEELAGLIDQLFTEVNPIPVKAALSMMGLIRDELRLPLTELSAKHRPSLKWELENLGLA
gnl|extdb|pgaptmp_001888			MQFGIFTVSDITPDPHTGRAPTEEARLQSIIRIALHAEQAGLDVFALGEHHNPPFFSSSPTTTLAYIGAKTTKLHLSTATTLITTNDPVKIAEDYAMLQHLVGGRMDLMLGRGNTGPVYPWFGKDIRAGIPLAIENYGLLRELWENDVVNWEGRYRTPLRGFTSTPRPLNGQPPFVWHGSIRSPEIAEQAAYYGDGFFHNHIFWPPEHTQQMVALYRERFAHYGHGTPEQAIVGIGGQIFMRPNSQDAIDEFRPYFDHAPVYGGGPSLEDFMRDTPLTVGSPQQVIDRTLGFRDYVGDYQRQMFLVDHAGLPEKVVLEQIDILGEQVLPVLRSEFERLRAPGTPSDPPAHSAVTAAPKSDSDEQAAPLHDTVTGASFYEQQPTLALSVEHAQQLLNQAEGR

gnl|extdb|pgaptmp_000212	K01104	E3.1.3.48; protein-tyrosine phosphatase [EC:3.1.3.48]	MTVTPLFDGVPNSRGVDGLPLASGGRTHAGVLYRSAALDTISDRGIATLRQLEVRAVVDLRSEAERERSPHRLPPNAGIDLIEAPITVSSLDPRELTELEEELAARPKVPPLAEAYRAMLSQAGEQFALAASVVARVSRTGGAVLVHCTAGKDRTGITAALLLELAGTDRQAVVANYCESERYLAGAWVQMMLDRLSREGMPVDDSVIKLVSTTPPDAIESALSWVDEHSGTAAQYLVESGLSGADVDQLRRVLG
gnl|extdb|pgaptmp_000984	K03784	deoD; purine-nucleoside phosphorylase [EC:2.4.2.1]	MATPHIEAENGDFAPAVIMPGDPKRAERIAHLILDEPRQVNSVRGMLGFTGTYQGQPLSVMGSGMGQPSIAIYATELFQFYGVERIIRVGTCGGISPKVSVGDTIVAMGAHTNSNFNDHRIPQVAFSALASYPLLSAAVSAAPEDLRLHVGAIFSSDAFYRVVPGQLEALAQYDVLGVEMETAALYGIAAELGKQALTVLTVSDHLLDHSGDMTAEERETKFQGALTLAAAAALS
gnl|extdb|pgaptmp_000985			MRLVAFDLDDTLAPSKSELPPSMGTALRSLLARVPVCVISGGNFAQFESQLLAGLNAEEVLLERLHLMPTCGTQYLRRQQGRWDQVYEEALSPEQKQRAIAALTVAAHDLGLWETDPWGDIIEDRHTQITFSALGQQAPLAAKKAWDPTGEKKAALVRLLSPALPDLEVRGGGSTSVDITARGVDKAYGMRKLVEQTGIAPERMLFIGDRLDEGGNDYPVKAAGWPTRAVGGWEETLTVIEELLAD
gnl|extdb|pgaptmp_000987			MAKKDSADQTASEPSAEQPAKKGRPTRTRREAELANKRPLVPNDRKLAKQIEREKRNEAYARQQLALQTGDERYLPYRDKGKARRFTRDYIDARWSFSEFLLPMMLLFLVVSLGIGLLGSNPELATTVMLAVTVALYSFFVFSIAEGIWVWQKIKRRFNAHYPKEIIPKGTWFYCYSRMIMARRWRSPKPQAARGEFPDGARKT
gnl|extdb|pgaptmp_000988			MSNFEALSTRVDRYLPAQIEGLRRLVQIPSISSDPARADQMAQSAQTVADMFAELGFNVELCQATAEDGTEGQPAVLAVKSAANPDAPTVLLYAHHDVQPLGEVDRWSYEPLELTEVGDRLYGRGSADDGAGIMVHVGALRALGEDLPVNVVVFIEGEEEIGSPSFTAFLEKYRDRLSADVIVVADSNNWTVDVPALTSSLRGVCSCDVKVEVLEHAVHSGMFGGPILDAVTLASRLIATLHDENGDVAVPGLGGNDRAEVEWQESDFRADASVVDGYQLAGTGDLAARVWTKPALAVIGMDVRPIAESSNTIAPQCTFRLSLRTVPGSDPRQSLSALTEYLQAQAPFGARVSVTPGELGPSYQADLSAPVAEKLRAALGSAWGTTPVAIGVGGSIPFISDFQAAFPGAQVLVTGVEDPQANAHSENESASRKTLRNATLAEALFLESLAE
gnl|extdb|pgaptmp_000991	K01802	E5.2.1.8; peptidylprolyl isomerase [EC:5.2.1.8]	MSRSRLAGAIATLGLLATLGLSGCAGQGGDSASESADQPTQSAQSDGGAQSGAAAEEGPEVDRDPSGSLPTITLPTEDSGPLMKPVSSSEPKVITAKTLTEGDGDKVGPDDFITVNYAGFLWDGKQFDSSYKSDGKSTPISFSLNQVIKGWKWGLNQTRVGDQVMLVIPPEYGYGKQANGQIPANSTLVFYVEILDTVPVNTDALKDAENTNAQLPVGISVQGDLGTEPEVIFADKSPMPEKAETIVLAQGTGPVITDADSVEYYAIVGYWGSSERAHTWEDGIQTVNPGSILVGERVGSRILIITPSADQANPASFTLVDVLAAHPSR
gnl|extdb|pgaptmp_000992			MSEDAINILVYSDDAATRQQVIEGVGLRPAKDLPTVKWHEAATPWGAVDGLKQHSPALLILDGETQKSGGMSVAREIRDRFDDVPPIVILTARPQDAWLAEWAGAAEVVTAPLDPLDLQQRVSTALRSAR
gnl|extdb|pgaptmp_000993	K00766	trpD; anthranilate phosphoribosyltransferase [EC:2.4.2.18]	MNQWSELAGDLVAGAELDYQSAYRLMDQIMAGELGEIRLASLLSLLAMRGPATDELHGLADAMRDNARRIGLPRAAVDIVGTGGDQAHTVNISTMAAIVVAAAGYPVVKHGNRASTSASGSADVLEALGVNLQLDPEQIVDVFDRVGIAFLFANNFHPSMRYAAATRRELGFPTVFNVLGPLTNPARPQASAIGVAKETIAPLVAGVFARRGTSAWVFRGAVRGLDEITTTEPVQVWRTVGNQVQEEVFDPAAEFGLPRAGLSDLRGGSPEHNAKVAREILAGESSPAADAVALNAAAGIVAARGIDSPLTGNAVEQLGSALSQAQEVLSSGAALNLLDRWVAASRG
gnl|extdb|pgaptmp_000994			MVTAIVMINCDINLIPEAAGQIAGIDGVSKVYSVTGDVDLIAVLALPQYDDLAGVVTESIAKVPGVRSMHTHLAFRTYSAEELEQAFHLGLD
gnl|extdb|pgaptmp_000996	K00895	pfp, PFP; diphosphate-dependent phosphofructokinase [EC:2.7.1.90]	MSVRRVALLTAGGFAPCLSAAVGGLIQRYNDLDPTIEVIAYQHGYHGLLTGNFVRVDEEGRAQAGILDKFGGSPIGNSRVKLTNAKDLVKRGLVAEGENPLAVAAEQLRTDGVDVLHTIGGDDTNTTAADLAAYLHEHNYELTVVGLPKTIDNDVIPIRQSLGADTAAEQGSLFAQNIIGEHRSGPRMLIIHEIMGRNCGWLAAETTRKYVKWVDEQEWAPALGLTAERWSPHALYLPEMHIDIDQEAKRLLSVMDELGNVNIFLSEGAGVPEIIAEMEARGEEVARDPFGHVRLDDINPGQWFAKQFAERLGAEKVMVQKSGYFSRSARANAYDLRLIQSMVDLAVECALRGESGVIGHDEERGDQLRAIEFPRIAGGKPFNVNVEWFAPLLARIGQPTQN

gnl|extdb|pgaptmp_000997	K01626	E2.5.1.54, aroF, aroG, aroH; 3-deoxy-7-phosphoheptulonate synthase [EC:2.5.1.54]	MIPWETWRGLPARHQPQYLDAERLRQVSEELRQRPPLVFAGEVDDLKDQMGRAGRGEALVLIGGDCAETFAESTAARLRLKVQTLLQMAVVLTYGSSKPVVKIGRIAGQYAKPRSSPTETHDGVTLPSYLGDAVNGFEFTPEARQHDPGRLLEMYHMSAASLNLIRAFTKGGYADLRRVHEWNKGFTSNPVYARYEDLAEEINRAVRFMAAAGADFNSLREVDLFSAHEALLLDYEAAMTRVDSRTGNLYDTSGHFLWIGERTRAVDEAHVEMLSKVRNPIGVKLGPSTTKEDIKGLMDRLNPDGEPGRLTFMTRMGAQHIRQTLPPLIEAAQADGRPVTWLSDPMHGNTISTAHGYKTRDFDTIMREVAGFFAVHEEMGSVPGGIHVELTGDDVTEVIGGAEGIDDLALLRRYETLVDPRLNHQQSLEIAFQVAELVRERIGTEAPIETEPPALLGEADLALG
gnl|extdb|pgaptmp_000998			MYVDELIGDLLTTQEAADLLGAYRTRVNQYSRENRLVIIQRDDRAYVPAGLLEELPEPVDSATHQPLENLRGTITLLRDSEFSAEEIAEWLWTPDEELGTTPIAALREGRHHQVNRIASAL
gnl|extdb|pgaptmp_000999	K13787	idsA; geranylgeranyl diphosphate synthase, type I [EC:2.5.1.1 2.5.1.10 2.5.1.29]	MTSAAPRLDATIAQISSKIDRTVRRSFLTFATRCSLPEVGLFTSTLLSSAESGKRFRALSSVLGAATWLARTDDAAPEELLLRAATESNLALGAALEFYQAAALVHDDIVDRAEERRGKPATHRAFAATEGSDHFGVSAGILGGDFLLSAAEMALDEAASPTRQPELRQRFHQMTGEVAYGQFLDLQASFAPLGELELIRQVIRLKSARYSVAHPVALGALQAGADPEWAHTLEQVFEPAGVAFQLRDDHLGVLGEPTQTGKPAGGDIVERKRTVLLALTVQHAPSAQRRQLEEIYRQEPGPEQVAQARDLIETYGVGPHEEYLARELDLAHHRLAEAKLPGPAQELCLAFIRLLVERDR
gnl|extdb|pgaptmp_001001			MNQTISRSTLERRELTALDRCDACGARAWVRAVLGETELLFCAHHGTKNLGALAEAADYLQDDRHLLD
gnl|extdb|pgaptmp_001002	K02470	gyrB; DNA gyrase subunit B [EC:5.6.2.2]	MSNSSEYTARHLSVLEGLEAVRKRPGMYIGSTDRRGLMHCVWEIIDNAVDEALEGHCDTIEVTLHPDHSVSVTDNGRGIPVDRVPGIDLSGVEVVFTKLHAGGKFGNGNYAASGGLHGVGASVVNALSARLDVEVDRAGKTYGMSFRRGEPGTFDDAKGRTPNSPFQPFEAAQDTPVIGKAKRGVTGTRVRFWHDFQIFPSSETFSWEGLIDRARQTAFLVPGLTIIVRDLREDEAREETFHFRGGVTDFVDFLAPDRAVCETWKIEGTGTYQEVVQQLGEDGHLRPVEVERTCEVECALRWGIGYDTTVRSFVNIIATPKGGTHLQGFEQGLLRAVRKQVEKQARKLKVTGKDGRVEKDDVLAGLSAVVTVRIAEPQFEGQTKEILGTPAVKNIVLKTVEDAVEQKLTSTKRGDKQEATVVLEKIVGEMKSRVSARIHKEISRRKTALETSTLPAKLADCRSNEVARSELFIVEGDSALGTAKAARSSEFQALFPIRGKILNTQRASTADMLSNAECANIIQVIGAGSGRTFDLSQARYGKVILMTDADVDGAHIRTLLLTLFFRYMRPLVEAGQVYAAVPPLHRIEVSARGKKDYIYTYSEAELHDELTKLKRRGKSYKEPIQRYKGLGEMDAEQLAETTMDPRTRSLRRITLADEEALRQAEDVFELLMGAVVAPRREFIVDHAYEIDRERIDA
gnl|extdb|pgaptmp_001003			MKNDSFPPDEEATLQWRPLTEASLPILHQLVQEVEGASFVPYPTSDREIAFWVQETPRWVGIYGEDQAGQVQAAALVAISGVDQSLCETRSFLQPRLQSKQMWETMMRWQLREAKELLKESGKPKPGLIRTSVHPYQAGLEGVLRSKGFSWVSSTVEMRRLLLDVPEVPNLGPYLGVEPWAEEYEDSARRLFNRLMSGDGTVSRQQWLAHRELFQPEWSFVAVSRQGDRPELAGFLMASAATETGTQEGFIDLLAVSEAAASMDTFQALALASMGRQAAAGVTHTGATVDEPGDPLTTRLYEGLGFTRFYEVKSYSAPC
gnl|extdb|pgaptmp_001004	K02469	gyrA; DNA gyrase subunit A [EC:5.6.2.2]	MTDTPEERISEIDVSEEMRGSYLEYAYSVIYSRALPDARDGMKPVQRRILFQMSQLGLRPDRGHVKSSRVVGEVMGKLHPHGDSAIYDALVRLAQPFNQRVPLVDGHGNFGSLDDGPAAPRYTEARLDRAALLLTDQLDEDTVDFVPNYDNQFQQPDVLPAAFPALLVNGASGIAVGMATTIAPHNPREALAGALHLLAHPDATVDDLMEYIPGPDFPGGGLILDLDAVAESYRTGRGTFKVRAKVVEERISARKMGLVVTELPYMVGPERVIEKIKDAVNAGKLKGISAVTNLTDRHHGLRLVIDIKAGFNPQAVLAGLYKLTPLEETFAVNAVALVNGQPRVLTLKDILQVFVDHRLEVVRRRTRFRLDKRLARQHLVDGLLLALVDIDEVIQIIRASDDATVAQERLMVAFDLSEIQAQYILELRLRRLTKLSRLELEQEQRELADEIAALRAILATEENLREAVAQDLRAAAEILDSPRRTVLSGAEPAVLPPPTDLEIPDLPCQVVLSAAGGLARVAGAAPLAPGGDLPSSVWRSVAATSTRAQVAVVTTDGQAHRLDVVTLPEVARTEDEPVSLAGAIPARELLGVEAVGVFALTDTPVLALGTAQGVVKRVRPEHPESKDTWPVISLDEGDQVIGLGPSADSHDLVFVTARAQLLRTGSTNVRPQGVSAGGMAGIKLAAGDRAIAFASVEPTEEATVLTVAQAAGTLAGTAYTSAKVTSLLQFPPKGRATQGVRCQRLLRGEDSLGLAWIGDHLPHAENASGKPVPLPELDERRDGSGTALKRAFDAVG
gnl|extdb|pgaptmp_001005			MHRPTSPWLGEVLPAVAAALGHPLEESSITLPAARSAIVIVVDGLGWHQLQAHRGHARTLSSFQGPVLTTCLPSTTAAALTCFSTGRLPGETRMVGYSVRHGDSVMNLLQFAPGVDPLTWQPQETYFSRLEGVEPFVVTAPKFAGSGLTQAAFRGATFVGRRSLAERFEAAARLARERPSLTYLYWSEIDHAGHRYGPGSPQWVEELEDFDAELSQFLRRLPADTLVVLTADHGMVQVHERLDLAEIPALAEGVELLAGEGRAVHVHAEDGAAVRARWADYLGDRARILAPAEYPGVFGDGPGNELMGEAVVFLAGNRVIVDSRTQSAGMVGLEGVHGSFTEEELEIPLLILS

gnl|extdb|pgaptmp_001006			MHAPIEHTEGDRGRWAGIFGTMGEDEGGQMIELEFLGTSADGGSVVFTDPEGERYCVAISDELRAAVRRDQTAPETRPLNRGQLRPAEIQALLRQGMTPEEIAERYGVAPASISRYESPVLAEKAWAISQAKQCAVGPDLTVEELVINRLATRGVDHESLTWSALRHPGDDWEISVTFIQSAVLRVATWRLSADGNRVDAIDQEAHWLTESVSPTEPVRALFGVQPSEPEPVREDQQLEAEVLVDQLNQRRGRRQPILDPVELEEEPTSSLPYFSTRVSTPESEEEAETLFTPPPPPTAPAAPKKKKQRRSVPSWDEIVFGARPD
gnl|extdb|pgaptmp_001007			MATDYDAPRKNEDDISEDSIEELQSRRADIGSNEVDEDENEAAESFELPGADLSKEELTVMVVPPQEDEFTCSQCFLVHHSSQLAYTDENGLPVCTECAA
gnl|extdb|pgaptmp_001008			MALFKRKADIPEPVAVEETPEEVLVGPKDSDGQGPPKGYVDLGSLYVPPVPGMQVRAQFEADGKTLHRIQLILGTSGMRVFVAAAPRSGGAWPELREQLAASVEKQGGTAEEVAGRYGTELHAKLPVRLANGEEGQTPVRFLGLEGPRWIVRVDIQGAAATGDEAQEKLCHFVIDNLIVNRGNEPRVRLSLLPLTIPKETVQV
gnl|extdb|pgaptmp_001010			MSQKWSDTLGTDSFSVTDAIGGPRGVIESLAPGLIFVVIFVLTGNLWWTVGSSAGLSVLFCAVRLGQRQPLTQALAGLIGVVIGVVWAVSSGRAENYFAWGLLTNAGYAAALLLSIVVRQPLGNWALTFLWDLPRSWMREGRGTGLYRRAVAVTWVWVAVFALRLGVQLPLYWAGAVAPLGVAKLVMGLPLFGLAAWFTWVLLRGRKPASVSQSEADSAPPAPLSSEQTEPPAH
gnl|extdb|pgaptmp_001011	K03499	trkA, ktrA, ktrC; trk/ktr system potassium uptake protein	MRIAVVGAGSVGIAIAREFIAHGHDVTLIDSSPDAIHISDVPQADWALADACSPAALEDAAVRECDSLVAATGDDKVNLVVSLLAKTEFAVPKVVARINDPGNEWMFNSNWGVDIPASTPRVMAALVEESISTGRAVQLLSLSNQTVLCFALTVPDNSVQIEVNPAEIDWPQELVITTLIRGGKPLDVSRVSGIRPGDELLITCARQDAPLVSELEALFAPS
gnl|extdb|pgaptmp_001012	K03499	trkA, ktrA, ktrC; trk/ktr system potassium uptake protein	MHFVIMGCGRVGARAATELDSAGHSVAIIDRRTVAFERLSDDFSGQRVTGNGLHRAVLERAGIAEAYAFAALTNGDNSNIIAARTVHQIYQVQQVVARIYDPDRAELYERMGIPTVASVKRTTAAVLKRLLPASATIAWDDPTGAVSLLRVRPSAAWIGVPFSQIEQRGGCRVAFVSRLAGIRVAEPKMVVQEHDELVIAQSGTEPGALRKLLSHAPEGELR
gnl|extdb|pgaptmp_001013			MTPPTVSDLPPIELRLGAPAHGGACVAHDQEGRLYFVRGGAPGELVRVRITSPQSRLSWAEVTEVIEPSADRISTTSVPGADLAFLSAPAQREWKAQVLRDQFQRVGSRELARAAEELGTLVVRPAPGDESDGWGRRTRARFRVAKNGHLSISGYRSHQLESVREFPLLDPVFAEAGVFTSPRWEARWRKGETVNLVAPTDSAPLVLAGKRCLDLKGKTAAPVGYWRVEAGGQSVRFQVRAAGFWQTHREAATVLAQAVLRGAGELTGKNVLELYSGAGLFTYFLAERAARVTTVESDRRAVEDAAHNLTQLDGGATTQLNVGKVDAEILNQAGFRPDLLVLDPPREGANRDLLQAVEADRIVLVSCDPAAAARDLALLVGRGYELAQLEAWDLFPNTHHVET
gnl|extdb|pgaptmp_001194			MSKEVASYGSWISPVTAQSYAGRSVTLTQLRMDGPDIYWVEGSPRREGRKVLLRRNALGQTGEVLPLLEGSRLVHVATRVHEFGGRAYAIKNGRIVVSNGTDDRVYYFDTRTLRAQLVPLTQLDKCRYGDFEIDEARGVVYAVCEDHTDPDHVQHSLVAIPLDGSGARDTSKIRTICDQTDFVSSPTLSPDGSKLAWVTWNRPELPWTKSELRVAAMNAAGEPFKQVTLVDRPDVAVTEPRWTFAGDLIHIDDSSGHANLYRTEGFTTNDGEPQDAWATRLRTRVLHPGPRSFTTPRWHLGLHSFDVLDNDHLICSWAEDSQWHLGTIQLSNGLMEEWDTGWWPIGNVAASEGRVVFVGDSSTHYPAIVRVDNAAVKVVRTSNEAEIDQDYNSRAEHVTWTARDGLKVHGFYYPPNNPQFTGPEDELPPLITMVHSGPANAALPGLSLAKQYWTTRGFAVLDVNYRGSTALGRKYRESLNGQFGVAEVADIVDGVNWLAEQKLIDSKRVAIAGSGTGGFTVMAALESTEVFSAGTSRYGYLDLRRTLTHAPIVDADYLRRLMDSSDSSDPVWAERSPINQLARVNAPVLLMGGANDPIVTPDEIEEVYQKLVDSGKEVAKVIFDNEGHGFVRADTLESAWRTELAFYADIWGIELQHPVPVEIANRGSRPVVNPV
gnl|extdb|pgaptmp_001193	K01662	dxs; 1-deoxy-D-xylulose-5-phosphate synthase [EC:2.2.1.7]	MRSLLDRIQTPRDLDSLTAAELVSLAEQIRAYLIESVAKTGGHLGPNLGVVELTLALHRVFDSPRDTIIWDTGHQAYVHKLLTGRKDFTQLRQPGGLSGYPSRAESAHDVVENSHASTAISWADGVSRERRRRGQAGSVVAVIGDGALTGGMAWEALNDMAEDQDRSLVVVVNDNGRSYEPTVGGMARHLAGLRTSEHYERSLAWGKRKLLSLGEPGRAAFEALHGLKAGIKDVLAPQVLFEELGLKYLGPIDGHDLIAVEYNLELAKSFGGPVIVHVITEKGRGYVPAEEDVSDRFHAVGKIHPETGLPVAPQRFGWTAVFAQEIVELAERNPDVVGITAAMKAPVGLQPLANRFPDRVVDVGIAEQHALTSAAGMAFAGAHPVVALYATFLNRAFDQLLMDVALHRAPVTVVLDRAGITGDDGPSHNGMWDLSLAAMVPGLRVAVPRDGATLRAELDEAVGDSSGPTLLRYPKGALPPDLAVAETSAAGDYLYRGREADVLLVALGPLAHYAVEAAQELKSRGIEADVLDPRWVLPVAPELVEVANSYRVVVSAEDGLEVGGVGSELARQTQVPVLVRGVPREFQAQGKREEILARLGLDSAGL

gnl|extdb|pgaptmp_001192	K00656	E2.3.1.54, pflD; formate C-acetyltransferase [EC:2.3.1.54]	MATITTAWTGFKTGRWEKTIDVRDFVQENYTPYEGDDSFLAGPTDRTQRVWETLCGLFPAERERGVFDIDNHVPGRITSHEPGYISADDNLIVGLQTDAPLKRAMIPNGGWRMVENALLTYGYEVDPIIKEIFTVYRKTHNQGVFDVYPKSVRAARSSHIVTGLPDAYGRGRIIGDYRRVALYGVDRLIEAKQADKAELDDRFSTDDVIQLREELAEQIRALQELKEMATSYGYDISGPATSAREAVQWLYFAYLAAVKEQNGAAMSLGRTSTFLDIYLQRDLEDGRLTEVEAQELMDDFVIKLRIVRFLRTPEYDALFSGDPTWVTETIGGNGCDGRTLVTKNSFRMLQTLYNLGPAPEPNLTVLWSEQLPEGFKRFCARASIETSAIQYESDSLIRESWGDDAGIACCVSAMAIGKQMQFFGARVNLAKALLYAINGGRDEISGRQVAPVSEPVGDGALDFEDVRTKFDGLLDWLARTYVDALNCIHYMHDKYSYERIEMALHDTEILRTMACGIAGLSVAADSLSAIKYAQVTPVRDETGLVVDYLIEGDFPKFGNDDDRVDAIAVGLVESFMNKIRALPTYRNSQHTQSVLTITSNVVYGKHTGNTPDGRRQGEPFAPGANPMNGRDTHGMLASALSVAKLPYDQAQDGISLTNTVVPSGLGRTLDEQEVNLVGLLDSYMGEQGYHMNVNVLNRDTLYDAMENPQNYP
gnl|extdb|pgaptmp_001191	K04069	pflA, pflC, pflE; pyruvate formate lyase activating enzyme [EC:1.97.1.4]	MSTTDLRVAGDGVTGEYRPAPASVPSARPQGQGLTGVESELPDSRRDLVNLVRTGSVASVHSWELVTAVDGPGTRMTVFFSGCPLRCLYCHNPDTMEMRRGEQVQTASLIDRMLRYRRIFARTGGGITLSGGEPLMQPKAVARILRAAKTAGIHTAIDTSGNLGWRCTDEMLADLDLCLLDLKSGHRSTYQQVTGARLEPTVAFGQRLARAGVEVWVRFVLVPGVTDDLENIAEVARLAAQISTCTRVEVLPFHQMGRDKWDELGMEYQLAEVEPPSAAAVESARQIFRQHGLTTY
gnl|extdb|pgaptmp_001190	K03684	rnd; ribonuclease D [EC:3.1.13.5]	MPELPVLTTLADGVPEVIDTQQGLSEAIEQLATSRASVAVDTERAQGFRYGAEAWLVQIRRADVGTFLIDSHALPKLAELNAVLDAPWIFHAAGQDLPSLAELDLVPPALFDTEVAARLTGVQHFSLQGVCEEVLGFSLEKAHQNENWSVRPLPLPWLRYAAMDVEVLPQLETALTERLEELGRLEWARQEFDYELTHPIKSKAPRWENLKGIGKLRRPQELAIARELWTTREEIAVRTDVSPGRILNGRGILEAALRDPQNRRSLQQIEFFRRPQARRYLDEWWEAIRRAHSLLPEEMPHGLPAGEGIPPARLWKRLRPEAQDRLQQVRALVERAAKPLDLAPEVVLLPATQRLLAWEPLDDLEQRLIEGEARPWQRELILAASKS
gnl|extdb|pgaptmp_001189			MSNTPPQFVAALASLRRVARPDSLTFREIPAPQRLAPYSAAVAIQTVQEHGGQPLGQSTFVILYDPEQAEIWGSPLRLVGQLRTQIDEEMSSDPLLGEVLWLGLRDELGEAAELLLGTVTREISQTFGGLELRSSTLNIELRCSWSPVQDDLAPHLEAWGNYLLQVAAIPSENYLGFEVHDA
gnl|extdb|pgaptmp_001183	K17810	asl; D-aspartate ligase [EC:6.3.1.12]	MELVPVILGGDVGAYTLGLECYEAFGVKSICVSAAPIDMITKSVIFEVEQVTPQASDEELLAVLRGIAAIHQNLLLLANTDAHVSFFARHREELSQHYLLPFPTTETIDLLCQKQSFAEVCAREGVPTPATVVVDLAEEADPEIDFTFPVVAKTASGAAYDRVSFPGKKKIWFIDSPEELTELWSTLRGAGFRDKFVVQENIPGDDTYMRSLTYYVSSTGEILLRAGAHVLLQDPSPTMIGNPVAMITRELPDLWELGDRILRAGNYTGFANFDIKIDPRDGTPYFLEVNPRIGRNSYYVVAAGQNPMAVMAQDLFLGESPEPVVAQETALYSLVPFKLILQTVKDPDLLAEVKTLPVVDPLDSPIETSKSRKAVARLQKLNYLRKFRRYAR
gnl|extdb|pgaptmp_001509			MARAVTLEEVGARAGVSRSTVSRVINGSPDASPKAVAAVMEAVTELGYVPNRSAQALASRKAAVVTALIPEDMERFFGDPFFGAIISGIEDGIRSTSLVLNLVVTSEASSDKILSFLVGGQSDGILVLSHHTSHRLIEAVERRIPVVYGGKPIGNPEEKNYVDVDNCEGGRMAARHLLAKGRRQLAMIAGPSDMPSAQERRAGFLELAGSAVIEAGDYSAASGAEAARRLLDAGGPVDGVFVANDLMARAAVDVFQASGRRVPEDIAVVGFDDSVAATVARPHLTTIRQDPFAQGQTMVQLLLSRLEGNEPARSVLLPLTLVERESA
gnl|extdb|pgaptmp_001510	K05350	bglB; beta-glucosidase [EC:3.2.1.21]	MNSQQIPSNFVLGAATAAFQIEGGADRGPSIWDTFCRVPGAVLDGDDGQVACDHYHRYREDVALMKQMGLDSYRFSTSWARIFPDGRHLNQAGLDFYSRLVDELLEAEIKPWLTLYHWDLPQALEDEGGWPSRDTAERFRDYALTVHRALGDRVDTWTTLNEPWCSSFLSYTAGAHAPGHQDLGEGMLASHHLLLGHGWATQALREADSNLELGLTLNLTPVQPLTSADKAVAEIVDGQANRWFLDPLFRGRYPADIVQEYRRVDEAAADRFTAAVRPGDLEAISTPIDVLGINYYQGDIVTAQPQLPAELAEKFGEFPLPPRGDAPVSRPVSAPTPSATPIYRPDTSLPRTAMGWQVDPLLLTELLQRVHEEYTGPAGVHLYVTENGAAFTDVVTDGQVHDQDRANYLQWHLGAVLDAQRLGVDVRGYFYWSFLDNFEWAWGYSQRFGIVYVNYETQERIVKDSGRLYAEIIATRKLDVEPNTGRVIRRGTLVE
gnl|extdb|pgaptmp_001107	K01426	E3.5.1.4, amiE; amidase [EC:3.5.1.4]	MLHELSAWEQVELLVSRQISPGELTAHYQKRIADHPEVGAFVTLTPQVAQRQLAELAEPGSTPLWGLPHADKDLVHRQGVPTLYGSLAVAAATRQYPAQLQAPSEPLIEQCDRLGLISLGKTNTPEFGLFGYTESEVAAPARHPEDLTLNAGGSSGGAAAAVAAGLLPAAIGSDGGGSVRIPAATVGLVGLKPGRGVVATDRGVDSPTGVVTGPLTRSVRDAALFFAALTEGDRTLAERELAAEAAPLRIAFAEDSPWNPTYDCHPEPVVLEALEQGLSLIGAAPDRVSLAHRRYADIFTTAWFRAAGSVPPLLDIELMHPVSRWLIESGRQLSPEQVEQNRQEVAAYGAEMSALLADWDVIVTPALGMAPQPVGSYPLDPQQNFALQVAYSPYTSWVNLVGWPALTVPVTRLPAGHLRQPLPFGIQLVGKPGSEWDLLRLGAALEARVK

gnl|extdb|pgaptm p_000401			MLIRRVDAEELRQAVSGAAFVPLEQATPWRKFSEANGHYLWGHLLWEEDGKVLAAATFYRYEVRGQKYLWTRNGPLWLKAPAPDREEEALDLLRTHLRQ HAKPYSFVRLHAWYEHPALHRPFRVIGYDRTVLIDGARGNREQAFQLLPTAGRRLIKKARRRLEEHNGTIVEATGLSREEFTEYYRIMEETGSRDGFTPHEFDY YWAMLQKLGPEHARLFALKLDGKLAGWDLVGVYGKRATAFYGATNAAARSSQTAPLLDFEVACLLGEEGIEGLDLMGIHSPRTPQLYDVGRYKLQFAQHY VDVPGLWDLPLSEPMYRTLELAYRTRTVYRRLTGRVRGQRGDD
gnl|extdb|pgaptm p_001182	K01868	TARS, thrS; threonyl-tRNA synthetase [EC:6.1.1.3]	MTTLTIDGEQQQVEPGTTGTDWYGKRREVVAMTVDGQAQDLFRELPAGATVTAIELSSPEGLQILRHSAAHVLAQAVQEANPQVDLGIGPPIENGFYYDFG VDTPFTPEDLRALEKAMARIVKEGQTFQRRVVTEEEARVELADQPYKLELISTKSGSNAEDGSSVEVGGGELTIYDNVRRGGEVAWKDLCRGPHLPNTKLIG NGFALTRSAAAYWRGSEKNPQLQRIYGTAWASKEELRDYQNRLAEAERRDHRRLGNELDLFSFPDEIGSGLAVFHPKGGITRMEMEEYSRRQHIAAGYDFV YSPHITKGDLFERSGHLGWYREGMFPPLKVDAEVDPETGAVTKPGVDYYLKPMNCPMHSLIFSSRGRSYRDLPLRLFEFGTVYRYEKSGVVHGLTRARGFTQ DDAHIYTTRQQMREEITTVLRFVLDLLRDYGLNDFYLEMSTKDPEKYVGTDEVWAEAETTLRSVAEESGLELVEDPGGAAFYGPKISVQARDAIGRTWQMST IQLDFNTPERLDLEYQAPEGTRERPVMIHRALFGSIERFFAILLEHYAGAFPAWLAPVQVRCVPVADAFAPYLDEVAAELRRRGVRAEVDRSDDRFGKKIRNA SKEKIPFVLIAGGEDAEHGAVSFRFRDGSQENGVPKAEAIERIAAHIAARKNTDEL
gnl|extdb|pgaptm p_001181	K19710	E2.7.7.53; ATP adenylyltransferase [EC:2.7.7.53]	METADEFAGEADGYERLWTPHRMVYINSDHGDHECPFCRAPKRSDEEGLVVYRGQTCFVVMNLFPYNPGHLLVCPYRHVAGYVDLTEEERREFGELTASA MRVAEAALGAHGFNTGMNQGAVAGAGVAAHLHQHVVPRWQGDANFFPIVAQTKAVPALIENTRQQLVAAWKEAADAR
gnl|extdb|pgaptm p_001180	K00995	pgsA, PGS1; CDP-diacylglycerol---glycerol-3-phosphate 3-phosphatidyltransferase [EC:2.7.8.5]	MLGNHGRSITLAIFTPLAIGLEKLRITPNMVTVAGTVISCTLAVTLLASGHLAVGGLALGVVLFMDSVDGVLARRTGTSSNFGAFLDSTMDRITDGFVFGSLLY WAVVGLPDGAVRQVSIVAGIICMTAIGVVPYVRAKAESFNVVAKVGIAERTDRLIVALLGGALTQWGLADWWFAVGLVWVAFASCVTVGQRIWVVHREL RSA
gnl|extdb|pgaptm p_001179	K22311	ptfP1; phosphatidylinositol dimannoside acyltransferase [EC:2.3.1.265]	MNLLYRLAWSVVPHLPPRLVDAVAGWVGALVGSLNIASVRQLRRNYHQLTGRDPSRSELRRAVASYFRCFAQQFSLPGWSDQYLRSGCIYPGAARVAELM TEGPVVLALTHSGNWDLAGAWFCQNHGPIVTVAEKLEPAELFDQYVNFRSNLGMEILGVAPGEKVFERLVETVAGRSVLVPLLADRDISGSGIRVQLGGGE ALVAAGPAALALRLKRPLIAGHITYERRGRRWVIRAEFTEPIPVPTPEPGETDVEALTRAWVRTIEPTLLRGLVDWHMMQKLYVADLDPARLARAEARHRGE TP
gnl|extdb|pgaptm p_001178	K08256	pimA; phosphatidyl-myo-inositol alpha-mannosyltransferase [EC:2.4.1.345]	MKIGIVCPYSFDVPGGVQFHIRDLTEELIRRGHLVSVLAPSEDPNPPAWLASTGGAMAIKFNGSVARLAFGPVVACRTRKWLEEGQFDVIHIHEPETPSLGLL ALMNADVPVVATFHAALDRSVVRQLTSGVLAPFLEKISARIAVSQEARRTLIEHHAGDAVIIPNGVYTASFREAEPDPRWVGTDERPVIVFLGRLDEPRKGLP VFAGAIEPVLAEFPGARFLVAGRGTADVLPKLPAVEVLGEITDAEKESLLKGATIYVAPQLGGESFGIVLVEAMAAGTTVVASDIAAFSAVLEEGEAGILFPTGS SEGLADALLARLRSADDRVARAGQRAAEKYDWSTVTEKILAVYQAVLPPSTQPQTGTAYELINERLGRAER
gnl|extdb|pgaptm p_001177			MWWILLAAVLLALVAYIWTLAVRIDRLHRRVATARGGLELALVKRAACSMQLATSGLLPRRDAEALTEAANGSLAAIEDTWRPPRFLAESALSRALRQVCTP EQSELICSTDLGRALWNELAEARYRLQISRTLYNQDVSLVQELRARRLVRFLHLAGFAPLPSYVDLDDAS
gnl|extdb|pgaptm p_001176	K24131	prsW; protease PrsW [EC:3.4.-.-]	MPANPGDRYRLSAQAPLRRAEGLNVGATDSTPAPPRWRVWETVLIVVFVLLFAAVVVIGGGSLATRGQLALVALVPLVLVTALLTWIDRWAPPGWRYRWL ALLWGAGVAASGAILVNSSLYQDLLLYRGSPEWAGTFAAVIIAPISEELFKGLGVVTVLVLARRQLTSTLSAVALAGLVGAGFAYVENLDYFWQAYQEGSTVF GFTVFARSVMSPFIHPMATSLTGLAVGSALLAGKGWFWRLPLGFVGAVAVHALWNGMAMLGAIWVLVYLVVELPLFIAWLTWLLVWSQRMARQIHLGL VPYVLTGWISPAEVALTVTPSGRRAARRWARKLKGRRPLRRLQRAVGRLGMDQQVMSRRGPNLDKIEQDRQHLAEVARLRAELKQLELAQ
gnl|extdb|pgaptm p_001175			MTKLGADWQVGPDGIPTRRAARVVLLSTDGKTLLFEGHDGDDPAHRWWFTCGGGIEPGESPRAAAARELAEETGLVVESARLVGPVLERDADFAFRNVL ARQIEDYYLVFLDGPARAIDTGGQTGSERNLVDRWRWFSAEQLVELAERETVYPLGLARYLRRWERGWDGVKLRVTERNSSHPPPN

gnl|extdb|pgaptmp_001174			MSGHSKWATTKHKKAALDAKRGKLFARLVKNIEVAARTGGGDPAGNPTLYDAIQKAKKNSVPADNIDRAVKRGSGAEAGGAEYQSIMYEGYGPGGVAFLVECLTDNRNRAASDVRLALTRNGGSLADPGSVSYLFERKGIVEVPAEGTNEETILEAVLDAGAEEVEAHGDVFVVTSGPSDVISVRTALQEAGIDYNSAEVEFQASTEVEMDVEGARKVMRLIDALEDSDDVQNVFSNMTLSEQTQAALEEE
gnl|extdb|pgaptmp_001173	K01159	ruvC; crossover junction endodeoxyribonuclease RuvC [EC:3.1.21.10]	MRVLGIDPGLTRCGLGVVEVDDSRRARLVSVGVARSSPDLATHFRLRDIAAAIREALEQYRPEVVAIERVFAHDNLQSVTTTMQVMGVAMSCVGEAGLPLAVHTPSEVKAAITGNGTADKAQVQHMVARILGLKSPPRPADAADALAIAICHAWRGNGLLGAGADGDIHVTLSGGVSARKTMTPAQAQWAEAVAASRRKGAVDPRRR
gnl|extdb|pgaptmp_001172	K03550	ruvA; holliday junction DNA helicase RuvA	MISTVRGSLLQTGLDWVVVEVGGLGLRVLTAPATVSKLPPRGQEVFLETYLVVRDDALTLYGFASAAEREMFEVLLGVSGIGPRTALGALAVLTPAELAGAIEQADLAVLQRVPGVGRKSAQRMVLELGGKLPSGIGASPDSQLRTEVAAALEQLGWGKPQVDKTLAGLDGDYPDASAMLRAALLVLGSHRG
gnl|extdb|pgaptmp_001170	K03210	yajC; preprotein translocase subunit YajC	MIIMLGLMVVAFIALSSFSKRSAQKRQVEHERMLAEQLVPGAWVHTSVGFFGRFVDLDGNVVILETPSGEETYWDKRVIRSVGELPFETEETEIEEVEEYSDEEGHEEPNTDTDTNEDRI
gnl|extdb|pgaptmp_001169	K03072	secD; preprotein translocase subunit SecD	MAEQSKSPWRALVSLIVVAVVGIGALIGAHFTQGAGFVPKLALDLEGGTQVILTPRLIEGSETTGTIDQDAMNEAINIIRQRIDASGVAEAEIATLGQDNIVVSIPGKADDQTLDLIRTSATMRLRPVLQIGAPGAIDPSLNSQSGAQSGDSATAGLTPEEYAFMVADQNGDGKLSTEPLTTPENASDPAWITEQVLYDFLLLDCTDPASLLSNSADDPALPLAACSTDGTGKYLLGPTEIEGQHVSSASSGMIYSQTGQPTGQIGVNIQFDSEGSKVFKDVSTRLYGLRDTDPVRNRFAFALDGNVITAPGMNAIIPDGGAQITGNFTVDSAKTLANQLSFGSLPLNFEVQSEQEISATLGSHHLAMGILAGIIGLILVVLWMIWQYRGLAIVSAGSLVVAAFLTYLAITILSWAIGYRLSLPGVTGLIIAVGITADSFIVYFERIRDEVRDGIPLSGAVERGWRRARRTIVISDMVNLVAATVLYVLAVGGVQGFAFTLGLTTIVDLVVIFLFTHPLMALLMRTRFFGEGHRWSGLDPEHLGAKSGTVYSGRGRVRQLEGEPTVDSGEQTIAERRRQARLAAKVEEESAE
gnl|extdb|pgaptmp_001168	K03074	secF; preprotein translocase subunit SecF	MMSYSQWGNELYSGKRSYRIVQRHRVFLGISLAAMLISVLLLGFRGLNPSIEFTGGTQFIVTQSAEPNPQTAEKVLKDAGLTENIRVTNLGNSGVRVQTPSLESAQMQDIRTQLADVYAVPLEDVQTNSVGATWGQDVTKRALQSIVIFVVLVAILMTIYFRSWAMSAAAMLALVNDLVVTVGFFSLTQVEVSPATVIGFLTILGYSLYDTVVVFDKVREVTKNYQDQERYTFAELVNLGVNQTLVRSINTSIVALLPVASILFIGSFALGAGTLTDISLALFVGLIVGAASSIFIASPALSVMEGFRAKSKEHTQRMLERRAARAEDPAAAVAAPTVAPKRPGRRLENAAQPKRKKRR
gnl|extdb|pgaptmp_001167	K00759	APRT, apt; adenine phosphoribosyltransferase [EC:2.4.2.7]	MTENLGVEIAELVEQNLRLVPNFPLEGVLFRDVTPLFASGPAFQELINLLADRYRGKIDMVAGLESRGFILAAPLATALGVGMVAVRKAGKLPGPVVGIDYDLEYGSARLEVQPESVPDGARILILDDVLATGGTAKASVDLLEMCGAHVVEIAVLLELVDLNGQVKIAGTPFASAVEVHES
gnl|extdb|pgaptmp_001166	K01139	spoT; GTP diphosphokinase / guanosine-3',5'-bis(diphosphate) 3'-diphosphatase [EC:2.7.6.5 3.1.7.2]	MSDSNNHERPAASGSFVRSGLAWFGSRSRDIAPAIEPLIRAVKAHHPRADTAVIERAYEVAERQHRGQTRKSGEPYITHPVAVATILAELGMTPPTLVAALLHDTVEDTDYTVEQLTADFGPEVAQMVDGVTKLDKIRYGAAAQSETLRKMLVAMSRDIRVLLIKLGDRLHNARTWKYVAPESAAKKAQETLEIYAPLAHRLGMNTIKWELEELSFKALYPEVYDQIDRLVAERAPQRDSYLSRVIAEIEEDLRRSRIRGTVSGRPKHHYSIYQKMVVRGKAFDEIYDLVAVRVLVESIKDCYAVLGAVHARWNPLPGRFKDYIAMPKFNLYQSLHTTVVGPEGRPVEVQIRTFEMHDRAEFGVAAHWRYKQDPSAASSDGDKMSAKEQMNWLKALVEMERETGDPEEFLDSLRFEIAGDEVYVFTPRGEVVVLPPKATPVDFAYAVHTEVGHHTVGARVNGKLVALDTVLESGDTVEAITTQAPNAGPSRGWLEFVSSPRARSKIKNWFSRERREESIELGKSHMAKSIRKQNLPLQRLMTHDSLNSVATAMGYQDVSGLYAAVGENHVSAQNVVSRLLDMLGGDAGTEETLAEAIIPRTVPPRSAGSSSDAAITVEGMNPNDVWVKLARCCMPVPGDDIVGFITRGEGVSVHRADCVNAVHLKETHPERFVGVEWSSDQSGSQYLVEVEIKALNRAGLLNDLSQVFSDHQIDILSGTMSTSGDQVATARFTFELPSVGYLDSVLSGLRRVEGVFDAARRLGSVKNSRS
gnl|extdb|pgaptmp_001165			MSSTTSNSGPHSFGRVDADGNVWVRDGNNERQVGSYPDGAPEDPLALYVRRFLDLEATVNLFETRLPGLSARDIDSTLQVLREQLVEPAVVGDIDSLRQRVEKLAEVAEARKAEIAVERAQAKEQALAKRTAIVEEAERIAGQDVDKTQWKQSGQKLRELLDEWKEAQRHGPRLDKTTEDGLWKRFSSARTVFDRNRRQFFAALDASQNEAKKLKEALIAEAEAIQLSEDWGPTSMAYRDLMDRWRRAGRAPRKIDDALWARFRAAQQVFFDRRRAHDQENDQRYAADLKVKEALVEQAEALLPIGDLEEAKQKLRAIQDQWEESGRLPNREGAKVEGRLRAVEEAVRQAEEAEWRRSNPETQARAEGMLSQLEDSIAELERELAAAQADGYADQVRDIT

gnl|extdb|pgaptmp_001164	K01069	gloB, gloC, HAGH; hydroxyacylglutathione hydrolase [EC:3.1.2.6]	MAKLQIETLPTDLFDANCYLLFEDGSDQVVVVDPGLGAFLQVNQRLSELGKRVGAVLLTHGHPDHVWEAAQVAGLGGENTPTYIPGPDRDWLRDPLGQLGFDQLTELPTEWVEPERVEDAPTGSWEILPHIYLNLLPAPGHSRGSAVVLIGGQVVFDGQELQAPTAFSADVIFAGSVGRTDLPGGDETEMRESLRTLANALDPHTVLLPGHGPKTTWGQELESNPYVRRAAGLKH
gnl|extdb|pgaptmp_001940	K01892	HARS, hisS; histidyl-tRNA synthetase [EC:6.1.1.21]	MANPVVPARGMRDIMPAEKKKRDRVLGIIRDVYRQAGFDEIETPAVEPLTRLLSNQGGENEKMIFEIMRRGLPADEPVIARDASDLGLRYDLTVPLTRFYATHAAELPRVFRALQTGPVWRAERPQKGRFRQFRQCDIDIIGDEHITAEVDLLVTTLAAFSALGMAEDIRVLLGDRRFLVDLLTAVGVESEHHDAALIALDKSDKIGADGVRAELLERGLCTPDQADELVSLNIDAEAALAAGEVSLPSGKTVSLYDLPAIVAAVRALAPTCQIEFDPTLVRGMGYYTGPIFEVAHRYRNFSVAGGGRYDHVVGKWLGREVPACGFSIGFERIIDLVDLPEEQEDRVALLYKPGSDPVPVYRLRAELQASGHAVGLVVPPRRLGGQFFESLAADGYSHFVDSRREDATLDDL
gnl|extdb|pgaptmp_001163	K05592	deaD, cshA; ATP-dependent RNA helicase DeaD [EC:3.6.4.13]	MPHASLPVDPEQSEITTTFSDLGLGQQTLSILDRLGFVTPTPIQTAAIPVLSQGRDVVGIAQTGTGKTAAFGLPLLEAIDPHQREVQALILAPTRELALQTSQALEQFAAGRKIDVVAVYGGAPYGPQLHALRTGAQVVVGTPGRVIDLIEKKGALNLGAVRYFVLDEADEMLRMGFAEDVEQITGALPDSRLTALFSATMPAAIERVAKQHLQDPVRLEVSTAASTVDTIRQTYAVVPPRFRFEALTRVLAVRDGGATIVFVKTRQDAEEVSLDLAAQGFRAAGISGDVAQTDRERLVSRLRSGHLDVLVATDVAARGLDVERIGLVVNYDVPREREAYVHRIGRTGRAGRDGESLTFFGPRDRFQLRQIERLTGERMEEVHVPSRAEVTQVLAQRKLSSLAELPAGPTSDLLQQALNDQLATGVSLHELALRMLAELTGTTSAKASRPTHISEAVVDQDGYFLSAEFTGQGGKRERRSEPVSRGKGHPGRQDVDFQHRYRVEVGRKDGVTPGALVGAIAGEGGLTGADVGHINIFPSFSLVELAEGLSPSQVKRIGRATVRGRRLRISEDSGPTQRKTRFADRSKHKRR
gnl|extdb|pgaptmp_001162	K01876	DARS2, aspS; aspartyl-tRNA synthetase [EC:6.1.1.12]	MLRTDTVGQLGLDRVGDTVTLTGWVDRRRDHGGVVFVDLRDASGVAQVVVRDESVAHELRNEYVLRVTGEVAKRPEGNENPDLPTGYIEVLGDRVEVLNSAAPLPFQVSTHEDAPTAGEEVRLKYRYLDLRRPAAQFPLRLRSKISRAAREVLYSHDFVEIETPTLTQSTPEGARDFLVPARLSPGSWYALPQSPQLFKQLLMVAGMERYFQIARCYRDEDFRADRQPEFTQLDIEMSFVEQDDVMRVAEDVLKAVWAEAGYELETPIARMPYLEAMERFGSDKPDLRFGYELTDLTEYFKDTSFRVFQAPYVGAVVMPGGASQPRRTFDKWQEWAKSRGAKGLAYITVKDDGTLAGPVAKNLSEAEAAGLADKVGANPGDCVFFAAGAPSASRALLGAARLEIAHRCNLIDEDAWAFVWIVDAPLFKPTSEAVDEGDVAVGEGAWTAVHHAFTSPKPEWADNFEQDPGHALASAYDIVCNGNEIGGGSIRIHRRDMQERVFKVMGIDQERAQEQFGFLLGAFQYGAPPHGGIAFGWDRIVSLLAHTESIRDAIAFPKSGGGFDPLTGAPAPITPEQRKEAGVDAKPKKSEPESDK
gnl|extdb|pgaptmp_001160			MRAIELHPDNPQSRLVNQVVDVLNSGGVIALPTDSGYAVACKMGNKAGMDRIREIRRLDEKHNFSLLCHSFAQIGELVIVDNKAFRAIKALTPGPYTFILTGTKEVPRMTLNKKKHTVGVRLPDHNTTQAIVAELGEPLLCSTLILPGQDEPLTDSEQVIEEVGNQVDVVVEGPVGDEGATTVVDFTPGYPEVVRVGAGSTELFE
gnl|extdb|pgaptmp_001159	K07478	ycaJ; putative ATPase	MDLFDALGTDNHGLPDAGPNAPLAVRMRPRSLSEVLGQDHLLEPGSPLRRLLSPASDESTGLSSVVLWGPPGTGKTTLAYLIARESGYRFVELSAVSAGVKDVREVITGAKHRLASSGVPTVLFVDEVHRFSKAQQDSLLPAVENRWVTLVAATTENPSFSVISPLLSRSLLLVLRSLSEEDIRIALTRAVGDERGLQGKFELEDEALAALVRLAGGDARRGLTLLEATAGVAAERGERTLTPEFVSQAANTALVRWDEDQHYDVASAFIKSMRGSDPDATLHYLARMIEAGEDPRFICRRIMIAAAEEVGLADTSVLQTAVAAAQAVAQVGMPESQLILAEAALAVALAPKSNSVTVGIGEALKDVREGKGGPVPLHLRDSHYSGAKSLGHGVEYRYAHDYPHAVASQQYLPDDLVAARYYRPSERGNEAILTKRLAMLREILDMR
gnl|extdb|pgaptmp_001158	K02986	RP-S4, rpsD; small subunit ribosomal protein S4	MAGNRSRKQVRQSRALGLPLTPKAVRYFERRPYGPGEHGRSRRRQESDYAVRLKEKQRLRAQYGIREAQMARTFEEARREKGLTGENLVELLEMRLDALVLRAGFARTTAQARQSVVHRHILVDGKIVDKPSFRVKPGQVIQVKPKSQTTVPFQVAAAGSHRDVLPAVPAYLDVELESLKATLVRRPKRDEVPVTCDVQMVVEYYSR
gnl|extdb|pgaptmp_001157	K01872	AARS, alaS; alanyl-tRNA synthetase [EC:6.1.1.7]	MRTAEIRQRWLDYFAANDHELRPSVSLISPDPSLLFTVAGMVPFIPYIIGEEEAPWPRVASVQKCLRTNDIDNVGHTTRHGTFFQMNGNFSFGDYFKEGAINYAFDLLSGPVDEGKYGLDADRLWVTIWDQDDESYRILTKQIGLDPKHIVRLPREENFWDTGQPGPAGPCAEWHIDRGPEYGPEAVGGTVDPGGDRYLELWNLVFDQYMRGEGAGKDYPLLHELDRKAIDTGAGLERLAFVLQDKPNLFEIDEVRPVITQAEEISGQRYGTNPDHDVRLRVVADHVRSSMMLINDGVRPGNDGAGYVLRRLIRRAVRSMRLLGVDEPTLPALLPVSRDAMAPSYPDLLTNWETISEVAFGEEESFRRTLDAGTTILDLAVNQALAEKTAEAPATLSGEQAFALHDTYGFPIDLTLEMAAEKGVQVDENSFRALMNEQKQRARADNLAKKSGHLDSSVYHRIAQTIGGGSQFTGYDHSVNEATVAGILVAGEAVPAATGEQEVQVILDRTPFYAESGGQLADHGEIAFPNGAVIEVTDVQAPIKGLFVHTGQLRGGDIALGDQAEARIDTDRRLAIARAHTATHMVHQALHEVVSKQATQAGSENSPSRMRFDFRHGKALTGTQMSDLESLVNEKLAGDLEVWDRTMALEDARALGAMALFGEKYGKEVRVVTIGDGWSKELCVGTHVPATGHIGRIAVLGESSIGSGVRRIDALVGDGAYQFQAVEHALVSQLSNLLKGRPEELPEKVSGLLDRLKDREKEISQLRTELLSGQIASLAQEARYLGDFKVAAGILGDVGAADAVRSAALELRTQLGEDSPTIVVAIGEAAGKPLVVVATNQSARERGAKAGQLVRVGAQALGGGGGGKDDIAQGGGAEVSAMPTALDRILSEIGSL

gnl|extdb|pgaptmp_001155	K07082	mltG; peptidoglycan lytic transglycosylase G [EC:4.2.2.29]	MSDSNNGVQPPNRLAPPFPSRKELHAARIPQRPAGEDAGAPEAQLPEAPVEPTSTPPTRREPPRRPRTVQPEEVQVETPVVSEAEPADWAPVAEPEAPAAPESPSDESGPSFPRVTESGPTFESPFPKRVSPATGAQPILPAAGPRQTEVATKKKKSRQLRRRLWTALIAIVILAAIGVGLWWALGSLQGGPGLRAADDYPAVDLTGPEAANHPTVEVTVEPGALGSEIGQALVDADVVKSVAAFTRAFDANPAAASIKPGTYTLHTQIPAVDALAALLDETNRKENTITVNPGQTVSQIAEKLETVAGFSADEVNAVVDDPSGVTDLPAAAKGNLEGWLWPGSYDFSPTSTPTEVFSEMVKGTVDYLKSEGVPEDKWQETLIKASIVEREVNREEDMPKVARVIENRIADPEGPTRGMLQMDSTVAYGVGGTGGLPDSAAFDDDNPYNTYKVKGLPAGPIASPSAAAIQAVLHPAEGDWLYFVTVNLTTGETVFTDSHDELQTLT
gnl|extdb|pgaptmp_001154			MTRWAAVIGSPIAHSLSPVLHRTAYRLAGLDWEYRQFEVTESSFPEFAESLDGDCAGISVTMPCKQVALSFADVSDGLAKTVGAANTLVPAAGLWGAFNTDVHGIVESVRAPFGDTLEEFAAQNRAARKQAVIIGTRATASSALAAAMTLGFDQVSVVGRSFQGAGNVTLAAGRLGVSFTPIKWDRTDLVREVLSRADLVLSTVPAEASAQLVEFAAPRPDQILLDVTYAAHSELTQTFVDAGALVIDPLAMLVYQGLAQVKLMSGHEVAFEPVYREVVAASK
gnl|extdb|pgaptmp_001153			MLWLNVCWCVLRAPHWHRRYSPGLPPATWLAIPIFATTLAFLVPVPVPGVMVQLGLVSALVDGVSRRLPLELSLLLAVELVLTSKVDPLGALWWAVPLAVGARWGQVGRGDVILAAVLGLESGPAGLVVALTAALLFVAVTRSRQTLAFGPFLLLGAVCGAGW
gnl|extdb|pgaptmp_001152	K01736	aroC; chorismate synthase [EC:4.2.3.5]	MLTWTTAGESHGPALVALIEGMLAGVPLTTQTIADELARRRLGYGRGARQKFEQDEVRILSGVRHGRTTGAPIAIEIGNSEWPKWETVMSADPVPPESLMVDAGRGDQREMARNRPLTRPRPGHADLAGMLSYHHSDARNILERASARETAARVALGACAKAYLAAVSQIQVVSNVVAVGPTRDESNRLPTPDEMGALDQSPVRNLDPAVARAFQQTIDRAKTEGDTVGGVAQVVAWNVPLGLGTHVNERDRLDARLAGALMSIQSVKAVEIGDGFVQAHQFGSAAHDEMVWDEDGQLARLSNHAGGLEGGTTNGQPLVVRAGFKPISTVPKGLRSVDLATGAATEPFHQRSDTCQIVPGAVIAEATVALQLARALDERVGGRFVEEARAHLAWYQEYL
gnl|extdb|pgaptmp_001150	K00891	aroK, aroL; shikimate kinase [EC:2.7.1.71]	MRPVVILVGATGSGRTLVGRALAAQLNTTVVETEDVIEAQTGQTLAELALADEPERFREQIGSAATAALGSSGVVTLLPSAVGDPKVAELMAEAGAAGSVLVALTADLTTLARRTGLNAPRSAALGQPRRWFGDHVRALVEQYSTLGAVEISTVDRDPAEVATEICQRFALQ
gnl|extdb|pgaptmp_001149	K02356	efp; elongation factor P	MATTNDLKNGMVLVIDGQLWQVVEFQHVKPGKGPAFVRTKIRNVLSGKNIDRTFNAGVKVETATVDRRDMTYLYRDGADYVFMDKNTYEQVNIPESVVADAANYMVENQDAIVAFHDDQVLAVELPATVVLEITHTEPGLQGDRSSAGTKPATVETGYEVQVPLFLEEGTRIKVDTRSGEYTGRAND
gnl|extdb|pgaptmp_001148	K03625	nusB; transcription antitermination protein NusB	MAKKQGTFSSRTKARKRAADVIFEAQQKGLDRDGEQLLDLLAQRQLVSAAQTPLPAYSRQIVRGVAQFRSEIDGQIQKHSKIKALDRLPGVDLAILRVAVWEMIGNRDDVDPIVAIDEAVAIAKSISTDRSPAFVNAVLDAIRRDLLAQGEVPAVPAEVAPAGEATTDELWDELLDEY
gnl|extdb|pgaptmp_001147			MTLPHLSAEQRKEALAMATAARKRRAEIGAQLKNREMTLAEVIALSETDSAIAKMRVMSLLRALPRVGPQRAHQALDDLGIIPTRRVQGLGCVQRESLVQYFQNLV
gnl|extdb|pgaptmp_001146	K00942	gmk, GUK1; guanylate kinase [EC:2.7.4.8]	MHDPVPVFVICGPTAVGKGTVVKALKAEYPQLEVSVSATTRPPRPGEVDGRDYYFVDEDQFARMRERGELLESAVVHGAHSYGTPRGPVEQSLAEGKTVLLEIDLAGARQVRQTLPEARFIFLLPPSWEELERRLIGRGTEGPEERSRRLETARVELDAAPEFDVCVINDKLDETVKELAEIMGLN
gnl|extdb|pgaptmp_001145	K03060	rpoZ; DNA-directed RNA polymerase subunit omega [EC:2.7.7.6]	MSGIVANPVGITYPPIDELVERTDSKYALVIYAAKRARQINAYNVQLESNMIQFVGPVVPISADDKALSIALREIVEDKLELGASTSK
gnl|extdb|pgaptmp_001144	K13038	coaBC, dfp; phosphopantothenoylcysteine decarboxylase / phosphopantothenate---cysteine ligase [EC:4.1.1.36 6.3.2.5]	MLPPASSSGGAPTVLLGVGGGIAAFKLVTVVRRLRQVGVDVYVLPTEASLEFVGEQTWQELSEHPIGVKVLHGPGELSHIELARRADLFLVAPATADLLAQLRLGLASNLLTATFLAADCPKLLSPAMHTNMWENPATQDNVATLRARGVEIIEPATGALSSGDTGAGRLPEPDAIVEEVLARLAPGALAGRRVVVTAGGTIEPIDPVRYLGNHSSGRQGIELALVAARRGAEVDLLLAQTQVPPPQHPRIRLVHTPTALEMRDALARLVPGAHALFMAAAVADYRPRAAAEQKLKKGTWAETIELVENPDLLREVAEAPWRPEVLVGFGAETGSEEQVLARGMEKARRKGADLLAINRVGDGHGFGEVENRLLVVDRTGQPVADLVGDKSVLAAQLVELAMGVSR

gnl|extdb|pgaptmp_001143	K00789	metK, MAT; S-adenosylmethionine synthetase [EC:2.5.1.6]	MITPLTSESVTEGHPDKICDRISDTVLDAILQEDPAARVAVETLATRGLIHVVGEVTTSAYVEIPKLVREVILGIGYNSSRTGFDGATCGVSVSIGQQSPDIAEGVDRSQEARTRGEPVDRRAAQGAGDQGMMFGYATDETPTLMPAPIWLAHSLARRLAQVRKEELVPGLFPDGKAQVTLGYADGRPVSVDTVVVSSQHDDSWQQGELAAALTRAVIEPVLAETELDQSELRVLVNPSGRFILGGPAADAGLTGRKIIVDTYGGAVPHGGGAFSGKDPSKVDRSGAYAARWVAKNVVAAGLARRCQVQLAYAIGSSEPVAIVVQTFGTGIVSDEVIARAVREVFDLRPLGIIEDLGLVRVRYADLAAYGHLGRDGYPWEETNRVQELRAAIG
gnl|extdb|pgaptmp_001142	K04066	priA; primosomal protein N' (replication factor Y) (superfamily II helicase) [EC:5.6.2.4]	MIARVLIDLDVPHLDRPFDYSIPDSLLASVQVGSLVRVKWGNRRISGWVVELGETSTHEGELSPLLRVLSVQPLFTPAMLQTYRYLAARFAVNLSQVLSLAQPARRQKIEQLLAGATRPVRADWSPAAAVPEYGDWVELLASGPLPRAVVQTVPHRQFSALHALVAECARREIPVIVTAPTLAGAKEIHRRLSEAFSPEVIGLQASELPQTTRYEVQLRTLRGHYLAVVGTRSAIWAPFTKPALQVVWEDGSAHYRERRTPQVDVLDVAVARARWEGYGLVVASLDRSVKAEALVQSGWARSFVPSPTVVRAHTPRIQVVGTDEFEREGMAVLSTVPNAAYQAIRTGLSAGPVLVQVMGVGHFLVLACPACHVRPRCVSCGKLLELTGPPAEAVGHCAGCGATNSVARCLACGHPNLTVLEVGAERTARELGRAFPQTDVVISTSTTKIRRRIRPKPQLVVATAGAEPAVRGGYEAVVVLEASRLAYADRLGAEEEALRRWFYTFALARPGGQGVLSGDVPLEVERALVLWRPTEFAEAAWEERKEIGLYPARWIVALEGPAPAINDILTQLETLPLSPAEPGLQIFGYTPTETDEVRAIVACQAGQALSLMAHLKAMAVARSLRRGPKVKVVVNPPDLFGGTEGLR
gnl|extdb|pgaptmp_001141	K00604	MTFMT, fmt; methionyl-tRNA formyltransferase [EC:2.1.2.9]	MRIIFAGTPEVALPTLQALTQSDHEVVGVLTRPPARRGRRAAPTPSPVATWARERELKVLEAARPGQVAEELAALGADLGVVVAYGAILRPEVLEIPTRGWLNLHFSDLPRWRGAAPAQWAIRAGDQHSATCVFQLEAGLDTGPVYSRLPVELTGRETAGELLERLAPVGAQQVVEVVDQLAAGTAVATPQASEGLTTAPQLTKQDGYVSFAASADETDRQIRSVTPNPGAWTQLPSGQVLRLGAATPLESDPELALAPGQVHADKHQVLVGCERGMLRLGQVAPAGKNWMEAPAWARG
gnl|extdb|pgaptmp_001140	K03500	rsmB, sun; 16S rRNA (cytosine967-C5)-methyltransferase [EC:2.1.1.176]	MGSRRWEATVSKPYRAKRPADPARLLAVRVLLQVEEEGAFANLALPRALRAEQQSNPKFDFRDAAFASELVYGTLRWRGYLDQVLAQFSSRPLTELEPVVWQLLRVGAYQLLFMRVPDHAAVAATVDAARELTTDGPVRMVNAILRSITRADDIDQIFAEIPDQDDRLAARYSHPVWMVRSFRRALEARGLPESELVPALAANNSIPKVNLVARPGLIAAQELAQEAEEILQRPTSQGQLSEYAVILDGGDPAALPAVREGRAAVQDEGSQLAALLLAAAPLTGSDRHWLDLCAGPGGKSALLGAVGAERGVDLVANEISAHRARLVEKSVRALANVTVTNLDGRTLPAPSGYRYDRVLIDAPCSGLGSLRRRPESRWRHQPEELAELVPLQRGLLQRGWELTRTGGVIAWVTCTPQVEETLEIVSWALEQGQVETIDTAATARILTPIELEPGPNQTLQLWPHRHGTDAMFVALLRKK
gnl|extdb|pgaptmp_001139	K01783	rpe, RPE; ribulose-phosphate 3-epimerase [EC:5.1.3.1]	MQIRISPSVLNCDMGRFREELQSIDNADRVHVDVMDNHFVPNLSWGLPIVEAAKQSTEVPIEAHLMIENPDRWARSFADAGCEMVTFHSEAAGAPIRLARELRGAGAKVGLAFKPASAIEPYLDFLDEFDMFLIMTVEPGFGGQKFLPQVLSKTRKLRQLVDQSGIELDIQVDGGINRETVVTAAEAGVNNFVAGSSVFTCADHRGEVDILRRLAQEHS
gnl|extdb|pgaptmp_001138	K24986	ribX; putative riboflavin transport system permease protein	MGTANRWLAWLAPLSLLAFLFLVWLIASGSTSALFLPSPRDFFARAADLFGTRWFWNRTAITFGEALLGSLVGAAVAIPTSWLIHRSRFVNAALQPFLGATQAIPAVALAPLLVLWVGRGLGAIVLLCALMVFFPILVATTVGLRHLDQDVIDAASLDGAHGLRMIVSIEAPLVAPSLLGGIRNGFTLSVTGAVIGEMVMGGAGLGQLLSQQQHNLDTAGMFVTVAVLCALAMVAYSAVYLLERRGKRLVGNSRERRGSRNA
gnl|extdb|pgaptmp_001137	K24985	ribY; putative riboflavin transport system substrate-binding protein	MRKRLLGLAAAGAVALAGCSSTGSDPSKAVTVGLTYIPNVQFAPVYLADFGDLDVKIRHHGSDESLFSALASGDEQVTVASGDEVLQARAQGLDVISVGAFYHHYPVEIIVPADSPIQSLADLKGKKIGLPGEYGSNWFGLLAALEQGGLTRDQVDIVSVGFTQAASLVAGEVDAIVGFSNSEPVALEQMGFAARSIPLDKGTPLVGAAIVSTEGYAQQHPDELRQVISALVGGMQQAIDDPEAAVKATATWDESLSDQQAQAGAMAILQATIPLWQDRQGRASALQDLDAWDRMGPYLAQLLDQPELADEQGATNDYVD
gnl|extdb|pgaptmp_001136	K01523	hisE; phosphoribosyl-ATP pyrophosphohydrolase [EC:3.6.1.31]	MTSFEDLFHELQVKAAEKPAGSGTVAELEQGIHYIGKKVVEEAAEVWLAGEYESDDALALEISQLIYHAQVMAVARGLTLDDIYRHL
gnl|extdb|pgaptmp_001135	K00765	hisG; ATP phosphoribosyltransferase [EC:2.4.2.17]	MLEIAVPNKGSLSEKSVQLLLEAGYRANRRGRELVVDDPENDLRIFFLRPRDIAVYVGQGRIHAGITGRDLLIDSGTPAQEYSQLGFARAKFRFAAPRGTIAQVREISGKRVATSYRTLVENYLAERGIQADVVRLDGAVESSIQLGVADLIADVVETGSTLRAAGLEVFGEPLLESEAILITNVQGQADPAVEVLNRRLQGVLVAQQHVLVDYHVPVDVLPRAVEITPGFESPTVAPLADNAWRAVRAVVRRDKVNQVMDDLLAIGARGTIVTELIASRLG

gnl|extdb|pgaptmp_001134	K13572	pafB; proteasome accessory factor B	MGQSETSLRILVLALNLINTRSGRTRDELQSLVQGYEALSDDTFQRTFERDIAALREAGFQVLVSSNPARYLVERKLLAPSSVELTSAEVDLLLQAAAAWESLSPAELSRLSLKLAGLTNSQVPGQPTIFHRLEGVEHLPQILEAIGQRQPIRFRYSSRRGVADRDVAPLGLMVRGPAVYLWGHDLNREGERYFRLSRIDGKIELVGEPNFYELPAEGPRDFAGDNFRIRPELWIRQGASPLVRLRCESVADSDESRPGWDRCRGRAADWESWERLIVSHCEDVVVEEPTQLAESIYRKLQAAAGEGAP
gnl|extdb|pgaptmp_001133	K13573	pafC; proteasome accessory factor C	MEDKTVTLARHLALVVTVAQEPGIRVAELARRFNRSEEDILADLEVLDRAGFGSQMPDEMFGFEWDRLRSRGEVKLWFDLNVTGVPPLSEQEAAGLITGLAALSPQLPPEVAEGIPTLLAKVLSLAPALADRSLGDLTQLLAVIDPEPASEVEDTVRAAIERREQMRLTYLSARSHRSDRTVDPISLTQETDGWVLRAWCHLVEAERHFRLDRILAAELTGHPAENRLGGRASKGSKCTVMLQPEAKWLVGQLPLRDVRSEGELIVGTFRVFDDDWLTAQLISLAPYLVAVRPQHWADRARERAQR
gnl|extdb|pgaptmp_001132	K03727	helY; ATP-dependent RNA helicase HelY [EC:3.6.4.-]	MAADEPSAAERMRLFHQQMRVDAAGVDDYLAELPFSADPFQRTALTHLALGRSVLVSAPTGAGKTIVGEGAVYLALREGTRAFYTTPIKALSNQKFREFSARFGADRVGLLTGDTSINPGAEVVVMTTEVLRNMIYAGADLSELGVVILDEIHYLADRVRGPVWEEVLIQLPEEILIVALSATVSNIEEFGRWIREIRSSCEVVVSSDRPVPLYQHMMVKHRIYDLFTTGHTLNPILADVVAEGRRPGRRPIPARINRPRIVELLERKSLLPAIVFIFSRAGCDEAVDQLLAAGVSLTTTSEQRLIREEVEQVLLAVPIADHGVLGLSRWQVALESGIAAHHAGMLPLLKEAVERLFARGLIKVVFATETLALGINMPARTVVLEALEKWNGSEHVRLTPGEYTQLTGRAGRRGIDVEGHAVVLQRGVVQPEELAQLASRQAYPLKSAFYAGYNMVVNLLAHASVGEVREVLESSFAQFQADDAVVGLASKLRRTQSRLAEVSAELHCSQGDAGEYFGLREDLSQLQKRLQRANREGNRTLVAHQLSRLRSGEVVSYRLRRQPQVAVVVGPAGGDWGPPAVRAIGTNGKMFLLSASDLTEPPTRVGQIRLPRSGVRRSRDRAQVAGQLRDFSVAPPRRQSRETPAEVRRLQQEERELEARIRQHPVHSCPDREQHAHLGHEWARIRRERDKIARQINARTSSIALEFDRVLSVLTELGYVRDGCVTERGEQLRQVFGERDLLVTECLQEGIFDGLSTEELAALCSALVYEPRGEAGEAGYLPTRALQQAFGRMGRVFARIHREEQRARLERTKEPSPGLVAVTYEWARGASLSTVMLEADLPAGDFVRWMRQTIDLLEQLRHLDERAEAAVKVLRRGVVQWTQG
gnl|extdb|pgaptmp_001131	K03820	lnt; apolipoprotein N-acyltransferase [EC:2.3.1.269]	MDARMILLVVLSGLAMWGAFPDQGWWPLMFVSLALLTGVVRRASAGWGAAYGALWAMVFFLPHISWMNIATNQTYLAWILLALAQAFFLALWGLSFAAVSRWPAAQTWWGEALAAAILWVGFEQIRARVPLGGFPWAKLPYGLVDSPLVHLAPWGGEVLVSAAAVVVAVWARRAVTGQWWLAVPALALFGAPALVPLETAPQNGTVNVLAVQGNVAIPMEETYAVAGQVTQNHVDETLRALDAGADPDLVIWGEDSVDRDPNTTELTAGLVEQVLERGQVPLVAGYQEYAHDERYNWYSVWYPGTGQGPDRYGKQHPVPWGEYVPWRSLSEFLATEAAAIRVDMASVDNPGLLPVTLADGRVVPLAVGICFEAGDEPIIAEGVRLGGEAIVIPTNNSQFRASAESTQQLQMARFRAAEFSRATLQVSTNGVSAVISPDGQVVAETDRQTAAHLSAEIPLRTSLTPAARWETALTGGAMGLAVLLGIGGLAWG
gnl|extdb|pgaptmp_001129			MADRALRGMQIGSKSLESEDGVIFAGRVEANYVCPRGHSFTVTLAADAEKPPTWECRCGQIAELVGETEAEEETKPAKPVRTHWDMLLERRSLEELDQLLQEQLEAYRSGELQLDTSYRRG
gnl|extdb|pgaptmp_001126			MASSNAVRREEIEPDQSWPRGFSHDPELSIGAVTEIIAGEFPATSVSKIRFLEDKGLIKPHRSPSGYRKYSRADVERIRFILAQQRDSWAPLKVIGDQLRALDAGHDIEPVPTARLVASEGKTVIPRSQETLSARELSDLTGVATERLEEYAQLGLIVPDLGGYFLTRTVSVVNLIVMLENAGIPARVLRSVRQGAERSADIVDQVITSRENRTKPGERERSRAQAADLSQLFGRLHQELLAVAVEKLSHT
gnl|extdb|pgaptmp_001125			MAQEPKNDPTQTAFFGAAAEEPLVEPLPLSERDREAVQALPAGTALLIVQRGPNSGARFLLDAPVTNAGRSPSADIFLDDVTVSRKHCQFIADNGGHIVRDSGALNGTYVNRERVDQAVLKEGDEVQIGKYRMTYQPSPHGK
gnl|extdb|pgaptmp_001121	K01928	murE; UDP-N-acetylmuramoyl-L-alanyl-D-glutamate--2,6-diaminopimelate ligase [EC:6.3.2.13]	MSSTASLRPQIEPIPLAEALVELSLSGPIPGGDRLVSGVSVDSNDLEAGWIFVAVGGETFHGIKFAPAAAERGAVAILTDERGVREAAEFKLEVPVIEVPDPRLAAALVAHRIYASTFADLVLVGVTGTNGKTTTTYLVRGALTPKYQRVALMGTLEVAVSDTPIVSSRTTHEAPVVYRALAVAAQNGYRAAVVEASSHALSLHRIAGLEFDSVLFTNLQHDHLDFYGTMDYYFAAKAQLFERTRARRGVVAVDDQWGQLLAHRAQIPVDTVSALSPTPPDLIGDQHHWAVTSLHNDPERWGISFQLRDPAGQQHDCFCPIPGKVNVQNAALALVAGTQLGVTLDEARAGLAETAPPPGRMELVTTIDRQPRVLVDYAHTPEAMSALLETLRPLVAGELIVVFGTDGDRDASKREELAAIAARGADRLWVTDENPRTEDPQQVRDYLIRGIRSVRPDLDRVLEVKTSRRDAIRRAILAGRPGDLVVITGKGAEPYQEIDGVKHAHLDSAVA
gnl|extdb|pgaptmp_001120	K03977	engA, der; GTPase	MRLALDQYELDEEDLALLADESLEAEEQRALSTLPVLAVIGRPNVGKSTLVNRILGRREAVVQDEPGVTRDRVIHQAEWAGRDFYLIDTGGWEVDVKGLARSVAEQAEIAIDQADAIVFVVDATVGPTATDERVVELLRRSGKPVVLAANKVDSAVQEADAATLWSLGLGEPFAISALHGRGTGDLLDALLQALPEEAAAARRMANDRMRRVALVGRPNVGKSSLLNALAGEDRAVVHDLAGTTRDPVDEVVELDGRLWNFVDTAGIKRRLHRTRGADYYASLRTEVAIERAEVALVLLDASVSLTEQDVRVIQQVVEAGRALVIVNNKWDEVDEERQWELRREQEKDLGQVQWAPRINLSALTTWHVNRIPRALDQALAGWETRVSTGKLNSFLGQLVAANPHPVRGGKQPRILFATQASTRPPRFVLFTTGFLEPTYRRFIERRLREEFGFVGTPIQISVRVREKRGRR

gnl|extdb|pgaptmp_001117	K00872	thrB; homoserine kinase [EC:2.7.1.39]	MWALGRQVTVEVPATSANLGPGFDSLGLALNWYDRSTVTVIEDGFEIVVHGEGADSVPCDQSHLVVATILEALADWGLQVPGLRFEATNTIPHGRGLGSSSAALVAGLLMAWGLAHPHEEVDRVWLLREAFAREGHADNVAPAIHGGFVITWAGAHNDSPDAGHRSRPSQIHSAVSAVALVPGVEVLTTAARSVLPSEVPFSDAVANASRAALLVHAMSQEPSLFLEATSDRLHQVHRAELMPKSIQLVKELRRAGFGALISGAGPTVLVLLDQDQLPGLRRAIAAADPNREFSAHELRPGY
gnl|extdb|pgaptmp_001116	K00133	asd; aspartate-semialdehyde dehydrogenase [EC:1.2.1.11]	MSAALTVAVVGATGQVGGVIRTLLEERAFPATKFRYFASPRSAGRTLPSPVGEVTVEDLTQVTVEDLRGIDVALFSAGATASKQYAPLFAEAGAVVVDNSSGWRKDPEVPLVVSEVNADQISARPKGIIANPNCTTMSIMPALKALDNAAGLERLHVASYQAVSGSGLAGVRELAGQIRAVADGPIEELTFRGDAVQLPEPNVYVAPIAFNVVALAGNLVEDGSGETDEEQKLRNESRRILNLPDLAVAGTCVRVPVFSGHAMAVHAEFSREILAETARSILETTPGVEVVEVPTPLQAAGADPTFVGRIRQDQSVPGNRGLVFFVAADNLRKGAALNAVQIAELVVAERA
gnl|extdb|pgaptmp_000196			MQKVWSFLRQYPVVAMALAVLIVGLVLNASEQTAIAHALISAFSGAFALWTAVGMIRDIIRGHFGLDILALLAIVSTLLVGEYWASIIVVLMLSGGEALEDYAANRAQRELTALLDRTPTSAHLLARAGETNADSSGDWSDTDVRDIEAESVQVGDLLLVLPGEIVPVDCILLSPTGSFDESSLTGESLPVSVVSGDELPSGAVNGSQAVRVRALKRSADSQYQQIIRLVQEAENAKAPTVRLADRFAVPFTVVALLIGGVAWIVSGEPVRFVEVLVLATPCPLLIAAPVAFMGGMSRSAKNGIIVKGGAALEQAAKVKSIAFDKTGTLTDGEPVLVEARPEPGSSSERLTQLAASAEQYSSHVLASGIRAGATSAGLTLENAAHASEDTGQGVEADFGGDPVRVGRYSYIQAVAPSAKRAQLEPGQVAVYVAEGSRYLGALILADHIRDESRAVVQWLGDHGVPEITMVTGDSAGTAQEVAKLVGITQVHASMRPADKVEVVRTLSPKPTMMVGDGVNDAPVLAAADLGVAMGARGSTAAGEAADVVILQDSLRPVVTLVAISRDTVRVALTSIWLGISLSVALMLVATTGAIPAVVGALLQEVLDLVAIVYSLRALTGRAPVLPEGGSQVPVGLPSSAKRAEVEPPTGMSISGQRPDSNG
gnl|extdb|pgaptmp_000392			MKVIVVGGVAAGMSFAARARRLNEDAQIIVLERGEYVSFANCGLPYFVGGEITDPDRLLVQTPQKLRNALNLDVRVRHEVTAVDTQAQTVTIQTTSGPTTETYDVLFLAPGAEAMRLPIPGIDSDRVLTLRTVDDARQMRAQVEAGIKRAVVLGAGFIGLEAAEALAQTGVDVTVVELADHVLPPLEVEVAHPVQDELGRLGIKTHLGVAASAIRPGAHSDTVVLADGTELQADLICLSAGVVPATAPFAAAGIETVRGAIVVDERGRTSAPNVYAAGDAVTSTDQITGEVRPVALAGPANRAGRLIADFVMRGEAARPLPRPLGTAIVRVGRLTAAMTGANRAALERNHIAHTTLHLHPNQHAGYFPGASQISLVVHIGEDGRLLGAQAVGGEGVDKRIDVLATAIRAQLHVEDLIDLDLSYSPPYGQAKDAVNLVGMVGANVLDGTLRLWYPAQYEEVAQSSLILDTRSRAEYETGYLPGSLNIPHTELRDRLDEVRGAASGRPVRVLCASGVRSAIAHRLLTQSGFDSASLSGGITSLRQYLGKRADTVIKV
gnl|extdb|pgaptmp_001106	K00147	proA; glutamate-5-semialdehyde dehydrogenase [EC:1.2.1.41]	MSNQDDVTQQILAIATRSRQAARKLAGASRNLKDGALHAMANALLQHQGQILSANARDLERGRTGGMSEGLLDRLELTSERIEQISDSLLELASLPDPVGEVLRGSTLPNGIRLQQVQVPMGVVGMIYEARPNVTVDAAGLGLKSGNAVILRGGSAAAETNTQIVQILSEAVASVGLPRDAIQSIDEFGREGAVALMKARGYVDLVVPRGGAGLIQTVVNEAKVPVIETGVGNCHVYVDREVDQDQALAIVINSKTHRPSVCNAAEKLLVHQDRVHDFLPRVLSALSEAGVQLTGDERVRAVTPPGVVVEPAEEDDWACEFLALRAAVRVVDSLDEAIDHIGRYSSGHTDAICTTSLAAADRFTAEVDSAVVNINASTRFTDGGQFGLGAEIGISTQKLHARGPMG
gnl|extdb|pgaptmp_000577	K07816	E2.7.6.5; GTP pyrophosphokinase [EC:2.7.6.5]	MSSDDDATIQPAGLDPQQIREQFVQMRRLLMVYEHALQTVLLKVDLLRDEFRLERDYNPIEHVSSRVKSPQSITDKARRKGVAVTTESLRANILDIAGLRLICSFQNDIFQVRERLLTHGDLRLREERDYISNPKPNGYQSLHLLVEVPVYLAGGRQWVPVEIQLRTVAMDFWASLEHKIHYKYRGEVPPDLTSSLKLAADMASTMDRTMERLYEEVRSLQPPSEPTEADPMLPLFRALESASMLEQFTRRDDDE
gnl|extdb|pgaptmp_000431	K00849	galK; galactokinase [EC:2.7.1.6]	MSVVIRDAWTRPDGAARVATLFTETFGQEPDGIWSAPGRVNLIGEHTDYNGGLALPIALPHRTYAALRRRDDDQVRLISAQEESQRSVDLAEAGPVGTLGEVDGWPAYVVGVAWAMRQAGYPVAGFDIAIDSCVPYGAGLSSSAALSGSAGVGLTDLFGLDLDREQLVRFCIAAENQMAGAPTGGMDQSASLRCQPGYALLLDNRDHSVQQIHFDLAARDFRLLVIDTKAPHALVDGQYAARRQTCHEAAQQLGVEYLADITDLDEALARLTDPVAQSRVRHVVTEIARTRQVAELLQKDADLAEVGRLFTESHISLRDDYEVSCPELDCAVEEALRAGAVGARMTGGGFGGSAIALVPATTVGEVMDGVVQAFAARGFAAPEFLVVEASAPGGRDA
gnl|extdb|pgaptmp_000973	K00641	metX; homoserine O-acetyltransferase/O-succinyltransferase [EC:2.3.1.31 2.3.1.46]	MDQDRLPPASGAWQETDPVGQRQFAHVGPLSLEAGGHLPEVTLAFESWGHLNEDGSNAVLVLHALTGDSHISGEVEPGHPTEGWWTDLIGPGAPLDTDHYFVVAPNVLGGCQGSTGPASLAPDGTHWGPRFPFLTIRDTVQAEVRLADQLGISQWQMVIGGSMGGMRALEWATMFPDRVAKLVPIATVARTSADQIAWAHTQLAAIVSDPGFQGGDYYGGPPGSGPHTGLSIARQIAQTTYRSAEELEARFGQMAQGEENPWEGGRYAVQSYLDHQGDKFVYRFDANSYKVLTEMFMAHDLGRGRGGVEQALGTISAQALVVAVDSDRLCLPEENRRIAEFLPHCVGVEMVNSHRGHDGFLIEFDQFGPLIKQFLNS

gnl|extdb|pgaptmp_000972	K01740	metY; O-acetylhomoserine (thiol)-lyase [EC:2.5.1.49]	MTQEWSFNTTQIHAGQNPDSETGARALPIYQTTSFVFADADQAANRFALAELGPIYTRIANPTTEVVENRIAALEGGVGALLVASGQAAEFLAILNLAHAGDHIVASPSLYGGTVNLFSQTLPKFGITVDFVENPGDPASWLAAVKPNTKAFYAETIPNPKGDILDIEVISGAAHSVGVPLIVDNTVGTPYLVRPFEWGADIIVESTTKYLAGHGTSIGGVIVDSGRFDFGADPEKYPGFNEPDPSYNGLVYARDLGAEGAFGVNLSYILKARVQLLRDIGAAISPFNAFQLSQGIETLSLRMDRHVDNALAVAQYLEAHPAVTKVNYSSLPSSPYYHLAQKYTPRGAGAVLSFEVEGGAEAGKKFVSALKLHSNVANIGDVRSLVIHPASTTHSQLSEAEQAAAGVAPGLIRLSVGLEEISDILADLNLGFAALEG
gnl|extdb|pgaptmp_000570			MTRTNRAEPPSVRAQIISAISGGLAETRVDLAKHLQLAPSTVSVHVHDLLAEGVLSEEGQADSTGGRPAKILRLLAPAGHLLVAELGGTHARLGLADLTGKLLATVERPINIEDGPDAVFDTLTEYFGDLEADYGHTSARRGVCVGLPGPVDVAKGRVETPSRMPGWNHYPAGRQLRDRLGTHALIENDANLLALGQDSLHPGLSSSITVKAGTGIGAGLVIGGALHHGATGMAGDLSHARLPDAGDILCACGNTGCLETVASGAALVREMRATGYDVTSLSEIIELANNGDGLATSLVRGAGRHLGEMLCPIVGFTNPQAVYLGGLLSTLELFVASVRSQLYDGSHPAVTKNLIIDRTGEGPDLALIGGARLLAQTILAESAR
gnl|extdb|pgaptmp_000567	K00639	kbl, GCAT; glycine C-acetyltransferase [EC:2.3.1.29]	MYTNRSQLIAELDSIRAAGLYKEERALTTPQGAHVETATGPALNFCANNYLGLADDPRIVQASRDSLDRWGYGLSSVRFICGTQDQHLALERELAQYLRRDDAILFSSCFDANGGIFAALFGPNDAIISDALNHASLIDGIRLSKAQRYRYQNRDLEDLRTQLSTARAQGAQTIVIVTDGVFSMDGYYAPLPGICDLAEEFEALVMVDDSHATGFVGPEGRGVAARYGVEDRVDILTGTLGKALGGASGGYVAAHQEIVDLLRQRARPYLFSNTLAPSIVAGAREALRLASRADQARAHLDEVSELFRQLMEDAGFELLPGSHPIVAVMFPGDDGAHLATAVASKMLELGVYVTAFSFPVVPRGEARIRVQLSAAHSEADVRQCVSAFTAARAAVS
gnl|extdb|pgaptmp_001323	K09710	ybeB; ribosome-associated protein	MTCTNHALELLEIAAKAADDALGLDPLALDVSEQLYLADIFFLVTADNPRHSRSIAEEITTEIKKATGQLPVATEGELGSGWVVVDYGDLVVHVLQPEERDFYALDKLWDKSPRLVTA
gnl|extdb|pgaptmp_001325	K03979	obgE, cgtA, MTG2; GTPase [EC:3.6.5.-]	MAEFIDRVKIFVTAGDGGHGCASVRREKFKPLGGPDGGNGGNGGSVVVQVDSNVSTLLSFHHRPHHKAKNGEPGKGDLRQGKNGDDIILPVPIGTVIKDESGELLADLQGDGARYVIAEGGRGGLGNAALANRARKAPGFALLGEPGEEATVVLELKSVADVALVGYPSAGKSSLIAALSAARPKIADYPFTTLIPNLGVVQAGEVRYTIADVPGLIPGASEGKGLGLEFLRHIERCSVIAHVIDCASFDPDRDPVTDLQTIEAELGAYEGDLAQIEGYVPLMERPRLIVLNKVDIPDGQAMADLMMPELERLGWPIFAVSAVTHQGLPALSYAMAAEVEAQRALRPALEETRVVLTPKAVGTKNAPVTVTPVQFGGETVFQVRGAKPERWVRQTDFGNDEAVGYLADRLAAAGVEDALVKAGAVPGSPVVIGPVEGGMIFDWEPTLSTGAELLGGRGEDPRLDPNLRRTTTERRREYKARMDGKEAARQELRAERDRGVWTDPEE
gnl|extdb|pgaptmp_001326	K02899	RP-L27, MRPL27, rpmA; large subunit ribosomal protein L27	MATKKGVGSSRNGRDSNAQRLGVKRFGGQSVNAGEILVRQRGTKYHPGANVGRGKDDTLFALSAGSVEFGNYRGRRVISVVEA
gnl|extdb|pgaptmp_001329	K00425	cydA; cytochrome bd ubiquinol oxidase subunit I [EC:7.1.1.7]	MEILPTVLDPVALGRWQFAITTVYHFILVPLTIGLSLGVAIMQTIWHRTGNEAWLKATRFFGKLLLINFALGVATGIVQEFQFGMNWSEYSRYVGDIFGAPLAIEALLAFFLESTFLGLWIFGWGRISKGLHLAMIWMVTIGVNLSALWILIANSWMQHPLGAVFNPATGRAELDGTSGFLSLLTSPMTWLTFSHVITSSWLLTGTFIAGIAIWWMTRSAREGGESGMNEARNVWLPITRFGLLVVIIGGLGTVTTGHFQGQEMVKFQPAKMAAAEGICMDTEGAAFTVAQFGSCPLDGSGEPTRLLTVPGVASFMATNSFSAEVEGVADIQERFVEMLNQNEAFVAQYGDASQYEFIPPQMVVFWSFRIMIGLAAFSAILAIWGLVATRGGRVPTSKRLGTFALICLPMPFIAASAGWIFTEVGRQPWVVHPNLSAIEGGDPVSQVLMMTDLGISTAVPAWQTLTTLILFTVLYAALGVVWYLLMRRYVLEGVHDGDKGTADDGTK
gnl|extdb|pgaptmp_001330	K00426	cydB; cytochrome bd ubiquinol oxidase subunit II [EC:7.1.1.7]	MEFLSILWFILIAVLWTVYLVLEGFDFGVGMLLPIAAKNDRERTQLVRSIGPHWDGNEVWLLTAGGATFAAFPAWYATMFSGMYLALFLILVLLIVRITAIEWRSKLRSERWRANWDWFQTVASFGVPLVLGVAFSNLVQGMQIEAQNRLTGAIIPPEQVAAVAGDGTTVYNLTGGFFSLFTPFTLLGGVMLLLVCLSHGIQFVSLKTDGLVAERANRLAGPVSVVATLLAAVWVVWGQFAYSANVLAWIPLVIAALALITSAAFSQPALRSPGKAFAASAIGIASAVAWIFSAMAPNVMKSAIAPEYSLTIPLASSTNGTLTVMTIVAVVLVPIVLAYTLWSYWVFRDRVGHRDVTPDSGLWPAKIRLGANFLTGKQ
gnl|extdb|pgaptmp_001331	K16013	cydD; ATP-binding cassette, subfamily C, bacterial CydD	MKPLDPRLLRYARSARRYILEIALLGTLMAALVIGQAFLISGAASPVITRSATLAEIMPTVGWLALVILARGLLLYLRESRAHRAADQTVAELREQVVNHAVALGPRWRATHGSNTVTLVTRGLDDLGPYFVRFLPQLLLVMTVTPATLVAILLLDFWSALVALVTIPLIPIFMILIGRLTQSASATKLAAMERLGSQLLDLLTGLPTLRALGRERGPRTALVDLSGRNTKATMQTLRIAFLSGGVLEFLATLSVALVAVQVGFRMVAGNIPLATGLVIIMLAPEVFEPLRQVGAQFHASANGVAAANSCFEILEQTPPPSGDEPAPDLTRTRLVFDGVSVAARGAWAPAELTEVVEPGQITALAGPSGAGKSTAVAALLGLEPPTRGQVLIGSVPLSRINLPSLWRQLSWVPQSPTLVPGTIRENVSPVASVEELDEAARLTGFDQVLASLALGWETPLGLGGLGLSVGQRQRLALTRALLVRPPLLVLDEPTAHLDALSEDQIVQILAALRSRGTTVLVIAHRAAVLGVADRVIQVQSAPATEEEMDRYPQLASSWTGQVIPVEMPGFLDPRALTEAEAHR

gnl|extdb|pgaptmp_001332	K16012	cydC; ATP-binding cassette, subfamily C, bacterial CydC	MKEDLRALRRVLKLLQIRPGGFALSLLLGVCGLGAAVALAATSAWLIARASQMPPVLTLTIAATSVRFFGISRALFRYLQRLASHQVALDGMDRLRLGIYDLLVAGPIERVARLQRGDLLNRTGADVDTVGDFVVKSLLPALVALIVGLGTVVGFAFLSVPAAVILAAMLLVSGVVVPLLTMRSTRAAEAAELESQRDLAVSAMTIMDGADELRVSGQLPALQAQLGAVTHRINRARAAADRPAALGTALDRVAMGLAVVGVLLVATPEVGATTLAAVAFAVLVLTPLSAFEGTAELAPAAAQLVRSAQAARRIVDLLGPEDEPERPVHTVPAGATLVAQDLAVGWPGHPVVAEGVSLELKPGTTTAIVGPSGIGKTTLLYTLAGMLAPRGGLCQLNGAATWGADRDQFTSVVSLTTEDAHIFGTTVFENLRVANPKLTREEAQDLIGRMGLRLWLEALPDGLDTLLTPTSISGGERRRLLLARALAAPAPLMLLDEPGEHLDSQTAQEILHELLAGTGKDRGVVVVTHRLSELALADRILLVEPAPDNGDAPARVVKVGTHEELLNNSSYYRWALEQEK
gnl|extdb|pgaptmp_001333			MAEAIVDLSGQVTGSTRAAVCDSNWVPLASHGLGADELHALADRQESPSALWPTFTDGITRELHPGADLLVLLAYSEPVDSDLWYRFNQIVRAGIQAWQLATARQLRLSWGPVTREFTQAVVADLDEEESLSLATELALRGGSAVGALLFLPSINRELACEFTAGEDTARFIGMPLEITELTREAFARGEGQCAPAYSQLVHDPLGALAQYGSGLVFPLLAGDETVGAMLLLRAPEAPSFSGEHLPHIQNFAATISLSLELAQGRQAQSVALMLEERDRIGRDLHDLGIQLLFATGMQLDKLRAEVEEGRYSNRRIAEELRGAIVSLEDAVGQIRQVVSGLKDTEERQTFVEQLEQEASRSRRVLGFAPSLILELDGHILEAGSDTWAERSAELTRRVGEELAEDAIATIREALSNVARHAGARSVKIEVSVNGRAPVGELLVSVIDDGRGINLSRNRSSGIANMGNRAAYRGGSFAVGMGPRGRGTSVVWRVPLLVK
gnl|extdb|pgaptmp_001335	K10563	mutM, fpg; formamidopyrimidine-DNA glycosylase [EC:3.2.2.23 4.2.99.18]	MPELPEVETISSGLARRIVGRTVTEVVGDQSRLFRHNPDGWETVSRALLHREIGAVGRRGKFFWLALAPQDQVGDGTRCLVIHLGMSGQVHAQLAGYSVPWGHKHEHLWVGLDNDASLSFVDQRQFGHLTVSRLEPAGPALIPGAIRHIAPDPLEAAFDLVAVTERARRSNRTVKSLLLDQGLVSGIGNIYADETLFRSRWPGWTRGRELSVDDWAQVFAHARQILTEAIAAGGTSFDELYVNVEGDPGYFSRSLAVYGREGKPCPGCGQPIERIVLEKRSHFYCPSFCGEAPPAKVPESGNFQ
gnl|extdb|pgaptmp_001336	K03685	rnc, DROSHA, RNT1; ribonuclease III [EC:3.1.26.3]	MSRGISGALNQNLGELLERWGTPIDPELLVTALTHRSFAYEQGGLDHNERLEFLGDSVLGLIVTDWIYRHFPSKPESVLSRMRIATVSQAPLAQTARALGLGDFLLLGVGEHKTGGPDKDSILCDALEALIGATYLSTGMEVTRQVVLQALSPLLEEVESLAQTTDWKTRLQEYIASSPYRDYQYQVDASGPDHRRQFAVTVLVDGQPTGRASASSRKHAENLAARDTLVQLVGPEVDPTVSVLPSDR
gnl|extdb|pgaptmp_001337	K07040	yceD, ylbN; DUF177 domain-containing protein	MSAKELQISLIDLPAEVGAHLEKTVDWVVPSGWSTGVLTLEEGQHLPVTVSLTALEDGVFVQVSAQGQLVGECVRCLDPVSVDFSADAGDIYVEGERSTDRKAHSGSADEIEVEGDDLDPVKVINRDTVDIEPLLRDAVFADAPLQPVCDEDCQGLCPQCGRRMDELEPGHHHEFTDPRFAALEGFFAAGDNEDE
gnl|extdb|pgaptmp_001338			MPERYVEPSDDQPQTSSIDLSAQDAVYGPSVLIAALDQIEVMVDEARAVPLSPNVIVNKAGIVDLLSQAREALPEDLIAADAVVADADAVLDRADATAEVTIAEANAKAKSIVEEARERADLMLREATEESDRKVARAQEEANQIRQRTQDDVERMVADARAQAEQMVARETVLVQAEDKARQLLHEARTQAGDLRLGADDYAATTLSQVSQVLADLARRTEAGRRTIAERSGFDRPDVDLQP
gnl|extdb|pgaptmp_001339	K00954	E2.7.7.3A, coaD, kdtB; pantetheine-phosphate adenylyltransferase [EC:2.7.7.3]	MPVAIMPGTFDPPTRGHLNLIARAANCFDSLVVAVGENIKKTPWFPVERRVELLQASIALDLPELTDRIEVAAYRGLVVNFAATRGATVIVKGVRDGLDLAAEQIQATHNRELGGLETLLLLAEPRWQHVSSSAVKELVTWEVDPLPYVPAPVAAALAHRTS
gnl|extdb|pgaptmp_001340	K08316	rsmD; 16S rRNA (guanine966-N2)-methyltransferase [EC:2.1.1.171]	MTRIVAGTAKGRQLAVPAQGTRPTSAKVREALFSMLESWNLVRERRVLDLFAGSGALAFEAFSRGASEITLVDKSRGAQKVLAQNARTVGASSARIVAGSAQAFVRSARATFDLVFVDPPYAWPEPDLTDLLADLLGLLDPDGLIVVERDARSPQPSLPAGLELEAERSWGDTRAYLIGRPPADPDAD

gnl|extdb|pgaptmp_001341	K03655	recG; ATP-dependent DNA helicase RecG [EC:5.6.2.4]	MKQPLETPAANFDPLAVPVKLRVTPAAARHLASLGIETVGDALNYAPRRYYHWGRLTALSQLNEGEEITVLAQVISARLVHNRSGKGVRFLVGITDGSSELTVTFFARNEYALTHHQRLLQPGETFLFAGKVSEYQGNLQLVQPSFEEIEADSEQSVERRRGRPIPIYRAKASVPSWKIAGLIDQIMATVNWEQVPDPLADPDGGLGQRHSLLSLSRAYQLLHQPEDDTDWQNARRTLAWVEALTLQTSLLQPRVQADLAGTHRSRPLVPGQGEELVERLIRGLPFQLTGGQLQAWQQISSDLEGTSPMQRLLQADVGAGKTVVALLAMLRAVENGQQAALLAPTEVLATQHLASIQRLLDLAQVEVPLHLLTGSQPASARENTLMHLAGGEPSLVVGTHALIQDGVEIPNLSVLVVDEQHRFGVSQREHLRQGRDPRPHLLVMTATPIPRTIALTVFGDLDVTAMRELPAGRTPVQTHRVPQENPVWMARLWQRAREEIAAGGRVYVVVPRIADEAEPAAPGVPLPSVDAVAAQLRAEPALAGIEVGIAHGQAKPEENARALERFARGEAPILVATTVVEVGVDVPEATMMVIWGAQQFGLSQLHQLRGRVGRSDRPSVCMLVHPVALNELAEARLQALVEHSDGFALAEADLRLRQEGDVLGVVQSGGRSSLRFLSVRRDGAIIAAARTEAERILAEDPQLEQHS
gnl|extdb|pgaptmp_001342	K02902	RP-L28, MRPL28, rpmB; large subunit ribosomal protein L28	MSSVCDVCGKGPMFGKSVSHSHVRTNRRFNPNIQRVRAVVNGTTKQINACTKCIKSDRIVRPA
gnl|extdb|pgaptmp_001343	K00946	thiL; thiamine-monophosphate kinase [EC:2.7.4.16]	MKLSDLTEGQIIASFQGLIPRGNFTDLGSGDDCAVVRAPGGRFVVTTDVLVAGQHFRLDWSSPEQIGARAAAQNLADIAAMGAEPTALVVSLVLPGDLELDWLTRMVAGMAGEVRPTGAGIVGGDLAVGDQLVIAVTAHGHVGERPITRAGARPGDTVAVVGTLGRSAAGLAALSRGLVAPELVGSAIPETFREAVSTYRVPKPPLRAGRLAAERGATAMMDVSDGLVLDATRLAEASGVHIDLSATALGPDRLALSAAGEALGTDPMTWVLTGGEDHALLVTFPAHVLVTDPFRAIGTVGAIGVDPSLGPRVTLEGRPITGGWDHFHQS
gnl|extdb|pgaptmp_001344	K03437	spoU; RNA methyltransferase, TrmH family	MSERLPVLANPRSDRMRTVASLGRRSFRLRKGLLRVEGPQAVRELLRFRPDHVTDVYATETAQARSADVWQQAREVTRWHHEVSDEVAEAVAPDAQGIFAVAHLAAVGGDPYQAELERPLVVLPSTQDPGNAGVIIRSADAFGAGGVITCVGTVDLTSPKVIRSSAGSIFHLPLAQGQDFAEALARLRADGRQILGTSGADGAIEAGTLDLSRPHAWLMGNEAQGLSAGEAAACDQLVRIDMTGAAESLNVGVASGICLYLSQLARST
gnl|extdb|pgaptmp_001345	K02887	RP-L20, MRPL20, rplT; large subunit ribosomal protein L20	MARVKRSVNAKKKRRVVLEQASGYRGQRSRLYRKAKEQVTHSMVYSYRDRRNKKRDFRRLWIQRINAASRANGLTYNRFIQGLNLAGVEVDRRMLADLAVNDPQVFAALVAQAKAALPADVNAAKTA
gnl|extdb|pgaptmp_001346	K02916	RP-L35, MRPL35, rpmI; large subunit ribosomal protein L35	MPKNKTHSGAKKRFRTTGSGKLMREKAGKRHLLEHKSSRRKRRLSGDTTVARPDLKEVRQLLGK
gnl|extdb|pgaptmp_001347	K02520	infC, MTIF3; translation initiation factor IF-3	MGTKAFLCLKRERKETPISDPRINDRIRVPEVRLVGPGGEQVGVVRVEDALRLAEEAGLDLVEVAPDARPPVAKLMDYGKAKYEAAQKARDARRNQANTQLKEIRFRLKIDDHDFEVKKGHVVRFLNQGDKVKVMIMFRGREQSRPEAGIRLLQRLAEEVGELATVESLPRQDGRNMTMVLAPTRRKSDALTDQRARRQAERDARRQKHEERAQKDAQRKNQS
gnl|extdb|pgaptmp_001348			MPSALNPDKFARLARRLASRPDRSDHGQLLPALGAALEVEELGDRLARVVAALGSERLIVPVPVEAHPDQAGGEHRPQDLDPDSQIPLATDERDGVTAIAVFSSAEALRHWDREARPLALTSQKVAITAIATGVPRLRLDPADQSLLLGRPAVEALAAGDRWLPGWEDESLRAHLSARAAELAPWASFVSVTLRPNPTGLTVEVSAAPREGHRRVREDFAALLAQLGQDPRLGAAAESVEFNPRLVHLA
gnl|extdb|pgaptmp_001349	K24017	priA; phosphoribosyl isomerase A [EC:5.3.1.16 5.3.1.24]	MLTLLPAIDVVDGQAVRLRQGEAGSETVYGAPVEVAADFVAAGAQWIHLVDLDAAFGRGSNLDLLRQIIAQVPINVELSGGIRDQASLEAALECGARRVNLGTAALEDPEWTERAIAQFGDRVAVGLDVRGETLAARGWTREGGNLWEVLARLDAAGCARYVVTDVERDGMLSGPNVELLRAVCARTDAPVIASGGVSSLEDLRTLRGLVGDGVEGAIVGKALYAGAFTLAEALAVAE
gnl|extdb|pgaptmp_001352	K01693	hisB; imidazoleglycerol-phosphate dehydratase [EC:4.2.1.19]	MTEPRLATAHRETSESTVSVRVNLDGTGRPQIDTGVPFYDHMLTALSRHSLIDLDVKSVGDTHIDVHHTVEDTAIVLGQALAAALGDKRGIRRYGTGFAPLDEALARAVVDLAGRPYVVCQGEPAGQEYHLIGGHFTGSMTRHVFESFAFNAGICLHVELLSGRDPHHIVEAQFKALALALREAVALDPRVSGVPSTKGSL

gnl|extdb|pgaptmp_001356	K02337	dnaE; DNA polymerase III subunit alpha [EC:2.7.7.7]	MASDSFVHLHNHTEYSMLDGASRIKPMVALAVRLGQPALGITDHGYLFGAYEFYRECTAQGIKPIIGLEAYITPGTSRFDRHRQLEWGEPWQRDDDVSAGGAYTHLTLLAHSTEGVINLFRLGSEASLDGQMGKWPRADRELIERYHEGITVLTGCPSSAIQTRMRLGQWDEAVREASELRDIFGPENFYVELMDHGIDLERRTRQDLLRLADMFDAPLVVTNDSHYVRKEDSRTQEAMLAINSGSTFLEEPKVRHPDGTVTGSRFVFEGEGYYIRSSEEMRRDWADLPQALNSTLEIAEKSQVHFRTTDEGANYMPNFPTPEGEDETSWFIKEVERGLRRRFPDHIPSEVRERANFESDVIISMGFPGYFLVVADFINWAKEHGIRVGPGRGSGAGSMVAYAMGITELNPLHHGLLFERFLNPERVSMPDFDVDFDERRRDEVIEYVRRKYGDDRVAQVVTYGTIKTKQALKDSARILGRDFQTGERLTKALPPSVMGKDIPVKGIFDESHPRYAEASEFRKLYEESPDLHEVVDMALSLEGLTRQWGVHACAVIMSSHTLTDIIPLMKRPQDGAIITQFDYPTCEALGLLKMDFLGLRNLTVISDALDNIALNGKELPDLNERDLDDRRTYELLSQGDTLGIFQLDGGGMRELLRLMQPDNFEDISAVGALYRPGPMGADSHTNYALRKNGRQEITPIHPELAEPLKDILGTTYGLIVYQEQVMQIAQKVAGYSLGQADMLRRAMGKKKKEILDREFEPFKAGMLERGYSMEAIEELWGILVPFSNYAFNKSHSAAYGLVSYWTAYLKANYPTEYMAALLTSTKENKDRRAIYLAECRHMDITVLTPDVNTSKGNFVADGENIRFGLSSIQNVGNNVVEAIVATREEKGAFESFRDFLDKVPQVVCNKRTIQSLIKAGAFDSLGHTRRALLSISDEAVDSVIDVKRNEAHGQFDLFAGLGGEDEGAAGLAITVPDIPEWDRKQKLAFEREMLGLYVSDHPLRGIAHSLSRYQDQQIAAILDAGDDFDGKQVKVAGLISSLQTRITKNGAPWAIVTVEDLAGSIEVLFFPRQYEKVQHLLAEDALVQVEGRASLRNDAVSITGNNLRVLELREDNNNPVEIRLDPSQCLPAVLQQVRQALLNYPGPQQVRVHLVEGRKTSIIQLDDRLRVAGTSSLISELKAILGPAAVTH
gnl|extdb|pgaptmp_001358	K06180	rluD; 23S rRNA pseudouridine1911/1915/1917 synthase [EC:5.4.99.23]	MSEVRELPVPEGLVGERVDAALSRLLGLSRVQVTDLLAEGRIEVNGQVPTKSARLHSNDWLRVELPSPEEKPTPVVEMQILYDDEDLVVVNKPVGVAAHTGPGWDGPTVLGNLEAAGFRIASGGPPERQGIVQRLDVGTTGAMMVAKSERAYSVLKQAFRDRTVDKRYHAIISGHPQNLHGTVDAPIGRHPSRQWRMAVLEGGKPARTHYDVIELMPGAALLDIGLETGRTHQIRVHMAALGHPIVGDTFYGADPVQAERLGLTRQWLHAYSVGFTHPRTEEYLEIQAPYPQDLVDALAQLRGES
gnl|extdb|pgaptmp_001359	K03101	lspA; signal peptidase II [EC:3.4.23.36]	MAEQEAERTESTGKHLSMGWVMLGVTAVAIILDQLTKIWAVTSLPEDGSAPYSFGFISLRLIRNPGAAFSLGAGSTWVFTIISTLVVIAILWWVAKGNVQSVLLAVVLGLIAGGAIGNLIDRLTQPPGFAQGHVIDFIDYNGFFVGNVADIWIVGGALGLVLYFALHRETPEVTDE
gnl|extdb|pgaptmp_001360			MALLTAEDVLEKKFQVVKFREGYDQVEVDEFLDEVVATVYALQVENSELKDQLEAANRRIAELSSGHPVEVAPAPVVEEPVVAAQPEPAPVAAPSQEDHASATSMLALAQRLHDEYVNDGRAEGERIISEARQRGDEIVREAEGRRDSVLTQLDQDRGLLEEKINELRNFESQYRRSLTDHLQTLLQEVSSEN
gnl|extdb|pgaptmp_001361			MLIILGTILSFLASAYTLILLARVVLDWARILAPRWRPTGPLLVLANFVYAVTDPPLRWLRQYIPPLRLGNIALDVGFLVLFVAVALLGRLGPVLIMMGR
gnl|extdb|pgaptmp_001362	K09772	sepF; cell division inhibitor SepF	MAAGLNWALEKMGWRVPEDELIEDDFAPVAEVHEYPFSAFEQTVQEEAPVKTSIERDSYLSESAAQDDRRRIVTVHPRSFSDARVVAESFRDGIPVIMNLTDMDDSEARRIIDFAAGLTFGLRGEIERVTTRVFLLSPEDTVVSNSRVGRTASYR
gnl|extdb|pgaptmp_001363	K03531	ftsZ; cell division protein FtsZ	MATPQNHLAVIKVAGVGGGGVNAVNRMIEVGLKGVEFIAINTDAQALLMSDAETKLDIGRDLTHGLGAGADPNVGRKAAEDHVEEIAESLQGADMVFVTAGEGGGTGTGGAPVVARVAREAGALTVGVVTRPFSFEGNRRSAQAETGITDLAKEVDTLIVIPNDRLLEISDRNISVLDAFRAADQVLLSGVQGITELITTPGIINVDFNDVKSVMQDAGSALMGMGAATGEDRALRAVETAVSSPLLEASIDGAHGVLVFIQGGSNLGLHEVSEAVTLVQQASHPSANIIFGHVYDDSLGDEFRVTVIAAGFDAQKAAEAGLPAAASKPIQEVAPEVSAPLAAPVATPAPAPEVSAPREAAPTATAPIAAPAHRAEPQVPTRTGPIRIRPATEPTPAVKRSESFVEPR
gnl|extdb|pgaptmp_001364	K03589	ftsQ; cell division protein FtsQ	MSQAPAKVAQPAPTGAVATAAEREGLAKKWQQDQPSQLQERQQEKKRARRHLRLVNLVRVAAFAALVGLIVWVVGFSPLLAVRADHVEVSAQSGEVNTAPVEARVSQEVGTPLARVNTRQLQSDILADPSISTAQVHRVWPNGLSVGLTPRQPVMAVHVGSAYQRMGPDGVVIDEVAEPPAGLILVEAGGKALGETQVDLVLALWQVLPEAIRARVVAIELAGTNFTLTLDGGAKVIWGDSSQAELKGQVLGLLLGQREASVYDVSTPDRPSIK
gnl|extdb|pgaptmp_001365	K01924	murC; UDP-N-acetylmuramate--alanine ligase [EC:6.3.2.8]	MSEEYYFIGIGGAGMSVIAELLWQEGYRVSGSDRQDSAVLDRLRARGITAHASHDAHQVPPEAVVVVSSAIRETNPELAQARARGQQVLHRSEALALAARGKRFVAVAGAHGKTSTSALLTQALVACGADPSCALGGPILGLDSGALLGQGDIFVAEADESDGSFLNYRPTVALVTNIEADHLDHFGSIEAFEGIFFEFAQRIVPGGALICCAEDPGSARLAARARTELTELTVLTYGRAEYAPDLVLTDDWANASGSGGTVTVAAEPELRADLNLQVTGAHNVLNATGAWGAGLALGFEPTQFAEALGAFRGAGRRFETKGVVAGRRVIVDYAHHPTEIEAALKQARLVAGTGRVVVVFQPHLYSRTLNFADRFARILAEADRVVLADIYAAREDPMPGVSSQLVVDRLLQAGVDVEYRAGATVNESALLGAELCAPGDLLLLVGAGDINQGADAVLDYWERV

gnl|extdb|pgaptmp_001366	K02563	murG; UDP-N-acetylglucosamine--N-acetylmuramyl-(pentapeptide) pyrophosphoryl-undecaprenol N-acetylglucosamine transferase [EC:2.4.1.227]	MTKRRVLLAGGGTAGHVNPLLAVGTRLRDLGWDVQVLGTAEGLESQLVPAAGFPLTVVPKVPLPRKPSPQLLTLPGRLVHAREVASEALSGAEVVIGFGGYVSVPAYWAARSAKVPVVIHEQNARPGLANRWAARFAAAVAVTFAGTDLRARRGLTETTGLPLRAPIAALVAKRATPTGLAEARARGAEALGLDPDRPTLLVTGGSLGAQHLNETMVQAAEMLPAEAQVLHLTGRGKDGPVREHLPASVRDRWQVRDYLATMEDALAVADLVVCRSGAGTVAELSALGVPAIYVPLPIGNGEQRLNAADVVAAGGAELFEDAAFSQAVVTSTVFPLLADAERRARMGEAARNSSVGDGTEAVINLALKVAKA
gnl|extdb|pgaptmp_001367	K03588	ftsW, spoVE; cell division protein FtsW	MAVNLRVPKIKFARANSSDSTYSPIATYYMILVAALMLAGFGVIMTFSATAVHNISMELNPYLVFSRNVVITLVSLIAAVAASRFRPTTIRRWSWALFLAALVFQLMVVPFGYAQGGNQNWVRIPVINQMIQPSELLKLATCLMLAQVLANLGERVNNWKAVAVGVGLPAVASLGAVMLGHDMGTALIFLAITVGALWVAGAPLKWFGAIGLAGLAASAVLVMMNPSRLRRVMEILPGLGSAPDPSAPTQTAQGLWALGSGGLIGLGPGASRAKWNYLQEAESDFILAIVGEEFGLIGTLTLVVTLGVLVWGTLRLASHSPDAFVRIASGGIAAWLIFQGFVNIGSVTGLTPVIGVPFPLVSYGGSAFLFTALAIGVLLAFARSEAGLTGWWRNRAETGPRDPRVA
gnl|extdb|pgaptmp_001368	K01925	murD; UDP-N-acetylmuramoylalanine--D-glutamate ligase [EC:6.3.2.9]	MPGKIAVLGAGVSGLAAAAALRARGIQTAVFDQQSPEADVVVADGHLLGRAVVEWAPEAVVTSPGIAPHTPLLRTVRAAGLPVWSEVELAWRLQEDSPRAGRPWLAITGTNGKTTTVGLLTAMLAANGEAVAEVGNVGLPITSQVDSEATVFALEISSFQLETTESMAPEAAICLNVESDHLDWHGSEQAYREAKARVYHRAGVSCYFADDPVVAELVPAGALALRCGEPGPGEIGVSEGWVVDRRSDPVRLVELRLIPFYQDRRCPPALLQDIVAAVALARVHGVEPEAIAAGLAAFEPAAHRGAVVAVQDGVTYIDDSKATNAHAAAAALAGLAPLSAVWIAGGDAKGQDFTHLVRQVADRVRVAILIGVDREPFRRAFAQVSPKTPIVEVMGEGTPAQWMAQVVDHARQWAEPGDTVVLAPGCASWDQFNSYGERGDCFAAAVRAER
gnl|extdb|pgaptmp_001369	K01000	mraY; phospho-N-acetylmuramoyl-pentapeptide-transferase [EC:2.7.8.13]	MIGVIISFGVAMLLSLLLTPVFIKFLVRREYGQFIRQDGPTGHFTKRGTPTMGGVVFIFAILVGWLVGYLSVGSWPDYSSLLLAGLLFGLGVIGFLDDYIKISRHRSLGLNPLAKIIGQILIGSAFAVLAVWQPNSAGVTPASMTISFIRPSNLNLAFAGFGLGVFLFILWANLLVSAWSNAVNLTDGLDGLATGASIAAFGGYTLIALWQFSQSCSRAVTNTVGCYETRDPLGLAIFGAAVVGALIGFLWWNASPAQIFMGDTGALALGGAFAGMSIFTGTEVLAIVIGGLFFIITMSDVIQILVFKRTGKRVFRMAPLHHHFELKGWQEVTIVIRFWIIAGLFALVGVGLFYGEWAATI
gnl|extdb|pgaptmp_001370	K01929	murF; UDP-N-acetylmuramoyl-tripeptide--D-alanyl-D-alanine ligase [EC:6.3.2.10]	MKRSSAWVAQAVGGQLHGPDRPVTASVETDSRACTPGSVYVARRGDQADGHDYAPAAIAAGAVCLVVERLLDLDVAQVLVTDSTQALGQLARSYLAELRAAGPISVVGITGSAGKTTTKDLLGQLLSSQAPTVYPVASFNNEVGCPLTVLRADEETRYLVLEMGASGRGHLTYLTSLAPLDVAVELMVGQAHLGGFGSVEQLAASKEELVEGLVEDGIAVLNADDPRVAAMAGAAPGRVLRFSRLGQSADLWAEAVALDELGHPSFQLCTSDQRQPVSLQLVGIHQVSNALAAAGAALAVGLDLPTIARELSGAGALSAHRMDVRLDRQWRGQDLLTVIDDAYNANPDSMTSGLITARLIAGSHRLVAVLGEMLELGPESAALHRQVGSRADVDVLIGVGEGSRDLLESSPAAVKHWVSDANEALDVLATTIASGDTVFLKGSYGSGVWRVADDLLAADTMEAQP
gnl|extdb|pgaptmp_001371	K03587	ftsI; cell division protein FtsI (penicillin-binding protein 3) [EC:3.4.16.4]	MKEAPPTVEGQTVPDPRQHKSRQRSRWFGALTVLALTVCAIQLFSIQIIRGPALAEQGRKVRTSATEVSAPRGKIVDASGQTLVDSVETYHIAVNQKNILEWKHRDEDGKLIGQGPADAAEQLAPLLKMDPAELGGMMLGDSTYAYLAKNVDAATFRKIKALGIYGIEWEPVYQRAYPGGNTAASIIGSVDANGLGNSGLELVYDDVLTGIPGEESFEIGPTGEVIPGAKTVSKEARTGGTVHTTIDADLQNSVQEYLDQAVKTYEADWGSVVVLDVATSRVLAMGDSGLQSPAKGPQPSGSVQWVIEPGSVGKILTVATALEQGTVTPDTVFSVPDRYETKDGEVITDIHEHETYQRTVAGIITESSNTGAVQIGETVTDQARHETMSKLGLGKLTGIELPGESPGILAPAEDWLGRSIYTTMFGQGYAMTPLQEAAMMAAIGNGGVWQGPRLVSGTTDANGKFHAEETPEPVQAIQPETAQILLKMMEGVSTDAQIGTGTAAAVEGYRVAIKTGTSELPSGGTVATVAGVLPADKPQLAIAVVLNNPRSGYLSSDSAAPLFHQVASAAVTSLGIPGSTGPVELYPTAP
gnl|extdb|pgaptmp_001373	K03438	mraW, rsmH; 16S rRNA (cytosine1402-N4)-methyltransferase [EC:2.1.1.199]	MEQQQDARDLHVPVLRDTCVELLLPAFQVSDPVAIDGTLGMGGHAEALLSAQPNLRLIGIDRDRQALQLAGDRLARFGDRFVPFHGEYDRVDEVAREFGRRGQVDGILLDLGVSSLQLDDPERGFAYSRPAPLDMRMDQSSGQSAAELLATAPVGELTRILREYGEEKFARKIAQAIVRRRETEMLTTTAELAELVKDVIPAPARRTGGNPAKRTFQALRIAVNDELGILERTLPRALDSLRVGGRLVVESYQSLEDRIVKRTLAQGLKDTAPPGLPVVPEANRARLRALVKGALKADPTEVAQNPRSQSVRLRAVELVAPWSQS

gnl|extdb|pgaptm<br>p_001374	K03925	mraZ; transcriptional<br>regulator MraZ	MFLGTYEPKLDDKGRLILPAKYREQLAGGLVITRGQERCLYVFPAAEFQEIFQKIRQAPLSSKQARDYTRLMLSGASDEIPDKQGRVTIPVALRQYAGLDRDLT<br>VIGVGSRAEIWDTASWTEYLQRQEDEFAATKTEVIPDMF
gnl|extdb|pgaptm<br>p_001375			MALSEYEKQVLAQMEAQLKEADPSLASVMTSSLPEETDVVPTGRLSPRRIALGSIIGVGGLAIIIAGVSFGYGWVAALLGVLGFACMVGGVLLALKSDPVGDA<br>DGSRADSGSKRKRKDKDFMSRQRSKWDERGPRAQ
gnl|extdb|pgaptm<br>p_001376	K02346	dinB; DNA polymerase IV<br>[EC:2.7.7.7]	MSTAPRSDRAKRDWGDDDSATTILHVDMDSFFASVEVVENPSLAGVPLIVGGVGNRGVVTSCTYDVRALGVRAGMPIGRARALAPQAVVVPGSRGLYRD<br>YSHRVMEILSAITPDFEPISIDEAFLDVAGARRRLGQPREIAQLIRNQIRERVSLPASVGIGNSKTVAKIASSHAKPDGVLLIPAERTVEFLQSLPVGALPGIGRKT<br>QELLDRRGLSTVGQLAELSVQQLGRIVGEAHAYDLHRVAWGEDRRRVGGRAPEKSISTEETFPVNLTERKAVERYLLAAAHDCAARLRDAELVAWTVQIKLR<br>DADFRTITRAQTLTAPTDLGREVAAAALRLFAVERLGRGGVRLAGVGVTGLRPRDEGVQIGLDEDLRPLAAERTMDRVHSKFGRKSLQPATLLGETASLPREK
gnl|extdb|pgaptm<br>p_001377			MSVPAPEIRFDRHGSIASVFIDGVEQSRVDLEHPDQLQFEYMEQMDVVLNLAHPAPTPIKAAHIGGCGCALAWAWQVQRPDSRQLAVEVDEYLATQART<br>WFPLPRKPLLRIRAGEGREVLSSSQAHFQVIVRDAFASRTVPAHLQTVEWDRVVSAHLAPTGLYLANAAHGGGSNARGDVAAARQEFGQVVLIGESKVLKG<br>ARWGNLVLAAWQESQLIDVAELDRRLRRLPLPVQVLSGAELDRWLGGHPPATD
gnl|extdb|pgaptm<br>p_001378			METEKLYTDGPLAGAKAPIMITHFDGAMDAGSTGALSVVQLLSNLSPRRVATFDSDRLIDYRSHRPMMMVENWVTREVVAPEIALDLVHDDTGTPVLILH<br>GPEPDSRWETFTQNVAELARKAGVEVMVSLYGFPAAVPHTRPVSVHVQSTDEDLVPQQGSLLGSWQLPAPLSHFLQYRLSGQNLHGITLYAAVPYYMAEG<br>AFPRGASALLRRLSEMTDLSLPIGDLEQGADADADQVDTLVAQNPEVLRTIEALEVHYDQMAAAPNSPLSSNSTALLSMPAPDEPKDKDSESVAEALGDAIE<br>KYLRAQTKTEEGEGSPEELDASDEAEPGPKHAAPRAWEQDRGLED
gnl|extdb|pgaptm<br>p_001380	K00058	serA, PHGDH; D-3-<br>phosphoglycerate<br>dehydrogenase / 2-<br>oxoglutarate reductase<br>[EC:1.1.1.95 1.1.1.399]	MTTALLLENPDACADDIFASEGIEVRRISGSLDEAELIEALQGVQILGIRSKTEVTARVIEAAPNLLAIGAYCIGTNQIDLEAASRHGVAVFNAPYSNTRSVVELAI<br>AEIIMMGRRLVERNNSLHQGVWNKSASHAHEIRGRTLGIIGYGSIGTQLSVLAEAMGLKVIFYDRAERLALGNARRMHSMEEVLQQADVVSMHVSGEASN<br>TGLIGDREFSMMKPRSLFINLSRGHVVDLDALRKHLLTGHIAGAAVDVFPHEPKSTGDPFESPLADIPNVILTPHIGGSTVEAQEDIARFVSGKLSAYLGDAGTY<br>MSVNFPSVSMPRSEGATHRLALMHFNVPGVMGKLNRGFAKLGVNVVGQSLGTKGEYGYALTDISGPLPEARLGELAAMESTIRLRYLDLG
gnl|extdb|pgaptm<br>p_001381	K01897	ACSL, fadD; long-chain<br>acyl-CoA synthetase<br>[EC:6.2.1.3]	MTLTEKLRKNYAPGVPFEIGIPDTRLHDYLLGAASRYPQRVALDFMSKQTTYRQLEDQVRRAATVLAGAGVKPGDRVALVLPNCPQHIVAIFATSLLGAIIVE<br>HNPLAPEEELRSEFERHGATVVVAWENSVEKLGFLGPTATVFGVNLADDIPRTSRVLLKLPLPSIRAKRDLLGAKTPGYVRPWAATVAKAQPRPGTGPAHPD<br>DTALLIHTGGTTGVPKAVALTHRNICVNAVQDVAWVPPLHEGAEVFYAVLPFFHAYGFGISLMAAVRLGATIAVFPKFDPVQICLSQRRLPCTFFIGVPPIYER<br>LLKSARELEISLESITYAISGAMALDPKLAAEWEAATGGYLVEGYGMSEASPTLLGSPLSPKRRPSTLGIPYPSTEVRIVDPEQTEVDVADGEVGELIVRGPQVF<br>NGYWNDPEETATVLKDGWLHTGDLVKVVDGFIVMADRRKELIISGGFNIYPSQVEAAVRSMPGISDVAVVGMPGGTRGEDVVAALVLEAGASLTLADVR<br>QWAEKSLAHYALPRQIVVLQELPKSQIGKVMRKRVREQLAELQSGITDVQAGLGERLSNLATQVSEMAEHASAAVSHTLRKDKEPASTPELPEENPAPDAA
gnl|extdb|pgaptm<br>p_001384	K01356	lexA; repressor LexA<br>[EC:3.4.21.88]	MAKSANVPEIRVSKRQQQILDFVRQYGKEVGYAPSVREIGRAVGLASPSTVKHHLDYLSRVGLLQRRDRSPRALIVDSGPSRKKSSPTPPTSAVSPTVIEVPVTI<br>SEGETTEVPLVGRIAAGAPILAEQAVEEFFALPTRFTGSGQLFALEVHGDSMIDEGILDGDYVIVRAQATAQDGEVVAAMIDDEATVKVLSQTAGHTWLLPR<br>NSNYAPIFGDHAQILGKVVTVIRAV
gnl|extdb|pgaptm<br>p_001387	K03665	hflX; GTPase	MSADDQSLDRAAEIAQRVLARQGTALESTATQGDEGEGGELEREARAGTRRTTSHSTELEDVSEVEYRQVRLERVVLVGLRQEGSLAEAEASLRELQALTET<br>AGAQVVDSVLQSRPLPDAATYLGKGKAKELADLVRDVEADTVIVDGELSPSQRRGLEDVVRVKVVDRTALILDIFAQHAKSREGKAQVELAQLEYLLPRLRG<br>WGESMSRQAGGRVAGGAGIGSRGPGETKIELDRRRIRTRMARLRRQLLQMAPARYNRRAHRRRGQVPSVTIVGYTNAGKSTLLNRLTNADVLVENALFAT<br>LDPTVRRTTTPDGRVYTLTDTVGFVRNLPTHLVEAFRSTLEEAGEADLLLHVVDAAHPDPEGQVRAVRAVLAEIDGAEAIPEMIVLNKCDLATPERLAALRAT<br>FPQAVQVSAATGKGLDQLREAIAQALPRPQLLLDVVLPFSQAKLVSLTHEEGEVIEEEWTEDGIHLVARVDEPLAQKIRAHS
gnl|extdb|pgaptm<br>p_001388			MTEHYYSEAPLPTEDELRLLPVSVRGRDLRMWVSSHVFSTGRLDPGTRELLRALPELPAEGTFLDLGCGWGPVAVTAALESPAAKVWAVDVNPRALELTRR<br>NARENGAENVHAALADEAWKQSADVRFDRILSNPPVRIGKAAVQELVDRWLSRLAPTGEAWLVMGKNLGGDSLLKWLQGRGYEAVKHSSRKGYRIIRVV

gnl|extdb|pgaptmp_001389	K00791	miaA, TRIT1; tRNA dimethylallyltransferase [EC:2.5.1.75]	MTGAQMIAVVGQTASGKTDLGLLLAERLGGTVVNADSMQLYRGLEIGTAKTPVDQRRGIPHLLFDVLEIEQEASVARYQQEARQAVSQLLAEGRTPIVVGGSGLYVRALLDRFEFPGTDPEVRSRLEERCQQEGPGALHAELSRVDPAAAAQIHPHNARRIIRALEVIELTGAPYSAQLPDGKYFFEPTVQVAIRWDYDQLDRRINERTREMFADGGIVEETRALLDSGRHFGKTAARATGYAQALAVLRSELTVPEAIEQVSLLTRQLARRQMKWFKRDERIRWLNPGQLPG
gnl|extdb|pgaptmp_001392	K06168	miaB; tRNA-2-methylthio-N6-dimethylallyladenosine synthase [EC:2.8.4.3]	MNKTPRTYAVRTLGCQMNEHDSERIAGLLEEAGLIPVESVPTAAARATDAGDLGADVIVLNTCSVRENAANRLFGNLGQLAAVKRERPGMQIAVGGCLAQQMREGIVERAPWVDAVFGTHNIDVLPTLLARAAHNEKAAVEIEESLKVFPSTLPTRRESVYAAWVSISVGCNNTCTFCIVPRLRGREQDRRPGEILAEIDAVVREGAIEVTLLGQNVNSYGVSFGQRGAFADLLRAAGATPGLERLRFTSPHPAAFTDDVIAAMAQTPTVMPSLHMPLQSGSDRVLREMRRSYRRARFERLIEKVRDQMPEAAITTDIIVGFPGETDADFEETMDVVRSVRFSSAFTFQYSPRPGTPAAGRSDLIPADVMAERYQRLLDLQNQISLEENQAQVGRTVKVLVAQGEGKKDGQTRRVSGRAADNRLVHVALPDGLEPRPGDLVVAEVTYGAPHYLLADAAQVRSTRAGDAWEARQQEEDTSVVNLGMPTLKYRGQ
gnl|extdb|pgaptmp_001393	K03743	pncC; nicotinamide-nucleotide amidase [EC:3.5.1.42]	MATDRAAKLVAGLRRAHLHLAVAESLTGGQLCATIVEVPGASDVLRGGVCTYAPDTKVEILGVSAELIAERGTVNQEVAQQLAAGARRLFGSEVALATTGVAGPGPAEGHPAGTVYVAIDAPGHQTWTAHQFAGDRQQVRNQSVEAALRLLEDYLAKHYNADDE
gnl|extdb|pgaptmp_001394	K03565	recX; regulatory protein	MATAWDSALRYLGQRAHSRQELIQKLTRRGYDQEEIDQTLMRLDQADLLDDAQFARDYTRSLLSSRGSSRREVQRELGKKGISSDLVADVLADLPDDFDRALAGARKKMAGLADLEKDVRWRRTLAYLARRGFPEATCYAAVRAAEEEHNGN
gnl|extdb|pgaptmp_001395	K03553	recA; recombination protein RecA	MAREKTLRKSTSTNPDRDKALELALTQIDRQFGKGSIMRLGDDTRPPVKVIPTGSLAMDVALGIGGLPRGRIVEIYGPESSGKTTVALHAVASVQRDGGNAAFIDAEHALDPVYAQALGVNTDDLLVSQPDTGEQALEITDMLVRSGGIDLIVIDSVAALVPKAEIEGDMGDSHVGLQARLMSQALRKITGALSATGTTAIFINQLREKIGVFFGSPETTTGGKALKFYSSVRIDVRRIETLKEAGQPVGNRTRAKIVKNKMAPPFKQAEFDIIYGRGISREGSIIDMGVECGVVRKSGSWFTYGKDQLGQGKENVRQFLVDNPELADEIERQILTELGIGTAPAEEEQAAPVSEEPTEASA
gnl|extdb|pgaptmp_001396			MRRSEFWEALELAFGSGPGRSLAADLHLLAVNGTAEEALAAGVAPDRVWVALIEESGADPALRWVHRRPRRKSGVSDGGEPLF
gnl|extdb|pgaptmp_001398	K00995	pgsA, PGS1; CDP-diacylglycerol---glycerol-3-phosphate 3-phosphatidyltransferase [EC:2.7.8.5]	MNQQVSNINLPNALTVLRIILVPVFAWVYLLGAHWWALAIFLFAAVTDQLDGHLARKWNLITDFGRLADPLADKALTLTAFVLLSIGYQSPWMWAFTILVAIRELGITVLREVLRHRGTVVAASSGGKLKTVLQILLIILMLIPWGNLAVINWIIVGLAVVTFAITMISGWQYLWAAWRGSRP
gnl|extdb|pgaptmp_001400			MSAQLPPAPLTPVGPALGRFITTESVVLAVNVQLSKLPERGAATTAYSAISSAGGGFTLLSVVAAQGIDTNLAAPLGTGPNSSLARRQLAAARINTLTDVFVGDIGVAMVFVEDDGNNTTVRTPGVESEPTLAGLEAIELHPGDLVHISGTDLATPQSDVVVEWGSRLPDDVTMVLAISPAVQEVSTDVWAQLLPRADVVTMNIREAAYLSRFLDQSSPGTGIRHIMRPEAAVVRRLGVMGCEVQATRDADMVNIPSYQSYRVDTTGVGDTHVAAMCAGLLQGLDLVGACQRANAAAALMVARGSTPQAPTAAEIDGVIQRGTVL
gnl|extdb|pgaptmp_001401	K12574	rnj; ribonuclease J [EC:3.1.-.-]	MRSIFENLPEPPPVPSGALRVLPLGGLGEVGRNMNVLETEGKLLVVDCGVLFPEEHQPGVDLILPDMSAIADRLDDIVALVLTHGHEDHIGAVPYLLKLRPDIPIYGSELTLAFLEPKLKEHRLSASGLHVVAEGDRIKLAPFDAEFVSVTHSIPDALAVFVRTKAGTVLITGDFKMDQLPLDRRLTDLRSFARFGEEGVDLFMVDSTNAEVPGFITAEVEIGPVLEQVFAQTDGAIIVASFASHVHRVQQVINAAQQCGRTVALVGRSMERNMTIARERGYLQIPDGAVAELKTVEKLPRDRQVFMVTGSQGEPMAALARIAGGTHQSISAGPGDTVVFASSLIPGNENSVNRVINELMGLGAKVVHRGNAKVHVSGHASAGELIYCYNIVQPKNVMPIHGEVRHLVANGQLAVKTGVDPTRVVLADDGVAIDLKDGRAVIAGAVPCELVYVDGKSVGEISEDELRERITLGSEGFISIFAVVDKEMGTVLAGPVLRAVGMAEDDSVFDDILPEVDKALRDAAAPGGVSARTLQQVMRRVVGRWVSRRLRRRPMLVPVVVEQ
gnl|extdb|pgaptmp_001402			MRTLNHNFVRPAQPEHAQFIAGLQVQAMAEVLNAELGPDAPVDQLDQEAIAAAWSQTIAQPERARHGVFVATASDQPEGYVAFGDGAERSPLAEDGVAVEILGLEVVPDQRRQGHGSRMLAAAADLVRDEGGDYLQVWLAPAEEEKIRFFQSAGFAPAGLRRTLDVMGRPLTQHLWFATLDRENPAPTS

gnl|extdb|pgaptmp_001403			MPDSSLDPVKLPLASAARSDVLEMPDGPLTVRRSILPGGVRVITEELPGTQTTTLGLWVATGSRDEQESEAGASHFLEHLLFKGTPTRSAFDIAAAFDEVGGESNAATSKETTHYWAKTLDSDADMTLATLTDMVTSSLITDEDVNTERTVIIDELAMAEDSPADVVHEAFATALFGDDPLGRPIGGTTASVSDLPAETIRELYRRHYHSRNLIVAGAGHLDHEQICAGLSQALALTDWDRDEAAAPASRDLLAGTGSEPPLEVVDRREVEQAHILVGSRWLNATDEQRPTSNVLLTILGGGMSSRLFQEIRERRGLAYTTYAFDSAYLDTGYFGLYAGCAPENVDEVERIMWGEVEKLAEAGVPEAELRRAKGQLRGNLALGLESSASRMMRLGRSEVTERFISVDAALARIEAVQVEEIRQMAEQMLAGPRARALVTNR
gnl|extdb|pgaptmp_001405	K00962	pnp, PNPT1; polyribonucleotide nucleotidyltransferase [EC:2.7.7.8]	MEAENLVSAEAIIDNGRFGTRTIRFETGQFAQQAAGSAVVYLDGETMVLSATTVGKHPKDQFDFFPLTVDVEERQYAAGRIPGSFFRREGRPGTEAILACRLIDRPLRPAFVKGLRNEVQVVETILAVHPNDAYDVVAINAASMSTQIAGLPFSGPIGGTRVALIDGQWVAFPRWSEMENSVFNMVVAGRLITDENGNEDVAIMMVEAGGGETQWDLIQAGAIKPTEDVVADGLEAAKPFIKVLCEAQKEVAAKASKETAEFPLYFDYTDEELAAVRDKVGDRLVAAIATEGKLAREQAVDEVRDLVIGSLVDEFPDSEKALKAAFRSVEKETIRQRTLRDEIRMDGRGPRQIRTLMAEVEVIPRVHGSALFQRGETQILGVTTLAMLRMEQQLDNLSPASHKRYMHNYNFPPFSTGETGRVGSPKRREIGHGDLAERALLPVLPSREEFPYAIRQVSEALGSNGSTSMGSVCASTLSLLQAGVPLRAPVAGIAMGLMTGEVDGQPKAITLTDILGAEDGFGDMDFKVAGTAEFITALQLDTKLDGIDSQLLRAALAQARDARLEILDFMAKAISTPDEMSAHAPRILTVQVPVDKIGEVIGPKGKMINQIQEQTGADITIEDDGTVYVGATNGESAEAARALINQIANPQMPEVGERFVGTVVKTTSFGAFVSLTPGKDGLLHISQVRRLVGGKRIDSVEDVLQVGQQVEVEISEIDERGKLSLSAVVDEEAAGEAEEAPRNSEKKEERSGDRRPRQRNRRRRNNGDESAE
gnl|extdb|pgaptmp_001406	K02956	RP-S15, MRPS15, rpsO; small subunit ribosomal protein S15	MPLSKEQKDQIIAEYATHEGDTGSPEVQVALLSARINELTEHFKTHKHDHHSRRGLLLLVGRRRNLMKYLESIDVDRYRSLRDRLGLRR
gnl|extdb|pgaptmp_001408	K11753	ribF; riboflavin kinase / FMN adenylyltransferase [EC:2.7.1.26 2.7.7.2]	MKIWTSLDQVPGDQKSVVTIGNFDGMHLGHARVIGSCVDRAKKRGCESVALTFDPHPRSVHQPGRPVPLIMTTQDRLDAMAVTGLDATLLAHYDRSLYSLSAEDFVQEYLVERLGAVEVVVGEDFRFGQGNAGTVDTLRQLGRMFGFDVTMVTDVVSPGGRRWSSSWVRELLQLGNVEEAASVLGRFHRVSGLVVHGAKRGRELGFPTANVSAPGQLLPADGVYAGWLVREAELPGQDRWGGGQEFLPAAISVGTNPQFGGTNRTVEAHVLGRSDLDLYDEEVTVVFVRRLRPMLSFASVSDLLAQMDEDLRHTAQTLGVRTARRVDPEAVTAGA
gnl|extdb|pgaptmp_001410	K03177	truB, PUS4, TRUB1; tRNA pseudouridine55 synthase [EC:5.4.99.25]	MSSAGILLVDKPAGLTSHDVVGKVRRLARTRAVGHAGTLDPAATGLLILGVNRGTKLLTYLTGLDKTYRATIRLGADTTTDDAEGEVLSAPGCPPLAADVLEAALAQFRGRIQQVPASVSAIKVDGKRAHALVRAGQQVELAARPVTILRLEQLGPARVVDQFLDLDVEVECSSGTYIRSLARDLGRVLGVGGHLTSLRRTTIGPWTVAEATPLADLPPDPTLLSFDQVCPTLFPLVEITPAQAERFRYGQVPELDVTLAEGEVRSIGVPGANVAGLVQVKSGRLKPAFIIQPI
gnl|extdb|pgaptmp_001411	K02834	rbfA; ribosome-binding factor A	MADESRVRRVQENIKMTVARTLERRVKDPRLGFITITEVKATRDLQHATIFYTVLGDEKAARDTRKALESAKGLIRSEVGKALGLRLTPTISFEADRLPDEAASIEEALRAARERDAEIARHSADKEYVAGEDPYREEPVESDDDE
gnl|extdb|pgaptmp_001414	K02600	nusA; transcription termination/antitermination protein NusA	MEIDMTALRTVAEELGVSPNALVDAIEEALLRAYHNLPGAITPARIELDQKSGKAIVWAADEDEDGNQIGEFDDTPSNFGRIATATARSIIAQRLREAEDSRVLGSFAGKEGTVVTGVVQQARDRNLTLVRIGDEFEAILPEGEKVPGERYEHGDRIRAFVVSVDRGDRGPRILLSRTHPGLVEGLFRREVPEIQEGLVQIKAIAREAGHRTKIAVAATRDGINAKGACIGPMGGRVRNVMAELGGEKIDIVDWSADSARFVANSLSPARVSRVVVHSETNRTATAIVPDFQLSLAIGKEGQNARLAARLTNFHIDIHADTDTAEGEELGVSRSSVPDLSAEVSPDGKAVIIEGDQTEVIGD
gnl|extdb|pgaptmp_001415	K09748	rimP; ribosome maturation factor RimP	MEAEAVEAQVLALLQPLAVTADAVVESVRLVQRGEESVLEVTVDRAEGTASLPLDDVAELSRSFSEALDAADPLTGTYTLEVGTPGAESELKALRHYQRNLGRTIRVKTVDGEKLEGQLTAVGEDSFTLRLPDGEREITFDEVRKARPRVTFG
gnl|extdb|pgaptmp_000391			MQACDPESQKKILNRLRRAQGQLNAVINAVDADSSCRDVVTQLAAVSRALDRAGFAIIATAMRACMNEADEEGGDDRLTPEELEKLFLTLS

gnl|extdb|pgaptmp_001436	K02316	dnaG; DNA primase [EC:2.7.7.101]	MAGLIRREDIEEVRNRTRIEEVVGEQVTLRPAGVGSLKGLCPFHEERTPSFHVRPQLGLWHCFGCGEGGDAIAFVQRINHLDFAEAVEFLAEKVGVTLRYEGGGNRRRTEEPGRRQRLLEAHRNAEDFYREQLQTPGAAVGRQFLAERGFDQAAADRFGVGYSPASWDGLTRHLRGRGFTDQELITAGLAVQGNRGLYDRFRNRLMWPIRDLTGATIGFGARKLDEEPDSPKYLNTPETPIYRKSHVLYGLDLAKQDIVRQRRIVVVEGYTDVMAAHLAGETVAVATCGTAFGSDHVQLVRRLLGDEADPAAGVVLTSGRARGGEIIFTFDGDEAGKAAARKVYVEDQKFAAQTFVAIEPHGWDPCDLRQRRGDEAVRQLIESRVPLFEFVLRSVLAQLDLDTAEGRVAAVRAGAPVLAGIKDIALRQEYVRQFAGWVGVEMSRVSGAIRNVGRPRPAAGIASPAVPSNDPVVRMERQALEALLQRPLDVVGMGFELLSPESFSLPIHRAVHEAILAAGGLDRYLDFLAAAEQEVGMGEQSVSLATRRWIEEIRSGAGPQVSEVIRQLAVAPLAQDDASSVRDYAVGLMRALVRLELTRRVAALRLRLGRLDPSDPDYEEIFAELVELDSQRREYADHSG
gnl|extdb|pgaptmp_001438			MIDSNLKETNTSTLTRQIYRGERGVTSWWNTAAVCDLPGAYGPADLLGLIGLLPAIRRMGFSAILLRLSLPDLNPQRDYIAKLVKATHAAQLKILVRVRSDAATVSPQDSPPPVELDNDPDTLIERTRVLMGAGVDGVDLGLIDDDPARPHREDRIRQFTETVNRQLAEVASLDSTVILTAEASVAHPEFFEHHVQEDWFHHLRDDSLLQAPWDASELRKRVAKAYSSRAPLGQTVAWRPAFNPAAEGADARTPNPGSWADGAPIGRVNAFLTFVSSLPGALYLPFVSAGGQVEVEEGSRPQLRLSLANDPQSRYQHDLTTRILTLRERKHLSHSNLALIDDISWAHPGVSVHLSGHIMVVLNASSEPVAVPAKHLPLLYSDGFVFSDATHTVIQPDTCAWFEPAPAN
gnl|extdb|pgaptmp_001439	K03785	aroD; 3-dehydroquinate dehydratase I [EC:4.2.1.10]	MVNPISLGRAGVHPVQLGPGHLVVAVPVLASTSAEAYRQWCHAADRGADLVEWRLDPLLTSGEAERAFAVAPSWREQESCPVLVTVRTAAEGGDWSAVPGEYLDWVERAGTWADAVDIELATPAVGELVDRSHQQGARVVLSRHVLAGSVDPGELQATLEQMVTLGADVVKVAWQVQSRLDVDQILAAQRWAAETLPVPAVIIGMGPAGQATRRGQAARLSAFTFGVGAVESAPGQLSIAELRAADLALGDEGEPSRA
gnl|extdb|pgaptmp_001440			MGPLNLWSPVILAPMAGVTDLPFRRLCREFGERPLDPGQLSTAEAGKDAPAGLYVMEMVTARALVEGNRETWRMLEPDPDERVRSVQLYGTNPATMAEAARLLVDRHLADHLDMNFGCPVPKVTKRGGGAALPWKLDLYRDIVTGVVREADRRGIPVTVKMRIGIDDDHVTVFEAARIAQAAGVVAVGLHARTQEQYYSGHARWEWIARLKEQLKVPVLGNGDIFSAQDAREMMAQTGCDAVVIGRGCQGRPWLFEEIVADLWDRPAPTPPNLGQVCRVIERHADLMVQTLGEELRAMREMRKHLGWYLRGFSVGGATRQQLGLVSTRAELSTILATLNAEEPFPEVAAGPRGRAGKAKRPHLPDGWLSTRTVSEQEKALLHLAEADGGSGG
gnl|extdb|pgaptmp_001441	K01880	GARS, glyS1; glycyl-tRNA synthetase [EC:6.1.1.14]	MANSSRLDAVISLAKRRGFVYPCGEIYGGTRSAWDYGPLGVELKENIKRQWWARNVREREDVVGLDSSIILPRQVWEASGHVQAFTDPLIECQTCHHRFRQDQLIEEFAEKNEVDEATVAMTEISCPDCGTRGNWTEPKAFSGLLKTYLGPVDDESGLHFLRPETAQGIFVNFSNVMTSARKKPPFGIAQIGKSFRNEITPGNFIFRTREFEQMELEFFVEPGTDEEWHQYWIDERLAWYTDLGINPEHLRLYEHPQEKLSHYSKRTVDIEYTFGFQGSLWGELEGIANRTGYDLGVHSEASGAKLDYFDQQANERWTPWVIEPSAGLTRSLMAFLVEAYQEDEAPNTKGGVDKRVVLRLDPRLAPVKVAVLPLSRKEELVGPAKELAASLRKLWNVEYDDAGAVGRRYRRQDEIGTPYCITYDFDSVEDQSVTIRDRDTMEQVRIPVPEVRAWLLDRLDVAR
gnl|extdb|pgaptmp_000812	K09817	znuC; zinc transport system ATP-binding protein [EC:7.2.2.20]	MIPISFHELEVGYGAAPILHGISAEITAGSCVALTGANGSGKSTLLKALLGLIPHQSGQINLFGVELPGGGGPVHQVPWGQIGYVPQRNSVGGGISSSVREIVETGLLGPRRWWRPRGSKAQVDEALELVGLRARQRDIFQQLSGGQQQRTLIARALVRQPQMLLLDEPLTGLDRHNREVLAGVIGAQKAAGHTSLIVLHELGELRPLIDRELRISSGHLVHDGAHLTEEEHHHHEPPRRGVAPGMNLEIK
gnl|extdb|pgaptmp_000811	K09816	znuB; zinc transport system permease protein	MIETLLLRSLLAAVLVGLSAPIVGTYLVQRRLAMLGDGIGHVALTGVALGWLVGTATNLVPVDQLAIPGAILTSIIGAVVIELVRRSGRSSADVALAILFYGGIAGGVLLMGVAGGTSTQLNSYLFGSLATVTWMDITIIGVLAAVVIALGVGLGPALFSVCNDEDFAQTSGLPVRGLSMLIAVMSALTVAISMRVVGALLVSAVMIIPVAIVQLWRRPFRQTMALASILGAVLSASGLWITTSVDLSPGAMIVMLAIGVYALMFVGVGQWRRWHLHHREKVVREA
gnl|extdb|pgaptmp_000810	K03711	fur, zur, furB; Fur family transcriptional regulator, ferric uptake regulator	MQRMTRQRAAVLEQLSQQSDFRSAQQVHDDLHSAGQKVSLATVYRNLQTLVDNQQVDTVRGLDGEILYRLCTEEAHHHHLICRSCGRVEEIRPELLESWVNNLAAEHGFHQPTHFLEIFGLCEQCYSKVPSS
gnl|extdb|pgaptmp_000809			MSVPSWAESGPLLIGFLFVVVFCRAQGTYWLARTLPAAAARTKKTTGWQARLAAWFDGPTPRKGAALLERWGIVIIPLCFLTVGIQTAIIAGAGVVRMNWRRFTLAMIPGAIAWAFLYGLGLLAVWTAAVTALAGNPWSYVALVGIVALFYLLKVVKQRASRRLNQVDCPAKAPLPGTVSQEPLP

gnl|extdb|pgaptmp_000807	K03584	recO; DNA repair protein RecO (recombination protein O)	MLRTYRDQAIVLRSYKLGEADRIVILLGKESGQIRAVAKGIRKPTSRFGGRLDAFNLVDLQLHRGRNLDTVTQVELLSGYARPLGENYEAFTAAKVMVETVQKLTESAPEPAYYPLLHGALGALVAGRIPPDLITASFLFRLMRLAGWDPALHSCAGCGQPGPHRLFSAEVGGFVCSSCAPPGSTEAPPAELIQALLNGDWEQAGQTGPRQGEQVRNLAGWWTQYHLEQRLRSFPFLQKESDEYS
gnl|extdb|pgaptmp_000806	K01649	leuA, IMS; 2-isopropylmalate synthase [EC:2.3.3.13]	MPVSKYRAALDTNPISLPDRTWPDQRLTRAPRWLNTDLRDGNQALVEPMDPGRKRKMFDLLVKMGFKEIEIGFPAASQTDYDFVRALIEDEAVPEDVTVSVLTQSRPELIERTVQSLVGFPRANVHLYNATSPLFREVVFRNDWPATVDLAVSGTREVMAQAEKFLDDGTVFGYQYSPEVFIDTEIDKALEISERVSDVWEVGADRELILNLPATVERTTPNVYADQIEYMCRHLTRREHTTISVHPHNDRGTGVAATELALLAGADRVEGCLFGQGERTGNVDLITVALNLYTQGVDPELDFSDIDRIRQVSEYCTGMRVPDRTPYAGDLVYTSFSGSHQDAINKGFALRQQQIDEGAKESELVWQIPYLPIDPHDVGRSYEAVVRVNSQSGKGGIAYLMSNAHSMELPRRLQVEFSRLVQRHSDTVGGEVDADTLWRIFADAYLPTSAAPGLEPWGRFVLGKTSVTADNDGARLHAELFDQGQQVILSATGNGPIDAFVRALSERGMDIRILDYAEHALTHGEDADAAAYIEVEFDNQVVWGVGIDPSIARASYKAVISALNRALR
gnl|extdb|pgaptmp_000771			MARPIHRTSHRWERLNAAADGAFFLLATAASCWLFVVSLSNIHSFHWTVFAWLILLWATLAYLLLPRIHRVLTTLYVPDYFIGRARTTEGLLGDPVNLAFHGTEAQVEAAMHRAGWVKADPITLRTSLGIALSTLTRKSYPQAPVSPLLLFNRQQDLAFQQEVEGNPAQRHHIRFWQCPPAWPLPGGTRVGWLGAATYDKSVGLSLFTLQVTHKIAENTDQERDHVVQTLQDANPEVKVRVLADFSTAYHSRNGGGDSIKTDGALPIVDLRPVPASKPAEPTHLPSHLDDAKEFLRAERPIPIYVAMLLILLQIGGMVAWSVLALRDSGSYAGAVTKLLPDQGLLAAATPDQIVRTYVVSYLLGAAVFAGLAALMWDGFRPARLFLMAAATLTIVPAFVTWWRLGSSPEFDQYLLSVMMAVPLLMALTSPSVTEWADQRTDLRRARRTARKQVKAGSGK
gnl|extdb|pgaptmp_000803	K03699	tlyC; magnesium and cobalt exporter, CNNM family	MSDLSTIPFVPLLILGLACLALAGFLSAIEISLSSLSRAYVEDLAEDGSKAAVRLSRVLEERPRAVVGLHGARVVSVTVAVLSVTLVTMDLLQPSQLSWWVVALIVLGVMALIEVLMVLVLPWFMVSRNYLSVALLGSRLTLRLLAVTHWFDPLIGRASARLGSERSDAQRLAVAEDLREIADEVGEADSFDEEDKEMIRSVFELGQTRVREVMVPRTSMVTIESDKSLDKALRLFLRSGFSRVPVIGQDVDDAIGILYFKDVVRRLIDHADLAASPVMSYVRPAVFIPETRFVDDELREMQANNTHLALVIDEYGGVSGLVTLEDLIEELVGEVVDEHDRAELEPEQLAPNVWRVPARYSLNDLEELVGLEFDDEAVDSVGGLLTWAIDRVPLPGAEATVHGVHLVAEETVGRRNEVGSILVTRVDPEPAAEEEQGE
gnl|extdb|pgaptmp_000802	K07042	ybeY, yqfG; probable rRNA maturation factor	MSVEVINETTYPVELAEFGALAEYVLTQMHVGSAVELSIMFIDPEAMERLHEDWLGLPGPTDVMSFPMDELRPGTPASPTQEGILGDVVICPDVAVEQAKAAGHSDMDEMLLLATHGILHLLGYDHAEPEEEEIMFGLQRKLLLTFLAQA
gnl|extdb|pgaptmp_000801	K06217	phoH, phoL; phosphate starvation-inducible protein PhoH and related proteins	MDPAAFLGPADQVVRTIERGFPQVEIYVDHRQLRVVGPPATVNLVVTLLEELVALVQDGGTIDAATVQEVIALLPPATAGVRPPALQTGRGKVIKAKTPGQAAYLEDLETYPIVFGIGPAGTGKTYLAMAEAVSALLEGQVKKIVLTRPAVEAGENLGFLPGSATEKIDPYLRPLFDALDELLEEGSLPRLMASGAIEIAPLAYMRGRTLNDAYIILDEAQNTTPAQMKMFLTRLGFGSHMVVTGDDSQIDLGRGMTSGLVLIQEILKDVAQVKFSYLTSADVVRNPLVAEIIDAYSRWEAGRPQEAGTGPGSRPSPSSIERQTP
gnl|extdb|pgaptmp_000800	K09761	rsmE; 16S rRNA (uracil1498-N3)-methyltransferase [EC:2.1.1.193]	MTLPVFFAPELLGADRDDRGAALSWSGPEADHARKVMRLGPADRLDLVDGAGTRATCVIESVSSTGVELTVAESRVEPAPAVTVTLVQALAKGGRDEQAVELCTELGVDRIVPWSAQRSIARWPADRAEKSRQKWVNVVRAATKQSRRAFLPVVDQLVDSGRLAQQLAEISRRGGLVLICDEEGTRTLTDQVHMWEQTAGEIVVVVGPEGGLTDAERDAFRDGGGQLVLIGPTVLRSSTAGGAALTLVNVLTGRWR
gnl|extdb|pgaptmp_000799	K03686	dnaJ; molecular chaperone DnaJ	MRDYYEVLGVSSNATADEIKKAYRKKARQLHPDYAGADSEEAFKELSVAYDVLSDPQKRQQYDLGGPAAFSGGGAGPTGDFGFADLFETMFGGMGGFGGPTGPTQRTRRGQDSLIAVEVTLEEVVFGTKKEVKVNTAIECDVCHGSCCEPGTSPTTCPTCGGTGSVTRVQQTLLGAIRTSAPCRTCQGHGQTIDHPCHECAGEGRVRASRTVSFEVPAGVENGTRIRLAGKGEVGPAGGPAGDLYVEIREKPHPLFARQGIHLHTRIDVPVALAALGTVFTLDTLDGQQELVIDPGTQPEAELTLKGLGVARLGGSGRGNLYVHVGVKTPTNLDDRQRELLEELAELRGEQRVEPVGAGTHGPLKWFKDKLGGL
gnl|extdb|pgaptmp_000798	K03705	hrcA; heat-inducible transcriptional repressor	MARERQLEILRTIVSHYVDTREPVSSKTVAADGGMDVSSATIRNEMNVLESEGLIYQPHTSAGRVPTELGYRTFVDGLIDLQPLPEPQRRAIEQFLNEAVDFEDVIARTVRLLAQLTRSAAVAQFPVRAAARLRRLEVVDLASRWLIVVAITNDGQVYERRLDAGEAPGEDALTELRDRLNLQLEDMSATSIRLIAEDVVNEFKPAHRRLARLVVDTLLELLSQQTQSRLIVAGLSNLARTGEDFADVSGVLDALEQQVALLRLLSEVHTDQLQVSIGTENRDEDLEQTSIVSGAYHTADNGSAHVGIVGPTRMNYARSLIAVEAVSRYLSRLMLGENH

gnl|extdb|pgaptm p_000796			MGSLPPGDPWPPAPDRWDRAQQLGAERPTSAYIHVPFCTVRCGYCDFNTYTTEFGPGADRATYAQSVRAEIAHSARRLSLAPPAPLQTVFFGGGTPTLLDP<br>AELGLILAELNSSFGLAPGAEITVEANPETLTEDRVRQLADLGITRLSVGMQSAVPEVLSVLDRQHRPEQLPLVAQWARAAGLSYSVDLIYGAPTETVAQWET<br>SLRVALALDPDHLSAYSLIVEPGTKLAAQVARGDLPAPDDDEAAEKYQLADQLLAEAGYRWYEISNFARPEPGEEGLVATELTHASKHNLAYWRDWNWW<br>GYGPGAHSHWGDLRWWNVKHPRAYAGRLDQGLDPALAGETLDEPTRALEALMLGIRTAEGLALGPELSGVDQLLDRGLVELNAAGDRLTLTLSGRLLADL
gnl|extdb|pgaptm p_000795	K03439	trmB, METTL1, TRM8; tRNA (guanine-N7-)-methyltransferase [EC:2.1.1.33]	MEQLHTADEVQFRARTKSFVRRSRGLAQNLSRVLDQHGATYLVPVARADSATTIAPDTKIDWDQQFGRRAPLVVEVGSGNGEQIVAAAAAHPEQNYLSFE<br>VWHPGVAKMISRAASAGVTNLRIVEADAAQALPLLLGPDSVSEVWTFFPDPWRKKRHHKRRLVDGEFAGAVATILAPEGCWRLATDWDDYAWQMRDTI<br>EAEPNLVNPHRGARPDPLDPEPTRGGFAPRFPGRLLTRFEEKGLEAGRTVHDLTAIKPARKEQA
gnl|extdb|pgaptm p_000794	K03596	lepA; GTP-binding protein LepA	MSVPSPEQLQLIKPAHTPQANVRNFSIIAHIDHGKSTLADRMLQLTEVVNPREMREQFLDRMDIERERGITIKSQAVRMPWVVGDEAYALNMIDTPGHVD<br>FTYEVSRSLAACEGALLLVDAAQGIEAQTLANLYLALENELVIIPVLNKIDLPGAQPEKYADELASLIGISPDEVMLASGKTGEGVAEILDRIVREVPAPAGDPTA<br>PTRAMIFDSVYDSYRGVVTYVRVVDGSLKHRERIKMMSTGSVHELLEVGVISPDPVPAGGLGAGEVGYLITGVKDVRQSRVGDTVTTSVRGATEALSGYRDP<br>KPMVFSGIYPVDGSDFPDLREALVKLQLNDAALTYEPESSVALGFGFRCGFLGLLHLEIVRERLEREFNLDLIATAPNVVYSVTKEDGTEVRVENPSAFPDGKID<br>QVREPMVAATILAPSEFVGAIMELCQERRGALGGMDYLSPERVELRYRLPLAEIVFDFFDQLKSRTRGYASLDYHEDGSEVSDLVKVDILLNGEQVDAFSAIV<br>HKDAAYSYGVKMTKRLKELIPRQQFEIPVQAAVGSRVIARETIKALRKDMLAKCYGGDISRKRKLLEKQKEGKKRMKSIGRVDVPQEAFIAALTSDAPTGKK
gnl|extdb|pgaptm p_000792	K02968	RP-S20, rpsT; small subunit ribosomal protein S20	MANIKSQIKRNRTNEKRRIRNQAVKSELKTLVRKTREAVEAGDKALAEENLRIACRKLDVAVSKGVIHKNQARNRKSKLALRVNAMEG
gnl|extdb|pgaptm p_000791	K02340	holA; DNA polymerase III subunit delta [EC:2.7.7.7]	MAGRAGGGRGAQDNSPALVLSDLAPAPVLLFRGSERALADRALAYLRTQIRQRDPEVEVVEADASSCPPGQLSVWTSPSLFGDRRLIILLGVEEAGVELTDEL<br>LQTIANPPDDAILALYHRGGVKGKKIVDAVKRAGFPIWATEPLKFPRERLALVADEVANLNAKAPRECLQMLVDAVGDDLHELLGTTRRLVHDGGGVITREA<br>VDAHFAGRVDTGGFDVADAMAAGDGPRAVVLARRAFESGVAPVLVVAALAYQLRSLAKVSVPGLSGPSLGMRPAAEKQARRNLRHWSAESLGAAICAVA<br>RADADVKGASKDPEGAVERCLIEISRRNAAR
gnl|extdb|pgaptm p_000790			MGEEARPRDYRLVPLAGAAWLGVVSALQGWGIAGVLGGELALACGWGWVWFRTDWGKKLRWLGLATGVALALGGVAGQLAARDYRADPAVQLSDG<br>AQLRALVRLDSGAVLRATPWGQPEAVATGSITAVAEGDRWIRSGAQVRLSGSPDVLLNVGPGTTLRVSGRLERGFWEQPPRVGQVRVRTATVAAEPSAW<br>LRWVGLVRNRMLDLCAGLPAPAGALVLGMSVGDRQLLDQDLSEAMRVASLTHLTAISGTHIAVAMAVISLVIPGWKWVRPLFLLAFLAVLIGVVGPTASVV<br>RAAAMAALVIVGWWGYRSSQSQVALAAVVLGIVLLRPWMALQYGFTLSVLATAGIIFSGTRWADRLGRWSVSRWGKVMGERPLKWGSSAVAVSAAASL<br>WVLPVLVTLNPWISLWAIPANVLATPAVAPTTILGLLAATTVTWAPTLADWCAKLAALPAGWISWVAQLFASLPAAKLPWPAGPTGPILAAILVGLVLGTIEF
gnl|extdb|pgaptm p_000788	K00286	proC; pyrroline-5-carboxylate reductase [EC:1.5.1.2]	MRLGFIGTGAMTSAIVKGLREAGPSDTGIFLYDVNTHAAEQLAAVTDSATVDEPTDLLAKCDLIVLGVKPHVQSSVLRQLAPHVTADACPALLSIAAGRTLAAI<br>RSDLAEAQPQPHLIRVMPNVNAQIGLSMSAVAYSDGTPAEVQEQARTLLEAVGLVMDLPEQLFSAYSALAGCSPAWFFQIVDSLAQAGVKYGLSKAQALT<br>AVTQSMVGSARLLQLSAEEGVNPSALIDRVCSPGGTTVAGLLAAQEAGLSPALVKAVDAAVARDLELG
gnl|extdb|pgaptm p_000787	K01873	VARS, valS; valyl-tRNA synthetase [EC:6.1.1.9]	MPDSHSPRTLPAARVPDRVSTDGLEEKWGEAWNEQQTYRFDRTAERSQVYSIDTPPPTVSGSLHIGHVFSYTHTDTVARFQRMRGKRVFYPMGWDDNG<br>LPTERRVQNYFGVRCDPTLPYEPGFVPPHEGGEGKSIKAADQQPISRRNFIELCVRLSEEDEQQFEKLWRYLGLSVDWSLTYQTIGDEARQVAQAAFLSSLER<br>GEAYQAEAPGLWDVTFQTAVAQAELEAREYPGDYHSLAFHGSGGDVVIETTRPELLAACVALIAHPDDERYQPLFGTTVTSPGFNVEVPVLAHPAAEMDKG<br>AGIAMCCTFGDLTDVEWWRDLQLPMRAILEKDGRITRSTPDWITDPQGVALFEQMAGKTTFGARKAVVEALAASGEMIGDPVPTKRMTNFFEKGDKPLEI<br>VASRQWYIRNGGRDFTRAGQAQNLREELLAAGRQLNFHPDFMRVRYENWVQGLTTDWLVSRQRFFGVPLPLWYRIDEHGTVRYDQVLTPTAEQLPVDP<br>TIDVPPTFTEDQRNQPGGFAAEVDIMDTWATSSLTPRIPTGWLSDPDLFSRVYPMDLRAQGQDIIRTWLFSTVLRAVSEFGELPWTNAAISGWILDPDRKK<br>MSKSKGNVVTPMGLLEKHGSDAVRYWAASARLGTDAAFDEGQMKIGRRLAIKVLNASKFALTMGDEGADLLLDPALVVNPLDASELANLRRVIEVATEAFT<br>GYDHTRALEVTETFFWTFCDDYLELVKERAYNREGQWSEAEANSARATLALVVDAVVRLLAPFLPYSTEEVWSWYRDGSVHVAPWPNPDEYLVEGAQPEL<br>MDAASAALIVLRRVKSDAKVSPRTPLLSVTVSGPAELLGLAQTVSSDLEATSHAQTPINWVEGGEELTVTQVELGEAPKRQK

gnl|extdb|pgaptmp_000783	K04092	tyrA1; chorismate mutase [EC:5.4.99.5]	MEALLRERSAIDNFDAALIHILAERFRCTEQVGHIKARGGLPPADREREAQQVQRLRALAEESGLNPDFAEKFLQFMVTEVIRHHEVIKREYD
gnl|extdb|pgaptmp_000782	K03544	clpX, CLPX; ATP-dependent Clp protease ATP-binding subunit ClpX	MSRLAEGTDLLKCAFCGKSQKQVRKLIGGSGVYICNECVDLCREILEEELEEQVIPRQISLPKPKEINAFLDSWVIGQDQAKKALSVAVYNHYKRVRHREAGNSEEMDGTKSNILLLGPTGTGKTHLARSLARLLEVPFCIVDATALTEAGYVGEDVENILLKLIQEADGDIKKAERGIIYIDEIDKISRKGENASITRDVSGEGVQQALLKIIEGTRASVPPQGGRKHPHQQFLEIDTSGILFIAAGAFSGIEEIVRQRLGRRITGFGTDLQAAAEVEDFYAEINADDLHKYGMIPEFIGRLPVLTSTSELSEEDLSRILTEPKNSLVAQYQHLFDLDEVELEFTSEALSAMARLAMARKSGARGLASIIEHTLSDLMFEIPSRPEVERVVITGEAVEGTAAPLLYVNTAKESRSA
gnl|extdb|pgaptmp_000781	K01358	clpP, CLPP; ATP-dependent Clp protease, protease subunit [EC:3.4.21.92]	MNSTNAYFEAMARQLPEARYVFPSFEERTAYGYKRQDPYAKLFEDRIVFLGVQVDDASADDVMAQLLVLESMDPDSLITMYINSPGGSFTAMTAIYDTMQYIRPQIQTVCLGQAASAAAVLLAGGSPGKRLALPNARVLIHQPAMDGVRGQASDIQIVADEIDRMREWLEDTLARHSGMDVEQVRRDIERDKIFTADQAKEYGLIDQVLASRKMA
gnl|extdb|pgaptmp_000780	K01358	clpP, CLPP; ATP-dependent Clp protease, protease subunit [EC:3.4.21.92]	MSHPEVTSQDVQNPSGLGDSVYQRLLKERIIWLGGEVTDDSANAVCAQLLLLAAENPDEDIYLYINSPGGSVTAGMAIYDTMQYVKPDVATVGMGLAASMGQFLLTAGSPGKRFITPHTRVLMHQPSGGAGGSATDIRINAELILHMKRELAEIIAEATGKSVEQIDRDFDRDKWFTAQEALEYGFVDHLVSGQAEVSEKSKQEEGN
gnl|extdb|pgaptmp_000772	K03545	tig; trigger factor	MKSTVESLEPTRVKVTVEADYDELKPDMDKAYREIAQQVSIPGFRKGHVPPRIIDQRFGRGVVIEQVVNEVLPGLYSRAVMDNELRPVSQPDVDVVEVPAAEGEPGGLLKFTAEVDVVPAFDVPEFEGLEVEVSPVEVDDEAVQAELDELRGRFATLKNLERAAEDGDYLTLDLEAKVGDEVIDTLSEVSYELGSGNMLEGQDEALRGQEAGAEVTFTSTVRGGEYAGQDATIEVKVISVKERELPEADDDFAQMVSEFDTAEELLADLREQVARRGVSQQALEARDKLLAQLLEQTEILLPESAIEHELSHRVDENTSDEDKQSIREAVENDLRQSIFLETLAEKSDVQVGQQELFEFMMQTAQTFGMDPGQLFQDQRQIQNMMVELARTKALVAVLRGATVKDTNGELVDISEFTADPAEAEAPSFEEQIEEVTEEVAEEAKED
gnl|extdb|pgaptmp_000294	K02566	nagD; 5'-nucleotidase [EC:3.1.3.5]	MTIELSKVTSKGEFGPVMAWLTDMDGVLIRENSAIPGAQDFLEELRRRELPFLVLTNNSIFTNRDLSARLAQSGLDVGEDQIWTSANATAAFLSQQSPRSSAYVIGSAGLTTALHNADYIMTEVNPEYVVLGETRTYDFNAITTAIRLIESGSKFVATNPDVTGPSDEGTLPATGAVAAMISAATGKSPYFIGKPNPVMIRAGLNKIGAHSETAAMIGDRMDTDILAGVEAGLRTHLVLSGSTTLEQVADYPYRPAWIHNSIADILALL
gnl|extdb|pgaptmp_000308	K01126	E3.1.4.46, glpQ, ugpQ; glycerophosphoryl diester phosphodiesterase [EC:3.1.4.46]	MRIIAHRGVSSLAPENTMAAFVKCLEVGAKWFECDVRMLGDNSLIVAHDETAERTTTGTGRFFDLTFADLRRLDAGRWFGDGFRLERVPELATVLDLLNTTGLSANLELKFEESDPEVVRTYLETIARSTRALKQPDRVLVSSFSAELLSGMAALAPDLARALLVDEVGASVDQVVAQARDLGCDAVNPGLEGLTADRVQALKAAGMAVYVWTVNSVETARELAEWGVDGVFTDYPQDLLAAGLDQE
gnl|extdb|pgaptmp_001072			MIDIGLVGAFLGGVLSLLSPCSVMLLPAFFAYAFTSPAKLLSRTGIFTLGLLTTLVPLGVFSGILGSLLGENRSLLITVVAGLIIVIGLIQLLGISLPGLSMREAAGTDRTSVVSVFLLGAVYAVAGVCTGPILGAVLMMASLGGSAGYGALLLAVYALGMVFPLLVLTLVWQRWGAGATRWMRPRLIRIGPWENSLVAVVTGLLSIGLGTLLLVTDGTANLGGLVSISTQFRLESAVANLGAAVPNWAFALGAALILTLVIWLVRRRNRRPQPAQSWPVAPGGTEEGRG
gnl|extdb|pgaptmp_000990			MSDVTTALPTHEVILTDEAAVKVQSLLEQEGRDDLRLRVAVQPGGCSGLIYQLYFDERLLDGDAVREFGGVEVVVDRMSVPYLSGATIGFADSIERQGFTIDNPNAGGTCACGESFH

<table>
<tr><td>gnl|extdb|pgaptmp_001014</td><td>K01681</td><td>ACO, acnA; aconitate hydratase [EC:4.2.1.3]</td><td>MTKDSFKARGTLAVGGKEYDIFRLAAVPGTEKLPYSLKILAENLLRNEDGENITADHIQAIANWDPAAQPSHEIQFTPARVIMQDFTGVPCVVDLAVMREAFAQMGGDPNAINPQVPAELVIDHSVQIDVFGQKMAFQRNVEREYERNYERYQFLRWGQTAFDSFKVVPPSTGIVHQVNIEYLARGVFTAEQDGRTLAYPDTCVGTDSHTTMVNGLGVLGWGVGGIEAEAAMLGQPISMLIPRVVGFKLTGSIPPGATATDVVLTITEMLRAHGVVGKFVEFIGEGVGQVPLANRATIGNMSPEFGSTAAIFPIDQVTLDYFRLTGRSEEQVQLIEAYAKEQGLWHDPSAQVEYSEYLELDLSTVVPSIAGPKRPQDRISLTESKNSFRSSLKDYVDYDTEVHETSLDHALEGSFPASDPVNLDDMKDRNGSQPEHHELQGDRAHKVVPVTLADGTETVLDHGAVAIASITSCTNTSNPSVMLAAGLLARNANQKGLKVKPWVKTSLAPGSQVVTDYYEAAGLMPDMDALGFNLVGYGCTTCIGNSGPLPPEVSKVVNEEDLAVVSVLSGNRNFEGRINPDVKMNYLASPPLVVAYALAGTMDFDFVADPLGQDQDGNDVYLADIWPDADEVQRVIDSSVTREMFLKDYADVFKGDEHWNQLEIPHGSTFTWEGDSTYVRRAPYFEGMPATPEPVQDITGARVLLRLGDSVTTDHISPAGSFRSDSPAGQYLLEHGVQPRDFNSYGSRRGNHEVMIRGTFANIRIRNEMLPGVEGGFTRDFTREGGPQTTVFEAAQNYLAEGTPLVVLAGAEYGSGSSRDWAAKGVSLLGVKAVITESFERIHRSNLIGMGVLPLEFPAGENRNTLGLDGTEVFDISGVTELNEGRTPATVHVRATKDDGSTVEFDARLRI</td></tr>
<tr><td>gnl|extdb|pgaptmp_001171</td><td>K03551</td><td>ruvB; holliday junction DNA helicase RuvB [EC:5.6.2.4]</td><td>MDDDLLDGAVHDEERAAEAALRPRSLAEFIGQPSVRQQLSVLLAAARGRQEAADHILLVGPPGLGKTTLAMIVAQEMGASLRLTSGPVIQHAGDLAAILSSLQEGDVLFIDEIHRLARTAEEMLYLAMEDFRVDVIVGKGPGATSIPLALPRFTVVGATTRSGLLPAPLRDRFGFTAHLEYYSPGELTEVVNRSAQLLGVPVEADAAGEIASRSRGTPRIANRLLRRVVDYTQVAEHARVELEQARSALHLFEVDQLGLDRLDRAVLEALTKRFGGGPVGVGTLAMTVGEEPETVETVSEPYLVREGFIVRTPRGRCATQRAWEHVGLVPPAGSPVSGIAQLSDFREEPGPNGTQPLFNQ</td></tr>
<tr><td>gnl|extdb|pgaptmp_001156</td><td>K07447</td><td>ruvX; putative pre-16S rRNA nuclease [EC:3.1.-.-]</td><td>MIVRAGIRLGVDVGSVRVGVARSDQAGVLVLPLETVPRDRRGQDLRRIVALVREYEAIEVVIGLPLHLAGGEGESARMARSFGRALKRRLPKTRVCLVDERLSSNQAHGRLTEAGLSQAQQRMIVDQVAAQVILEQALASERATGLPPGEPIEKVSPDERNGSE</td></tr>
<tr><td>gnl|extdb|pgaptmp_001127</td><td></td><td></td><td>MNAQTTAGEPRQGMLFGDPLENLGAAAGYRGPIACQAAGITYRQLDYWARTGLVVPSIRMAKGSGSPRLYSFRDILVLRVVKKLLDAGVSLQQVRTAVGQLADRGVDDLASITLMSDGASVYECASTDEIVDLLEGGQGVFGIAVGRVWREVEGSLAQFPVEDQGEVIGLDQLEQRRRQKRNAS</td></tr>
<tr><td>gnl|extdb|pgaptmp_001118</td><td>K01733</td><td>thrC; threonine synthase [EC:4.2.3.1]</td><td>MEPSIAQPGLINRYRQWMPLGETDPVITLDEGSTPLVFAPKLSALVQGEVWVKVEGANPTGSFKDRGMTVAVSMAAAEGAQAVVCASTGNTSASAAAYAAKAGMTPVVLLPAGKIAQGKLAQSIIYGAKIVQIRGNFDDCLTIARKLDEAYPIALVNSVNPHRIEGQKTAAFEIIDALGRAPDLHLLPVGNAGNITAYWRGYTQYFEAGLAGSRPQMWGFQAAGAAPLVLGHPVLEPETVATAIRIGNPASAEQALAARDESGGLIAAVTDEEILAAQAYLAAEEGIFVEPASASGVAGLRKRAEAGQVPPGKLIVITVTGNGLKDIDTALTGRSFETAVADADVYQAAQACGL</td></tr>
<tr><td>gnl|extdb|pgaptmp_001105</td><td>K00931</td><td>proB; glutamate 5-kinase [EC:2.7.2.11]</td><td>MKKPLRRRDQIPTSHRVVVKVGSSSLTGEDGHLDIARLERLVNVLSERRLAGREVVLVSSGAQAAALGPLELPGKPKDLATAQAAASVGQGLLMAEYTRAFGRHDLRVGQVLLTAEDILRRGQYRNSRRALERLLSLGVVPIINENDTVATDEIRFGDNDRLAALVAHLVQADALVLLTDVDGLYDSPPSLPGARKLTEIHRFEEVEHLDIRARGSKVGTGGMVTKVQAAALATSSGIPVLLTSADEAAEAMAGQEVGTWFHPTGRRLPARQLWLAYAAKIHGQVVIDEGAVRAVGGRGASLLPAGVVGVNGTFRPGQAVEIVSESGLPIARGLSGFSSSRIPEVMGRSMAEIEEQLGHDYAHEVVHRDELVVMARHRRLSE</td></tr>
<tr><td>gnl|extdb|pgaptmp_001324</td><td>K00969</td><td>nadD; nicotinate-nucleotide adenylyltransferase [EC:2.7.7.18]</td><td>MRDRRRNQESEKRRQRSQGQRRIGVMGGTFDPIHHGHLVAASEVMDRFNLEQVVFVPAATQPFKAGREVAPAEHRYLMTVIATASNNRFTVSRVDIDRGGLSYTYETLRQLHAEDPEVEWYFITGADALESILEWKNAPELFELAHIIGVTRPGHVLGKAELPADSVSLLEIPALAISSSDCRERIRTGKPIWYLVPDGVVQYIEKYGLYR</td></tr>
<tr><td>gnl|extdb|pgaptmp_001327</td><td>K02888</td><td>RP-L21, MRPL21, rplU; large subunit ribosomal protein L21</td><td>MVYAIVKAGGRQEKVSVGDTLVVDKLADEIGSTVEFQPVMLVDGENINTDAEKLAKVSVKAEIVDSAKGPKITIIKYKNKTGYRKRQGHRQPLSVVKITEIA</td></tr>
<tr><td>gnl|extdb|pgaptmp_001334</td><td>K07695</td><td>devR; two-component system, NarL family, response regulator DevR</td><td>MIVDDHEIVRRGIAEIIDRESDLEVVAEAGTVEDAIRRASLVKPDVILVDLQLPDGTGVDIMKSLSESNPQIRSVVLTSFDDDNALSESVAAGARAFILKTVRSGEITDVVRDVAAGRVLLDERTMTRRRGEIDDPTVNLTPTERKVIELIGDGMSNREIGDSLGIAEKTVKNHITSLLAKMGLQRRTQVAAWVASQRSAAWRSGAK</td></tr>
</table>

gnl|extdb|pgaptmp_001353	K00817	hisC; histidinol-phosphate aminotransferase [EC:2.6.1.9]	MRLPVRPDLAAVEPYGAPQLEVPVRLNVNENPYRPGPEVVESVTERIRGAMTGLNRYADRDATELRAGLARYLARESGVELAVDQVWAANGSNEIMLQLLSAFGGPGRIALGAAPTYSMYQEYARDTFTDWELLAPPADGGLFPGVQVDRVIEALHRVRPAVFFLPNPNNPTGQMVPLTEIERLLEAARWSGPTVDGEPSATLIVVDEAYAEFRQPSTASALTLLERYPHLVVTRTMSKAFAAAGLRLGYLAADPALVYEIQKVRLPYHLSLLTQAAATAVLEHTDAQLAQVALLRGERERLADWLRRQGYRVAPSEANFLLFGPLEDRESVWQQLVDRGVLIRIVGPAGCLRVTVGTPEENERFRTALVEVTQ
gnl|extdb|pgaptmp_001379			MSPDQLSAGEQAEIQHEQQFVDRAYANLDSSRARYRVAQREVEAQGAWGTPQARTERDAMAAHYGDQAARLESIEDRLVFGRIDPEEGGTIYIGRAGLRDEDGSRLLVDWRAPAAQPFYQATAVEPHGVIRRRHISTHLRRVRALEDEVLNTEAAHRSGLQFQGEGALISALSQARDGRMGDIVSTIQAEQDAVIRAEDRGLLVVQGGPGTGKTAVALHRAAFLLYTHRERLERSGVLIVGPSPVFLRYIEQVLPSLGETGVVSVTMGDLLPGYSTNLADSIDVAEAKGSLAWLPTLAQAVKDLQRIPAADHTFAVGGRRAVLTRELVRAARTRARRTGKPHNEAREGFALELVNELAQQLAGPDPDPETLSWWREEVRALPAARREINLCWMPTRAADLLRRLYARPDLLARVNRGLSPEQLRLVQRPADAPLTVSDIPLLDELEELLGFSSLLGPAPATKSRADEDEVRRAQEAIDSQQLGGGLVSAEVLAEQTRGQRDWQPLAERARSDRTWTFGHVVVDEAQDLSPMAWHALMRRCPSQSFTVVGDLDQHRGHSHPQSWVEVLGPAGRALQDERSLTISYRTPKALTELSQDVLAEVGAPVRFPLTSARDIPNSLADTVTGDLPAAVREVMAQELELLDREFGPGGGRVGVLVSDERATSWRADMEGVTSFNQRVSLLSVVAAKGLEFDTTIVVEPAEILAD
gnl|extdb|pgaptmp_001385	K00016	LDH, ldh; L-lactate dehydrogenase [EC:1.1.1.27]	MPTARATSLYPSAYGKPAKIAVVGAGAVGTAVAYACAIRGAARDIVLYDINETKVQAEALDMAHGIQFTPVGSITGSADIEIVRGSDLVIVTAGAKQKPGQTRMELAESTVSLMKVIVPQLMEVAPDAVYLYVTNPVDVVTYVALKIAGAPRRQIFGSGTVLDTSRLRYLVSLETGVATQNIHAYVTGEHGDSQVALWSSAEIGNVPLTQWGPTLSGRRFDHALRTEIAEEVVNSAYTIIEGKGATNYAIGLAASNIVNAVLRDESRVLTISSLLDDWQGISDVCMAAPTIVGRAGAGRVLRPPLTDEELAGLTDSANHIRSVARSLGF
gnl|extdb|pgaptmp_001397	K27043	clgR; XRE family transcriptional regulator, stress-response regulator	MFTKEPPLLRAELGEVLRSLRQAQGRTLREVSSSAQVSLGYLSEVERGQKEASSELLDAICNALGTPLWFVLREVSDRLALLDEARVPDTVPDNLLPATLLS
gnl|extdb|pgaptmp_001399	K03466	ftsK, spoIIIE; DNA segregation ATPase FtsK/SpoIIIE, S-DNA-T family	MRARKDQSKAKADANRSSGAGRIKGAAPAKASKATPSKAEPAGPSAPARFFRALGRGLAGLVHAWRSTDSQLKRDAFAFIFLAVAGVVALREWFGISGDAGAVIHHAVAGAVGIFAVVVPLLLVLVAVGLFRARKDPQAMPFRFAGGIGLSLALTGLVQVSRNNPSLSPIAGVEEAGGILGWFIGYPLMRLLSLWGAVVVLVALACYSILVMTRTPLREVPIKARELWASLRERTGRTQVDNDHLAAGDDEWLQEINRERKPAPTYVDQPTRAVDSFDIALEDDYPVDAPTEAVYAVEDYPAEPDYTMTAPVPVPTAMPVSEAVEPDDQTVALAEITSEDLSPLEAARAAEMANYQVPSVGLLQQGAPHLERSAANDRVVEALQTVLDQFDIDAQVVGFSRGPTVTRYEVSLGPGVKVERITALSKNIAYAVASADVRILSPIPGKSAIGVEIPNSDRETVALGDVLRSPAAQRNPHPLLVGMGKDVEGGYVVSNLSRTPHLLVAGQTGSGKSSFINSMIVSILTRATPEQVRMILVDPKRVELTIYEGIPHLVTPIITDPKKASEALEWVVREMDARYNDLADFGFKHVDDFNKAVVAGRVLPPAGSQRKLRPYPYLLVVVDELADLMMVAPRDVEASVQRITQLARAAGIHLVLATQRPSVDVVTGLIKANVPSRLAFATSALADSRVILDQPGAEKLIGQGDALYLPSGMNKPLRVQGAWVDEAEIHRVVEAAKSQMQPEYREDVIEPPKTAKVADDIGEDLDDLLAAAELVVSTQLGSTSMLQRKLRIGFARAGRLMDLLESREIVGPSQGSKAREVLVQPEQLASVLAVLRGEAELVEKAQSSPTEERSVTPPTPNTGTVVMETKTVQLEGR
gnl|extdb|pgaptmp_001437	K01129	dgt; dGTPase [EC:3.1.5.1]	MDRELADWHLPLYGDFDQERFVTEPPKAPTRTPFERDRARILHSSALRRLGTKTQVLGPASDDFVRTRLTHSLEVAQVGRGIGAELGCDPDIVDAACLAHDLGHPPFGHNGERALNEAAADAGGFEGNAQTFRLVVRLEPKVISGDLRAGLNLTRATLDAICKYPWARGEGPNLEKSQRKYSVYSDDRPVFDWLRQHAPEGRRCLEAQVMDYSDDIAYSVHDLEDAVVTGRAKLPALRDPGRLRQVLADTIDWYGASFSEDELADAARRLIELPYWPEDFVPTYQGNAAMKDLTSQLIGRFCGEAVAATRGQYGSEPLGRYRANLVTPREVLAEIQFLKGVAVSFVMSPREHDPRYYQQRTIVLDLVEALMEAGPTELEPQFAQEWESATNHAGHLRAVVDQVASLTDRSAVQWHARLCGMLSSLL
gnl|extdb|pgaptmp_000808	K00806	uppS; undecaprenyl diphosphate synthase [EC:2.5.1.31]	MSIPDPRPIRAETKMPKHVAVIMDGNGRWANARGLPRTEGHRAGELALMDTVAGAVEAGVGYISMYAFSTENWSRSPAEVRFLMGYSRDTIRRRAAQLHRWGVRIRWVGRAPRLWKSVINELRAAEELTANNRGTQLLLCVNYGGRAELTDAARALAEEVERGERTARSINEKALARHLYVPDVPDVDLLIRTSGEQRISNFLLWQLAYAELDFVDLAWPEFGREQLWERLLAYGSRQRRFGGAVDLVEPA

gnl|extdb|pgaptmp_001018	K01915	glnA, GLUL; glutamine synthetase [EC:6.3.1.2]	MFDSAQSVVDFIARENIEYLDVRFCDLPGVQQHFTVPAQSLDAEELSRGMMFDGSSVRGFTAIHESDMKLLPDFSSAYLDPFRAAKTLVLLFSIVDPLTDQPF<br>SRDPRQVALKAEAFLRSSGIADTCFIGAEAEFHIFDSVRYQVQPENTFYQIDAASAPWNTSSELPNLGHQMTFKGGYFPVSPQDHYADLRDSMSNLLHQVG<br>LEVERAHHEAGAVGQQEINYRFASLTQAADDMLKFKYVIKNAALAAGKSATFMPKPVYGDNGSGMHTHLSLWKDGEPLFYDERGYASLSDTARWFIGGLL<br>EHGPALLAFTNPSLNSYRRLVPGFEAPINLVYSSRNRSACIRIPVTGTSPAAKRVEYRVPDPSANPYLSFAACLMAGIDGIRNRLEPALPIDKDLYELPPADYQDI<br>AKLPTSLDGALAALKADHDFLLEGDVFTEDLLDTWITMKETQELAPSYVYPHPMEFQLYYGV
gnl|extdb|pgaptmp_000918	K13063	phzE; 2-amino-4-deoxychorismate synthase [EC:2.6.1.86]	MNYALIHRQGSPTVDRLSGTVEEVFRLADIALTGTEVLAMVPFRQVQERGFPARDDQLPILCLQVDRLETEPVADLITRLPAQAPPLEDLGFTQSDDDYAAV<br>VRTVIEEEIGSGEGANFVIRRDYRARLEADREQAALAWFRRLLERESGAYWTFAFITDQMIAVGASPERHVSTTGGVVRMNPISGTLRNGQPDVDTLLSFLA<br>DRKESEELVMVVDEELKMMSAACPDGGVMRGPFLKPMSQLIHTEYLLEGHSRLDPREVLRLTMFAPTVTGSPMGNACAVIARHEPTPRGYYSGVLARFTP<br>TERGYEVDAPILIRTAFLDADGQVSVSAGATLVRHSDPRAEAAETRAKAGGMLTALGLAQAPTPAVEQPTGSRPPVADPRVEAALADRNRHLAPFWRDEQ<br>VSGAELRAQVLVVDCEDDFTAMLAHQLRRLGLDVQVVPWHQVQDVEQPDLVVFGPGPGDPLSQTNPRIVRLRELIAQRLGRPLLAVCLSHQVLSDQLGLE<br>LRKLPAPRQGTPRLVRIAGQEARIGYYNTFTAVAADGSQVRGLTVDSEPDTGFLHALTGPGLASVQGHLESVLSFDGYDTLRRLVSDILDQ
gnl|extdb|pgaptmp_000919			MSVTLGQLIQFMEHRYPPATAESWDRVGLVVGNRSQPISRVLLAVDPVPEVVAEARGYDLLLTHHPLYLRGTSFLSEDEAKGRMVTDLIRRGTALYCAHTNG<br>DANAGGVADALADLLGLTGTEPLIPESGLGRIGTVPAQTVREFAHLVVERLPAGPTGLLVGGDLDAVISRVAVSGGSGDSFLEAARAAGAEVYVTADLRHHP<br>ASEHLINGGPALICGSHWATEWPWLPRLQAQLRAEFADSLQVDVSAQVTEPWALHLPTKGPTR
gnl|extdb|pgaptmp_000920	K07164	uncharacterized protein	MKALPLEQTHLVELQRLDLALARLRHQDRTHPARQEIVELAGRASDLQQAIVAAEARLDGASRQIEQVESEIEKVRQRRDLQQRRLDEGKVPIRDMSALEHE<br>VASIETRIATLEDKAMELMEGREKLAAGIEAARQNHAALLADQGAAEQRLAADLAVTGEEIDQLEAQRRHVVELLPAQLVTAYEGLQARLGPRVVIEMHEG<br>TLVDAPVELPLSELSELAMHPADQLYISDETEYLIART
gnl|extdb|pgaptmp_000923	K00886	ppgK; polyphosphate glucokinase [EC:2.7.1.63]	MTNIACGIDIGGSGIKGALVDLTSGEFIGERVRIETPHPATPEAVAKTCVEVLSQLQAPADVPVGIAMPAPLPQGIVPFMANLDASWEGVHAQSMYESFLGH<br>PVTVLNDADAAGLAEAVFGAAQGQDGLVIVTTLGTGIGSALIYRGVLIPNSELGHLEIGGYDAESRASAAQRVKQDLGWKKWAKRLQKYYSHVEMLFSPDL<br>FVVGGGVSKNSDKFLPLLSLRTPIIPAQLRNRAGIVGAAVAADRANQ
gnl|extdb|pgaptmp_000924	K01265	map; methionyl aminopeptidase [EC:3.4.11.18]	MNSKLAARAPLGTLTPGRISPERSVPRGIERPEYLFHDGPEVVTASDVKSPETIELIRQAGKIAADAIAKAGEAIRPGVTTDQLDAIAHQYLIDQGAYPSCLNYM<br>GFPKSICTSINEVICHGIPDDRPLEEGDIINLDITAYRNGVHGDTCAMFTVGEVDEESRLLIERTEQAMLRGIKAIRPGREINVIGRVIESYAKRFKYGVVRDYTG<br>HGVGEAFHSGLIVPHYDAAPAYNQTMEVGMVFTVEPMLTLGDVDWEQWDDNWTVVTRDRGRTAQFEHTVAVTEDGADILTLP
gnl|extdb|pgaptmp_000925	K00606	panB; 3-methyl-2-oxobutanoate hydroxymethyltransferase [EC:2.1.2.11]	MSRVRIPHLQKAYREGRRLTMLTAYDATVAPLLEAAGVDMLLVGDSLGNVALGHQSTLPVELSDMIRSTEAVARSTTRPLIVTDLPFGSFEQGTAQAFGSAA<br>TLLKAGAQAVKMEGGAERAHLIRFLVENGIPVMGHLGYTPQAENALGGPRLQGKGELGDKLLADAHAVAEAGAFGLVLEMVPASLARRVTEELDIPTIGIG<br>AGPDCSGQVLVWADMAGMGAWSPSFARRFGEVGQALTEAARAYVEETQAGTFPGPDHYRES
gnl|extdb|pgaptmp_000926	K01889	FARSA, pheS; phenylalanyl-tRNA synthetase alpha chain [EC:6.1.1.20]	MELDPSALDPRDEDAVSRWSAQAAAQLTAATDLTELKARKTEIFGDRSALTLANRTIKDLPGPEKAVAGKNLGRARQQLNQLLAERQEQLEQAHLAAVLVD<br>EGVDVSVASDRFPVGARHPLSLLIEEAGDFFLGLGWSIAEGPEVEHEWFNFDSLNFDADHPARQMADTFYVDGTSAGGAAAQDGSLVLRTHTSPVQSRTLL<br>EQGVPLYAVCPGKVFRSDALDATHTPVFHQIEGIAVDRGLTMADLKGVLDALAKALFGPEVKTRLRPSFFPFTEPSAEMDFWFPQKKGGPGWIEWGGCG<br>MVNPAVLIANGIDPDEYTGFAFGMGLERTLMLRHGISDMRDIVEGDMRFSEQFGLFGKGQ

gnl|extdb|pgaptmp_000927	K01890	FARSB, pheT; phenylalanyl-tRNA synthetase beta chain [EC:6.1.1.20]	MPYLSLSWLRDHVEVPGETTIAEVAEALVSVGIEEEEIHPAKVIGPLVVGRVLSVAPETHSNGKTVNYCRVDVGPYNDEPGTGKEHSELASRGIICGAHNFTVGDRVVVALPGAVLPGPFPIAQRKTYGHVSDGMICSERELGLGQDHDGIIVLDRQFPALSELAVGEDLIGPLQLGEEVLEVNVTPDRGYAFSIRGLAREYSHSTGAVFTDHGLAGELPPASDQGFTVEADPELCDRFVTQVVRGLDPHAPTPQWMVDRLEQAGMRSISLPVDVTNYVMLDLGQPLHAYALESVAAPIVVRRAKAGEPFTTLDGVERELSDEDIVITDSPNGQAGSRLIGLAGVMGGLDSEVGEHTTDVVIEGAHFNSVSIARTSRRHKLSSEASKRFERGVDPELAPVAVQRTAELLVQYGGGQLDPVRFDLDRTVPLPAVRLRLSEAERLTGRSYAPERIVEILELIGAQVTRDGDELVVQPPSWRPDLTGPAHLVEEIARIDGYDEIPTRLPATATSAALTPIQTTRRRVAQTLAQAGAVEVLSYPFIGSAHDRQHLAADDPRRQALRLRNPLADDAPLLRTTILDSLLDVAERNAARSNPRLAIFELGSVTLPAGTVPAPIPGVSQRPTEAELAALHAGVPAQPWHVAGVYGGPLGPSAVLSPTRTWDWADAIAQARRVASAVGVDLQVTRAWVPADTPRIPGPPVPTPAAAPEAVAPWHPGRVARLYARRGKALVEVGLAGELHPQVVSEYGLPARTAAFELDLDLLHDLVQAEPVQVEKVSVYPVAKLDLALVLPQTVPAADVERVLQQSVGPVLESLQLFDIYQGSQVAEGSRSLAYSLTLRAPDRTLGSKEVAKLREKAIHDLTKRLGAELRA
gnl|extdb|pgaptmp_000929	K03402	argR, ahrC; transcriptional regulator of arginine metabolism	MAQANTPIATKAARHGIITEVLASQPISSQEQLRRVLADRGIEIAQATLSRDLLEMEATKVRSEDGSLVYTVPNWDGTPTHQQEGTMTRLARWCQDLLISGDAAQNLLVLRTPVGAANLLGSAIDTARLDGVVGTIAGDDTILLICRDTEAAAAVYRQLLELADPAPSQKTTRSKHVAG
gnl|extdb|pgaptmp_000930	K01940	argG, ASS1; argininosuccinate synthase [EC:6.3.4.5]	MSQGKDRVVLAYSGGLDTSVAIGWIQEQTGQEVIAVAVDVGQGGEDLQVIRQRALDCGAVEAYVVDARDEFANDYLVPAIKANSMYQGRYPLVSALSRPLISKHLVKAARQFGGSTVAHGCTGKGNDQVRFEVSITSIAPDLKCIAPVRDLALTRDVAIDYANKHSLPIETTKHNPFSIDQNVWGRAIETGFLEDIWNAPTKDVYNYTDDPTYPPLPDEVEITFEQGVPVALDGQKKSVLEIVQELNRRAGAQGIGRIDIVEDRLVGIKSREIYEAPAAIVLINAHEELENVTLEREQARFKKRVDLRWGELVYDGQWFSPLKQSLDAFVDSTQLYVSGTVRMTLHGGRAVVTGRKSDTSLYDFNLATYETGDTFDQSHSRGFIEIYGLSAKQAAARDARFGRGLQL
gnl|extdb|pgaptmp_000933	K01478	arcA; arginine deiminase [EC:3.5.3.6]	MTARPRGDPDELDVLIVVGLGPTHPSNETTFVPRVESEVGRLESVIVHRPGLEIERLTPQNHDELLFDDLLSPTAAKREHDYFVEVMRDRGVQVLHFTDLLIETLDHPEARQYVLENTINRLYLGPLLAPAVEEWAAELDSAALASVCIEGLTLGDWQRVSPTSSLVAQTLAENEFLISPLPNHLFARDASAWIYGGVAINSMKRESRRREPLHYSAIYQWHPRFAGADFTRWTNGTSGPIRSVEGGDIMVLGDGLLAIGLSERTNPQGVERLASRLFAQGQADHVLAVMLPHQREFMHLDTVLTQVDRDSFIIYPPIRSARTISLVRDGDRPRLVMDDLPLEQALPRALGRPIRFIIPEGTEAELAREQWNDGFNMLALSPGQVVAYDRTPRSIRALEQAGIEVFSVFGSELGRGRGGPRCMSCPVRRAQV
gnl|extdb|pgaptmp_000934	K01755	argH, ASL; argininosuccinate lyase [EC:4.3.2.1]	MTTSGGNPEPEKISLWGGRFSGGPAEALTRLSVSTHFDWRLAPDDLAGSRAHARALAQVGLLTSDQLRGLLSALDRLEQDVLAGTFVALPEDEDVHTALERGLIERAGAELGGRLRAGRSRNDQIATLIRRYLRREGRRLAGLVLDVAEALLGQAYAARGAIMPGRTHMQHAQPVLVAHQLAAHIWPLVRDLERLQDWDRRAAVSAYGSGALAGNTLGLDPEQVARDLGFTSAVANSIDGTAARDVVAEFSFILTLIGINLSRVSEEIIIWNTKEFDYVTLHDSFSTGSSIMPQKKNPDVAELARGKAGRMIGDLTGLLATLKGLPLAYNRDLQEDKEPVFDQIDTLEVLLPAVAGMIATMSLHYDRMEELAPQGFSLATDIAEWLVKQGVPFRNAHELAGECVRVCEERGQELWELTDADLAEISPELTPDVRSVLSLSGSVEARAGRGGTAPERVAEQLGEAQANLDRLREWSQTN
gnl|extdb|pgaptmp_000935	K01866	YARS, tyrS; tyrosyl-tRNA synthetase [EC:6.1.1.1]	MSDLLEELQWRGLIAQHSDWETLTDALNSGPVTFYCGFDPTAPSLHHGHLVQVIIMRHLQRAGHHALALVGGGTGLIGDPRASGERSLQSTDVVAGWADGLRAQLERLLDFTGDNPARMVNNLDWIGSLSAIELMRDLGKYFRMGTMLNKDIVARRLESDEGLSYTEFSYQVLQAYDYLELYRRYGCTLETGGNDQWGNLVGGMDLIRKVEGKQVSVLTSPIITKADGTKFGKTEGGAIWLDPQLMSPYAFYQFWLQTSDDDVIRFLKIFTFLSRGEIEELEQQVREAPHLRAAQKRLAAEVTAYVHGAEALAEAESATAALWAGGDLRSLSASVLGEAVEKLPRGHWTPGETTLTEAMVSSGLEKGRAAARRTIESNGLSVNNEKNPDPEYVLQEADALPGGV
gnl|extdb|pgaptmp_000415			MSGDRGAKELLAEALKDQLQVMALSKVTVTGLTKQVGLTRQAFYYHFVDVYDLAVWVFETEVADHIMSHASYDRWAEGYQQLLTYMREHRTGVYAVIHSLGHHELERFLYGQFHQMMEAIVAELRGDLTVSAADQERVIRHFALVVLGYCMYWLASDMEADPSELVPEIEFLLQGQVRHALETYAARA
gnl|extdb|pgaptmp_000414			MFLFDSIPWYSWAAWFVVLGALIGLNELTRRSKAGGLLFFVALPIILTIFVWPNTATGSTGTWFHWVKVYSALAGCLGFMLIRYSPKVAAKRAALIFPPAILAINIMEACIRDFQVGAMNADGIVDGVYMLSGPWNWMNGIAGLINLITICGWFGIFISRGKSKDMIWPDMLWFWVIAYDLWNFAYVYNCVGDHSFYAGAALLISCTIPAFLIKKGAWLQHRAHTLALWMMFTMAVPSFVTTSKFSVAASGDPAALFTVSLIALSANIAVLIYQLYTVVKYRLNPLRDELYTRLPQHREVVEANAPLHSETPAEPLATPVSSR

gnl|extdb|pgaptmp_001936	K01885	EARS, gltX; glutamyl-tRNA synthetase [EC:6.1.1.17]	MSEGAGRYAPSPSGPLHIGNLRTALVAWVMARQTGRRFWLRIEDIDPHRTGAADQQIAELASLGLDWDGPVLTQTSRLARYDEVLAELAERDQLFECYCTRREIAEATRAPHTPPHHYPGTCAHLSEAERQERRAQLGDRAPALRLRSPKRDWTVHDEFFGDYSGPVDSFVLRRGDGAPAYNLACVVDDGETGVDQVVRGADLLPTSPGQAYLAQLLGYRTPTYAHVPLVVNSAGQRLAKRDGAVTLEDLGWPTERVLELLTGSLGGPAVTSLAEFRNTFTPADLPLVPYEFGVTTGLRPHRGG
gnl|extdb|pgaptmp_001935	K02071	metN; D-methionine transport system ATP-binding protein	MISLVDVSKIYQMPGGSNVVALDNVSLEVEQGAIHGIVGQSGAGKSTLIRCLTALERPTSGQVLVDGFDMAALSRSQLRDARRRIGMVFQQANLLDARTAAANIGYPLKIARQPADQIKARVEELLGLVGLAGRGDSYPAQLSGGQQQRVGIARGMASNPPVLLCDEPTSALDQESTDQILTLLKELRDASGVTVVIITHQMSVVTEICDSVTLLQNGRVVQSGSVDQVLSDVGSPLSEALIPPPSVEDADLRPGTKLLDVAFTSRPGVPTGATMLNLIASMGGDIASGRFESIGELQVGRLAVSVPAYSAASIIEELRRNNVAVEERTQ
gnl|extdb|pgaptmp_001933	K02073	metQ; D-methionine transport system substrate-binding protein	MRKIRNFALIGAAGALALAGCSTGTSDQSTDQSADQSTSQSDAAASGETVTLTVGASPSPHAVILQYVQDNLAADAGLDLKIVEYTDYVQPNEALAAGDLDANYFQTIPYLEEESEARGYDFVAGEGIHLEPLAIYSDKIESLDDLPEGAKIGIINDPTNQGRALALLAENGLVELPASGDVNVNTVTKKKDFTFVETEGAQLGRSLADVDIAVINGNFAQEAGLAPADSLAIESTENNPALNVLVWAKGSPKEDSIKKLDELLHSPEVAEFIQQQWPDGSVIPAA
gnl|extdb|pgaptmp_000416	K00761	upp, UPRT; uracil phosphoribosyltransferase [EC:2.4.2.9]	MEIIHTNHPLVSHKLTVLRDKNTTPSQFRHLVDELVTLLAYEATREVSVVPVQIETPVAPTEGLGLAEPRPMVVPVLRAGLGMLEGMLRLLPTAEVGFLGMVRNDETLEIDTYAERLPDDISGRQVFVLDPMLATGSTVVEAIEYLLKRGAKDVTVVSILAAKPGLETVERGVGHRAQVRVVVAGIDPELNENNYIVPGLGDAGDRLYGVVD
gnl|extdb|pgaptmp_001916	K04564	SOD2; superoxide dismutase, Fe-Mn family [EC:1.15.1.1]	MPVYVLPELPYSYDALEPYISAEIMELHHSKHHQAYVDGANAALAALAAAREAGDQAAINLHEKNLAFHLGGHSNHSVFWKNMTPNAKPGPEGALKEAIDASFGSLDAFKKQFGAAALGLQGSGWGVLAYDTISGGLVTFQLYDQQGNVPVGTVPLLMLDMWEHAFYLDYKNVKAKYVEAWWNVVNWDDVAERYQRAKEGFGSLLV
gnl|extdb|pgaptmp_001697	K02355	fusA, GFM, EFG; elongation factor G	MAQEVLTDLNKVRNIGIMAHIDAGKTTVTERILFYTGINYKLGETHDGASTTDWMEQEKERGITITSAAVTTFWKGYQINVIDTPGHVDFTVEVERSLRVLDGAVAVFDGKEGVEPQSETVWRQADKYDVPRICFINKMDKLGADFYFSVQTIIDRLGANPIVMELPIGAESDFEGVIDLIGMRSLYFPAKDENGQPTMGSLVVEGEIPADMVEKAEEYREKLMEAAAEGSDELTELYLENGELTNEQIKQGIRALTISGTAFPVYCGTALKNTGVQPVLDAVIDFLPSPLDIGNVHGFVPGKEDQELTREPSEDAPFSALAFKIAAHPFYGKLTFIRVYSGKIESGQQVLNSIKGKKERIGKIFQMHSNKENPVEMAHAGHIYAVIGLKDTTTGDTLCSINEPIVLESMTFPKPVIHVAVEPKSKADQEKMGLAIQKLAEEDPTFTVELDQETGQTVIGGMGELHLDIIVDRMRREFKVDANVGKPMVAYRETITRPVEKYEYTHKKQTGGSGQFARVIIALEPMEANAEEDFIFEDKVTGGRVPREYIPSVGHGIRAAMDTGVLAGYPVVGVKATLLDGAYHDVDSSEMAFKIAGTMAMREASKKAGAVLLEPVMEVEVRTPEEYMGDVIGDLNSRRGAIQSMDEQHGVRVVRALVPLSEMFGYVGDLRSKTQGRAVYSMQFNSYAEVPKAVAEEIIGKSRGE
gnl|extdb|pgaptmp_001698	K02992	RP-S7, MRPS7, rpsG; small subunit ribosomal protein S7	MPRKGPAPKRPLADDPLYGSKVVSQLVNRILLDGKKSTAERIVYGAMEIVAEKTEQDPLTVLKRALENIRPSLEVRSRRVGGATYQVPVEVRPSRATTLALRWLVDFSRKRREKSMTERLANEIMDAANGLGAAVKRREDMHKMAESNRAFAHYRW
gnl|extdb|pgaptmp_001534			MTERKTLGVGILGAGFIGNFHVRAWQGVRDGDIVAVCSRTLTKAKELAKTAAETGVGTDVASYTDVVDLVRDPRVDAIWVLTPNYTRLEVIRAITDEVIQGRAELTAIAIEKPLGRTVAEATEVLEMVQSAGLLHGYLENQVYAPGLVRAREVIWQRGAAAAGSPYLARAAEEHSGPHNTWFWNGVTEGGGVLNDMMCHSVEAGRFLLTPPGKSSSEWLRPVAVTATIASLKWGREEYARQLKADYPGVVDYTKSPSEDYAHAVFEFVNGDGEPVVVEATTSWSYVGAGLRLSFELLGPEYSMESDTLSTESKVFLSRSLEQSEGEDLIEKQNAEQGLMPLLSDESVSYGYTGENAALTRDFLNGTQPLESLVAGVEVMELLMAAYRAAETGQTVRWPIDLHDF
gnl|extdb|pgaptmp_001533	K10440	rbsC; ribose transport system permease protein	MRQPEAPLEEMPTGTRNSVPSWLRNPALVAGIIAIILIVVGQLVSPGFGSYGQVVSMLRVASFLGFIAIGQTIVILTGGDGIDLSVGKVATFAAIIASKMMAGSDEGLLLAIAVPLAVGAAIGLVNGLGVLYLRIPPFVMTLGMMGVVQGLILAYTQGKAGGRSAPALTALVNDRWLFQLPGVLFLWLIATILITLMLRHTSLGWNIYAVGANRTAARLSGVPVNRTILLAYSASGLFAALGGIMLLGYTETVFLNLADDYTLRSVAAVVIGGTLVSGGIGGYVGSAVGAILLTVLTSFLTTINMPESGRIVINGLVLITLLAVYGRQRRLRS

gnl|extdb|pgaptmp_001532	K10440	rbsC; ribose transport system permease protein	MSELIVRDGSKPWTSRLTFTLSSAITGLLLVAVVVVNVAVQPTFFTLYSFTSNFATFVPLVFAALAQAIVVIGGGLDLSIGAQVALISVIALRVMDGQDSRIVLGL<br>LAAILAGAICGAINGLVVAIVRLQPLIATFATASVFSGLALFVLPAPGGAVPPAMTSGYRMAVAFVPVPVIIVVLGGLLWWMVSKTKFVRHLYAVGGDREAAY<br>ASLVPVTSVIFSSFTVASVFTSFAAFAVLANTGSGDPLIGANMALDSIAAVVLGGIALSGGRGKPIGAIAGALILAISTNILAFMRVPTTYRALASGLIIIFALALSVL
gnl|extdb|pgaptmp_001531	K10441	rbsA; ribose transport system ATP-binding protein [EC:7.5.2.7]	MSLLEMTGVSKRYGGVQALAEAHLVVEKAQVHGLLGPNGSGKSTLNKVLTGTVKPDRAHIRLAGQDLQINRPLDAYHHRISAVYQQLSLIPQLTVAENLLLG<br>TEITGQGFLKSRKMRAAAEEAIAPFWPGLDEGTTLDTEVSRLSPGSQQLVEVAKAMARKPQILVLDEATASLRRDQVQLVFDQVHALIEQEVSVVFVSHRLE<br>EILALCSHATILRNGQTVATVDLATTSQPELVQLMVGEELTSAPPNRTTARGKSRDQVVLAVENLHSETLTGVDLEVQAGEIVGLGGLQGQGQSDLLHVLFG<br>DWPHSSGTISTGGRVRDIRHPRGGIGAGLALVPGDRSTQGLFMKRPILENLSIVSLPRRLRGGVFVSLRQERKVGGAEVQRLRIKIGSLDDPVSSLSGGNQQK<br>VVLGKWLLDQPQVVLLDDPTKGVDIGAKSEIYSIIRELTAAGVGVVLNSSDDEELLELCDRVLVMYEGAVVDHLIGDEITKDNLVAAALRVEEKGGDHV
gnl|extdb|pgaptmp_001530	K10439	rbsB; ribose transport system substrate-binding protein	MKSRGFVWSAVAALAGVSLLAGCSSPTPASGGNEPATESGAEGTASSAGPFTIGVSNAFVGSEYRTQMIEAIQDVFDEYKEQGLVDDLILENADADVNGQI<br>QQIRNLINSGVDAIIVDPNSASALSAVFKEATDQGILVYAIDQAVDSPEVINVGISQQDLGAANAQWFADQLKEGDKIVTVEGATGNPATDARWAGAEPIFK<br>EKGIEVLTRGDGGWDQTTGQTVATDLLATYPEIAGIWTYDGMAQGVLKAIEAAGKTDSVVTSGEARVGFMRMWNDLLPSGFSSVGIINPPGTGATAMHFI<br>INQLQGKEIDESKLVDGHTIVLPLAPAITNENFEAEWEKVKDQPDTYVLDSILSADEVAAYFK
gnl|extdb|pgaptmp_001529			MRWKGWPVPTGSRDPAKANRSALLAELLAAGPMSRVELSRRTGLSPATTNRLTAALQESGLVQEVGSDLSTGGRPSMLVQFNPDARRILAADITQDVVET<br>AAINLGGKIEERHSRAISGLSPEEKAEALRSSLTEALASWEGLPPVAVGVSVPGPVTDAGVVTLAPAVGWYDFPLGSKLADCCPGPIIVENDVNLIAYGEFFCG<br>AIPEANSLLAIGVFQGVGAGIVEHGRLWRGQGGAAGQFGRMLMDVNGLREDRKGFGQVERRLGETALRDRAVEASVLFSDDASADALFDQVERGEPRAA<br>ALFSEAMDEYAFQLVNLCAIVAPEVIVFDGLFGRWSHLVIPALSERLHENVLHEPILSPSGLQGDAKLVGAALYALDAAGGILELA
gnl|extdb|pgaptmp_001706	K03046	rpoC; DNA-directed RNA polymerase subunit beta' [EC:2.7.7.6]	[illegible]<br>WYFKGVPSRLGYLLNLAPKDLEKVIYFAAYMITEVDEAGRREDENMLRDELEVEKKRMETGRDAAINRRYEELESRLAELEADGAKPEALELERKTTDREVGK<br>LRKKWDNEIENLDRVWERFRDLKVGDLEGDEGLYRAMKTRYGTYFEGSMGAQAIQKRLRDFDLAAEVEILRDIIANGTGQRRTRALKRLKVVNAFLATGNK<br>PESMVLTKIPVIPPDIRPMVQLDGGRFATSDLNDLYRRVINRNNRLKRLLDLGAPEIIINNEKRMLQESVDALFDNGRRGRPVSGAGNRPLKSISDMLKGKQG<br>RFRQNLLGKRVDYSGRSVIVVGPQLKLHQCGLPKQMALELFKPFVMKRLVDKNYAQNVKAAKRKVERQHSEVWDVLDEVIREHPVLLNRAPTLHRLGIQAF<br>EPQLIEGKAIQLHPLACGAFNADFDGDQMAVHLPLGAEAQAEARILMLSTNNILKPSDGRPVAMPSQDMIYGLFYLTQDPDDQFADDLDEDGNVRVPEYT<br>SVAEATMAFDAGRITLNRQIKLRFTDLVPPSDWEAPEGWSEGEPIELVTTLGRALFNEALPVDYPYVNRAVKKGLLSQIVNELATRYPNVLVAESLDALKSSGF<br>YWATWSGGTISFADIQQAPHKEAILAEYEEKAAEIQRQFDMGLIGDDDRYRELVDIWTECTAKVAEDMRDNFSAQNPVYRMVDSGARGNWSQIQQLAG<br>MRGLVSDPKQKLIERPIKANYREGLTVAEYFIATHGARKGTADTALRTAESGYLTRRLVDVSQDVIVREENCGTRRGVEIVIGYPTSDGALAPSEILETTAYARV<br>LARDAVAPDGTVVAEAGSEVDAAVLDEMLAAGIEKIRVRSVLTCDSLVGTCAACYGRSLASGKRVDIGEAVGIIAAQSIGEPGTQLTMRTFHTGGAASGGSDI<br>TQGLPRVQELFEARTPKGEAKMNEAAGVVKIDDDDPSIRKIIIKRDDDKEDLVIEVSRRQDLLVRDGQHIETGTQLTEGRLDPKEVLRLRGGSAAQTQLVSEV<br>QEIYRSQGVDIHAKHIEVIVRQMMRRVTVLEPGDTNFMPGELVDSVAFREENRRIAAEGGTSAKGRQMLMGITKASLATDSWLSAASFQETTKVLTEAAM<br>NSKIDPLVGLKENVILGKLIPAGTGLARYNNVTVEPTAEAMAASNYNPIDFPAPDDTEGLEDFEQFLANMDLGPGFLSN

gnl|extdb|pgaptmp_001707	K03043	rpoB; DNA-directed RNA polymerase subunit beta [EC:2.7.7.6]	MAASSSAAFEPKTRISFAKLHEPLQPPNLLDVQIQSFDWLLGNDAWKARVEAAKANGQFDIPDVSGLEEVFAELSPIQDMGKTMSLSFQAPTLESPKCSIDEAKEKDMTYAAPMYVRAEFFNHKTGEIKGQYVFMGDFPLMTPQGTFIINGTERVVVSQLVRSPGVYFERALDKTSDREVFSARFIPARGAWLEFEIDKRDAVSVRIDRRRKLSITHFLKALGLTESEIRTEFADYPLLLETLEKDSAHTQDEALTDIYRRVRPGEPANAEAGETLLNNFYFENSRYDLAKVGRYKINKKLGLNTDPKATTLRIDDVVAAIKYMLALQQGEKTLPGMRGDEPIEVRVESDDIDHFGNRRVRAVGELIQGQVRTGLSRMERTVRERMSTQEPESITPNSLINNRPVVAAIKEFFGTSQLSQFMDQNNPLAGLTHKRRLSALGPGGLSRDRASMDVRDVHQSHYGRMCPIETPEGPNIGLIGSLATFARINPFGFIETPFRVVKDGRVTDEIVYLTADDEFGQNIAQASSPIDENGHFTEEFVLCRISGEDPNLMPASEVTYMDVSARQMVSVGTSLIPFLEHDDANRALMGANMQRQAVPLLTTEAPLVGTGMERRTALDAGDMILARTPGVVTDVSADKVEVSTDAGGRDVYKLKKFERSNPGNCTNQRVLVNEGDVVAVGDVIADGPATDHGELALGKNLLVAYMSWEGLNYEDAIILSRRIVEDDVLTSIHIEEYEVDARETKLGVEEITRDIPNISEEALAELDERGIIRIGAEVQAGDLLVGKVTPKGETELTSEERLLRAIFGEKAREVRDTSLRVPHGEEGIVIGVQEFNEDDDDLGPGVRQSVRVYVAQRRKITIGDKLAGRHGNKGVVSKILPVEDMPFLEDGTPVDIILNPMGVPGRMNVGQVLEFHTGWIAHQGWDAREAKARGEEWTKHLSEEALVADPNSTIATPVFDGLEAQELLGLLSVARPNRDGDRLIDEFGKARLFDGRSGEPFPYPIAVGYKYMLKLHHLVDDKIHARSTGPYSMITQQPLGGKAQFGGQRFGEMEVWALEAYGAAYALQELLTIKSDDTIGRVKVYEAIVKGEDIPEPGIPESFRVLIQEMRSLCLAVDALDAEGNVID
gnl|extdb|pgaptmp_000864	K06997	yggS, PROSC; PLP dependent protein	MSTPENVPVYPPAHTVADFTANLAAVRAKIEAAASRVGRDPSSVRLLPVSKTVPEERLRLAVAAGCHQLGENKVQEAKRKWQNLTDLEISWAVIGHLQTNKAKDVAAFANEFQALDSLRVAEALDRRLDRLGRSLDVYVQVNTSQEPQKYGLPPEEVRAFLHQLNQFETLRVQGLMTLALFTSDGERVRECFTLLRNLRDQIRGEDPALLGPGELSMGMSGDYEIAVEEGATCVRVGQAIFGSRALPDSYYWPGETNA
gnl|extdb|pgaptmp_001711	K02935	RP-L7, MRPL12, rplL; large subunit ribosomal protein L7/L12	MAKLSNEELIAQFKEMTLLELSEFVKLFEEEFDVTAAAPAAVAVAAPAAGDAGAEEEEKSEFDVILDSAGDKKIAVIKAVRALTGLGLKEAKDLVDGAPSPVLEGAKKEDAEKAKEEIEAAGGSVTLK
gnl|extdb|pgaptmp_001712	K02864	RP-L10, MRPL10, rplJ; large subunit ribosomal protein L10	MAKADKQAKVAELTDKFRDSGAVLLTEYRGLTVGQLKELRRALGSDVSYAVVKNTLATLAVREVGMDFLAEDLNGPTAIAFVSGEPVEAAKALRDFAKANPALVIKSGAMDGQKLSVDEVKRLADLESREVLLAKAAGAMKAKVSQAAYAFNALPTKLARLGAALAEKKQEEAA
gnl|extdb|pgaptmp_001714	K03930	estA; putative tributyrin esterase [EC:3.1.1.-]	MSIQSWNSVQSAELEMATSLALVTPGEYLPRQGPDPRLLILLHGLTGNHTQWLVRADLQEMADRHNLVIALPDGQRSFWLNQVHGLRWGAWVGAELPALLARRLRLSPDRPLIGGLSMGGYGALRAAFDYPRTFAGAFSLSGTLDVTEPAFRGRHPDLYQIGFGSAEAARPEDDLVARAAGSAELPPVFASCGTGDRLLDQNRRFAEAMGRSGHRVEYREGPGEHNFAFWTHWLPVATEWTLAAASD
gnl|extdb|pgaptmp_001715	K02863	RP-L1, MRPL1, rplA; large subunit ribosomal protein L1	MAKRSKKYRAAAERIVSGVLYTPLEALKLAQETSVTSFPSTVEGVFRLGVDPRKADQMVRGTVSLPNGTGKTSRVLVFAVGDKATQAEEAGADEVGGDELIEKVAKGYTDFDVAVATPDMMGKVGRLGRVLGPRGLMPNPKTGTVTMDVAKAVKEIKGGRIEFRVDKHGNVPFIVGKTDFTAEQLVENYAAVLDEVLRLKPAAAKGRYLLKAGVSTTMGPGIPVDPSKTKNLLED
gnl|extdb|pgaptmp_001716	K02867	RP-L11, MRPL11, rplK; large subunit ribosomal protein L11	MAPKKKVTGLIKLQIPAGQANPAPPVGPALGQHGVNIMEFCKAYNAATESQRGNIIPVEITVYEDRSFTFITKTPPAAEMIKKAAGVPKGSATPHTAKVGKLTQAQVREIAETKMQDLNANDVDAAMKIIAGTARSMGITVDA
gnl|extdb|pgaptmp_001717	K02601	nusG; transcription termination/antitermination protein NusG	MSDLEESWDLEIGEPAAEAELADAEVVEVAEELAPASEEELAPAEAAAELDPIEQLRIDVMSELGDWYVVHTYSGHERKVRANLEQRITNYHMEDKIFRVEVPMEEVIEIHNTVPKKVQRVRIPGYVLVCMDLDESSWRVVKETPAVTGFVGDQYNPVPLQIDEVVEMLTPGVLAAAQAAGVETAVAAPEPEINVDFEIGEVVTVTDGPFAELNAEISEIMPETQKLKVLVTIFERETPVELSFDQVQKADA
gnl|extdb|pgaptmp_001718	K03073	secE; preprotein translocase subunit SecE	MTDTANPSEAGKQSADRTKSGATRRRDDESFEHKRNIFQRIALFLREVIGELKKVRYPTMEEWRQYFVVVIVFVAVLMAFTGLVDFIFAKINTLVFV

gnl|extdb|pgaptmp_001720	K00812	aspB; aspartate aminotransferase [EC:2.6.1.1]	MSKEPLHRVSARLAAIAPSATLAVDAKAKALKAAGRPVIGYAAGEPDFPTPENVVEAALRAAKEPSSYKYSPAAGLPALREAVAQVTSQSSGIATDASQVLITNGGKQAVFQACAALINPGDEVLLPAPYWTTYPETIQLAGGVTVPVEGSFENGYKVSVEQLEAARTERTVALIHCSPSNPTGAVYTEEETAAIGQWALENGIWVISDEIYDHLVYGDAKFTSILTTTPELANQTVLLNGVAKTYAMTGWRVGWMTGPADVIKAATSFQSHATSNVNNIAQKAALEALTGPQDEIERMRQAFDRRRILMVDLLRALPGFEVPVPDGAFYAFPRVQGLIGREIRGRTPQNTTELAEIILDEVEVALVPGEAFGTPGYLRLSYALGESDLVSGLERIQGLLS
gnl|extdb|pgaptmp_001721			MNLVPYLSSTATPRATVLLIHGFGEHHRRYLPFISALNQRGYDVWTFDFTGHGHSPGPRARVDVARLIGEHLEARRELREVSRTEKTFLCGHSMGGLVTLASTLLDPSDLAATAVTGPALRPLPKVHPLLARFARAAGRIVPGFPTVELARDRLSHDPQVLIDLANDPLAFAGKVPLLTASSMIEQGARVIENAPMLSVPVLILHGSEDALADLEGSAEFVVAAPDQAEMIIVDGAYHEVLNELNGERSGQEIIDWYDRW
gnl|extdb|pgaptmp_001722	K01488	add, ADA; adenosine deaminase [EC:3.5.4.4]	MVADLRALPKAHLHLHFTGAMRPSTMRELAESTHTRLPAHLLEADPLKVPADARGWFRFQRSYDAARALVRSEEVMRRIVREAAEDDAAEGSVRLEMQVDPTSYAPWVGGLTPALEIVLDEAKEASRATGVQVAIIVAASRTRHPLDARTLARLAGNYAGDGPGEVVGFGLSNDERVGHTADFSAAFRIARNAGLAGVPHGGELLGPSSLREVVGHLKPARIGHGVRSTEDLDLLTRLVDDGISFELCPSSNVNLGVYATAGDVPLRQLVQSGARVALGADDPLLFRSRLVDQYRLAREAHGFSDAELAALAQGSIEASLASEADKARWKGQIRAWLAASGT
gnl|extdb|pgaptmp_001765			MTTSTPRTGTPTPERPTITIELPSGWGRAGAAGLMAALLGWVVPVAVAMVGFWAVADSPWLRQQNWQDAVALGSSFWALSLGSPAQLGLLSLTLIPLGWSLFQLLSLRLLLGRMRQFSANSIWAAIPGFVLPALVLSLSIPTEGIWWRATLGSLLIATVAAAWVFWRSGRRFPRWLQRIRPIGSGLALAFGYLGVMLVIGLVALTVSAGVHHEQMGQAAASVGAVGGQAVILWLAQLAYLPVAAVWALAWLLGATLVGVGGEVFSAAQPPTAAVPLPAWALVPTEAGASPWWVFLLVAICYSLFTWLHLRRRNLKLVALTVGLGAVVGAGLFAAWLALSTGGLGTGALAHLGPQVGPATVAFALTFLLPATVPPLLLHPSTIAACRRQISLWRQPADHVDSTTEAEEVAEAEAEAVATETGEDQ
gnl|extdb|pgaptmp_001766	K01902	sucD; succinyl-CoA synthetase alpha subunit [EC:6.2.1.5]	MTIFLDSSDRVIVQGMTGAEGSKHTRRMLGAGTNVVGGVNPRKAGTSVTFEVEGYGPLADQVGTKQVDVPVFGTVQEAKDQTGATVSVVFVPPRFAKDAVLEAVEAKMDLVVVITEGIPVADSVEFVGRALQAGVRLIGPNCPGIISPAQSNVGITPADITGPGPIGLVSKSGTLTYQMMYELRDLGFSTAIGIGGDPVVGTTHIDALRAFEADPNTEVIVLIGEIGGDAEERAAQFIAESITKPVVGYVAGFTAPEGKTMGHAGAIVSGSAGTAEAKKEALEAVGVAVGRTPTQTAELARAAYQ
gnl|extdb|pgaptmp_001767	K01903	sucC; succinyl-CoA synthetase beta subunit [EC:6.2.1.5]	MDLYEYQARDLFEAHGVPVLRGIVATDPEQARLAAEDLGAFPVVVKAQVKIGGRGKAGGVKLAHSAADAEAWASQILGMDIRGHTVHSVMIAEGADIAAEYYFSILLDRANRCHIALCSREGGMDIETLAVERPDALARVALDPATGIDLEVACKILTEAGFAPDEQERIAPVLVTLWDTYRGEDATLVEVNPLVQTGSGQIIALDGKITLDDNAAFRHPEHAGLADNRSEDQREVAAQAAGLNYVRLDGQVGIIGNGAGLVMSTLDVVALAGEPYGVKPANFLDIGGGASAEVMAKGLDVILGDPQVRSVFVNVFGGITACDEVARGIIGALELLGDSASKPIVVRLDGNRVEEGRDILQEANHPLVTMVPTMDQAAARAAELAAN
gnl|extdb|pgaptmp_001768	K03657	uvrD, pcrA; ATP-dependent DNA helicase UvrD/PcrA [EC:5.6.2.4]	MDLFDAFANSHSAAPSGPRSRAAQQAASALTVSWEGTDAFSDREQNPETLLEGLNQQQAEAVAYQGGPLLIMAGAGSGKTRVLTHRIAYLLATGRATAGQILAITFTNKAAAEMRERVGALVGPQARRMWVSTFHSACVRILREQYRAAGLKSTFSIYDQQDSQRLLGMILRAHDVDTKRFTPRLISARISDLKNELITPSQYFEQVPSDPVSEVVADAYAEYQKRLTQANAVDFDDIIMRTVQTLQAHPDVTEYYQRRFRHILVDEYQDTNHAQYVLVRTLVGVDRSALPPAELTVVGDSDQSIYAFRGASIRNIEEFELDFPNAHTILLEQNYRSTQNILSAANAVIKHNGGRRPKNLWTAQGAGDPIVVDAADSEHDEARLVVREIDDLGAKGVDMGDIAVFYRTNSQSRALEELLLRMGIPYRIVGGTRFYERQEIKDALAYLQAITNPDDTVALRRIINTPRRGVGARAEAALMAHADRYGISLGEALADAREGADRPIEGLAPRAAKSVAEFWAMMQDLRALARADEPAAHILDEVLTRSGYLESLRASEDPQDASRVDNLAELLSVAEDFDTTRREAPPEEGEQLGSLDAFLERVALVADADQVPSEGKRGGEVTLMTVHTAKGLEFPYVFVTGMEDGTFPHRRSLEDPAELAEERRLAYVAITRARQRLYLTRAATRSAWGLPEEMPPSRFLDNLPEENIERRHQTTRERLASRPGGSDGGRVFGAGAPGSLKRTFVRQTAPSSAPTRRLGGRLGESEDKPVLQLKVGDRVQHGTLGEGVVIGMEGSGKTSVARIDFGSTTKRLLLR

gnl|extdb|pgaptmp_001769			MPDNRIFSRRWAHWGRATGRWLTSGPISLAIAVVLVGVFVLSLVLPSQEVEGALRAGLGEAWWTIFTAWAAVRSLAQLVFDVLLLVTLGIALERVLGSRIFLLVGALSYWVGALLALATVKLVEVVDPRWGDLMSREAIVGVSALLLAVAAAASVRLAPLWRRRLQTFVVTVLVILVLFAGYLGALFTVYATACGLLAGALLAGREHPRTSRTELVGGGPQDGRFLVALIVSGVVVGSVMSISTTHMVGVLAHLQYLAGMEMIDPALGTCTGEVVDNCGYYQGLLSPRGLHILVPQLLQLALAWGLHRGRHAALVGTLTLQGTSVALGLANLIILWGHRDHWDVYGMNSSVVALSRLAATVAIPLIIGLIVWSTRSLFTVRTAPGTARNLLIKVGVATLVSWAVAVLVALFWAGAHDPGAAVLRTSGSFLLALLPSPSLIVDLGAAGTDPIWPVGTYLALVPWVVLGVLMLAAFRQQQLPQAIGRSQFIEMTRRFGAGSMGWIATWRGNHYWSSQTGEGAVAYRAGGGVALTVTDPVCAPERARATMEEFVAFSLEQGLVPAFYSIHQDSAEVARQWGWPVLQVAEETVLDLPTLAFTGKKFQDVRTALNRAQKEGITAQWVRWESCDSRLKAQISAISKQWVEEKPLPEMGFTLGGLDELDDPEVRILLAVDGEGRVHGVTSWMPIYQRGEVTGWTLDFMRRRDGGFKPVMEFLIASAALWAQEEGYRQLSLSGAPLARAAGSEDADSSAPLDWVLNLLGEALEPVYGFRSLLQFKAKFKPRYEPIYLTVPTLGALPGAGLAIGHAYLP
gnl|extdb|pgaptmp_001770			MNPPPRRLRLSSGRSLSWVECGCPTGPVLLYFHGAPGSALEALVLDEAARAQGVRVVAPDRPGIAGSDPEPGRTVGDWVPVVEELARALDLTEVTVLAWSGGSPYALACARARPDLVRAVGLVAPLTERENWLTRLERVSARPWLATVRVLTRVSPALIAGAFRLGGGGPRSALLAASLTRALQPGSTGAATDLAVLGRAPQPGPIPQPVTIWAGGRDAYISARQVVSLARRLGQAQVRFIARATHAQLLVDHADEIVAAIRGPNWGLNVN
gnl|extdb|pgaptmp_001282			MTIRLLVIDDHPLVRSGVRAELEGVEDFEVVGEAGDVPSAIEACHALVPDVALLDVHLPGGAGGGGPEVARACTDLPQTKFLALSVSDSPTDVVSVIRAGARGYVTKSIGSGELADAIRRVAGGDAAFSPRLAGFVLDAFGTEQVTDREDELDLLTTREQEVMRLIARGYTYKEVANELFLSVKTIETHVSAVLRKLQFSNRHELA
gnl|extdb|pgaptmp_001281			MNRPPLLRAKRHSPVRPRPVLAGVCSGVAYHLGWSVGTVRLLAFVSSLMMGGGILLYLWLWATVPREGAPTYPPVNPWELPDAPAATLRQPLPNPTSSTLNPSVPVASTQLFMVGIGILGLSMFLWLAPNILGISWLVLVWALVILGGVALVWFQALRINTSPKWQTTGFVLLGVLLIVFGLVRLMVDLNIVPKLDFGVLLGLAVAAVILVALIPLGIKVVDDLTAAKTSEVREAERAEIAAHLHDSVLQTLTLIRAGADDPVRVRSLALTQERELRAWLYTGQAEPEESVAQALKNQASEVEATYGIAIEVVTVGDAVPSPAELAAVAAAGEAMTNAARHGQPPISVFQEVRPKVLEIFVKDAGDGFDPEAIPEDRHGYRHSILGRVERVGGSVTIRQRAGTEVGIVVP
gnl|extdb|pgaptmp_001772	K01874	MARS, metG; methionyl-tRNA synthetase [EC:6.1.1.10]	MSTILSAVAWPYANGPRHIGHIAGFGVPSDVFSRYMRMAGHDVLMVSGTDEHGTPILVAADQEGVTARELADRNNELIVEDLAKLGLSYDLFTRTTTGNHYRVAQEMFKVVRDNGYLIEQTTLSAISPSTGRTLPDRYIEGTCPICGYEGARGDQCDNCGNQLDPTDLLNPHSRIDNEVPEFVETTHYFLDLPALAEVLTEWLETKEGDWRPNVIRFSQNFLEEIRPRAMTRDIDWGIPVPGWEDQPGKRLYVWFDAVIGYLSASIEWARRTGDPDAWRKWWNDPEALSYYFMGKDNIVFHSQIWPAELLAYNGKGTRGGKPGEYGELNLPTEVVSSEFLTMEGRKFASSRGIVIYVRDLLARYQPDALRYYISAAGPETADADFTWEEFVRRTNNELVAGWGNLVNRTASMIHKRFGEIPEPDALDPRDEHLLDQLAAGFGEVGDLIRTHHQKAALAAIMRLVGEANRYVTETEPFKLKSEEQQPRLRTVLWVLAQAVSDLNTMMAPFLPHSANAVDEVMGGRGDVAPMPRIDWVEDLDVTNDDGSARRYPIITGDYRSARTWERRAVVVGTPVAKPKPAFVKLDEAIIEEELARFGA
gnl|extdb|pgaptmp_001773	K08281	pncA; nicotinamidase/pyrazinamidase [EC:3.5.1.19 3.5.1.-]	MGNALLIVDVQPTFCEGGELPIDGGNACAQRIAQYADEHTDDYDLIVTTQDWHIEPGGHFSTEPDFVDTWPPHGVAGSPNAEVHSALADLHVDYAVKKGQYRAAYSGFEGETPDGTTLTEVLRSEGIDQADVVGLALSHCVKETALDARRLGLRVRVLEDLSEPVSRELGEAAVAQLREAGVCVTRSGK
gnl|extdb|pgaptmp_001774	K07056	rsmI; 16S rRNA (cytidine1402-2'-O)-methyltransferase [EC:2.1.1.198]	MNEGTIWLAATPIGNTEDASPRLRAALEQADWIGAEDTRRLKALCQRLGVTYRGRVVALHDHNEAERGPELIERAAAGETVLIVSDAGTPTVSDPGYRLGQLAIARGVRLRPIPGPSAALAALSVSGLPSDRFTFEGFVPRKEGEGRRRLEQLQADPRTQIWFESPRRTARTLALMAEILGENRPAAICRELTKTYEEVLRGTLAELSAQVGEDLRGEITLVVGGAVLDGTEPLPVERVHQLVAKGMRLKDAAAEVARQTGARKNDLYRAALATSPTE
gnl|extdb|pgaptmp_001699	K02950	RP-S12, MRPS12, rpsL; small subunit ribosomal protein S12	MPTIQQLVRKGRKRKRTKSDSPALQGSPQRRGVCTRVYTTTPKKPNSALRKVARVRLSNGIEVTAYIPGEGHNLQEHSMVLVRGGRVKDLPGVRYRIVRGALDTQGVRDRKQARSHYGAKKEKN
gnl|extdb|pgaptmp_001306	K00567	ogt, MGMT; methylated-DNA-[protein]-cysteine S-methyltransferase [EC:2.1.1.63]	MSTASAAHLSGAQLSFSIVQTPIGRLLLASGPRGLVRIAFENEDFGRILSELEAVTGLAPIGDEAALAPAERELAEYFAGSRREFSVDLDLGLTSGFRRTVVEEMGKIPYGQTSTYGELAARAGSPLAARAVGSACATNPLPIIYPCHRVVRQGGGPGGYLGGREVKEYLLQMERQNRA

gnl|extdb|pgaptmp_000342	K03790	rimJ; [ribosomal protein S5]-alanine N-acetyltransferase [EC:2.3.1.267]	MPVSPSWWERLRAPSVWGRSVEELTLDFPAPGANGLLRPDTFIRHLRIRPAWGSDHQKLEAVRRQNRLWLSPWEATLPPGSNELLPTAGEYRRRVERQMHDGESLVMVIEADGEVAGLVSISGVQRGAMSQGNLGYWIGQQWARQGVTSLAVAAVVDLVIGELGLHRLEINVRPENAASLGVARRLGLRHEGLRVRYMCIAGAWADHEGFAVDAEMLSEGGLVERRIVGRYQP
gnl|extdb|pgaptmp_000341	K03750	moeA; molybdopterin molybdotransferase [EC:2.10.1.1]	MRSVAEFYQDCLGSARQQPPLDVQLSDAVGCVLAEEVQAPFDLPVANVAAGDGYAVRTADLAGANPDHPVTLQVTTEVRAGDVSPTVLVARSAVRIASGAPVPEGADAVVALEFTDHGVASVQVRTQPAVGENIRFQGEDVERGDIVLRPGTRVGARQVALLAGVGRSRVVVHPRPRVVVLSIGDELVEPGSKARPGTVFDANGHALTTAISDAGAEVFRVPAVPDNRSILKNTIEDQLVRADLIITTGGISYGSGDTVREVLGTLGTVRFDNVAAWPGHILGVGTVGDGTPIFCLPGDPVSAQVCFEVYVRPALRQMQGWAKLTRPSVQARVDRGWYSPRGRREFVRVRLVGDPRQGYQARVMGKPASLWLSALAASNALAIVPENVTNVRAGDALQCL
gnl|extdb|pgaptmp_000337			MSPSIPSRESQSGALQDSAPPVEQTPADQPRPTRAVVDDWRTQLADTMRQELASNPETRLDLGHPHPGGLAQETALHPTRESNLPRDSDQQRVSLAAALDVLRQAQRMIDDHGSVTLYLSIGTARWLEDDARRTTPILLRPIDMTMGEDSEIYLALRPGIEISNRLLMVLSRYGQPIETADLLNLVRTRHGFSPEAALQLVREAGGAVPGFELEDTLSVGVFSHPTSALLRELGAPQWLSLSAPVRALAGDAQARGELDFEPAPPNPGDRDPWAERGLGEQPPQLADVIEAATGPNSVLVEAPAGEDYSHTVAAIAAEHAAQGRSVLVVAAQGAAQQAVADALTREGVAGVANLIGGTARSAETVQQHLQSAFLDASDDFDKDAIDEMRTRLRRRREELSSYTESLHQEFPEWGVSAFDALQVLTDLTSIPGGPTTRVRLSRASLEALAADQGQAARELLQQASSLGMFSGDVLRNWWTGVELLDDPSVEQALHAVRELSERVLPQTERDMQAVSAKTGLRLAANVRDWEAELDLLRGVRQSLDVFVPRVFERSAADMVLATASKEWRRDRGINLRGSQRRRLVKQAKDLLVPGAHVPDLHRELVSVQERRDQWRKLAAPETWPTVPADITAVLATFELLMQSLHLLNRYLEPVYGNLYELSMEELGGLMDALAADPAGARELPERVRIASRLEAMGLGELMADLQERRVDGEALSLEVDLAWWATALSMMLSQDPRLGGFDPSQLQDALGELRDLEGQQAASLGPQARSRIMRLRQQALAEIGSNYEQTQRELAVPMAAAAYYARQPISWRLMPIVITAPALVPLVVPWGRHVDVVLLAGLDEVSLPALIPVLARGRQVIAVGPGGPQQENFAELARALPKVTMTPQPQRVNDAIVGLLSRYDVGSAGISIPSRRTQGRLDLQLVDGTGMPAPGLHAIESSAAEVEAVVQRVREHAHHQADVSLAVVALNDRHAERIEAALRTAVATDTTLRGFVRAGRTEPFVVIGPEAAHGLRRDRVIVAVGYAKTPHGRVIHDFGPYSQPGGEHLLAQVLRTARGDLDLIAAFDPAEVDTERLRQPGAHMLVDLLKLGSTVQAESETPWPTLEVAPDHLLVDLAERLYGLGLNVVPNLGVPGGLRIPLGIGHPEVPGELLVAVLTDDDDYISEPSLRVRDRLIPELLEEQGWKVRIELSMAVFIDPNKEAEAIVQLVLDAVDEFYDRHPELRPEPTLIEVEAGPATAAELDLLDEEPNPMGEDQSATEDEEATEAEQEEAPTQEESLFELEDTAAHLREIKAREGRPSIAPGLPLAAYGDDQLDEVAQWIIQGAPELGGDELVEELRKTLGLHRRGAQSDAVLRNVVRRNRAALDLPEETPFQGQDEPELEVELEEPTVE
gnl|extdb|pgaptmp_000813	K01895	ACSS1_2, acs; acetyl-CoA synthetase [EC:6.2.1.1]	MTSVYYPNQETIDHAWVQDWAKVEREAAADPQAYWAAQAAELTWSQPWDQVLDDSNKPFYKWFVGGKTNIVTNAVDRHLDGPRKNKLALLWESEDGRETRTFSYFSLNREVEVMANVLKAMGVQQGDRVTIYLPRIPEVFFAMLACAKIGAVHSVIFAGFSADALTSRIDDSESKIVITADGSWVNGSIFPLKEIVDEAVRFSPTVENVVVVRRTGTEVTMDPLRDHWYHDLTALPIARGRCETVQVDSEHPLFILYTSGSTGAPKAILHTHGGYMVGTYTTLRNTFDIHDEDRYWCTADAGWITGHSYLIYGPLLNGATIFMYEGGPTYPHPDRWWSLVEHFGITIMYTAPTAIRSLMRFGDAWVNRHDLSSLRLLGSVGEPINPEAWRWFYEVVGQGQAPIMDTWWQTETGIFQIAGVPSMPQKPGSAGHPVFGQEAEVVDADGNPVPDNTEGFLVLKHPNPAMMRTIYKDDQRYVDTYWSLTADRYVTGDAARRDEDGYFWVLGRTDDIIKVSGHRLGTAEVESALASHPAVAEAAAIGLPHEVKGNAIHVYAVLNPGFAGSREMTEDIRAHVSHHLSPIAKPEEIIYVETLPKTRSG
gnl|extdb|pgaptmp_001486			MSKSLAQAGPFARERVLYPLTARSVHVRQTSRPAPHFIRLTVAGADLEGMKAHGPGDHLRVFFPDPASGILNAPSTGPDGLIPPPAPGLHRDFTPLHLRTGADGVPELDLDFFLHHHPGPAAQFATRAEPGDRLVLVGPRGSITAPPGAERLVCFVDETALPATARWLQLTPPSTAVEVYTRADRWVETYLADNGGRGERVHPLVTDLVSAARAAHLGRSTYVFAAGEASELATLRRFILKELGLAGEQCDFSGYWKRGVSEYDHHAPLD
gnl|extdb|pgaptmp_000335			MDQTEIVVGVDGSRESLDAALWAGEHAKRVNGHLTVVCAYPTASYSAAALDGGFAVVDDESLHQGALDAANEAAAAVREQVGVDPEVSALVGDPSIVLAELSKECDLIVIGSRGRGGFADRLLGAVSSAVPAHSKCPVVTVPPHRSGKPFTPIERIVVGVDGSDQASTALVKAVDLAFAWQAELTAVVAIPVATAGGAMAWLPVAVDRQVLLDDIMESLNSAIEKALNGRDMWVARHVLDGSPAALLTEFSTAVDLVVVGTRGRGGFAGMLLGSTSQTVLHHSTCPVMTVPSRHRDRRPSP
gnl|extdb|pgaptmp_000334			MDLTQTSEFGRITLRRAAGVTATGIMVTAGLAGVANAAPTLESEAKPKLADPSVNLTDQSVGTIVSLDKAWDPGDEVSVAVKAPVEVAEPVEEEVEEVSRSEEREELVEEPVYVPSGSVNASSVVEAAYQLLGIPYAWGGESMAGVDCSGLVKMAFAAVGVNLPHSSDGIAAAGTMIPASEAQPGDVVAYPGHVAIYVGNGMMIEALDYGYVSQLSPVRGGGWFVRI

gnl|extdb|pgaptmp_000333	K26252	dtxR, ideR; DtxR family transcriptional regulator, iron-dependent repressor	MASFDLIDTTEMYLKTIYEMEEDGVTPLRARIVERLGHSGPTVSQTIARMERDNLVTVRDDRRLELTDEGRGIAVEVMRKHRLAERLLVDVIGLSWTQAHDEACRWEHVMSAAVEDRLEDLLAGPEFDPYGNPVPGRGVSNATEVNIETALEGKDEVTGTIVRIGEPIQAEPWLLKKFEELSARPGAKVWIRRVPSGYQIGLDRPEGGREPLVLEPVFAAHLFVTLP
gnl|extdb|pgaptmp_000332	K00831	serC, PSAT1; phosphoserine aminotransferase [EC:2.6.1.52]	MMTQELPPRSLWPRDGRFGAGPSKVRPAQIDELGQSNLWGTSHRQAGVIGLVQSIQQQLRELFSVPEGYEIVLGNGGATAFWATACVSLIREWGNFAVFGEFGGKFAADGRSAPWMNATVDEAPAGELAIMNTALPGGLGLGPDAYCYPHNETSTGVVSPLYRAPDPEALTLVDATSIAGAATVDLSLVDAYYFSLQKCFGADAGLWVAILSPRAVARAEELNRLSSRPQFGILNLTSAIRSARKGQTVNTPAIGTLLLVHSQLEWMLAEGGLAEMAARAEAGADAIIRWAESRPWATPFVEVPAWRSPVVSTVDLAEEISAGELAARLRPAGIQDIEGYRGLGRNQLRIASFPSIDQADIDSVLACIDWAVEH
gnl|extdb|pgaptmp_000331			MSKTFHSLKYPNFRLWIAGNVVASTGTWMQRVAQDWLVLTVLTDGAGSQLGIVTALQFLPLLVLSPWMGALADRVNRRTLLQITQASTGILGLGLGLLVLTNQAELWMVYCFALAGGVASAADSPARQAFVSELVPPTSLANAVGLNSAAFNTARLIGPAVSGLIIEWVGMGPVFLINAGLFLMPVLTLALMNPDLLRRPPLVPRAKGQIREGFAYVRSRPDIVSIMVIMGMVSAFGLNFQMTSAMMATEVFGKSAGEYGMLGSYMAIGSLAGALIAARRARPRLRLIVGAALIFGLFEAALALAPSYFWFAVMSAPTGLASLTLITAANAAVQISTAPEIRGRVMSLYMMIFMGSTPLGAPIVGWIGEQFGARWSLGIGAIACVVVALLVGVWGKVHWDVHLQVDRSPYRLRAIGPVERAGLDPITLKPLSDPPEQQAQRGD
gnl|extdb|pgaptmp_000329			MPENEINEPAPKLDRSVRRDELLLEAVEVARQAAVEISRPESVGEHVGARMVAKGVAAHRFHCQDAGYPGWDWEVSVARAPRSKTVTVCEVSLLPGEGALLAPEWVPWEERLRPGDVSREDVLPYSANDPRLMTNLEQTDPELVDELGVEELGLGRPRVLSEYGIQQAADRWYASDQGPVPAAKQTCATCGFLLKLPGSLGRVFGVCANEWSPDDGRVVSLDHSCGAHSETDTTKRKPQWPMQSSRIDEFQITVDDLRQ
gnl|extdb|pgaptmp_000328	K03704	cspA; cold shock protein	MPTGKVKFFDVDRGFGFIGGEDGREVFLHASALPDDLPAPRPGQRVEYGIADGRRGPQAISVRALSALPSVARAQRRKPQEMVPVVEDLIRLLDSSSDSLRRGKYPDNAGKIAKVLRAVAEDFSA
gnl|extdb|pgaptmp_000327			MKTKHNWLARLGGAATVGLATVLLGVAASAEPPLAITDRVTDPNGFLDASQTQEAHDALSAAAEDGLALYAVAVPTFDGQDATGWCLQTGEQSHLAGDSVIFAVAYEQRDAAWCTGADSNQVSDSELNRAFEAARSELGRSNPLQPEDLTAAIVTFGERVSASANGSSSGSSVGALVWVAIAIMIAAVLAVVVSSSRRGARLRKAGLGPKASPAAQQKLVDESSQQLLYADEALRRSEDELSFATAQFGTLETKSLATAIEAAKPVLQQAFEVLSRMNDEPNLAAKAQLAGQIQELVGRVMPAVATAQKVLKDRRERERTAEQQAAQLSEQIGEADRRLEAARAELQSLQQHFSASSLASIANNPDQAEAELTEAREHVAQVQQLVATDRTRAVQELDLAATALAQALRLIETVTGARTALEQSAQVLTEAIASISSDLDDVARLSADRASFAPLVDEAKAAIEAGIAARSGQGDPLSAIERLRLAEDGLDRALAPLRSAHDQFQKETQTAQQRIAGAQTLVLQAQAQLQAHGAYATMEQRNSVAKAQSLLTSAQSLLESNPGQAAQQATQAESFARSALATAPQPAGRRSSGMNMSDVFLWSMILGNMGGGRSSGGHSSGWGGSSGSSWRGSSGGFGGGGGFSGGRGGSSGF
gnl|extdb|pgaptmp_000326	K03969	pspA; phage shock protein A	MAQKESVFGRVSQLLKANINALIDKAEDPAKMIDQLIRDYTNEIVEAEKAVAQTIGNLRLAEKDHEADVAAANDWGQKALAASNKAEECRASGDTAGADKWDGLAKVALGKQISFEQEAATAEPQIATQQQTVEQLKAGLSQMKERLSDLKTRRDQLAARQKTAEAQAKVNDAIKSINVLDPTSELARYEEKIRRAEAQVQGQMELAGDSLEDQFAELQTDASQLEVEARLAALKKKD
gnl|extdb|pgaptmp_000325	K00864	glpK, GK; glycerol kinase [EC:2.7.1.30]	MSEEKYVLAIDQGTTSSRAIIFNHSGEIVSVGQQEFEQIFPNPGWVEHDPIEIWESVRAVVADALQKAEINRHSLAAVGITNQRETAVVWDKTTGLPVYNAIVWQDTRTSKIIRELAGDQGMDRYRSKVGLSLATYFSGPKVKWILDNVEGAREKAEAGDLLFGNTDAWVLWNMTGGVDGGVHATDVTNASRTMLMDLKTLQWDPEICADFGIPMSMLPEIRSSSEVYGYGRKQGLLIDTPIAGILGDQQAATFGQACFTPGMAKNTYGTGCFMLMNTGEEVVFSDNGLLTTLCYKIGDQKAVYALEGSIAVAGSLIQWVRDNLKLIESAPEIEEVALTVEDNGGVYIVPAFSGLFAPYWKDDARGAIVGLTRYNNRGHIARAALEATAFQTREVLDAMEADSGAKLSELRVDGGMIANNTLMQFQADVLGVDVVAPQVAETTALGAAYAAGIAVGYWDGEADVIANWAEGKRWKPQMDKTEVERTYRLWKKAVTRTFDW
gnl|extdb|pgaptmp_000324	K02440	glpF; glycerol uptake facilitator	MNAFEIFASEFFGTMLLIILGTGVVANNLLPKDKGKDTGFLMVNFGWGLAVFVGVYGAWKTGGHLNPAVTIAKWVATFYDPNVTLNGQPMGSEFAVPATASNIALYLVAQFLGAFIGAIIMYLAYKKQFDEDAPAAHKLGVFSTGPEVRSYGWNLVTEAVGTFILIVFVLVAGGTPTAVGPLAVALVIVGIGASLGGPTGYAINPARDLGPRIAHAVLPIKGKGDSDWGYSWVPVVGPIIGAVVAVVVTYALSLSSLDFWPL

gnl|extdb|pgaptmp_000322			MNVRDELAQEAATLYYLQDQKMESIASKLGVSRSTVSRLLSYAREVGLVRISITAPPGTSDTLAQKFEETFGVHTWVVPVGSADTDLNRLHNVSAVAAERLINMLFPGATLGIAWGNTTSMIAQNIPRVDLPGLTVVQLNGASNAAESGLPYAQAIITQAAQALGAKMVNFPVPAFFDFASTKEAMWRERSIQQVLQTIDSCDVALFGVGSLDGSIPSHVYSGGFLDQKELDQARRDGVVGDVCTVLLRRDGSTDMKLNARASGPTPAQLQKIPRRLCVAAGDSKALPLVGALRSGVATDLVIDSSLARVVLNQLRLERRPARRIVQ
gnl|extdb|pgaptmp_000321			MTQQPPLFPLPEGALRPQARVIVLTGPSGSGKTSLTNRLGFPSLSLDNFYRNDDEEGMPRLAGGLIDWDDPASWAQDEAMEALSTLCLTGRARVPIYNIPTNQRTGMEQFDLGGQQVVIVEGIFASLLVEPLRQEGLLLGAVCIARSPMRNAWFRLARDLGEARKPVPVLLWRGFKLALSEPKQVQRWIGHGCEPAKSLGAADRRIREIVASAHIPPPPI
gnl|extdb|pgaptmp_000319	K04567	KARS, lysS; lysyl-tRNA synthetase, class II [EC:6.1.1.6]	MSTNENQPAVDPALADAPEQFRVRSAKRQRLLAAGINPYPVEVPITATIAELRDKYAGLEAGEETQDYYGVAGRVVLARNSGKLCFATLQDGAGNRLQVMLSAREVGVESLTQYKADVDLGDHLFVHGKMIASRRGELSIMAEPAEGIPAWQLASKALRPLPKTYEAEDGTEVSLSDDARVRRRYLDMIIRPAARNMVRTRAAIVRSLREYFHESGFIEIETPMLQVVHGGAAARPFSTHMNAYDTELFLRIAPELFLKRAVVGGVEKVFEINRNFRNEGADSSHSPEFTMLEAYEAYGTYDSMARRVQEMVQKAARDVFGTEQVTLSDGTVYDLSGQWHSISLYESISEKLGRKVDAQTPRAELLAIAAELGEEIPDHYVDGKIAETIFEVLVGDELYEPTFVRDFPEDTSPLTRGHRSIPGVTEKWDLYVRGFELATAYSELVDPVIQRERFEAQALAAANGDPEAMVLDEDFLEAMEYAMPPAGGMGMGLDRLIMALTGEGIRETIT
gnl|extdb|pgaptmp_000318	K01579	panD; aspartate 1-decarboxylase [EC:4.1.1.11]	MTEFPVYREMVSGKIHRARVTGADLNYVGSVSIDSDLLAAADILPGQKVDVVDVTNGARLTTYTIVAPAGSGTVQLNGAAAHLIHQGDIVIIIAYAHLPEELARTREPRVVLVDANNRQVEVGHDLGR
gnl|extdb|pgaptmp_000313			MDSLRAVGADAPESALEDEAEFLVHAWNVPERTAHNVKYLSSVIERLADFDGVTADPDVLKLAAVYLVTSTVNTWEALGGWESARPVEPIHAEERLRALGVPGPTADRVASLVRQLSSRKLPVDDLEAQILFDALLATIGTTPQRYARFRDQLAREAHGEKDPRYLRARKRFITKILGRSRLFLTPFAAPWTEPVRENLLGELSQINALLGEEDGAGDVVRGEDTGPLLIRGAADRLRAARKGAAERAAREAVPVVRQPAAEKAAARTADPSEDQNPDDTSTLEAFDDPFSHRRSKRH
gnl|extdb|pgaptmp_000312			MMAIYQVDSGQVASAAATASQSSATIQSEVAQMMSLLRGLEASWGGAAALTFQGLIEQWQLTQLQVEESLRAISTQLAQAATTYAEAEDAAAALFHG
gnl|extdb|pgaptmp_000311	K04077	groEL, HSPD1; chaperonin GroEL [EC:5.6.1.7]	MSKFISYDEEARRGMENGLNKLADTVKVTLGPRGRNVVLDKKWGAPTITKDGVSVAKEIDLEDPLERIGAELVKEVAKRTDDVAGDGTTTATVLAQALVHEGLRNVAAGSNPIELKRGIDKAVDAIVAQLHKIAQPIETSDQIASTASISANDPEIGKLIAQAFETVGPEGVITVEETNSFDTTLETTEGMRFDKGYLSAYFVTDAERQEAVLEDAYVLLVESKISSMKDLLPVLEKVMQTGKPLLIIAEDIEGEALATLVVNKIRGIFKSVAVKAPGFGDRRKAMLQDMAILTGGQVISETVGLTLEGADLDTLGTARKVVVTKDETTIVDGGGEKEALDARVKQIRAEIEATDSDYDREKLQERLAKLSGGVAIIKSGAATEVELKERKHRIEDAVRNARAASEEGIVAGGGVALIEAAKGVLGDLDVAGDEKIGVAIVRQAVESPIKQIAENAGIDGGVVVDRVSRMESGHGLNAATGEYGDLLTEGIADPVKVTRSALQNAASIAGMFLTTEAVVGDKPEPPAPAAAGGMDDMGGMY
gnl|extdb|pgaptmp_000310			MSSTYPADEFEHPGEDLPVGAHRQPPSRWRPVIPFLVILVVVPLLAWGVSYLLQRRDAGDDAAQSAPPAVTQQSGQSDARPTPSPTPTPTQPSAAPTAPDETKPEEEENPELGIDYALTIEVLNATDISGYAGQIAADLEAAGFTSVIADNTSGWITEVNTVFFTSAEQEATAHQVASIAGIDSVVLDPDATGGEGIVVLLVE
gnl|extdb|pgaptmp_000309	K03648	UNG, UDG; uracil-DNA glycosylase [EC:3.2.2.27]	MQAGPRPLTDIVDPGWARALAPVADTIAELGQMLRAELRDGQKYLPAGTDVLRAFTYPFDEVKVLLVGQDPYPTPGHAMGLSFSVAPGVEIPASLRNIYRELSDDVGPIAPTGGDLTGWARQGVCLLNRVLTVRPGQPGSHRGRGWEAVTQRAIEALVERDRPLVAILWGKDAQSLLPLLGSTPAITSVHPSPLSAYRGFFGSKPFSRANELLAQQGADPIDWTDLPVS
gnl|extdb|pgaptmp_000307			MPQSLQIKAANVDPALLDLPWEIPLEEWPEDILAALPRGISRHVVRFVNLSGRVIAVKEIGETVAGREYELLWDLARLGAPSVMPTAVVTGRRDLAGEELNGVLITEHLQFSLPYRALFRQSLSPETATKLIDALAVLLVRLHLLGFYWGDVSLSNTLFRRDAGSFSAYLVDAETGELHPQLTDGQRDYDVDLARVNIIGELMDLQSGGYLDEVDDVISIGTRIVERYEALWSELTAPELIESDQRWKVERRIERLNALGFSLGELTMSSDPGSQRLTIQPKVVDAGHYHRRIMRLTGLDVGEEQARRLLDDLDTYRAVHQLSNVPEEIVAHQWLREAFEPVIAAIPTNLTGKLEPAQVYHEYLEHRWFMAEQRQQDVPQEEAIASYIENVLSKKEDELLFLDSELLGLADPDD

gnl|extdb|pgaptmp_000306	K10112	msmX, msmK, malK, sugC, ggtA, msiK; multiple sugar transport system ATP-binding protein [EC:7.5.2.-]	MASVTFDNATRIYPGSDRPAVDKLNLEIADGEFLVLVGPSGCGKSTSLRMLAGLEDVNSGRIFIGDRDVTDVQPKNRDIAMVFQNYALYPHMTVRDNMGFALKIAGRPKDEIRQRVDEAAKILDLEPYLDRKPKALSGGQRQRVAMGRAIVRKPQVFLMDEPLSNLDAKLRVQTRTQIAQLQRDLGVTTVYVTHDQTEALTMGDRIAVLKDGILQQVGTPRDMYDRPANSFVAGFIGSPAMNLGTFTIDGKVARLGTATVPLSDETLAAVTPEDNGQIVIGFRPEALRLVASDADGVIPIKVELVEELGSDAYIYGQLAGDAGDAKLGSGEEGGGRQLVVRVPPNSAPPAGSVIHAEIAPGQQHNFSAATGVRLP
gnl|extdb|pgaptmp_000304			MGQKSSKRTEDSVQAKAKAMREAQAKADRRTRNMIIAAVSIIVVAIIAALVFVVTNEGSKNPSPSNEAIPTQFENGEPIVVSHLGVGVRDENLPDLEEYFSYTCSWCAYLDSAVGEKFAEAAKNGEFNYILQPVNTAYMPFQGPATFASLQVAANAPDQFLAFHQALTDYFYGQLQAQDQSVIGNADSSQAQVVTLAQQVGVPEDVINQFGGTAEAYLEKTTKAWTEADVQGREGSLGTPELVFDGSKVAWQQGTPDEIWAGILESLKALGFTPAN
gnl|extdb|pgaptmp_000002	K01372	BLMH, pepC; bleomycin hydrolase [EC:3.4.22.40]	MSPALTPELIERCRSVVDSNSAARLSRNAVTLSGLDAVSLDRAKATTPTSMSTKLDKWKVTNQKKSGRCWLFAALNLMRVDAMEKMGLKDFQLSQNYAVFWEKFERSNYFLEDQISLAEAGEPLDSRLTQFLLADVLNDGGQWDMLVGVFEKYGVVPQEAMPETEASSNTGRMNSQLRLLLRRVALELRELPTVEARRELKAKTMEEVYQILVINLGVPPRAFDWQWRDQDGQFHRDGELTPLEFKAKYVSLDLTEYVCLVDDPRTAHPKNAPLTVDHLGNVVGGPSVLYLNVDISVMKELAARSLRDGHPVWFGCDVGQQSERKAGLLVADLYDYQGVLGVDLTTTKEQRVLMGESLMTHAMVLTGVDEVDGQPRRWRVENSWGDEIGDQGFFTMDDQWFSDYVFEVVVKKAELPEQLRAALTQEPLVLPAWDPMGALA
gnl|extdb|pgaptmp_000921	K09861	uncharacterized protein	MLIWLPPSEGKLAPTAGPALGPLSFPELSSARAEVAEALIRVSASPEAGKILGLGPKSVSEAAANLELWTGPCAPAAELYTGVLYANLLPYPAAFPTSTWIFSGLFGVVRPGDYLPNHRLSMNVSLPGLGPLATWWRDRLLPLLPEGETIVDARSGPYRRALPARSAHVIEVHPPAGVSHQAKAYRGQVARYLLERLPAEAGLAETLACLESVCELGPTKATAAGGSLTQVRAIRYSVSSEM
gnl|extdb|pgaptmp_001093			MKTVKLTTEPFSCPSCVAKIERLLAQTPGVESSQVMFNSSKVKVTFDEEQTSAQQLADAVTKLGYPVLNIA
gnl|extdb|pgaptmp_001094	K01534	zntA; Zn2+/Cd2+-exporting ATPase [EC:7.2.2.12 7.2.2.21]	MNQKVRTRLAFVAGALLVVGLPLHWWVSPQWASPVLIAASLVAGTPIAIKAWQALRVRAFSIDLLVTIAVVGALIIGEYVESAVVSFLFLFGAWLEARSLERTRSSLRGLIELAPTIATVEREGVRQEIPASELEVGDLIVVRAGERIAVDGVVAEGEAEVSEASITGEPIPVGKRRGDHVFASTIVETGFLQVRAKEVGDDTTYARIIELVEEAQESKTRRQRFLDRFSQYYTPAIVVGAIVAFALTRDLSFALTFLVIACPGALVISVPVAAVAALGNIARHGVLSKDAQGLEDLAGADTLVLDKTGTLTQGQPSVTGVHPRSGVTSDQLLTWAASVELASEHHLGRAVVRAATERDLALTNPTAVEVWAGLGLGATVDDHRVLVGSERLLAEQGLALSEPVAASSGATAVLVAVDSAVLGWLEVSDPIRPEAQAALEHLRQTGFKQIVMLSGDRPEVAAQVGAELGLDRALGGLLPGDKAEVVADLQREGRRVAMVGDGVNDAPALARAQVGVAMGRSGTDVSVETADLVLLTDRLDQLAHARRVSQLAVRIMKENMALALGTVAFLLAGVLFHAVHLAGGMLIHEASVLLVVLNALRLTSLRMPASQP
gnl|extdb|pgaptmp_000296	K01624	FBA, fbaA; fructose-bisphosphate aldolase, class II [EC:4.1.2.13]	MPIASPEVYNEMLDRAKAGKFAYPAINVTSSQTLTAALQGFAEAESDGIIQVSVGGAEYWSGSTIKDRVAGTLAMAAYAREVAKNYPVTVALHTDHCAKQNLDSWVIPIMELEAAEVAAGREPFYQSHMWDGSAVPLDENLEIAQKMLKLAVASRTILEVEIGVVGGEEDGVVGEINEKLYTTAEDGLKTVEALGLGENGRYLTALTFGNVHGVYKPGHVKLRPEILGEIQTAVGAAHNAGDRPFDLVMHGGSGSTEEEIALAVANGVIKMNVDTDTQYAFTRPVADWMYKNYDGVLKIDGEVGNKKTYDPRAWGKAAEKGMADRVLEACQRLGSVGTKM
gnl|extdb|pgaptmp_000295			MNDATHLPGEPEREVGVGPWTGPWPEGDHYDPDLLEAGDRRNVEDQYRYWRREAIQEAIAAKSLGLEVAIENLGHDFNIGSIVRTANALGVSAVHIVGRRRWNRRGAMVTDRYLPVDHFPEVDSFAKDADLRGRILIGIDNLPGSKPLELADLPREATLVFGEESAGLSPQMRDRCREIYHITQYGSTRSMNVGHAAAIAIWAWVIQHGPRQKQHLA
gnl|extdb|pgaptmp_000292	K01142	E3.1.11.2, xthA; exodeoxyribonuclease III [EC:3.1.11.2]	MRIATWNINSIRTRVDRVLAFLERSEVDVLALQETKCRDDQFPTAPFEEAGYHLAVHGLNQWNGVAVISKYPILEVQIGFPGQPEFREVVEARALGVTVDSPAGPLTVWSLYIPNGRELDDPHYTYKLDWLAQLKSAATGWLTTDPNALIALVGDWNVAPEDVDVWDMGFFEGKTHVSPPERAAFAAIGEVGFTEVTRPLVTNYTYWDYQRLRFPKNEGMRIDFVYASPALAKRVSAAEIDRNERKGKGASDHVPVIVDWD

<table>
<tr><td>gnl|extdb|pgaptmp_000293</td><td>K07124</td><td>uncharacterized protein</td><td>MGTALVTGATSGLGREFCWQLAAAKHNLVVVARNESRLEELAEELRQVAAIKVQVLRADLAEPEDLARVCQRLEQNEPEIGGPVGLLVNNAGFGLGKRFLNDTLDRELYGLEVMVRAVMVTSHFAGRAMVDRGHGAILNVSSVAADTGMGTYSAHKAWVRAFSEGLAEELHGTGVTCTVVCPGLVQTEFHERMGTDMSEVPDFAWAPADQVVSQALDAVRRGQVIVTPTALYKVVNGAARVAPRGVVRAVTRLLPHV</td></tr>
<tr><td>gnl|extdb|pgaptmp_000291</td><td></td><td></td><td>MEIPFPHALVAIDQAGEPIWQEGDLSQPFPFASVTKLLTALGVHLAVQDGYLRLDQPAGPEGSTVAHLLAHASGLAPEGEGTRMAARVGSRRIYSNQGYEVLGEVVTAATGDPDWTRNRLLAPLGAEGITIAGTPAWSAVGSALDLSLVLQELLQPELLSRASWEAFRSPAFPSLRGVLPGYGTQERNLWGLGPEIRDRKHPHWTGTRADPSTFGHFGVSGSFLWVDPTRAVGALFLGQERFGDWHKANWHQLNDRLIELAEAR</td></tr>
<tr><td>gnl|extdb|pgaptmp_000290</td><td>K03695</td><td>clpB; ATP-dependent Clp protease ATP-binding subunit ClpB</td><td>MSQNLTTKSQEALSSAVQMASAVGNAQVEPLHLLAALLEQPEGIAISLLAAVGAQRGEVGAGTRTALVALPASSGSSVTQPQTSRQLMNVLTASQKLAESRGDAYVSTEHLLIALAGSDSEAGRILRQNGATAEELEAALAKLRPEPVTTANPEGTFQALSKYGRDLTEVAREGKLDPVIGRDAEIRRVIQVLSRRTKNNPVLIGEPGVGKTAVVEGLAQRIVEGDVPESLRGKRLISLDLGAMVAGAKYRGEFEERLKAVLEEISNSDGEIITFIDEMHTVVGAGASEGSMDAGNMLKPMLARGELRLVGATTLDEYRENIEKDAALERRFQQVFVGEPSVEDTVAILRGIAPKYEAHHKVTISDGALVAAATLSDRYITGRQLPDKAIDLVDEAASRLRMELDSSPIEIDELQRAVARLKMEETYLRESDEDDRDPATQERLVKLQEDIANTEEQLQGLTLRWEAEKAGRNRVGDLRAQLDELRTAFELALREGRYEEAGRIQNGEIPAIERQIAEAELADAEEAEHAAEPMVADKVGPQQIAAVIESWTGIPTGRLLQGETEKLLEMEAWIGKRLIGQHPAVQAVSDAVRRSRAGVSDPNRPTGSFLFLGPTGVGKTELAKSLAEFLFDDPRAMIRIDMSEYSEKHSVARLVGAPPGYVGYEEGGQLTEAVRRRPYSVVLLDEVEKAHPEVFNILLQVLDDGRLTDGQGRTVDFRNTILILTSNLGSQYLIEPLLSPEEKREAVMGEVRRNFKPEFINRLDETVVFEPLTKHELGQIVDLQVAEMAARLESRRITLTVTDEARAWLEDHGYDPAYGARPLRR</td></tr>
<tr><td>gnl|extdb|pgaptmp_000288</td><td>K07009</td><td>gatD; lipid II isoglutaminyl synthase (glutamine-hydrolysing) [EC:6.3.5.13]</td><td>MSESTVRIGVLAPEVLGTYGDSGNALILAERARRRGFPAEIVTVNLSEPIPDQLDLYSLGGGEDTAQALAADHLRSERGLSRAVAAGRPVLAICASLQILGRIYVDAAGNEISGLGLLDLETHPRGERAIGEVVTRPLLPELTQLLTGFENHGGGTRLGPDARPLGEVLAGTGNGFDRVEGAVQGSIIATYLHGPVLARNPELADLLLSQALGQDLAPLELPAIDQLRQERLSDAGVDPAVN</td></tr>
<tr><td>gnl|extdb|pgaptmp_000287</td><td>K23393</td><td>murT; lipid II isoglutaminyl synthase (glutamine-hydrolysing) [EC:6.3.5.13]</td><td>MTRPLTFGRRLRGAVATILGGSARDLSRLLGRGSGGMIGGRVALAVDPSVLATLAHDKQAVIVSGTNGKSTTTKMVRAALGTAGTVASNTLGDNMPPGVATALMNEPRAPFAALEVDEMYVPQVAAEVRPAVLVLLNLSRDQLDRVGEIGAVERRLRQGVEENPQAHIVANCDDPLIVSAAWNAPHVTWVAAGSSWGADSTTFPRTGGQVTRTEDGWFVDEQIRRPDPDWWVDQDSRLHGPGVELPLKLSLPGQVNLHNAAQAIAAAVELGVPAAQAAEAVGQVRDVAGRYQEFDVDGRGVRLLLAKNPAGWQEALAMVDPTVNQVVIATNGQIPDGVDLSWLWDVRFEELPQPERLFASGDRAADLAVRLGYAGLEAKLVPDPLSAIRQCQPGPVEVVANYTAFRDLLPQLKQIDRAGRAQ</td></tr>
<tr><td>gnl|extdb|pgaptmp_000285</td><td>K03686</td><td>dnaJ; molecular chaperone DnaJ</td><td>MGEQDWFNQDFYQTLGVKKDADSKEIKKAYRKLARQWHPDQNPGDLAAEEKFKQISEAYSVLSDPEQRERYDAIQQMAQGGARFTPGSGGGGGFEDMFGSMFNGAGRGGTYQFQTSGGGGGFDDILSSLFSGGGRGGRGPGAGYSSFGMPAQAGADLTAQTTLSFRDAYLGTSIRLKVGERTLTANIPAGVRDGQKIRLRGKGQPGSGGGPAGDLVITVQVKKDPLYSVEGNNLRVRLPISFPEAVLGAQVAVPLPDGTSVKVKVPAGSSSGRVLRVGGRGMHRGKKRGDLLVELSIHLPAPDTTELKELAEQVQAAQGEWDPRANLGGER</td></tr>
<tr><td>gnl|extdb|pgaptmp_000284</td><td>K03687</td><td>GRPE; molecular chaperone GrpE</td><td>MSQDAVNPDEFAGETPENSGGPGRPESTGADQTAPAGSPEQEAVVPVEQDPNAQLEATVAELEQAKEDLARARADLYNLNQEYGNYVRRAKSEGTAQRQLGQEEVAEALLGVLDDISAAREAGELGTGPFASIATKLEETLASRFHLERYGAEGDPFDPQLHEALMAQTNPDIEHPVIKQVLQPGYRINDRVLRATKVMV</td></tr>
<tr><td>gnl|extdb|pgaptmp_000283</td><td>K04043</td><td>dnaK, HSPA9; molecular chaperone DnaK</td><td>MAKAVGIDLGTTNSAIAVLEGGEPTIIANAEGARTTPSVVAFSKNGEVLVGEIAKRQAVTNVDRTITSVKRHMGTNWSTQIDDKTYTAQEISARILSKLKHDAEQYLGDQVTDAVITVPAYFNDAERQATKDAGQIAGLNVLRIINEPTAAALAYGLDKGKEDELILVFDLGGGTFDVSLLEVGKDDDGFSTIQVRATSGDNRLGGDDWDQRIVDWLIQQVKNKTGVDLSKDPVALQRLKEAAEQAKRELSTATTTTISLQYLSMSTEGPIHLDEKLTRAKFEDMTSDLLERTKRPFQDVIREAEISVKDIDHVVLVGGSTRMPAVSDLVREMTGGKEPTKTVNPDEVVAIGAALQAGVLQGDRSDVLLIDVTPLSLGIETKGGVMTKLIERNKAIPTRATEVFSTAEDGQSSVLIQVFQGEREFTRDNKPLGTFELTGIAPAPRGVPQIEVSFDIDANGIVHVSAKDRGTGKEQSMTITGGSALPKDDIERMVREAEAHAEEDKARRAEQDTRNLAEQTVYSMEKLLKENDEKISEGTKTEVQAAVDETKKALEGEDIDAVKSALDKLNEVGMKIGQEVYAEAQASAAESDLPSQDDVNAEEEVIDAEIIEDEEDK</td></tr>
<tr><td>gnl|extdb|pgaptmp_000281</td><td></td><td></td><td>MTTELVTTVTPEIVAALDHLLPQLSSSAPTLDAEGVSALIDQDGVYLFAYRNEPGSPILGLLTLATFRIPTGLRAWVEDVVVDEAARGHGAGQQLVEAAIEQAFSLGCRTVDLTSRPSREAANRLYQRCGFELRETNVYRYAR</td></tr>
</table>

gnl|extdb|pgaptmp_000280			MTHTANYLDYLNASPSPFHAAHEAGRQLAEAGFVEVDERQQWPSEPGSYFLIRSGALAAWIIPPQVSQTAGFAIFGSHTDSPCFKLKPTPDHLSPDGWGQLAVEVYGGMIWNSWLDRELALAGALYDRSGQAHLVRTGPLARIPQLAIHLDRTVNEGLKLNPQQHLRPVWTVDQPTASIGALLAEATGLPEEEVASADIFLVPSQGAALFGDREQFVAAGRQDNLSSVFAGLTALKATAPNPEVIPVLVAFDHEEIGSATRTGAAGPFLEDVLRRTAGAVGATGDQFWQLMARSSCISADAGHSVHPNYAQLHDDDTRPLLGRGPLLKINANQRYASEGQGISLWHRVCAAAGVESQDFVSNNAVPCGSTIGPITATRLGLLTVDVGIPLLSMHSAREMSHVA
gnl|extdb|pgaptmp_000272	K04762	hslR; ribosome-associated heat shock protein Hsp15	MDKLRVDIWLWATRQLKSRSLATSAARAGHVRINGEPAKASAPVRVGDEVRLRIQGFDRILRVLDLPPKRLGAPAAQQCYQDLTPPRPRYIPVAVRDKGAGRPTKKERRQLEALRGEEWARDRRR
gnl|extdb|pgaptmp_000271	K06221	dkgA; 2,5-diketo-D-gluconate reductase A [EC:1.1.1.346]	MVGFGTYKVADRAQEIVESALEVGYRHLDTAQMYGNEAEVGRAWTGSGLARSDLFITSKLDNPNHREPEFSASWQQTLEDLQTDYVDLFLIHWPLPMRYGGDFVTTWRQLEELHHSGQARAIGLSNFEPEHIETILANTEVVPHVIQVESHPFFHNERVEACAREHSIVFEAWSPLARGGTTTDPTLGEIGAKYGKSPAQVALRWGLQTGHVVLPKASTPARQRENLDLFDFRLTATEMALIDQLDRGEAGRTGGHPLTRG
gnl|extdb|pgaptmp_000270			MIRRRWMTGILLVALTATLSACTATAPRSGQSDAEGHPDQPRPTAPPLPPLTVLDASYPAAGVSVPGLRGQRILNSAGISARWADLPGAKEFNRKLAEVVKQQIEALTAELGTDYTPEAPPAPAGLSDRGCVPGSTFRSAEQIVSDPDLAPPGTALTIVCDPVLAAGPHFGERLRFIRSHGGQVTSDTVETLYTETGTDRVAREAELLDAERLPDLYQQAEKLAAPIGQLTGALTPPEGAPLLRDHVSAIQFGPDGSLLLTVDQGFFAAHPRREDAPPPTPFSLRLSPQLAQKYLTDLGRAIASSIAQPWAGTPQVNAGQAFVDCDLVPCLALTFDDGPSSNTPQLLQDLAAHDSAATFFLIGKNVAALPQVVADEVAAGHQLGNHTWGHPSLTKLPLEAASKEISDTTAAIVSASGVSPALVRPPYGDWNQVLQERIQLPFVLWDIDTQDWREPPEEDLIRSAATDPDPGDIVLMHDIHATTTAAIPKILAQLRDRGFTLVTIDQLFDGAV
gnl|extdb|pgaptmp_000269			MDLSDNYRNEAIVRMADGTVKQTNLLSGTEVWTVPGRGDRPLPSSLPAPDPIDYAQENSWCAFCQNRYLETPPEKSRLVLEDGRWKTLQALSADELFQTTAEFRRIPNLFEIVSFSYWMLNHNHTPSEAAHRRMAQYLASSTGYEHVMSVVKARILASGVSAEDFDRMSESEKLKYANGFFSGGHDVIVARRHYADGATDASQLAGSGTLTPEEHFQYLQMTVRAMQDLYQLNPAVKYVVAFQNWLKPAGASFDHLHKQLVAIDQKSVQTVAEIDRIRQHPSIYADILQVGATRQLLIAQNDYAIALAGFGHRYPTVAIWPLGKPVDPWEASEEQLRGVSDILHAVHAATGAEVPSNEEWYHRPPTTVTPVRWRILLKWRISTLAGFEGGTRIYLNTIDPWQVQARTVARLKELRDEGKIAPIRIGDECRVSAADLE
gnl|extdb|pgaptmp_000265	K00262	E1.4.1.4, gdhA; glutamate dehydrogenase (NADP+) [EC:1.4.1.4]	MNEKVQAVYEEVLRRNPGEPEFAQAVHEVLESLSPVIDRHPDYAEYALLERLVEPERQILFRVPWVNDNGEVKVNRGYRVEFNSVLGPYKGGLRFHRSVNRNIVKFLGFEQIFKNALTGRGLGAGKGGSDFDPHGKSDNEVMRFCQAFMTELSRHIGAMTDVPAGDIGVGAREIGYLFGQYRRLTNRYDTGVLTGKGLDWGGSLGRKEATGYGEVYFARDMMATRGQDLEGKVVTVSGSGNVAIYAIEKAQAFGAKPVTFSDSSGWVYDEDGIDLDLLKEIKEVRRGRVADYVAARPSAVLNTEGRPWQVPCDVALPSATQNELDEQDAIALVKNGCQVVAEGANMPSTPDAIEIFQEADILYGPGKAANAGGVAVSALEMQQNASRANWTFERVEDELAQIMVDIHETCLATAEEYGQPGDYVLGANAAGFMRVADAMKEQGVI
gnl|extdb|pgaptmp_000262			MIFQPLVSPVVLVLFAVLWVALTLLTVRARAPRRLALGALIGLILAGPSIPGEELEMTSNVEIYLAVDRTGSMAAEDWEDGQPRLAGVARDIVELVDDTAGARYAILTWDSTARLELPVTTDSSAVDSFAQVLHQELAEFSAGSSLSRPADLLLATLADAAESRPENKRFLVVFTDGEETDPEDFSPAWAQIANLIDGGAVLGYGTEGGGPMRTYPDGEYITDEEGNQELSYLDPATLEQLADTLGVPLLLNPTSLDQLDFMAEAQQIQDERNQQQTYRYLVWPLALGAGVLLLFKLRVLARDWRAN
gnl|extdb|pgaptmp_000261			MRYPWLTALILVAAGALLAWQLARRSEATRTWLANSATLFALPSFRTQQRRYRVLMGSAAGAVLAAVCASAVLAGAPVDRRVENPALARRDLVLCLDASGSMLPYDGQILDHMREVSAHFSGERMALQMWSAQTVVKFSLTDDYQLIDDVLAEAAGVIRRGYMGEEGEYVLVSNELFDYLHGVDAPDGEEIASLVGDGLASCVLGFDHRDQERSRTVILVTDNEVQGPQIYTLEQAVKFATDQDVQIIALYPSPDGALTSEGEALRSLVEGAGGTFYQADDPGSVEGIVADIQAQQLVEAEGRNRVVETDRPRTAALWLAGSSLLLLALLAWRRL
gnl|extdb|pgaptmp_000259			MLHDYGKLVSLPVQRRLLTQLAGMHRGARAGTSHEFLEMDEYKVGDDVGDIDWKATARHNQPVVKQFEATAILSAYLLLDAGANMAAAASLTETKKQVALEFVTAICWLMRGDHLGLVLGNQAEVRAVPARSGTAHSQQILRLAERAQVSGPAANLDRLLRYPLPHRSMVLLVTDAYQLNESTALALRRLQARHSVFVFLVADLDPTEAGPGPVRDVGGALIPQFAQTNPTVAYQWANLNRARLQWVEQVLNSVPHATAASRAEVLPALLELFDRHA

gnl|extdb|pgaptmp_000257	K01596	E4.1.1.32, pckA, PCK; phosphoenolpyruvate carboxykinase (GTP) [EC:4.1.1.32]	MSVTEQDVRAAAPSNAPEDVITWVAEIAALTQPDRIEFADGSDAEWDRLTSEMVDSGMFIRLNPEKRPNSFLARSLPSDVARVESRTYICCENPEDAGPTNNWADPKEMKEILHERFTGSMKGRTMYVIPFSMGPVGGPISQLGIEITDSPYVVVNMRIMTRIGTPAMDLIAEGKPWVPAVHSVGAPLAEGETDAVWPCNDEKYITHFPETNEIWSYGSGYGGNALLGKKCYALRIASTMARDNDWMAEHMLILRMTDEKSGKDYHITAAFPSACGKTNLAMLQPTIPGYKISTVGDDIAWMRPGPDGRLYAINPEAGFFGVAPGTSYDTNPMAMETMKANTIFTNVALTDDGDVWWEGIDGEVPAHLIDWHGNDWTPESGTAAAHPNSRFTTPASQCPIISEDWEAPQGVPVDAILFGGRRATNVPLVAEAYDQEHGVFVGATVASEVTAAALDAKVGSLRHDPFAMLPFCGYNMADYWGHWIKMGKLLGDKFPTVYQVNWFRKDENGKFLWPGYGDNSRVLDWIVRRRAGEVEAVDGVTGRYPKFEDFNIDGLEGFTKEDWDKLYTIDPEAWAAETEDSAEYFKQFGDKVPAGI
gnl|extdb|pgaptmp_000256	K07220	uncharacterized protein	MGKTKDRGPSFFEILKAQASQLVTAVDLLTTLFNAPEEERYLLRDRLHEVEHEADNLNHSIIRKLNQSFITPFDREDLQALASLLDDCMDLLDEAGDLLVLYNLHDIPEPVFSLLQAQMQVLNQCAFLTAENMPNLKRPQDMREYWVEINRLENDGDKAYRRTLKHLFDTGIDPITVIKLKDVVEVLEKCTDKFEDLGNAIESIAVK
gnl|extdb|pgaptmp_000255	K03306	TC.PIT; inorganic phosphate transporter, PiT family	MDLNLVLVVVVIAIALLFDYTNGFHDAANAIATSVSTRALTPRVALAMAAVMNVVGALLGTEVAKTIGEGIIDIYQYSNSTDVSIQRMGLIIVLAALVGAVTWNYITWWFGMPSSSSHALIGGLVGAGLASATQVQWTGILDHVVIPMVVSPVIGFVAAFLIMKGIMRWLANRPFQRTMRRFRYAQTVSAAAMALGHGLQDAQKTMGVIVMALLAGGYGETHNIFDPVTGDMQIPLWVKLSAAAAISLGTYSGGWRIMRTLGRRIIDLDPARGFVAEGVSAAVLYIAAFGAHAPISTTQTITSAILGAGATKRWSAVRWNVAGNIVVAWFLTLPAAALVAGIMFFLFHTVIPG
gnl|extdb|pgaptmp_000253	K23258	mutT1; 8-oxo-(d)GTP phosphatase [EC:3.6.1.69]	MVPRPMALPQRLIRSAGALVWRPVGDLPEPGTPLGPNDIQVLLVHRPRYRDWSWPKGKAELNEPLAQTAVREVEEETGELVYLQKPLTLQRYRLGSGQTKEVHYWAAQALGPDPAKAARPPVERAPSKEIDQARWCRPSKARQILTRRGDRRLLTELLQHGARGELTSSTVILLRHAKARSRKKWEGSEADRPLTRLGGMQALDLPPLLSAFGANHLISSPWLRCRQTVGPYAELTRLPIHTDDVLTEDADPRGATELMRSLLAERTGVHVVSLHRPGLPALLQPLLEGGLRSPGVEEGLRTAEMFVAHVSHGDQPRVLDLERHIPYTYLT
gnl|extdb|pgaptmp_000252	K00937	ppk1; polyphosphate kinase [EC:2.7.4.1]	MTSPDSVPEAPVEEAPPEISDWEPALPEELFLDATKLVGEEHQDEEYPIAEVDRLAELTQEEWEDEVSPILGLPLDRSKPMGLDHPLPDGRFADRELSWLAFNERVLEQSEDPSVPLLERLWFSAIFSSNLDEFFMVRVAGLQRRIAAGIAVPTASGLAPREILDLILRRTRELTTRQAEDFLEDLLPALDDNGLHLRSWNDLDEAQRERLATYFRRRVFPVLTPLAVDPSHPFPYISGLSLNLAVVVHNPVSGKRHFARIKVPETLPRLINVDTANTDGNPEDHVRQRRGATFIALEDLITAHLDHLFPGMEILEAHTFRVTRNEDLEVEEDDAENLLTAMEEELSRRRFGQVVRLEVTDSITPFVRNFLIDRLEISAGDVFQLPPPLDFTAFNQLHDLDLPALKYPPFVPVTPAGLSEVESSQPGDIFASIRNHDVLLHHPYDSFATSVQQFITQAARDPHVLAIKQTLYRTSEKSPIVSALIEAASLGKQVVAIVEIKARFDEDANISWARQLERAGVHVVYGVLGLKTHCKLSLVIREEADGLRRYCHIGTGNYHPGTARGYEDLGLLTADPSVTQDLTRLFNQLSGYAPKSTFRRLLVAPVSIRSGLIERIEEQMARRLRGEDAWIGIKVNSIVDEATIDALYRASQAGVKIDIVVRGICALRAGVKGLSENIKVRSILGRYLEHSRIYAFGVGEQQEAWIGSADLMHRNLDRRVEALVSIEDPEQVAYLVTLIKTGVSPRISSWQLRKSGAWKRRTHDKDGAPLQDIQELLMDEASARVVGR
gnl|extdb|pgaptmp_000251			MEARRFRRPAMLDPKEAEEIMGDGDPAEEAEIAHTSAWALMGVPEGDFDQEHVDRLVEVVQSQGVDAIAGLWDRSSEITLAGALWRLYLLWQWHQLDPATVDERFDEGREALLAQGETPDSIPPLGDVLRGLEIVLGGRASEDQLSPVLEVAARTLEVMAAGVSAGPRWIESDEHELAHPVTRRPRALLDTAQELRESAR
gnl|extdb|pgaptmp_001701	K07776	regX3; two-component system, OmpR family, response regulator RegX3	MNQRILLVEDEESISAPLVFLLEREGYRVETVADGNEAVARFSDGEFDLVLLDLMLPGQPGTEVCRAIRAQSSVPIIMLTAKDSEIDVVVGLELGADDYITKPYGARELIARIRAVMRRRGEAEDPVSADEVLDEAGIILDTERHSLTVRGEDVAIPLREFELLEFLMRNSGRVLTRGQLIDRVWGSDYFGDTKTLDVHIKRLRSRIEADPAHPELITTVRGVGYRFR
gnl|extdb|pgaptmp_001700	K07768	senX3; two-component system, OmpR family, sensor histidine kinase SenX3 [EC:2.7.13.3]	MSAERDPRDRHLAPAPAGTPEPSAPSSPVEVTSSYPVAKAILHELDSFAVILGPDLQLLYANPAARQSTHVSGIELSDSRFLAQAKQVLNTGTATSHVNEDETPVRIQFIPLPDRHLVVLVEDLGEEQRVQAMRRDFIANVSHELKTPISAISLLAEAVVEGADDPEVVREFSKQLLKQARRLGNLTRDIIQLSEAQSDLRPEDRQPVDLRELVVQEVEAHRDLATGQDVSVELVLPEPASRPAITAGVPSALSTAVDNLLSNAIRYSPPGSRVRVTMTFEKDTLMVTVADQGEGIALEHQHRIFERFYRVDRSRSREGGGTGLGLSIAHHIMRGHGGAIDLWSRPGEGSQFTLTFPLLRPSQLPADSQKRDPDEPANPAS
gnl|extdb|pgaptmp_001702	K02040	pstS; phosphate transport system substrate-binding protein	MKNQVKLGALSGAALLALAALGGCAANEVGVAPTEQSSASGADAAADSNLSGELIGAGASSQGAAEEAWIAAFQSAHPGVTVNYDPAGSGAGRETFQQGASAFAGSDRAFKLEEIEAGPFQSCAPESDIVELPVYISPIALIFNIEGVDSLNLDAATVAKIFTGQITKWDDPAIAEHNPGVKLPSTNITAVHRSDDSGTTGNFTDYLSAAAPSDWTVGSIETWPTEFGGEGAQGTSGVVEAVSRGIGTIGYADASRAGELGTVALKVGEEYVSYSPESAAAVLDASPLEPGRTDHDLAIDLDRTTDQAGVYPLVLVSYLIACQDYPSAETADLVSNYLTYVVSAQGQEAAASAAGSAPISEDLRAKLQTAISAIGAK

gnl|extdb|pgaptmp_001703	K02037	pstC; phosphate transport system permease protein	MYPVISIAEGSKSGSSARGEGLFAGAGRLAAVTVVAILAAVGAFLVVRSVPALVAGPAQASLLDGNFVSYVLPLLFGTVWAAIWALVLAVPVAIGVALYLTHYAPPRLRAGLGYLVDLLAAIPSVVFGLWGITVLAPALQPIYVWLNEHLGWIPLFSGTVSGTGRTILTAGVVLAVMILPIITALSREVFLQSSRSVEEAALALGATRWEMIRLAVLPTSRPGMVSAAMLGLGRALGETMAVAMVLSATGTMTLQLLTSQNPSTIAANIALSFPEAYGTNINVLIATGLVLFATTFLVNAAARKILSRSER
gnl|extdb|pgaptmp_001704	K02038	pstA; phosphate transport system permease protein	MAIETTVLSPTPRPLVPPQVTAPLRGPSRPLPRSASTAIIGGAIALSVATAWLGGTREPNPAALLLPAALGALVGMTVTMVLSWRVEGRRASVDRLATTLVTGSFLLALVPLGSLVLTVISRGLGRLDAEFFTHSMRNITGAGGGALHALVGTLLITGAAALIAVPLGILTAVYLTEYGSGRLARSVTFLVDVMTGIPSIVAGLFAYAAFAFILGPQARMGIVGAVALAVLMVPVVIRSSEAVLRLVPADLREASLALGVPKWRTILKVVLPTAAGGLATAVMLSIARVVGETAPLLITAGFTASMNYNLFSGRMQSLPVFVYTQFANQGTPAEAFLDRAWTGALILIVLVMALNLCARLVARRVAKQR
gnl|extdb|pgaptmp_001705	K02036	pstB; phosphate transport system ATP-binding protein [EC:7.3.2.1]	MSKSITIDNLNAYYGNFLAVEDINMHMAPRTVTAFIGPSGCGKSTLLRTINRMHEVNPGARVDGKILLDGEDLYDPAVDPVRVRRQVGMVFQRANPFPTMSIRENVLAGARLNNRRLPKDEAEQLVEGALRGAGLWDEVKDRLGKSGSSLSGGQQQRLCIARAIAAGPQVLLMDEPCSALDPISTATIEELIAELKQHYTIIIVTHNMQQAARVSDKTAFFNIAGAGKPGKLVEYSDTDLMFSNPLVEATADYVSGRFG
gnl|extdb|pgaptmp_001526	K01421	yhgE; putative membrane protein	MRGPKLTGNAFANILVILAIAFIPLMYGGLLSSAYQSPIEHLDHLRAAVVNEDQPATANLISGQRQTLDVGEMLVDTLTDPAPGQDVGFTWEELGRADAEAQLADGTLAAILYVPRSLSADVIQIGTSAAPNARPAQLELVTDDGVNYLTGTMARTVATALEDEIRATASKTYVDAILGSFGTIRQGMVSAADGADQLNTGADQLDRGLGELNSKIPSLSDGASQLSSGAEQLAGGANSLNSGLDAYTAGVDQAAAGANTLRGKTGQLQSGIEQLRAGSAQVAAGNKKLDDGFHQISDPVSELAPLPDELLKLVQDTAANLDELSAQCQQDYPGSDLCLFLEDLQSHQGEITAKAGEIADQANEAIGAVGQLQGSLDQLADGSAQLESGLTTLQTQVGSTSDGAANQTLIGGINALSSGLDQLSANSEQLRAGGGQLSDGSRQLAAGTSELNSQVPTLAAGVGQLDEGAGRLHDGTEQLAGALAEGSDQVPDYNHQDQDSIASTVSQLVGVEPIRANPVANNGAGFSPMFLSLALWVGAIAIFLVLPALDRGVRPGEHWALQAIRPGSTAALMAMIQAVIAVVLTNFSVQMEAKDLAGFVLVAVLASLTFAVINQACIAVLAYRGRFVSIVLLVLQITSMGATFPVETMPAFFQWIHPWLPMTYTQLSFRALIAGAGVPNIVWHTVGVLLLWWVVAVLVILWAAHHRYQKRPLPYDQALLPDNYPLDEDASPEELARRRDLKTEVFADWRAARHAVEGRFIMDRQRRGATTAVLEPSAPDEPNSDPEAPAADEGDSPTPTDQG
gnl|extdb|pgaptmp_001089			MNVVSLTELAQEQLAKAADASNGRSAVTIYGGHEHHLRQTLIAIRAGEKLADHANPGEATLQVLVGQVTVRSGSNETVLQMGEYLVIPDAVHSVHAEEDSAVLLSVSVRRR
gnl|extdb|pgaptmp_001632			MTSSVQLLAQPGPWQQLQYLQSELGEAATDPTTWPKLGQALRQAGHDPELVVALVSQLQLRAEARGKFGESAQHMLFTRAGLEQATRFQVSLLHALRYANAGVKRVADLGCGIGSDALALATTGLEVLAVDRDPEALAAAAINLREFPKTQVLEADVMTMPRPEVDGVFLDPARRSHDRRLFRPEQWSPPWDKVTEIFSWGLAAGVKLAPGIDHHYLGIGSHAQWLSYDGALVEASLWSTQLAPEGPGRSALVYHHHTIDVLSEECAPDAPLTPAPVGALEQYLFEPDPAVIRAGLLARLADLTGTHLVHPKIAYLSGPSPTDSPYWTGFEVLAEVALRPKVVKRQLRELGAGPVEVKKRGADVDPAQLQKQWQQKAGIPVVVFATRVGSSHRAIIARRLKG
gnl|extdb|pgaptmp_001931			MDKEILTWTEFGEASRELARQVRDSGWEPDLLISLARGGLIPGGALAYALDLKTIGSINVEFYTGEGTTLAEPLLLPPFMEVSADLGGRALIIDDVADSGKTLKLVVDLLSKQGVRDPAGELVRFEVRTAVLYRKSRTIIEPDYCWRSTDRWISFPWSTLPPV
gnl|extdb|pgaptmp_000945	K01554	8-oxo-dGDP phosphatase [EC:3.6.1.58]	MVQDRAQSGFPVESSEQLWDGAVFGLREDRVQLPGGDRPVVRQYLTHPGAVGIVPVRFTGSDPLDAELLLLRQYRHPVRAELWEIPAGLLDHPGEEPLAAAKRELREEADLGARTWSVLVDLFTSPGASEEALRIFLATDLFAYEEAFARAEEEAQLVPHWVDLRSAVAGVLAGDLHSPSAIAGILATQATLLQPGFGRREPAAPWLRKPRN
gnl|extdb|pgaptmp_000211			MVEYRYLGSSGFKVSEVTLGNWVTHGSQVGSEVAIATVHKALDLGITSFDTADAYANRAAEQILGEALRGQRRQSLEIFTKVYWPTGPGGANDCGLSRKHIMDSAHNSLRNLGTDYIDLYQAHRYDYETPLEETMVAFADLVRQGKVLYIGVSEWTAEQIREAAQLARELRIQLVTNQPQYSLLHRVIEAKVIPTCEELGMTQIVWSPMAQGVLTGKYLPGQPAPEGTRATAQVAESKFIQKFMDDQTLTAVQQLRPIADSLGLTLAQLAIAWVLQNPNVSAALVGASRPEQLEDTVRASGVSIPVEVMEQIASITEPVANLDQQDTYTVSPASRPC
gnl|extdb|pgaptmp_000209			MTQSGPNETLLGEEAIADALNQPPERASLFGLVSLAFDQAKTLLTTQIELAKLKVTKAAKKFGAGAGLAVVGLILLFYFIFWFFRTIEMLFLLIVPAWAASLITLGIILLLMILLISVGALLINRGTKDVPDVGGEIQANVDAVKEGLGK

<table>
<tr><td>gnl|extdb|pgaptmp_000206</td><td>K05571</td><td>mnhG, mrpG; multicomponent Na+:H+ antiporter subunit G</td><td>MTLLGAILILVGAFFTLVGAVGLFRFRTLYARMHAATKPQMLGLLCLLSGLALTIRTWQAVLGCVLILAIQMVAAPVASHLMGRSAYRQGLADGPELVVDELAADTDD</td></tr>
<tr><td>gnl|extdb|pgaptmp_000204</td><td>K05569</td><td>mnhE, mrpE; multicomponent Na+:H+ antiporter subunit E</td><td>MTESRTRPTPAQLRAHRQALANRRPRLHLALDLTIWLLFVWMAAFGSLEPLALIGALLAAVAVQWLFPLPNRAGIYQVHLLSLVWLILRFIWDMARAGLHVVWLIIANPPRHDAILRIQVRTSVPEYLALLVAMTTLVPGTVVVEVKAKERVLYLHCLDVEGQGGLEALRANTLAQEARILRAVAPRELQREVGVSRGRG</td></tr>
<tr><td>gnl|extdb|pgaptmp_000203</td><td>K05568</td><td>mnhD, mrpD; multicomponent Na+:H+ antiporter subunit D</td><td>MNLLHEWNWLLPLPVLIPLLSAGLALVVGRRPRLQQLISVTALGLSLVIGVILVLGAAVAPMALDVGSWAAPIGITLVADKLSALMLLISQVVTLAVLLYTVGENFSDSSPNAPVAIYHPTFLILVAGVSNAFLTGDLFNLYVGFEILLAASFVLITLGGTRGRIRAGTVYVVVSLVSSAIFLTAIALVYGAVGTVNLALLAERLPEIDPGTALLLQSLLLVAFGIKAAIFPLSAWLPDSYPTAPAPVTAVFAGLLTKVGIYALIRLQFLLFPQNPLTDLLGVLGLLTMLVGILGAVAQDDIKRLLSFTLVSHIGYMVWGISLATPASLAAAIYYALHHILVQGCLFLIVGLIERHSGTTSGRRLSNLARTAPVTAIMYLIVGFNLVGVPPFSGFIGKLGLAEASVQVGTPMAWALLAGGLVTSFLTLYVVVKFWNRAFWQTPDSGSELAINYEQREHLSGREERRLRRAVRAARTQRRAYRLTELSRTEGERGAGHSNPIMYGAAIGLIVVQLGMAFGAGPIYGYVTEAAREIVGGDVYVEAVLGTTGRGEGISNDVGTPATPPWETDPAPSVPVPRPTEGSGHD</td></tr>
<tr><td>gnl|extdb|pgaptmp_000202</td><td>K05567</td><td>mnhC, mrpC; multicomponent Na+:H+ antiporter subunit C</td><td>MPSLAMLLLAGALVGTGVYLALERTLTRVFIGLSFITNGVNVLILAMAGPAGLPPLLWGDWGQIADPLPQALILTSIVLSLGTTAFGLALAYRSWRLTGHDEVVDDIEDRRLARLAQRQRRRGEDYGVEGTEDPGVDYDRSSEGSDQ</td></tr>
<tr><td>gnl|extdb|pgaptmp_000201</td><td>K05565</td><td>mnhA, mrpA; multicomponent Na+:H+ antiporter subunit A</td><td>MFFVLGLHLLAAICAPLLVRWWGRQAFLPLALAPLSAVGWALAHTQQAFHQPVVEQLNWVPALGLTVAFRLDVLSWLMMLIVGGVGALVMVYASRYFAPDARSLGRFAGVFVAFAGSMLGLVTADHTMAVYMFWEFTTVLSYLLIGHHHQRGPARAAARQAILVTTSGALAMFAGLVMLGLAPGGSFRLSELVANAQSGVLATHSPLVVTAAVLVLIGALTKSAQFPFHFWLPGAMAAPTPVSAYLHAAAMVKAGVYLVARLTPGFAEVPLWSPVVVTFGLITMLIGSYRALRQYDLKLILAFGTVSQLGLMMAAVGFGTQEAMAAGLVLLVAHSLFKSALFLTVGAVESSTGTRDLRELSGLWRHKPVLAIGAGVAALSMAGLPLTTGYLGKEALLTDLLHRSWPVLVIVVLGAMLTLAYSWRFWWGAFATKRLRMERPIQPVSPMMRVPILLLAAGALLGLAPGWLELVAAPPAQGLPGSVHLAWWSGWGPGLATLLIVAGGLALILNRPKVARWQRQLAPRGGLVRVYTWSLVELELVAAQVTSLLHRGSLPGELSTIFVTMVVLAAVALTRVDVPSQPVVLWDSPVQAALVVLAIGAALIAARSLRRMKAVLALAATGMLITLLFATQGAPDLALTQLVVEAVSIVVFVLVLRNLPPYFSKRPLSLSRWWRGLVGASVGVVVAVGGWVAAASRIHEPVSNLMPEEALGFGNGQNVVNVILVDMRAWDTVGELSVLLVTATGVASLIYLKSRSGQIDKAPPAALAEGQYLPGAAALKPQDRSLVLEVATRVLFPAMLVLSIWLLLVGHNNPGGGFAGGVVAGLAFVLRYLAGGRFELGEAMPFPAGRLLGFGLFVAAAGGAAPLLFGNAVLQSTPVDLTLGPLGDLHFTTAMILDIGVYLLVI</td></tr>
<tr><td>gnl|extdb|pgaptmp_000200</td><td></td><td></td><td>MAPTPMVLTAGALTIIMTLVGLWFFGRGVGQIVGIITAGQPESGRWHHPWGRAGRALWQTLSHQTFRGRPLVRAAHWLVMVSFVVLVVTLVTSYLQVWRPDSVLPFFGTWAPWNWLTEFFAWAGLLGIVALMLVRLRTGSSERTSRFFGSTRWQAHFVEWVILLVCLAVLILHGLQARGDTFHFPLTAWLGSGFADLSPSTRASLIVLVSTGKIVVSMAWMIVVGLQVSMGVSWHRFLAPLNLMTSREDDLSKSLGALAPIPTVDGELDEDHLGLGTVDDLTWKDRLDVLSCTECGRCQDVCPAWNTQKPLSPKLLTLAVRDRAAQTHSGDILGALLAAGVSQTDGDQLVPDVLGPDMIWDCTMCGACVEQCPVDIEHVDRIANLRRFQVLMESAFPRELRRPFKGMETKGNPYGLPAKKRLDWAKDLAFDVPVIGEDLEDATEVEYLFWVGCAGAYDDRAKKTTAAVAELLHTAGVSFAVLGSGESCTGDPARRAGNEVLFQLLAEAAIDTLNEAKAQRIVVTCAHCFNTIANEYPEMGGHYEVIHHTQLLNRLVREGALTPTAPPEGQRITYHDPCFLGRHNRVFSAPRELLGSMLPVIEMPRSGENSFCCGAGGARAWMEETRGTRIATNRLQEAADTGATTVATACPFCTQMFDSASASVTGVPAPEVKDVAMLLLEGVHRSQAQDQA</td></tr>
<tr><td>gnl|extdb|pgaptmp_000198</td><td>K01494</td><td>dcd; dCTP deaminase [EC:3.5.4.13]</td><td>MLLSDRDIRRGIEAGRIDLDPLDLDLIQPASIDVRLDRYFRLFDNHRYPVIDPAQEQPGLTREVVATGEPFVLHPGEFVLGSTYERVTLGDDIAARLEGKSSLGRLGLLTHSTAGFIDPGFSGHVTLELSNTATMPILLHPGMKIGQLCFFDLSSPAEHPYGSAQLGSHYQGQRGPTPSRSYLRFELTVLDEDELEAPPPTEQDRAK</td></tr>
<tr><td>gnl|extdb|pgaptmp_000190</td><td>K03671</td><td>TXN, trxA; thioredoxin</td><td>MSTIEVTQENFEATITENPIVLLDFWAEWCGPCRQFGPIYEKASETNTDIVFGKIDTDAEQELSQGAQISSIPTLMAFRDGIAIFRQAGALPGPALDDLIRQIRELDMDQVRADIAAANGDPV</td></tr>
</table>

gnl|extdb|pgaptm p_000189	K06187	recR; recombination protein RecR	MFEGAVQSLIDELGTLPGIGPKSAQRIAFYLLAEENEEVERLIGAIKDVKTKVKFCTQCGNVTESEICSICADPRRDETVICVVEEAKDIQALERTRVFRGRYHVL GGAIDPLSGIGPDQLRIRELYRRLADSTVTEVILATNPTIEGEATATYISRLLAPLEVSISRLASGLPVGGDLEYADEVTLGRALEGRRSL
gnl|extdb|pgaptm p_000188	K02343	dnaX; DNA polymerase III subunit gamma/tau [EC:2.7.7.7]	MAVALYRRYRPDNFDDVVGQDHVTELLKNSLDSGRTSHAYLFSGPRGCGKTTSARILARCLNCAEGPTSTPCGVCESCVELAAGGPGSLDVVEIDAASHNGV DDARELRERASFAPTRDRYKIFILDEAHMVTSAGFNALLKIVEEPPEHIKFIFATTEPEKVIGTIRSRTHHYPFRLVGPTVLLPYLQSLCEREGITVEDGVLELVIRA GGGSVRDSLSVLDQLIAGCGGQSVSYAQATGLLGYTDTSLLESMVAALGQGDGARAFQIVENAVDSGVDAIQFTKDLLQLLRDVLICALVPSQAKEVLPHVP QDRLTRMLEQAQAWGTRALSRRADQIDEALRNMSGTASARLQLELLLGRMLAEDPSAEHEAAPAPAPAPARTSAPAPVERVDPASVAPVQHKTPAPASE RPQGNDRRQAPPAATRPAAPPTPSQSEPRRPAAASTGSSNKTLAEQWAQVGTIVGQVSRVFEVVVKGTTPVELDGATLRIAPSASVRNRLLSGGAELTAISA AVKQVFGPAVEVEVGEGKRATPSTSPTSTPAPTSAPSRPDSPSRPTPPVAVEPEPGLAVAPDPTPGSTPQSAPEPTPQPAPRPAPELEREPEPPAPPAYDPRD DEVSLHDPTVETAPNRGVPAILDVLGGTLIEEIEEER
gnl|extdb|pgaptm p_000402	K03313	nhaA; Na+:H+ antiporter, NhaA family	MSSPESTNRPKVIATFLSPGSEREERRILRILRAETVGGVLLVLSALVALIWANSPWSDSYFALRDFEIGYAPWNLKLSLGTWAADGLLAIFFFLVGLELKREFVA GDLRKFSTAIVPMAAAAGGVLVPALIYTLVVRNYPNLHHGWAIPTATDIAFAVSVLALIGSALPSAVRIFLLTLAVVDDLIAIFIIAIFYSTDINYVALLISFAVIALY GAIAQYRRLHFALKPATAWYILLPLGVVAWAFMHMSGIHATIAGVLLGFTIPVKPPRGLPAHGNTGLAGQFEHRFRPISAGVAVPLFAFFSAGVAVGSFDGFI HTLTSPSALGVMAGLVFGKPIGISLTTWLITRIGPIRLDPSVKWLDLFGMSVLAGIGFTVSLLVSELSFEAGHAAHAETKVAILTASVIAALLSSLILVPRNRLYRR
gnl|extdb|pgaptm p_000177			MSTPENNAPAPQHLPGPHKSPRQIYRETMARRQMRIFSIIIVFLLAALALAALTLTGIIPTPFGNEFSKKIDYAEMGDTPCPTEDARPVSVAGAQLQVLNASSV SGLAGSVAGTLEEMGYGIALVDNASDPFMGNVQLDVGPASVDLAYTIARYFEAPVRIKLRELPIGTVSITLGEGFQGLLPADDLTALATSTSRLQPLRECLPVD PKSVPETQQSGGQQSGPQSGSQSGEGAQSE
gnl|extdb|pgaptm p_000176	K08744	CRLS; cardiolipin synthase (CMP-forming) [EC:2.7.8.41]	MPDSPSSSANQGGMSTAQPEMSDRIWTLPNLISISRLLLLLPLTVWFLVQQNYVGALWSLVALGITDWLDGFLARRLNLVTRFGKNLDPLADRLSVLAVVW ALIVDGILPWPYIVVIAGIDVTVGLLGLAWFGGPPDIPVSRVGKARTAGILVSLPLLILEAATGIEWIGVVGLVILTFSVIGHVWAGLGYLRIMHALHRTRLAEVA AAKETQQ
gnl|extdb|pgaptm p_001903	K01990	ABC-2.A; ABC-2 type transport system ATP-binding protein	MPTYPIVIEHLTKRFGSVRALEDVSFTVTPGRVTGFLGPNGAGKTTTLRILLGLDQADSGQALVGERPYRDYPSSALVVGASLEADSFHPGRSGRNHLRVYGA AMGADPERIDEVLSMVGLTGAAGRKVGGYSLGMRQRLGLATALLADPPVLVLDEPANGLDPEGIRWMRELLQYLAGQGRTVLISSHLLGEVQQIAQDIVII NRGQVVAAGDLREIEAGRSTSVLVNSVDNAALARALHAAQLSASRAGNQLAVTGATPEAVGAIALQAGVALTHLSQSQDDLEAMFLELTGGAR
gnl|extdb|pgaptm p_001902	K01992	ABC-2.P; ABC-2 type transport system permease protein	MTRFAQALRAEWLKIFTTKTWWILTLIMVVYLGFTALLLGATLSTVEADPIMVYTTASTIGFIFPLLLGTLAVTGEFRHHTIIPTFLTVPKRGLVLAAKLLVHFVLG AAMGVIAFATTIGAGAFFFSGQTGLDQAETWQIIGRCLLVMALWAAVGVGVGSLVPNQAAALVVVIGFTQFLEPTLRLVAAVQENLAPIGKFLPGAASDAL GGASFYSSFSSMTGSGAATGLLWWQAGLVFLGYGALAVGLGYWLRWRRGV
gnl|extdb|pgaptm p_000175			MQSPLSSILRWKVHGTGTSIAPGEVVLPRERLSWPRTVGIGAQHVVAMFGATFLVPLLTGFPPNTTLFFTAIGTILFFLITAGRLPSYLGSSFALIAPILAVSQTLG ASYALGGIIATGATLALVGIIVHFAGIKWIDLVMPPIVTGAIVALIGFNLAPAAWNWVKEGAVTALITIVAILLITVLFKGIIGRLSILFGVLIGYVAAVIQHEVDFT AVQQAAWFGFPEFHSPQFSASTLGLFIPVVLVLVAENVGHVKSVSAMTGENLDDLTGRALFADGISTMLAGSGGGSGTTTYAENIGVMAATRVYSSAAYVV AAGFAFLLSMMPKFGALIATIPAGVLGGAATVLYGMIGMLGVRIWVQNRVDFSDPVNLNTAAVAMIVAIADYTWAPGGMVFGGIALGSGAAIIVYHSMR GIARWRGTNLEAATPASAPSGTELDSEAYARRHRVSDGVDGVLPVEDARETND
gnl|extdb|pgaptm p_000174	K01951	guaA, GMPS; GMP synthase (glutamine-hydrolysing) [EC:6.3.5.2]	MKPFLLVSTRPEEEALVSEYQAYLQSTGLSEDRLELAEFDLVGLPPVELGDYQGVFVAGSPYGNITAGTATSKTQRWVAEELREFFRQVLAAEIPCLATGTAM SLLTELLGGKVADEHMELSEVTWVTQTREGMEDPLLAGVAEDFLAYVSHTEAARELPAGAVRLAWSPNCPVQMFRHGQHIYATQFSPELDGEAIQRKTEM YADAGDFRVGDLDMLVGTGRHRTGGQQSALILRNFVKQFS

gnl|extdb|pgaptmp_000173	K04069	pflA, pflC, pflE; pyruvate formate lyase activating enzyme [EC:1.97.1.4]	MTDIAIAGLQPLSTVDWPGKLAAVLFLQGCPWSCPYCHNYQILDPATPGAVPWEEVQRLLAKRQGLLDGVVFSGGEATRQRAVVEAAAEVKEMGFQVGLHTAGAYPSTFARLLDQNLVDWVGLDIKALPGDYQGVAGPAVSADKAQQSLELLLKSGVDYEVRFTLWQGGLDYAEKVAAWCQSQGVENFVLQRLQTQHLPPNYNPGPEVTNWGQAQAEEMLKQYEFPQSLVRV
gnl|extdb|pgaptmp_000172	K21636	nrdD; ribonucleoside-triphosphate reductase (formate) [EC:1.1.98.6]	MSRYDVDAIGTIDEYLERADWRVNANANQGYSLGGMILNSAGKIIANYWLEKVYSPRAAAAHRRGDLHIHDLDMFSGYCAGWSLKRLLEEGFSGVPGAIASTPPKHFSSAAGQVVNFLGTLQNEWAGAQAFSSFDTYMAPFIRLDNMEYDQLKQQMQELIFNLNVPSRWGSQCPFTNLTFDWTCPDDLKDEHPVIGGEVCDFTYGDLQEEMDLINRAFIEVMSGGDSNGRVFTFPIPTYNITPDFEWDTPNVNALFEMTAKYGLPYFQNFVNSDLNPGDIRSMCCRLQLDLRELLKRGNGLFGSAELTGSVGVVTVNMARLGYRFKNDMRGLLDELDHLIDLASGTLEAKRVVVQQNIDNGLFPFTKRYLGTLDNHFSTIGVNGMNEMVRNFTDDKHDLTDEFGQEMSIAILDHVRERMVELQESTGHLYNLEATPAEGTTYRFAKEDRKHYPGILQAGTDEQPYYTNSSQLPVGYTDDPFQALEEQEVLQSKYTGGTVLHLYMGERISNGEACKQMVKRSLTAFKLPYITITPTFSICPVHGYLNGEHFLCPECSEPQECEVWTRVMGYFRPVQSFNIGKKGEYNERTMFTESAANEHGELVST
gnl|extdb|pgaptmp_000171			MGQLPPGFQAAARRRRSLVVLLSAISLIALSYLALYTVAGQNIDNLTMEAVAASARWLHLRLGAVSAAVSIPALGAISIGVVAVAIARRAALAWRALLVVGGANATAQLLKEILPRPELGVGIQLENSYPSGHVTYAAAIAVALIMVAPRGFRSPAALVGWLWTTLMGLMVISQGWHRLADVVGALLIVALWGFASAPAELRPRVQPGLTRAGTYLAAATSAIGVTLFVVSVIIITPEVFAPLSYGEIGEMVEVGTREGVLFSLATLLLLGGLGGLLLNGIDRLSEAR
gnl|extdb|pgaptmp_000169			MSSAPLHLAPEGAGDPYPLTCRRLVHALNELGYSTERHPGSNRVDFEADAHQLRAYWHPGKDILAIQTRWDSEHPYRSAEYALFAAADNWNRESYFPTVYLLESPDHTALVVADMIAPCSMGLSDRQLSEYLDTGVTQGLRAMNYIRSVAERVLGLGHPHHG
gnl|extdb|pgaptmp_000168	K06877	DEAD/DEAH box helicase domain-containing protein	MANPASPRLREVLGQLDSSTGPEQERHLVAEVHLESRPAQTEAWPSSIHPEVQAAFRAGGAERVWSHQAEALTALAGGADVVLATGTGSGKSLAAWAPVLSALADFSADRPVSEAWRLSELRHKPTALYLAPTKALAADQLTHLNQLASHLTIPVRLSTADGDTSREVKDWARAHADIILSNPDYLHHVLLPGHQRWVRFLSSLRYIIIDEMHYWRGLTGAHIVLVLRRLQRLARHYGADPQFVMLSATIANPLEVGQQMSGRERVVAITEDGSPQGAKHLVFWQPGVRDNPAGDEAEADDLAPTLPTIRVSATTEAAALCSQFVAEGARVLTFVRSRGAAETVAAQVQDRLSWLGWSGQQAAAAYRGGYLPEERRELEASLRSGQLRSLATTNALELGIDISGLDATITAGWPGSRASLFQQMGRAGRAGTDGISILIAGDNPLDQYLVHHPEEILTEPEANILDTHNPWVIAPHLCAAAAELPLRADELPAFGLSSADLLNQLCQQDYLRYRSDRWVWNATLPDSAHSLTSLRGEGADIQIVEVDSGRVVGTVPSNRADAELFPDAIYVHQGHTYHVLELSPFTSDGTQRVAVVEKLTTRFRTRTASHKSVTIMSTEDTWTSPDGLVTWHYGSVDVGERVTDYDLLRLPGLEFISNHELAFPERFLPTMATWYTLDRRALREAGIGPEDLPGALHAAEHAAIGLLPLLAMCDRSDLGGLSIPEHQQTELPTVFVHDGYPGGAGYAQHGFHHAHQWVSLTAQALSACPCEDGCPACIQSPKCGNRNHPLSKPGALALLEFLVTHSPVG
gnl|extdb|pgaptmp_000330	K06901	pbuG, azgA, ghxP, ghxQ, adeQ; adenine/guanine/hypoxanthine permease	MSTTDTEKQVQPANGALDRFFKISQRGSTVSREVRGGLVTFFAMAYILVVNPSILSAAAPESGPITTAAIAAGTALIAGLLTIAMGVFANFPMSMAAGMGLNAMVAFSLVGGEGLTYQEAMGLIFWEGLIILVLVLSGFREAVFRAVPQQLKSAISVGIGLFIAFVGLANAGIIRAGAGTPVQLGADGHLSGWPMAIFIFGLLLVIVLYVRKVKGAILFGIIGATILAFIVQAVAKVPGAGDDSPTGWMGTVPEFHGSPVSLPEFGTLGQVDFFGAFEKLGVLSVILLIFSLMLADFFDTMGTMVAVGAEGDLLDESGTPPHSARILMIDSLGAMAGGLGGVSSNTAYVESTAGVGEGARTGLASVVTGLLFLLALFFAPLMTLVPAEAASTALVFVGFLMMTQVTEIDWSRQEMALPAFLTIAMMPFSYSISVGIGVGFVSYTVIQIARGHARKIHPLMWVVAALFVVYFAMTPIQQLLLG
gnl|extdb|pgaptmp_000323	K00111	glpA, glpD; glycerol-3-phosphate dehydrogenase [EC:1.1.5.3]	MANGEELDVLVIGGGVTGAGIAVDAATRGLNVGIIEAQDWSSGTSSRSSKLIHGGLRYLYQLDFKLVHESLRERGLLLIQNAPHLVKAQPLLWPLKIPVVERTYSAVGVGMYDAIAQAAHPGAVPIQRHHTKKGSLKLCPALKDDALNGSIVYYDARVDDSRLVITLVRTAVRYGAKAASRTQLIEILKDNRGRVNGAVVEDLETGERYTIKTKAIINATGVWTEKTQALAGTTGGLKVLASKGIHLVIPKDRIKSEVGLFLRTEKSVLFIIPWKRYWIIGTTDTKYHESLLNPVADEADIEYLLEHANSVLKDPLTKDDIIGTYAGLRPLLQPGTLDGDAAKSTKVSREHTVTEAAPGLTVIAGGKLTSYRQMAEDAVDFMLGKERAAKQPCVTGKTPLEGADGYRALWARREAMARETGLSVDHIEDMLDRYGNNIDVIFDMIKDTPDLGEPLAEAPEYLRAEVAFGVTHEGALHLEDILLHRIRLVYEHRDAGLAAMPEVADIMGHYLGWDEDTKEREMDLYRRACEAVREAELITDESAAQAVRDEVEPIRPMLDV
gnl|extdb|pgaptmp_000258	K03924	moxR; MoxR-like ATPase [EC:3.6.3.-]	MSYLTPEETATATDLIGRIRQIFAQRIVGQLELRDSLTAALMAGGHILIESVPGLAKTTAAHTLAQAVSGSFRRIQCTPDLMPNDIIGTQIYLPTTGRFETQLGPVHANFVLLDEINRSSAKTQSAMLEAMQERQTTIGGEHFPLPDVFMVMATQNPIEEEGTYVLPEAQMDRFLMKVVISYPTPAEEMSILQRVGEEPELNDPLTLAEVEQLQELTSRVFVHDSIKSYIVDLVNTTRGSGPAPLPGFTHHVRLGASPRGAIALQQVAKAHALQLGRSYVVPDDVKALRYSVLRHRLIRTWDAVADDVS
